# Design, synthesis and application of carbazole macrocycles in anion sensors

**DOI:** 10.3762/bjoc.16.157

**Published:** 2020-08-04

**Authors:** Alo Rüütel, Ville Yrjänä, Sandip A Kadam, Indrek Saar, Mihkel Ilisson, Astrid Darnell, Kristjan Haav, Tõiv Haljasorg, Lauri Toom, Johan Bobacka, Ivo Leito

**Affiliations:** 1 Institute of Chemistry, University of Tartu, Ravila 14a, 50411 Tartu, Estonia, https://analytical.chem.ut.ee; 2 Johan Gadolin Process Chemistry Centre, Laboratory of Molecular Science and Engineering, Åbo Akademi University, Biskopsgatan 8, FI-20500 Turku/Åbo, Finland

**Keywords:** anion sensors, carboxylates, ionophores, macrocycles, sensor prototype

## Abstract

Carboxylate sensing solid-contact ion-selective electrodes (ISEs) were created to provide a proof-of-concept ISE development process covering all aspects from in silico ionophore design to functional sensor characterization. The biscarbazolylurea moiety was used to synthesize methylene-bridged macrocycles of different ring size aiming to fine tune selectivity towards different carboxylates. Cyclization was achieved with two separate strategies, using either amide synthesis to access up to –[CH_2_]_10_– macrocycles or acyl halides to access up to –[CH_2_]_14_– macrocycles. Seventy-five receptor–anion complexes were modelled and studied with COSMO-RS, in addition to all free host molecules. In order to predict initial selectivity towards carboxylates, ^1^H NMR relative titrations were used to quantify binding in DMSO-*d*_6_/H_2_O solvent systems of two proportions – 99.5%:0.5% m/m and 90.0%:10.0% m/m, suggesting initial selectivity towards acetate. Three ionophores were selected for successful sensor prototype development and characterization. The constructed ion-selective electrodes showed higher selectivity towards benzoate than acetate, i.e., the selectivity patterns of the final sensors deviated from that predicted by the classic titration experiments. While the binding constants obtained by NMR titration in DMSO-*d*_6_/H_2_O solvent systems provided important guidance for sensor development, the results obtained in this work emphasize the importance of evaluating the binding behavior of receptors in real sensor membranes.

## Introduction

In 2013, Otto S. Wolfbeis asked the supramolecular community a question of justified critique: why do we have so few true sensors? [1]. Indeed, in the recent decades the progress towards new actual chemical sensor systems has been slow. We have seen numerous potentially very promising receptor candidates from synthetic chemistry – essential as the central recognition elements of chemical sensors. However, the research typically ended with demonstration of the ability of analyte binding. Rarely have the receptors found their way into functioning chemical sensor prototypes. The reason for this is that the step from a well-binding receptor to a sensor prototype poses significant challenges.

The development of a chemical sensor consists of a number of steps that require knowledge and skills from different disciplines. Although not always necessary [2], specific selectivity towards a particular analyte is accessible with the use of a specialized ionophore.

We directed our efforts towards sensing carboxylates. The detection of these analytes is important in a number of areas and there is an obvious lack of easy-to-use sensing methods. Carboxylates are challenging analytes for two reasons. Firstly, binding is mainly achieved through either ion–ion interactions or hydrogen bonding. The primary site of interaction is the X-COO^−^ group. The geometry of the carboxylate group (and to a large extent also its charge distribution) is the same for all carboxylate anions. Therefore, high affinity towards carboxylates is easily achievable, but selectivity towards a specific carboxylate anion will prove challenging.

Secondly, the X-COO^−^ group itself may become protonated under specific conditions. In this scenario, selectivity would be lost immediately. This puts restraints on the molecular design of the receptor molecule. The host molecule cannot include any functional groups that would allow the analyte to become protonated.

In order to incorporate both functional parts of a carboxylate, a macrocyclic receptor architecture is desirable. By using a cyclic structure, it is possible to accommodate a solvophobic environment alongside polar functional groups. The benefits of this protein-inspired architecture have recently been praised in the use of naphthotubes [3]. Association between the receptor and the analyte may change considerably when the receptor is attached to an electrode surface or embedded in a sensor membrane. Water, as the most common environment for real-life sensing, also influences binding [4]. Within an ion-selective electrode (ISE), lipophilicity is mostly contributed by the low polarity membrane, which is commonly based on plasticized PVC [5].

**Figure 1:**
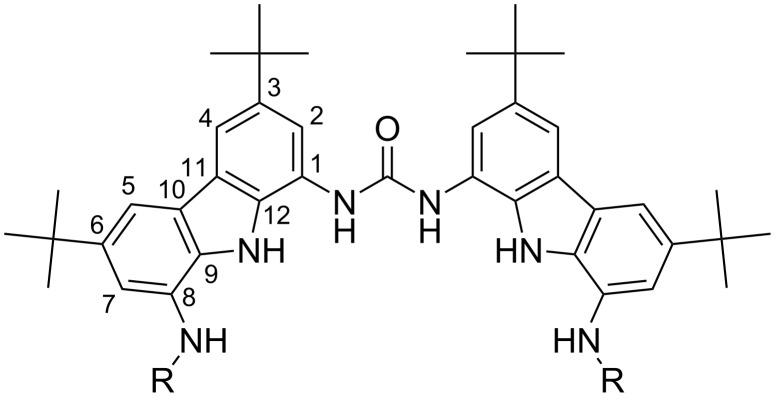
The biscarbazolylurea moiety.

One possible receptor family for carboxylates is based on the biscarbazolylurea moiety (Figure 1). This moiety fulfils several key design requirements. At least four hydrogen bonding sites (N–H groups) are available and positioned in a favourable geometry for carboxylates. Additional hydrogen-bond-donor (HBD) groups can be added with substituents. The solubility of such receptors can be tuned using functional groups in the 3,6-positions of the carbazole. High lipophilicity is achievable by using alkylation and leads to the possibility of using the receptor dissolved in the lipophilic polymeric sensor membrane, without the need for chemical linking. Cyclization of the receptor is possible via the positions 8 of the carbazole, using, e.g., amide bonds.

Anion receptors containing carbazole and amide functionalities were investigated in numerous works [6–9]. In some cases, these functionalities were incorporated into macrocyclic systems, thereby offering valuable insight for design criteria. For example, a carbazole-urea macrocycle was reported previously [10], however, the binding of anions occurred outside the receptor due to modest dimensions of the macrocyclic cavity. Using click-chemistry, a carbazole-triazole macrocycle, “tricarb”, was prepared that showed the ability to form non-covalent superstructures [11].

The rings should be of remarkable size for accommodating even small anions, such as acetate. In many cases the receptor is formally a macrocycle, but the anion is bound on its “surface”, not inside the ring [10,12]. A well-known example is the calix[4]pyrrole family, which emerged as an attractive neutral host for anions [13]. It is formally a 16-membered ring, but for the above reasons no anion, not even F^−^ fitted inside the ring – all bound anions reside on the surface of the ring [14]. Often in such cases, two receptors stack the guest molecule in a “sandwich” like manner, e.g., the “cyanostar” macrocycle [15–16]. Although not a macrocycle, a successful binding motif that is noteworthy for its ability to bind carboxylates in polar environments, is the guanidiniocarbonylpyrrole (GCP) moiety designed by Carsten Schmuck and Michael Schwegmann [17].

The transition from complexation studies to the next phase of constructing a prototype sensor is where progress often stopped and one answer to Wolfbeis’ question can be found. If we ever wish to see the developed champion molecule in commercial use, several conditions must be met. The yield of the synthetic pathway can be low and require optimization. The chemical characteristics of the champion molecule might exclude its successful commercialization – solubility issues, chemical instability, secondary effects during binding (e.g. superstructures), too high affinity constants, insufficient selectivity towards the analyte, etc. If a champion with sufficient characteristics is found, then it can be incorporated into a prototype sensor.

Different strategies are available for electrochemical sensor development [18–19]. While several cation-sensing electrodes have been successfully commercialized, only a limited number of anion-sensing electrodes are commercially available. This is largely due to the challenges associated with the selectivity of ionophores towards anions [20–21]. In this work, potentiometric solid-contact ion-selective electrodes (SC-ISEs) were chosen as the sensor type for several reasons. Potentiometric ISEs possess several sought-after characteristics for a sensor: easy to produce, low manufacturing and operating costs, easy to use, portable, and sufficiently quick response [5,22–23]. SC-ISEs have been used for decades in research and can be used to make less fragile and more easily miniaturized ISEs than the conventional ISEs [24]. Other electrochemical readout principles can be used with these sensors, but potentiometry has well established theoretical foundations and protocols for the characterization of ISEs. Equilibration times are typically also short in potentiometry. This is appropriate for the current study where the life span of the sensors may turn out to be shorter than expected.

The potentiometric response of an ISE to the activity of the primary ion, *i*, can be described by the Nikolsky–Eisenman equation (Equation 1), which expands upon the Nernst equation by accounting for the interference caused by the activities of interfering ions, *j*, with a potentiometric selectivity coefficient, 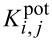.

[1] $$E=E^{0}+\frac{RT}{z_{i}F}\ln\left(a_{i}+\sum_{j}K_{i,j}^{\text{pot}}a_{j}^{z_{i}/z_{j}}\right)$$

The selectivity of SC-ISEs is typically achieved by coating the ion-to-electron transducer with an ion-selective membrane (ISM) containing the ion receptor (ionophore). The ISM matrix can be made of several different types of materials such as glass, crystal, or polymer. pH electrodes with glass membranes are the most used ISEs and produce highly selective responses over a wide range of concentrations. Polymeric membranes have gained popularity due to their robustness, the ability to fine-tune their properties and the ability to target a larger variety of ions compared to e.g. glass. Plasticizers may be required to improve transmembrane diffusion rates unless the polymer is self-plasticizing. Plasticized poly(vinyl chloride) (PVC), which was used in this study, is an example of a commonly used membrane matrix.

Permselectivity is conferred to the polymeric membrane by the addition of an ion exchanger, which is a lipophilic ion, and an ionophore. The ion exchanger provides permselectivity for anions or cations depending on whether the ion exchanger salt contains a lipophilic cation or anion, respectively. Additional selectivity is provided by the ionophore, which is typically confined to the membrane in one of two ways. One possibility is to immobilize a receptor in a polymeric membrane with the use of covalent bonding. Another strategy is to dissolve the receptor molecule in the polymeric membrane, which was the approach that was used in this study.

**Figure 2:**
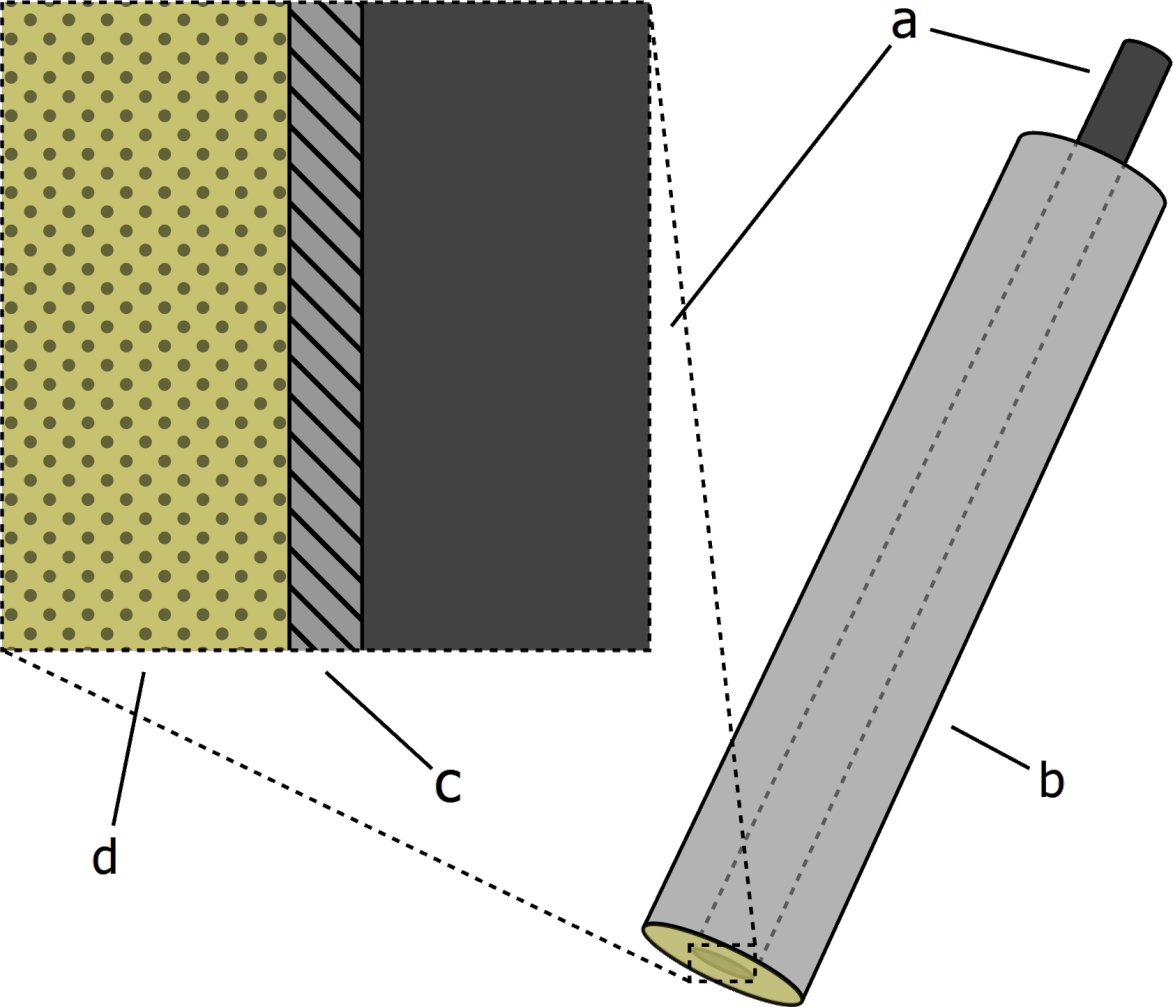
The structure of the solid-contact ion-selective electrode (sensor): a) glassy carbon as the electronic conductor; b) insulating shell made from PVC; c) PEDOT-Cl as the solid contact (ion-to-electron transducer); d) an ion-selective membrane with a plasticized PVC matrix. The inset shows a cross-section of the center of the sensor tip.

In this case, the receptor molecule must be highly lipophilic. It is usually easier to tune the solubility of the receptor molecule than it is to add the functional groups necessary for covalent immobilization [25]. Therefore, these types of sensors are easier to prepare but their life span is shorter due to the possibility of the receptor molecule leaking from the polymeric membrane into the analyte solution. The ratio of ionophore to ion exchanger is an important parameter and typically an excess of ionophore (e.g., 2:1) is used to prevent coextraction of ions with the opposite sign from the sample along with the primary ion. The construction of the SC-ISEs used in this work is shown schematically in Figure 2. In these electrodes, poly(3,4-ethylenedioxythiophene) doped with chloride (PEDOT-Cl) was used as the solid contact (ion-to-electron transducer).

## Results and Discussion

### Design

**Figure 3:**
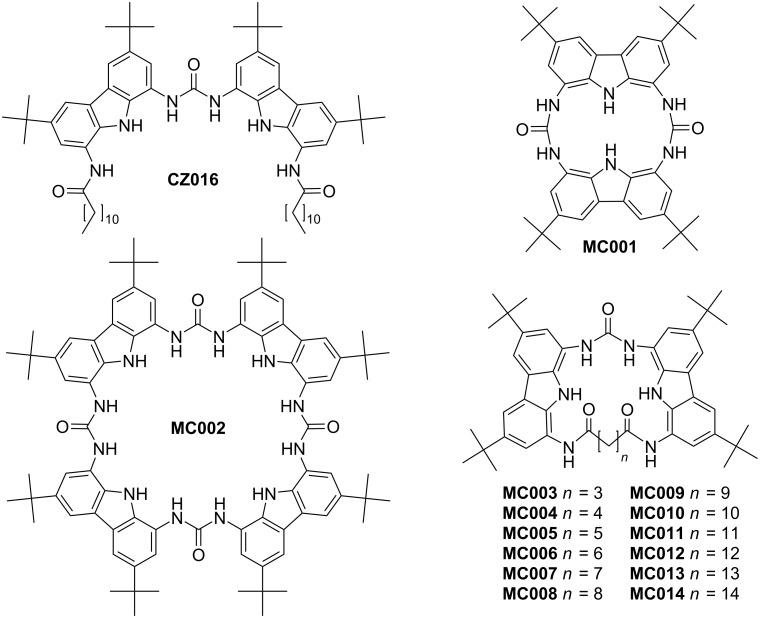
Studied receptor molecules.

We aimed to investigate the effects of ring size on the binding of carboxylates and the biscarbazolylurea moiety was selected. The studied receptors are shown in Figure 3. In order to achieve macrocyclization, we decided to use linear aliphatic dicarboxylic acid residues (from glutaric to thapsic acid) with different chain lengths, allowing manipulation of the ring size. Similar strategies for closing macrocycles have previously been published [26–28].

To predict possible binding properties, all methylene-bridged macrocycles and their binding to several carboxylates were modelled computationally using COSMO-RS. Several observations were made from the computational structures.

**Figure 4:**
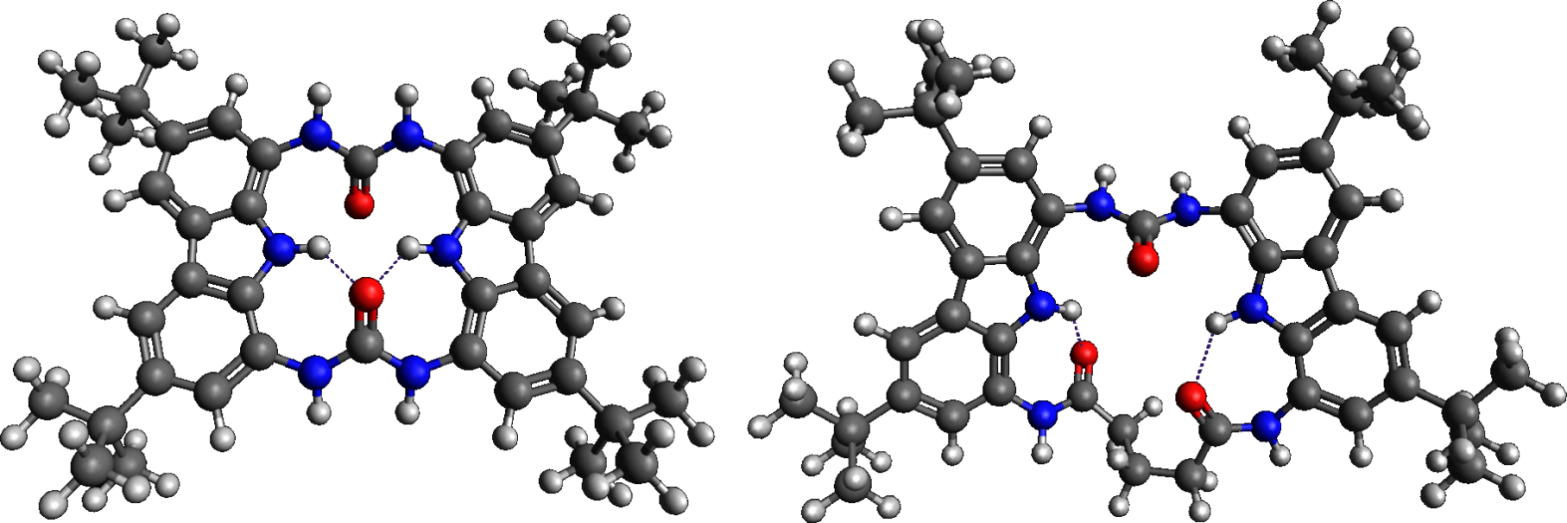
**MC001** and **MC003** lowest energy conformers (COSMO-RS) showing intramolecular bonds. Color coding: white – hydrogen; green – carbon; blue – nitrogen; red – oxygen.

Intramolecular hydrogen bonds between the urea carbonyl and the carbazole NH protons were present in macrocycles **MC001** and **MC003**, as indicated in Figure 4. The rings of these macrocycles, alongside with **MC004** and **MC005**, were too small to bind any of the studied anions. In the case of **MC001**, two anions could interact with it in such a way that binding occurs outside the macrocycle. For **MC003**–**MC005**, the ring could accommodate the carboxylate group COO^−^, however, the residue –X would be forced out of the macrocycle plane. It was expected that these macrocycles will have lower affinity constants.

**MC002** was of sufficient size to accommodate smaller carboxylate ions. However, in the most stable computed structures the hydrogen-bond donor NH fragments directed towards the outside of the ring. In addition, the increased ring size could accommodate solvent molecules. Thus, an additional hindrance to anion binding could occur due to the need to displace solvent molecules.

**Figure 5:**
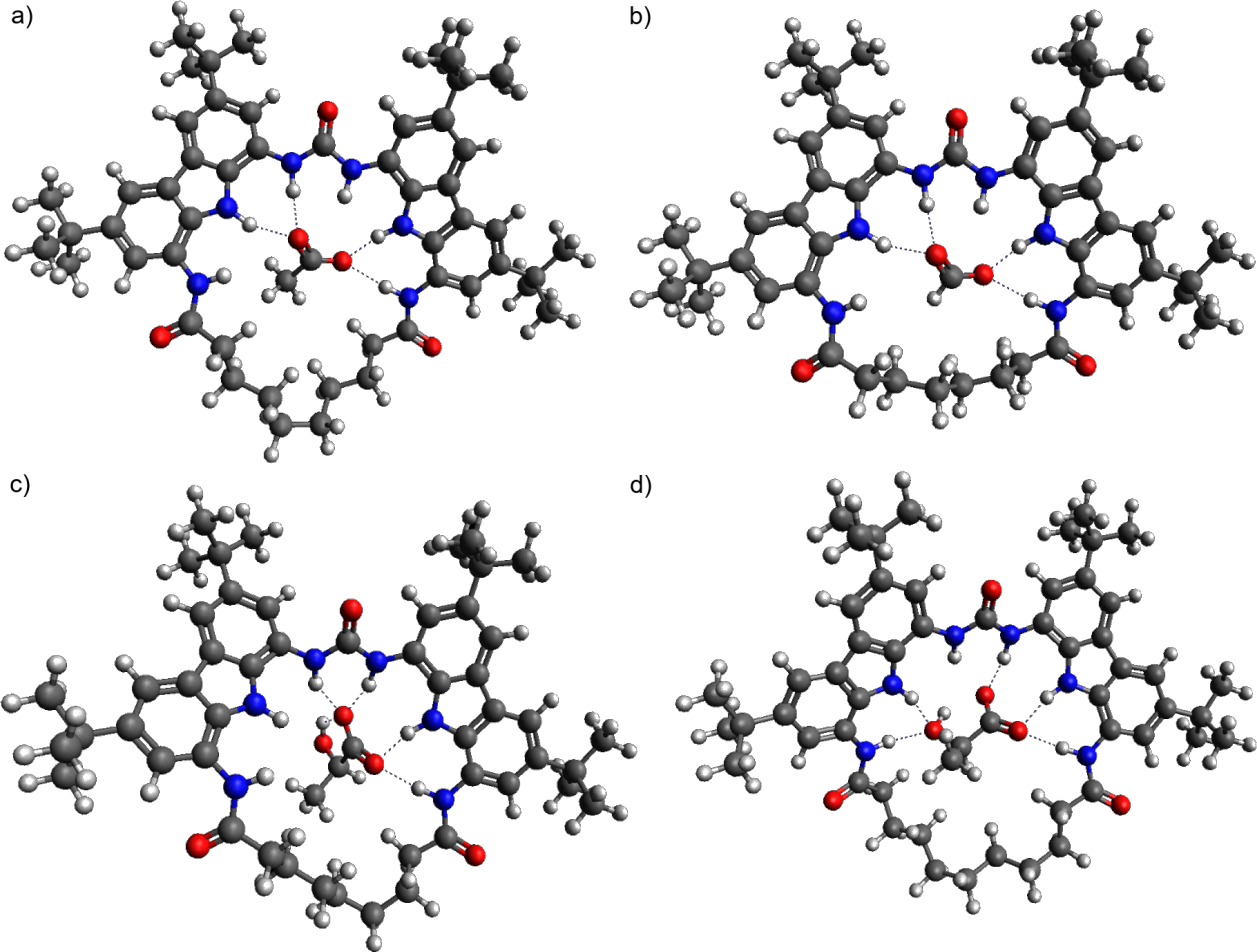
a) Complex of **MC008** with acetate; b) complex of **MC006** with formate; c) complex of **MC007** with lactate showing an intramolecular hydrogen bond; d) complex of **MC009** with lactate showing an additional hydrogen bond with an amide NH of the macrocycle.

According to the computations, the rings formed by macrocycles starting from **MC006** had sufficient size to fit anions, starting with formate. **MC008** was able to fit acetate and lactate. Thus, an additional binding site was available for lactate. In the complexes of lactate with **MC006**–**MC009**, a hydrogen bond was present between the oxygen of the hydroxy group of lactate and the NH group of the macrocyclic amide. See Figure 5 for visualization. An equilibrium likely existed between two conformers where one complex had an additional hydrogen bond and the other included intramolecularly bonded lactate. For the lactate complexes with the larger receptors, whether one dominant conformer or a mixture of two conformers was preferred seemed to be dependent on the –CH_2_– linker length.

Benzoate and pivalate were too large to fit the rings of even the biggest macrocycles. However, the out of plane residue –X should be able to, at least to some extent, benefit from the solvophobic effects of the –CH_2_– linker if binding occurred in water (and possibly also in DMSO).

### Synthesis

**Scheme 1:**
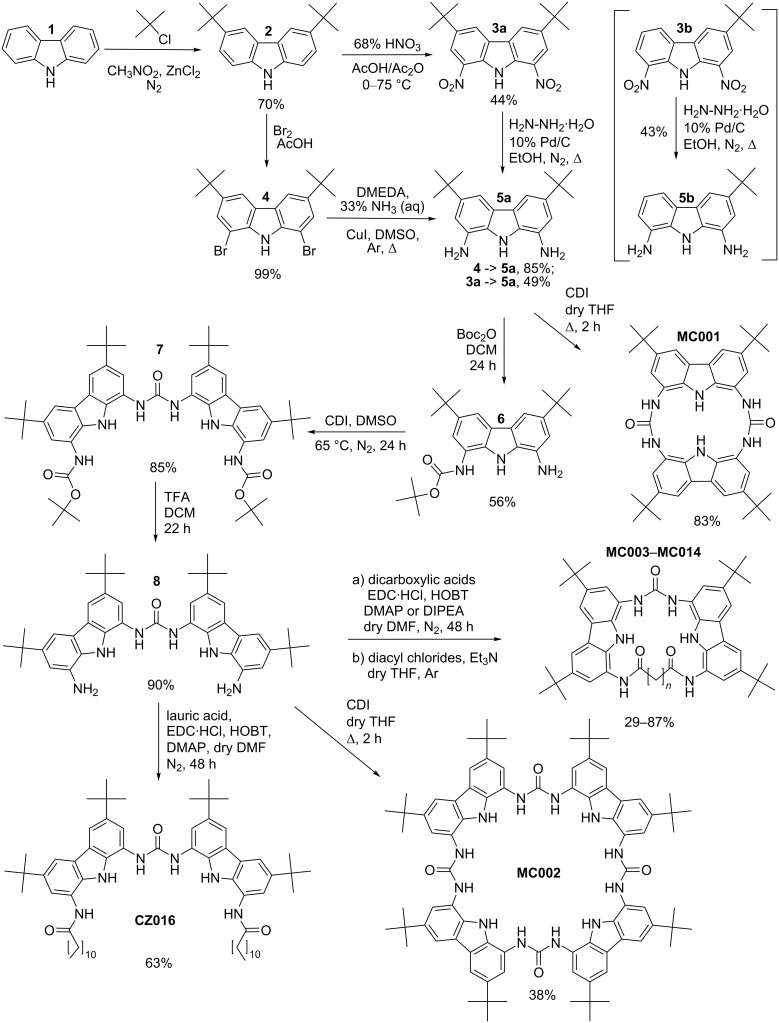
The synthetic pathway to receptors **CZ016** and **MC001**–**MC014**. The reaction yield for **2**–**3a**/**3b** is given as a sum for two compounds, as they were inseparable by purification.

The general synthesis route is presented in Scheme 1. It was to some extent similar to the one that has previously been published by Sanchez et al. and Liu et al. [6,29] (see below). The synthetic route relied on the initial alkylation of the 3 and 6 positions of carbazole for tuning the solubility profile and for directing reactivity towards the 1 and 8 positions. The obtained molecule **2** was then nitrated and hydrogenated to obtain the amine **5a**. The amine group was protected with a Boc-group at one substituent and coupled using CDI, affording the protected biscarbazolyl derivative **7**. TFA was used for deprotecting **7**, which led to the corresponding free amine derivative **8** that could directly be used for cyclization.

We were unable to successfully follow all steps in the synthesis scheme by Sanchez et al. [6]. The problematic step was the nitration reaction. Due to the highly acidic environment partial loss of one of the *tert*-butyl groups in **2** took place. This considerably reduced the yield and resulted in a mixture of products **3a** and **3b** that was challenging to separate due to similar properties. Careful optimization of the nitration process did not lead to acceptable results. Therefore, we decided to modify the reaction scheme. After alkylation, compound **2** was instead brominated, followed by amination of **4** leading to the desired diamino product **5a**. Amine protection and subsequent coupling procedures, leading to **8**, were the same as described in reference [6].

It is worth noting that the described cleavage problem during nitration was specific to *tert*-butyl groups. We also studied the possibility of hexyl substituents instead of *tert*-butyl groups [25]. In this case, the nitration process yielded only the desired product. However, since this possibility was investigated after the production of the first macrocycles bearing *tert*-butyl substituents, we decided to not switch substituents.

The formation of macrocycles from **8** was achieved following two different synthetic strategies. For receptors **MC003**–**MC010**, amide bond formation was carried out using dicarboxylic acids alongside EDC·HCl, HOBT and DMAP/DIPEA. This method was accompanied with a systematic decrease of the yield with the lengthening of the –CH_2_– methylene bridge. To counteract the decrease of yield a second approach was adapted for the larger receptors **MC011**–**MC014**. For this the corresponding dicarboxylic acids were first converted to the diacyl chlorides, which were then used for cyclization. This mitigated the yield drop and provided access to the higher macrocycles **MC011**–**MC014**.

In addition, three other receptor molecules were prepared. **MC001** and **MC002** were prepared using CDI coupling of the corresponding amino derivatives according to Scheme 1. The synthesis of **CZ016** is also described in Scheme 1. Details of all synthetic procedures can be found in Supporting Information File 1.

### Binding measurements

The binding properties of the macrocycles are expressed by the logarithms of the binding (association) constants log*K*_ass_, which were measured using our previously published relative NMR method under fast exchange conditions [30]. Analogous to our earlier works with similar receptors, a binding stoichiometry of 1:1 was ensured by using low concentrations of receptor and anion – mimicking the real situation when using the receptors in sensors. The stoichiometry was confirmed by data treatment – the experimental results agreed with the 1:1 binding model. Please refer to Supporting Information File 1 for additional details on the relative binding measurements. The binding affinities for **MC002** with lactate and pivalate were obtained by UV–vis measurements as absolute values [31]. Five carboxylates were studied in the form of their tetrabutylammonium salts: pivalate, acetate, benzoate, lactate, and formate. The anions were titrated in DMSO-*d*_6_/H_2_O solvent systems of two proportions – 99.5%:0.5% m/m [30] and 90.0%:10.0% m/m [31].

**Figure 6:**
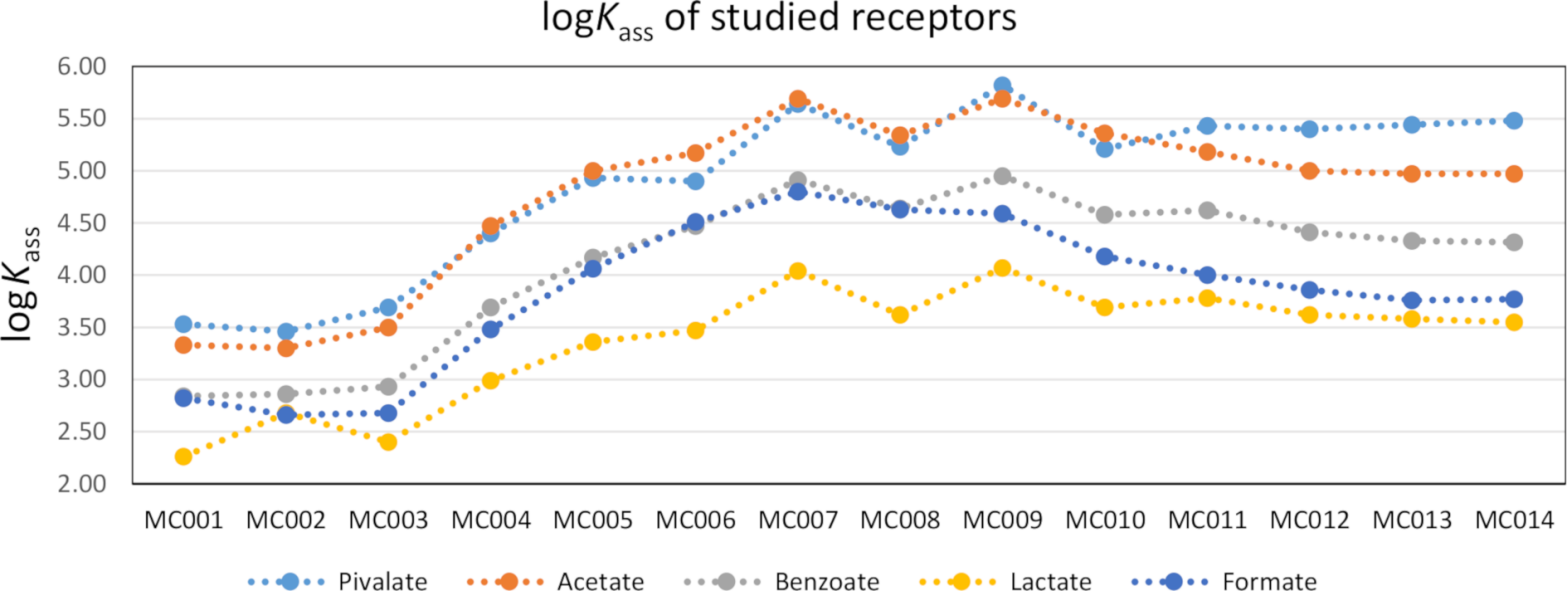
Binding affinities of the studied receptors towards different carboxylates in DMSO-*d*_6_/H_2_O (99.5%:0.5% m/m).

The 0.5% m/m water addition was necessary to make the results more stable by minimizing the effect of DMSO’s hygroscopicity and thereby stabilizing the water content in the solution. Since anions are solvated considerably more strongly in pure water than in DMSO, we also studied binding using a higher water content in DMSO. The 10.0% m/m mixture was expected to lower the binding affinities for all receptor–anion combinations. The results are presented in Table 1 and Figure 6.

**Table 1:** Binding affinities log*K*_ass_ of different carboxylates in 0.5% and 10% DMSO-*d*_6_/H_2_O solvent systems.^a^

anion receptor	pivalate		acetate		benzoate		lactate		formate	
	0.5%	10%	0.5%	10%	0.5%	10%	0.5%	10%	0.5%	10%

**CZ016**	5.16	^b^	4.82	^b^	4.20	^b^	3.29	^b^	3.81	^b^
**MC001**	3.53	^b^	3.33	^b^	2.84	^b^	2.26	^b^	2.82	^b^
**MC002**	3.46	^b^	3.30	^b^	2.86	^b^	2.68	^b^	2.66	^b^
**MC003**	3.69	^b^	3.50	^b^	2.93	^b^	2.40	^b^	2.68	^b^
**MC004**	4.40	2.06	4.47	1.97	3.69	1.63	2.99	1.75	3.48	1.69
**MC005**	4.93	3.63	5.00	3.78	4.17	3.80	3.36	4.03	4.06	3.58
**MC006**	4.90	^b^	5.17	^b^	4.47	^b^	3.47	^b^	4.51	^b^
**MC007**	5.64	4.12	5.69	3.86	4.91	3.70	4.04	3.07	4.80	3.26
**MC008**	5.23	3.80	5.34	3.75	4.64	3.67	3.62	3.55	4.63	3.55
**MC009**	5.82	4.60	5.69	3.74	4.95	3.73	4.07	2.73	4.59	2.84
**MC010**	5.21	^b^	5.36	^b^	4.58	^b^	3.69	^b^	4.18	^b^
**MC011**	5.43	^b^	5.18	^b^	4.62	^b^	3.78	^b^	4.00	^b^
**MC012**	5.40	^b^	5.00	^b^	4.41	^b^	3.62	^b^	3.86	^b^
**MC013**	5.44	^b^	4.97	^b^	4.33	^b^	3.58	^b^	3.76	^b^
**MC014**	5.48	^b^	4.97	^b^	4.32	^b^	3.55	^b^	3.77	^b^

^a^All anions were used as tetrabutylammonium salts at ambient temperatures. The standard uncertainties of the log*K*_ass_ values, accounting for consistency of measurements, uncertainty of anchoring and possible systematic effects (see reference [8] for details) were in the range of 0.04 and 0.09; ^b^solution not prepared due to low solubility of the receptor.

Initially, the trend suggested a steady increase of the binding affinity with increasing ring size. This, however, reached a plateau at around 7–8 carbon atoms in the chain and the maximum affinity value for any given anion did not increase thereafter, except for the case of pivalate (**MC009** had the highest log*K*_ass_). With chain lengths of more than 9 carbon atoms the log*K*_ass_ values started to decrease. The initial increase of the binding affinity suggested that just fitting into the ring was not a sufficient criterion for predicting binding affinity. This was because for the macrocycles **MC003**–**MC005**, the computational geometries suggested that the ring is too small to bind any of the studied anions. Additionally, for **MC003**, intramolecular hydrogen bonds could be present.

For most anions, the champion molecules were **MC007** and **MC009**. According to the computation results, neither benzoate nor pivalate were able to fit into the ring of these receptors because these anions were too large. Yet, **MC007** and **MC009** bound benzoate and pivalate better than larger macrocycles where the steric strain was expected to be lower.

As expected, the increase of water content in DMSO reduced binding affinities. The reduction was not uniform for the different anions. According to Table 1, it was related to the hydrophilicity of the ions – in the case of smaller and more hydrophilic anions the reduction was more significant. A comparison between all anions was possible with receptor **MC009**. The binding strength reduction with **MC009** was 1.95 with acetate, 1.75 with formate, 1.34 with lactate, and 1.22 with both pivalate and benzoate.

Compound **CZ016**, being an open-chain receptor, competed well with the other macrocyclic structures. This was partially due to the increased freedom of movement of the alkyl chains as opposed to the fixed macrocycles (please see the Supporting Information File 1 for computational geometries), allowing for more dynamic complexes to form. Such affinity was not entirely surprising as both, the open-chain receptor and the closed macrocycles, employed only aliphatic substituents in the 1 and 8 positions of the biscarbazolylurea moiety.

### Preparation and characterization of sensor prototypes

The sensor prototype preparations were done similarly to our previously published method for an acetate-selective solid-contact electrode [32]. Three macrocyclic receptors with different ring sizes were chosen as ionophores: **MC005**, **MC009**, and **MC012**. Ion-selective membranes (ISMs) were prepared from poly(vinyl chloride) plasticized with bis(2-ethylhexyl)sebacate (DOS), which was chosen since it was considered to be suitable for use in membranes with monovalent primary ions [5]. The compositions of the ISMs were 2 wt % ionophore, 50 mol % anion exchanger relative to the ionophore, 65 wt % DOS, and 32 wt % PVC. A control membrane was also prepared with no ionophore, 0.7 wt % anion exchanger, 66 wt % DOS, and 33 wt % PVC. The dry weight of the membrane cocktails was 17% m/m. The sensors (Figure 2) were prepared with electrode bodies made from glassy carbon (GC) rods (diameter = 3 mm) encased in PVC cylinders (outer diameter = 8 mm). The GC rods functioned as the electronic conductors and the PVC shells were used to both control the amount of exposed GC surface and to function as the substrate for the ISM to adhere to. Poly(3,4-ethylenedioxythiophene) doped with chloride (PEDOT-Cl) was electropolymerized onto the exposed GC as a solid contact and provided improved potential stability in comparison to a bare GC surface [33]. The membranes were applied to the electrode bodies by drop-casting the membrane cocktails (100 μL/electrode) and allowing the solvent to evaporate. Three sensors were prepared with each of the membranes in an identical way and were conditioned in 0.1 M sodium acetate solutions. The sensors were characterized using electrochemical impedance spectroscopy (EIS) and potentiometry. Please see Supporting Information File 1 for detailed preparation and characterization data of the sensor prototypes.

**Figure 7:**
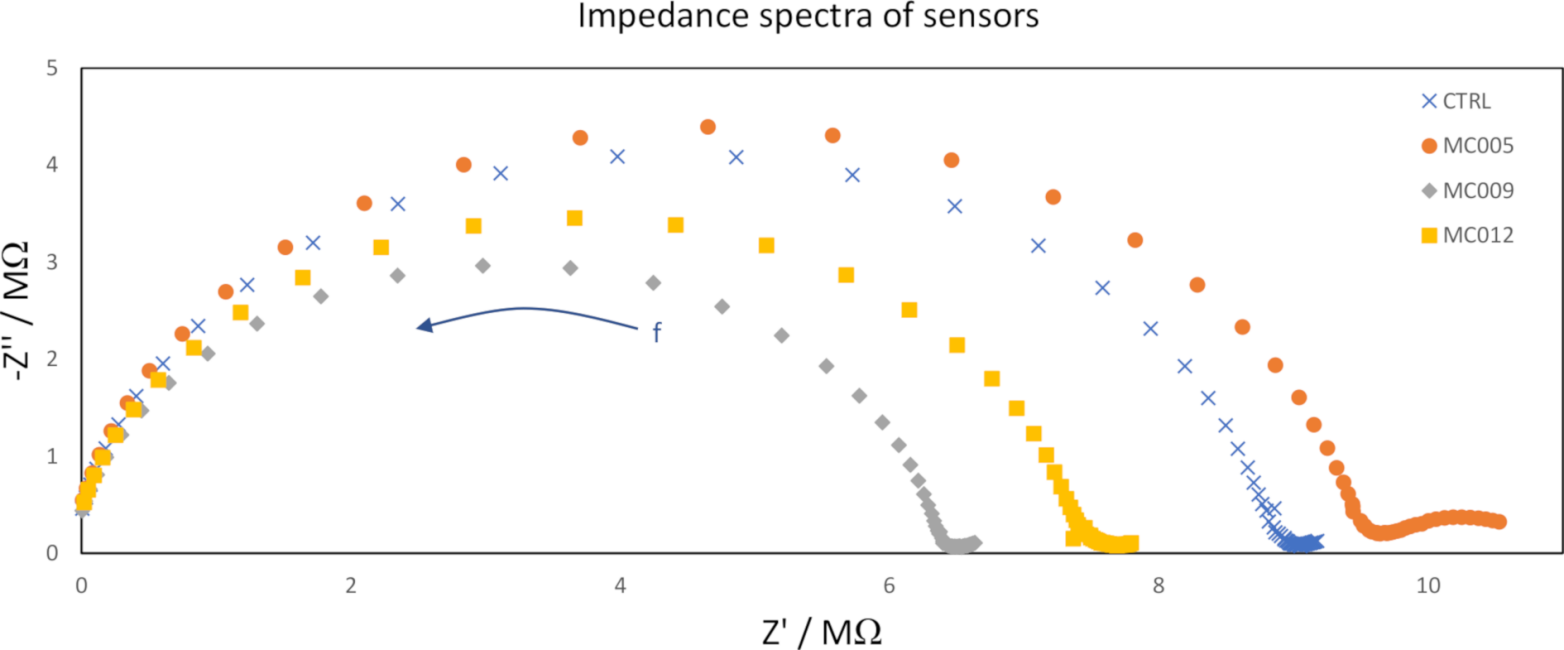
Impedance spectra of sensors with each of the membranes. The spectra were recorded in 0.1 M sodium acetate in the frequency range 100 mHz to 100 kHz at open-circuit potential.

EIS measurements were used primarily to ensure that the solid contact had a sufficiently large redox capacitance and to compare the influence of the ionophore upon the spectra, if any could be observed. Examples of the recorded impedance spectra are shown in Figure 7. The membrane bulk resistance and geometric capacitance were attributed to the high-frequency semicircle. The membrane bulk resistance and geometric capacitance were heavily influenced by the properties of the plasticizer, DOS (ε_r_ = 4.01 [34]), because it accounted for most of the mass in all of the membranes. The bulk resistances were in the MΩ range, which was typical for PVC membranes plasticized with DOS (for the given membrane geometry) [33]. There was some variation from sensor to sensor, which was attributed to variations in membrane thicknesses. The lack of any large semicircles or capacitive lines at lower frequencies showed that the solid contacts had large redox capacitances [33]. The low-frequency regions showed some differences between the different membranes and **MC005** stood out due to the presence of a partial semicircle that was absent in all other membranes. The low-frequency region is associated with transport phenomena and this semicircle was attributed to slow ion transfer kinetics at the ISM interfaces.

**Figure 8:**
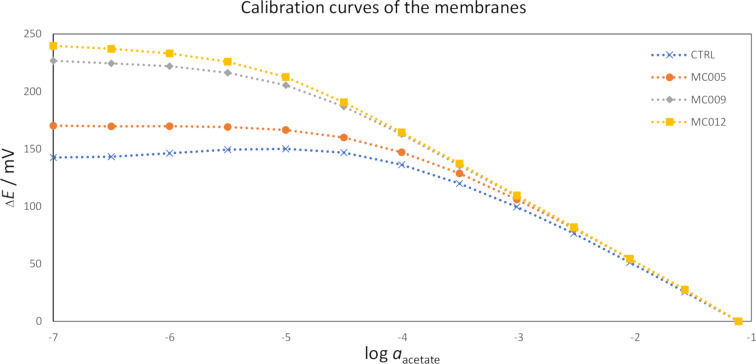
Calibration curves for each of the membranes. The calibrations were performed by diluting 0.1 M sodium acetate in half-decade steps until the potentials of the sensors levelled out. The potentials were shifted along the *y*-axis to start at Δ*E* = 0.00 mV at log a = −1.11.

Potentiometric calibrations in sodium acetate solutions were used to determine the linear ranges (*R*^2^ ≥ 0.999), slopes, and detection limits of the sensors (Table 2). Examples of the calibration curves are shown in Figure 8. All of the measured slopes were sub-Nernstian (i.e., less negative than −58.8 ± 0.4 mV/dec at temperatures of 23 ± 2 °C). The **MC012** and **MC009** sensors exhibited the best and second-best responses, respectively, according to all of the quantified characteristics. The responses of the **MC005** sensors were only slightly better than those of the control sensors. The poorer response characteristics and greater standard deviations of the **MC005** and control sensors could be attributed to their sensitivity to chloride, which will be discussed in more detail later.

**Table 2:** The response characteristics of each membrane determined from the potentiometric calibrations in sodium acetate. The upper linear limit was 10^−1.11^ M in all cases and activities greater than that were not measured. The uncertainties express standard deviations.

	CTRL	MC005	MC009	MC012

slope (mV/dec)	−52.14 ± 0.82	−54.59 ± 0.53	−55.77 ± 0.30	−56.56 ± 0.35
log *a*_LLL_^a^	−2.77 ± 0.39	−3.24 ± 0.36	−4.00 ± 0.00	−4.00 ± 0.00
log *a*_LOD_^b^	−3.74 ± 0.39	−4.16 ± 0.30	−4.94 ± 0.09	−5.12 ± 0.11

^a^Lower limit of linear range; ^b^limit of detection.

**Figure 9:**
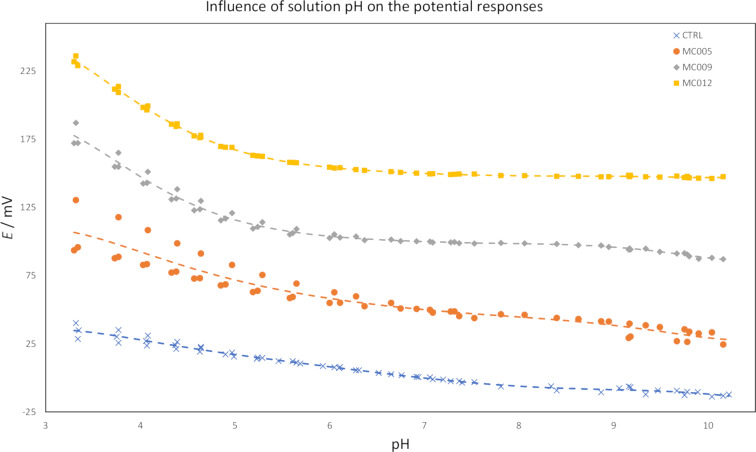
The influence of solution pH on the potential responses of the sensor prototypes (three sensors for each membrane composition). The measurements were performed in 0.01 M acetic acid. Potentials have been adjusted to overlap the responses of sensors with the same membrane.

The pH sensitivity of the sensors was assessed by measuring their potentiometric responses in acetic acid. The solutions were titrated from pH 3.3 to pH 10 with a mixture of sodium acetate of equal concentration to the initial acetic acid solution and concentrated sodium hydroxide. The activity of acetate was expected to increase between pH 2.8 and 6.8 due to the deprotonation of acetic acid (p*K*_a_ = 4.76) [34], which should result in a decrease in the measured potentials. The measured potentials were then expected to remain constant after pH 6.8 since the activity of acetate should no longer appreciably change with the increase of pH. Structural differences between the different macrocycles were not expected to cause differences in pH sensitivity. The results of these measurements are shown in Figure 9. The **MC012** sensors were the least affected by the change in solution pH and their responses adhered well to the expected response with minimal interference. The **MC009** sensors adhered quite well to the expected response up to pH 9 whereupon a clear decline in the potentials was present. A steady decline could be seen in the potentials of the control and **MC005** sensors throughout the entire pH range. This again may be explained by their sensitivity to chloride, but the **MC005** sensors exhibited a slightly stronger response than the controls did to the increase of acetate activity in the beginning of each measurement. The deviations observed in the responses of the **MC005** and **MC009** sensors below pH 6 could be attributed to interference from chloride. Chloride was at least partially responsible for the deviations observed above pH 8 and pH 9, but interference from hydroxide and/or carbonate cannot be ruled out.

**Figure 10:**
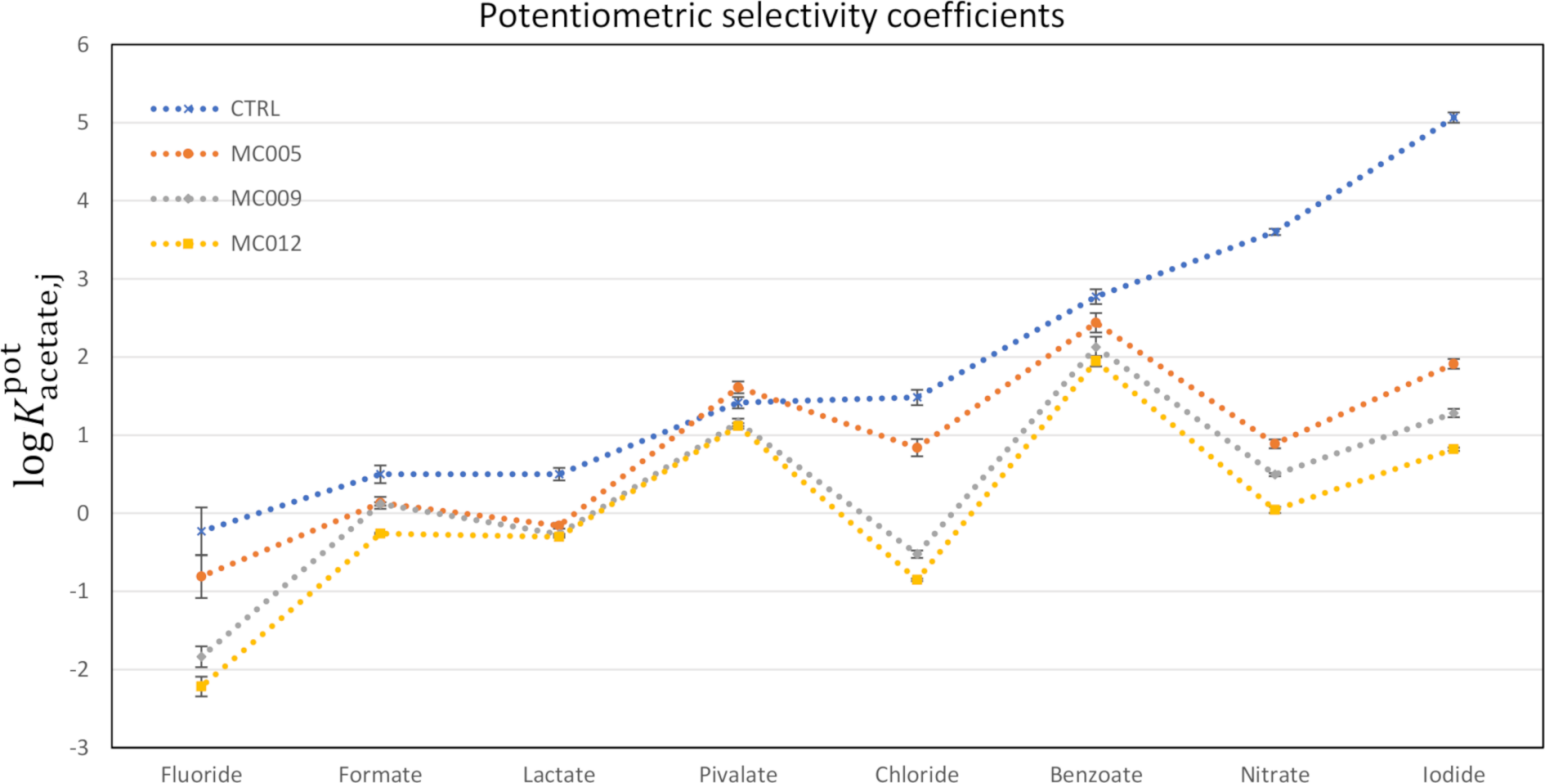
Potentiometric selectivity coefficients of interfering anions (relative to acetate) determined using the separate solution method at ambient temperatures. All anions were used as sodium salts. The uncertainties (error bars) express standard deviations.

The selectivity of each membrane was evaluated with the separate solution method. The results for each interfering ion are shown in Table 3 and Figure 10. The carboxylates that were used in the determination of binding affinities in DMSO were included to see how the affinities affect the selectivity of the ISMs towards acetate over other carboxylates. A few common non-carboxylate anions were also included in the selectivity measurements since the sensors should be able to discriminate interfering anions to be of practical use. Chloride was included primarily due to its presence in the filling solutions of the reference electrode. As a result, it inevitably leaches into all of the samples. The chosen non-carboxylate anions also represented different parts of the Hofmeister series. Chloride and nitrate are also common in real-world samples that such sensors might be used to analyse. Fluoride and iodide represented halides at opposite ends of the Hofmeister series.

**Table 3:** Potentiometric selectivity coefficients of interfering anions (relative to acetate) determined using the separate solution method at ambient temperatures. All anions were used as sodium salts. The uncertainties express standard deviations.

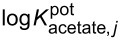				

ion *j*	control	**MC005**	**MC009**	**MC012**

iodide	5.06 ± 0.07	1.91 ± 0.06	1.28 ± 0.05	0.82 ± 0.02
nitrate	3.60 ± 0.04	0.89 ± 0.06	0.49 ± 0.02	0.04 ± 0.04
benzoate	2.77 ± 0.09	2.44 ± 0.12	2.13 ± 0.13	1.95 ± 0.07
chloride	1.48 ± 0.10	0.84 ± 0.11	−0.52 ± 0.05	−0.85 ± 0.02
pivalate	1.42 ± 0.07	1.61 ± 0.08	1.16 ± 0.05	1.12 ± 0.03
lactate	0.50 ± 0.08	−0.16 ± 0.04	−0.27 ± 0.00	−0.30 ± 0.01
formate	0.50 ± 0.11	0.13 ± 0.08	0.12 ± 0.02	−0.26 ± 0.01
fluoride	−0.23 ± 0.31	−0.81 ± 0.28	−1.84 ± 0.13	−2.22 ± 0.13

The selectivity pattern for the control membrane followed the Hofmeister series, as expected, with more lipophilic anions causing more interference. The addition of any of the ionophores improved selectivity in all but one case – the selectivity toward acetate with respect to pivalate was lower in the case of the **MC005** membrane than it was for the control membrane. The interference caused by non-carboxylate anions decreased by up to four orders of magnitude by the addition of an ionophore. Other carboxylates were discriminated to a lesser extent. The selectivity towards chloride was important in understanding the responses observed during the calibration and pH-sensitivity measurements. Both the control and **MC005** sensors were more selective for chloride than for acetate. When these sensors were combined with the reference electrode, which had a 1 M potassium chloride outer filling solution, the chloride interfered and caused drifting potentiometric responses – chloride gradually leached into the samples from the reference electrode over the course of the measurements.

Conversely, the lower sensitivity of the **MC009** and **MC012** sensors to chloride explained their superior response characteristics. The outer junction of the reference electrode was an opening with an adjustable plastic plug, which can be used to empty the outer filling solution compartment, and the greater standard deviations in the results could be explained by slight differences in the flow of chloride between measurements. These variations in flow rate were likely due to variations in the position of the plug as a result of handling the reference electrode while setting up the measurements. This flow of chloride resulted in narrower linear ranges, lower slopes, and poorer detection limits due to the mixed responses to acetate and chloride. The degree of deviation from the expected response in the pH sensitivity measurements decreased with the decreasing sensitivity to chloride of the different ISMs. This also supported the interpretation that chloride was at least partially responsible for the deviations. Finally, the greater standard deviations in the selectivity coefficients for fluoride were due to an abnormally large flow rate of chloride from the reference electrode during a single measurement. This affected the responses for sensors that were used.

The ISMs showed modest selectivities. However, incorporating any of the three receptors in the membrane as an ionophore clearly disrupted the Hofmeister selectivity pattern that could be seen in the control membrane where the lipophilicity of the interfering ion was the dominant factor in determining the selectivity. All of the ionophores had a greater affinity for pivalate than for benzoate based on the measurements performed in DMSO, but their corresponding ISMs were more selective for benzoate (log *P*_oct/wat_ = 1.88) [34] than for pivalate (log *P*_oct/wat_ = 1.48) [35]. However, the influence of the ionophores’ affinities for carboxylates were clearly visible from the suppressed selectivity towards anions without carboxylate groups (fluoride, chloride, nitrate, iodide), compared to the control membrane. Optimization of the control membrane's composition (e.g., reducing the amount of anion exchanger or using another plasticizer) could improve its selectivity to some extent, but a disruption of the selectivity pattern based on the lipophilicity of ions in favor of a more hydrophilic primary ion would not be expected. However, ionophores can do just that as shown in Figure 10 by the reduced selectivity towards nitrate and iodide [19].

The sensors with the ionophore **MC012** had the best performance in this study and those sensors also compared quite favourably to some of the previously published ISEs for acetate by exhibiting a wider linear range, slope closer to theory, and a lower detection limit [36]. There was no overall champion in terms of selectivity when comparing the **MC012** sensors to the sensors from these earlier publications, as they outperform one another at discriminating different anions. Further optimization of the membranes could be done by trying different compositions (e.g., amount of ionophore, polymer:plasticizer ratio) and plasticizers. While the sensors in this study were considered to be acetate-selective for the purposes of characterization, the sensors turned out to show the highest selectivity to benzoate among the anions tested. These sensors could thus also be used as benzoate-selective sensors in applications where benzoate would be the main analyte of interest.

## Conclusion

To construct a functional chemical sensor, extensive interdisciplinary effort is required. A variety of problems must be addressed in several development stages, all of which were attended to in this work. The conceptual design of macrocyclic anion receptors led to in silico complexation studies of biscarbazolylurea-type ionophores. Eleven novel methylene-bridged macrocyclic receptor molecules were synthesized and characterized alongside a similar open-chain receptor and two additional macrocycles. Experimental complexation studies with different carboxylates were carried out in two different solvent systems to select three champions for constructing anion sensor prototypes. Binding studies in solution suggested selectivity towards acetate, however, this prediction did not hold true once the ionophores were incorporated into sensors. Overall, the constructed sensor prototypes showed modest selectivity towards acetate, which increased as the ionophore's cavity size increased. The sensors showed higher selectivity towards the more lipophilic carboxylates pivalate and benzoate. Incorporating any of the three chosen ionophores was shown to reduce interference from more lipophilic non-carboxylate interferents (such as iodide or nitrate) when compared to a control sensor.

## Supporting Information

File 1 Experimental part.
