# Supplementary material for: Design, synthesis and application of carbazole macrocycles in anion sensors
Source: Beilstein J Org Chem. 2020 Aug 4;16:1901–14. doi: 10.3762/bjoc.16.157 (PMC7418101; doi:10.3762/bjoc.16.157)
Supplement: File 1 — Experimental part. [file Beilstein_J_Org_Chem-16-1901-s001.pdf]

BEILSTEIN JOURNAL OF ORGANIC CHEMISTRY

Supporting Information

for

Design, synthesis and application of carbazole macrocycles in anion sensors

Alo Rüütel, Ville Yrjänä, Sandip A. Kadam, Indrek Saar, Mihkel Ilisson, Astrid Darnell, Kristjan Haav, Tõiv Haljasorg, Lauri Toom, Johan Bobacka and Ivo Leito

Beilstein J. Org. Chem. **2020,** *16,* 1901–1914. doi:10.3762/bjoc.16.157

Experimental part

License and Terms: This is a supporting information file under the terms of the Creative Commons Attribution License (http://creativecommons.org/licenses/by/4.0). Please note that the reuse, redistribution and reproduction in particular requires that the authors and source are credited.
The license is subject to the *Beilstein Journal of Organic Chemistry* terms and conditions: (https://www.beilstein-journals.org/bjoc)

Table of contents

General methods	S1
Synthesis and characterisation of compounds	S9
References	S23
Analytical spectra and pictures of Cosmo-RS conformers	
^{1}H NMR spectrum (700.1 MHz) of compound **CZ016**	S24
^{13}C NMR spectrum (700.1 MHz) of compound **CZ016**	S25
^{1}H-^{13}C HSQC spectrum (700.1 MHz) of compound **CZ016**	S26
^{1}H-^{13}C HMBC spectrum (700.1 MHz) of compound **CZ016**	S27
HRMS spectrum of compound **CZ016**	S28
^{1}H NMR spectrum (700.1 MHz) of compound **MC001**	S29
^{13}C NMR spectrum (700.1 MHz) of compound **MC001**	S30
^{1}H-^{13}C HSQC spectrum (700.1 MHz) of compound **MC001**	S31
^{1}H-^{13}C HMBC spectrum (700.1 MHz) of compound **MC001**	S32
HRMS spectrum of compound **MC001**	S33
^{1}H NMR spectrum (700.1 MHz) of compound **MC002**	S34
^{13}C NMR spectrum (700.1 MHz) of compound **MC002**	S35
^{1}H-^{13}C HSQC spectrum (700.1 MHz) of compound **MC002**	S36
^{1}H-^{13}C HMBC spectrum (700.1 MHz) of compound **MC002**	S37
HRMS spectrum of compound **MC002**	S38
^{1}H NMR spectrum (700.1 MHz) of compound **MC003**	S39
^{13}C NMR spectrum (700.1 MHz) of compound **MC003**	S40
^{1}H-^{13}C HSQC spectrum (700.1 MHz) of compound **MC003**	S41
^{1}H-^{13}C HMBC spectrum (700.1 MHz) of compound **MC003**	S42
HRMS spectrum of compound **MC003**	S43
^{1}H NMR spectrum (700.1 MHz) of compound **MC004**	S44
^{13}C NMR spectrum (700.1 MHz) of compound **MC004**	S45
^{1}H-^{13}C HSQC spectrum (700.1 MHz) of compound **MC004**	S46
^{1}H-^{13}C HMBC spectrum (700.1 MHz) of compound **MC004**	S47
HRMS spectrum of compound **MC004**	S48
^{1}H NMR spectrum (700.1 MHz) of compound **MC005**	S49
^{13}C NMR spectrum (700.1 MHz) of compound **MC005**	S50
^{1}H-^{13}C HSQC spectrum (700.1 MHz) of compound **MC005**	S51
^{1}H-^{13}C HMBC spectrum (700.1 MHz) of compound **MC005**	S52
HRMS spectrum of compound **MC005**	S53
^{1}H NMR spectrum (700.1 MHz) of compound **MC006**	S54
^{13}C NMR spectrum (700.1 MHz) of compound **MC006**	S55
^{1}H-^{13}C HSQC spectrum (700.1 MHz) of compound **MC006**	S56
^{1}H-^{13}C HMBC spectrum (700.1 MHz) of compound **MC006**	S57
HRMS spectrum of compound **MC006**	S58
^{1}H NMR spectrum (700.1 MHz) of compound **MC007**	S59
^{13}C NMR spectrum (700.1 MHz) of compound **MC007**	S60
^{1}H-^{13}C HSQC spectrum (700.1 MHz) of compound **MC007**	S61
^{1}H-^{13}C HMBC spectrum (700.1 MHz) of compound **MC007**	S62
HRMS spectrum of compound **MC007**	S63

^{1}H NMR spectrum (700.1 MHz) of compound **MC008** S64
^{13}C NMR spectrum (700.1 MHz) of compound **MC008** S65
^{1}H-^{13}C HSQC spectrum (700.1 MHz) of compound **MC008** S66
^{1}H-^{13}C HMBC spectrum (700.1 MHz) of compound **MC008** S67
HRMS spectrum of compound **MC008** S68
^{1}H NMR spectrum (700.1 MHz) of compound **MC009** S69
^{13}C NMR spectrum (700.1 MHz) of compound **MC009** S70
^{1}H-^{13}C HSQC spectrum (700.1 MHz) of compound **MC009** S71
^{1}H-^{13}C HMBC spectrum (700.1 MHz) of compound **MC009** S72
HRMS spectrum of compound **MC009** S73
^{1}H NMR spectrum (700.1 MHz) of compound **MC010** S74
^{13}C NMR spectrum (700.1 MHz) of compound **MC010** S75
^{1}H-^{13}C HSQC spectrum (700.1 MHz) of compound **MC010** S76
^{1}H-^{13}C HMBC spectrum (700.1 MHz) of compound **MC010** S77
HRMS spectrum of compound **MC010** S78
^{1}H NMR spectrum (700.1 MHz) of compound **MC011** S79
^{13}C NMR spectrum (700.1 MHz) of compound **MC011** S80
^{1}H-^{13}C HSQC spectrum (700.1 MHz) of compound **MC011** S81
^{1}H-^{13}C HMBC spectrum (700.1 MHz) of compound **MC011** S82
HRMS spectrum of compound **MC011** S83
^{1}H NMR spectrum (700.1 MHz) of compound **MC012** S84
^{13}C NMR spectrum (700.1 MHz) of compound **MC012** S85
^{1}H-^{13}C HSQC spectrum (700.1 MHz) of compound **MC012** S86
^{1}H-^{13}C HMBC spectrum (700.1 MHz) of compound **MC012** S87
HRMS spectrum of compound **MC012** S88
^{1}H NMR spectrum (700.1 MHz) of compound **MC013** S89
^{13}C NMR spectrum (700.1 MHz) of compound **MC013** S90
^{1}H-^{13}C HSQC spectrum (700.1 MHz) of compound **MC013** S91
^{1}H-^{13}C HMBC spectrum (700.1 MHz) of compound **MC013** S92
HRMS spectrum of compound **MC013** S93
^{1}H NMR spectrum (700.1 MHz) of compound **MC014** S94
^{13}C NMR spectrum (700.1 MHz) of compound **MC014** S95
^{1}H-^{13}C HSQC spectrum (700.1 MHz) of compound **MC014** S96
^{1}H-^{13}C HMBC spectrum (700.1 MHz) of compound **MC014** S97
HRMS spectrum of compound **MC014** S98
Relative binding affinity measurements
Stacked ^{1}H NMR spectra (700.1 MHz) of a mixture of receptors **CZ016**; 1,3-diindolylurea; 1,3-dicarbazolylurea + TBA-acetate S99
Stacked ^{1}H NMR spectra (700.1 MHz) of a mixture of receptors **MC001**; 2,7-(COOBu)2-indolocarbazole; 2,9-(COOBu)2-indolocarbazole + TBA-acetate S100
Stacked ^{1}H NMR spectra (700.1 MHz) of a mixture of receptors **MC002**; indolocarbazole; 2,7-(Cl)2-indolocarbazole + TBA-acetate S101
Stacked ^{1}H NMR spectra (700.1 MHz) of a mixture of receptors **MC003**; 2,7-(COOBu)2-indolocarbazole; 2,9-(COOBu)2-indolocarbazole + TBA-acetate S102
Stacked ^{1}H NMR spectra (700.1 MHz) of a mixture of receptors **MC004**; 1,3-diindolylurea; 1,3-dicarbazolylurea + TBA-acetate S103

Stacked ^{1}H NMR spectra (700.1 MHz) of a mixture of receptors **MC005**; 1,3-diindolylurea; 1,3-dicarbazolylurea + TBA-acetate S104
Stacked ^{1}H NMR spectra (700.1 MHz) of a mixture of receptors **MC006**; 1,3-diindolylurea; 1,3-dicarbazolylurea + TBA-acetate S105
Stacked ^{1}H NMR spectra (700.1 MHz) of a mixture of receptors **MC007**; 1,3-diindolylurea; 1,3-dicarbazolylurea + TBA-acetate S106
Stacked ^{1}H NMR spectra (700.1 MHz) of a mixture of receptors **MC008**; 1,3-diindolylurea; 1,3-dicarbazolylurea + TBA-acetate S107
Stacked ^{1}H NMR spectra (700.1 MHz) of a mixture of receptors **MC009**; **MC008**; 1,3-diindolylurea + TBA-acetate S108
Stacked ^{1}H NMR spectra (700.1 MHz) of a mixture of receptors **MC010**; 1,3-diindolylurea; 1,3-dicarbazolylurea + TBA-acetate S109
Stacked ^{1}H NMR spectra (700.1 MHz) of a mixture of receptors **MC011**; 1,3-diindolylurea; 1,3-dicarbazolylurea + TBA-acetate S110
Stacked ^{1}H NMR spectra (700.1 MHz) of a mixture of receptors **MC012**; 1,3-diindolylurea; 1,3-dicarbazolylurea + TBA-acetate S111
Stacked ^{1}H NMR spectra (700.1 MHz) of a mixture of receptors **MC013**; 1,3-diindolylurea; 1,3-dicarbazolylurea + TBA-acetate S112
Stacked ^{1}H NMR spectra (700.1 MHz) of a mixture of receptors **MC014**; 1,3-diindolylurea; 1,3-dicarbazolylurea + TBA-acetate S113
Stacked ^{1}H NMR spectra (700.1 MHz) of a mixture of receptors **CZ016**; **MC002**; 2,7-(COOBu)2-indolocarbazole; 2,9-(COOBu)2-indolocarbazole + TBA-benzoate S114
Stacked ^{1}H NMR spectra (700.1 MHz) of a mixture of receptors **MC001**; 2,7-(COOBu)2-indolocarbazole; 2,9-(COOBu)2-indolocarbazole + TBA-benzoate S115
Stacked ^{1}H NMR spectra (700.1 MHz) of a mixture of receptors **MC003**; 1,3-diindolylurea; 1,3-dicarbazolylurea + TBA-benzoate S116
Stacked ^{1}H NMR spectra (700.1 MHz) of a mixture of receptors **MC004**; 1,3-diindolylurea; 1,3-dicarbazolylurea + TBA-benzoate S117
Stacked ^{1}H NMR spectra (700.1 MHz) of a mixture of receptors **MC005**; 1,3-diindolylurea; 1,3-dicarbazolylurea + TBA-benzoate S118
Stacked ^{1}H NMR spectra (700.1 MHz) of a mixture of receptors **MC006**; 1,3-diindolylurea; 1,3-dicarbazolylurea + TBA-benzoate S119
Stacked ^{1}H NMR spectra (700.1 MHz) of a mixture of receptors **MC007**; 1,3-diindolylurea; 1,3-dicarbazolylurea + TBA-benzoate S120
Stacked ^{1}H NMR spectra (700.1 MHz) of a mixture of receptors **MC008**; 1,3-diindolylurea; 1,3-dicarbazolylurea + TBA-benzoate S121
Stacked ^{1}H NMR spectra (700.1 MHz) of a mixture of receptors **MC009**; 1,3-diindolylurea; 1,3-dicarbazolylurea + TBA-benzoate S122
Stacked ^{1}H NMR spectra (700.1 MHz) of a mixture of receptors **MC010**; 1,3-diindolylurea; 1,3-dicarbazolylurea + TBA-benzoate S123
Stacked ^{1}H NMR spectra (700.1 MHz) of a mixture of receptors **MC011**; 1,3-diindolylurea; 1,3-dicarbazolylurea + TBA-benzoate S124
Stacked ^{1}H NMR spectra (700.1 MHz) of a mixture of receptors **MC012**; 1,3-diindolylurea; 1,3-dicarbazolylurea + TBA-benzoate S125
Stacked ^{1}H NMR spectra (700.1 MHz) of a mixture of receptors **MC013**; 1,3-diindolylurea; 1,3-dicarbazolylurea + TBA-benzoate S126
Stacked ^{1}H NMR spectra (700.1 MHz) of a mixture of receptors **MC014**; 1,3-diindolylurea; 1,3-dicarbazolylurea + TBA-benzoate S127
Stacked ^{1}H NMR spectra (700.1 MHz) of a mixture of receptors **CZ016**; 2,7-(COOBu)2-indolocarbazole; 2,9-(COOBu)2-indolocarbazole + TBA-formate S128
Stacked ^{1}H NMR spectra (700.1 MHz) of a mixture of receptors **MC001**; **MC004**; 1,3-diindolylurea + TBA-formate S129

Stacked ^{1}H NMR spectra (700.1 MHz) of a mixture of receptors **MC002**; 1,3-diindolylurea; 1,3-dicarbazolylurea + TBA-formate S130
Stacked ^{1}H NMR spectra (700.1 MHz) of a mixture of receptors **MC003**; 1,3-diindolylurea; 1,3-dicarbazolylurea + TBA-formate S131
Stacked ^{1}H NMR spectra (700.1 MHz) of a mixture of receptors **MC005**; 1,3-diindolylurea; 1,3-dicarbazolylurea + TBA-formate S132
Stacked ^{1}H NMR spectra (700.1 MHz) of a mixture of receptors **MC006**; **MC008**; 1,3-diindolylurea; 1,3-dicarbazolylurea + TBA-formate S133
Stacked ^{1}H NMR spectra (700.1 MHz) of a mixture of receptors **MC007**; 1,3-diindolylurea; 1,3-dicarbazolylurea + TBA-formate S134
Stacked ^{1}H NMR spectra (700.1 MHz) of a mixture of receptors **MC008**; **MC009**; 1,3-diindolylurea; 1,3-dicarbazolylurea + TBA-formate S135
Stacked ^{1}H NMR spectra (700.1 MHz) of a mixture of receptors **MC010**; 1,3-diindolylurea; 1,3-dicarbazolylurea + TBA-formate S136
Stacked ^{1}H NMR spectra (700.1 MHz) of a mixture of receptors **MC011**; 1,3-diindolylurea; 1,3-dicarbazolylurea + TBA-formate S137
Stacked ^{1}H NMR spectra (700.1 MHz) of a mixture of receptors **MC012**; 1,3-diindolylurea; 1,3-dicarbazolylurea + TBA-formate S138
Stacked ^{1}H NMR spectra (700.1 MHz) of a mixture of receptors **MC013**; 1,3-diindolylurea; 1,3-dicarbazolylurea + TBA-formate S139
Stacked ^{1}H NMR spectra (700.1 MHz) of a mixture of receptors **MC014**; 1,3-diindolylurea; 1,3-dicarbazolylurea + TBA-formate S140
Stacked ^{1}H NMR spectra (700.1 MHz) of a mixture of receptors **CZ016**; 1,3-diindolylurea; 1,3-dicarbazolylurea + TBA-lactate S141
Stacked ^{1}H NMR spectra (700.1 MHz) of a mixture of receptors **MC001**; 2,7-(COOBu)2-indolocarbazole; 2,9-(COOBu)2-indolocarbazole + TBA-lactate S142
Stacked ^{1}H NMR spectra (700.1 MHz) of a mixture of receptors **MC003**; 1,3-diindolylurea; 1,3-dicarbazolylurea + TBA-lactate S143
Stacked ^{1}H NMR spectra (700.1 MHz) of a mixture of receptors **MC004**; 1,3-diindolylurea; 1,3-dicarbazolylurea + TBA-lactate S144
Stacked ^{1}H NMR spectra (700.1 MHz) of a mixture of receptors **MC005**; 1,3-diindolylurea; 1,3-dicarbazolylurea + TBA-lactate S145
Stacked ^{1}H NMR spectra (700.1 MHz) of a mixture of receptors **MC006**; 1,3-diindolylurea; 1,3-dicarbazolylurea + TBA-lactate S146
Stacked ^{1}H NMR spectra (700.1 MHz) of a mixture of receptors **MC007**; 1,3-diindolylurea; 1,3-dicarbazolylurea + TBA-lactate S147
Stacked ^{1}H NMR spectra (700.1 MHz) of a mixture of receptors **MC008**; 1,3-diindolylurea; 1,3-dicarbazolylurea + TBA-lactate S148
Stacked ^{1}H NMR spectra (700.1 MHz) of a mixture of receptors **MC009**; 1,3-diindolylurea; 1,3-dicarbazolylurea + TBA-lactate S149
Stacked ^{1}H NMR spectra (700.1 MHz) of a mixture of receptors **MC010**; 1,3-diindolylurea; 1,3-dicarbazolylurea + TBA-lactate S150
Stacked ^{1}H NMR spectra (700.1 MHz) of a mixture of receptors **MC011**; 1,3-diindolylurea; 1,3-dicarbazolylurea + TBA-lactate S151
Stacked ^{1}H NMR spectra (700.1 MHz) of a mixture of receptors **MC012**; 1,3-diindolylurea; 1,3-dicarbazolylurea + TBA-lactate S152
Stacked ^{1}H NMR spectra (700.1 MHz) of a mixture of receptors **MC013**; 1,3-diindolylurea; 1,3-dicarbazolylurea + TBA-lactate S153
Stacked ^{1}H NMR spectra (700.1 MHz) of a mixture of receptors **MC014**; 1,3-diindolylurea; 1,3-dicarbazolylurea + TBA-lactate S154
Stacked ^{1}H NMR spectra (700.1 MHz) of a mixture of receptors **CZ016**; 1,3-diindolylurea; 1,3-dicarbazolylurea + TBA-pivalate S155

Stacked ^1H NMR spectra (700.1 MHz) of a mixture of receptors **MC001**; 2,7-(COOBu)2-indolocarbazole; 2,9-(COOBu)2-indolocarbazole + TBA-pivalate S156
Stacked ^1H NMR spectra (700.1 MHz) of a mixture of receptors **MC003**; 1,3-diindolylurea; 1,3-dicarbazolylurea + TBA-pivalate S157
Stacked ^1H NMR spectra (700.1 MHz) of a mixture of receptors **MC004**; 3,4,4-Cl3-diphenylurea; 2,7-(COOBu)2-indolocarbazole; 2,9-(COOBu)2-indolocarbazole + TBA-pivalate S158
Stacked ^1H NMR spectra (700.1 MHz) of a mixture of receptors **MC005**; 1,3-diindolylurea; 1,3-dicarbazolylurea + TBA-pivalate S159
Stacked ^1H NMR spectra (700.1 MHz) of a mixture of receptors **MC006**; 2,7-(COOBu)2-indolocarbazole; 2,9-(COOBu)2-indolocarbazole + TBA-pivalate S160
Stacked ^1H NMR spectra (700.1 MHz) of a mixture of receptors **MC007**; 1,3-diindolylurea; 1,3-dicarbazolylurea + TBA-pivalate S161
Stacked ^1H NMR spectra (700.1 MHz) of a mixture of receptors **MC008**; 1,3-diindolylurea; 1,3-dicarbazolylurea + TBA-pivalate S162
Stacked ^1H NMR spectra (700.1 MHz) of a mixture of receptors **MC009**; 1,3-diindolylurea; 1,3-dicarbazolylurea + TBA-pivalate S163
Stacked ^1H NMR spectra (700.1 MHz) of a mixture of receptors **MC010**; 1,3-diindolylurea; 1,3-dicarbazolylurea + TBA-pivalate S164
Stacked ^1H NMR spectra (700.1 MHz) of a mixture of receptors **MC011**; 1,3-diindolylurea; 1,3-dicarbazolylurea + TBA-pivalate S165
Stacked ^1H NMR spectra (700.1 MHz) of a mixture of receptors **MC012**; 1,3-diindolylurea; 1,3-dicarbazolylurea + TBA-pivalate S166
Stacked ^1H NMR spectra (700.1 MHz) of a mixture of receptors **MC013**; 1,3-diindolylurea; 1,3-dicarbazolylurea + TBA-pivalate S167
Stacked ^1H NMR spectra (700.1 MHz) of a mixture of receptors **MC014**; 1,3-diindolylurea; 1,3-dicarbazolylurea + TBA-pivalate S168
UV–vis spectra of absolute binding affinity measurement for **MC002** with TBA-lactate in 99.5%:0.5% m/m DMSO-H_2O S169
UV–vis spectra of absolute binding affinity measurement for **MC002** with TBA-pivalate 99.5%:0.5% m/m DMSO-H_2O S169
UV–vis spectra of absolute binding affinity measurement for **MC004** with TBA-acetate 90.0%:10.0% m/m DMSO-H_2O S170
UV–vis spectra of absolute binding affinity measurement for **MC005** with TBA-acetate 90.0%:10.0% m/m DMSO-H_2O S170
UV–vis spectra of absolute binding affinity measurement for **MC007** with TBA-acetate 90.0%:10.0% m/m DMSO-H_2O S171
UV–vis spectra of absolute binding affinity measurement for **MC008** with TBA-acetate 90.0%:10.0% m/m DMSO-H_2O S171
UV–vis spectra of absolute binding affinity measurement for **MC009** with TBA-acetate 90.0%:10.0% m/m DMSO-H_2O S172
UV–vis spectra of absolute binding affinity measurement for **MC004** with TBA-benzoate 90.0%:10.0% m/m DMSO-H_2O S172
UV–vis spectra of absolute binding affinity measurement for **MC005** with TBA-benzoate 90.0%:10.0% m/m DMSO-H_2O S173
UV–vis spectra of absolute binding affinity measurement for **MC007** with TBA-benzoate 90.0%:10.0% m/m DMSO-H_2O S173
UV–vis spectra of absolute binding affinity measurement for **MC008** with TBA-benzoate 90.0%:10.0% m/m DMSO-H_2O S174
UV–vis spectra of absolute binding affinity measurement for **MC009** with TBA-benzoate 90.0%:10.0% m/m DMSO-H_2O S174
UV–vis spectra of absolute binding affinity measurement for **MC004** with TBA-formate 90.0%:10.0% m/m DMSO-H_2O S175

UV–vis spectra of absolute binding affinity measurement for **MC005** with TBA-formate 90.0%:10.0% m/m DMSO-H_2O S175
UV–vis spectra of absolute binding affinity measurement for **MC007** with TBA-formate 90.0%:10.0% m/m DMSO-H_2O S176
UV–vis spectra of absolute binding affinity measurement for **MC008** with TBA-formate 90.0%:10.0% m/m DMSO-H_2O S176
UV–vis spectra of absolute binding affinity measurement for **MC009** with TBA-formate 90.0%:10.0% m/m DMSO-H_2O S177
UV–vis spectra of absolute binding affinity measurement for **MC004** with TBA-lactate 90.0%:10.0% m/m DMSO-H_2O S177
UV–vis spectra of absolute binding affinity measurement for **MC005** with TBA-lactate 90.0%:10.0% m/m DMSO-H_2O S178
UV–vis spectra of absolute binding affinity measurement for **MC007** with TBA-lactate 90.0%:10.0% m/m DMSO-H_2O S178
UV–vis spectra of absolute binding affinity measurement for **MC008** with TBA-lactate 90.0%:10.0% m/m DMSO-H_2O S179
UV–vis spectra of absolute binding affinity measurement for **MC009** with TBA-lactate 90.0%:10.0% m/m DMSO-H_2O S179
UV–vis spectra of absolute binding affinity measurement for **MC004** with TBA-pivalate 90.0%:10.0% m/m DMSO-H_2O S180
UV–vis spectra of absolute binding affinity measurement for **MC005** with TBA-pivalate 90.0%:10.0% m/m DMSO-H_2O S180
UV–vis spectra of absolute binding affinity measurement for **MC007** with TBA-pivalate 90.0%:10.0% m/m DMSO-H_2O S181
UV–vis spectra of absolute binding affinity measurement for **MC008** with TBA-pivalate 90.0%:10.0% m/m DMSO-H_2O S181
UV–vis spectra of absolute binding affinity measurement for **MC009** with TBA-pivalate 90.0%:10.0% m/m DMSO-H_2O S182
Cosmo-RS geometry pictures
Lowest energy conformation of receptor **CZ016** S183
Lowest energy conformation of receptor **CZ016** with pivalate anion S183
Lowest energy conformation of receptor **CZ016** with acetate anion S184
Lowest energy conformation of receptor **CZ016** with benzoate anion S184
Lowest energy conformation of receptor **CZ016** with lactate anion S185
Lowest energy conformation of receptor **CZ016** with formate anion S185
Lowest energy conformation of receptor **MC001** S186
Lowest energy conformation of receptor **MC001** with pivalate anion S186
Lowest energy conformation of receptor **MC001** with acetate anion S187
Lowest energy conformation of receptor **MC001** with benzoate anion S187
Lowest energy conformation of receptor **MC001** with lactate anion S188
Lowest energy conformation of receptor **MC001** with formate anion S188
Lowest energy conformation of receptor **MC002** S189
Lowest energy conformation of receptor **MC002** with pivalate anion S189
Lowest energy conformation of receptor **MC002** with acetate anion S190
Lowest energy conformation of receptor **MC002** with benzoate anion S190
Lowest energy conformation of receptor **MC002** with lactate anion S191
Lowest energy conformation of receptor **MC002** with formate anion S191
Lowest energy conformation of receptor **MC003** S192
Lowest energy conformation of receptor **MC003** with pivalate anion S192

Lowest energy conformation of receptor **MC003** with acetate anion S193
Lowest energy conformation of receptor **MC003** with benzoate anion S193
Lowest energy conformation of receptor **MC003** with lactate anion S194
Lowest energy conformation of receptor **MC003** with formate anion S194
Lowest energy conformation of receptor **MC004** S195
Lowest energy conformation of receptor **MC004** with pivalate anion S195
Lowest energy conformation of receptor **MC004** with acetate anion S196
Lowest energy conformation of receptor **MC004** with benzoate anion S196
Lowest energy conformation of receptor **MC004** with lactate anion S197
Lowest energy conformation of receptor **MC004** with formate anion S197
Lowest energy conformation of receptor **MC005** S198
Lowest energy conformation of receptor **MC005** with pivalate anion S198
Lowest energy conformation of receptor **MC005** with acetate anion S199
Lowest energy conformation of receptor **MC005** with benzoate anion S199
Lowest energy conformation of receptor **MC005** with lactate anion S200
Lowest energy conformation of receptor **MC005** with formate anion S200
Lowest energy conformation of receptor **MC006** S201
Lowest energy conformation of receptor **MC006** with pivalate anion S201
Lowest energy conformation of receptor **MC006** with acetate anion S202
Lowest energy conformation of receptor **MC006** with benzoate anion S202
Lowest energy conformation of receptor **MC006** with lactate anion S203
Lowest energy conformation of receptor **MC006** with formate anion S203
Lowest energy conformation of receptor **MC007** S204
Lowest energy conformation of receptor **MC007** with pivalate anion S204
Lowest energy conformation of receptor **MC007** with acetate anion S205
Lowest energy conformation of receptor **MC007** with benzoate anion S205
Lowest energy conformation of receptor **MC007** with lactate anion S206
Lowest energy conformation of receptor **MC007** with formate anion S206
Lowest energy conformation of receptor **MC008** S207
Lowest energy conformation of receptor **MC008** with pivalate anion S207
Lowest energy conformation of receptor **MC008** with acetate anion S208
Lowest energy conformation of receptor **MC008** with benzoate anion S208
Lowest energy conformation of receptor **MC008** with lactate anion S209
Lowest energy conformation of receptor **MC008** with formate anion S209
Lowest energy conformation of receptor **MC009** S210
Lowest energy conformation of receptor **MC009** with pivalate anion S210
Lowest energy conformation of receptor **MC009** with acetate anion S211
Lowest energy conformation of receptor **MC009** with benzoate anion S211
Lowest energy conformation of receptor **MC009** with lactate anion S212
Lowest energy conformation of receptor **MC009** with formate anion S212
Lowest energy conformation of receptor **MC010** S213
Lowest energy conformation of receptor **MC010** with pivalate anion S213
Lowest energy conformation of receptor **MC010** with acetate anion S214
Lowest energy conformation of receptor **MC010** with benzoate anion S214
Lowest energy conformation of receptor **MC010** with lactate anion S215
Lowest energy conformation of receptor **MC010** with formate anion S215

Lowest energy conformation of receptor **MC011** S216
Lowest energy conformation of receptor **MC011** with pivalate anion S216
Lowest energy conformation of receptor **MC011** with acetate anion S216
Lowest energy conformation of receptor **MC011** with benzoate anion S217
Lowest energy conformation of receptor **MC011** with lactate anion S217
Lowest energy conformation of receptor **MC011** with formate anion S217
Lowest energy conformation of receptor **MC012** S218
Lowest energy conformation of receptor **MC012** with pivalate anion S218
Lowest energy conformation of receptor **MC012** with acetate anion S218
Lowest energy conformation of receptor **MC012** with benzoate anion S219
Lowest energy conformation of receptor **MC012** with lactate anion S219
Lowest energy conformation of receptor **MC012** with formate anion S219
Lowest energy conformation of receptor **MC013** S220
Lowest energy conformation of receptor **MC013** with pivalate anion S220
Lowest energy conformation of receptor **MC013** with acetate anion S221
Lowest energy conformation of receptor **MC013** with benzoate anion S221
Lowest energy conformation of receptor **MC013** with lactate anion S222
Lowest energy conformation of receptor **MC013** with formate anion S222
Lowest energy conformation of receptor **MC014** S222
Lowest energy conformation of receptor **MC014** with pivalate anion S223
Lowest energy conformation of receptor **MC014** with acetate anion S223
Lowest energy conformation of receptor **MC014** with benzoate anion S223
Lowest energy conformation of receptor **MC014** with lactate anion S224
Lowest energy conformation of receptor **MC014** with formate anion S224

General methods

General purification procedures

Purification of the compounds was performed by column chromatography on silica gel (pore size 60 Å, 230–400 mesh for macrocycles **MC001**–**MC010** and 70–230 mesh for macrocycles **MC011**–**MC014**). Analytical thin-layer chromatography (TLC) was conducted on TLC plates (silica gel 60 with fluorescent UV_{254} marker on aluminium backed sheets).

Chemicals and raw materials

All starting materials and solvents used in syntheses were acquired from commercial sources (Sigma-Aldrich, Acros, Alfa Aesar, Fluka) with at least 97% purity and used as received. DMSO-d_6 for NMR was obtained from Sigma-Aldrich with at least 99.9% purity. Dry solvents were prepared at least 72 h before use in round-bottomed flasks supplied with 3 Å molecular sieves under an argon atmosphere.

All inert gases used for syntheses were of at least 5.0 purity. Water used in this work was obtained from a MilliQ Advantage A10 system or from an ELGA PURELAB® Ultra system.

For sensor development, the following chemicals were of Selectophore™ grade: bis(2-ethylhexyl) sebacate (DOS, ≥97%, Fluka), tridodecylmethylammonium chloride (TDMACl, Fluka), high molecular weight poly(vinyl chloride) (HMW PVC, Fluka), and tetrahydrofuran (THF, ≥99.5%, Sigma-Aldrich). Nitric acid (65%, J.T. Baker) was diluted for use in the electrode cleaning procedure. The remaining chemicals were of analytical or reagent grade with at least 99% purity, except for 3,4-ethylenedioxythiophene (EDOT, 97%), and acquired from Acros Organics, Alfa Aesar, Fluka, Merck, Sigma-Aldrich, and VWR Chemicals.

General procedures for compound characterization

NMR measurements, characterization and assessment of purity of all synthesis products were carried out on a Bruker Avance-III 700 MHz NMR spectrometer. All spectra were recorded using TopSpin 3.2 software and the chemical shifts calibrated against the residual solvent signal.

COSMO-RS calculations were done using COSMOThermX19 parametrization BP_TZVP_C19 (solvent: DMSO with 0.5% water). UV–vis spectrophotometric measurements were carried out using a Thermo Nicolet Evolution 300 spectrophotometer.

For electrospray (ESI) HRMS analysis, the samples were first dissolved in DMSO with a concentration of ≈ 2 mg/ml and then diluted in MeOH containing 0.1% HCOOH ($pH_{abs}^{H_2O}$ [1] in the range of 3–5) for ESI+ measurements or MeOH containing 0.1% $NH_3{\cdot}H_2O$ ($pH_{abs}^{H_2O}$ in the range of 8–9) for ESI− measurements. Concentrations of compounds were chosen in such way that appropriate signal intensities were achieved and were in the range of 0.5–2 µg/mL. HRMS spectra were obtained on a hybrid Varian 910-FT-ICR-MS system with electrospray ion source. Ionization parameters were as follows: spray chamber temperature, 40 °C; spray needle voltage, 5000 V (−4500 V in negative mode); nebulizing gas (N_2) pressure, 30 psi; API-drying gas (N_2), 15 psi at 300 °C; shield voltage, 600 V; capillary voltage 40 V. The ions, selected by quadrupole, were guided into the FT-ICR analyser cell. Ion guide parameters, FT-ICR ion guide and excitation parameters were optimized for the mass range (*m*/*z* = 100–1000). Ion collection time varied from 300 to 1000 ms. FT-ICR analyser cell parameters were: DAC rate 8000 kHz for *m*/*z* range of 100–800 direct (broadband); ADC rate 4 MHz; transient length was either 1024 K (262.144 ms) or 2048 K (524.288 ms). For the calibration of the mass axis, samples were spiked with an in-house prepared internal calibration solution, which contained ions with the following exact *m*/*z* values [2]: $C_{16}H_{36}N^+$ (*m*/*z* = 242.28423); $C_{19}H_{29}N_4PF_3^+$ (*m*/*z*

= 401.20765); $C_{26}H_{45}N_7P_2Cl^+$ (m/z = 552.28947); $C_{26}H_{64}N_{13}P_4^+$ (m/z = 682.43526) in ESI+ mode. For ESI− measurements, a calibration mixture containing anions of perfluorinated Brønsted superacids [3] was used. The ions used for calibration were: $C_{12}F_{10}NO_4S_2^-$ (m/z = 475.91145); $C_8F_{17}NO_2SH^-$ (m/z = 497.94620); $C_8F_{18}NO_4S_2^-$ (m/z = 579.89868); $C_{12}F_{26}NO_4S_2^-$ (m/z = 779.88591). Concentrations of the calibrants in the infused solutions remained within 0.5–1.0 μM.

For nano-ESIMS analysis, the solution was infused using a borosilicate emitter (Thermo Fisher Scientific) to an LTQ Orbitrap XL (Thermo Fisher Scientific) mass spectrometer using a spray voltage of 1.8 kV (tube lens 110 V, capillary temperature 200 °C). The instrument was operated in positive polarity. Ten mass spectra were collected at a resolution setting of 100,000 at 400 *m/z*, spectra were averaged, and the presence of expected compounds was confirmed by mass accuracy within +/− 5 ppm and by similarity to theoretical isotopic distribution.

The reference electrode that was used in all electrochemical measurements was a 6.0726.100 double-junction Ag/AgCl reference electrode (Metrohm AG, Switzerland). Glassy carbon (GC) rods (SIGRADUR® G, HTW Hochtemperatur-Werkstoffe GmbH, Germany) were used as counter electrodes and to prepare working electrodes in-house by fitting them into PVC rods (Simona AG, Germany). All electrochemical polymerisations of solid contacts and impedance measurements were performed with Autolab PGSTAT20 or PGSTAT30 potentiostats (EcoChemie BV, Netherlands) equipped with a FRA2 modules. An EMF16 multichannel interface (Lawson Labs, Inc., United States of America) was used for all potentiometric measurements. A pair of 800 Dosino pumps controlled by a 905 Titrando (Metrohm AG, Switzerland) were used for automated dilutions. pH measurements were performed with a Thermo Scientific Orion Star A111 pH-meter and a Thermo Scientific Orion 9157BNMD pH-electrode. All measurements were performed at room temperature (23 ± 2 °C).

Relative binding affinity measurements

Binding of anions in solution was studied using our previously published relative ^{1}H NMR titration method.[4] For sample preparation, a few milligrams of receptors were weighed into a NMR tube alongside an anchor molecule, to which binding had previously been quantified using absolute binding measurements.[4–6] The compounds were dissolved in 700 µL DMSO-d_6 with a water content of either 0.5% or 10.0% (m/m) H_2O. Solutions of carboxylates were prepared by weighing the corresponding tetrabutylammonium salt into a vial and dissolving it in 1 mL DMSO-d_6 with a water content of either 0.5% or 10.0% (m/m) H_2O. For each analyte, two solutions were prepared: a dilute (0.17–0.44 M) and a concentrated (0.65–1.41 M) solution. During the titration, a blank spectrum of pure receptors was initially obtained. Then, the analyte was added using an automatic titration syringe. The chemical shifts of the formed host–guest complexes were recorded for each titration step. The dilute solution was used during the first part of the titration, where complexation was partial. The concentrated sample was used towards the end of the titration to ensure full complexation of all host molecules. The recorded chemical shifts of all titration steps were used to calculate the differences of affinity to the anchor molecules, as described in reference [4]. Spectra of all titration experiments are available as supporting information.

Preparation of the membrane cocktails

The solvent polymeric membranes were prepared using PVC, DOS as the plasticiser, and TDMACl as the anion exchanger. The ion-selective membranes (ISMs) were prepared with the following composition as the target: 2 wt % ionophore, 50 mol % TDMACl relative to the ionophore, 65 wt % DOS, and 32 wt % PVC. A control membrane was prepared with no ionophore, 0.7 wt % TDMACl, 66 wt % DOS, and 33 wt % PVC. The membrane components were dissolved in THF to produce membrane cocktails containing 83 wt % THF. See Table S1 for the final compositions of the membrane cocktails.

Table S1: Compositions of the membrane cocktails used in this study

component	wt.%			
	**CTRL**	**MC005**	**MC009**	**MC012**
ionophore	0.00	0.33	0.33	0.33
TDMACl	0.12	0.12	0.11	0.12
PVC	5.77	5.40	5.30	5.45
DOS	11.22	10.75	11.03	10.80
dry (total)	17.11	16.61	16.78	16.69
THF	82.89	83.39	83.22	83.31

Preparation of the sensor prototypes

Glassy carbon rods (3 mm diameter) encased in PVC (8 mm diameter) were used as the electrode bodies for the sensors. The electrodes were polished using sandpapers, diamond pastes (1–15 μm), and alumina paste (0.3 μm). The electrodes were cleaned prior to electrochemical polymerisation of the solid contact by ultrasonication in deionized water and in ethanol and soaking in 1 M nitric acid. A layer of PEDOT doped with chloride was deposited as the solid contact onto the exposed GC surface ($A \approx 0.07\ cm^2$) of each electrode. The polymerisations were performed one sensor at a time with a three-electrode cell using a double-junction Ag/AgCl/3 M KCl/1 M KCl reference electrode, a bare GC rod as the counter electrode, and the sensor as the working electrode. The monomer solution, which contained 0.01 M EDOT and 0.1 M KCl, was deaerated by bubbling N_2 through the solution prior to the polymerisations and then blanketed with N_2 during the polymerisations. The polymerisations were performed galvanostatically by applying a constant current of 14 μA for 714 s (10 mC polymerisation charge). The sensors were conditioned in 0.1 M sodium acetate at least overnight and then air-dried prior to drop-casting the membranes. The membranes were drop-cast onto the sensors in two passes of 50 μL with approximately 30 minutes between passes for a total of 100 μL of membrane cocktail applied to each sensor. The THF was allowed to evaporate overnight and then the sensors were conditioned in 0.1 M sodium acetate for at least three days prior to characterisation. Three sensors were prepared with each membrane for this study.

Electrochemical impedance spectroscopy

The sensors were conditioned in 0.1 M sodium acetate prior to the electrochemical impedance spectroscopic (EIS) measurements. EIS measurements were performed in 0.1 M sodium acetate with a three-electrode cell using a double-junction Ag/AgCl/3 M KCl/1 M KCl reference

electrode, a GC rod as the counter electrode, and the sensors as the working electrodes. The measurements were performed potentiostatically at 0 V versus the open-circuit potential using a sinusoidal excitation signal with an amplitude of 100 mV (RMS). Sixty-one measurement points were measured in the frequency range 100 mHz–100 kHz.

Potentiometric measurements

The sensors were conditioned in 0.01 M sodium acetate prior to all of the potentiometric measurements. Liquid junction potentials were estimated using the Henderson equation. Activity coefficients were estimated using the extended Debye–Hückel equation.

Calibration

Potentiometric calibrations were performed with 0.1 M sodium acetate solutions that were diluted with deionized water in half-decade steps. The dilutions were performed at seven-minute intervals with pre-programmed pumps until reaching concentrations where the sensors' potentiometric responses levelled out. Mixing of the solutions was left to the pumping action that occurred during the dilutions. Multiple calibrations were performed with each sensor over the course of their life spans.

pH Sensitivity

The pH sensitivity of the sensors was assessed by measuring their potentiometric responses in 0.01 M acetic acid that was titrated with a mixture of 0.25 M sodium hydroxide and 0.01 M sodium acetate. The pH of the samples was monitored during the measurements and the samples were titrated from pH 3.3 to 10 with constant stirring.

Selectivity

Potentiometric selectivity coefficients, $K_{\text{acetate,j}}^{\text{pot}}$, were determined according to the separate solution method (SSM) using solutions with activities of interfering ions equal to that of acetate in a sodium acetate solution with the concentration 0.01 M. The solutions were deaerated by bubbling N_2 through each solution for at least 15 minutes prior to each measurement and then letting N_2 blanket the solution during each measurement. The deaeration was done as a precaution to minimise possible interference due to species originating from dissolved atmospheric carbon dioxide. Constant stirring was used throughout each measurement. The reference electrode was placed in the sample solution after rinsing the sensors with deionized water and drying them. The measurement was started, the sensors were immersed in the solution, and the potentials were measured for at least seven minutes. The measurements were performed with the interfering anion solutions in the order of increasing lipophilicity. The selectivity coefficients were calculated using the experimental slopes determined for each electrode and the potentials that were recorded seven minutes into each selectivity measurement.

Synthesis and characterisation of compounds

Compound **2** was prepared as in reference [7]. Compounds **6–8** were prepared based on reference [8], but with slight modifications (please refer to Scheme 1 in main text).

1,8-Dibromo-3,6-di-*tert*-butyl-9*H*-carbazole (4) was prepared according to known procedure [9].

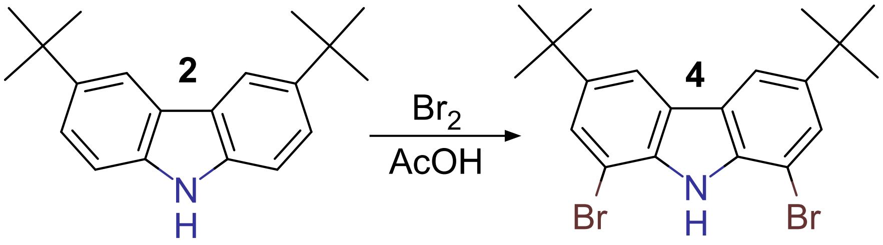

For preparation of 3,6-di-*tert*-butyl-9*H*-carbazole-1,8-diamine (**5a**), a general procedure from the work of Jung *et al.* [10] was modified as follows:

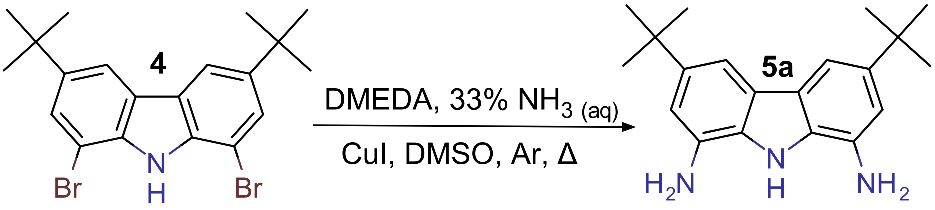

A 100-mL pressure tube (withstanding a maximum pressure of 10 bar at 120 °C) was charged with a stirring bar, 1,8-dibromo-3,6-di-*tert*-butyl-9*H*-carbazole (compound **4**, 2.00 g, 4.57 mmol) and CuI (261 mg, 1.37 mmol). DMSO (24 mL) was added and argon gas was bubbled through for 5 minutes while the mixture was magnetically stirred. Under argon counterflow, *N*,*N'*-dimethylethylenediamine (221 µL, 2.06 mmol) was added, and argon was bubbled through the mixture for an additional minute. After that, the argon flow was stopped and the flask was closed with a screwcap. The contents were magnetically stirred for 20 minutes and then cooled in a water bath (10–15 °C). The stirring was stopped and the screwcap removed, then under an argon counterflow, 12 mL of 33% aqueous NH_3 solution were carefully

added to the mixture by tube sidewalls without disturbing the contents. Argon flow was removed and the pressure tube was tightly closed with a screwcap. The mixture was vigorously stirred in an oil bath at 130 °C for 18 h. After that, the tube was cooled to approximately 15 °C and its contents were diluted with 100 mL of ethyl acetate. The mixture was filtrated and brine (250 mL) was added to the filtrate. Phases were separated and the aqueous phase was extracted with ethyl acetate (3 × 50 mL). The combined organic phases were washed with brine, dried over anhydrous Na_2SO_4, filtered and solvents removed by evaporation. The resulting residue was triturated with 10 mL DCM, then 100 mL of *n*-hexane was added and the mixture cooled in an ice bath. The precipitate was filtered and washed three times with cold *n*-hexane/ DCM 10:1 mixture to yield tan crystals (1.20 g, 3.88 mmol, 85%), the structure of which was confirmed to be identical with literature data [8].

General procedure for preparation of diacyl chlorides ($ClCO(CH_2)_nCOCl$, n = 11–14):

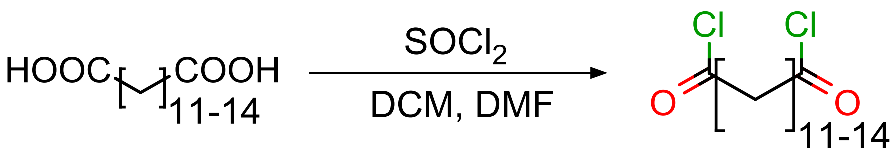

An oven-dried 10-mL round-bottomed flask was charged with a dicarboxylic acid (1 mmol) and a magnetic stirring bar. Then, 2 mL of dry DCM and 1 drop of dry DMF were added. The flask was flushed with argon and closed with a septum through which an argon-filled balloon was connected. The mixture was stirred and thionyl chloride (0.29 ml, 4 mmol) was added dropwise through the septum via syringe. After 4 h of stirring at room temperature the reaction was complete by ^1H NMR analysis. Solvents and residual $SOCl_2$ were evaporated, the residue dried in vacuo and used in further experiments without additional purification.

Preparation of compound CZ016

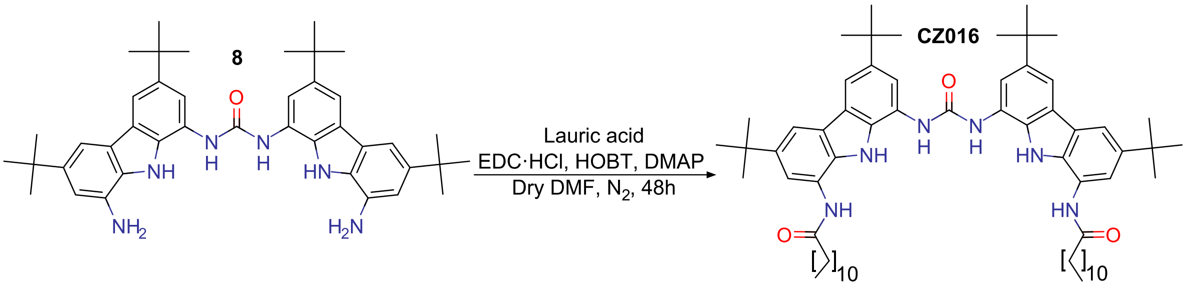

Under N_2 atmosphere, compound **8** (0.050 g, 0.08 mmol), lauric acid (0.038 g, 0.08 mmol), EDC·HCl (0.036 g, 0.19 mmol), HOBt (0.029 g, 0.19 mmol) and DMAP (0.023 g, 0.19 mmol) were dissolved in dry DMF (4 mL). The reaction mixture was stirred for 48 h at room temperature. After disappearance of starting material (monitored by TLC), the reaction mixture was quenched in water (50 mL). The formed precipitate was filtered and washed with water (50 mL). The crude product was purified by column chromatography eluting with 7–9% ethyl acetate in hexane to get compound **CZ016** (0.050 g, 0.05 mmol, 63.8%) as brown solid.

Data for **CZ016**. ^{1}H NMR (700.1 MHz, DMSO-*d*$_6$, +25 °C) δ: 10.10 (bs, 2H, amide NH), 10.00 (bs, 2H, carb. NH), 8.89 (bs, 2H, urea NH), 7.95 (s, 2H, CH-5), 7.93 (s, 2H, CH-4), 7.68 (s, 2H, CH-7), 7.53 (s, 2H, CH-2), 2.40-2.35 (m, 4H, -CO-CH_2), 1.61-1.56 (m, 4H, -CO-CH_2-CH_2), 1.42 (s, 18H, *t-butyl*-CH_{3a} or CH_{3b}), 1.40 (s, 18H, *t-butyl*-CH_{3a} or CH_{3b}), 1.37-1.20 (m, 32H, (CH_2)$_8$), 0.91-0.81 (m, 6H, -CH_2-CH_3). ^{13}C NMR (176.0 MHz, DMSO-d$_6$, +25 °C) δ: 171.5 (amide CO), 153.7 (urea CO), 141.8 (C-3), 141.5 (C-6), 131.2 (C-12), 130.5 (C-9), 124.6 (C-10), 124.5 (C-11), 123.0 (C-1), 122.6 (C-8), 116.5 (C-7), 116.1 (C-2), 112.1 (C-5), 111.9 (C-4), 36.1 (-CO-CH_2), 34.5 (*t-butyl*-C_a or C_b), 34.4 (*t-butyl*-C_a or C_b), 31.9, 31.8, 31.3, 29.0, 29.0, 28.9, 28.8, 28.7, 28.7, 25.2, 22.1, 13.9 (-CH_2-CH_3). ESI-FT-ICR (-): solvent ~ 0.01% DMSO:methanol, *m*/*z* of $[M{-}H]^-$ calculated for $C_{65}H_{95}N_6O_3$, 1007.74711 found 1007.74706.

Preparation of compound MC001

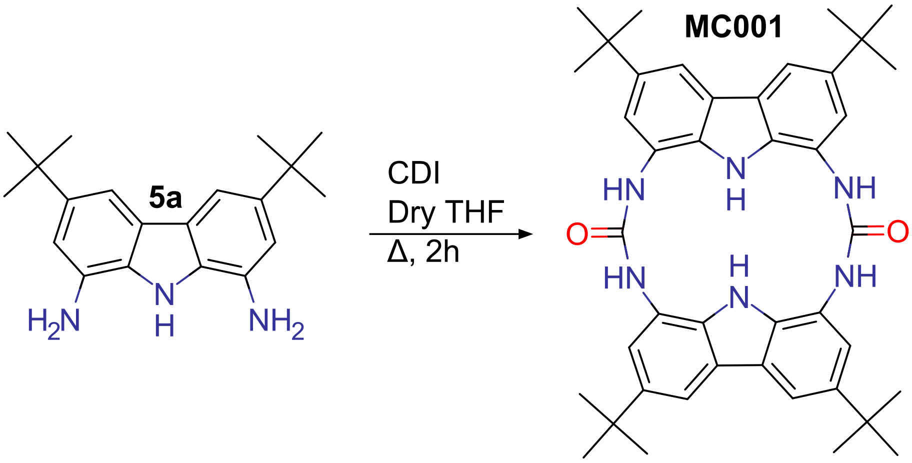

Compound **5a** (0.075 g, 0.24 mmol) was dissolved in dry THF (4 mL), then CDI (0.10 g, 0.43 mmol) was added. The reaction mixture was stirred at reflux temperature for 2 h. After disappearance of the starting material (monitored by TLC), the reaction mixture was cooled and concentrated under reduced pressure. The obtained solid was quenched in H_2O (100 mL) and extracted in ethyl acetate (50 mL). The ethyl acetate layer was concentrated under reduced pressure. The crude product was purified by column chromatography eluting with 1% MeOH in DCM to obtain compound **MC001** (0.070 g, 0.10 mmol, 83.3%) as off-white solid.

Data for **MC001**. ^{1}H NMR (700.1 MHz, DMSO-d_6, +25 °C) δ: 9.87 (bs, 2H, carb. N<u>H</u>), 9.20 (bs, 4H, amide N<u>H</u>), 8.01 (d, *J* = 1.4 Hz, 4H, C<u>H</u>-4), 7.16 (d, *J* = 2.1 Hz, 4H, C<u>H</u>-2), 1.44 (s, 36H, *t-butyl*-C<u>H</u>$_3$). ^{13}C NMR (176.0 MHz, DMSO-d_6, +25 °C) δ: 153.7 (urea <u>C</u>O), 143.2 (<u>C</u>-3), 134.4 (<u>C</u>-6), 126.9 (<u>C</u>-5), 123.9 (<u>C</u>-1), 117.2 (<u>C</u>-2), 113.5 (<u>C</u>-4), 34.9 (*t-butyl*-<u>C</u>), 32.3 (*t-butyl*-<u>C</u>H$_3$). ESI-ICR (+): solvent ~ 0.01% DMSO:methanol, *m*/*z* of $[M+H]^+$ calculated for $C_{42}H_{49}N_6O_2$, 669.39225 found 669.39217.

Preparation of compound MC002

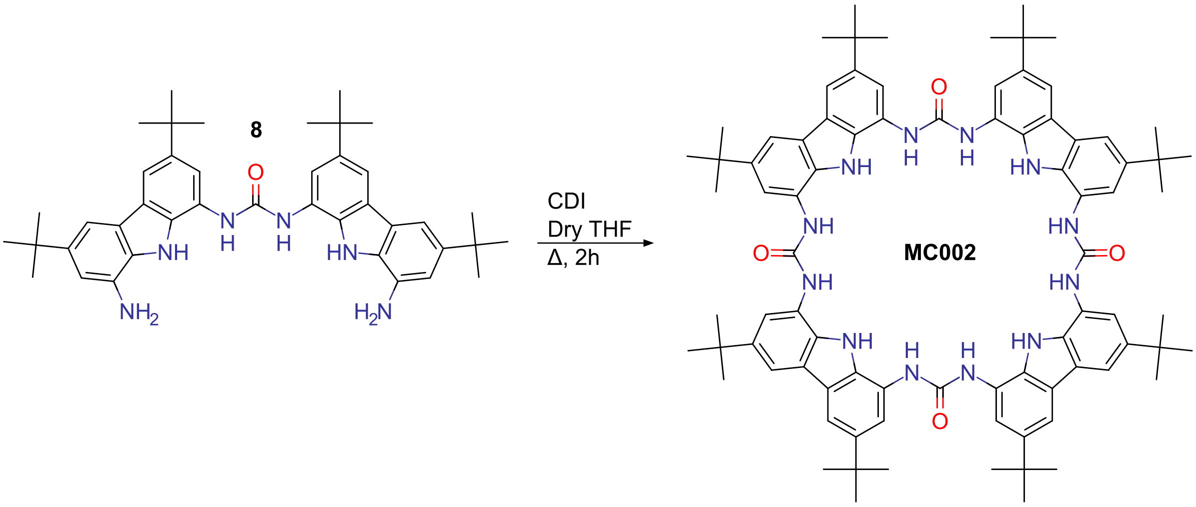

Compound **8** (0.10 g, 0.15 mmol), was dissolved in dry THF (25 mL), then CDI (0.04 g, 0.24 mmol) was added. The reaction mixture was stirred at reflux temperature for 6 h. After disappearance of the starting material (monitored by TLC), the reaction mixture was cooled and concentrated under reduced pressure. The obtained solid was quenched in 50 mL H_2O and extracted with 50 mL ethyl acetate. The ethyl acetate layer was concentrated under reduced pressure. The obtained solid was treated with chloroform (20 mL), filtered and washed with chloroform (60 mL). The solid insoluble in chloroform was pure compound **MC002** (0.04 g, 0.03 mmol, 38.4%) isolated as off-white solid.

Data for **MC002**. ^{1}H NMR (700.1 MHz, DMSO-d$_6$, +25 °C) δ: 9.86 (bs, 4H, carb. N<u>H</u>), 9.21 (bs, 8H, amide N<u>H</u>), 8.01 (d, *J* = 1.18 Hz, 8H, C<u>H</u>-4), 7.16 (d, *J* = 1.18 Hz, 8H, C<u>H</u>-2), 1.44 (s, 72H, (*t-butyl*-C<u>H</u>$_3$). ^{13}C NMR (176.0 MHz, DMSO-d$_6$, +25 °C) δ: 153.7 (urea <u>C</u>O), 143.3 (<u>C</u>-3), 134.5 (<u>C</u>-6), 127.0 (<u>C</u>-5), 124.0 (<u>C</u>-1), 117.3 (<u>C</u>-2), 113.6 (<u>C</u>-4), 35.0 (*t-butyl*-<u>C</u>), 32.3 (*t-butyl*-<u>C</u>H$_3$). ESI-ICR (+): solvent ~ 0.01% DMSO:methanol, *m/z* of $[M+H]^+$ calculated for $C_{84}H_{101}N_{12}O_4$, 1341.80687 found 1341.80722.

Preparation of macrocyclic receptors MC003–MC010

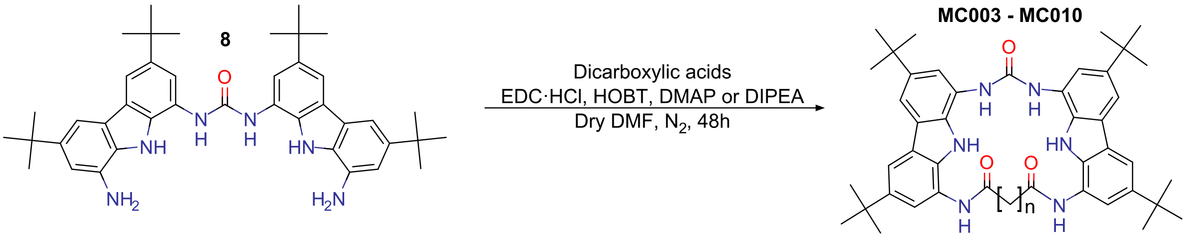

1,3-Bis(8-amino-3,6-di-*tert*-butyl-9*H*-carbazol-1-yl)urea (**8**) was dissolved in dry DMF alongside EDC·HCl, HOBT and, for receptors **MC003**–**MC006**, DMAP or, for receptors **MC007**–**MC010**, DIPEA. The corresponding dicarboxylic acid was added. The flask was charged with a magnetic stirring bar and closed with a septum, evacuated and backfilled with N_2 after which a N_2-filled balloon was connected through the septum. The mixture was stirred at room temperature. After disappearance of starting material (monitored by TLC) the reaction was quenched in water (50 mL). The formed precipitate was filtered and washed with water (50 mL). The crude product was purified by column chromatography eluting with MeOH in DCM to afford the macrocycles **MC003–MC010** as white to off-white crystalline powders in 29–87% yields. Please refer to Table S2 for exact reaction conditions for **MC003**–**MC014**.

Preparation of macrocyclic receptors MC011–MC014

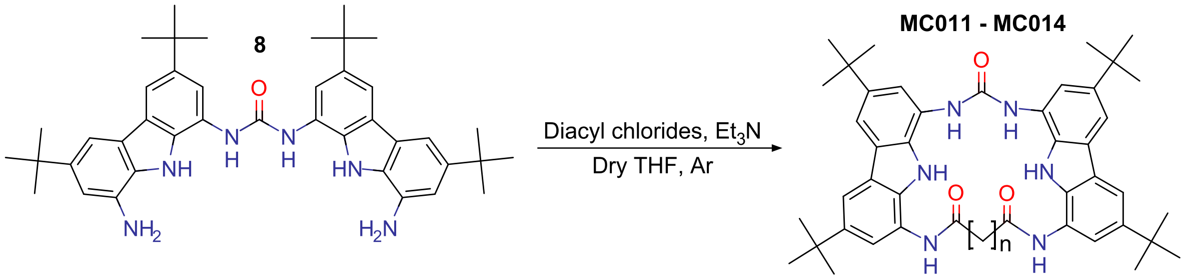

An oven-dried 100-mL round-bottomed flask was charged with a magnetic stirring bar and 1,3-bis(8-amino-3,6-di-*tert*-butyl-9*H*-carbazol-1-yl)urea (**8**) hydrochloride salt (100 mg, 0.139 mmol). The flask was closed with a septum, evacuated and backfilled with argon (3 times) after which an argon-filled balloon was connected through the septum. With a syringe, 36 mL of dry THF and 78 µL of triethylamine (0.56 mmol) were added and the contents were stirred. To the resulting suspension, the diacyl chloride solution in dry THF (0.139 mmol in 4 mL THF) was added dropwise during one hour. The mixture was stirred overnight at room temperature and then quenched by the addition of 0.1 mL of water. The solvent was removed by evaporation and the residue dissolved in 40 mL of DCM. The solution was washed with 20 mL of 10% aqueous K_2CO_3 and the phases were separated. The aqueous phase was extracted with DCM (3 × 10 mL) and the combined organic phases were dried on anhydrous Na_2SO_4, filtered, and the solvent evaporated. The residue was purified by column chromatography eluting with DCM-MeOH to yield the desired macrocycles as white to off-white crystals in 25–44% yields. Please refer to Table S2 for exact reaction conditions for **MC011**–**MC014**.

Table S2: Reaction conditions for **MC003–MC014**.

Compound	**8**	EDC·HCl	HOBT	DMAP/DIPEA[a]	Dicarboxylic acid
**MC003**	80 mg; 0.12 mmol	59 mg; 0.31 mmol	42 mg; 0.31 mmol	39 mg; 0.31 mmol	16 mg; 0.12 mmol
**MC004**	60 mg; 0.09 mmol	53 mg; 0.28 mmol	37 mg; 0.28 mmol	23 mg; 0.19 mmol	14 mg; 0.09 mmol
**MC005**	80 mg; 0.12 mmol	71 mg; 0.37 mmol	50 mg; 0.37 mmol	45 mg; 0.37 mmol	20 mg; 0.12 mmol
**MC006**	85 mg; 0.13 mmol	76 mg; 0.40 mmol	53 mg; 0.40 mmol	48 mg; 0.40 mmol	23 mg; 0.13 mmol
**MC007**	91 mg; 0.14 mmol	81 mg; 0.42 mmol	57 mg; 0.42 mmol	55 mg; 0.42 mmol	26 mg; 0.14 mmol
**MC008**	100 mg; 0.16 mmol	89 mg; 0.47 mmol	63 mg; 0.47 mmol	63 mg; 0.47 mmol	31 mg; 0.16 mmol
**MC009**	110 mg; 0.17 mmol	98 mg; 0.51 mmol	69 mg; 0.51 mmol	66 mg; 0.51 mmol	37 mg; 0.17 mmol
**MC010**	100 mg; 0.16 mmol	91 mg; 0.48 mmol	54 mg; 0.40 mmol	61 mg; 0.48 mmol	36 mg; 0.16 mmol

Compound	Solvent[b]	Reaction time[c]	Column eluent	Yield
**MC003**	5 ml DMF	60 h	1-2% MeOH/DCM[e]	70 mg; 0.09 mmol; 76%
**MC004**	5 ml DMF	45 h	1-2% MeOH/DCM[e]	50 mg; 0.07 mmol; 71%
**MC005**	5 ml DMF	45 h	1-2% MeOH/DCM[e]	80 mg; 0.10 mmol; 87%
**MC006**	5 ml DMF	45 h	1-2% MeOH/DCM[e]	50 mg; 0.06 mmol; 48%
**MC007**	5 ml DMF	40 h	1-2% MeOH/DCM[e]	60 mg; 0.08 mmol; 53%
**MC008**	5 ml DMF	40 h	1-2% MeOH/DCM[e]	75 mg; 0.09 mmol; 64%
**MC009**	5 ml DMF	40 h	1-2% MeOH/DCM[e]	40 mg; 0.05 mmol; 29%
**MC010**	5 ml DMF	40 h	1-2% MeOH/DCM[e]	50 mg; 0.06 mmol; 37%
**MC011**[d]	40 ml THF	overnight	1% MeOH/DCM[f]	52 mg; 0.06 mmol; 44%
**MC012**[d]	40 ml THF	overnight	1% MeOH/DCM[f]	47 mg; 0.05 mmol; 39%
**MC013**[d]	40 ml THF	overnight	1% MeOH/DCM[f]	48 mg; 0,05 mmol; 39%
**MC014**[d]	40 ml THF	overnight	1% MeOH/DCM[f]	31 mg; 0.04 mmol; 25%

[a] - DMAP for **MC001–MC010**, DIPEA for **MC011–MC014**; [b] - all solvents dried before use; [c] - all reactions at room temperature; [d] – amount of **8** for reaction was 100 mg; 0.14 mmol; [e] - gradient elution; [f] - isocratic elution.

Data for **MC003**. ^{1}H NMR (700.1 MHz, DMSO-d_6, +25 °C) δ: 10.24 (bs, 2H, carb. NH), 9.96 (bs, 2H, amide NH), 8.95 (bs, 2H, urea NH), 7.99 (d, J = 1.4 Hz, 2H, CH-5), 7.97 (d, J = 1.4 Hz, 2H, CH-4), 7.91 (d, J = 1.4 Hz, 2H, CH-7), 7.15, (d, J = 1.4 Hz, 2H, CH-2), 2.52-2.49 (m, 4H, -CO-CH_2), 2.12-2.11 (m, 2H, -CO-CH_2-CH_2), 1.43 (s, 18H, *t-butyl*-CH_{3a} or CH_{3b}), 1.40 (s, 18H, *t-butyl*- CH_{3a} or CH_{3b}). ^{13}C NMR (176.0 MHz, DMSO-d_6, +25 °C) δ: 172.1 (amide CO), 154.7 (urea CO), 142.1 (C-3 or C-6), 142.8 (C-3 or C-6), 132.1 (C-12), 131.1 (C-9), 125.2 (C-11), 124.4 (C-10), 123.1 (C-1), 122.9 (C-8), 117.8 (C-7), 117.1 (C-2), 113.0 (C-4 & C-5), 35.3 (CO-CH_2), 34.9 (*t-butyl*-C_a or C_b), 34.8 (*t-butyl*-C_a or C_b), 32.3 (*t-butyl*-CH_{3a} or CH_{3b}), 32.3 (*t-butyl*-CH_{3a} or CH_{3b}), 21.5 (-CO-CH_2-CH_2). ESI FT-ICR (+): solvent methanol (0.1% HCOOH), *m*/*z* of $[M+H]^+$ calculated for $C_{46}H_{57}N_6O_3$, 741.44867 found 741.44826.

Data for **MC004**. ^{1}H NMR (700.1 MHz, DMSO-d_6, +25 °C) δ: 10.17 (bs, 2H, amide NH), 9.35 (bs, 2H, carb NH), 8.90 (bs, 2H, urea NH), 8.01 (d, J = 1.4 Hz, 2H, CH-5), 7.90 (d, J = 1.4 Hz, 2H, CH-4), 7.84 (d, J = 1.4 Hz, 2H, CH-2), 7.40 (d, J = 1.4 Hz, 2H, CH-7), 2.58-2.52 (m, 4H, -CO-CH_2), 1.93-1.91 (m, 4H, -CO-CH_2-CH_2), 1.45 (s, 18H, *t-butyl*-CH_{3a} or CH_{3b}), 1.42 (s, 18H, *t-butyl*-CH_{3a} or CH_{3b}). ^{13}C NMR (176.0 MHz, DMSO-d_6, +25 °C) δ: 171.6 (amide CO), 153.6 (urea CO), 142.6 (C-3 or C-6), 142.4 (C-3 or C-6), 131.8 (C-9), 130.4 (C-12), 125.0 (C-11), 124.1 (C-10), 123.3 (C-1), 122.4 (C-8), 117.1 (C-7), 115.4 (C-2), 113.3 (C-5), 111.4 (C-4), 35.3 (CO-CH_2), 34.6 (*t-butyl*-C_a or C_b), 34.4 (*t-butyl*-C_a or C_b), 32.0 (*t-butyl*-CH_{3a} or CH_{3b}), 31.8 (*t-butyl*-CH_{3a} or CH_{3b}), 24.4 (-CO-CH_2-CH_2). ESI FT-ICR (+): solvent methanol (0.1% HCOOH), *m*/*z* of $[M+H]^+$ calculated for $C_{47}H_{59}N_6O_3$, 755.46432 found 755.46429.

Data for **MC005**. ^{1}H NMR (700.1 MHz, DMSO-d_6, +25 °C) δ: 10.07 (bs, 2H, amide NH), 9.93 (bs, 2H, carb. NH), 8.90 (bs, 2H, urea NH), 7.98 (d, J = 1.4 Hz, 2H, CH-5), 7.90 (d, J = 1.4 Hz, 2H, CH-4), 7.91 (d, J = 1.4 Hz, 2H, CH-2), 7.51 (d, J = 1.4 Hz, 2H, CH-7), 2.56-2.52 (m, 4H, -CO-CH_2-CH_2), 1.83-1.79 (m, 4H, (CO-CH_2-CH_2), 1.60-1.56 (m, 2H, -CO-CH_2-CH_2-CH_2), 1.44 (s, 18H, *t-butyl*-CH_{3a} or CH_{3b}), 1.40 (s, 18H, *t-butyl*-CH_{3a} or CH_{3b}). ^{13}C NMR (176.0 MHz,

DMSO-d_6, +25 °C) δ: 172.1 (amide $\underline{C}O$), 154.0 (urea $\underline{C}O$), 142.6 ($\underline{C}$-3 or $\underline{C}$-6), 142.3 ($\underline{C}$-3 or $\underline{C}$-6), 131.6 ($\underline{C}$-9), 130.9 ($\underline{C}$-12), 125.4 ($\underline{C}$-11), 124.5 ($\underline{C}$-10), 123.9 ($\underline{C}$-1), 123.1 ($\underline{C}$-8), 117.1 ($\underline{C}$-7), 115.6 ($\underline{C}$-2), 113.3 ($\underline{C}$-5), 111.8 ($\underline{C}$-4), 35.6 (-CO-$\underline{C}H_2$-CH_2-CH_2), 35.0 (*t-butyl*-$\underline{C}_a$ or $\underline{C}_b$), 34.9 (*t-butyl*-$\underline{C}_a$ or $\underline{C}_b$), 32.4 (*t-butyl*-$\underline{C}H_{3a}$ or $\underline{C}H_{3b}$), 32.3 (*t-butyl*-$\underline{C}H_{3a}$ or $\underline{C}H_{3b}$), 28.4 (-CO-CH_2-CH_2-$\underline{C}H_2$), 24.8 (-CO-CH_2-$\underline{C}H_2$-CH_2). ESI FT-ICR (+): solvent methanol (0.1% HCOOC), *m/z* of $[M+H]^+$ calculated for $C_{48}H_{61}N_6O_3$, 769.47997 found 769.48020.

Data for **MC006**. ^{1}H NMR (700.1 MHz, DMSO-d_6, +25 °C) δ: 10.11 (bs, 2H, amide N$\underline{H}$), 9.66 (bs, 2H, carb. N$\underline{H}$), 8.96 (bs, 2H, urea N$\underline{H}$), 8.34 (d, J = 1.4 Hz, 2H, C$\underline{H}$-2), 8.00 (d, J = 1.4 Hz, 2H, C$\underline{H}$-5), 7.85 (d, J = 1.4 Hz, 2H, C$\underline{H}$-4), 7.37, (d, J = 1.4 Hz, 2H, C$\underline{H}$-7), 2.51-2.49 (m, 4H, -CO-$C\underline{H}_2$-CH_2), 1.91-1.89 (m, 4H, (-CO-CH_2-$C\underline{H}_2$-CH_2), 1.55-1.53 (m, 4H, -CO-CH_2-CH_2-$C\underline{H}_2$), 1.45 (s, 18H, *t-butyl*-$C\underline{H}_{3a}$ or $C\underline{H}_{3b}$), 1.41 (s, 18H, *t-butyl*-$C\underline{H}_{3a}$ or $C\underline{H}_{3b}$). ^{13}C NMR (176.0 MHz, DMSO-d_6, +25 °C) δ: 172.2 (amide $\underline{C}O$), 153.2 (urea $\underline{C}O$), 142.7 ($\underline{C}$-3 or $\underline{C}$-6), 142.4 ($\underline{C}$-3 or $\underline{C}$-6), 131.7 ($\underline{C}$-9), 129.1 ($\underline{C}$-12), 125.2 ($\underline{C}$-8), 124.5 ($\underline{C}$-10), 124.5 ($\underline{C}$-11), 122.9 ($\underline{C}$-1), 117.7 ($\underline{C}$-7), 113.7 ($\underline{C}$-2), 113.2 ($\underline{C}$-5), 110.5 ($\underline{C}$-4), 36.6 (-CO-$\underline{C}H_2$-CH_2-CH_2), 35.1 (*t-butyl*-$\underline{C}_a$ or $\underline{C}_b$), 34.8 (*t-butyl*-$\underline{C}_a$ or $\underline{C}_b$), 32.4 (*t-butyl*-$\underline{C}H_{3a}$ or $\underline{C}H_{3b}$), 32.3 (*t-butyl*-$\underline{C}H_{3a}$ or $\underline{C}H_{3b}$), 28.4 (-CO-CH_2-$\underline{C}H_2$-CH_2), 24.8 (-CO-CH_2-CH_2-$\underline{C}H_2$). ESI FT-ICR (-): solvent ~ 0.01% DMSO:isopropanol, *m/z* of $[M+H]^-$ calculated for $C_{49}H_{63}N_6O_3$, 783.4956 found 783.4956.

Data for **MC007**. ^{1}H NMR (700.1 MHz, DMSO-d_6, +25 °C) δ: 10.00 (bs, 2H, amide N$\underline{H}$), 9.99 (bs, 2H, carb. N$\underline{H}$), 8.82 (bs, 2H, urea N$\underline{H}$), 8.10 (d, J = 1.4 Hz, 2H, C$\underline{H}$-2), 7.97 (d, J = 1.4 Hz, 2H, C$\underline{H}$-5), 7.89 (d, J = 1.4 Hz, 2H, C$\underline{H}$-4), 7.60 (d, J = 1.4 Hz, 2H, C$\underline{H}$-7), 2.51-2.49 (m, 4H, -CO-$C\underline{H}_2$-CH_2), 1.80-1.60 (m, 4H, -CO-CH_2-$C\underline{H}_2$), 1.55-1.46 (m, 6H, CO-CH_2-CH_2-$(C\underline{H}_2)_2$), 1.44 (s, 18H, *t-butyl*-$C\underline{H}_{3a}$ or $C\underline{H}_{3b}$), 1.41 (s, 18H, *t-butyl*-$C\underline{H}_{3a}$ or $C\underline{H}_{3b}$). ^{13}C NMR (176.0 MHz, DMSO-d_6, +25 °C) δ: 171.9 (amide $\underline{C}O$), 153.7 (urea $\underline{C}O$), 142.5 ($\underline{C}$-3 or $\underline{C}$-6), 142.3 ($\underline{C}$-3 or $\underline{C}$-6), 131.4 ($\underline{C}$-9), 130.5 ($\underline{C}$-12), 125.2 ($\underline{C}$-11), 124.5 ($\underline{C}$-10), 124.1 ($\underline{C}$-1), 123.0 ($\underline{C}$-8), 117.1 ($\underline{C}$-7), 115.5 ($\underline{C}$-2), 113.2 ($\underline{C}$-5), 111.5 ($\underline{C}$-4), 36.4 (-CO-$\underline{C}H_2$-CH_2-CH_2), 35.0 (*t-butyl*-$\underline{C}_a$ or

$\underline{C}_b$), 34.9 (*t-butyl*-$\underline{C}_a$ or $\underline{C}_b$), 32.4 (*t-butyl*-$\underline{C}H_{3a}$ or $\underline{C}H_{3b}$), 32.2 (*t-butyl*-$\underline{C}H_{3a}$ or $\underline{C}H_{3b}$), 28.2 (-CO-CH_2-CH_2-CH_2-$\underline{C}H_2$), 28.1 (-CO-CH_2-$\underline{C}H_2$-CH_2-CH_2), 25.0 (-CO-CH_2-CH_2-$\underline{C}H_2$-CH_2). ESI FT-ICR (-): solvent ~ 0.01% DMSO:isopropanol, *m*/*z* of $[M+Na]^-$ calculated for $C_{50}H_{64}N_6NaO_3$, 819.4932 found 819.4932.

Data for **MC008**. ^{1}H NMR (700.1 MHz, DMSO-d_6, +25 °C) δ: 10.10 (bs, 2H, carb. N$\underline{H}$), 10.05 (bs, 2H, amide N$\underline{H}$), 8.87 (bs, 2H, urea N$\underline{H}$), 8.10 (d, *J* = 1.4 Hz, 2H, C$\underline{H}$-2), 7.96 (d, *J* = 1.4 Hz, 2H, C$\underline{H}$-5), 7.89 (d, *J* = 1.4 Hz, 2H, C$\underline{H}$-4), 7.70 (d, *J* = 1.4 Hz, 2H, C$\underline{H}$-7), 2.51-2.49 (m, 4H, -CO-$C\underline{H}_2$-CH_2), 1.81-1.70 (m, 4H, -CO-CH_2-$C\underline{H}_2$), 1.45-1.11 (m, 8H, -CO-CH_2-CH_2-$(C\underline{H}_2)_2$), 1.44 (s, 18H, *t-butyl*-$C\underline{H}_{3a}$ or $C\underline{H}_{3b}$), 1.41 (s, 18H, *t-butyl*-$C\underline{H}_{3a}$ or $C\underline{H}_{3b}$). ^{13}C NMR (176.0 MHz, DMSO-d_6, +25 °C) δ: 171.5 (amide $\underline{C}$O), 153.5 (urea $\underline{C}$O), 142.0 ($\underline{C}$-3 or $\underline{C}$-6), 141.8 ($\underline{C}$-3 or $\underline{C}$-6), 130.3 ($\underline{C}$-9), 130.2 ($\underline{C}$-12), 124.9 ($\underline{C}$-11), 124.1 ($\underline{C}$-10), 123.5 ($\underline{C}$-1), 122.8 ($\underline{C}$-8), 116.0 ($\underline{C}$-7), 115.8 ($\underline{C}$-2), 112.4 ($\underline{C}$-5), 111.1 ($\underline{C}$-4), 35.7 (-CO-$\underline{C}H_2$-CH_2-CH_2), 34.6 (*t-butyl*-$\underline{C}_a$ or $\underline{C}_b$), 34.4 (*t-butyl*-C_a or $\underline{C}_b$), 31.9 (*t-butyl*-$\underline{C}H_{3a}$ or $\underline{C}H_{3b}$), 31.8 (*t-butyl*-$\underline{C}H_{3a}$ or $\underline{C}H_{3b}$), 27.9 (-CO-CH_2-CH_2-CH_2-$\underline{C}H_2$), 27.4 (-CO-CH_2-$\underline{C}H_2$-CH_2-CH_2), 24.3 (-CO-CH_2-CH_2-$\underline{C}H_2$-CH_2). ESI FT-ICR (-): solvent ~ 0.01% DMSO:isopropanol, *m*/*z* of $[M+Na]^-$ calculated for $C_{51}H_{66}N_6NaO_3$, 833.5088 found 833.5061.

Data for **MC009**. ^{1}H NMR (700.1 MHz, DMSO-d_6, +25 °C) δ: 10.11 (bs, 2H, carb. N$\underline{H}$), 9.99 (bs, 2H, amide N$\underline{H}$), 8.81 (bs, 2H, urea N$\underline{H}$), 7.95 (d, *J* = 1.4 Hz, 2H, C$\underline{H}$-5), 7.92 (d, *J* = 1.4 Hz, 2H, C$\underline{H}$-4), 7.88 (d, *J* = 1.4 Hz, 2H, C$\underline{H}$-2), 7.77 (d, *J* = 1.4 Hz, 2H, C$\underline{H}$-7), 2.51-2.49 (m, 4H, -CO-$C\underline{H}_2$-CH_2), 1.72-1.69 (m, 4H, -CO-CH_2-$C\underline{H}_2$), 1.45-1.11 (m, 10H, -CO-CH_2-CH_2-$(C\underline{H}_2)_3$), 1.43 (s, 18H, *t-butyl*-$C\underline{H}_{3a}$ or $C\underline{H}_{3b}$), 1.40 (s, 18H, *t-butyl*-$C\underline{H}_{3a}$ or $C\underline{H}_{3b}$). ^{13}C NMR (176.0 MHz, DMSO-d_6, +25 °C) δ: 171.9 (amide $\underline{C}$O), 154.4 (urea $\underline{C}$O), 142.4 ($\underline{C}$-3 or $\underline{C}$-6), 142.2 ($\underline{C}$-3 or $\underline{C}$-6), 131.4 ($\underline{C}$-9), 130.6 ($\underline{C}$-12), 125.1 ($\underline{C}$-11), 124.6 ($\underline{C}$-10), 123.8 ($\underline{C}$-1), 123.3 ($\underline{C}$-8), 117.1 ($\underline{C}$-2), 116.4 ($\underline{C}$-7), 112.8 ($\underline{C}$-5), 112.0 ($\underline{C}$-4), 36.6 (-CO-$\underline{C}H_2$-CH_2-CH_2), 35.0 (*t-butyl*-$\underline{C}_a$ or $\underline{C}_b$), 34.9 (*t-butyl*-C_a or $\underline{C}_b$), 32.4 (*t-butyl*-$\underline{C}H_{3a}$ or $\underline{C}H_{3b}$), 32.3 (*t-butyl*-$\underline{C}H_{3a}$ or

$\underline{C}H_{3b}$), 28.5 (-CO-CH_2-$\underline{C}H_2$-CH_2-CH_2-CH_2), 28.3 (-CO-CH_2-CH_2-$\underline{C}H_2$-CH_2-CH_2), 28.0 (-CO-CH_2-CH_2-CH_2-CH_2-$\underline{C}H_2$), 25.2 (-CO-CH_2-CH_2-CH_2-$\underline{C}H_2$-CH_2). ESI FT-ICR (-): solvent ~ 0.01% DMSO:isopropanol, *m*/*z* of $[M+H]^-$ calculated for $C_{52}H_{69}N_6O_3$, 825.5425 found 825.5426.

Data for **MC010**. ^{1}H NMR (700.1 MHz, DMSO-*d*$_6$, +25 °C) δ: 10.10 (bs, 2H, carb. N$\underline{H}$), 10.0 (bs, 2H, amide N$\underline{H}$), 8.82 (bs, 2H, urea N$\underline{H}$), 7.95 (d, *J* = 1.4 Hz, 2H, C$\underline{H}$-5), 7.92 (d, *J* = 1.4 Hz, 2H, C$\underline{H}$-4), 7.83 (d, *J* = 1.4 Hz, 2H, C$\underline{H}$-2), 7.78 (d, *J* = 1.4 Hz, 2H, C$\underline{H}$-7), 2.45-2.49 (m, 4H, -CO-C$\underline{H}_2$-CH_2), 1.81-1.70 (m, 4H, -CO-CH_2-C$\underline{H}_2$), 1.42 (s, 18H, *t-butyl*-C$\underline{H}_{3a}$ or C$\underline{H}_{3b}$), 1.41 (s, 18H, *t-butyl*-C$\underline{H}_{3a}$ or C$\underline{H}_{3b}$), 1.42-1.11 (m, 12H, -CO-CH_2-CH_2-(C$\underline{H}_2$)$_3$). ^{13}C NMR (176.0 MHz, DMSO-*d*$_6$, +25 °C) δ: 171.9 (amide $\underline{C}$O), 154.5 (urea $\underline{C}$O), 142.3 ($\underline{C}$-3 or $\underline{C}$-6), 142.2 ($\underline{C}$-3 or $\underline{C}$-6), 131.5 ($\underline{C}$-9), 130.5 ($\underline{C}$-12), 125.1 ($\underline{C}$-11), 124.6 ($\underline{C}$-10), 123.7 ($\underline{C}$-1), 123.3 ($\underline{C}$-8), 117.4 ($\underline{C}$-2), 116.2 ($\underline{C}$-7), 112.8 ($\underline{C}$-5), 112.1 ($\underline{C}$-4), 36.4 (-CO-$\underline{C}H_2$-CH_2-CH_2), 35.0 (*t-butyl*-$\underline{C}_a$ or $\underline{C}_b$), 34.9 (*t-butyl*-C_a or $\underline{C}_b$), 32.4 (*t-butyl*-$\underline{C}H_{3a}$ or $\underline{C}H_{3b}$), 32.3 (*t-butyl*-$\underline{C}H_{3a}$ or $\underline{C}H_{3b}$), 28.5 (-CO-CH_2-$\underline{C}H_2$-CH_2-CH_2-CH_2), 28.4 (-CO-CH_2-CH_2-$\underline{C}H_2$-CH_2-CH_2), 28.3 (-CO-CH_2-CH_2-CH_2-CH_2-$\underline{C}H_2$), 25.4 (-CO-CH_2-CH_2-CH_2-$\underline{C}H_2$-CH_2). ESI FT-ICR (-): solvent ~ 0.01% DMSO:isopropanol, *m*/*z* of $[M+Na]^-$ calculated for $C_{53}H_{70}N_6NaO_3$, 861.54071 found 861.54028.

Data for **MC011**. ^{1}H NMR (700.1 MHz, DMSO-d$_6$, +25 °C) δ: 10.11 (s, 2H, carb. N$\underline{H}$), 9.98 (s, 2H, amide N$\underline{H}$), 8.82 (s, 2H, urea N$\underline{H}$), 7.95 (s, 2H, C$\underline{H}$-5), 7.93 (s, 2H, C$\underline{H}$-4), 7.86 (s, 2H, C$\underline{H}$-7), 7.73 (s, 2H, C$\underline{H}$-2), 2.46 (t, *J* = 6.6 Hz, 4H, NHCOC$\underline{H}_2$), 1.67 (quint, *J* = 6.6 Hz, 4H, NHCOCH$_2$C$\underline{H}_2$), 1.42 (s, 18H, *t*-Bu C$\underline{H}_3$), 1.41 (s, 18H, *t*-Bu C$\underline{H}_3$), 1.37 – 1.22 (m, 14H, aliphatic C$\underline{H}_2$). ^{13}C NMR (176.0 MHz, DMSO-d$_6$, +25 °C) δ: 171.35 (amide $\underline{C}$O), 154.16 (urea $\underline{C}$O), 141.80 ($\underline{C}$3/$\underline{C}$6), 141.65 ($\underline{C}$3/$\underline{C}$6), 131.33 ($\underline{C}$-12), 129.90 ($\underline{C}$-9), 124.54 ($\underline{C}$-10), 124.15 ($\underline{C}$-11), 123.12 ($\underline{C}$-1), 122.88 (C-$\underline{8}$), 117.21 ($\underline{C}$-2), 115.65 ($\underline{C}$-7), 112.19 ($\underline{C}$-5), 111.78 ($\underline{C}$-4), 35.88 (NHCO$\underline{C}H_2$), 34.49 (*t*-Bu $\underline{C}CH_3$), 34.45 (*t*-Bu $\underline{C}CH_3$), 31.88 (*t*-Bu C$\underline{C}H_3$), 31.84 (*t*-Bu C$\underline{C}H_3$),

28.2 – 28.1 (4x aliphatic $\underline{C}H_2$), 24.74 ($NHCOCH_2\underline{C}H_2$). ESI-ICR (+): solvent ~ 0.01% DMSO:methanol, *m*/*z* of $[M+H]^+$ calculated for $C_{54}H_{73}N_6O_3$, 853.56995 found 853.57361.

Data for **MC012**. ^{1}H NMR (700.1 MHz, DMSO-d_6, +25 °C) δ: 10.11 (s, 2H, carb. N$\underline{H}$), 9.98 (s, 2H, amide N$\underline{H}$), 8.82 (s, 2H, urea N$\underline{H}$), 7.95 (s, 2H, C$\underline{H}$-5), 7.92 (s, 2H, C$\underline{H}$-4), 7.81 (s, 2H, C$\underline{H}$-7), 7.73 (s, 2H, C$\underline{H}$-2), 2.45 (t, *J* = 7.3 Hz, 4H, $NHCOC\underline{H_2}$), 1.67 (quint, *J* = 7.3 Hz, 4H, $NHCOCH_2C\underline{H_2}$), 1.41 (s, 18H, *t*-Bu $C\underline{H_3}$), 1.40 (s, 18H, *t*-Bu $C\underline{H_3}$), 1.36 – 1.21 (m, 16H, aliphatic $C\underline{H_2}$). ^{13}C NMR (176.0 MHz, DMSO-d_6, +25 °C) δ: 171.40 (amide $\underline{C}O$), 154.06 (urea $\underline{C}O$), 141.80 ($\underline{C}3/\underline{C}6$), 141.64 ($\underline{C}3/\underline{C}6$), 131.19 ($\underline{C}$-12), 129.96 ($\underline{C}$-9), 124.56 ($\underline{C}$-10), 124.11 ($\underline{C}$-11), 123.10 ($\underline{C}$-1), 122.81 (C-$\underline{8}$), 116.98 ($\underline{C}$-2), 115.70 ($\underline{C}$-7), 112.27 ($\underline{C}$-5), 111.73 ($\underline{C}$-4), 36.03 ($NHCO\underline{C}H_2$), 34.48 (*t*-Bu $\underline{C}CH_3$), 34.44 (*t*-Bu $\underline{C}CH_3$), 31.87 (*t*-Bu $C\underline{C}H_3$), 31.83 (*t*-Bu $C\underline{C}H_3$), 28.2 – 28.0 (4x aliphatic $\underline{C}H_2$), 25.02 ($NHCOCH_2\underline{C}H_2$). ESI-ICR (+): solvent ~ 0.01% DMSO:methanol, *m*/*z* of $[M+H]^+$ calculated for $C_{55}H_{75}N_6O_3$, 867.58560 found 867.58949.

Data for **MC013**. ^{1}H NMR (700.1 MHz, DMSO-d_6, +25 °C) δ: 10.08 (s, 2H, carb. N$\underline{H}$), 9.97 (s, 2H, amide N$\underline{H}$), 8.82 (s, 2H, urea N$\underline{H}$), 7.95 (s, 2H, C$\underline{H}$-5), 7.93 (s, 2H, C$\underline{H}$-4), 7.82 (s, 2H, C$\underline{H}$-7), 7.68 (s, 2H, C$\underline{H}$-2), 2.47 – 2.40 (m, 4H, $NHCOC\underline{H_2}$), 1.70 – 1.60 (m, 4H, $NHCOCH_2C\underline{H_2}$), 1.40 (s, 36H, *t*-Bu $C\underline{H_3}$), 1.34 – 1.17 (m, 18H, aliphatic $C\underline{H_2}$). ^{13}C NMR (176.0 MHz, DMSO-d_6, +25 °C) δ: 171.36 (amide $\underline{C}O$), 153.97 (urea $\underline{C}O$), 141.79 ($\underline{C}3/\underline{C}6$), 141.63 ($\underline{C}3/\underline{C}6$), 131.26 ($\underline{C}$-12), 129.99 ($\underline{C}$-9), 124.53 ($\underline{C}$-10), 124.12 ($\underline{C}$-11), 123.06 ($\underline{C}$-1), 122.81 (C-$\underline{8}$), 116.96 ($\underline{C}$-2), 115.68 ($\underline{C}$-7), 112.28 ($\underline{C}$-5), 111.76 ($\underline{C}$-4), 35.94 ($NHCO\underline{C}H_2$), 34.48 (*t*-Bu $\underline{C}CH_3$), 34.46 (*t*-Bu $\underline{C}CH_3$), 31.88 (*t*-Bu $C\underline{C}H_3$), 31.84 (*t*-Bu $C\underline{C}H_3$), 28.3 – 28.1 (5x aliphatic $\underline{C}H_2$), 24.81 ($NHCOCH_2\underline{C}H_2$). ESI-ICR (+): solvent ~ 0.01% DMSO:methanol, *m*/*z* of $[M+H]^+$ calculated for $C_{56}H_{77}N_6O_3$, 881.60125 found 881.60474.

Data for **MC014**. ^{1}H NMR (700.1 MHz, DMSO-d_6, +25 °C) δ: 10.08 (s, 2H, carb. N$\underline{H}$), 9.96 (s, 2H, amide N$\underline{H}$), 8.83 (s, 2H, urea N$\underline{H}$), 7.95 (s, 2H, C$\underline{H}$-5), 7.92 (s, 2H, C$\underline{H}$-4), 7.79 (s, 2H,

CH-7), 7.68 (s, 2H, CH-2), 2.47 – 2.40 (m, 4H, NHCOCH_2), 1.70 – 1.60 (m, 4H, NHCOCH_2CH_2), 1.41 (s, 36H, *t*-Bu CH_3), 1.34 – 1.15 (m, 20H, aliphatic CH_2). ^{13}C NMR (176.0 MHz, DMSO-d_6, +25 °C) δ: 171.35 (amide CO), 153.89 (urea CO), 141.78 (C3/C6), 141.60 (C3/C6), 131.18 (C-12), 130.09 (C-9), 124.53 (C-10), 124.13 (C-11), 123.03 (C-1), 122.75 (C-8), 116.84 (C-2), 115.78 (C-7), 112.26 (C-5), 111.67 (C-4), 36.00 (NHCOCH_2), 34.45 (*t*-Bu CCH_3), 34.41 (*t*-Bu CCH_3), 31.84 (*t*-Bu CCH_3), 31.81 (*t*-Bu CCH_3), 28.3 – 28.0 (5x aliphatic CH_2), 24.95 (NHCOCH_2CH_2). ESI-ICR (+): solvent ~ 0.01% DMSO:methanol, *m*/*z* of $[M+H]^+$ calculated for $C_{57}H_{79}N_6O_3$, 895.61690 found 895.62076.

References

(1) Suu, A.; Jalukse, L.; Liigand, J.; Kruve, A.; Himmel, D.; Krossing, I.; Rosés, M.; Leito, I. *Anal. Chem.* **2015**, *87* (5), 2623–2630. doi:10.1021/ac504692m

(2) Peets, P.; Vahur, S.; Kruve, A.; Haljasorg, T.; Herodes, K.; Pagano, T.; Leito, I. *J. Cult. Herit.* **2019**. doi:10.1016/j.culher.2019.09.002

(3) Teearu, A.; Vahur, S.; Rodima, T.; Herodes, K.; Bonrath, W.; Netscher, T.; Tshepelevitsh, S.; Trummal, A.; Lõkov, M.; Leito, I. *J. Mass Spectrom.* **2017**, *52* (9), 603–617. doi:10.1002/jms.3943

(4) Kadam, S. A.; Haav, K.; Toom, L.; Haljasorg, T.; Leito, I. *J. Org. Chem.* **2014**, *79* (6), 2501–2513. doi:10.1021/jo4027963

(5) Kadam, S. A.; Martin, K.; Haav, K.; Toom, L.; Mayeux, C.; Pung, A.; Gale, P. A.; Hiscock, J. R.; Brooks, S. J.; Kirby, I. L.; Busschaert, N.; Leito, I. *Chem. - Eur. J.* **2015**, *21* (13), 5145–5160. doi:10.1002/chem.201405858

(6) Martin, K.; Nõges, J.; Haav, K.; Kadam, S. A.; Pung, A.; Leito, I. *Eur. J. Org. Chem.* **2017**, *2017* (35), 5231–5237. doi:10.1002/ejoc.201700931

(7) Liu, Y.; Nishiura, M.; Wang, Y.; Hou, Z. *J. Am. Chem. Soc.* **2006**, *128* (17), 5592–5593. doi:10.1021/ja058188f

(8) Sanchez, G.; Espinosa, A.; Curiel, D.; Tarraga, A.; Molina, P. *J. Org. Chem.* **2013**, *78* (19), 9725–9737. doi:10.1021/jo401430d

(9) Gibson, V. C.; Spitzmesser, S. K.; White, A. J. P.; Williams, D. J. *Dalton Trans.* **2003**, No. 13, 2718. doi:10.1039/b301902k

(10) Jung, H. S.; Yun, T.; Cho, Y.; Jeon, H. B. *Tetrahedron* **2016**, *72* (40), 5988–5993. doi:10.1016/j.tet.2016.07.021

^{1}H NMR spectrum (700.1 MHz) of compound **CZ016**

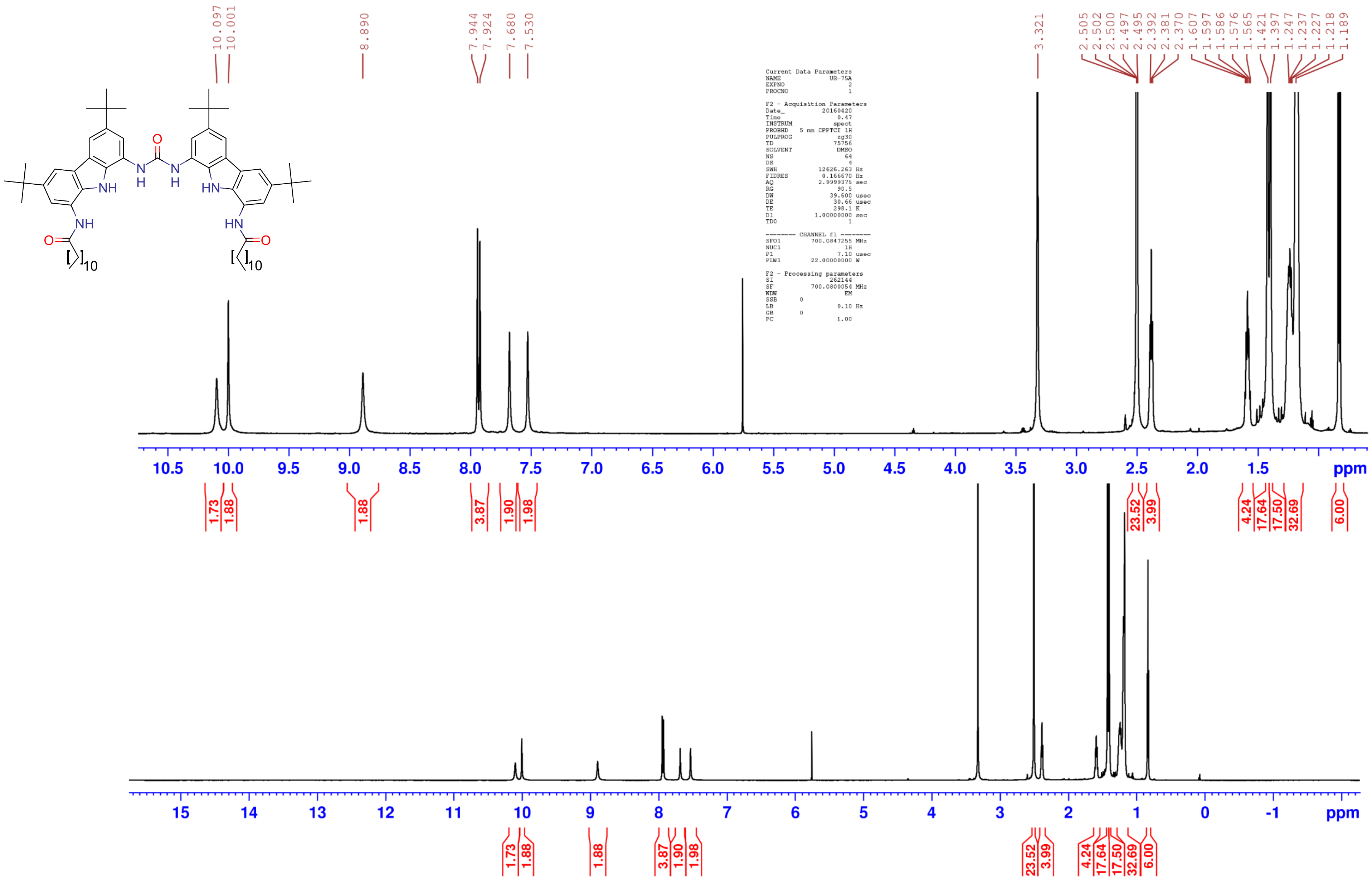

^{13}C NMR spectrum (700.1 MHz) of compound **CZ016**

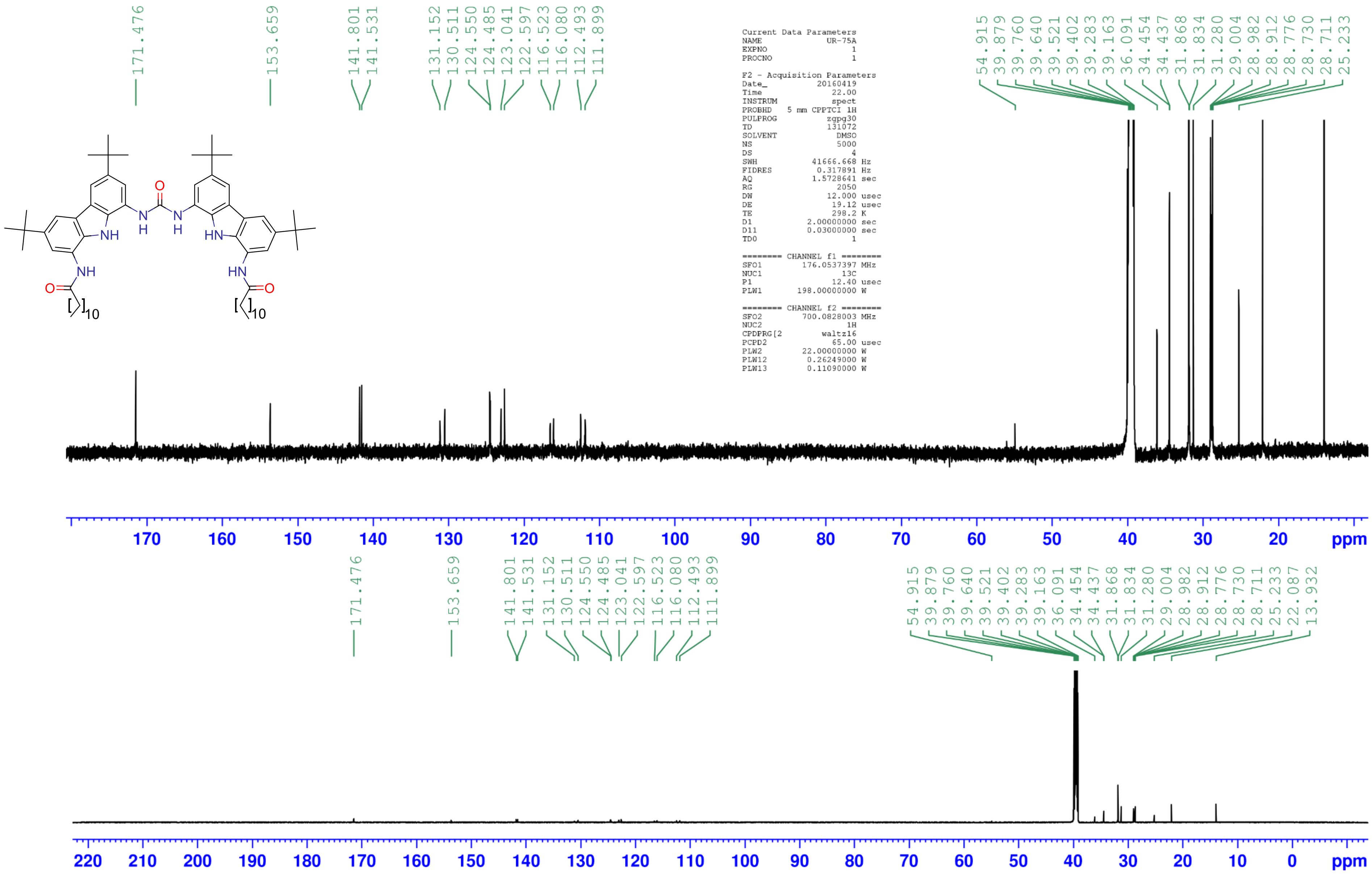

^{1}H-^{13}C HSQC spectrum (700.1 MHz) of compound **CZ016**

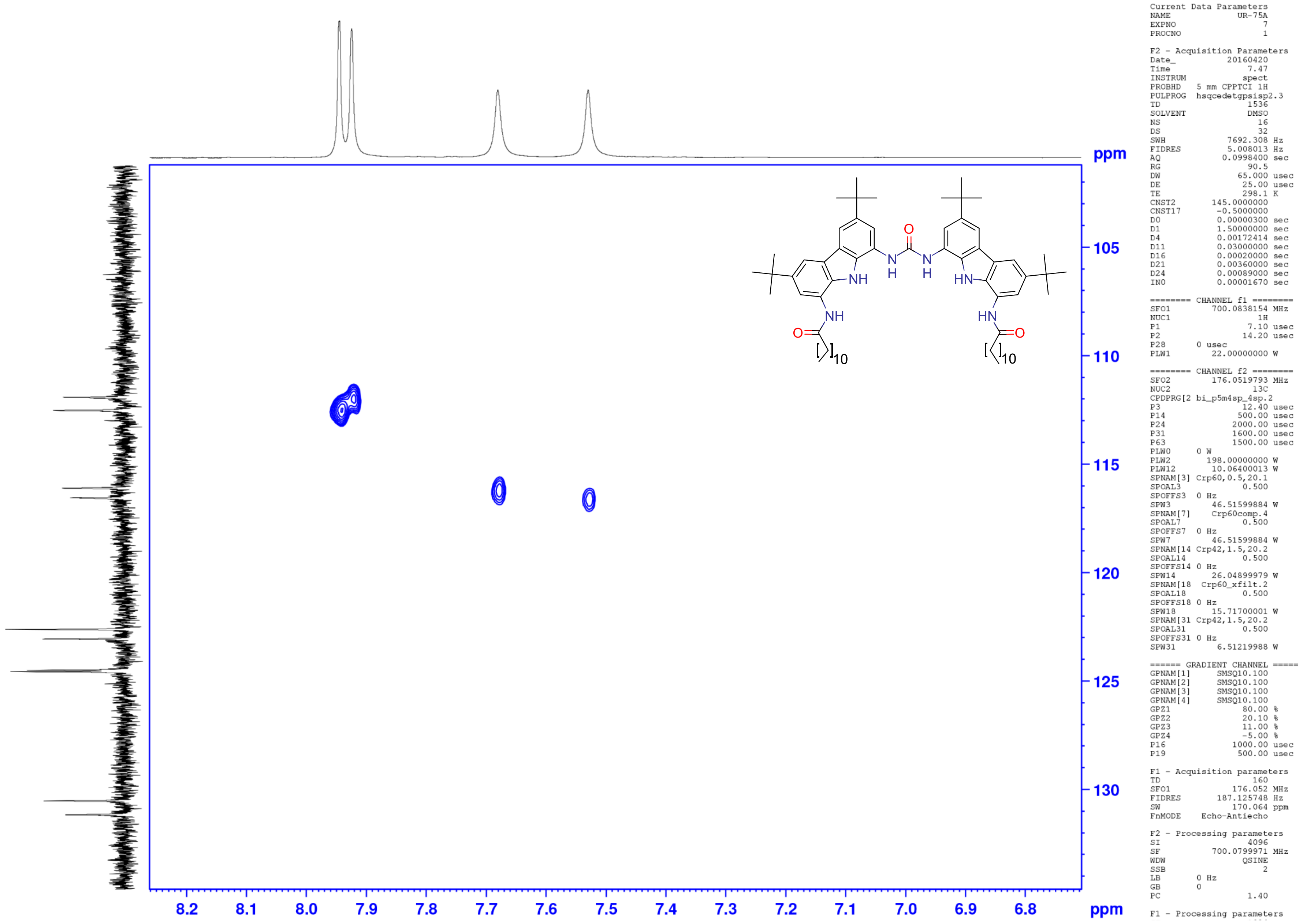

^{1}H-^{13}C HMBC spectrum (700.1 MHz) of compound **CZ016**

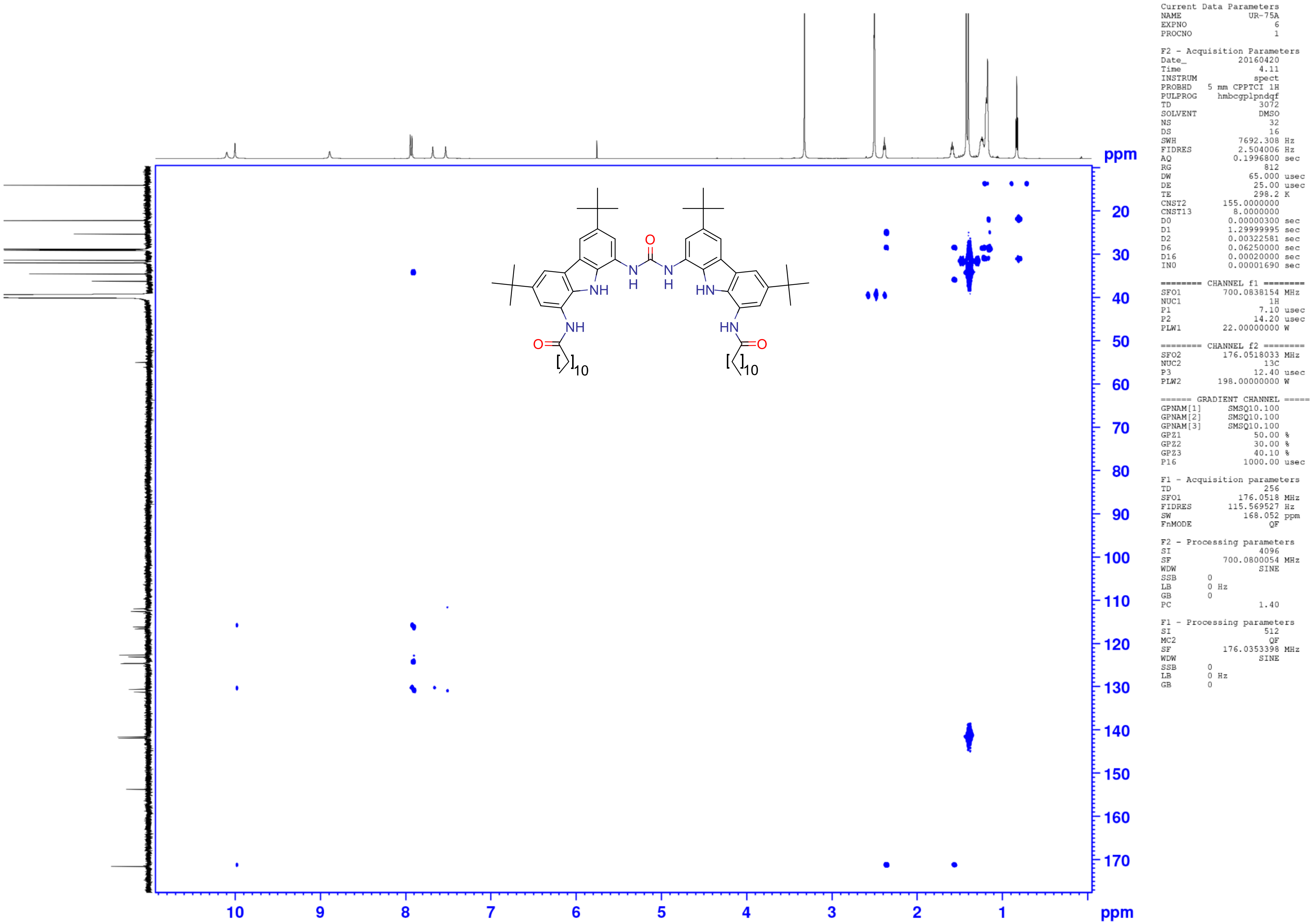

HRMS spectrum of compound **CZ016**

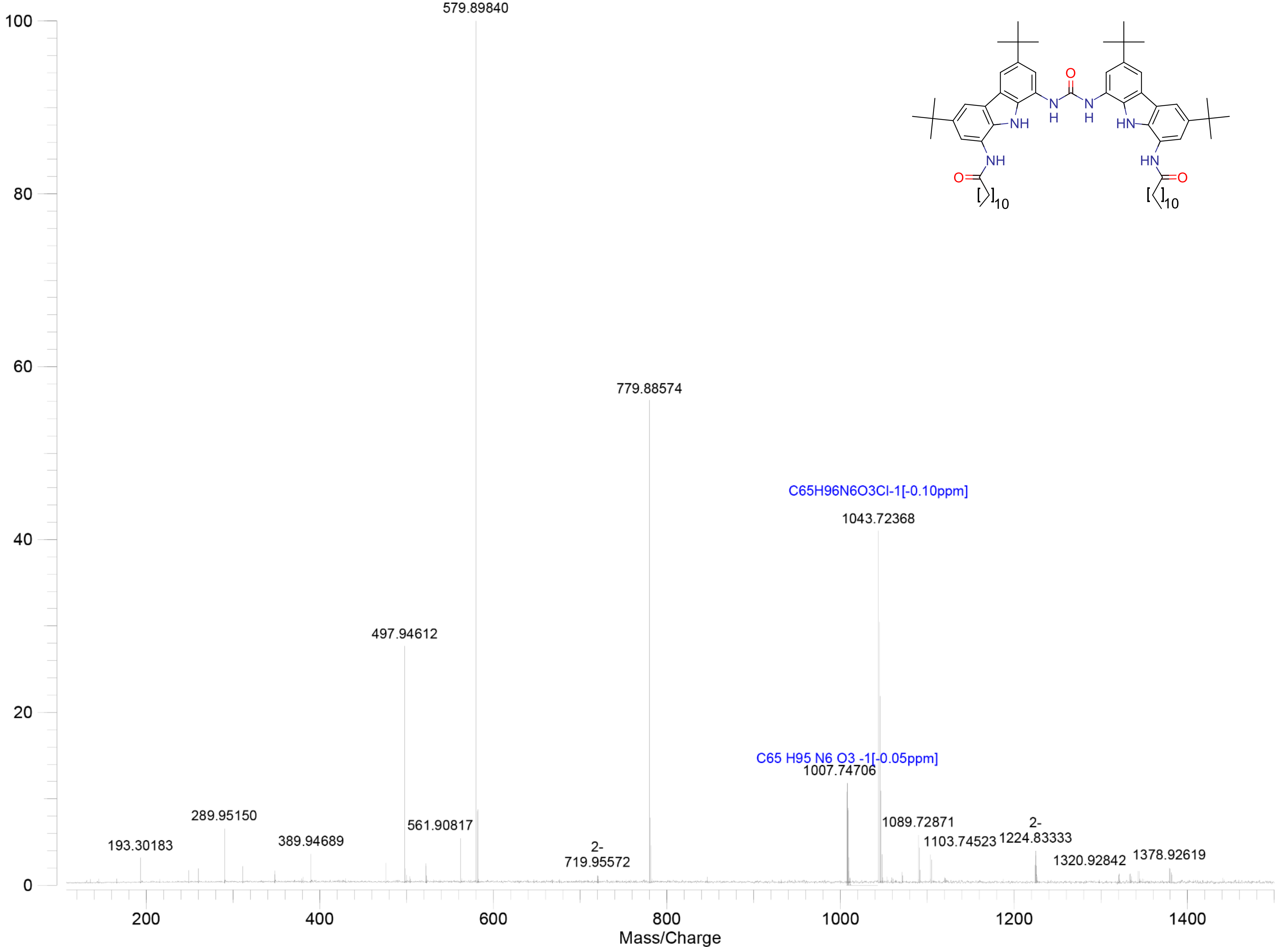

^{1}H NMR spectrum (700.1 MHz) of compound **MC001**

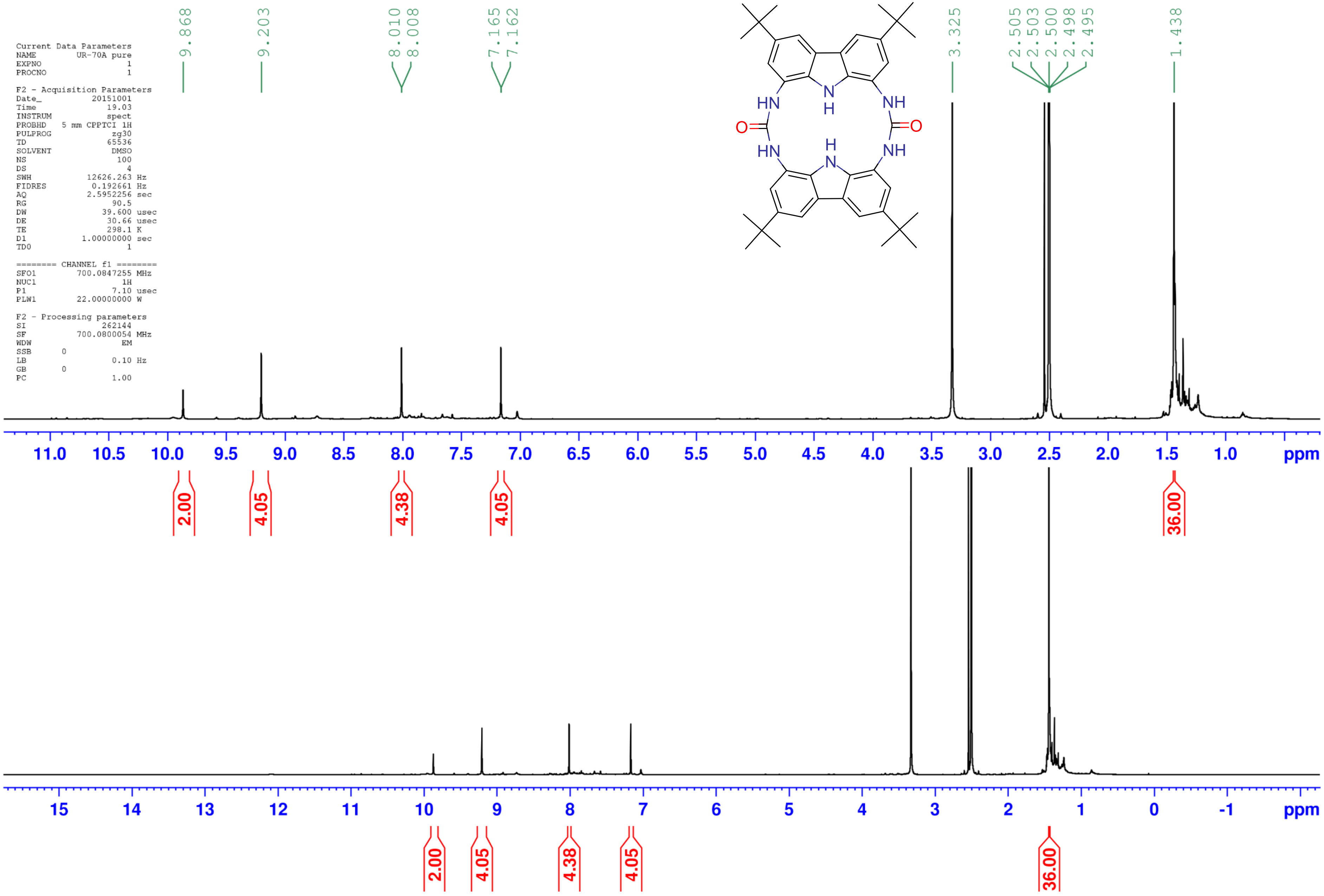

^{13}C NMR spectrum (700.1 MHz) of compound **MC001**

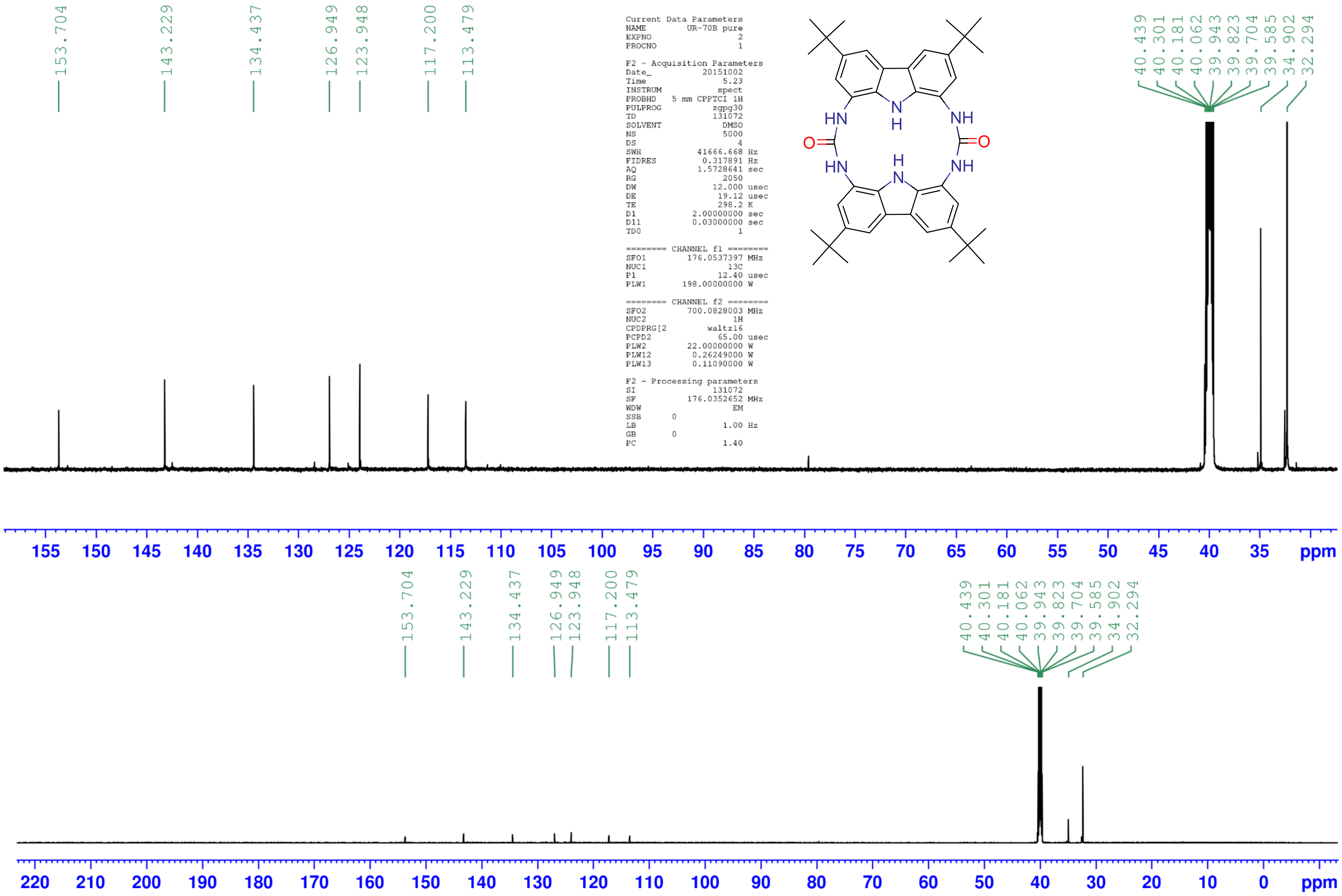

^{1}H-^{13}C HSQC spectrum (700.1 MHz) of compound **MC001**

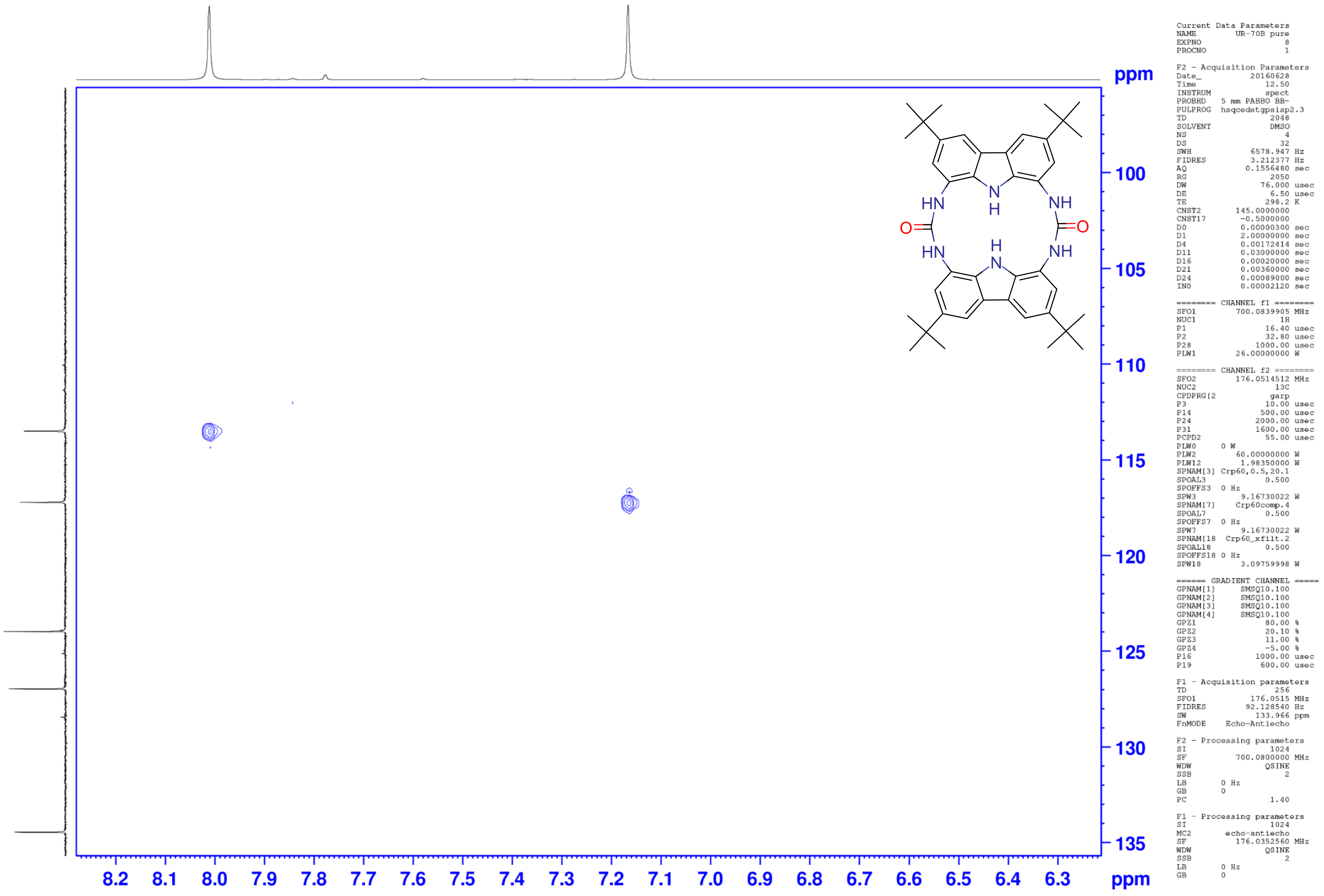

^{1}H-^{13}C HMBC spectrum (700.1 MHz) of compound **MC001**

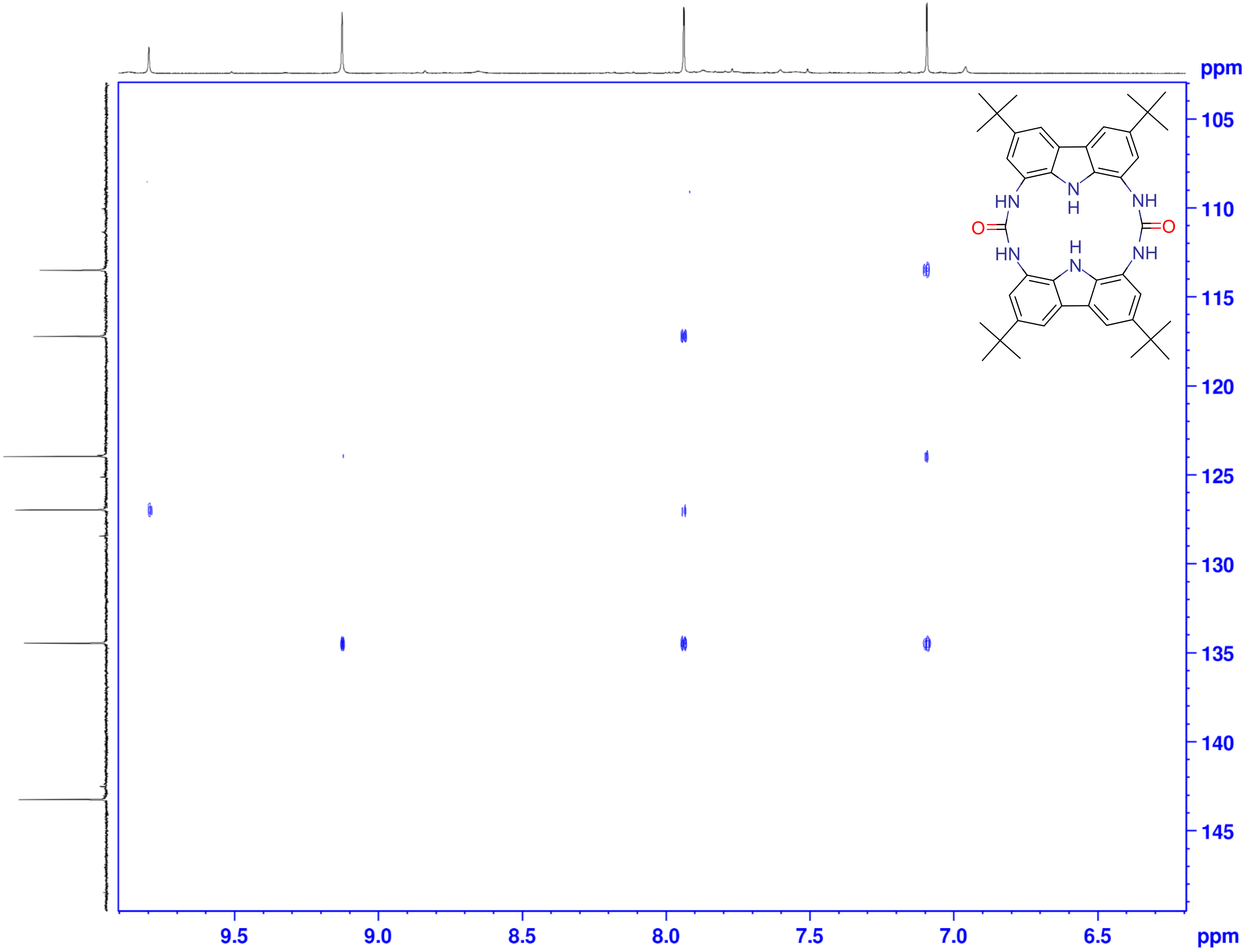


```
Current Data Parameters
NAME          UR-70B pure
EXPNO                   9
PROCNO                  1

F2 - Acquisition Parameters
Date_            20160628
Time                13.29
INSTRUM             spect
PROBHD    5 mm PABBO BB-
PULPROG     hmbcetgpl3nd
TD                   4096
SOLVENT              DMSO
NS                      4
DS                     16
SWH              7352.941 Hz
FIDRES           1.795152 Hz
AQ              0.2785280 sec
RG                   2050
DW                 68.000 usec
DE                   6.50 usec
TE                  298.1 K
CNST6         120.0000000
CNST7         170.0000000
CNST13          8.0000000
CNST30          0.5981181
D0             0.00000300 sec
D1             2.03276801 sec
D6             0.06250000 sec
D16            0.00020000 sec
IN0            0.00002120 sec

======== CHANNEL f1 ========
SFO1          700.0837080 MHz
NUC1                   1H
P1                  16.40 usec
P2                  32.80 usec
PLW1          26.00000000 W

======== CHANNEL f2 ========
SFO2          176.0514512 MHz
NUC2                  13C
P3                  10.00 usec
P24               2000.00 usec
PLW2          60.00000000 W
SPNAM[7]      Crp60comp.4
SPOAL7              0.500
SPOFFS7   0 Hz
SPW7           9.16730022 W

====== GRADIENT CHANNEL =====
GPNAM[1]       SMSQ10.100
GPNAM[3]       SMSQ10.100
GPNAM[4]       SMSQ10.100
GPNAM[5]       SMSQ10.100
GPNAM[6]       SMSQ10.100
GPZ1                80.00 %
GPZ3                14.00 %
GPZ4                -8.00 %
GPZ5                -4.00 %
GPZ6                -2.00 %
P16               1000.00 usec

F1 - Acquisition parameters
TD                    256
SFO1             176.0515 MHz
FIDRES          92.128540 Hz
SW                133.966 ppm
FnMODE       Echo-Antiecho

F2 - Processing parameters
SI                   2048
SF            700.0800557 MHz
WDW                  SINE
SSB                     4
LB        0 Hz
GB        0
PC                   1.40

F1 - Processing parameters
SI                   1024
MC2          echo-antiecho
SF            176.0352560 MHz
WDW                 QSINE
SSB                     2
LB        0 Hz
GB        0
```

HRMS spectrum of compound **MC001**

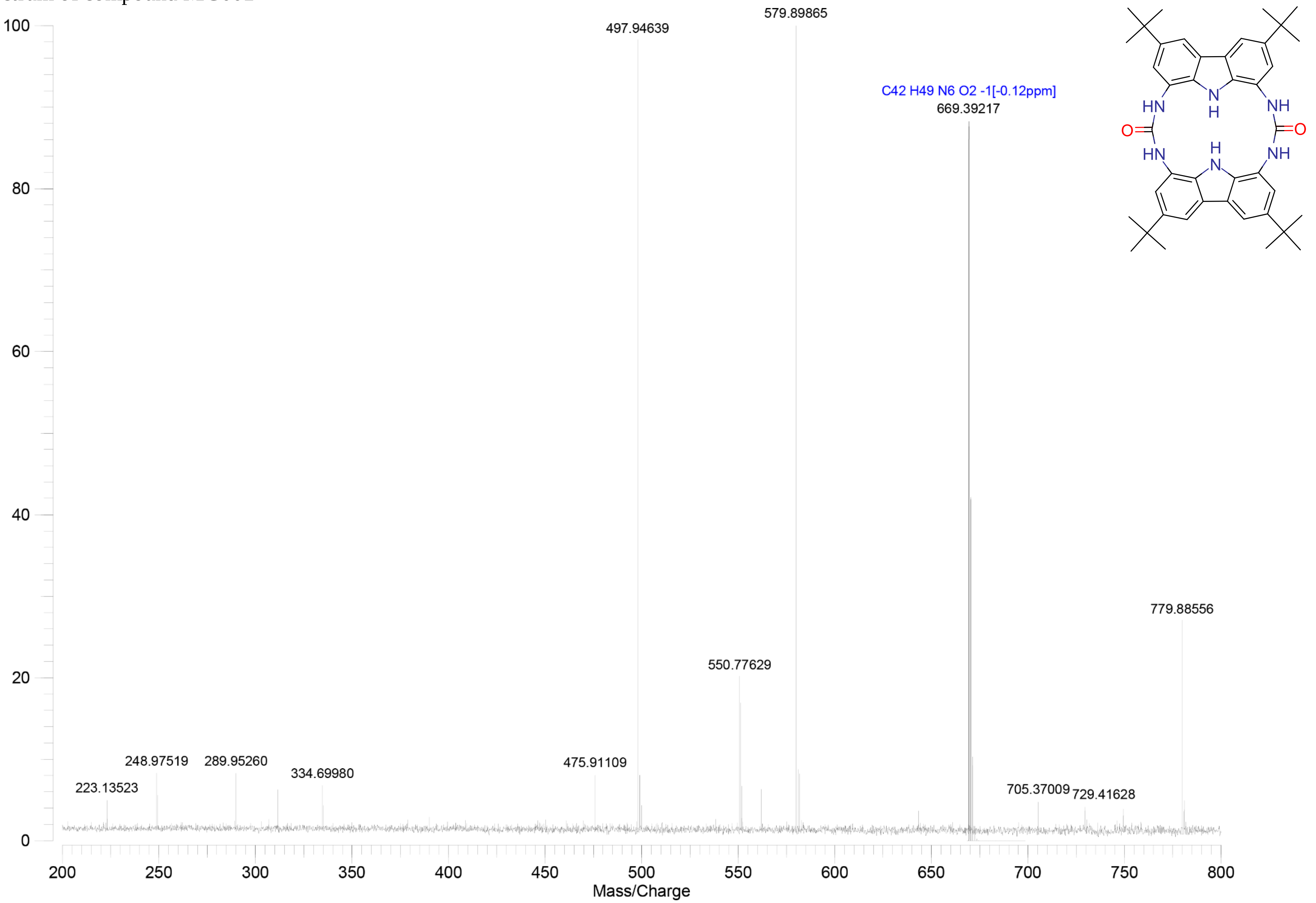

^{1}H NMR spectrum (700.1 MHz) of compound **MC002**

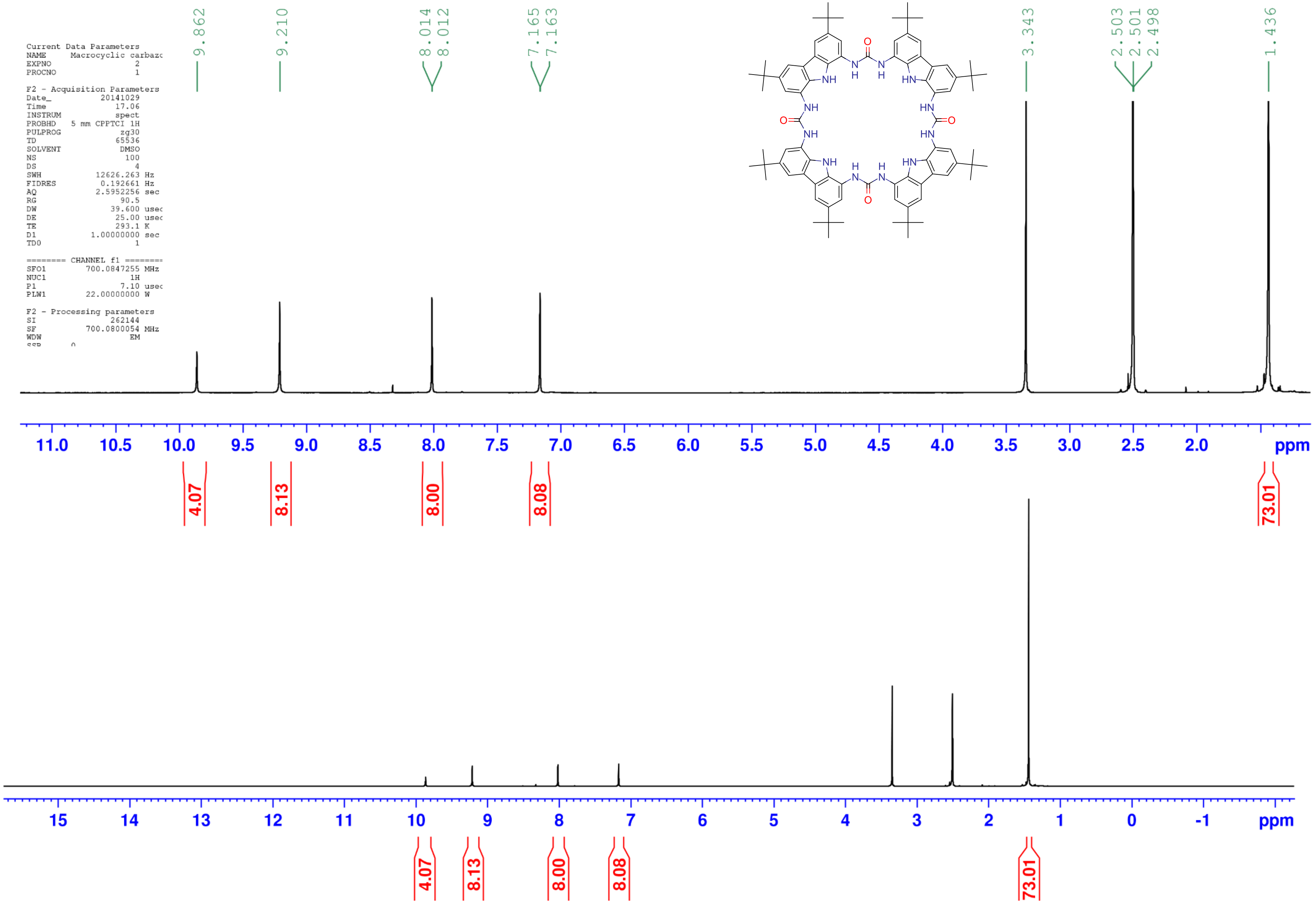

^{13}C NMR spectrum (700.1 MHz) of compound **MC002**

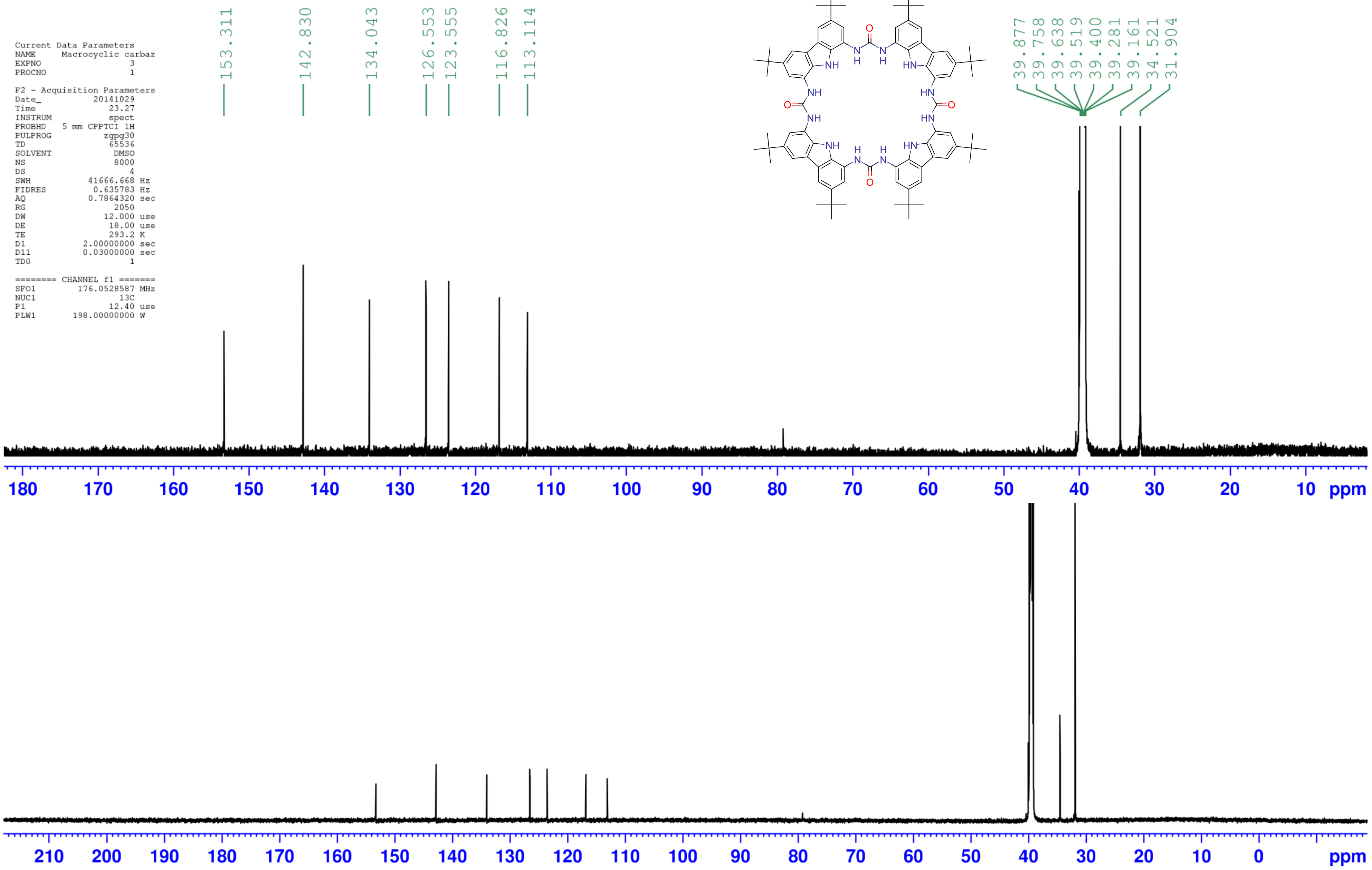

^{1}H-^{13}C HSQC spectrum (700.1 MHz) of compound **MC002**

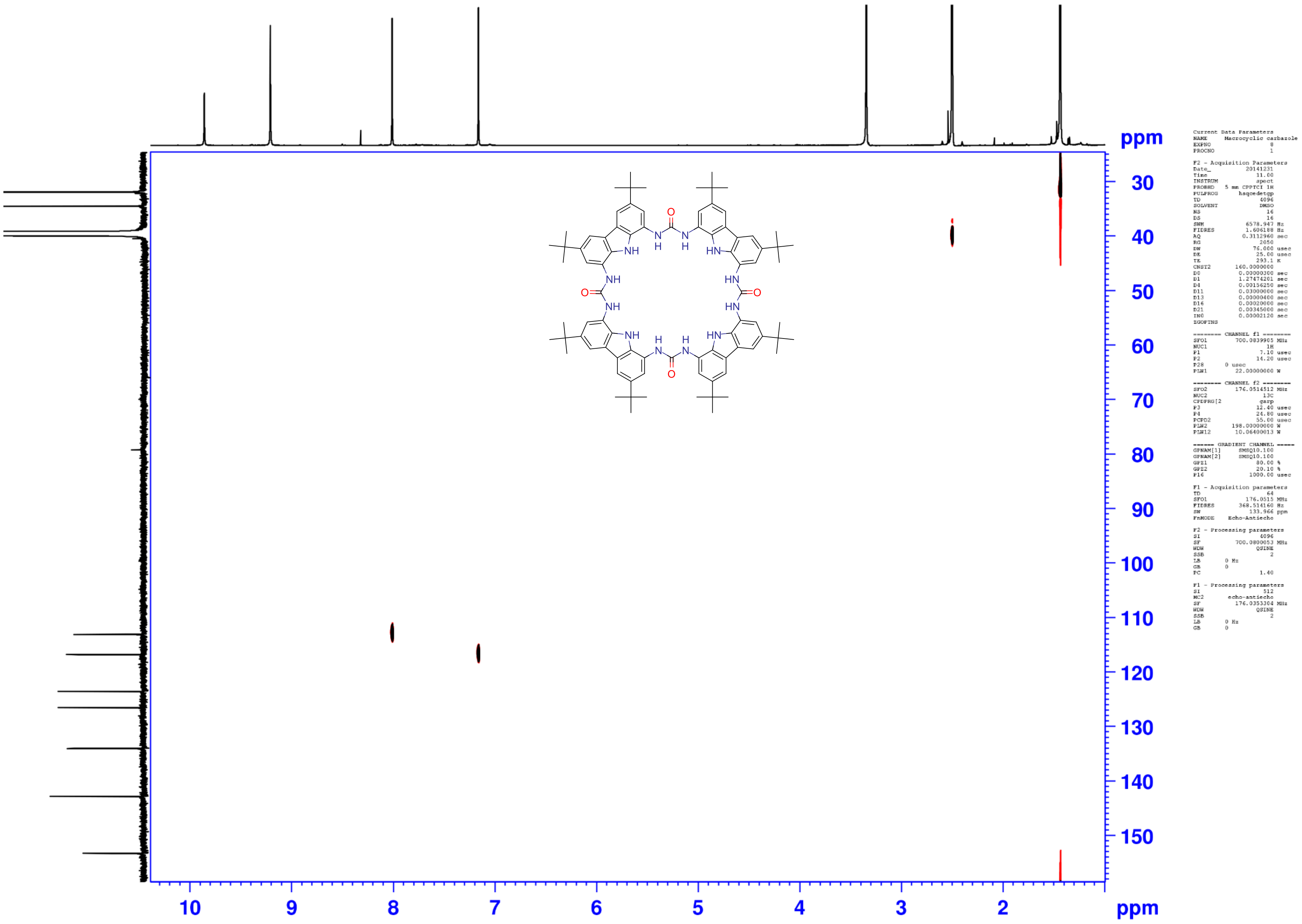

^{1}H-^{13}C HMBC spectrum (700.1 MHz) of compound **MC002**

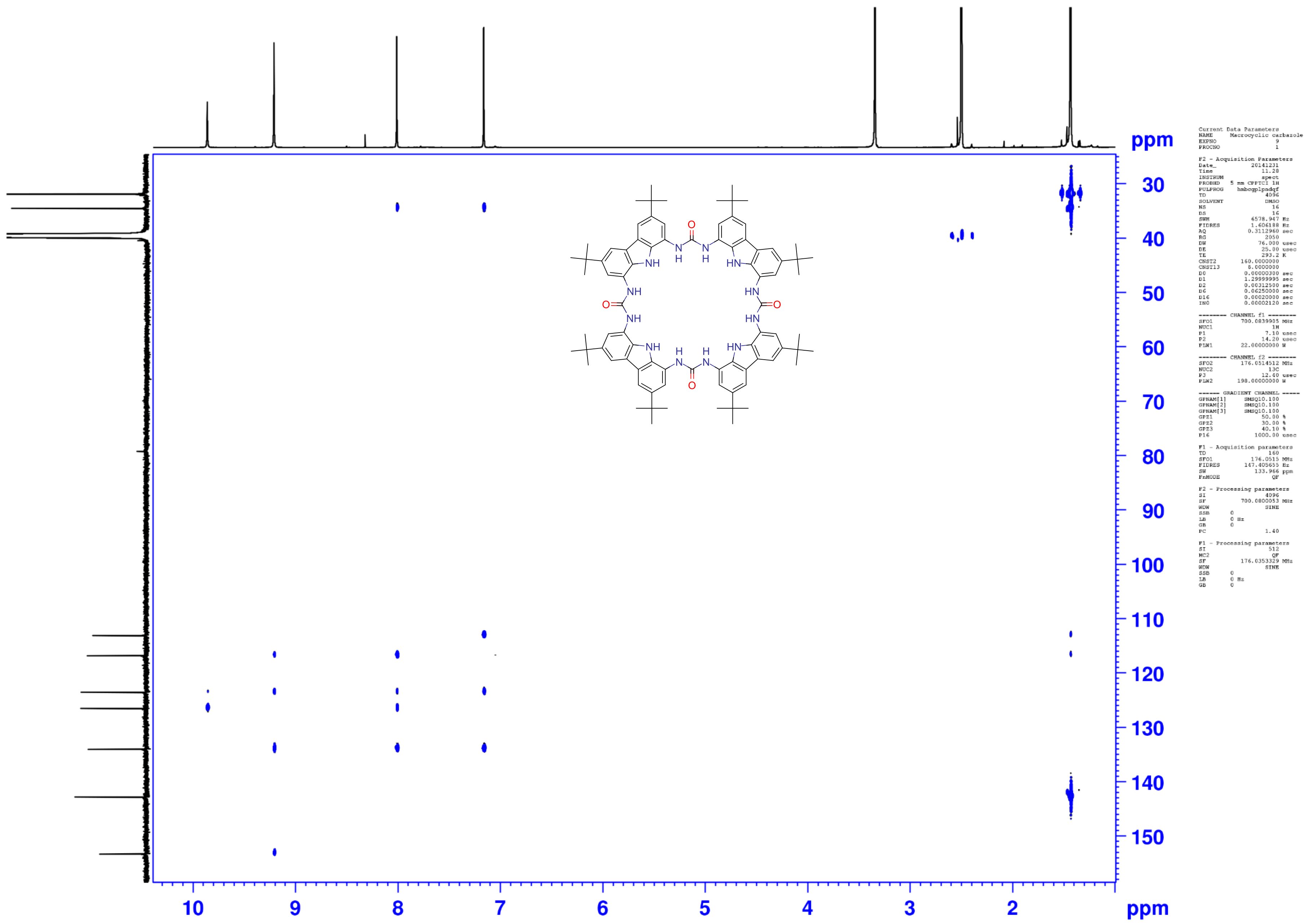

HRMS spectrum of compound **MC002**

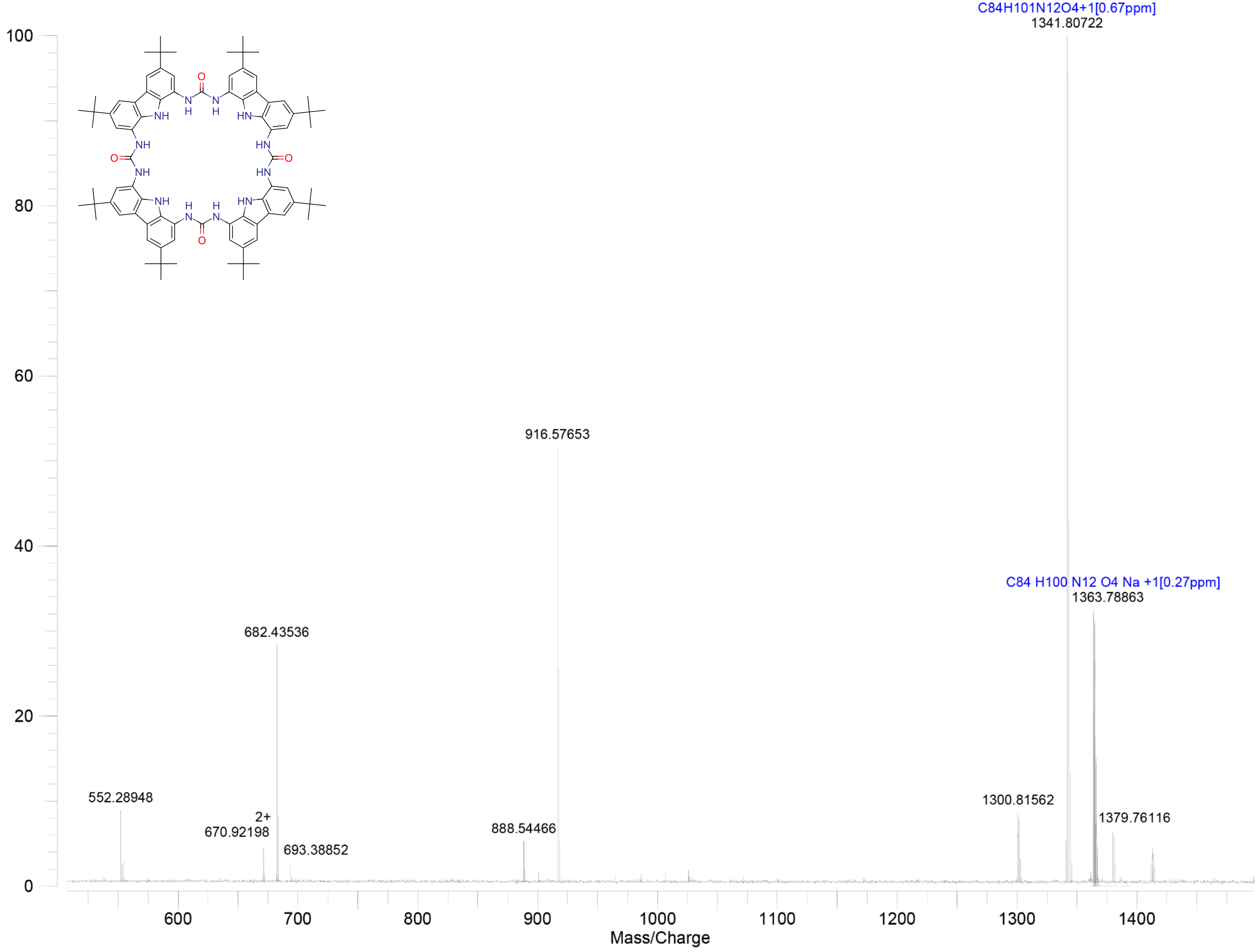

^{1}H NMR spectrum (700.1 MHz) of compound **MC003**

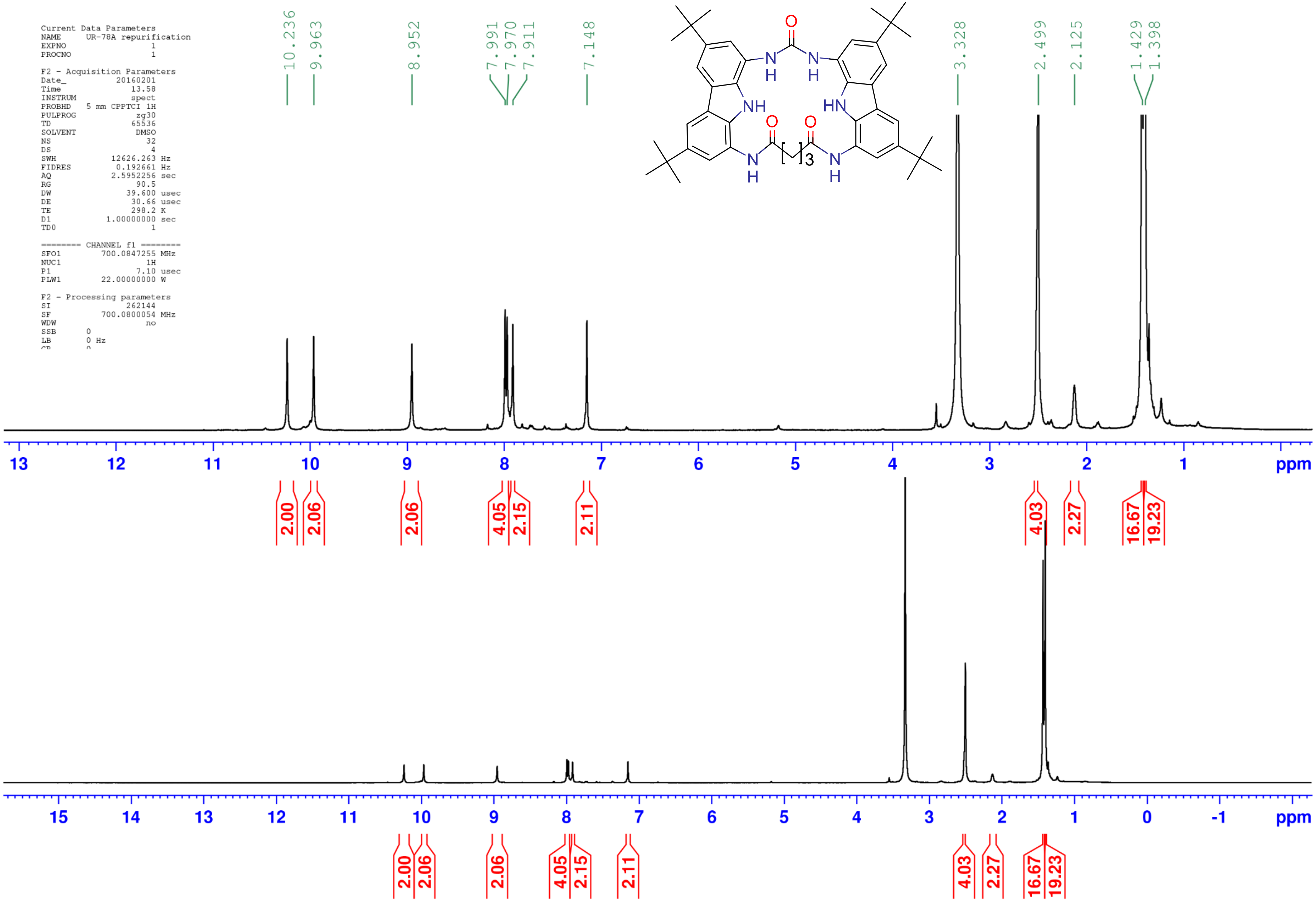

^{13}C NMR spectrum (700.1 MHz) of compound **MC003**

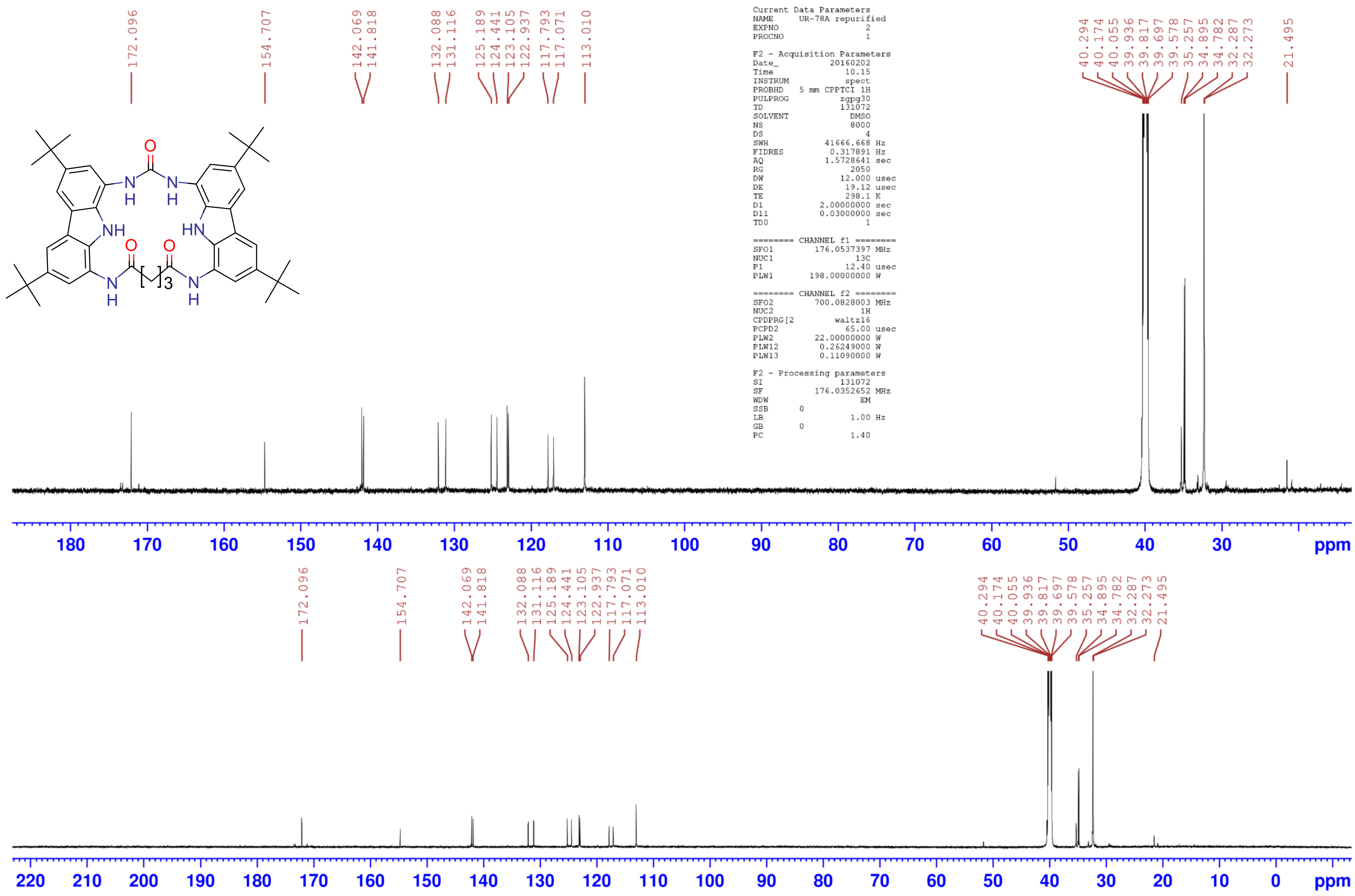

^{1}H-^{13}C HSQC spectrum (700.1 MHz) of compound **MC003**

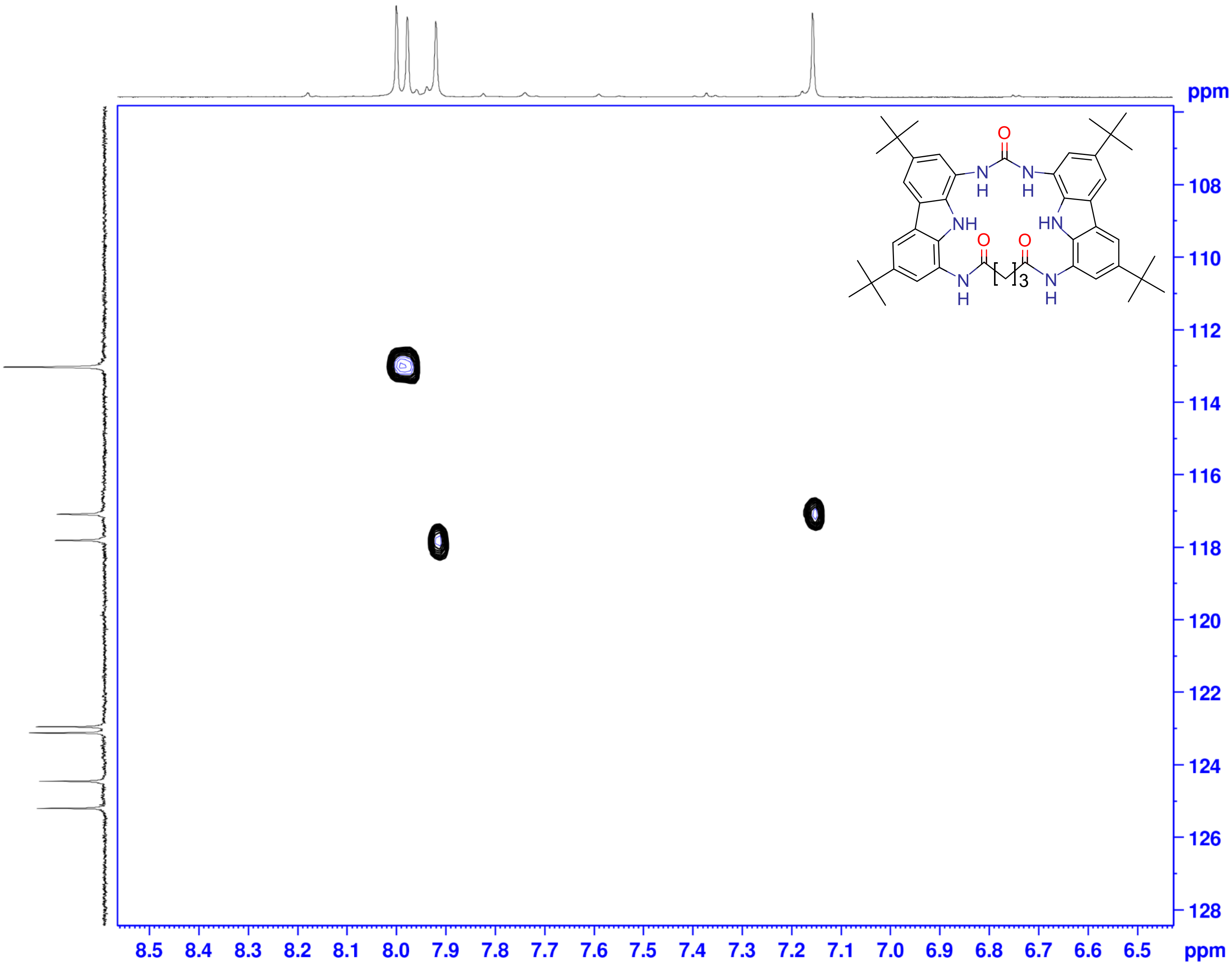


```
Current Data Parameters
NAME              UR-78A
EXPNO                  4
PROCNO                 1

F2 - Acquisition Parameters
Date_           20160602
Time               15.10
INSTRUM            spect
PROBHD   5 mm PABBO BB-
PULPROG       hsqcedetgp
TD                  1024
SOLVENT             DMSO
NS                     2
DS                    16
SWH             7246.377 Hz
FIDRES          7.076540 Hz
AQ             0.0706560 sec
RG                  2050
DW                69.000 usec
DE                  6.50 usec
TE                 298.2 K
CNST2        145.0000000
D0            0.00000300 sec
D1            1.50389099 sec
D4            0.00172414 sec
D11           0.03000000 sec
D13           0.00000400 sec
D16           0.00020000 sec
D21           0.00345000 sec
IN0           0.00001830 sec
ZGOPTNS

======== CHANNEL f1 ========
SFO1         700.0840045 MHz
NUC1                  1H
P1                 16.40 usec
P2                 32.80 usec
P28              1000.00 usec
PLW1         26.00000000 W

======== CHANNEL f2 ========
SFO2         176.0541798 MHz
NUC2                 13C
CPDPRG[2            garp
P3                 10.00 usec
P4                 20.00 usec
PCPD2              55.00 usec
PLW2         60.00000000 W
PLW12         1.98350000 W

====== GRADIENT CHANNEL =====
GPNAM[1]       SMSQ10.100
GPNAM[2]       SMSQ10.100
GPZ1               80.00 %
GPZ2               20.10 %
P16              1000.00 usec

F1 - Acquisition parameters
TD                   256
SFO1            176.0542 MHz
FIDRES        106.728142 Hz
SW               155.193 ppm
FnMODE    Echo-Antiecho

F2 - Processing parameters
SI                  1024
SF           700.0800000 MHz
WDW                QSINE
SSB                    2
LB                     0 Hz
GB                     0
PC                  1.40

F1 - Processing parameters
SI                  1024
MC2         echo-antiecho
SF           176.0352560 MHz
WDW                QSINE
SSB                    2
LB                     0 Hz
GB                     0
```

^{1}H-^{13}C HMBC spectrum (700.1 MHz) of compound **MC003**

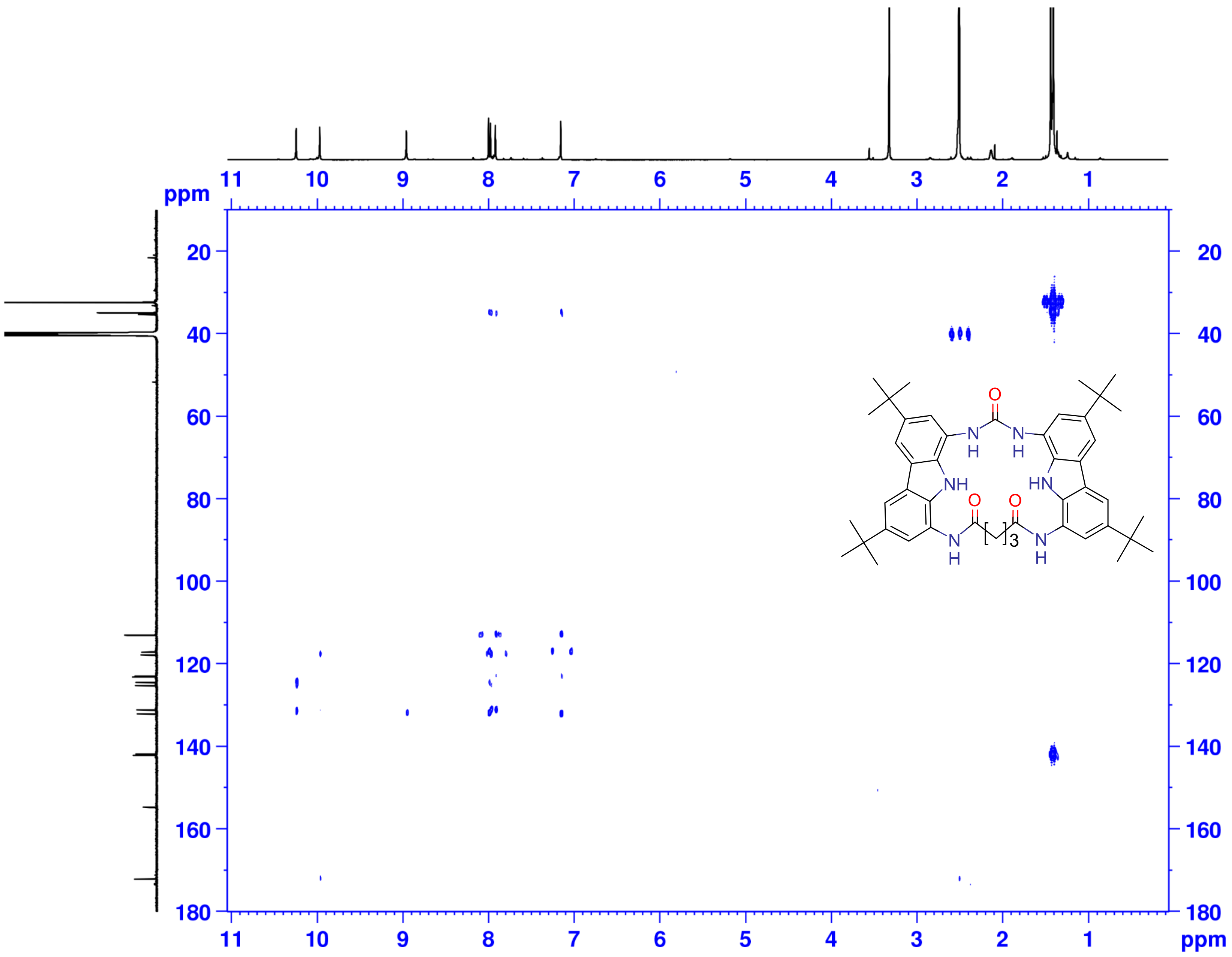

Current Data Parameters
NAME UR-78A
EXPNO 9
PROCNO 1

F2 - Acquisition Parameters
Date_ 20160602
Time 17.46
INSTRUM spect
PROBHD 5 mm PABBO BB-
PULPROG hmbcgpndqf
TD 4096
SOLVENT DMSO
NS 8
DS 16
SWH 7692.308 Hz
FIDRES 1.878005 Hz
AQ 0.2662400 sec
RG 2050
DW 65.000 usec
DE 6.50 usec
TE 298.1 K
CNST13 8.0000000
D0 0.00000300 sec
D1 1.52621400 sec
D6 0.06250000 sec
D16 0.00020000 sec
IN0 0.00001670 sec

======== CHANNEL f1 ========
SFO1 700.0838895 MHz
NUC1 1H
P1 16.40 usec
P2 32.80 usec
PLW1 26.00000000 W

======== CHANNEL f2 ========
SFO2 176.0519793 MHz
NUC2 13C
P3 10.00 usec
PLW2 60.00000000 W

====== GRADIENT CHANNEL =====
GPNAM[1] SMSQ10.100
GPNAM[2] SMSQ10.100
GPNAM[3] SMSQ10.100
GPZ1 50.00 %
GPZ2 30.00 %
GPZ3 40.10 %
P16 1000.00 usec

F1 - Acquisition parameters
TD 128
SFO1 176.052 MHz
FIDRES 233.907181 Hz
SW 170.064 ppm
FnMODE QF

F2 - Processing parameters
SI 1024
SF 700.0800000 MHz
WDW SINE
SSB 0
LB 0 Hz
GB 0
PC 1.40

F1 - Processing parameters
SI 1024
MC2 QF
SF 176.0352560 MHz
WDW SINE
SSB 0
LB 0 Hz
GB 0

HRMS spectrum of compound **MC003**

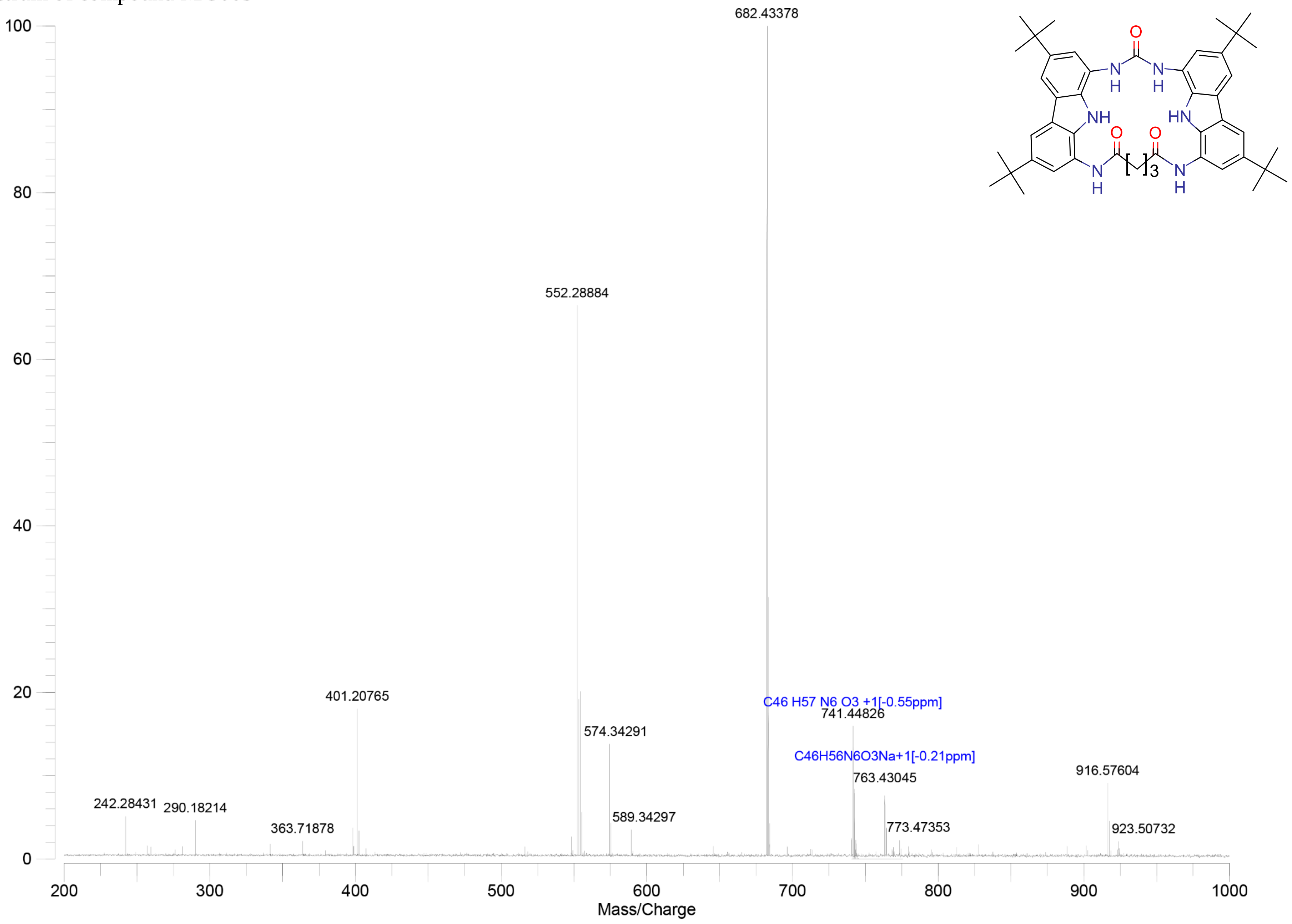

^{1}H NMR spectrum (700.1 MHz) of compound **MC004**

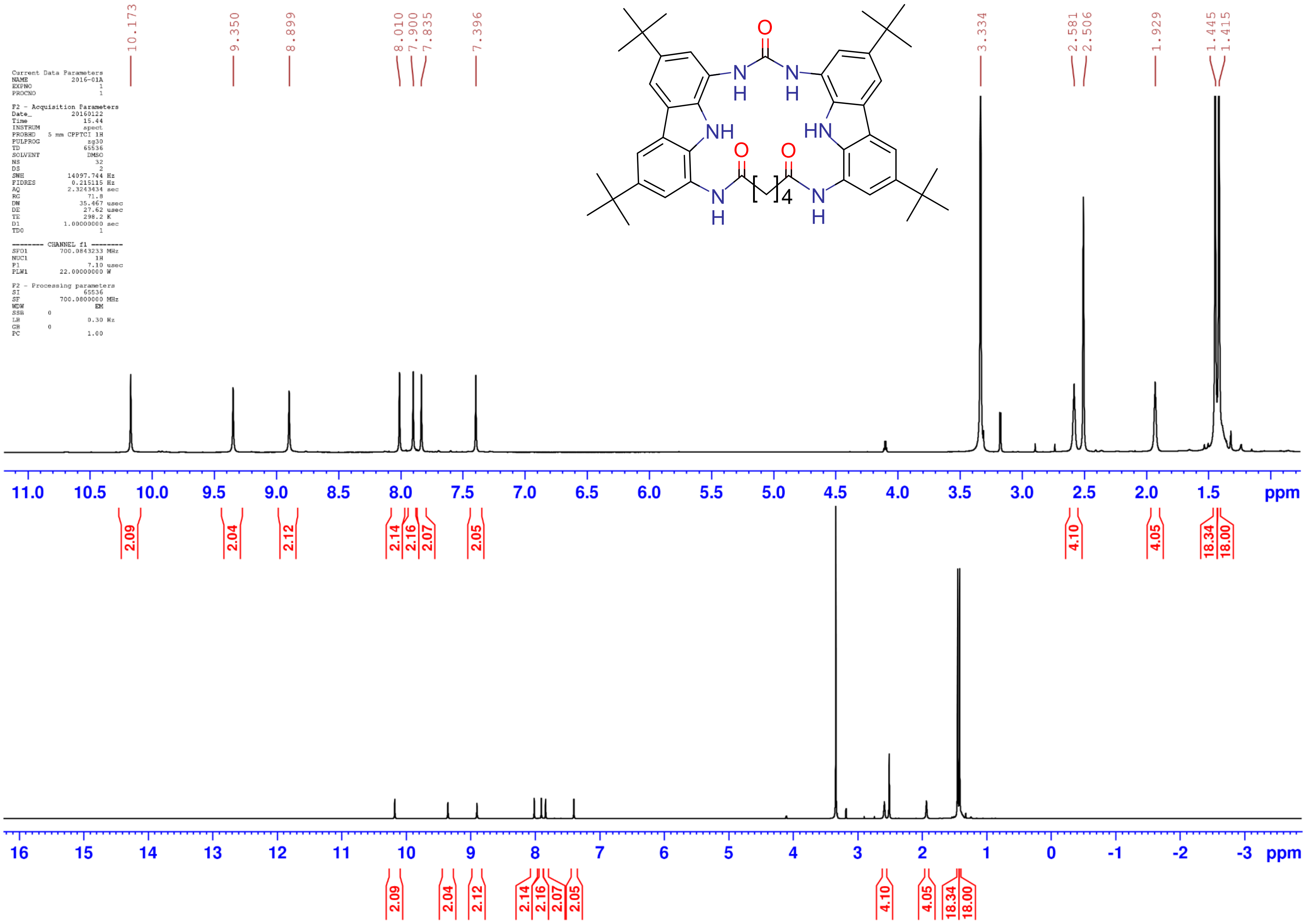

^{13}C NMR spectrum (700.1 MHz) of compound **MC004**

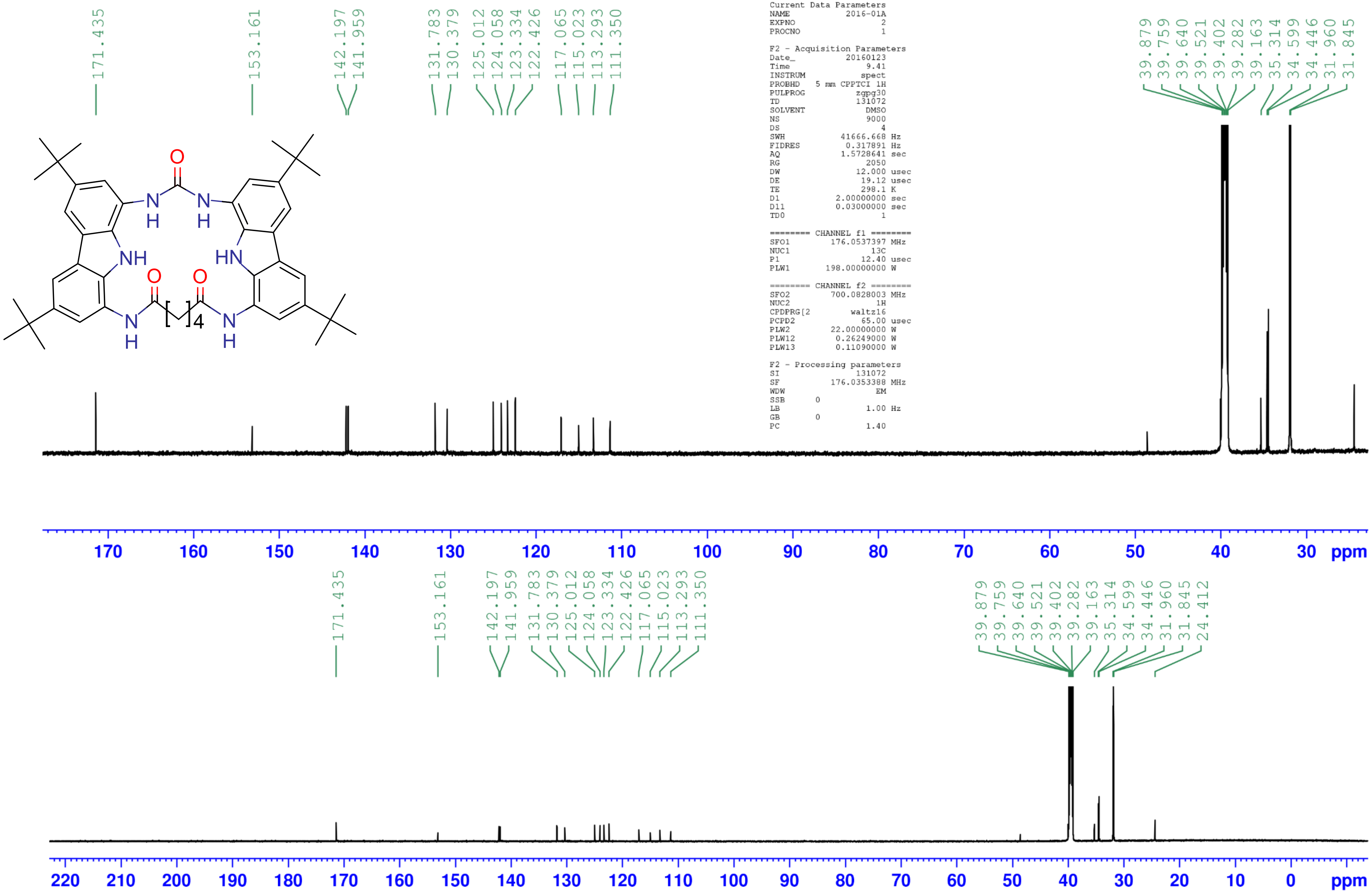

^{1}H-^{13}C HSQC spectrum (700.1 MHz) of compound **MC004**

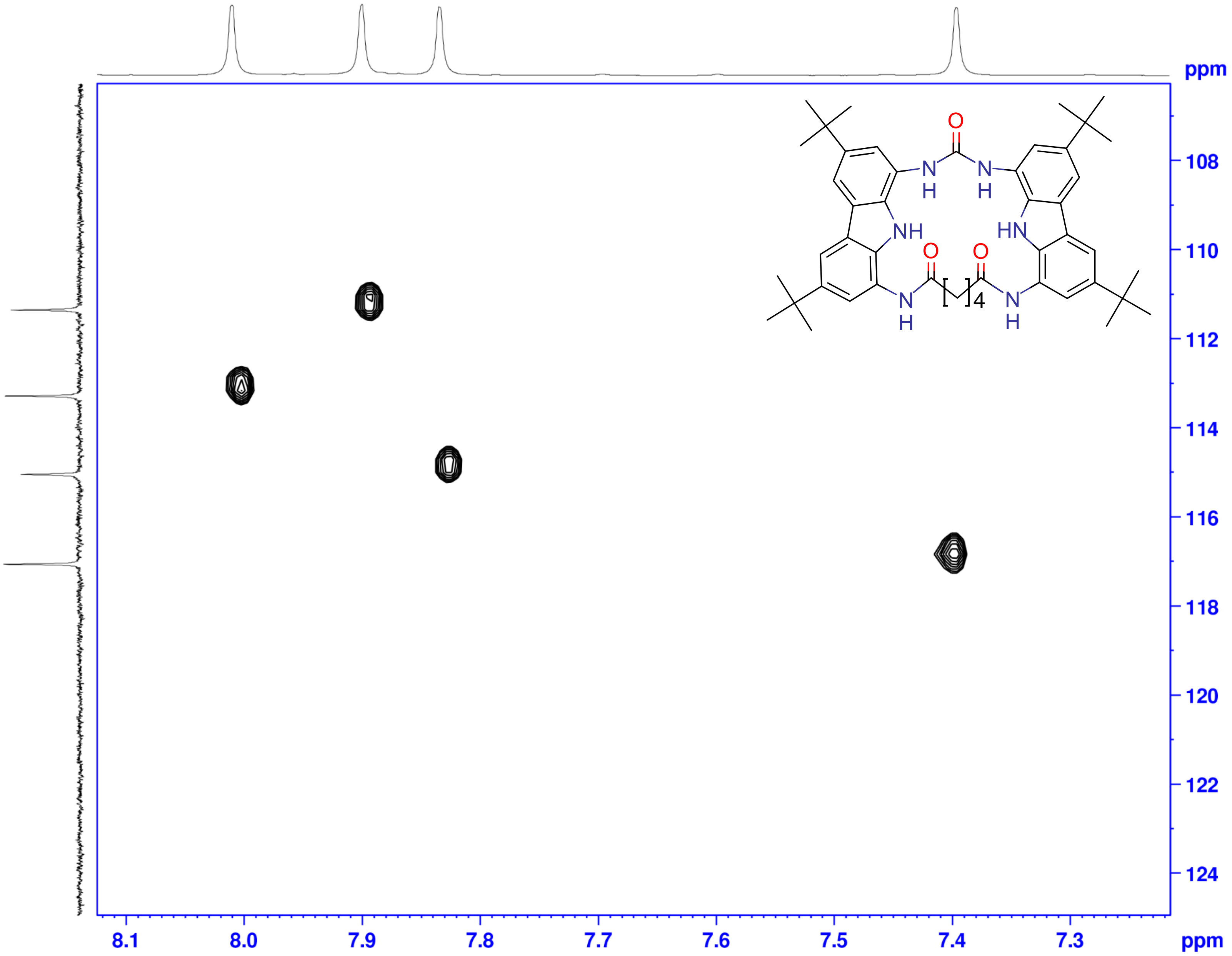


```
Current Data Parameters
NAME              2016-01A
EXPNO                    5
PROCNO                   1

F2 - Acquisition Parameters
Date_             20160719
Time                 13.03
INSTRUM              spect
PROBHD   5 mm PABBO BB-
PULPROG  hsqcdietgpsisp.2
TD                    2048
SOLVENT               DMSO
NS                       4
DS                      32
SWH              6893.382 Hz
FIDRES           3.365909 Hz
AQ              0.1485483 sec
RG                    2050
DW                  72.533 usec
DE                    6.50 usec
TE                   298.3 K
CNST2        145.0000000
CNST17        -0.5000000
D0            0.00000300 sec
D1            2.00000000 sec
D4            0.00172414 sec
D9            0.06000000 sec
D11           0.03000000 sec
D16           0.00020000 sec
D24           0.00089000 sec
IN0           0.00001800 sec
L1                      20

======== CHANNEL f1 ========
SFO1         700.0841235 MHz
NUC1                    1H
P1                   16.40 usec
P2                   32.80 usec
P6                   26.00 usec
P28                1000.00 usec
PLW1          26.00000000 W
PLW10         10.34500027 W

======== CHANNEL f2 ========
SFO2         176.0524705 MHz
NUC2                   13C
CPDPRG[2 bi_p5m4sp_4sp.2
P3                   10.00 usec
P14                 500.00 usec
P24                2000.00 usec
P63                1500.00 usec
PLW0      0 W
PLW2          60.00000000 W
PLW12          1.98350000 W
SPNAM[3] Crp60,0.5,20.1
SPOAL3               0.500
SPOFFS3  0 Hz
SPW3           9.16730022 W
SPNAM[7]     Crp60comp.4
SPOAL7               0.500
SPOFFS7  0 Hz
SPW7           9.16730022 W
SPNAM[14 Crp42,1.5,20.2
SPOAL14              0.500
SPOFFS14 0 Hz
SPW14          5.13369989 W
SPNAM[31 Crp42,1.5,20.2
SPOAL31              0.500
SPOFFS31 0 Hz
SPW31          1.28340006 W

====== GRADIENT CHANNEL =====
GPNAM[1]       SMSQ10.100
GPNAM[2]       SMSQ10.100
GPZ1                 80.00 %
GPZ2                 20.10 %
P16                1000.00 usec

F1 - Acquisition parameters
TD                     256
SFO1              176.0525 MHz
FIDRES          108.506943 Hz
SW                 157.781 ppm
FnMODE      Echo-Antiecho

F2 - Processing parameters
SI                    2048
SF            700.0800000 MHz
WDW                  QSINE
SSB                      2
LB        0 Hz
GB        0
PC                    1.40

F1 - Processing parameters
SI                     512
MC2         echo-antiecho
SF            176.0353390 MHz
WDW                  QSINE
SSB                      2
LB        0 Hz
GB        0
```

^{1}H-^{13}C HMBC spectrum (700.1 MHz) of compound **MC004**

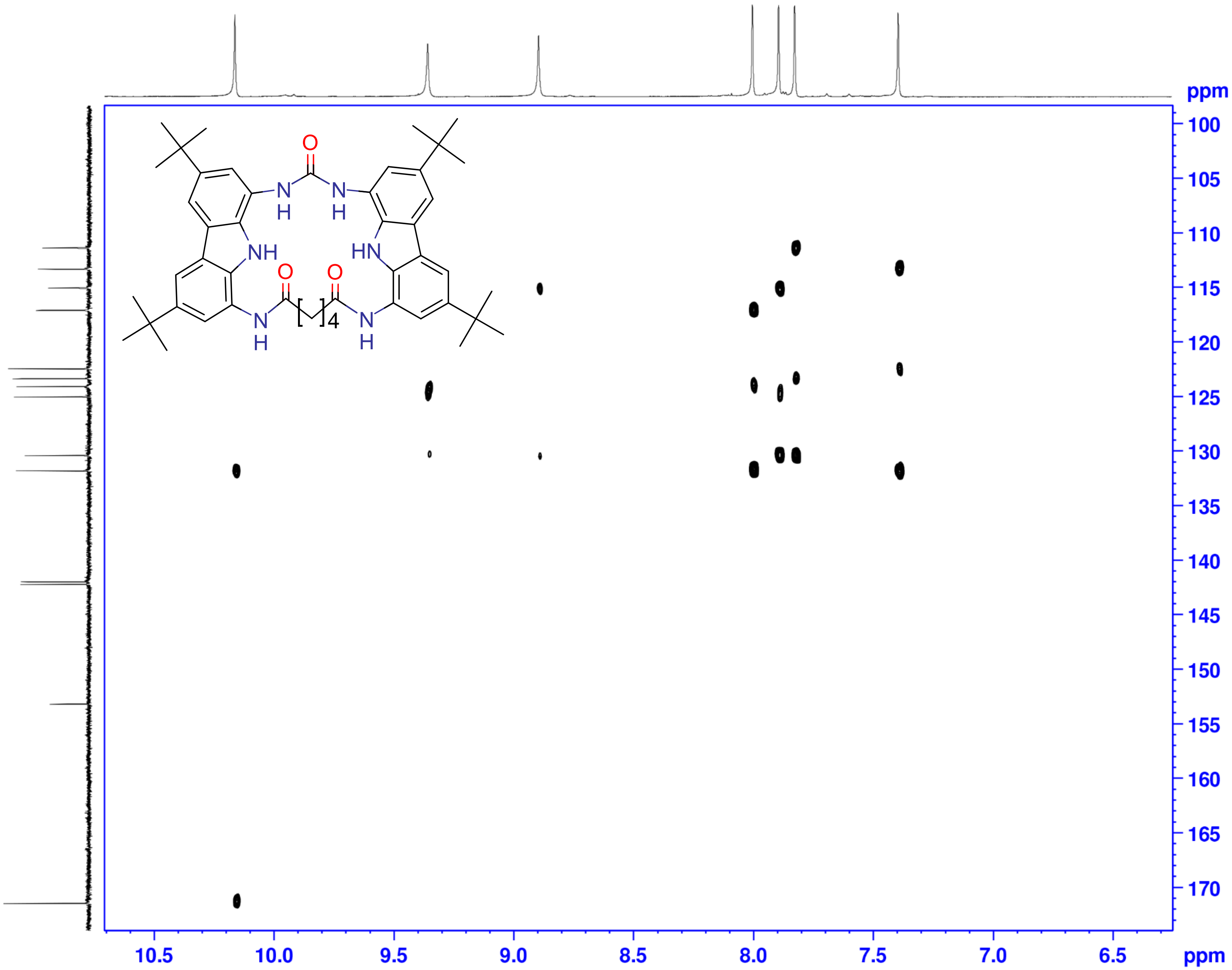


```
Current Data Parameters
NAME            2016-01A
EXPNO                  9
PROCNO                 1

F2 - Acquisition Parameters
Date_           20160721
Time                9.10
INSTRUM            spect
PROBHD   5 mm PABBO BB-
PULPROG   hmbcgplpndqf
TD                  2048
SOLVENT             DMSO
NS                    16
DS                    16
SWH             7692.308 Hz
FIDRES          3.756010 Hz
AQ             0.1331200 sec
RG                   287
DW                65.000 usec
DE                  6.50 usec
TE                 298.2 K
CNST2        145.0000000
CNST13         7.0000000
D0            0.00000300 sec
D1            1.50000000 sec
D2            0.00344828 sec
D6            0.07142857 sec
D16           0.00020000 sec
IN0           0.00001780 sec

======== CHANNEL f1 ========
SFO1         700.0838854 MHz
NUC1                  1H
P1                 16.40 usec
P2                 32.80 usec
PLW1         26.00000000 W

======== CHANNEL f2 ========
SFO2         176.0525075 MHz
NUC2                 13C
P3                 10.00 usec
PLW2         60.00000000 W

====== GRADIENT CHANNEL =====
GPNAM[1]      SMSQ10.100
GPNAM[2]      SMSQ10.100
GPNAM[3]      SMSQ10.100
GPZ1               50.00 %
GPZ2               30.00 %
GPZ3               40.10 %
P16              1000.00 usec

F1 - Acquisition parameters
TD                   160
SFO1            176.0525 MHz
FIDRES        175.561798 Hz
SW               159.554 ppm
FnMODE                QF

F2 - Processing parameters
SI                  2048
SF           700.0800045 MHz
WDW                 SINE
SSB      0
LB       0 Hz
GB       0
PC                  1.40

F1 - Processing parameters
SI                   512
MC2                   QF
SF           176.0353388 MHz
WDW                 SINE
SSB      0
LB       0 Hz
GB       0
```

HRMS spectrum of compound **MC004**

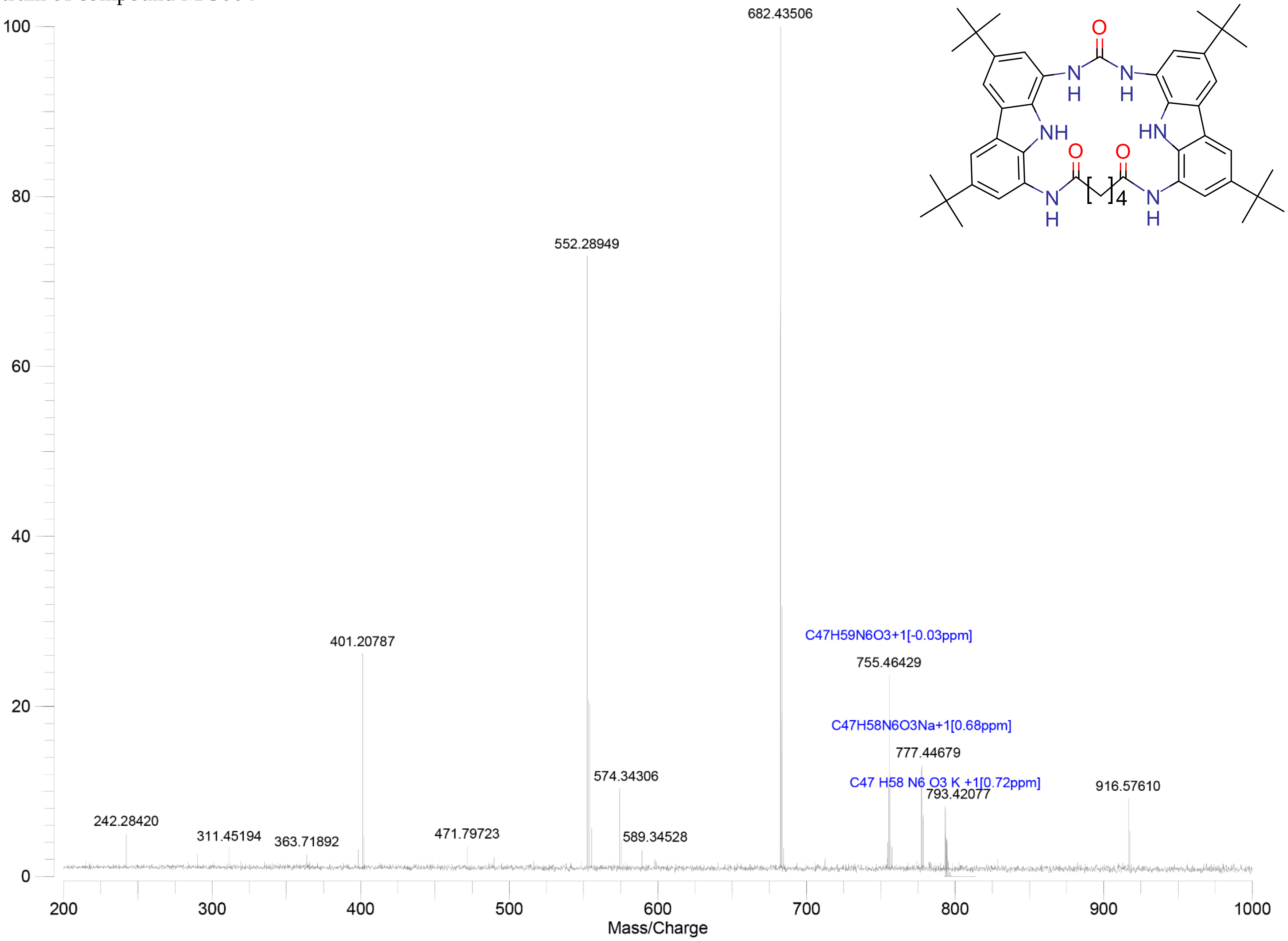

^{1}H NMR spectrum (700.1 MHz) of compound **MC005**

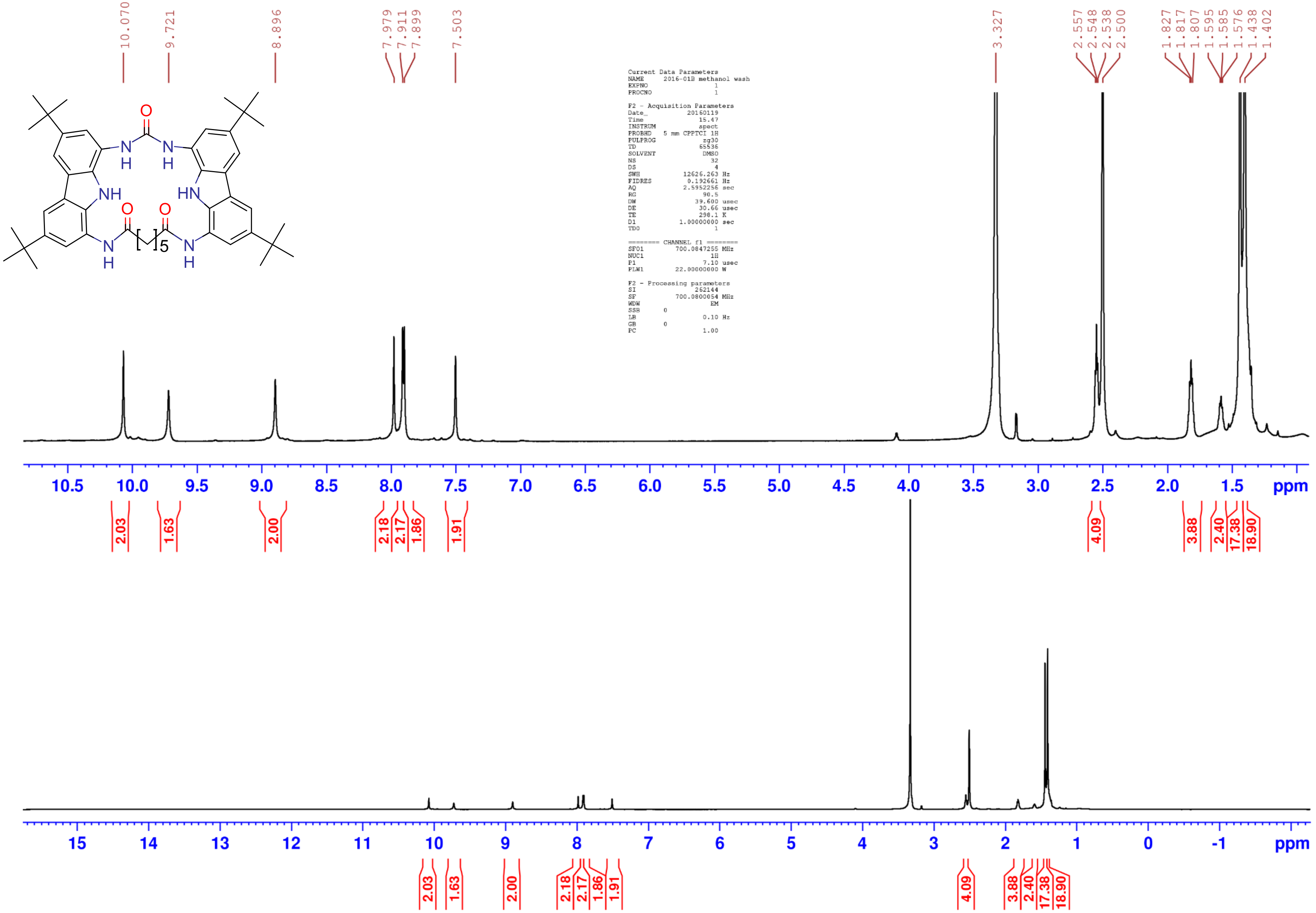

^{13}C NMR spectrum (700.1 MHz) of compound **MC005**

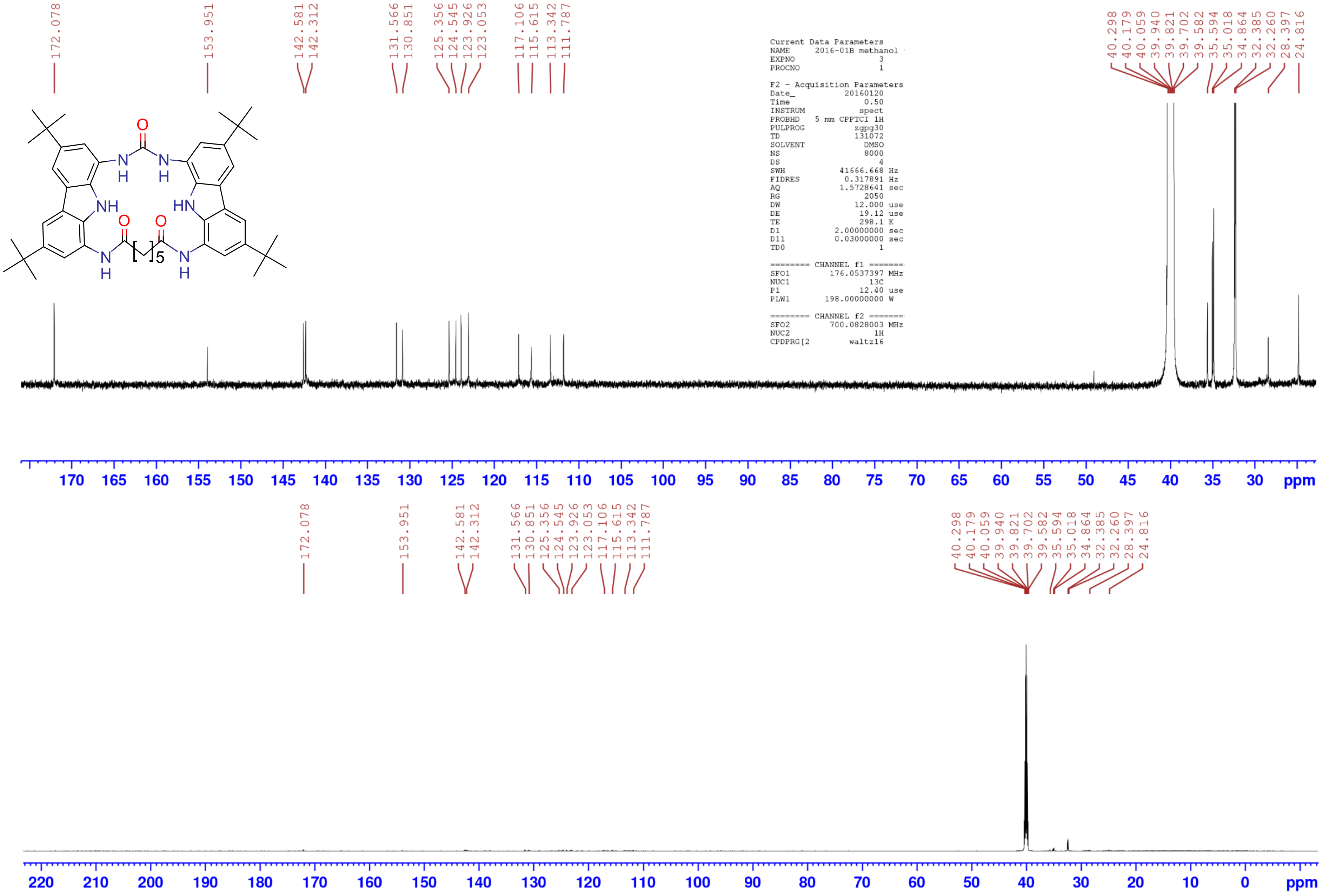

^{1}H-^{13}C HSQC spectrum (700.1 MHz) of compound **MC005**

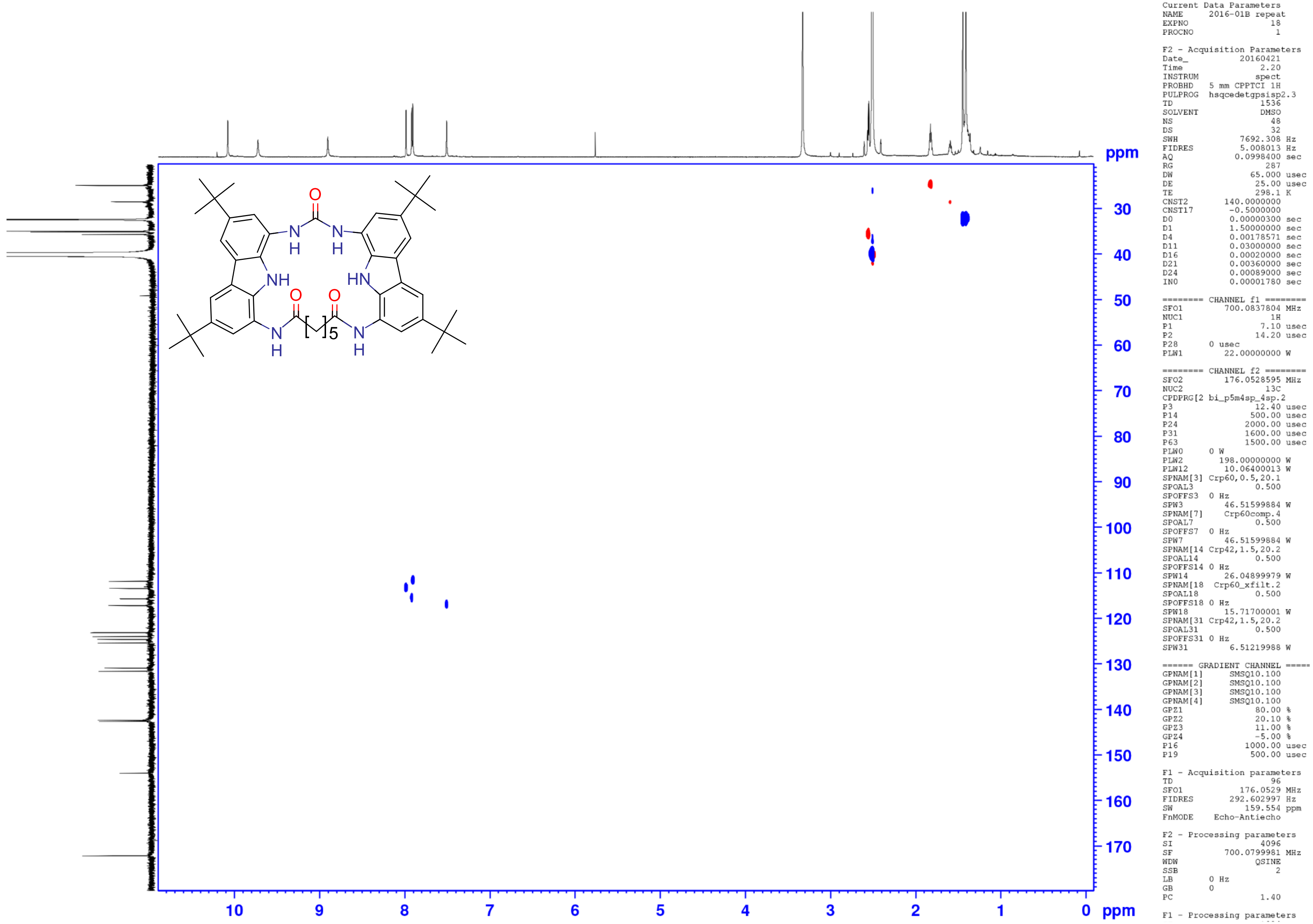

^{1}H-^{13}C HMBC spectrum (700.1 MHz) of compound **MC005**

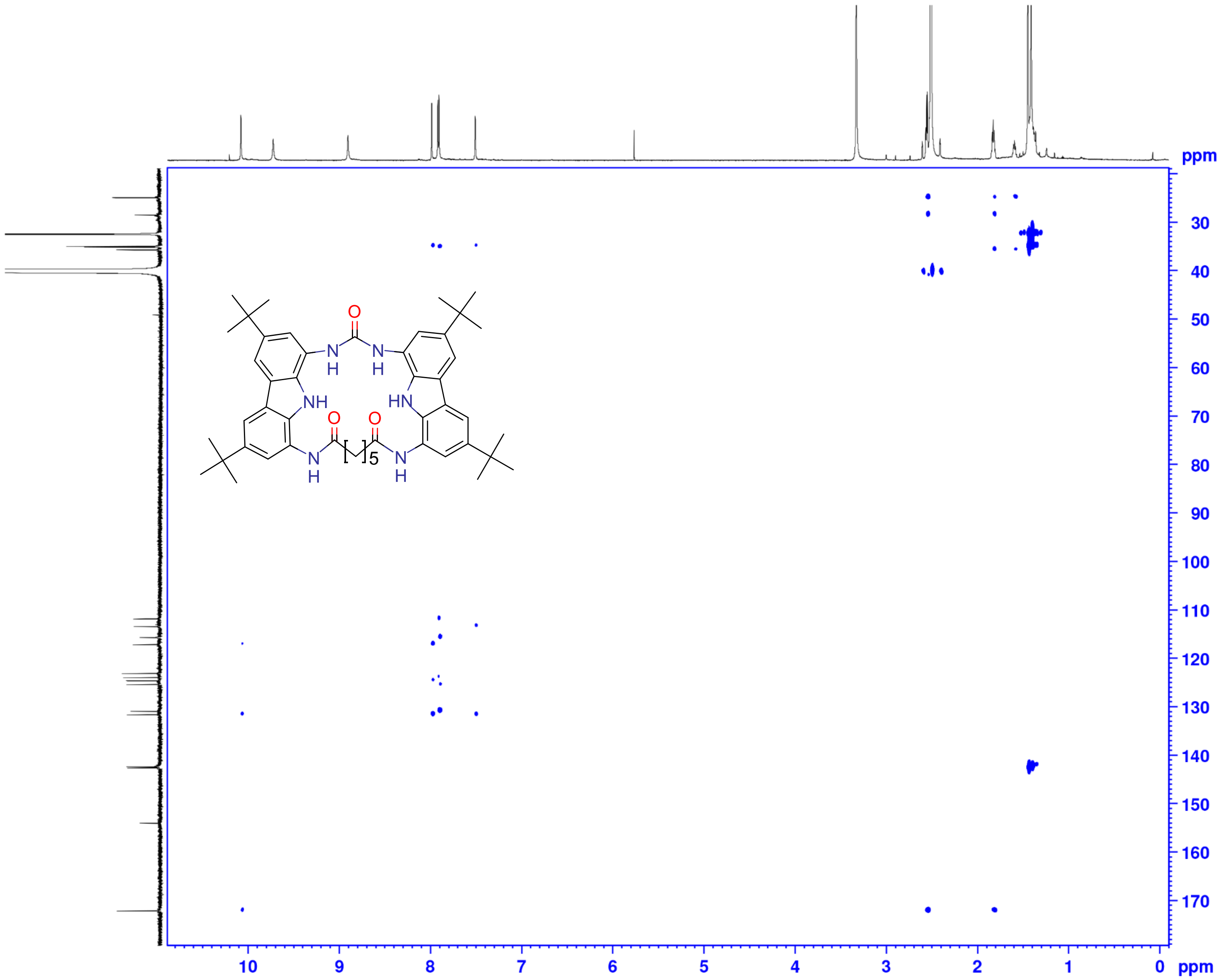


```
Current Data Parameters
NAME      2016-01B repeat
EXPNO                  17
PROCNO                  1

F2 - Acquisition Parameters
Date_            20160420
Time                19.08
INSTRUM             spect
PROBHD   5 mm CPPTCI 1H
PULPROG     hmbcgplpndqf
TD                   3072
SOLVENT              DMSO
NS                     64
DS                     16
SWH              7692.308 Hz
FIDRES           2.504006 Hz
AQ              0.1996800 sec
RG                    812
DW                 65.000 usec
DE                  25.00 usec
TE                  298.2 K
CNST2         145.0000000
CNST13          8.0000000
D0             0.00000300 sec
D1             1.29999995 sec
D2             0.00344828 sec
D6             0.06250000 sec
D16            0.00020000 sec
IN0            0.00001770 sec

======== CHANNEL f1 ========
SFO1          700.0837804 MHz
NUC1                   1H
P1                   7.10 usec
P2                  14.20 usec
PLW1          22.00000000 W

======== CHANNEL f2 ========
SFO2          176.0526835 MHz
NUC2                  13C
P3                  12.40 usec
PLW2         198.00000000 W

====== GRADIENT CHANNEL =====
GPNAM[1]       SMSQ10.100
GPNAM[2]       SMSQ10.100
GPNAM[3]       SMSQ10.100
GPZ1                50.00 %
GPZ2                30.00 %
GPZ3                40.10 %
P16               1000.00 usec

F1 - Acquisition parameters
TD                    256
SFO1             176.0527 MHz
FIDRES         110.346046 Hz
SW                160.455 ppm
FnMODE                 QF

F2 - Processing parameters
SI                   4096
SF            700.0800053 MHz
WDW                  SINE
SSB       0
LB        0 Hz
GB        0
PC                   1.40

F1 - Processing parameters
SI                    512
MC2                    QF
SF            176.0352560 MHz
WDW                  SINE
SSB       0
LB        0 Hz
GB        0
```

HRMS spectrum of compound **MC005**

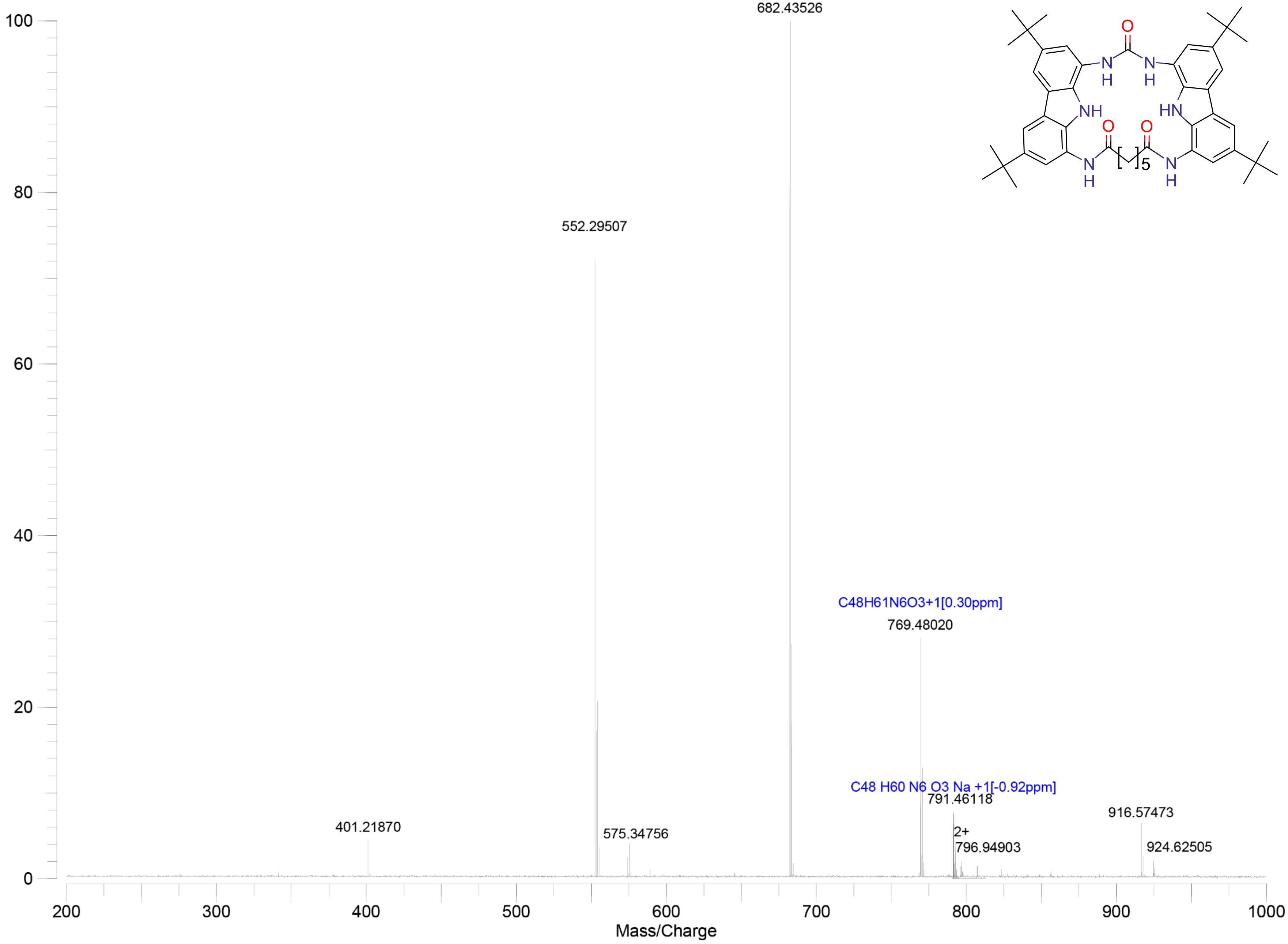

^{1}H NMR spectrum (700.1 MHz) of compound **MC006**

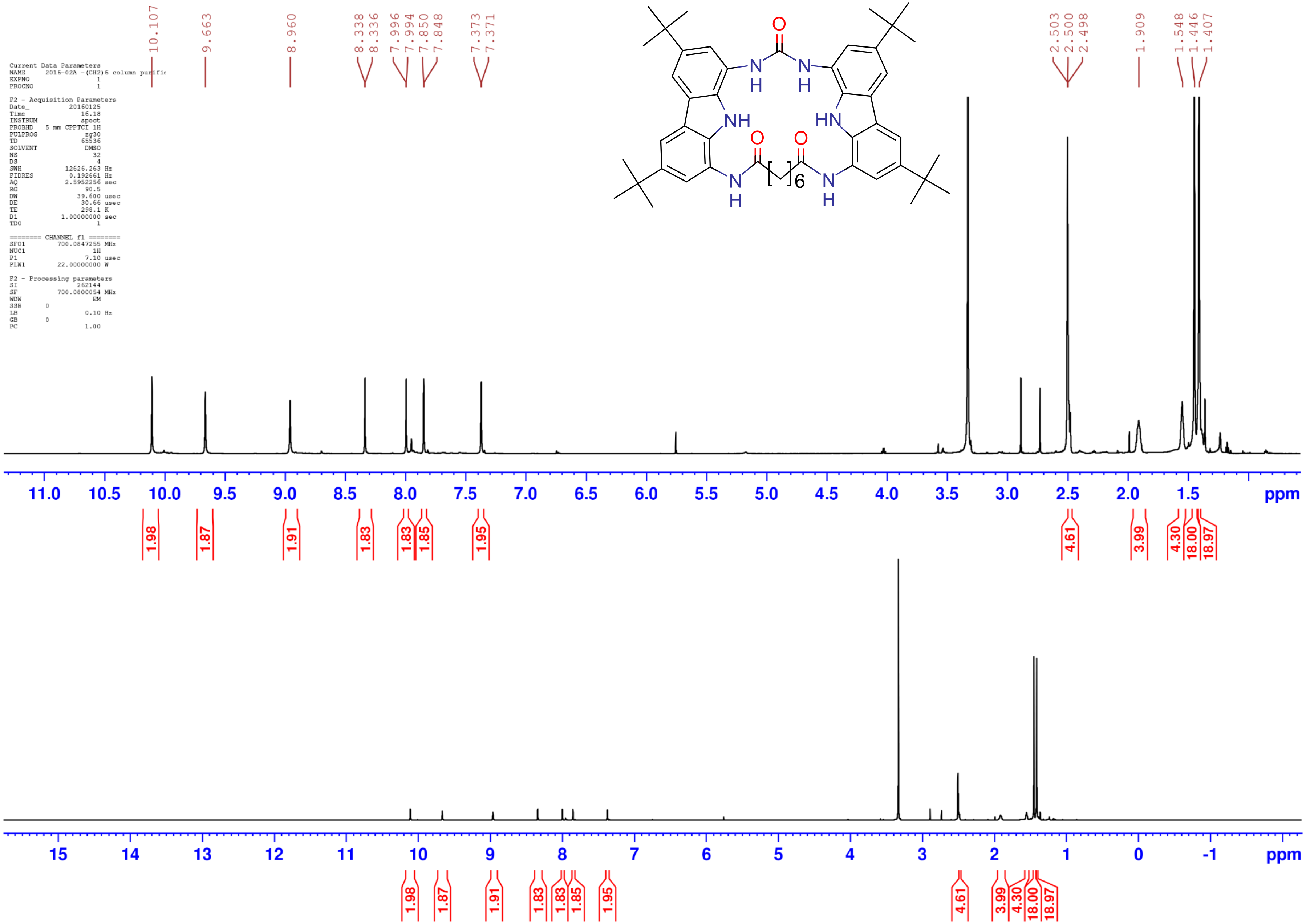

^{13}C NMR spectrum (700.1 MHz) of compound **MC006**

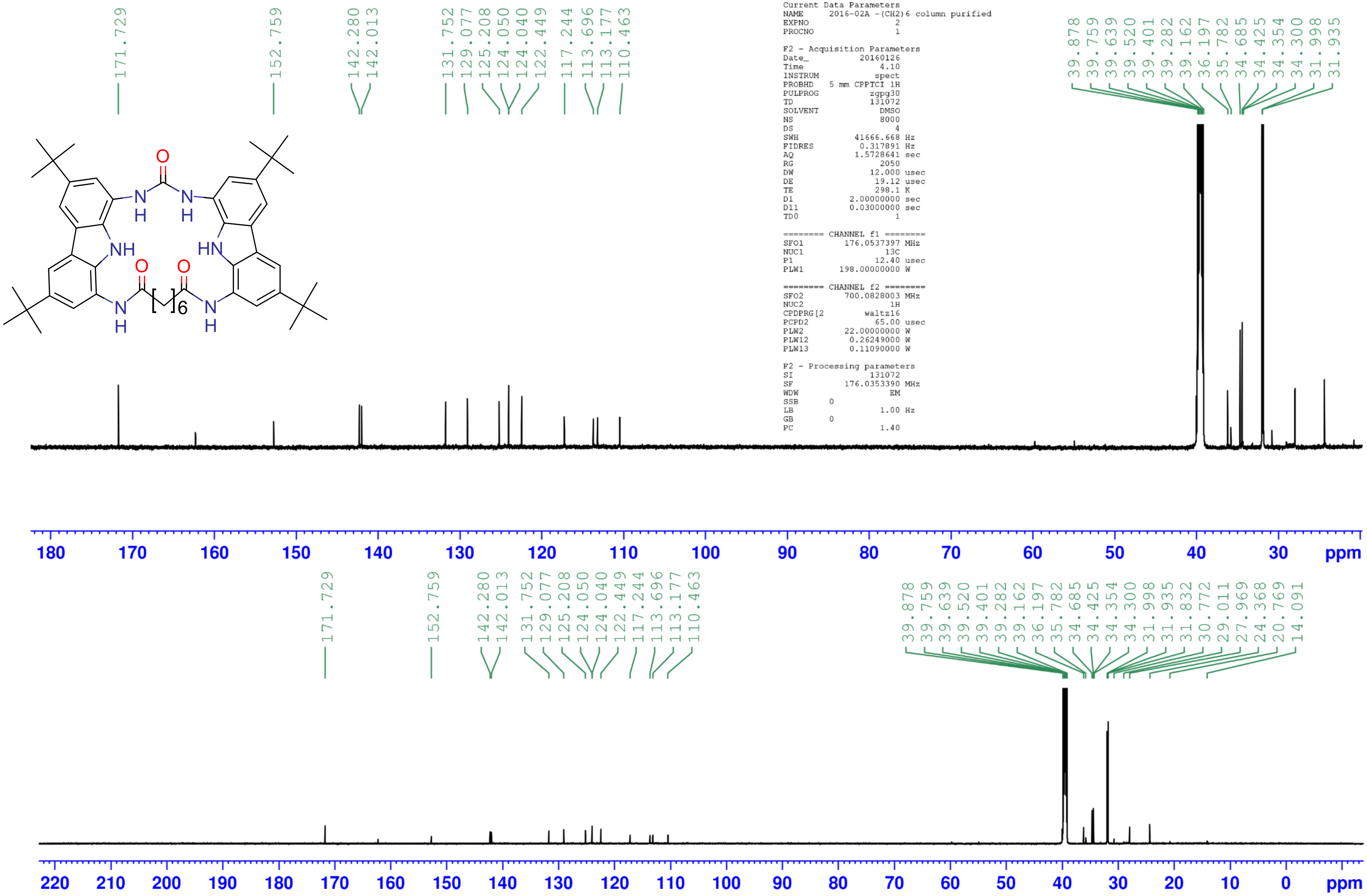

^{1}H-^{13}C HSQC spectrum (700.1 MHz) of compound **MC006**

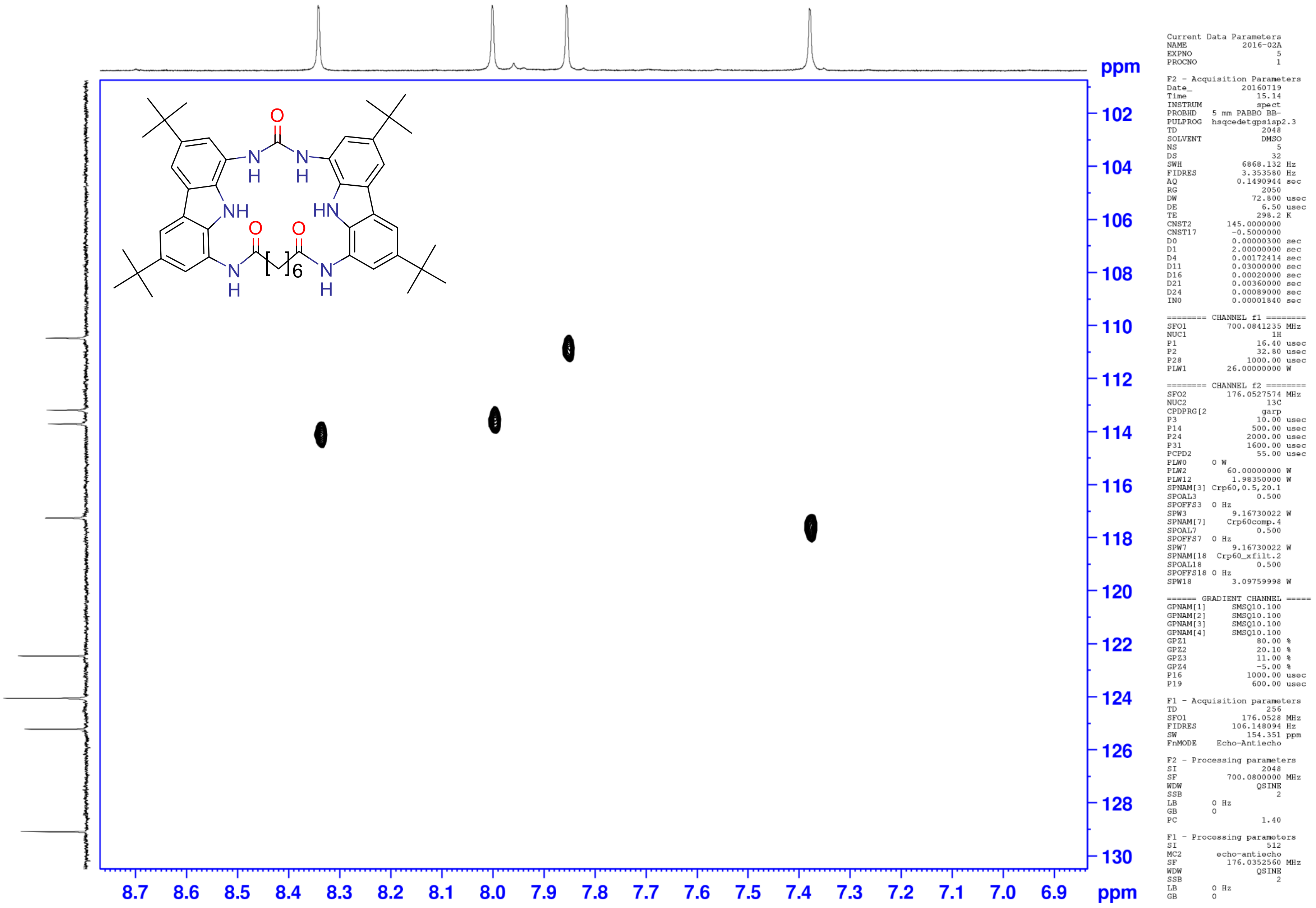

^{1}H-^{13}C HMBC spectrum (700.1 MHz) of compound **MC006**

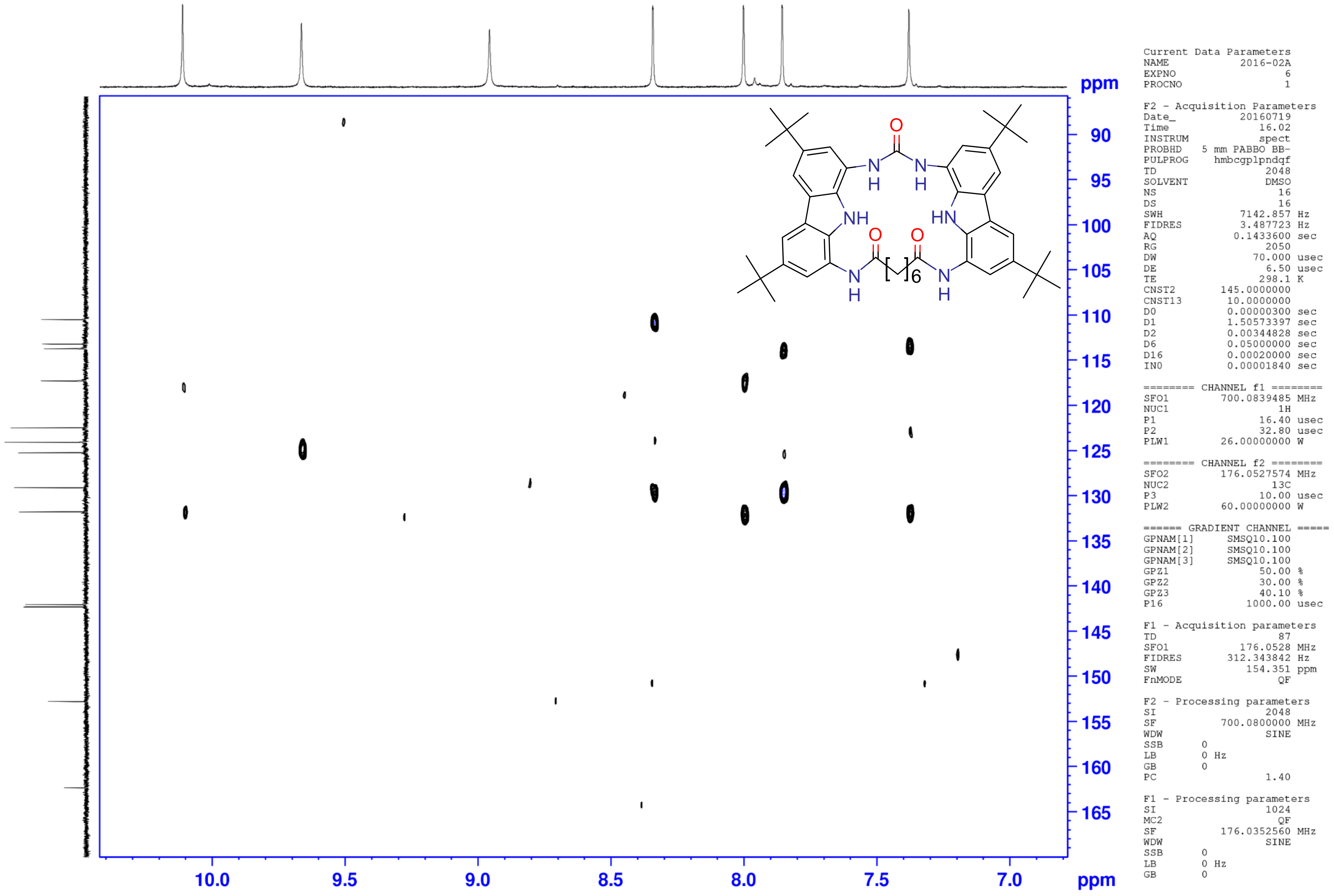

HRMS spectrum of compound **MC006**

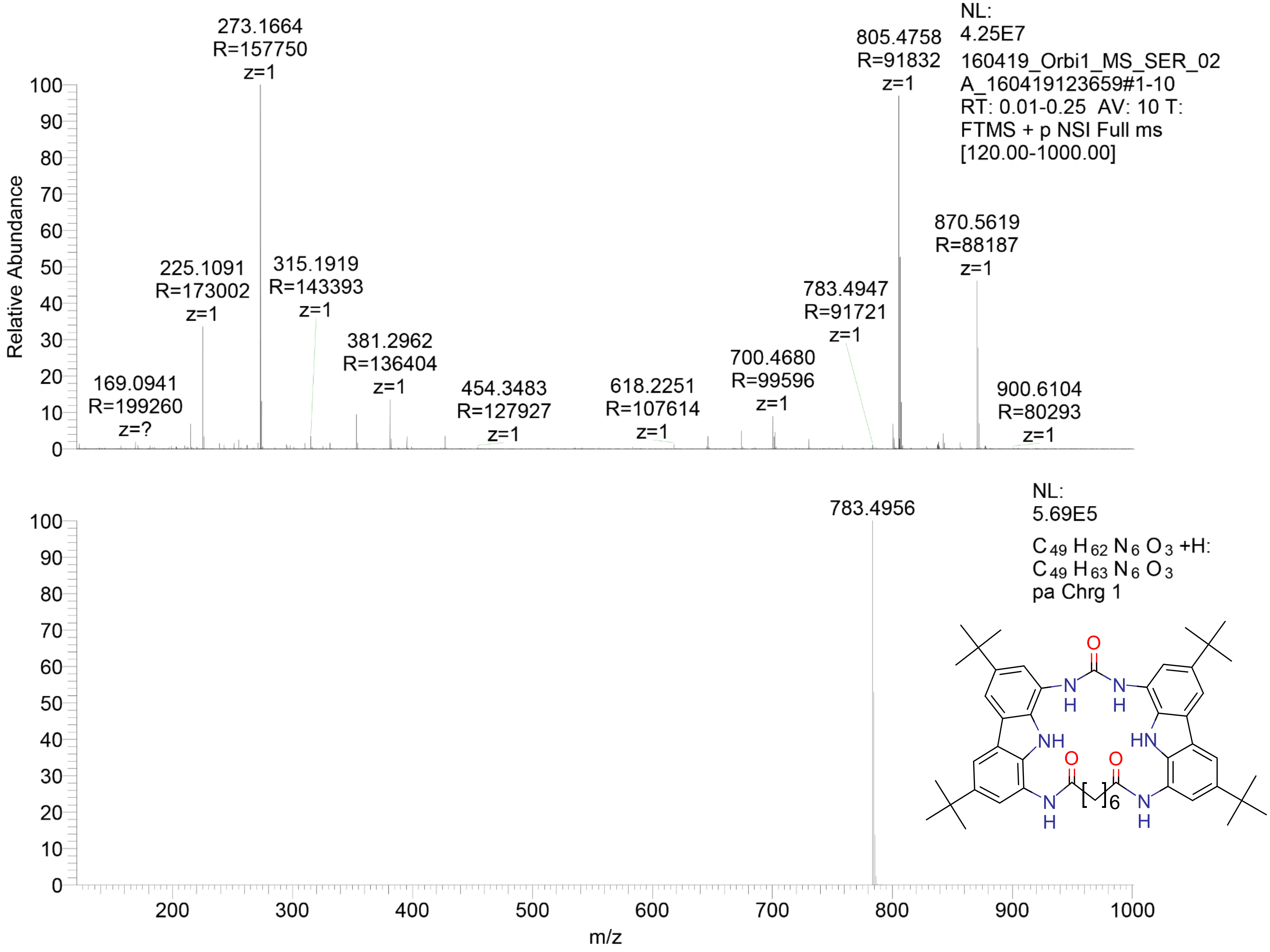

^{1}H NMR spectrum (700.1 MHz) of compound **MC007**

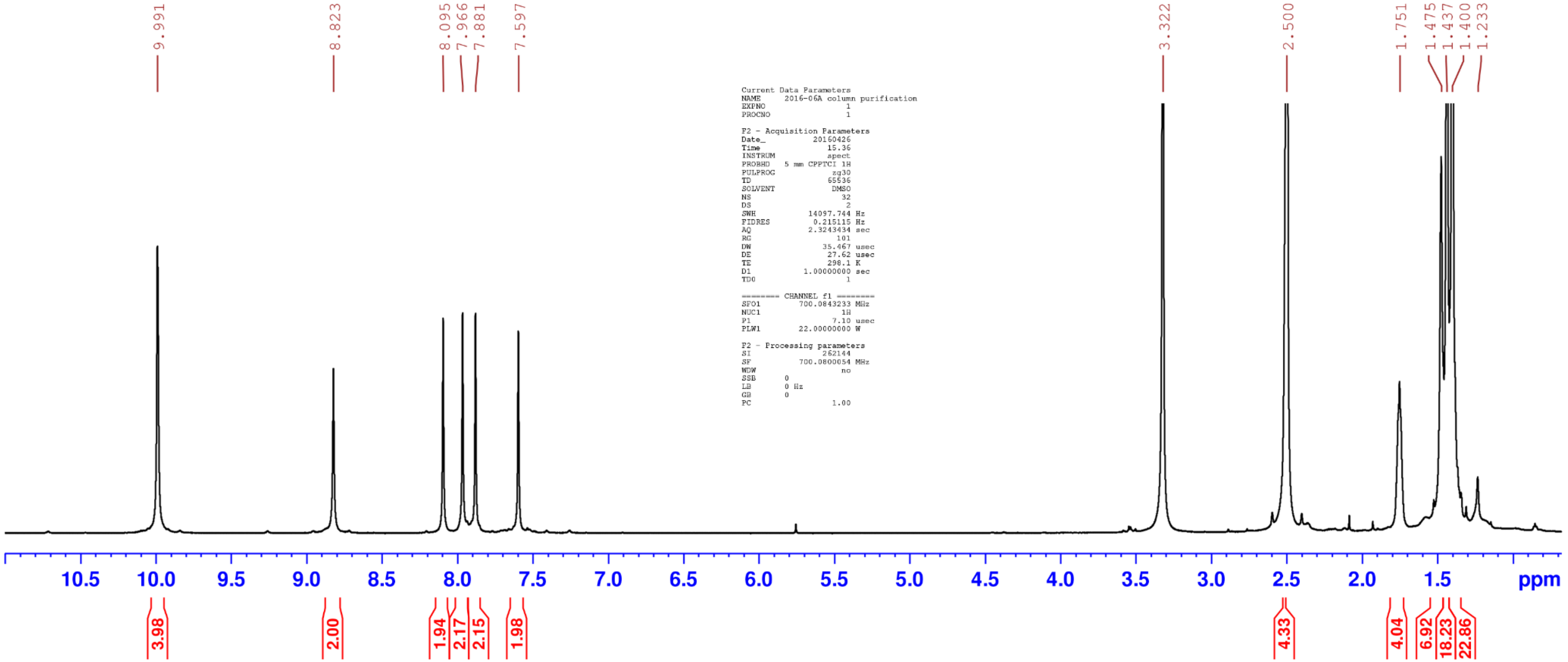

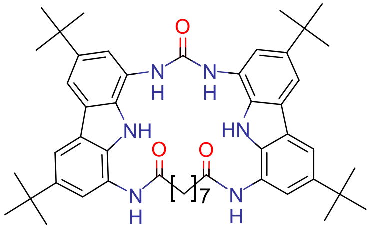

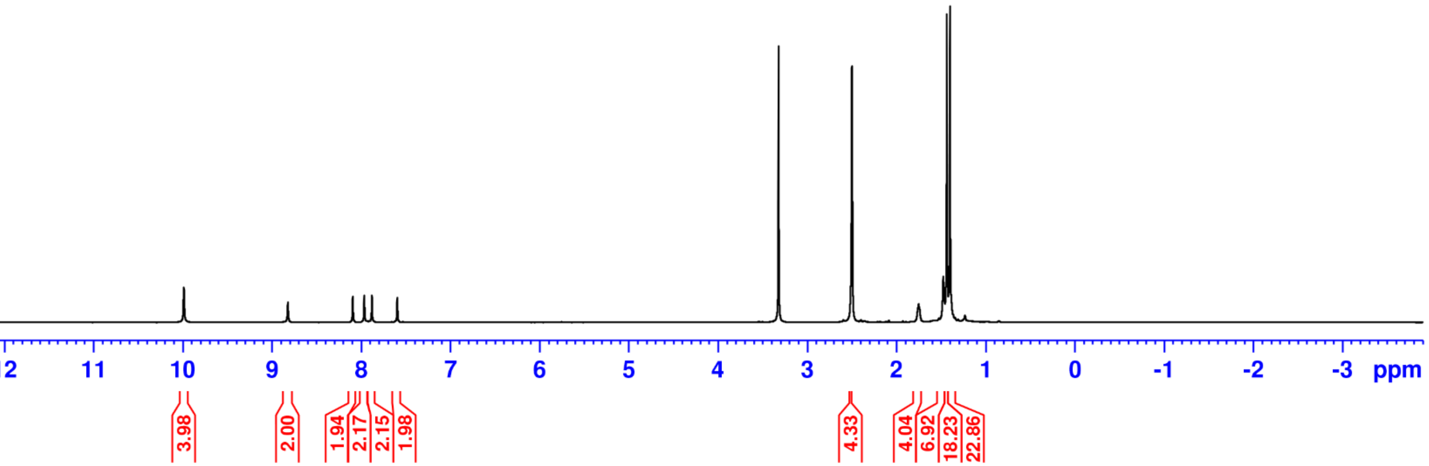

^{13}C NMR spectrum (700.1 MHz) of compound **MC007**

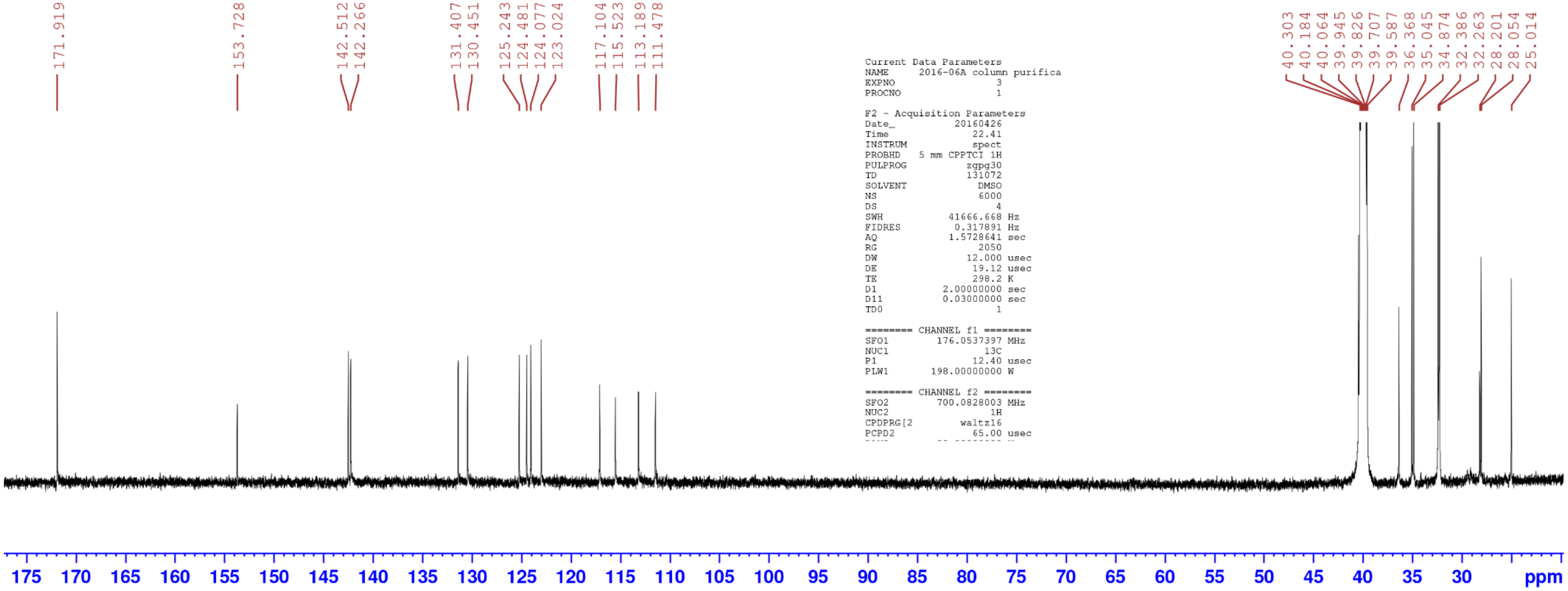

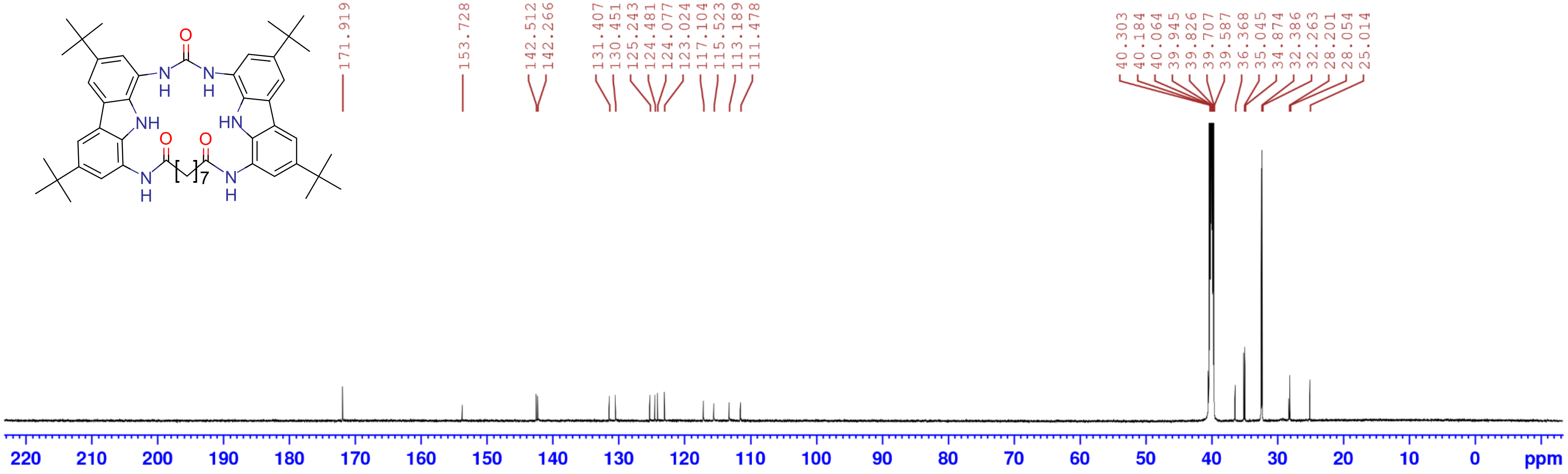

^{1}H-^{13}C HSQC spectrum (700.1 MHz) of compound **MC007**

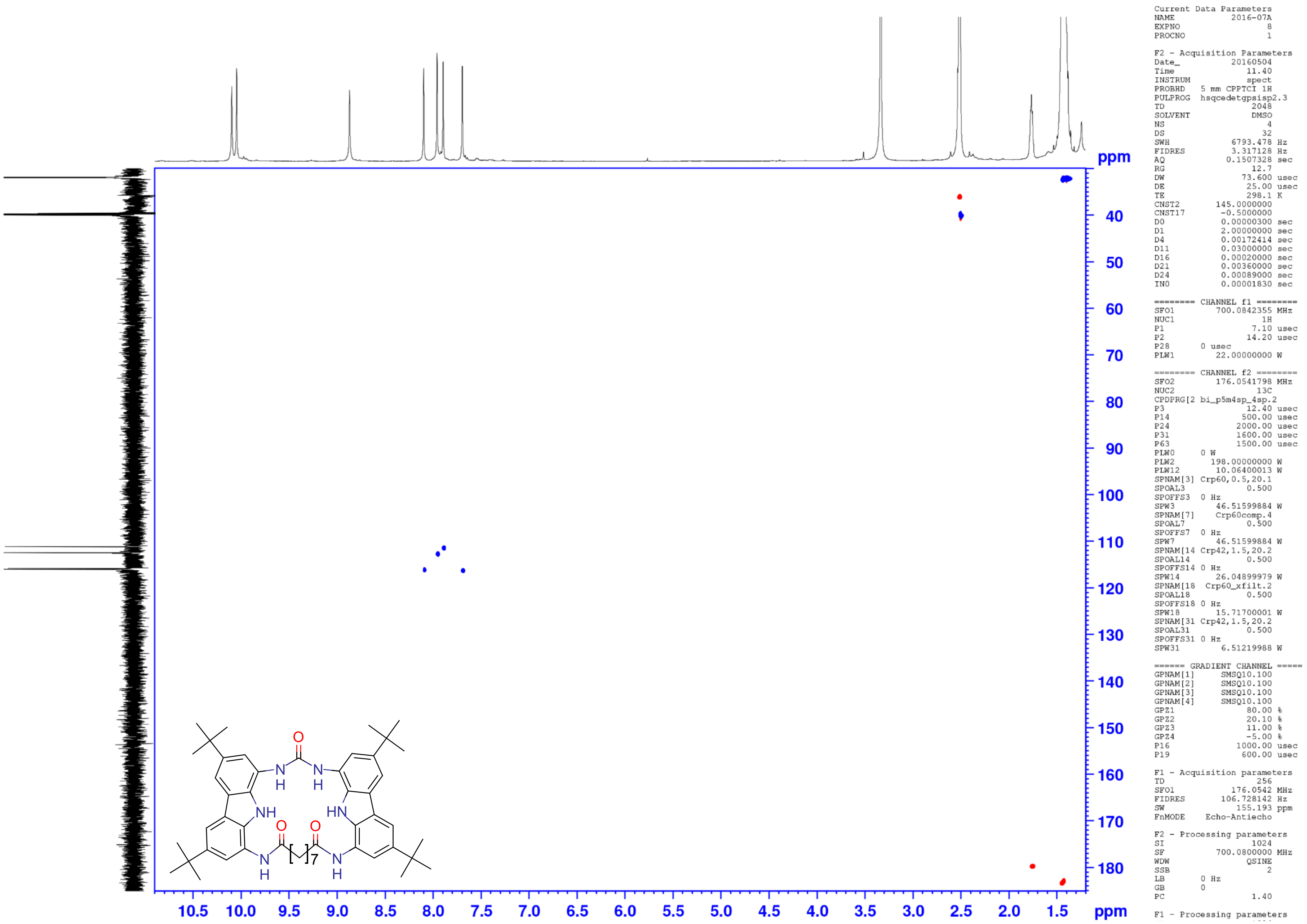

^{1}H-^{13}C HMBC spectrum (700.1 MHz) of compound **MC007**

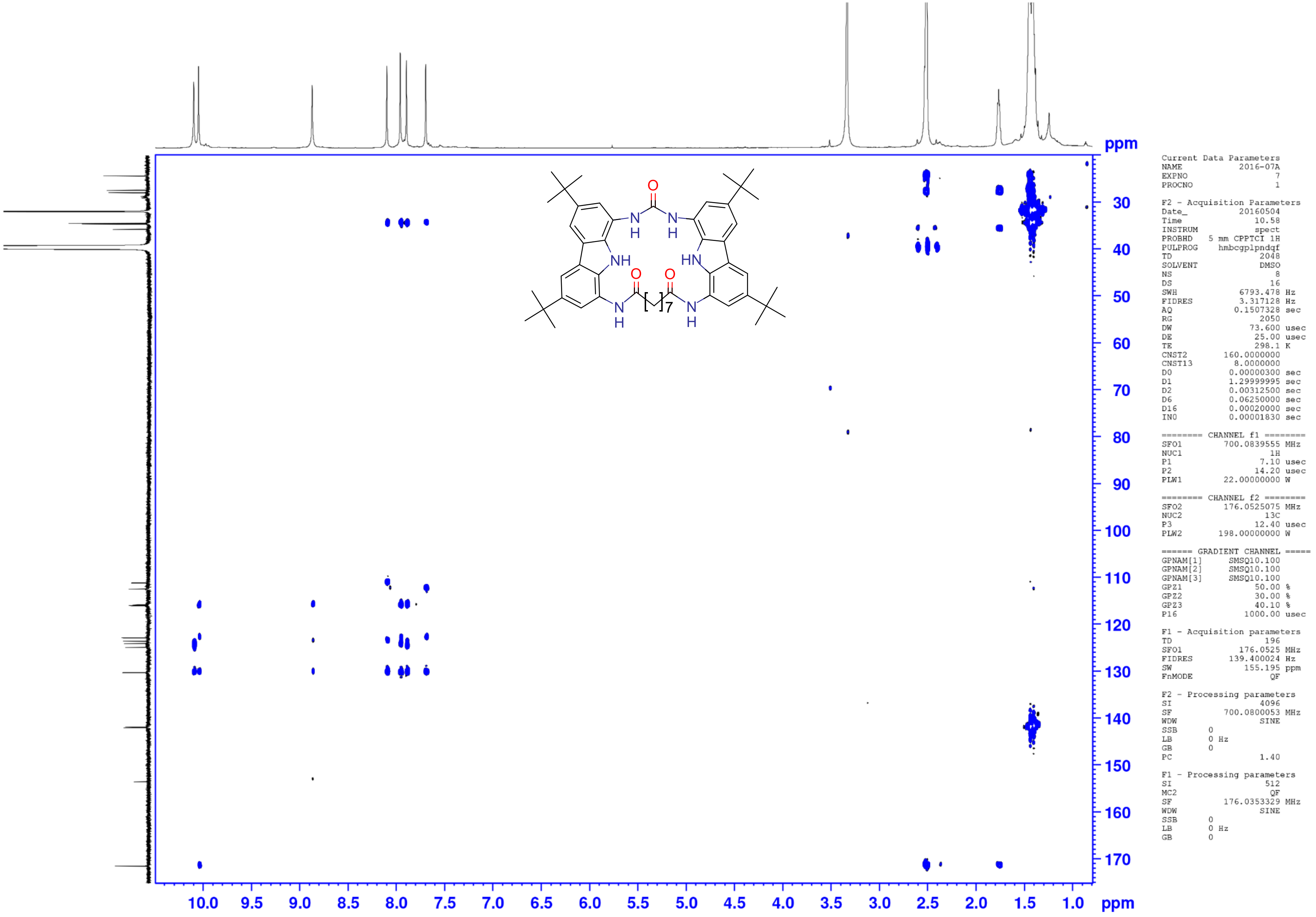

HRMS spectrum of compound **MC007**

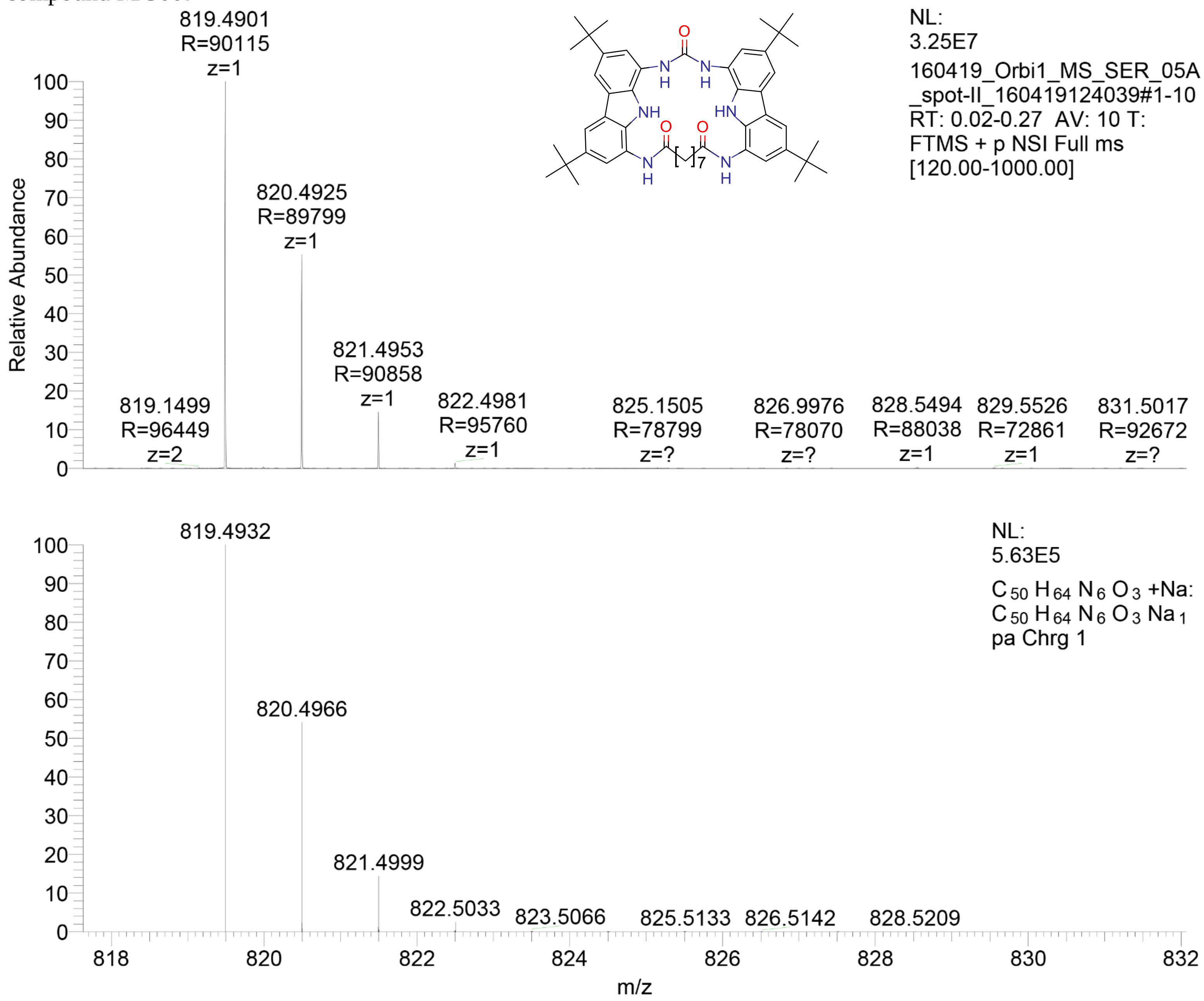

^{1}H NMR spectrum (700.1 MHz) of compound **MC008**

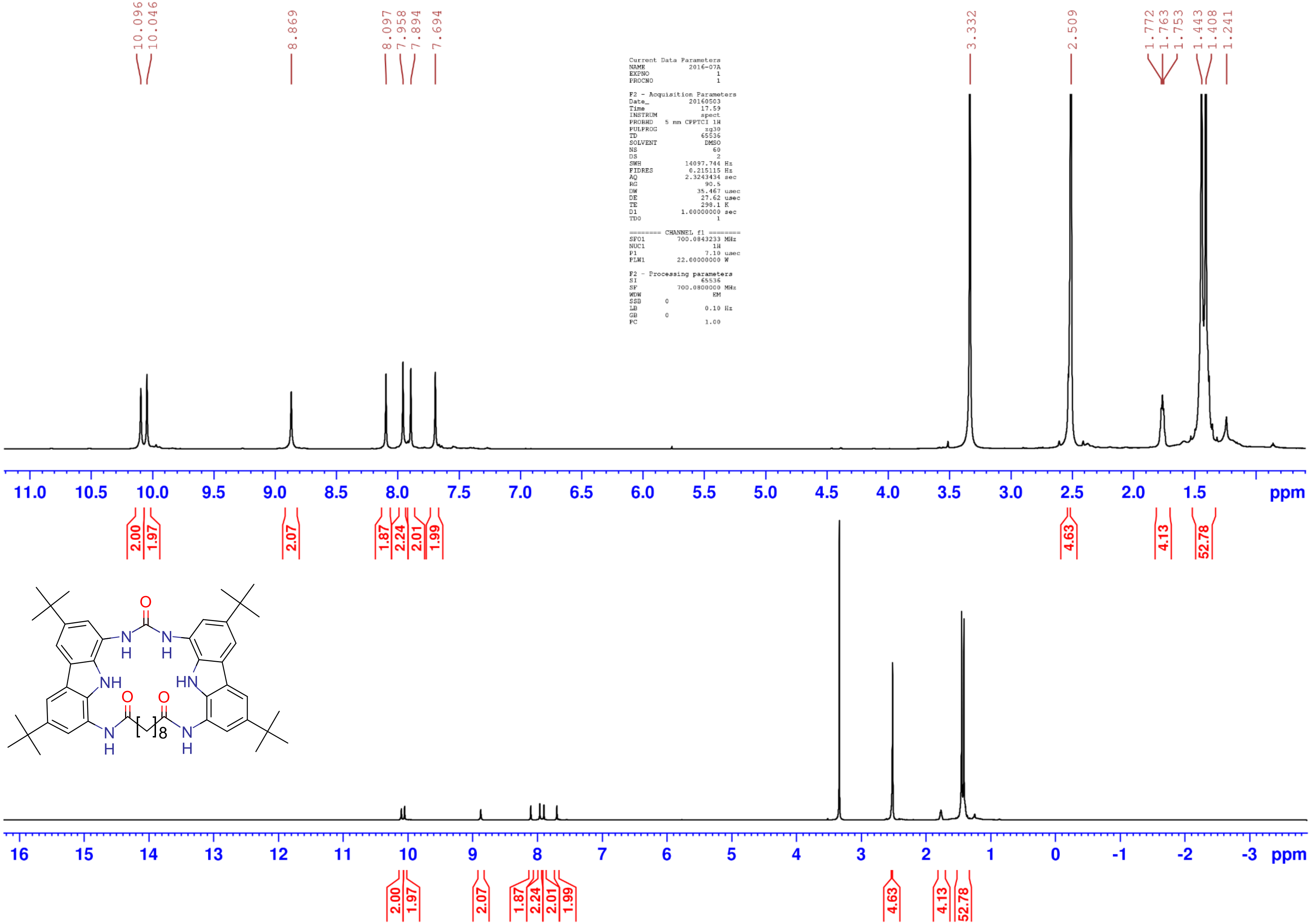

^{13}C NMR spectrum (700.1 MHz) of compound **MC008**

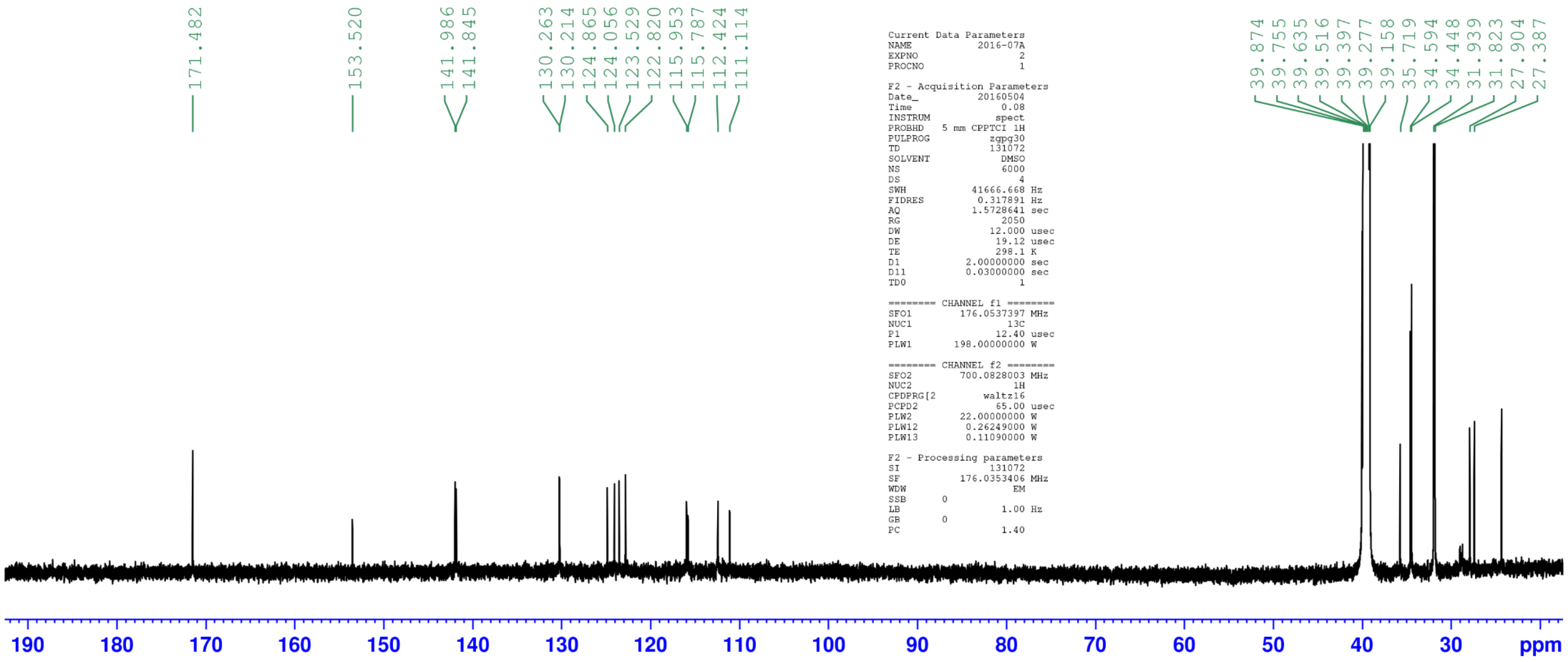

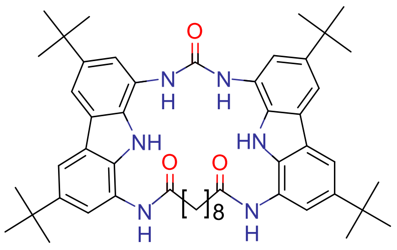

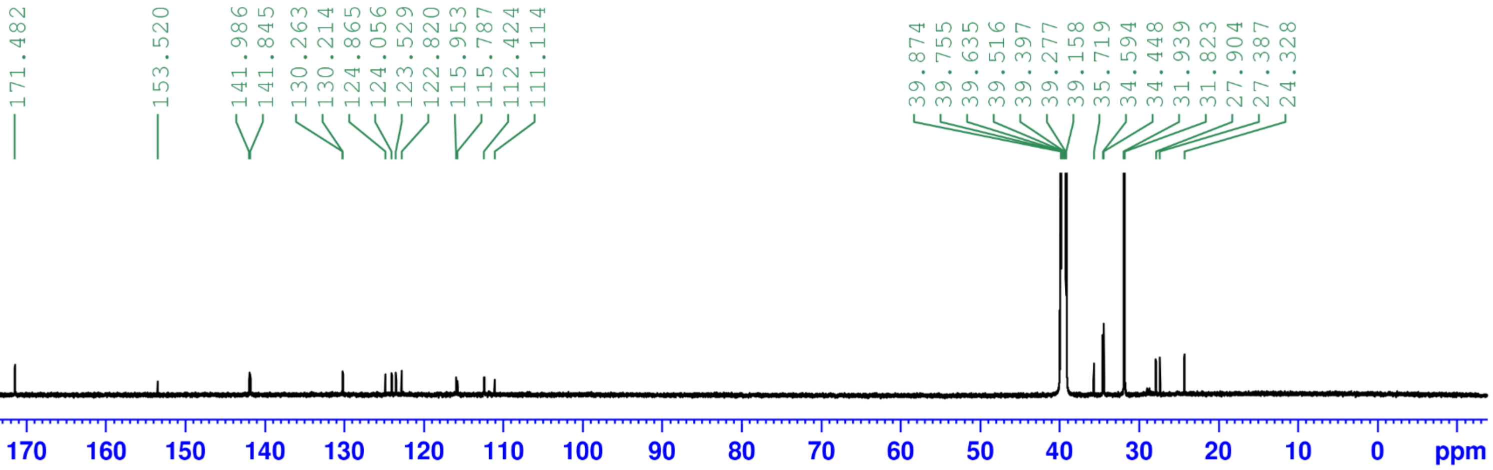

^{1}H-^{13}C HSQC spectrum (700.1 MHz) of compound **MC008**

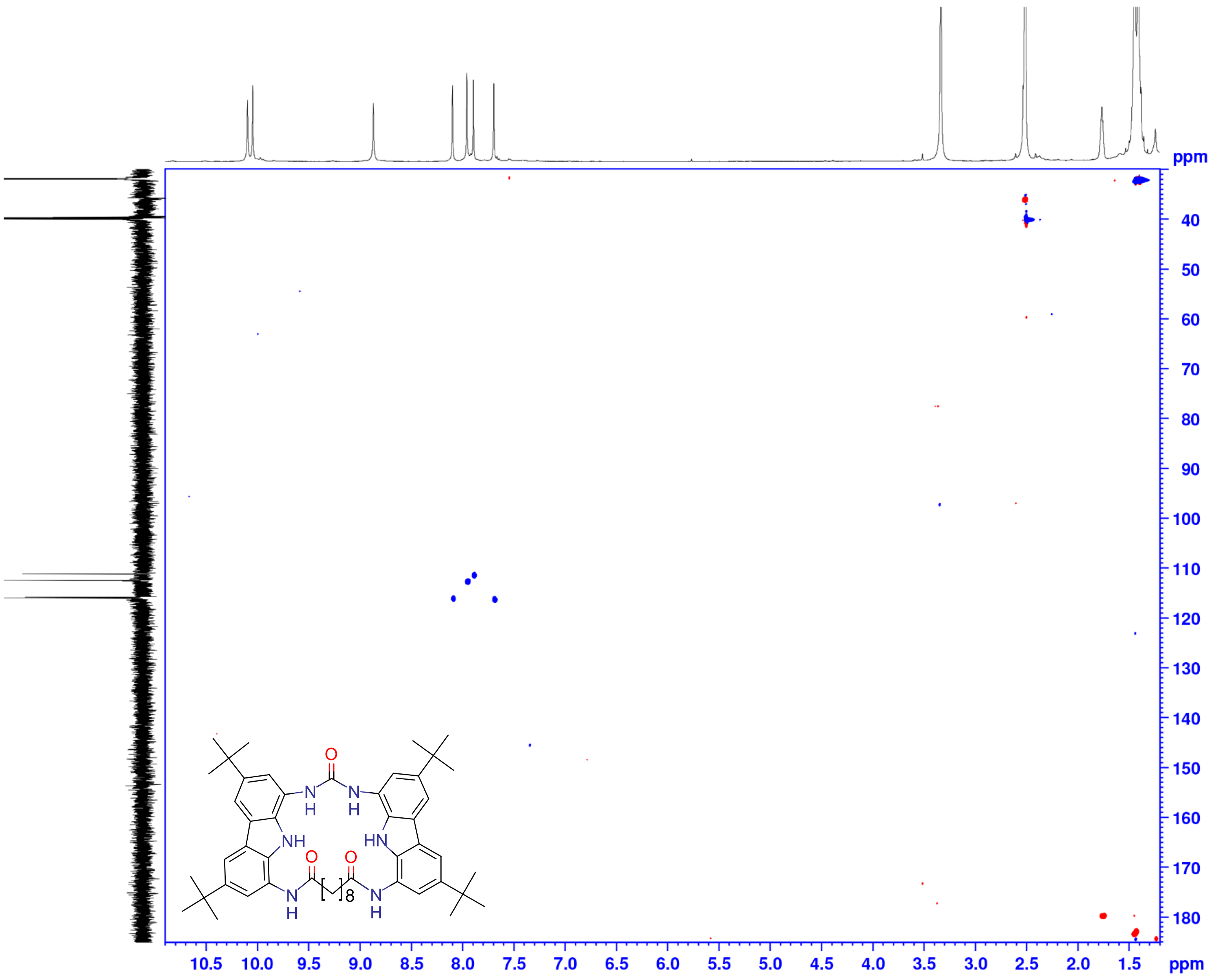


```
Current Data Parameters
NAME            2016-07A
EXPNO                  8
PROCNO                 1

F2 - Acquisition Parameters
Date_           20160504
Time               11.40
INSTRUM            spect
PROBHD   5 mm CPPTCI 1H
PULPROG  hsqcedetgpsisp2.3
TD                  2048
SOLVENT             DMSO
NS                     4
DS                    32
SWH             6793.478 Hz
FIDRES          3.317128 Hz
AQ             0.1507328 sec
RG                  12.7
DW                73.600 usec
DE                 25.00 usec
TE                 298.1 K
CNST2        145.0000000
CNST17        -0.5000000
D0            0.00000300 sec
D1            2.00000000 sec
D4            0.00172414 sec
D11           0.03000000 sec
D16           0.00020000 sec
D21           0.00360000 sec
D24           0.00089000 sec
IN0           0.00001830 sec

======== CHANNEL f1 ========
SFO1         700.0842355 MHz
NUC1                  1H
P1                  7.10 usec
P2                 14.20 usec
P28      0 usec
PLW1         22.00000000 W

======== CHANNEL f2 ========
SFO2         176.0541798 MHz
NUC2                 13C
CPDPRG[2 bi_p5m4sp_4sp.2
P3                 12.40 usec
P14               500.00 usec
P24              2000.00 usec
P31              1600.00 usec
P63              1500.00 usec
PLW0     0 W
PLW2        198.00000000 W
PLW12        10.06400013 W
SPNAM[3] Crp60,0.5,20.1
SPOAL3             0.500
SPOFFS3  0 Hz
SPW3         46.51599884 W
SPNAM[7]     Crp60comp.4
SPOAL7             0.500
SPOFFS7  0 Hz
SPW7         46.51599884 W
SPNAM[14 Crp42,1.5,20.2
SPOAL14            0.500
SPOFFS14 0 Hz
SPW14        26.04899979 W
SPNAM[18  Crp60_xfilt.2
SPOAL18            0.500
SPOFFS18 0 Hz
SPW18        15.71700001 W
SPNAM[31 Crp42,1.5,20.2
SPOAL31            0.500
SPOFFS31 0 Hz
SPW31         6.51219988 W

====== GRADIENT CHANNEL =====
GPNAM[1]      SMSQ10.100
GPNAM[2]      SMSQ10.100
GPNAM[3]      SMSQ10.100
GPNAM[4]      SMSQ10.100
GPZ1               80.00 %
GPZ2               20.10 %
GPZ3               11.00 %
GPZ4               -5.00 %
P16              1000.00 usec
P19               600.00 usec

F1 - Acquisition parameters
TD                   256
SFO1            176.0542 MHz
FIDRES        106.728142 Hz
SW               155.193 ppm
FnMODE     Echo-Antiecho

F2 - Processing parameters
SI                  1024
SF           700.0800000 MHz
WDW                QSINE
SSB                    2
LB       0 Hz
GB       0
PC                  1.40

F1 - Processing parameters
```

^{1}H-^{13}C HMBC spectrum (700.1 MHz) of compound **MC008**

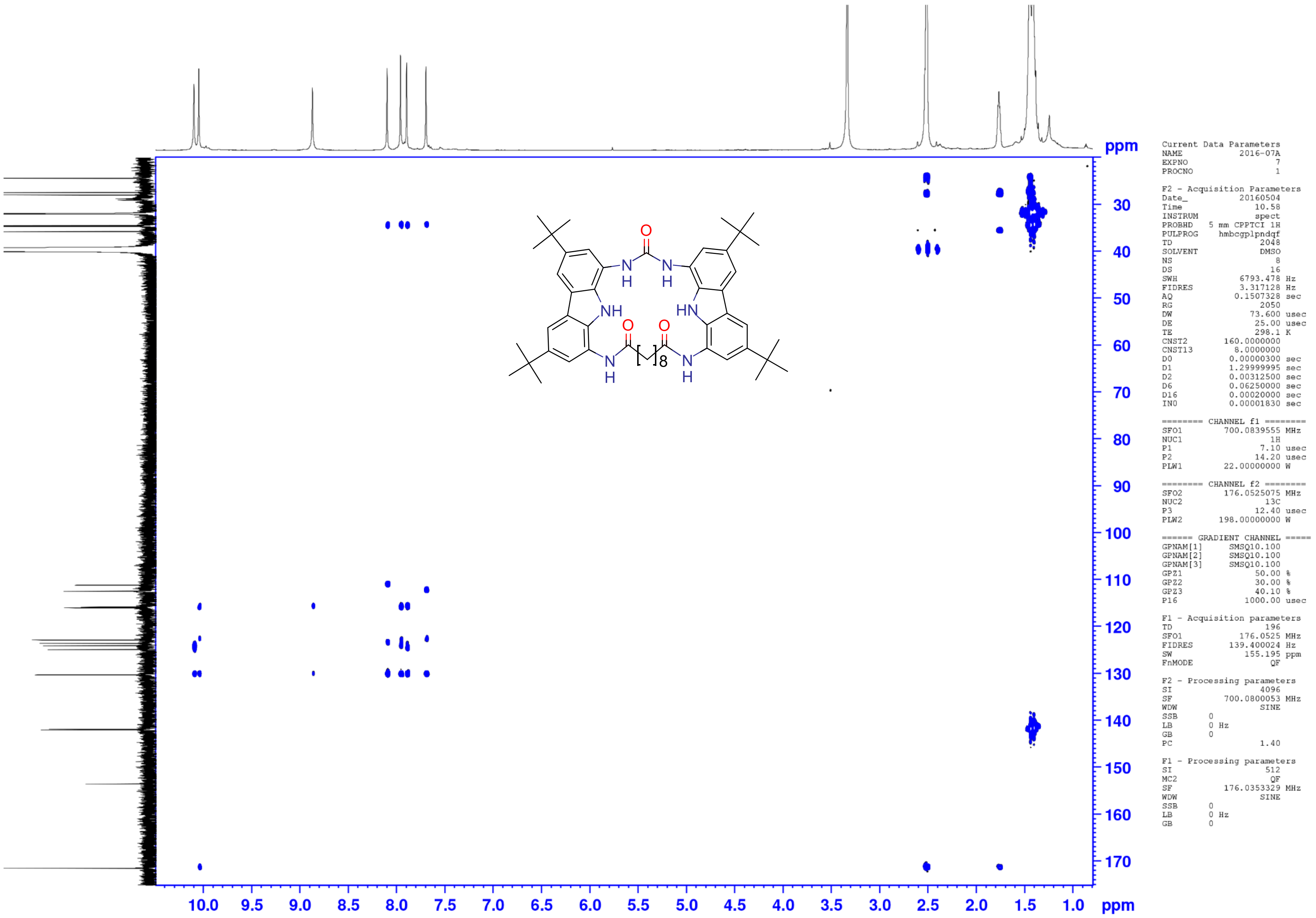

HRMS spectrum of compound **MC008**

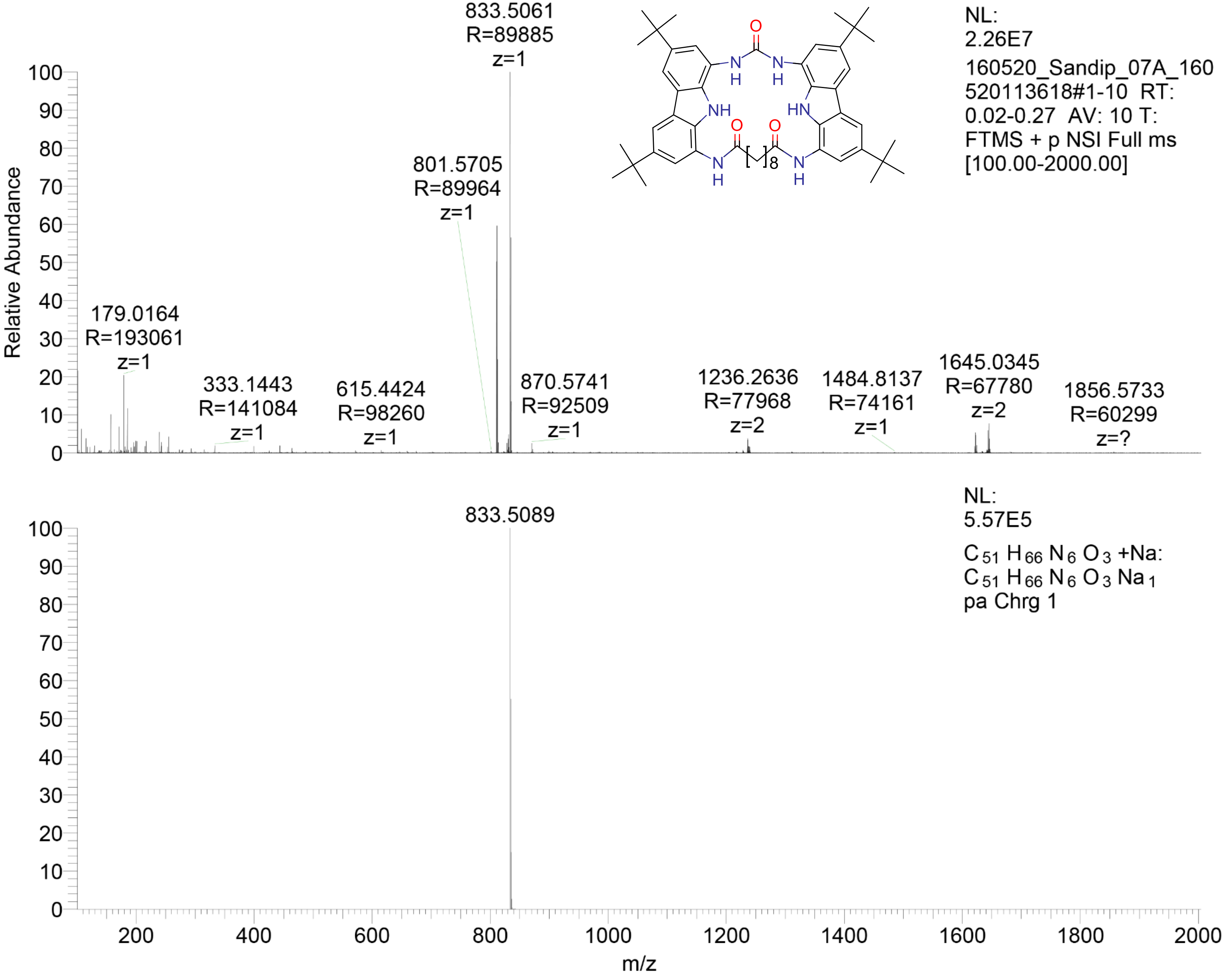

^{1}H NMR spectrum (700.1 MHz) of compound **MC009**

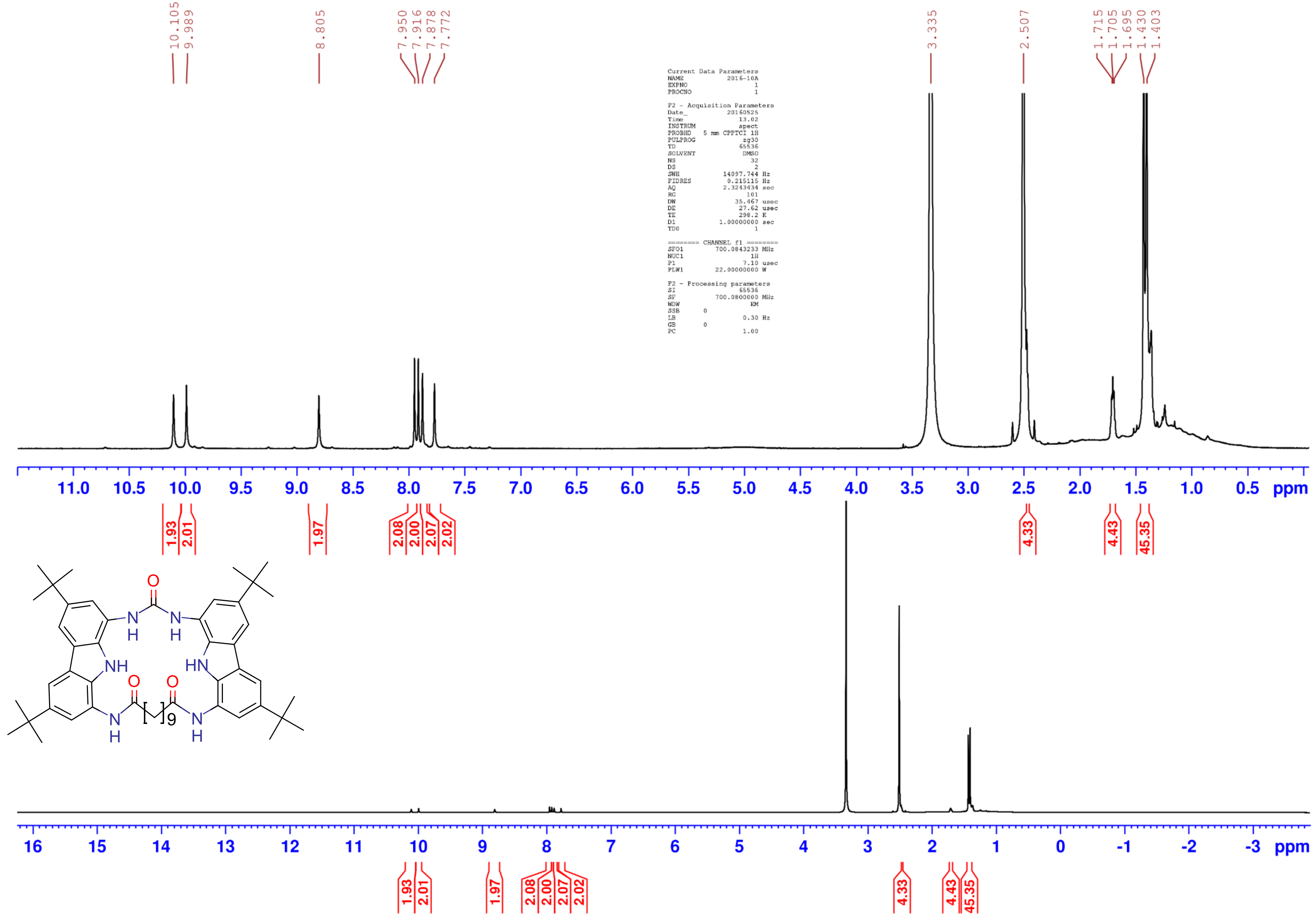

^{13}C NMR spectrum (700.1 MHz) of compound **MC009**

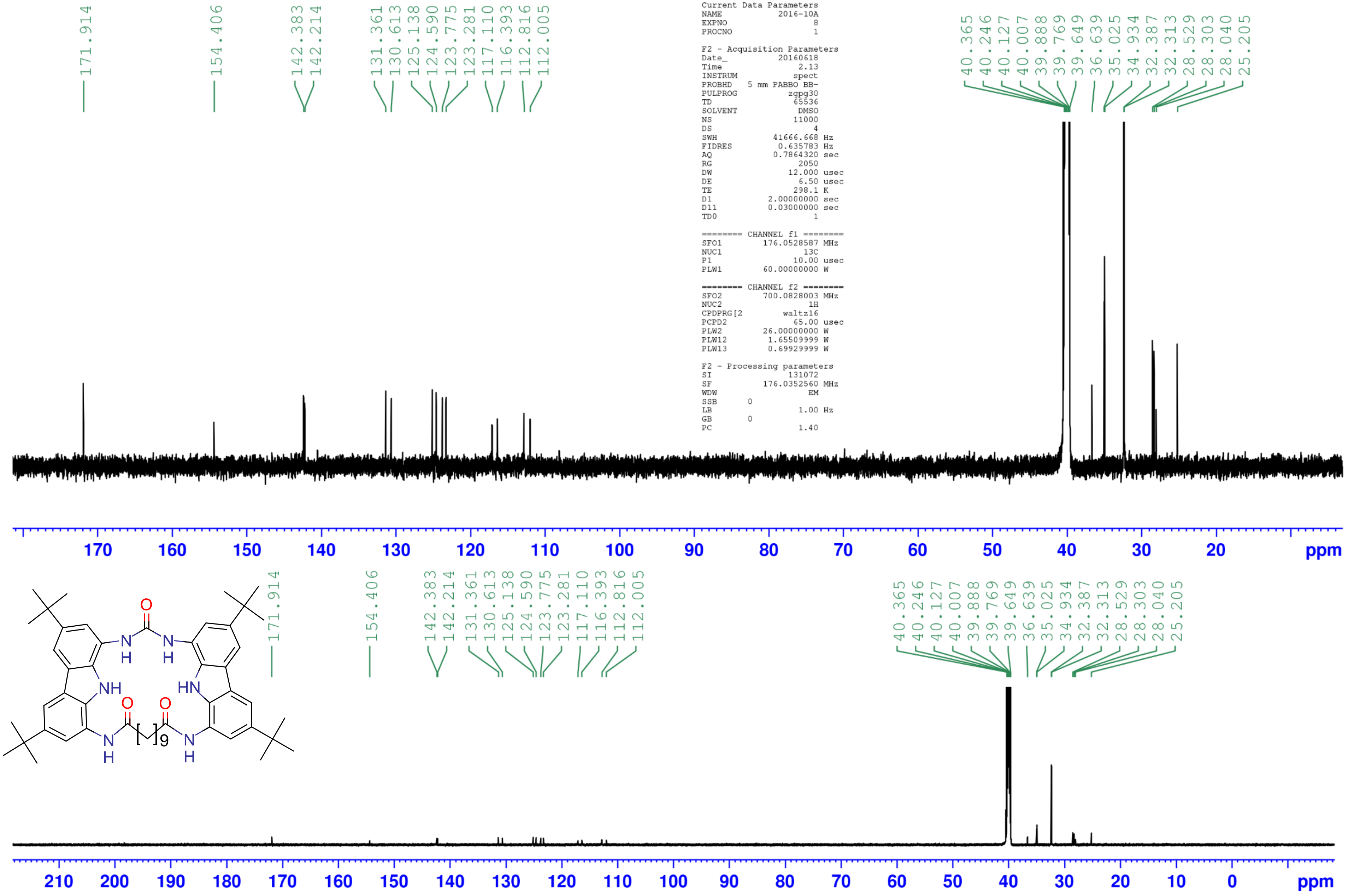

^{1}H-^{13}C HSQC spectrum (700.1 MHz) of compound **MC009**

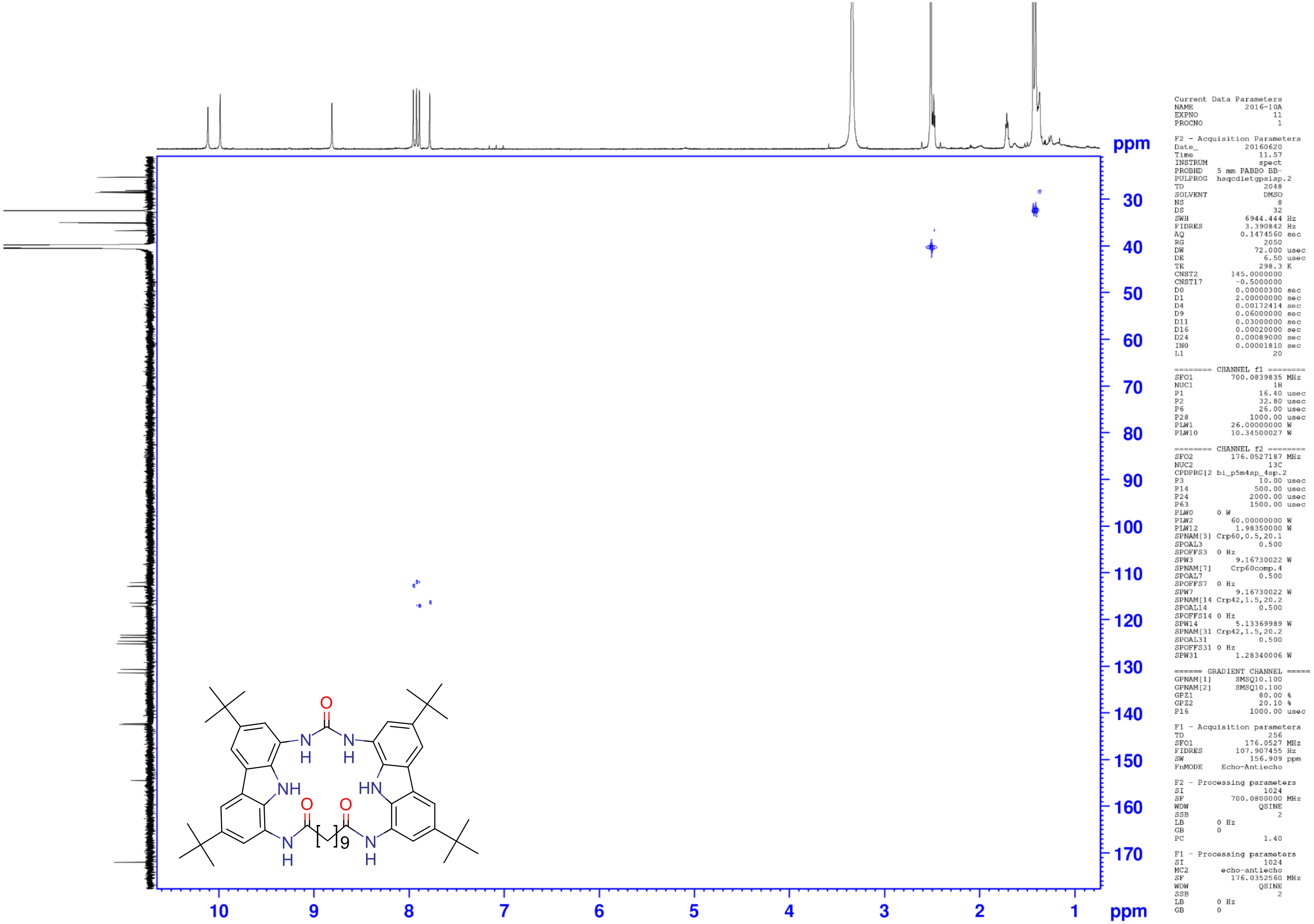

^{1}H-^{13}C HMBC spectrum (700.1 MHz) of compound **MC009**

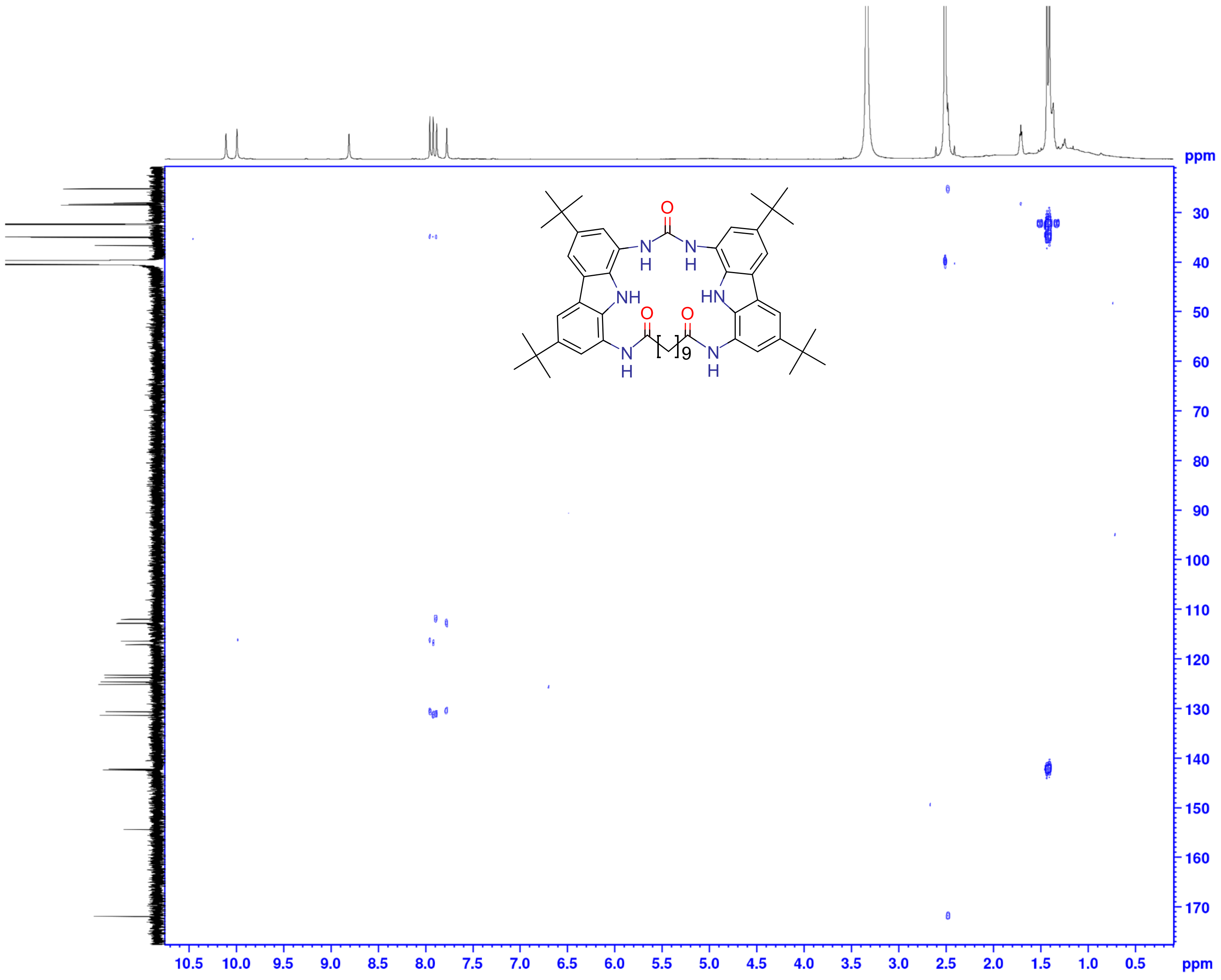


```
Current Data Parameters
NAME            2016-10A
EXPNO                 12
PROCNO                 1

F2 - Acquisition Parameters
Date_           20160620
Time               13.15
INSTRUM            spect
PROBHD    5 mm PABBO BB-
PULPROG    hmbcgplpndqf
TD                  2048
SOLVENT             DMSO
NS                     8
DS                    16
SWH             7462.687 Hz
FIDRES          3.643890 Hz
AQ             0.1372160 sec
RG                  2050
DW                67.000 usec
DE                  6.50 usec
TE                 298.1 K
CNST2        145.0000000
CNST13        10.0000000
D0            0.00000300 sec
D1            1.51023996 sec
D2            0.00344828 sec
D6            0.05000000 sec
D16           0.00020000 sec
IN0           0.00001810 sec

======== CHANNEL f1 ========
SFO1         700.0837974 MHz
NUC1                  1H
P1                 16.40 usec
P2                 32.80 usec
PLW1         26.00000000 W

======== CHANNEL f2 ========
SFO2         176.0527187 MHz
NUC2                 13C
P3                 10.00 usec
PLW2         60.00000000 W

====== GRADIENT CHANNEL =====
GPNAM[1]       SMSQ10.100
GPNAM[2]       SMSQ10.100
GPNAM[3]       SMSQ10.100
GPZ1                50.00 %
GPZ2                30.00 %
GPZ3                40.10 %
P16               1000.00 usec

F1 - Acquisition parameters
TD                   128
SFO1            176.0527 MHz
FIDRES        215.814911 Hz
SW               156.909 ppm
FnMODE                QF

F2 - Processing parameters
SI                  2048
SF           700.0800000 MHz
WDW                 SINE
SSB      0
LB       0 Hz
GB       0
PC                  1.40

F1 - Processing parameters
SI                  1024
MC2                   QF
SF           176.0352560 MHz
WDW                 SINE
SSB      0
LB       0 Hz
GB       0
```

HRMS spectrum of compound **MC009**

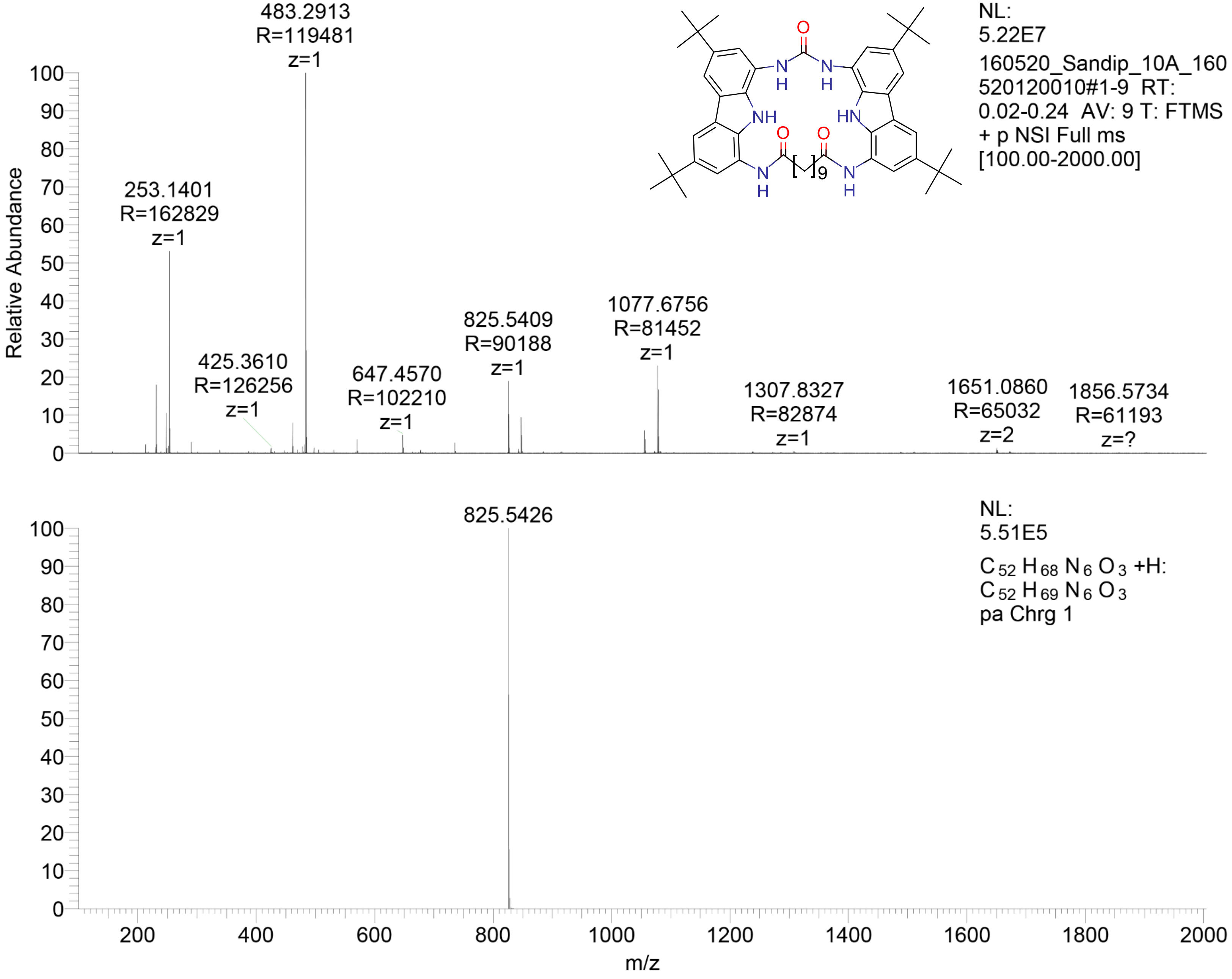

^{1}H NMR spectrum (700.1 MHz) of compound **MC010**

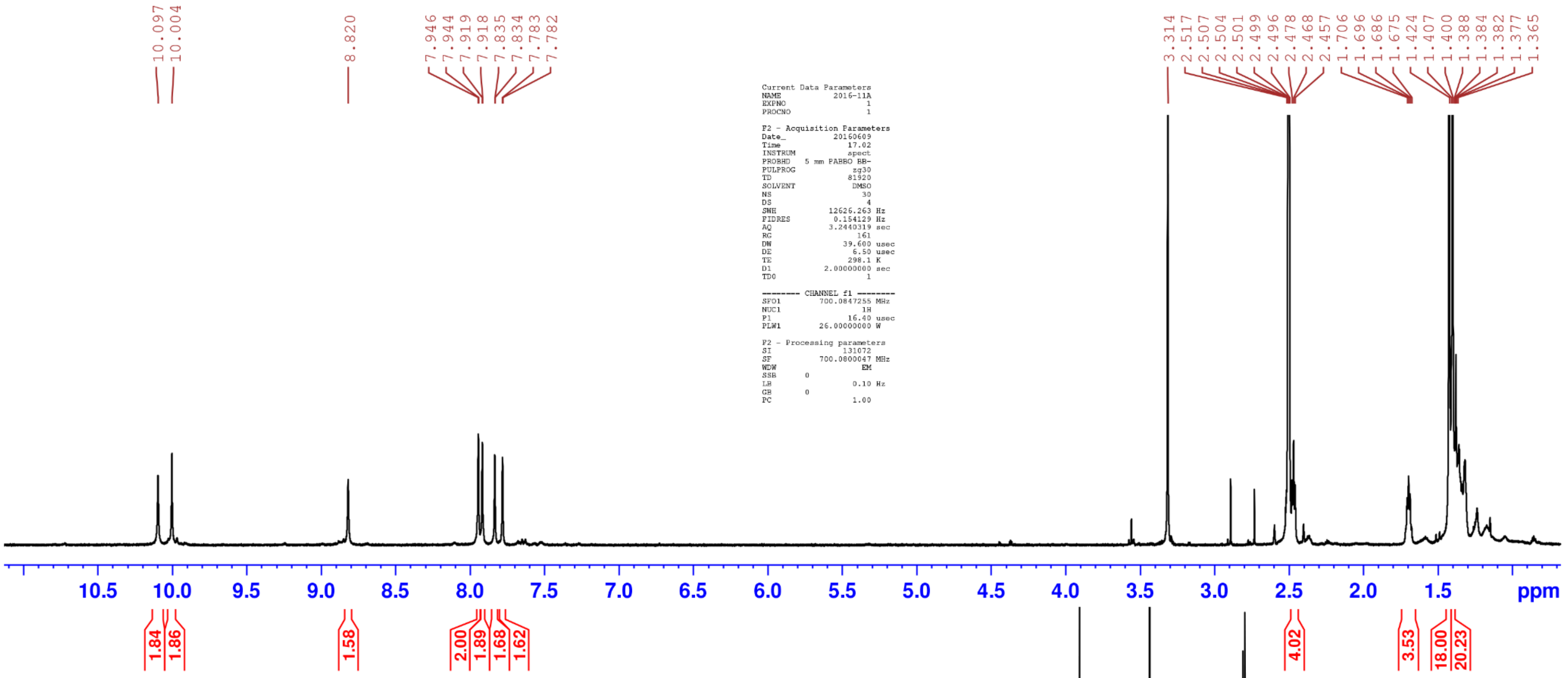

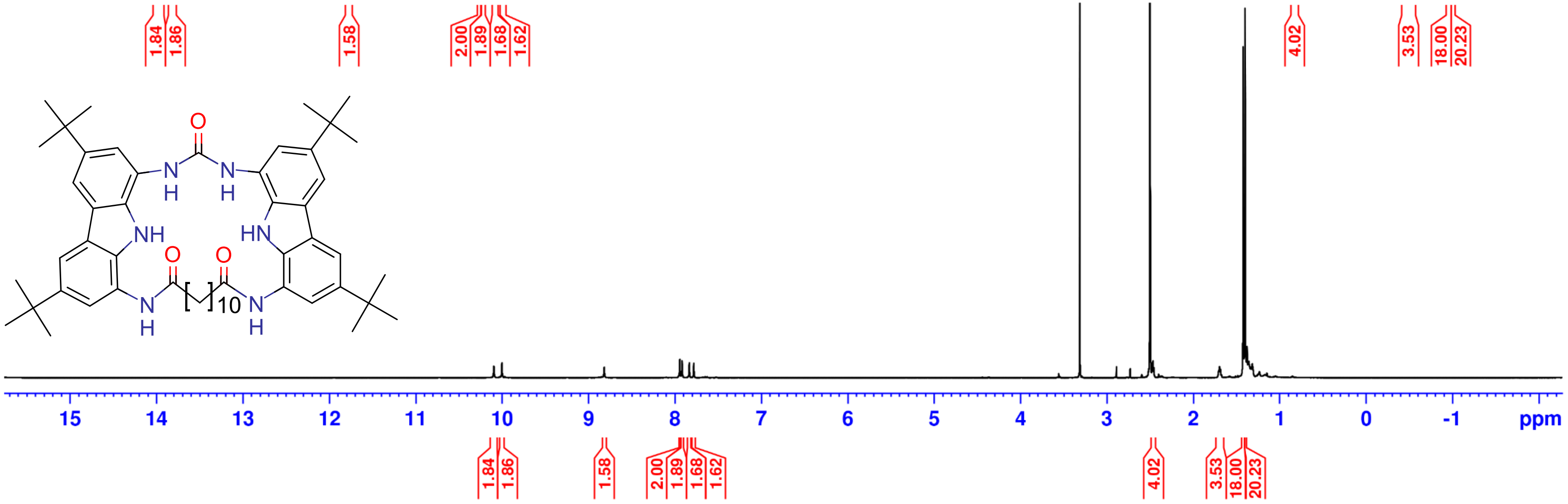

^{13}C NMR spectrum (700.1 MHz) of compound **MC010**

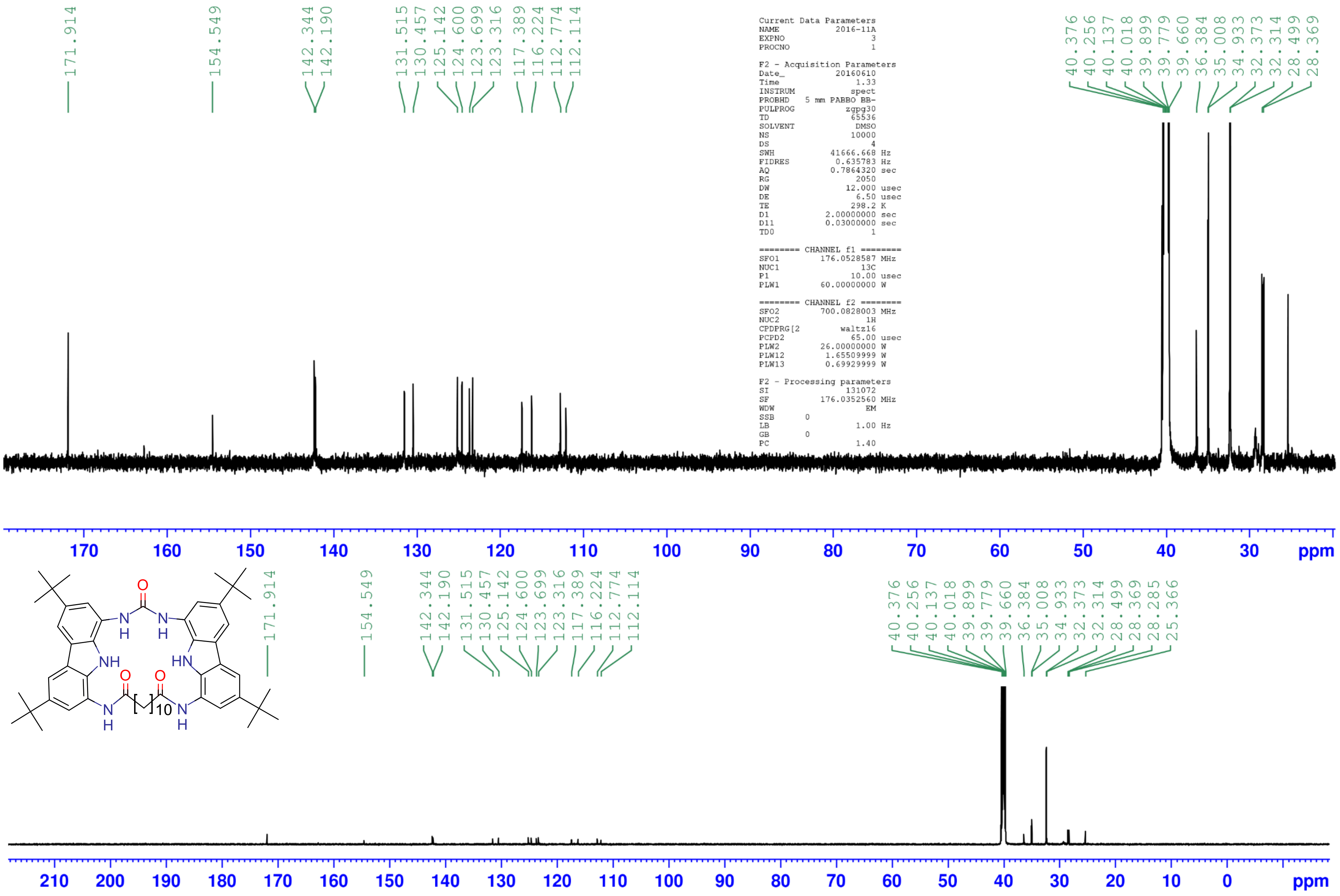

^{1}H-^{13}C HSQC spectrum (700.1 MHz) of compound **MC010**

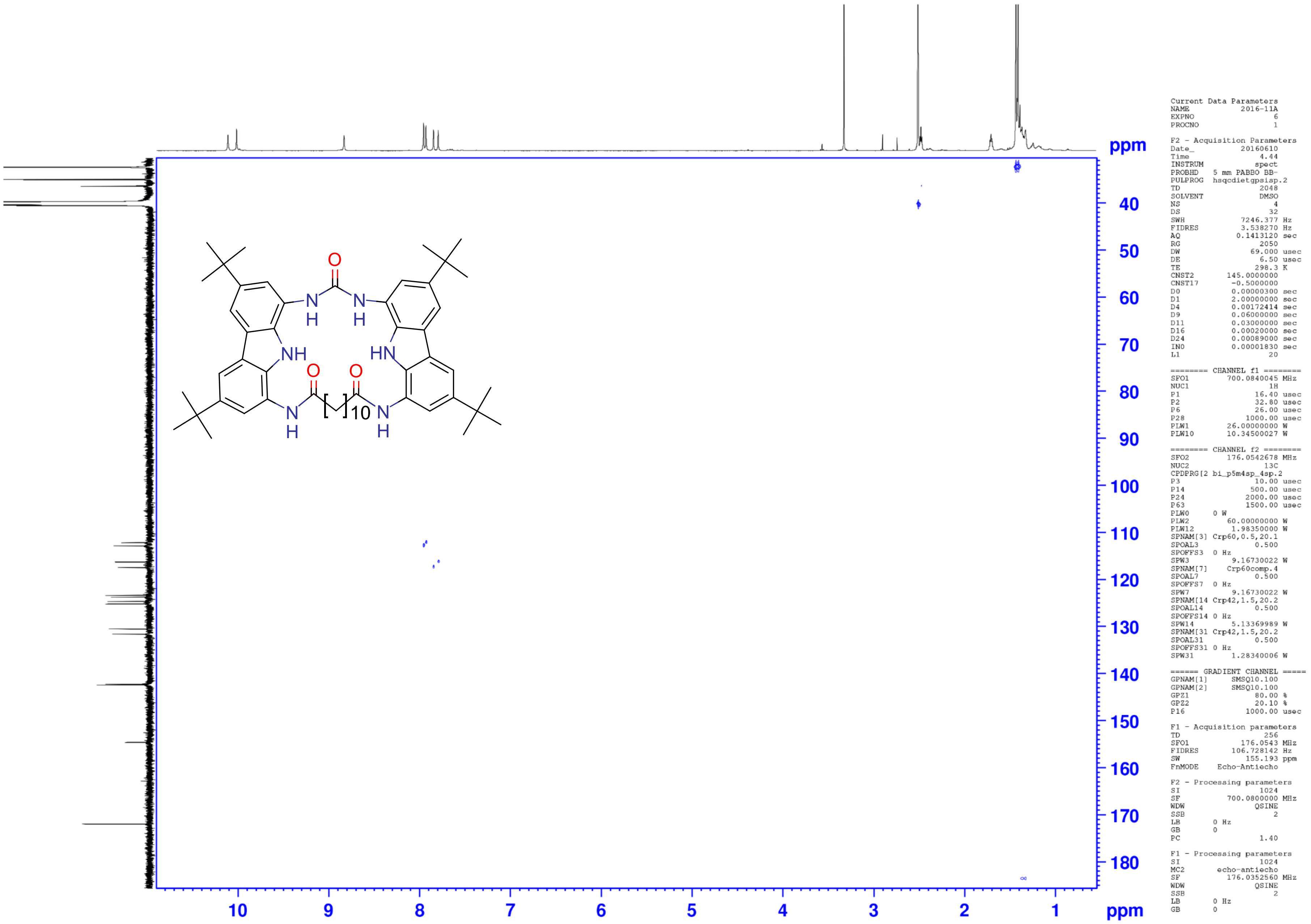

^{1}H-^{13}C HMBC spectrum (700.1 MHz) of compound **MC010**

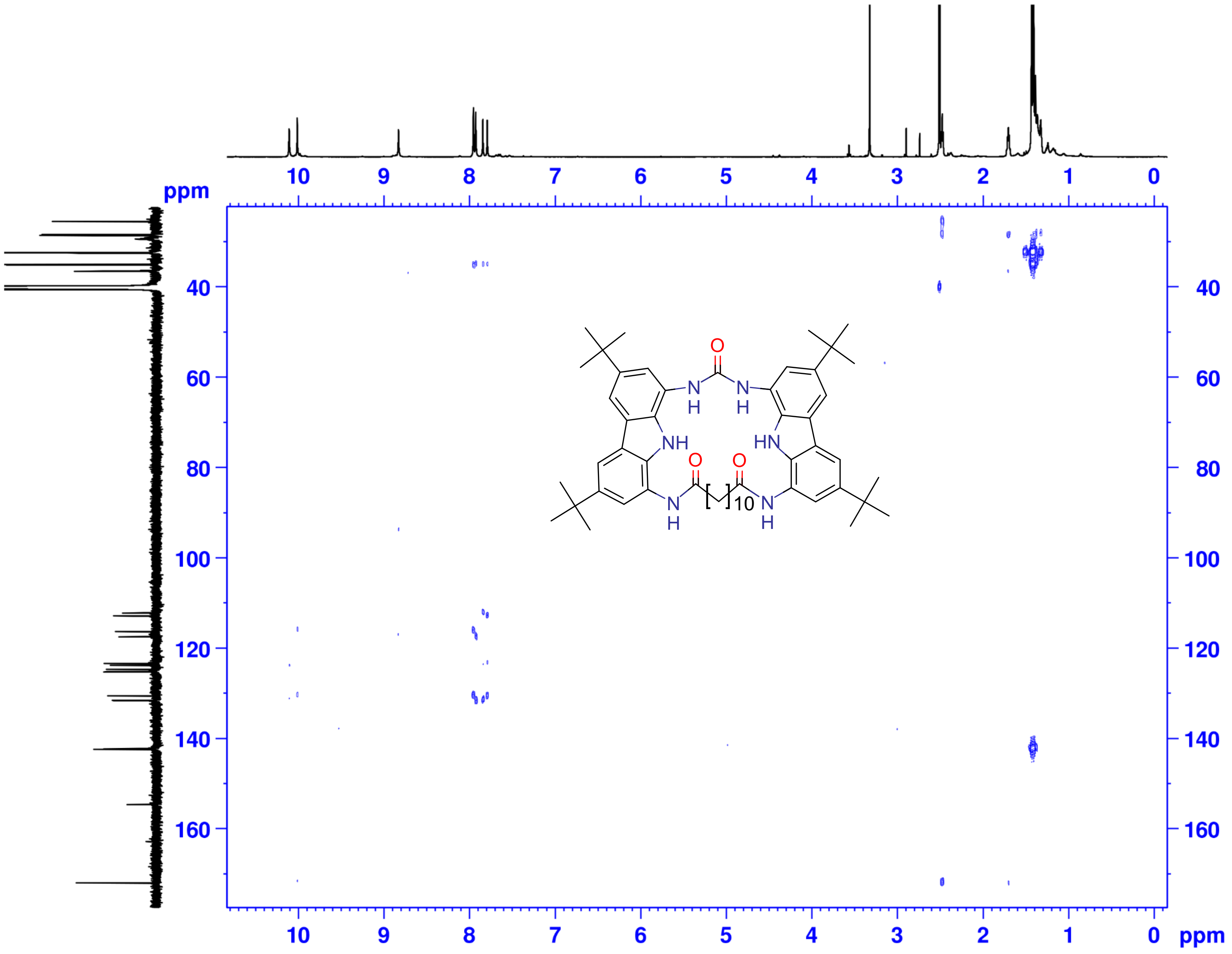


```
Current Data Parameters
NAME             2016-11A
EXPNO                   7
PROCNO                  1

F2 - Acquisition Parameters
Date_            20160610
Time                 5.23
INSTRUM             spect
PROBHD   5 mm PABBO BB-
PULPROG    hmbcgplpndqf
TD                   2048
SOLVENT              DMSO
NS                      8
DS                     16
SWH              7692.308 Hz
FIDRES           3.756010 Hz
AQ              0.1331200 sec
RG                   2050
DW                 65.000 usec
DE                   6.50 usec
TE                  298.1 K
CNST2         145.0000000
CNST13         10.0000000
D0             0.00000300 sec
D1             1.50819194 sec
D2             0.00344828 sec
D6             0.05000000 sec
D16            0.00020000 sec
IN0            0.00001830 sec

======== CHANNEL f1 ========
SFO1          700.0837396 MHz
NUC1                   1H
P1                  16.40 usec
P2                  32.80 usec
PLW1          26.00000000 W

======== CHANNEL f2 ========
SFO2          176.0528307 MHz
NUC2                  13C
P3                  10.00 usec
PLW2          60.00000000 W

====== GRADIENT CHANNEL =====
GPNAM[1]       SMSQ10.100
GPNAM[2]       SMSQ10.100
GPNAM[3]       SMSQ10.100
GPZ1                50.00 %
GPZ2                30.00 %
GPZ3                40.10 %
P16               1000.00 usec

F1 - Acquisition parameters
TD                    128
SFO1             176.0528 MHz
FIDRES         213.456284 Hz
SW                155.194 ppm
FnMODE                 QF

F2 - Processing parameters
SI                   2048
SF            700.0800000 MHz
WDW                  SINE
SSB      0
LB       0 Hz
GB       0
PC                   1.40

F1 - Processing parameters
SI                   1024
MC2                    QF
SF            176.0352560 MHz
WDW                  SINE
SSB      0
LB       0 Hz
GB       0
```

HRMS spectrum of compound **MC010**

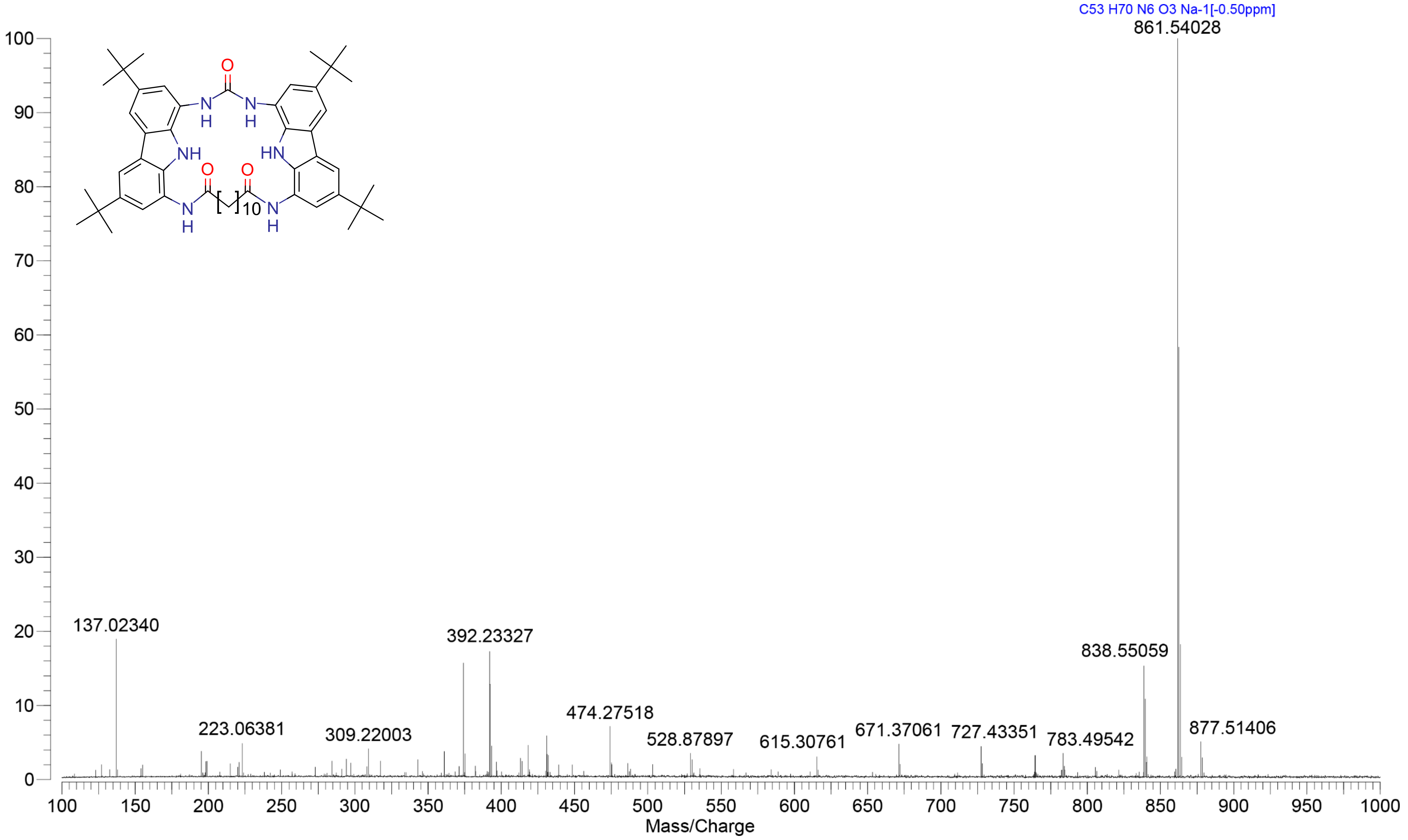

^{1}H NMR spectrum (700.1 MHz) of compound **MC011**

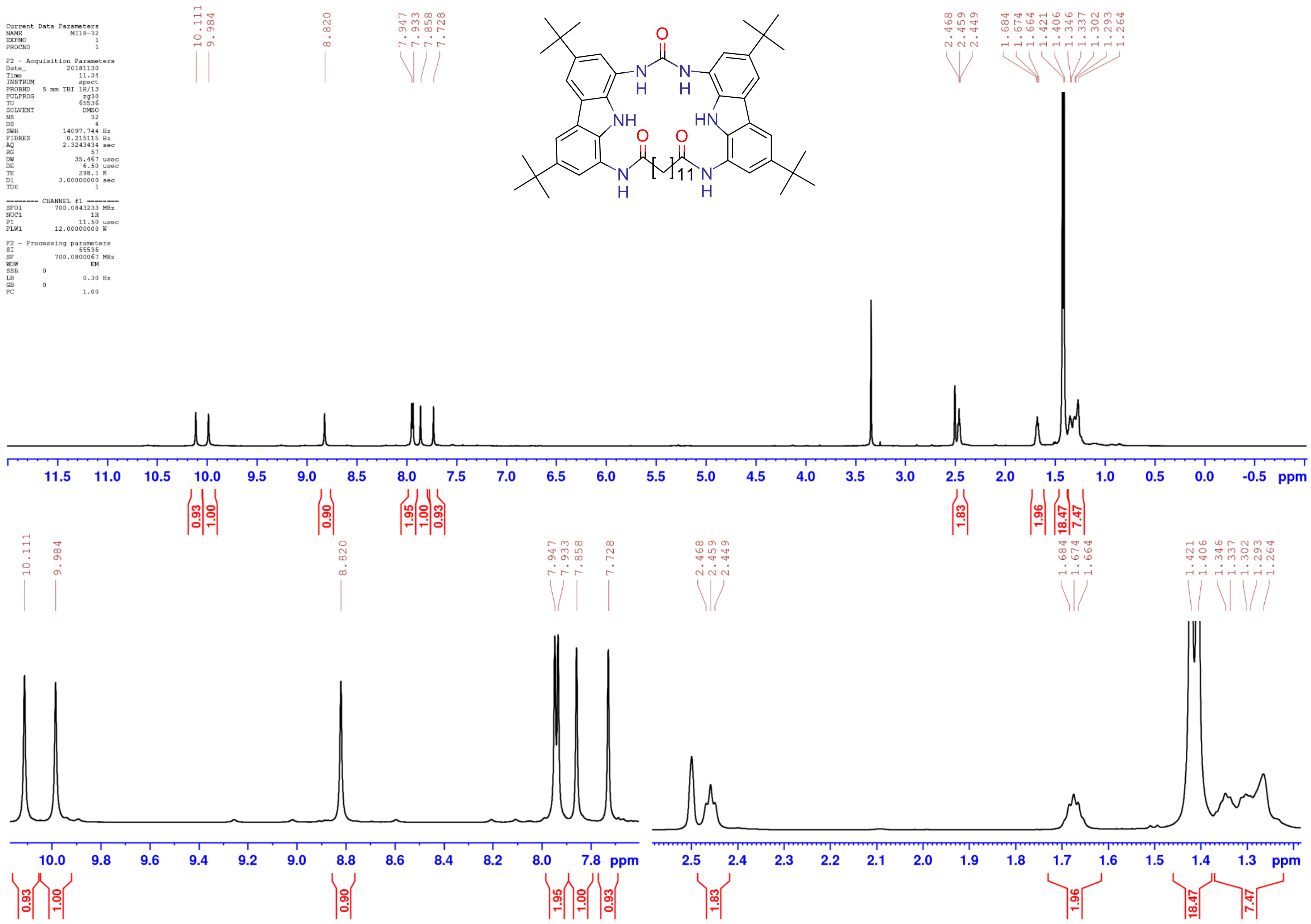

^{13}C NMR spectrum (700.1 MHz) of compound **MC011**

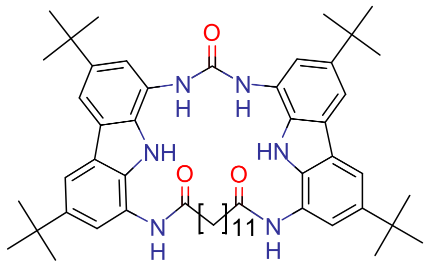

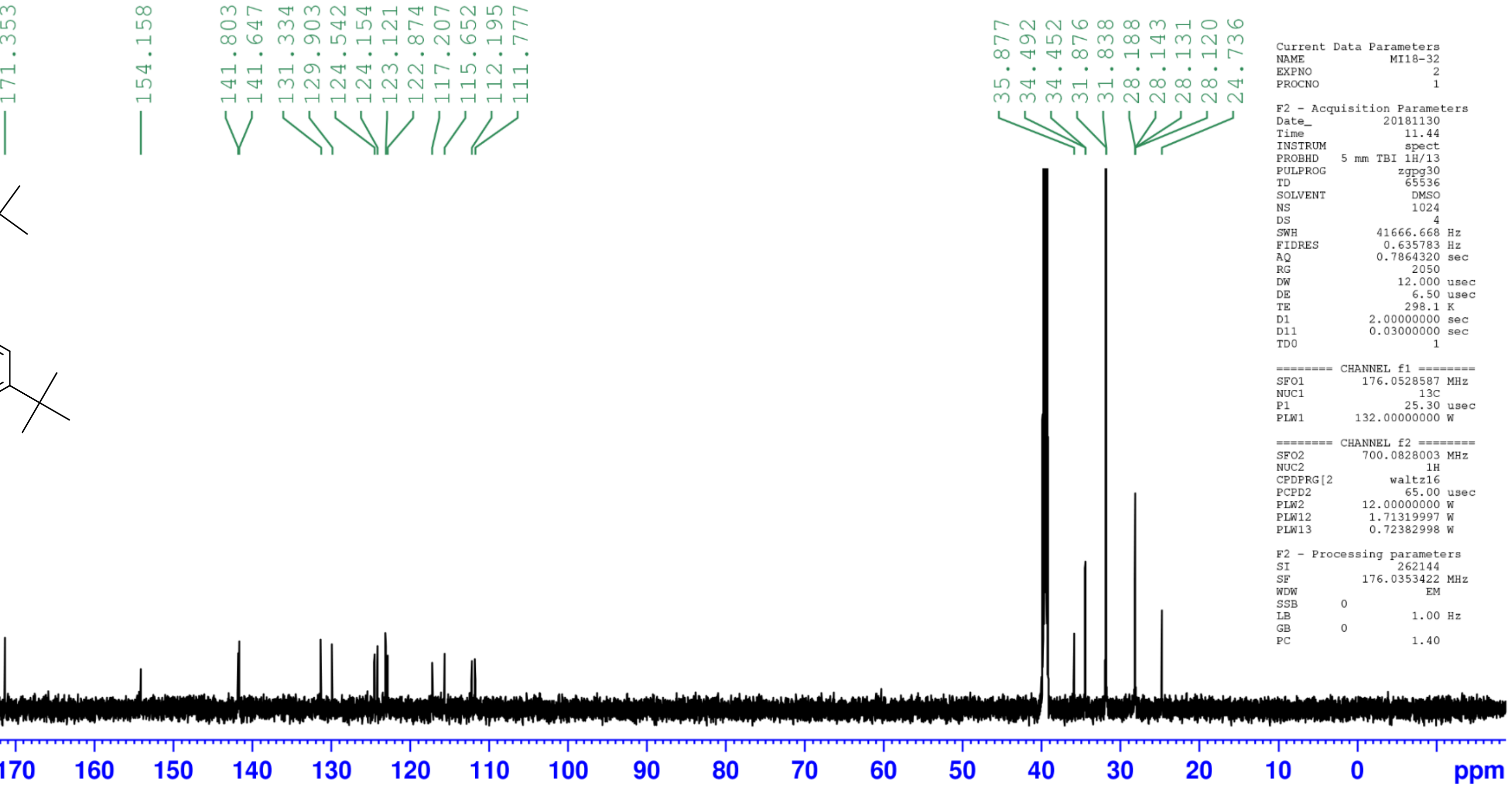

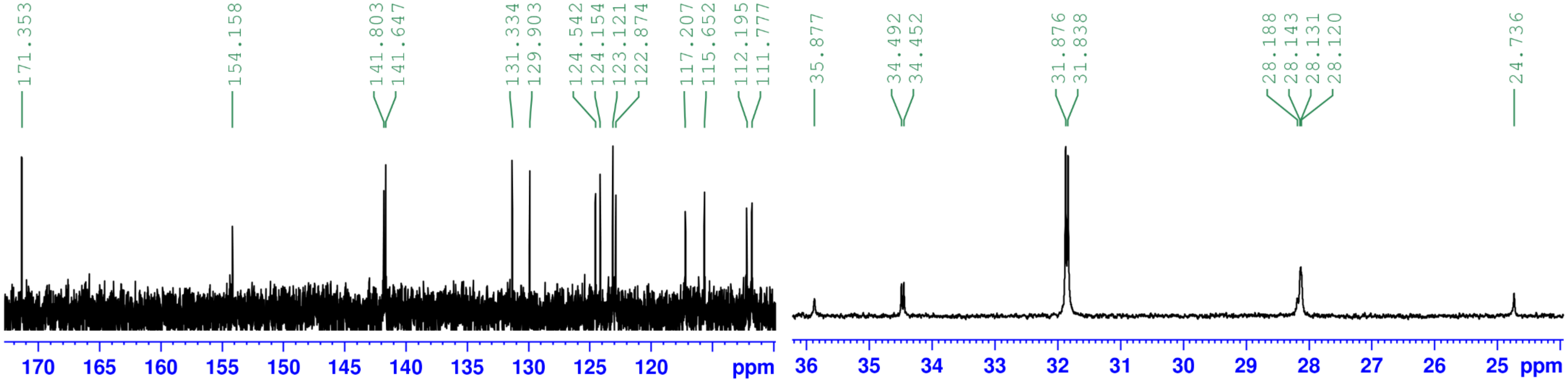

^{1}H-^{13}C HSQC spectrum (700.1 MHz) of compound **MC011**

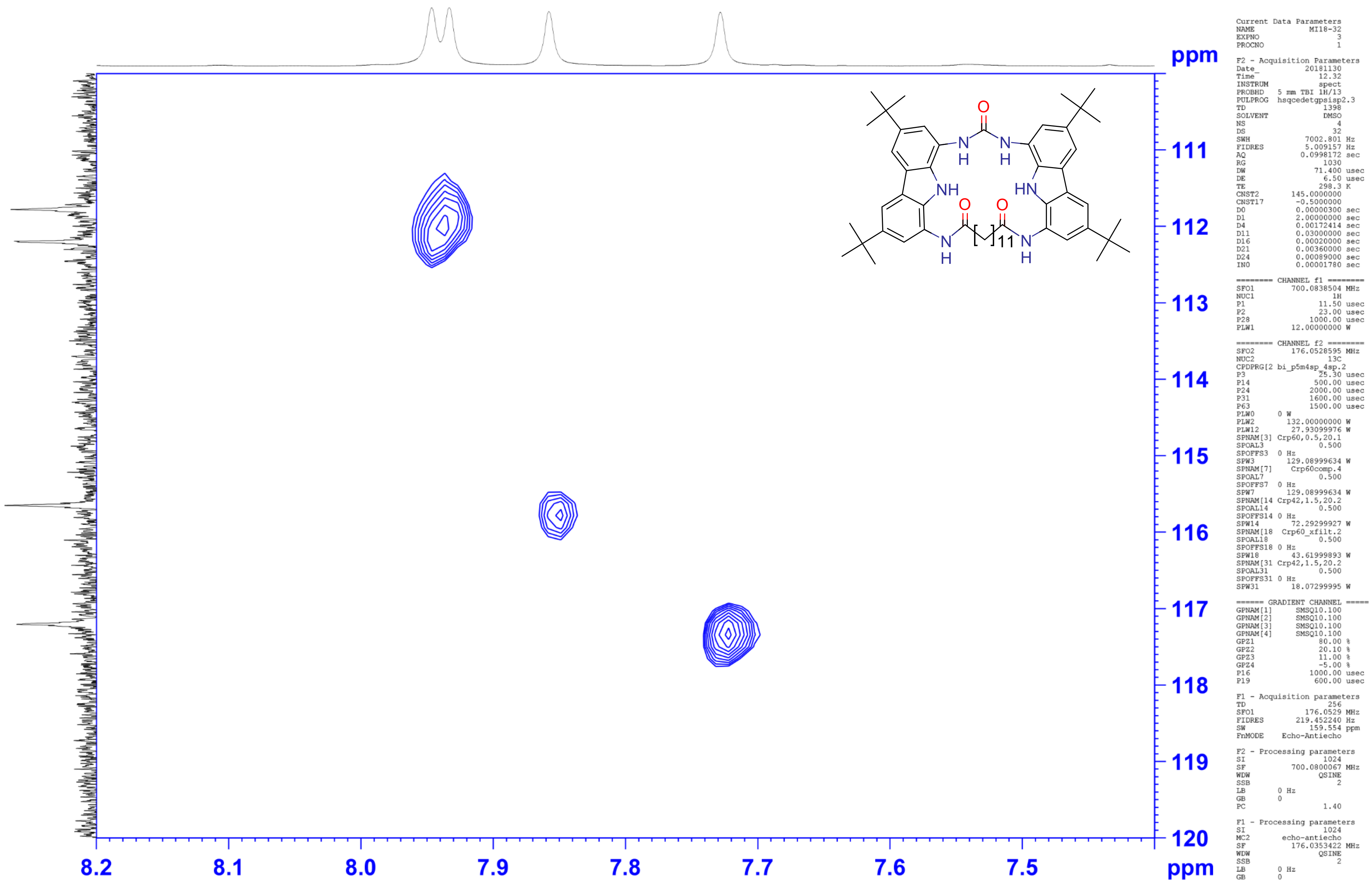

^1H-^{13}C HMBC spectrum (700.1 MHz) of compound **MC011**

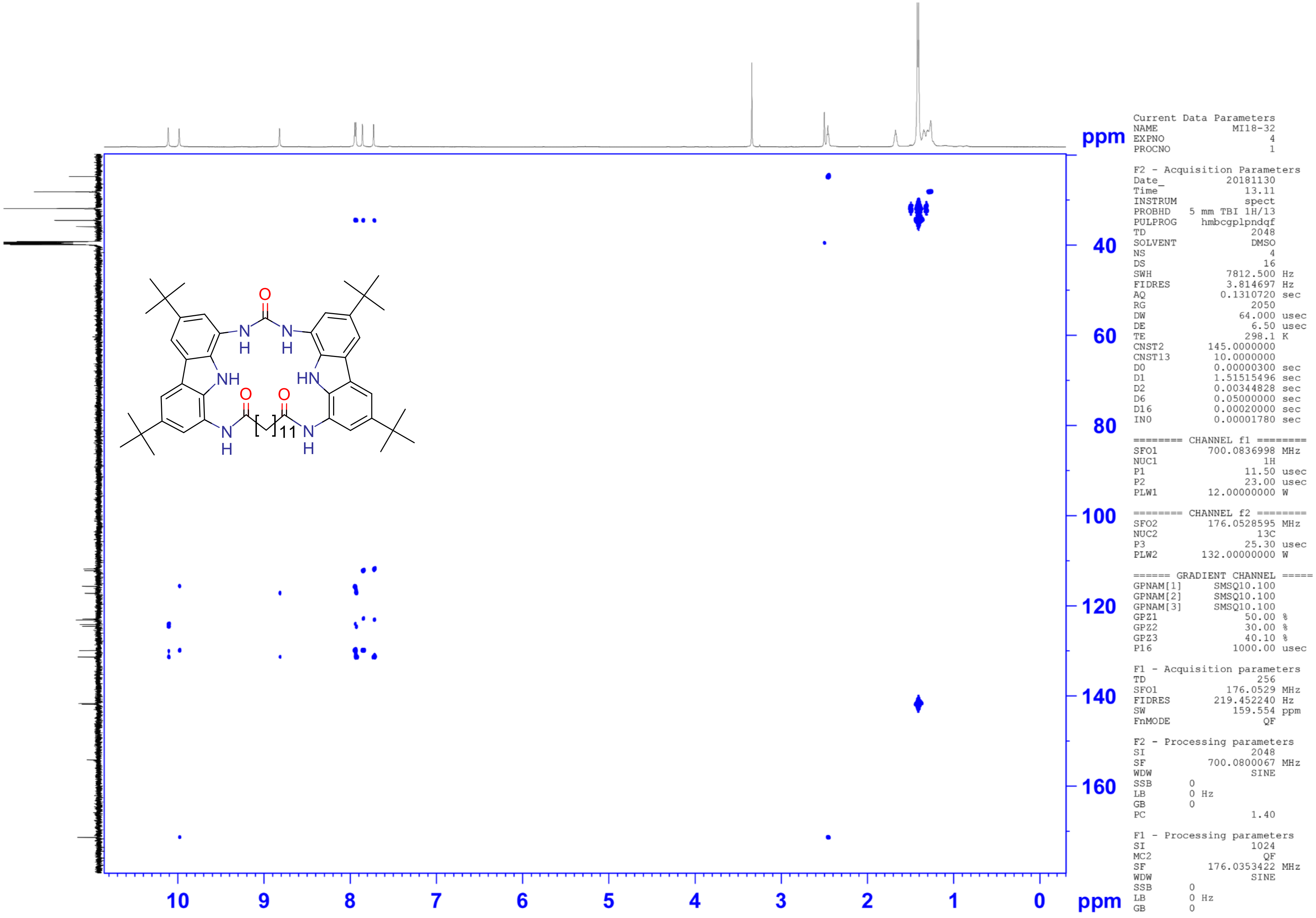

HRMS spectrum of compound **MC011**

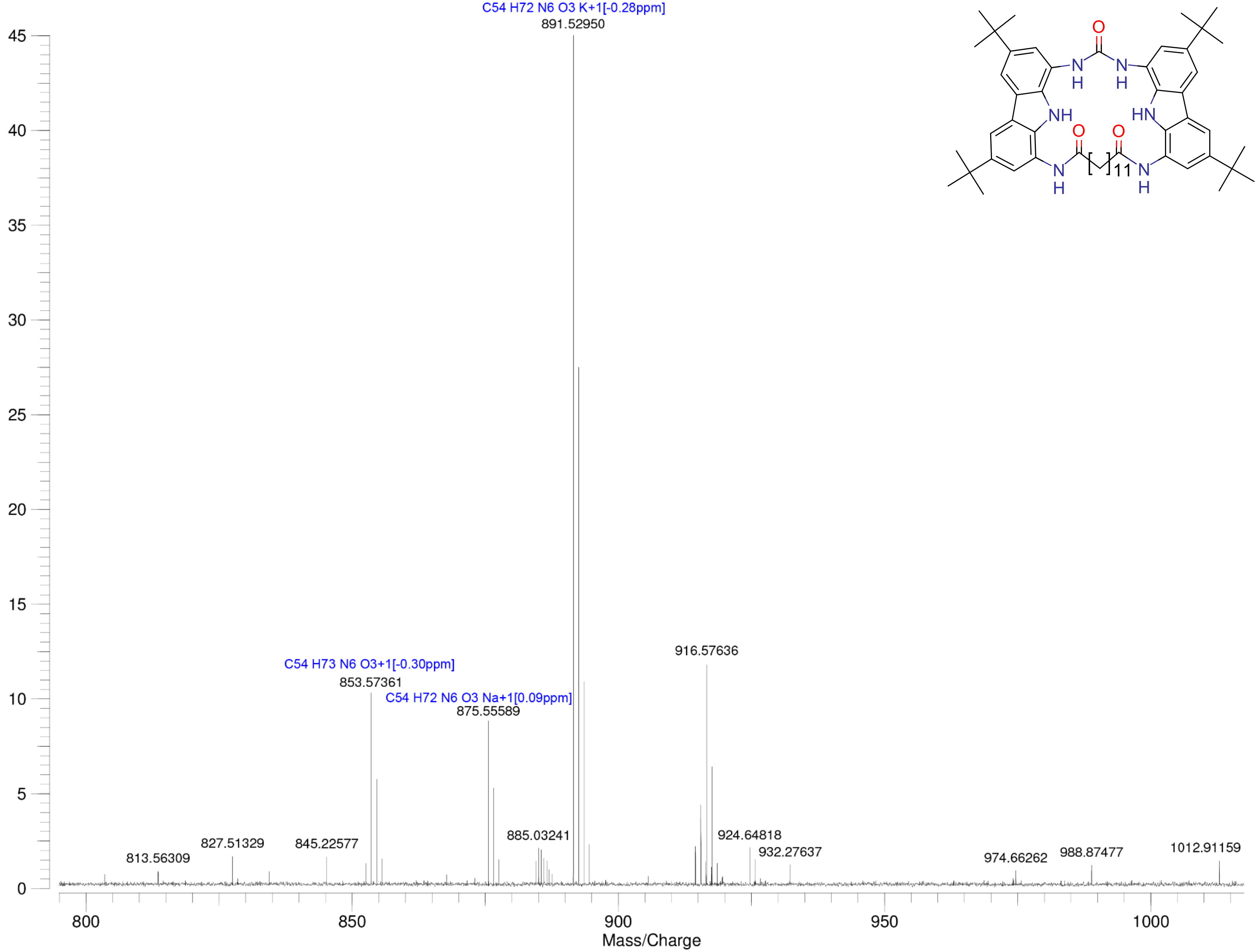

^{1}H NMR spectrum (700.1 MHz) of compound **MC012**

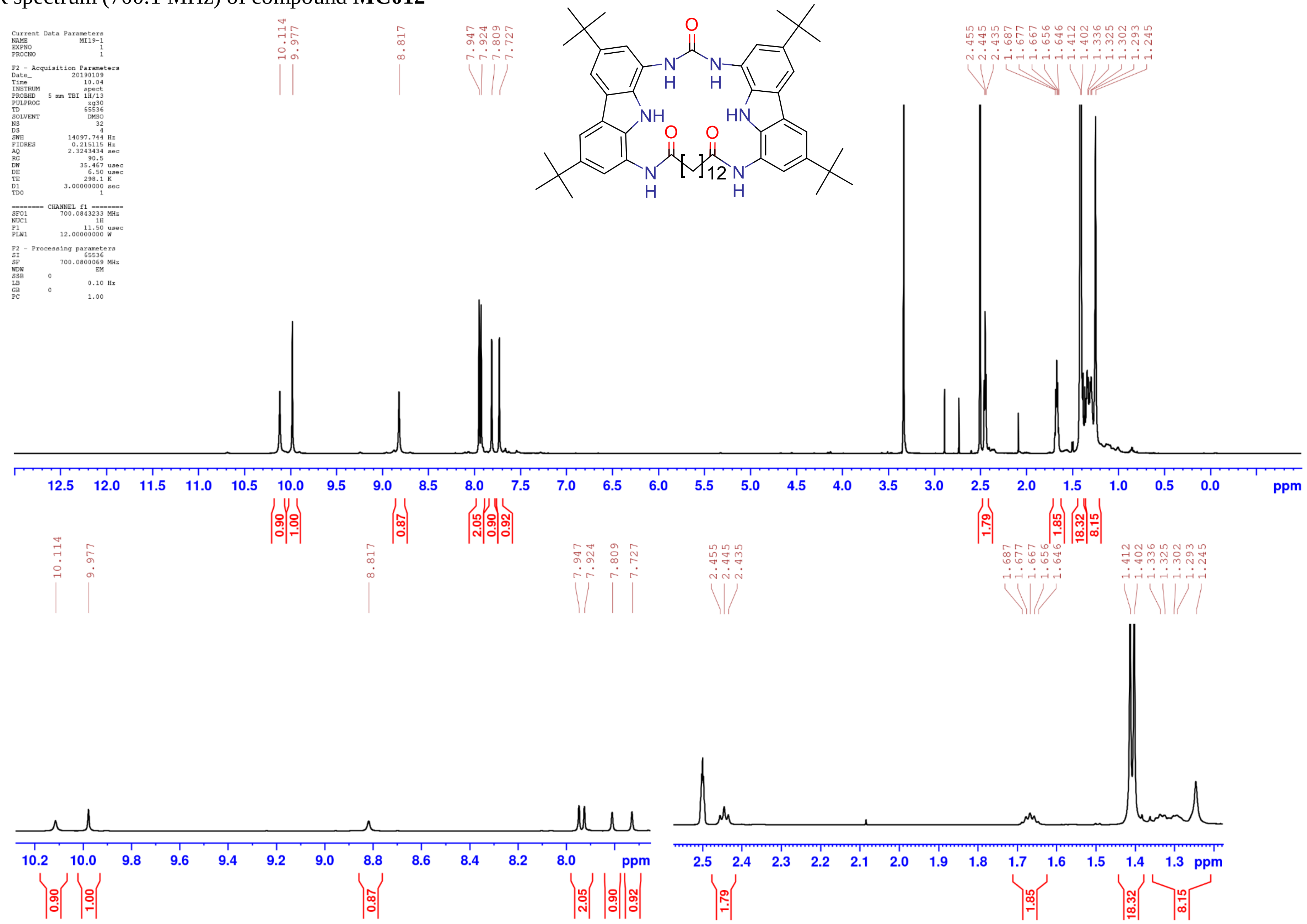

^{13}C NMR spectrum (700.1 MHz) of compound **MC012**

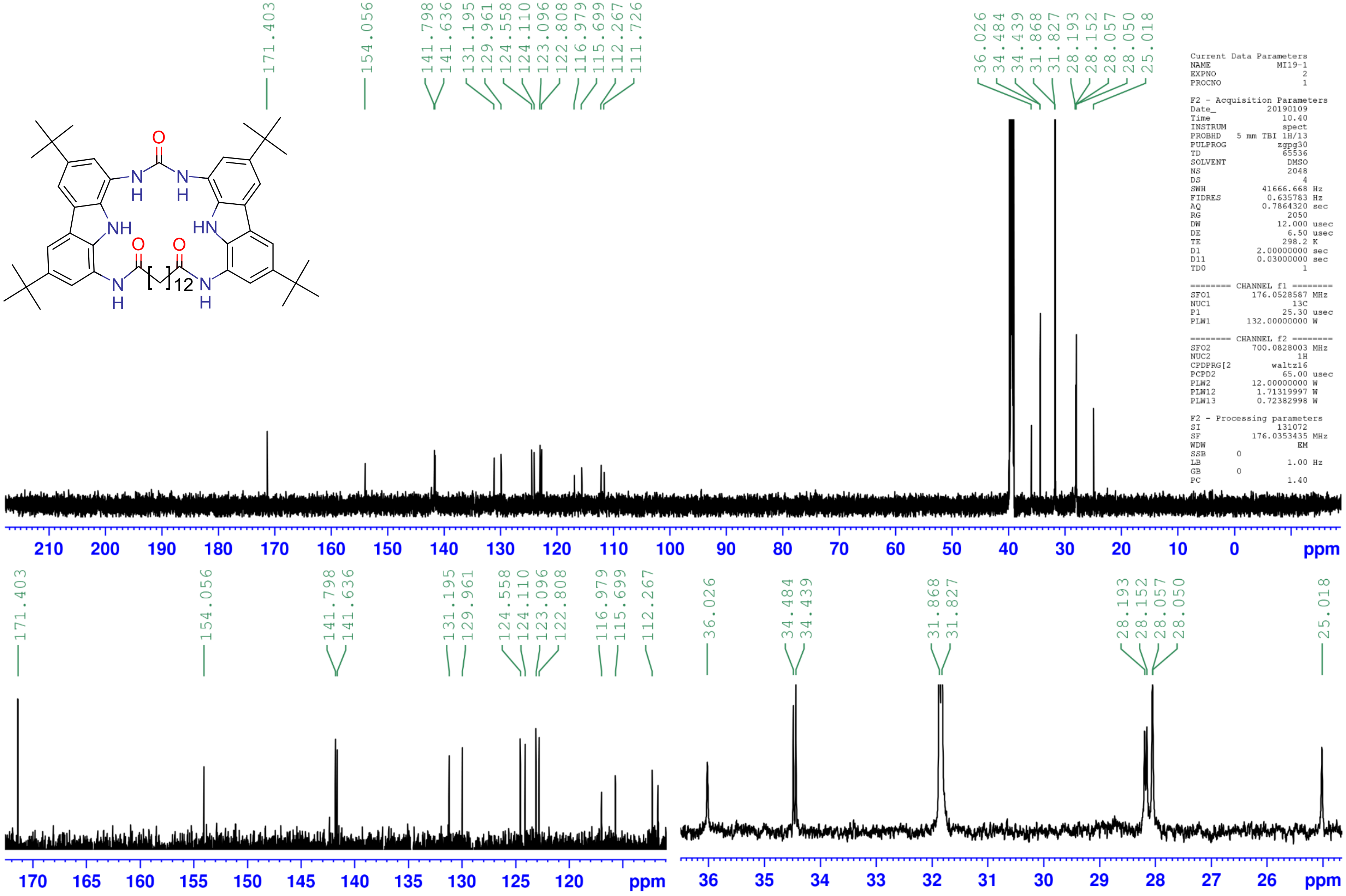

^{1}H-^{13}C HSQC spectrum (700.1 MHz) of compound **MC012**

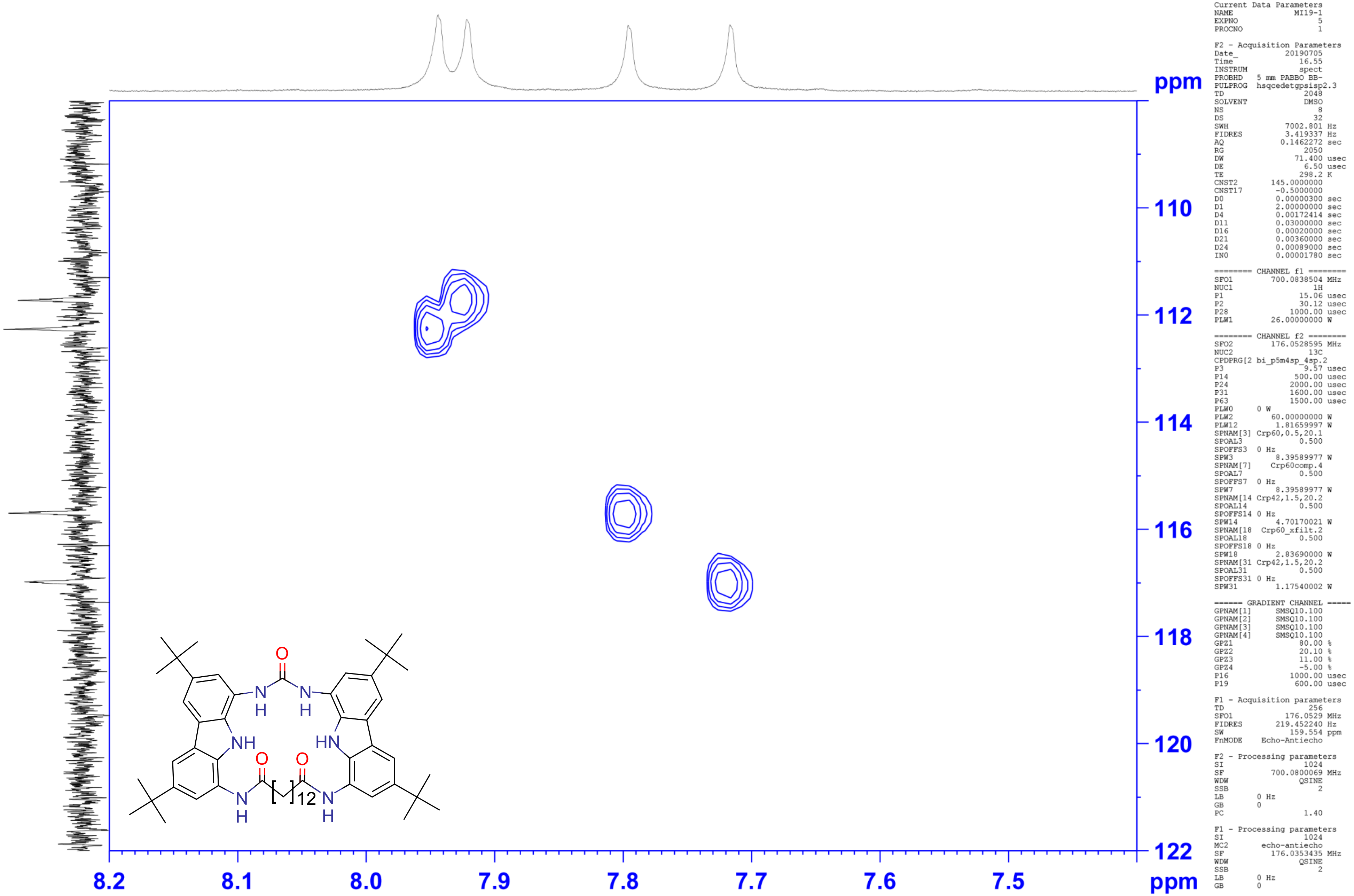

^{1}H-^{13}C HMBC spectrum (700.1 MHz) of compound **MC012**

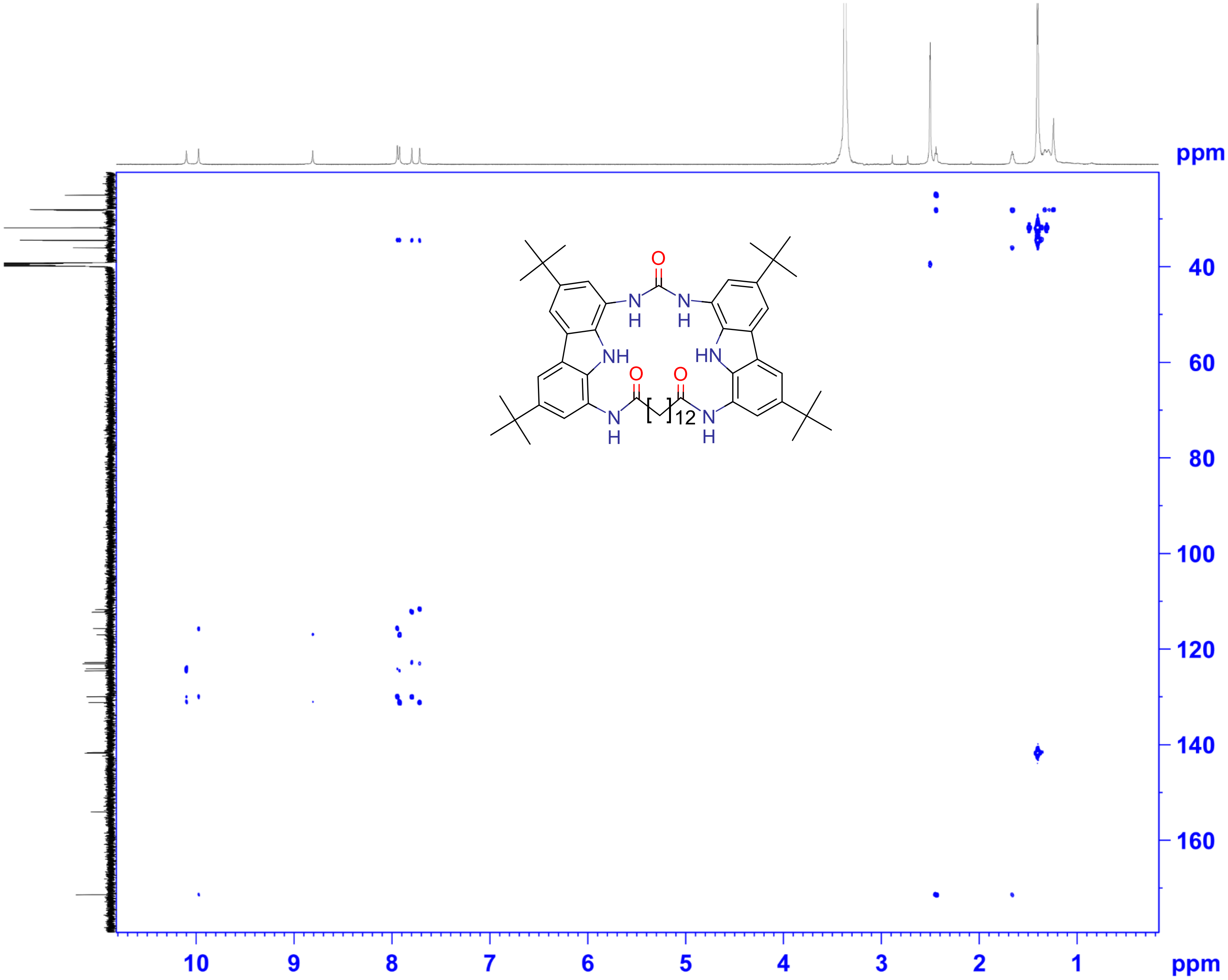


```
Current Data Parameters
NAME              MI19-1
EXPNO                  6
PROCNO                 1

F2 - Acquisition Parameters
Date_           20190705
Time               18.12
INSTRUM            spect
PROBHD   5 mm PABBO BB-
PULPROG    hmbcgplpndqf
TD                  2048
SOLVENT             DMSO
NS                     8
DS                    16
SWH             7462.687 Hz
FIDRES          3.643890 Hz
AQ             0.1372160 sec
RG                  2050
DW                67.000 usec
DE                  6.50 usec
TE                 298.2 K
CNST2        145.0000000
CNST13        10.0000000
D0            0.00000300 sec
D1            1.50901103 sec
D2            0.00344828 sec
D6            0.05000000 sec
D16           0.00020000 sec
IN0           0.00001780 sec

======== CHANNEL f1 ========
SFO1         700.0838481 MHz
NUC1                  1H
P1                 15.06 usec
P2                 30.12 usec
PLW1         26.00000000 W

======== CHANNEL f2 ========
SFO2         176.0528595 MHz
NUC2                 13C
P3                  9.57 usec
PLW2         60.00000000 W

====== GRADIENT CHANNEL =====
GPNAM[1]      SMSQ10.100
GPNAM[2]      SMSQ10.100
GPNAM[3]      SMSQ10.100
GPZ1               50.00 %
GPZ2               30.00 %
GPZ3               40.10 %
P16              1000.00 usec

F1 - Acquisition parameters
TD                   256
SFO1            176.0529 MHz
FIDRES        219.452240 Hz
SW               159.554 ppm
FnMODE                QF

F2 - Processing parameters
SI                  2048
SF           700.0800069 MHz
WDW                 SINE
SSB      0
LB       0 Hz
GB       0
PC                  1.40

F1 - Processing parameters
SI                  1024
MC2                   QF
SF           176.0353435 MHz
WDW                 SINE
SSB      0
LB       0 Hz
GB       0
```

HRMS spectrum of compound **MC012**

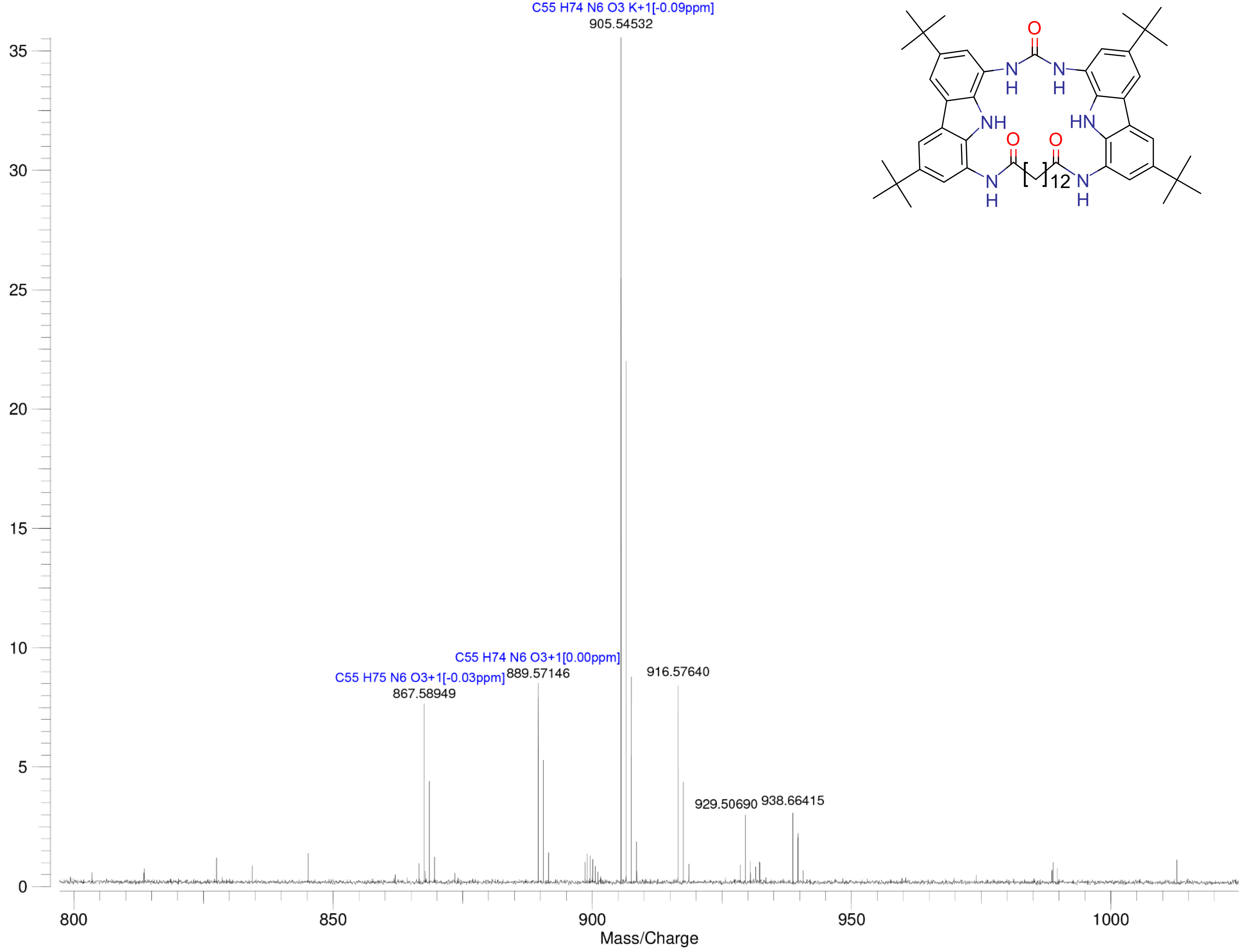

^{1}H NMR spectrum (700.1 MHz) of compound **MC013**

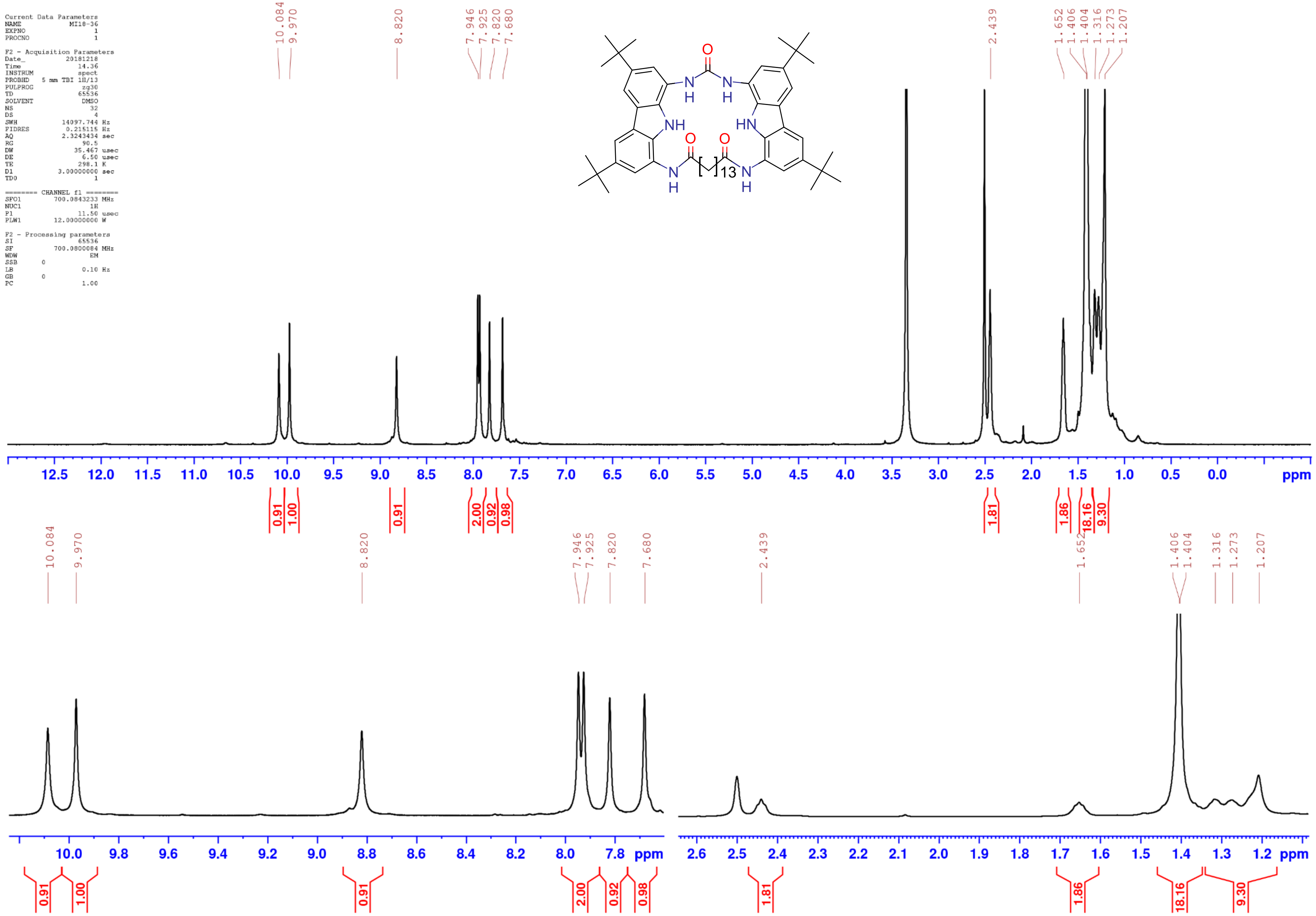

^{13}C NMR spectrum (700.1 MHz) of compound **MC013**

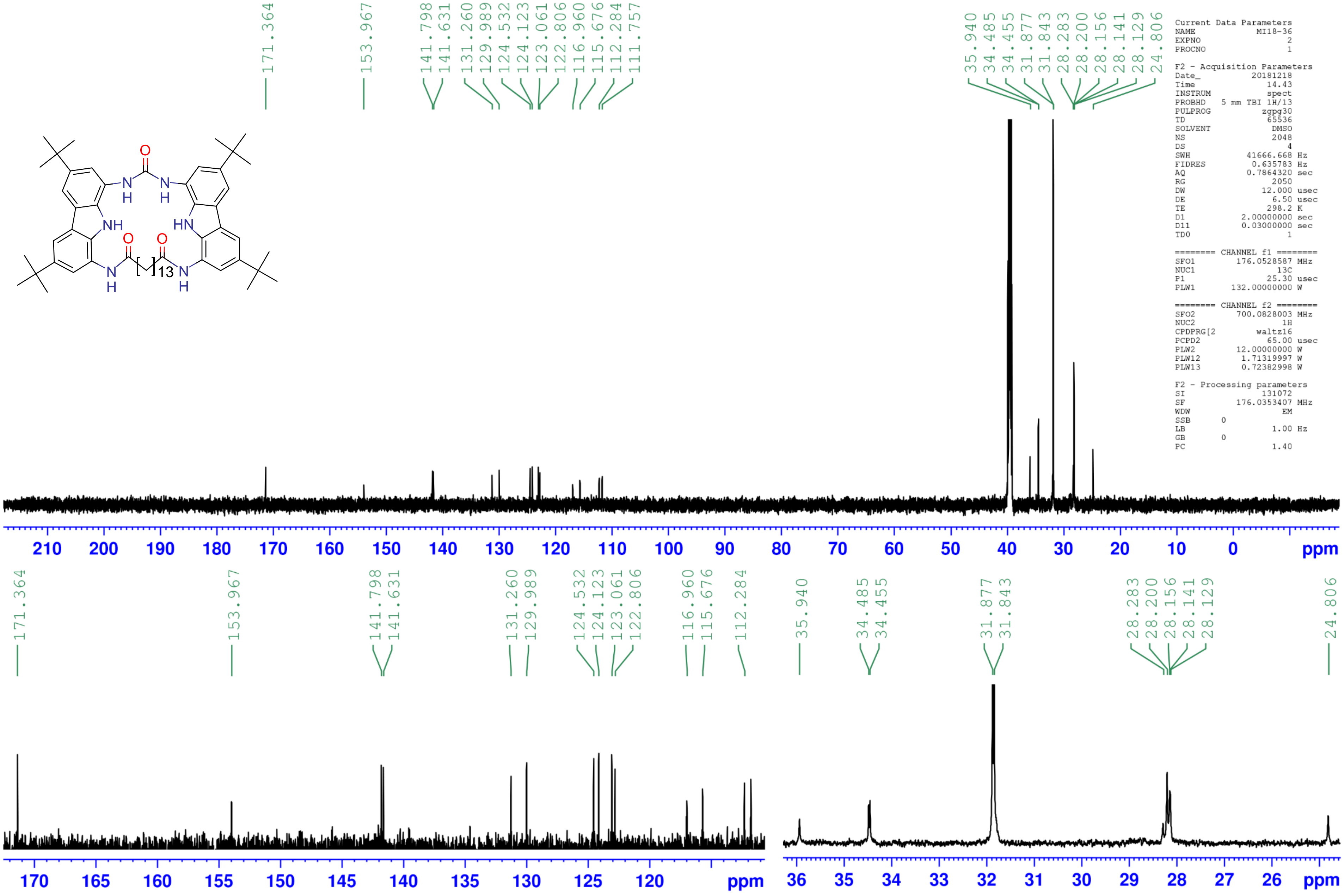

^{1}H-^{13}C HSQC spectrum (700.1 MHz) of compound **MC013**

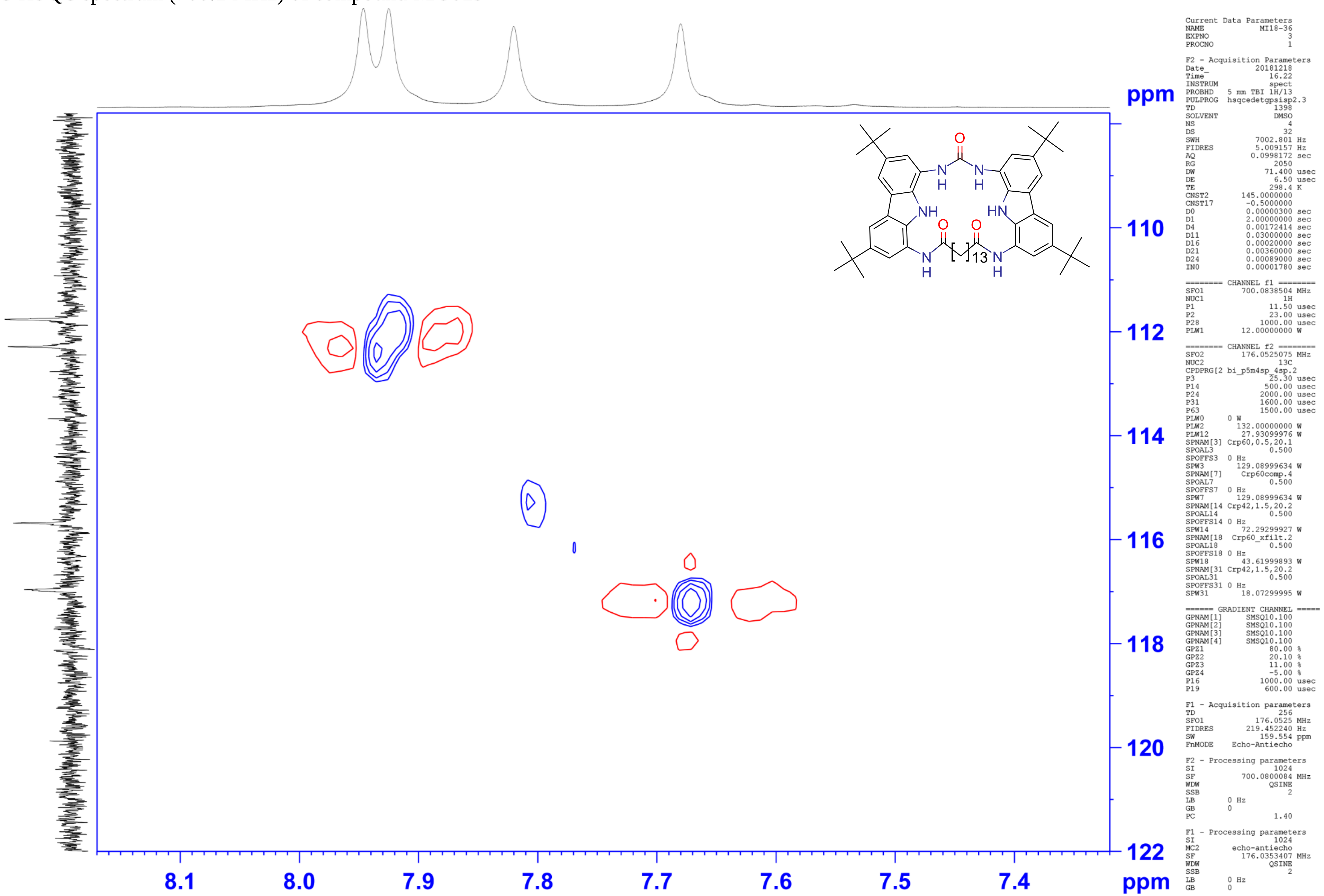

^{1}H-^{13}C HMBC spectrum (700.1 MHz) of compound **MC013**

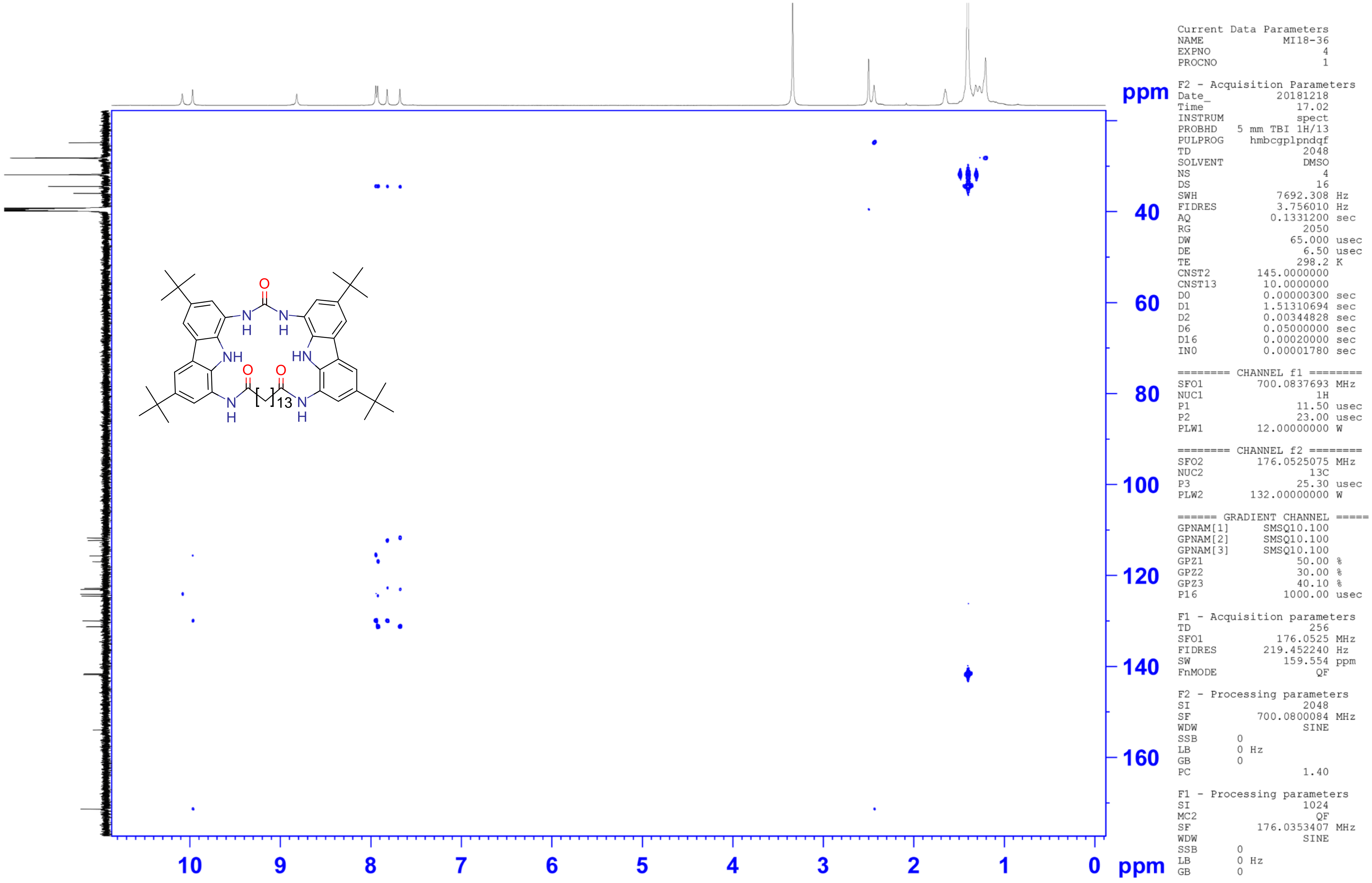

HRMS spectrum of compound **MC013**

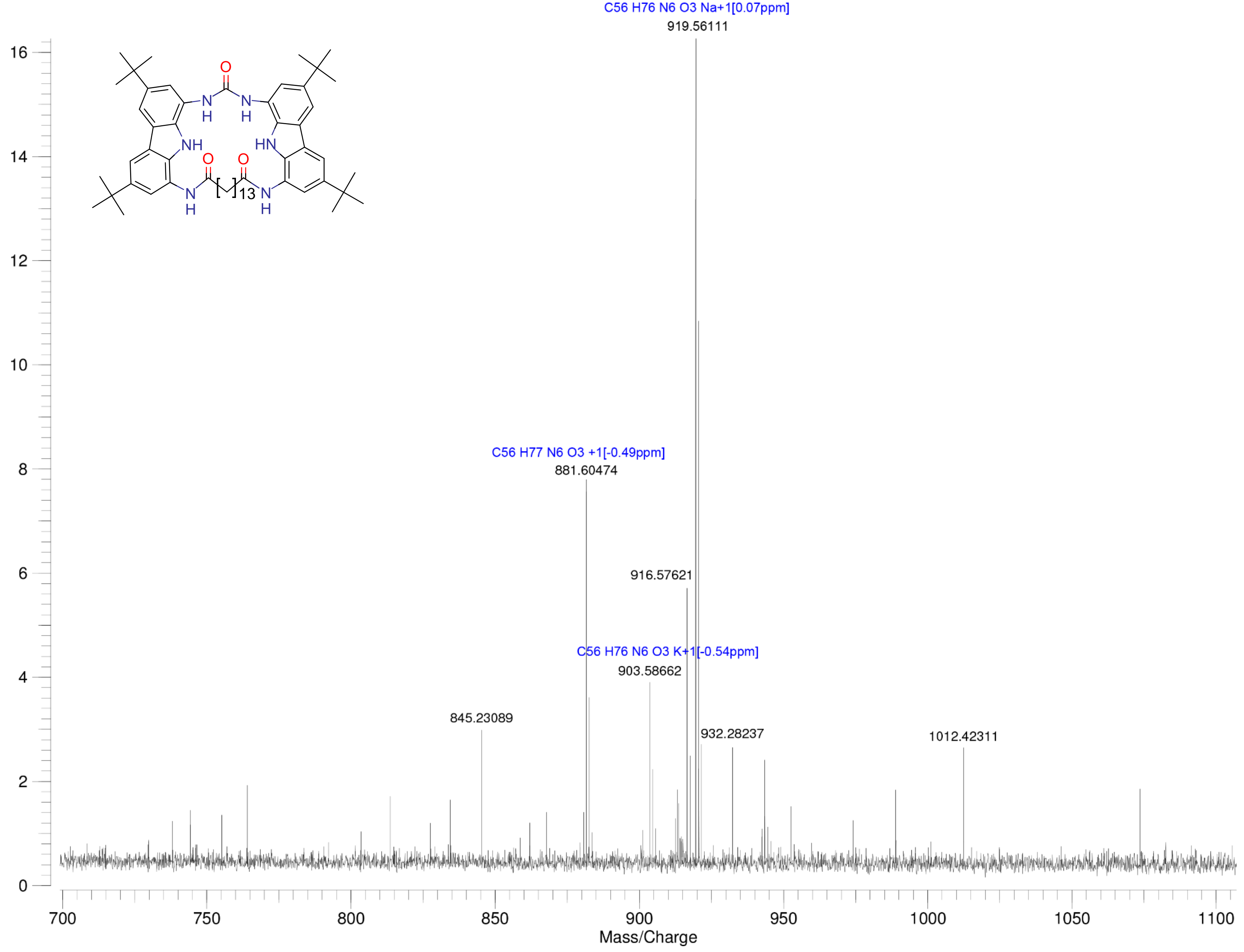

^{1}H NMR spectrum (700.1 MHz) of compound **MC014**

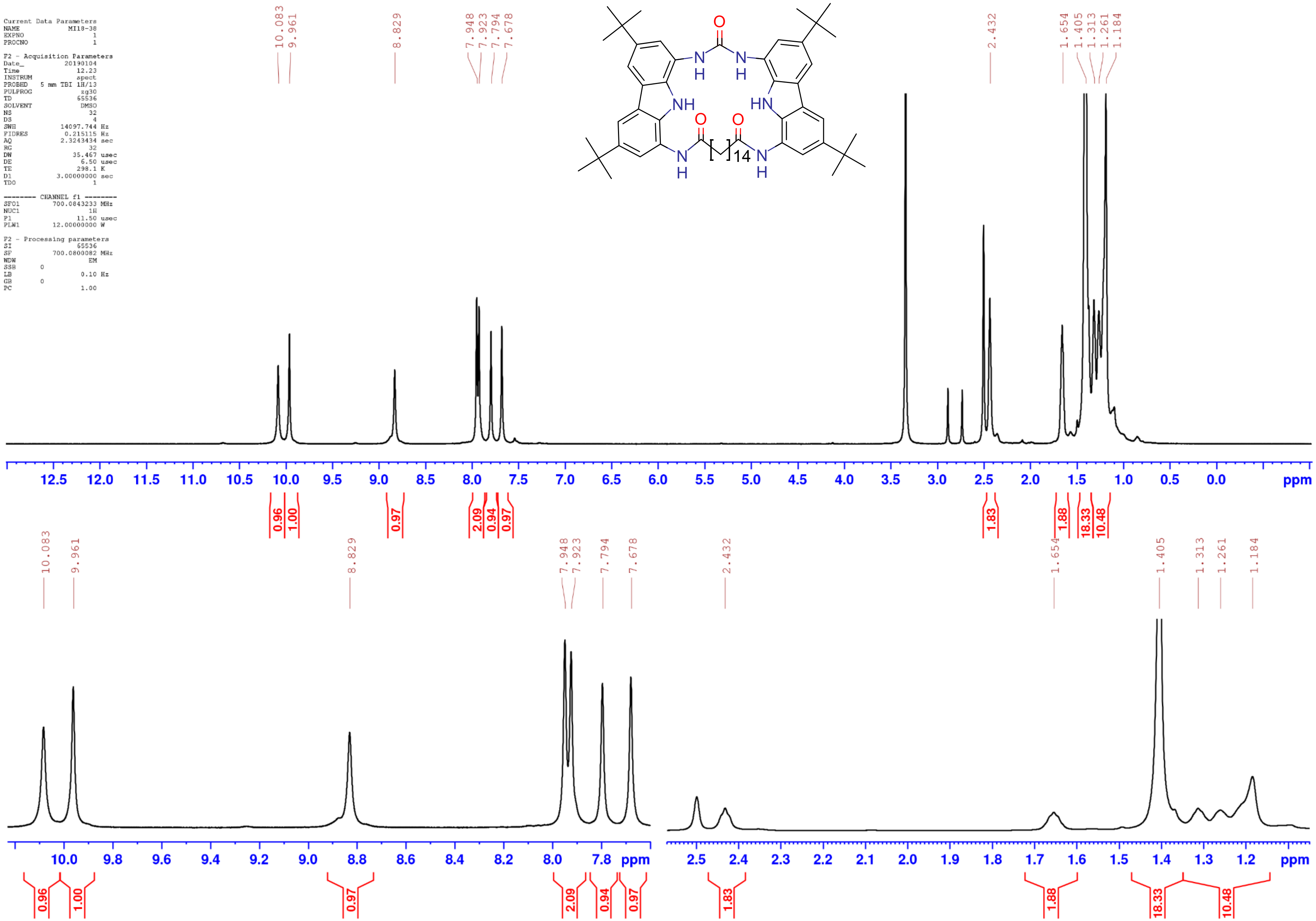

^{13}C NMR spectrum (700.1 MHz) of compound **MC014**

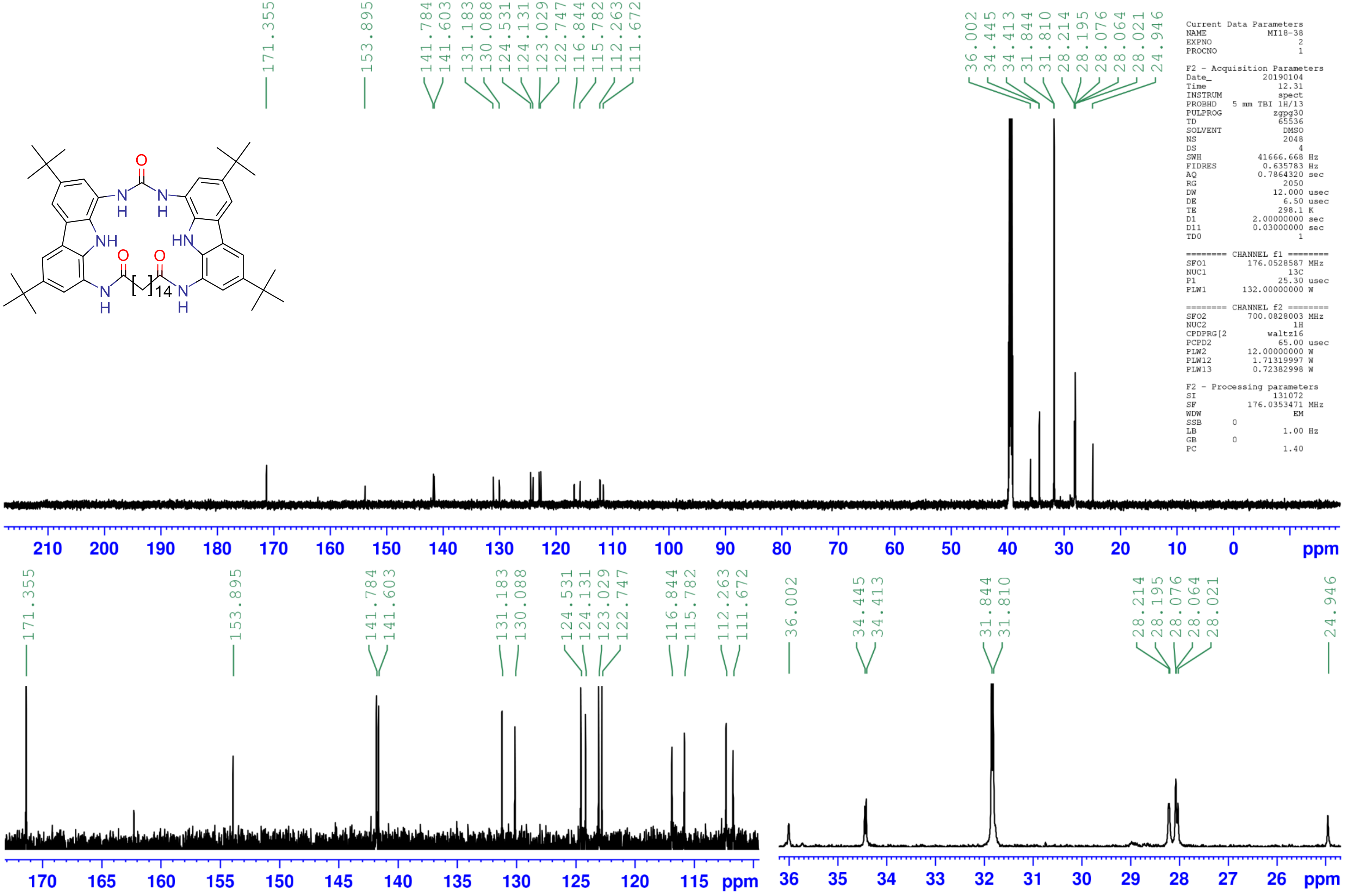

^{1}H-^{13}C HSQC spectrum (700.1 MHz) of compound **MC014**

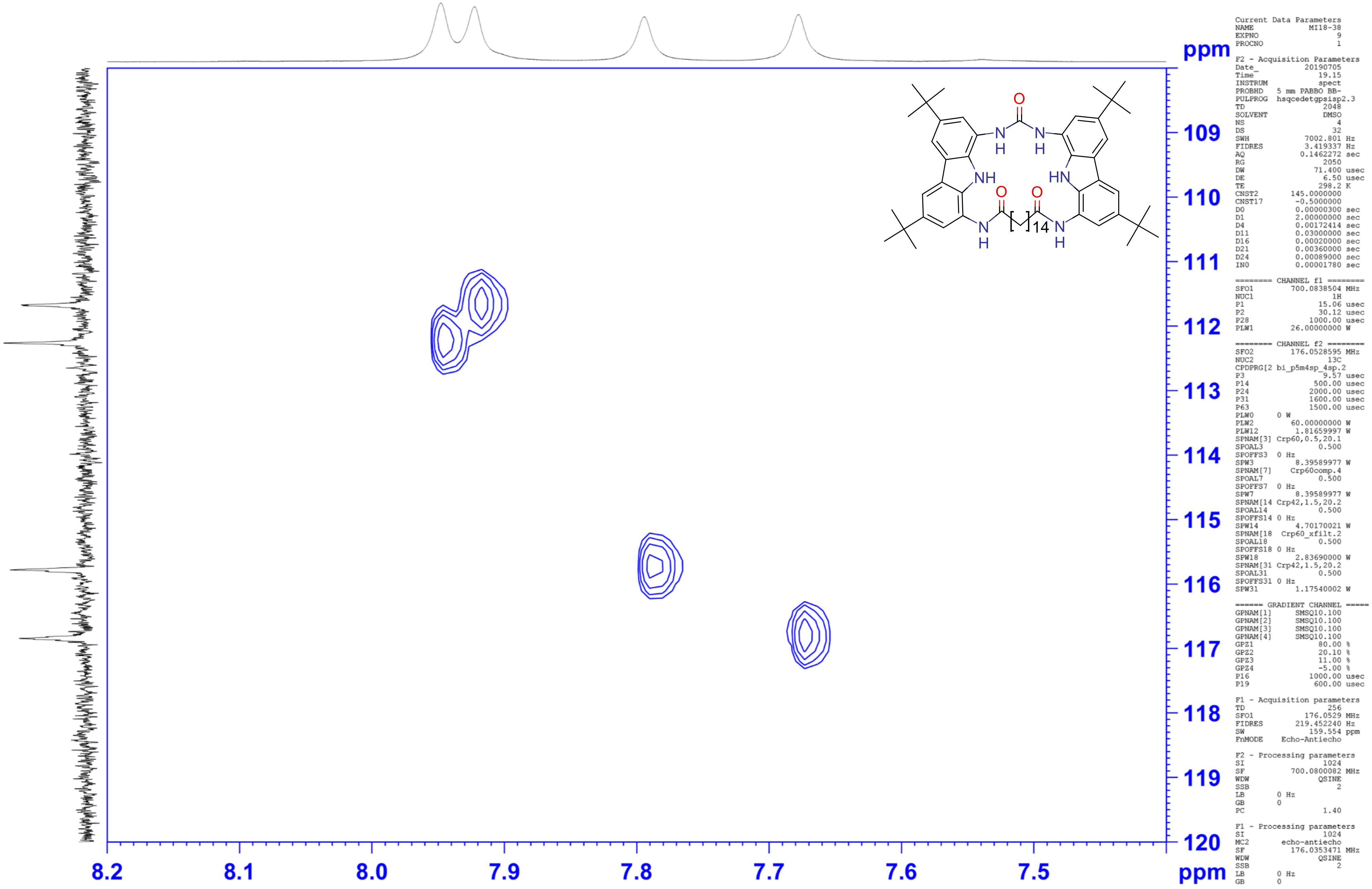

^{1}H-^{13}C HMBC spectrum (700.1 MHz) of compound **MC014**

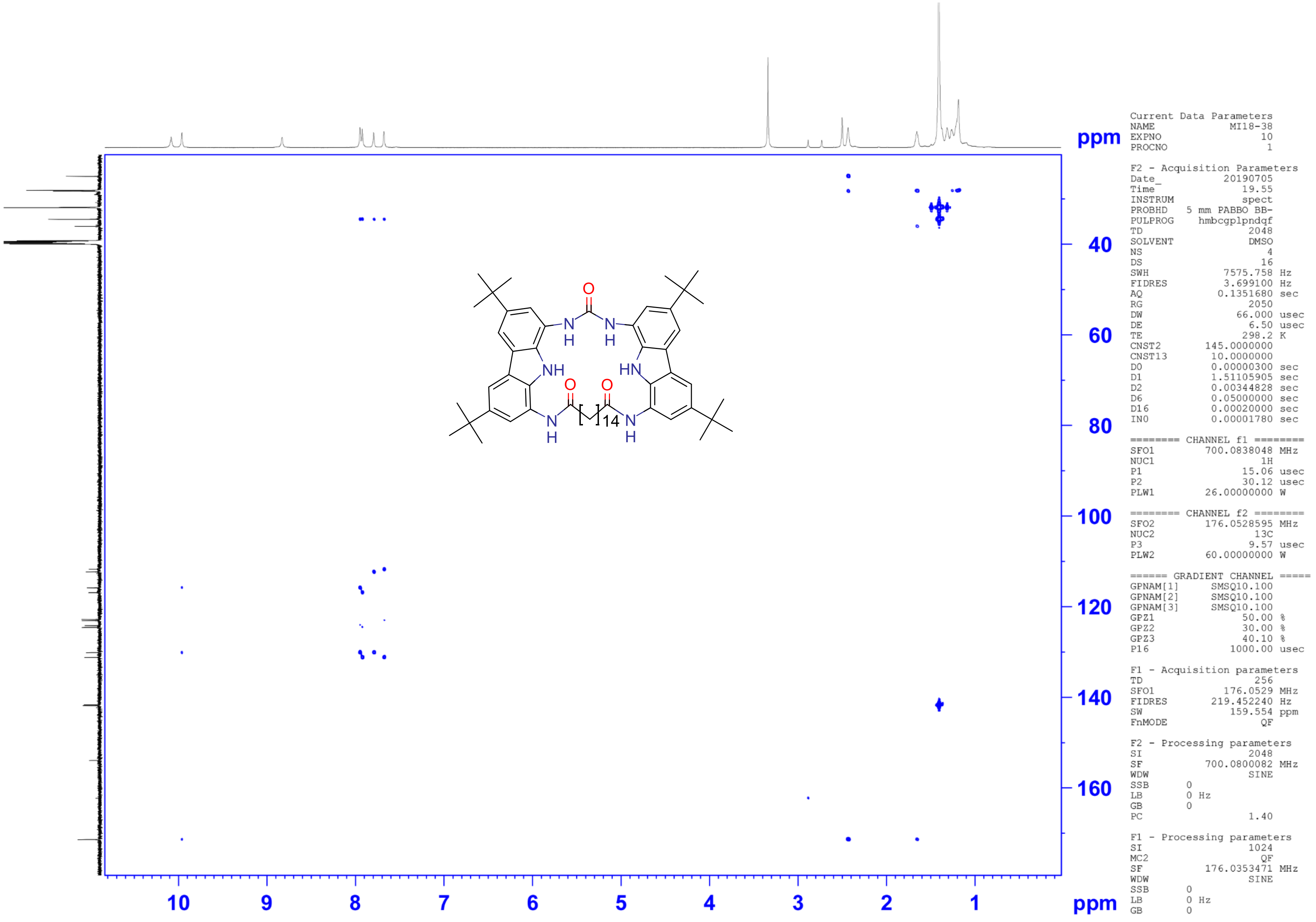

HRMS spectrum of compound **MC014**

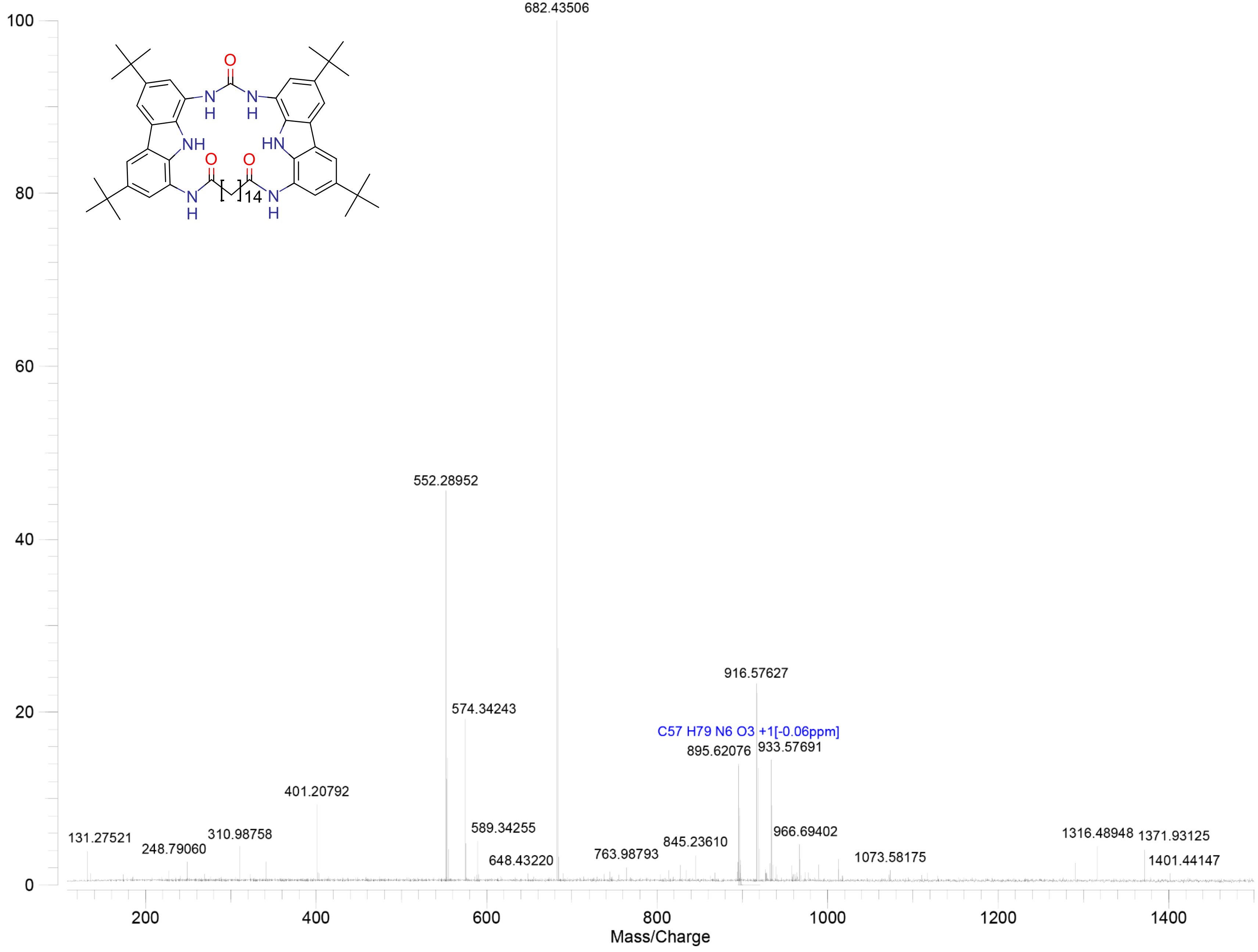

Stacked ^{1}H NMR spectra (700.1 MHz) of a mixture of receptors **CZ016**; 1,3-diindolylurea; 1,3-dicarbazolylurea + TBA-acetate

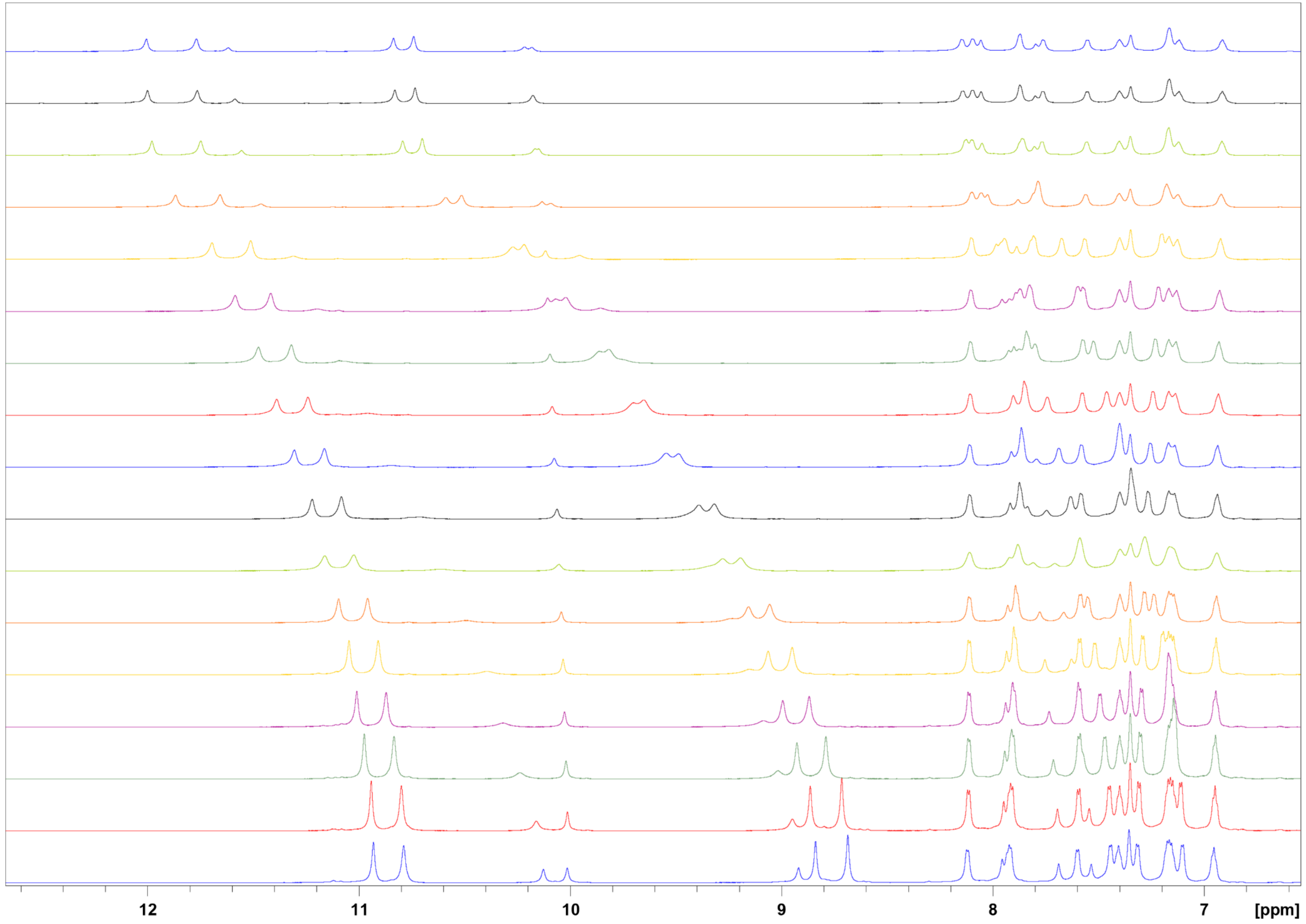

Stacked ^{1}H NMR spectra (700.1 MHz) of a mixture of receptors **MC001**; 2,7-$(COOBu)_2$-indolocarbazole; 2,9-$(COOBu)_2$-indolocarbazole + TBA-acetate

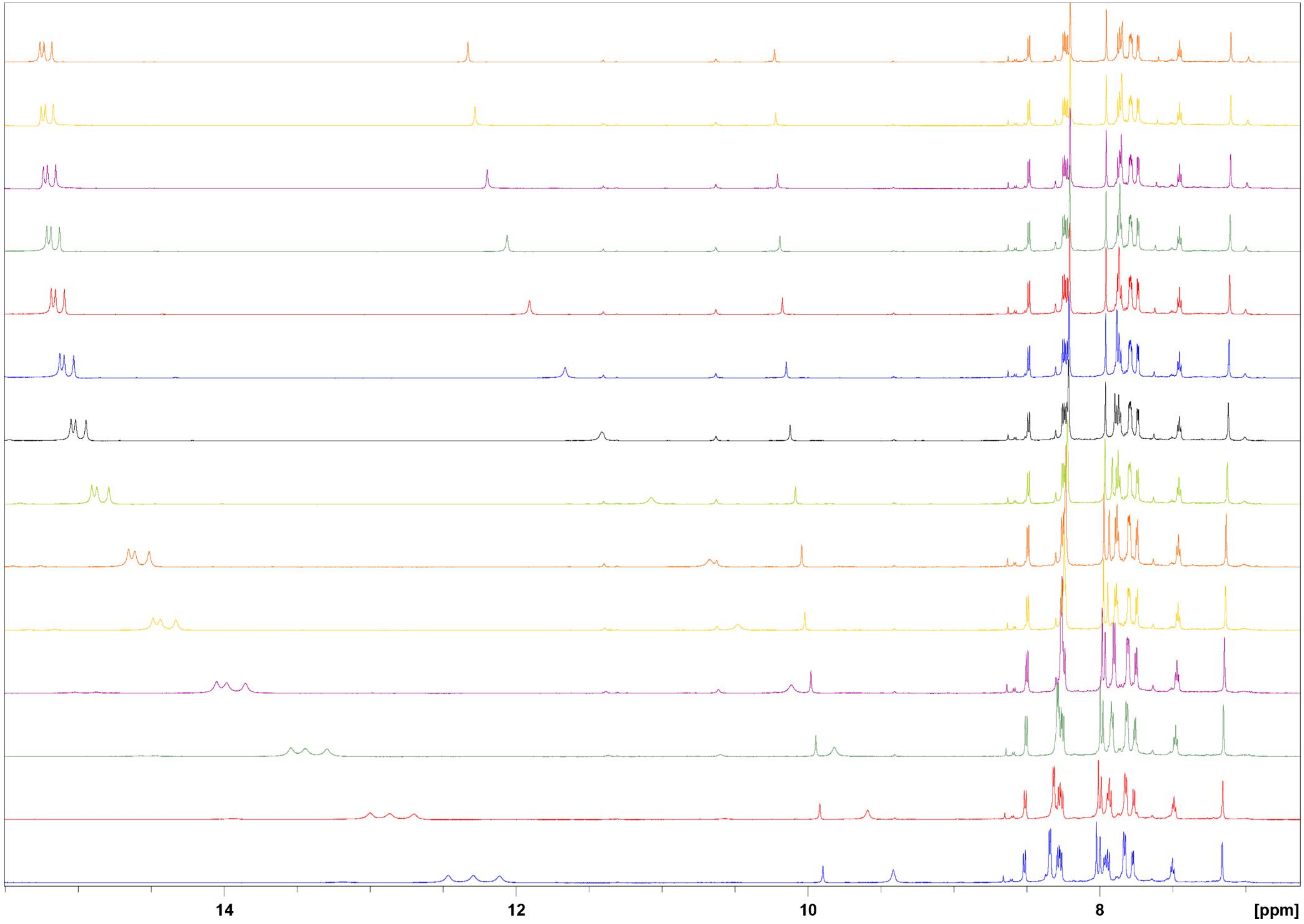

Stacked ^{1}H NMR spectra (700.1 MHz) of a mixture of receptors **MC002**; indolocarbazole; 2,7-$(Cl)_2$-indolocarbazole + TBA-acetate

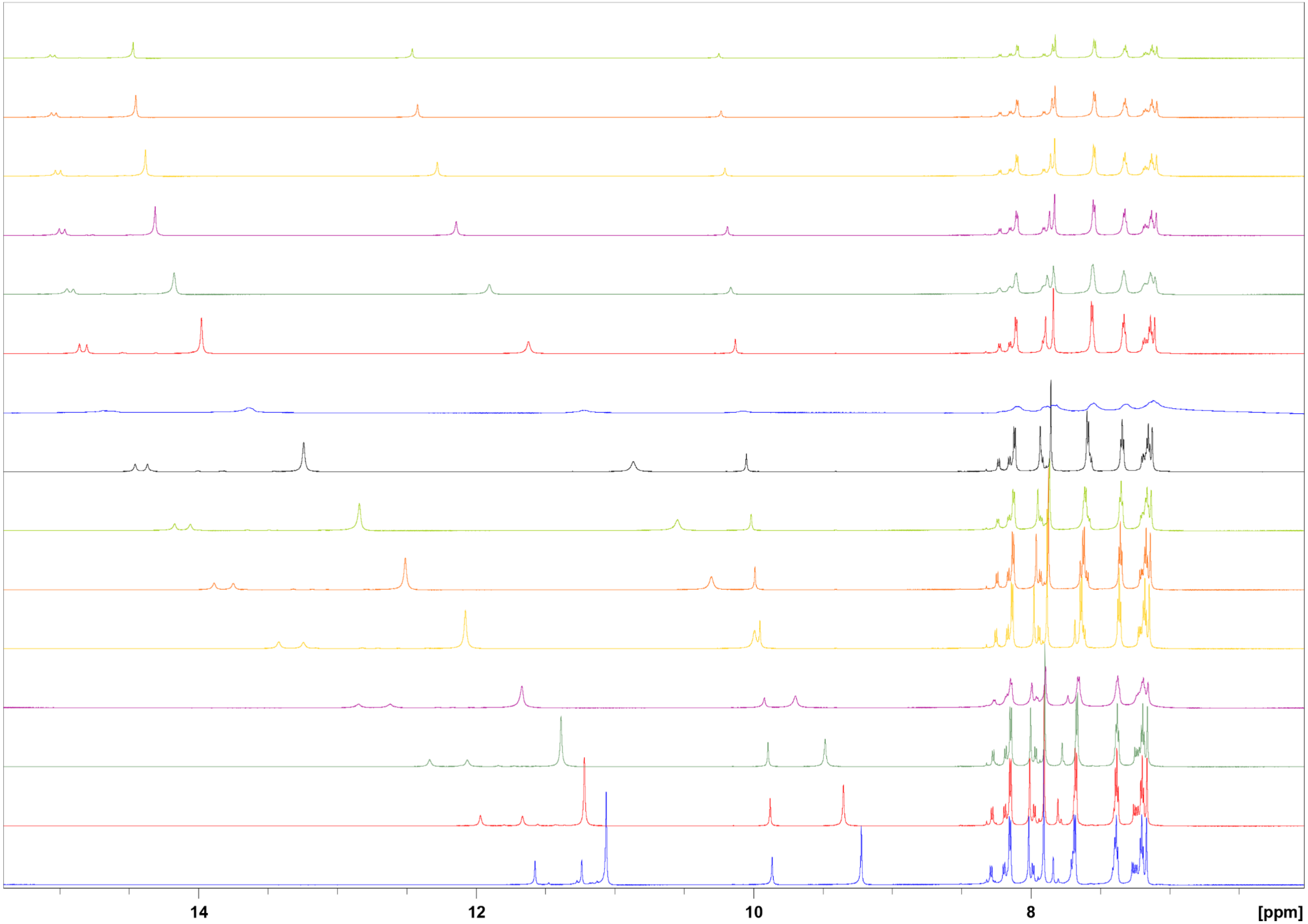

Stacked ^{1}H NMR spectra (700.1 MHz) of a mixture of receptors **MC003**; 2,7-(COOBu)$_2$-indolocarbazole; 2,9-(COOBu)$_2$-indolocarbazole + TBA-acetate

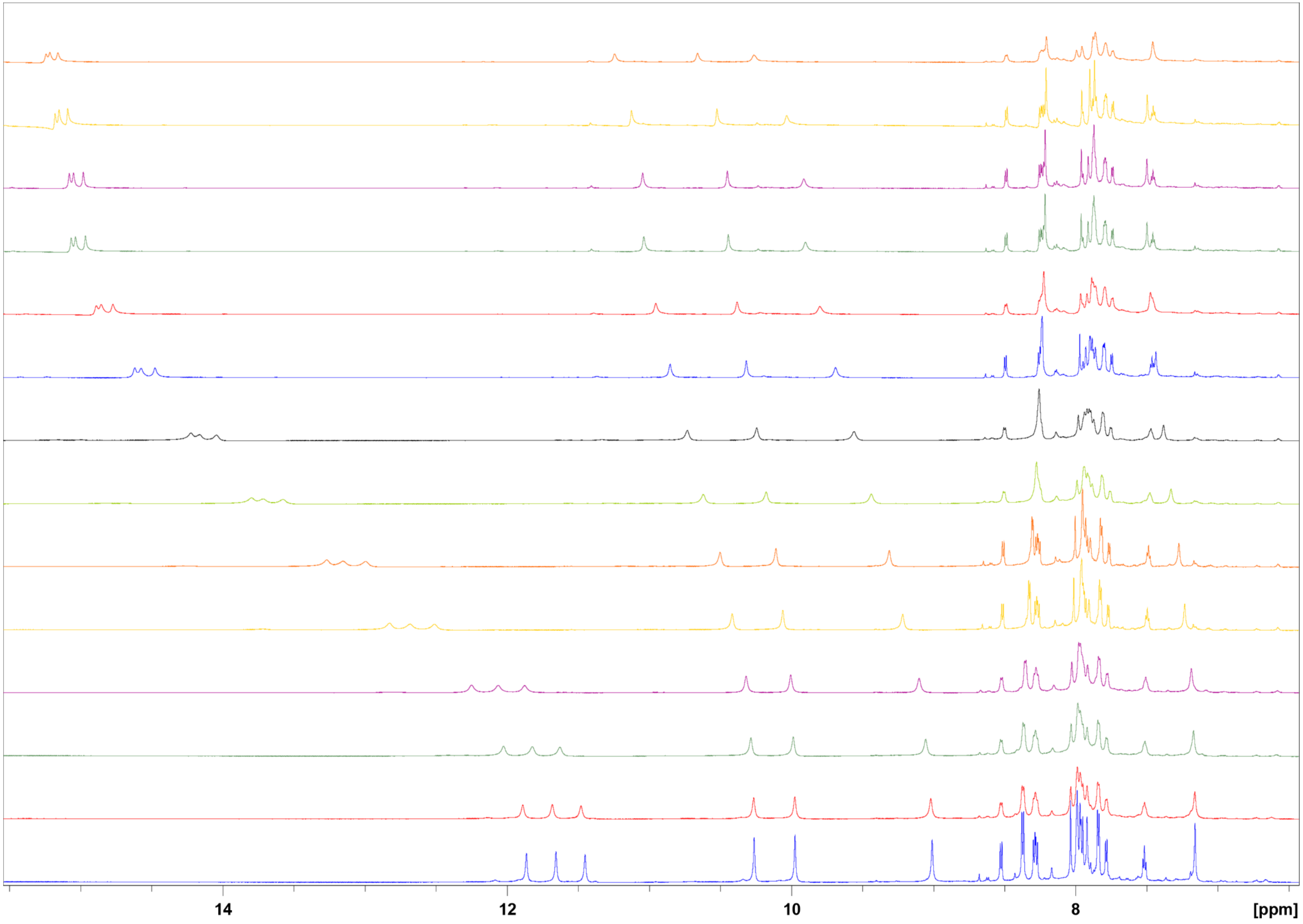

Stacked ^{1}H NMR spectra (700.1 MHz) of a mixture of receptors **MC004**; 1,3-diindolylurea; 1,3-dicarbazolylurea + TBA-acetate

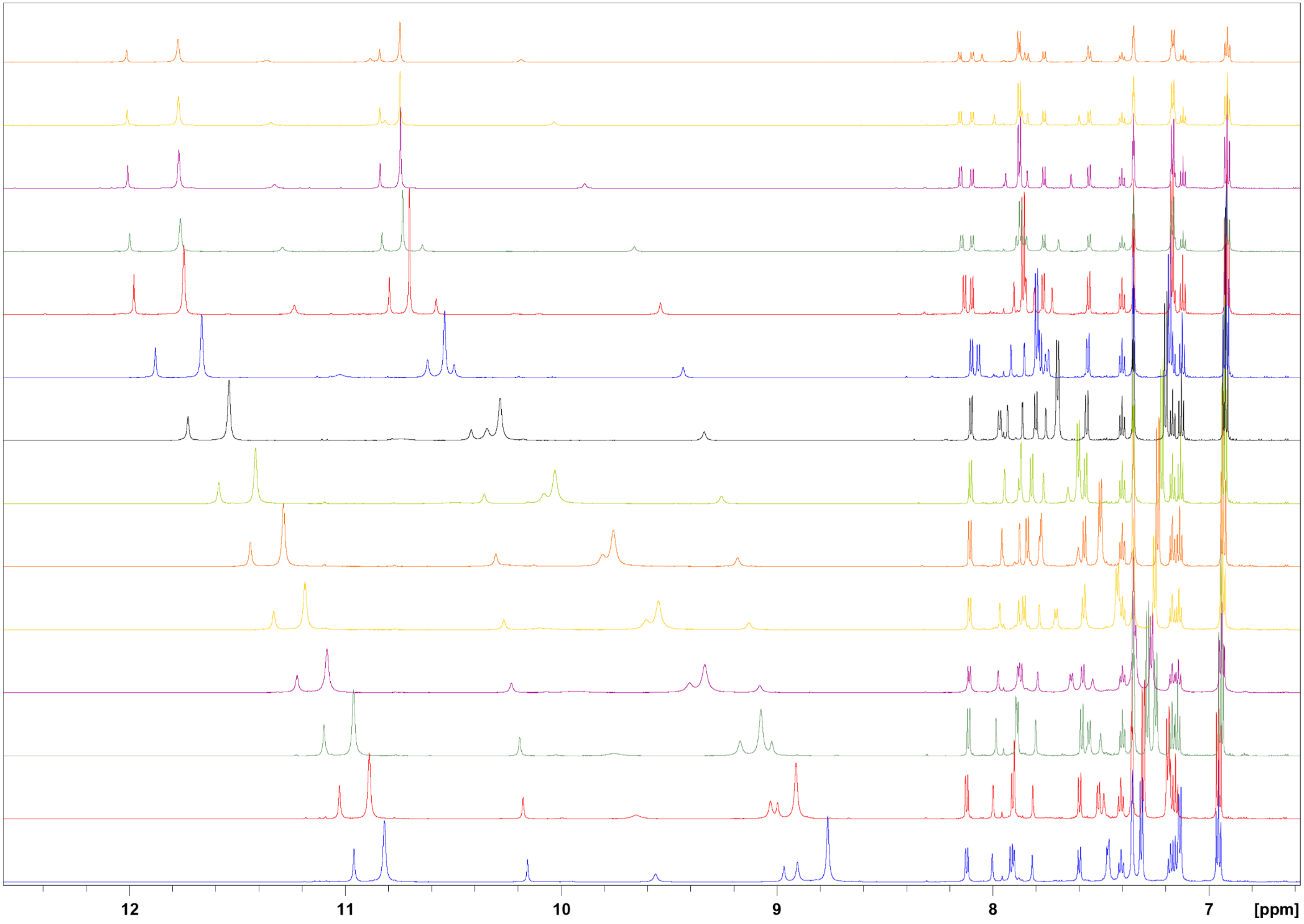

Stacked ^{1}H NMR spectra (700.1 MHz) of a mixture of receptors **MC005**; 1,3-diindolylurea; 1,3-dicarbazolylurea + TBA-acetate

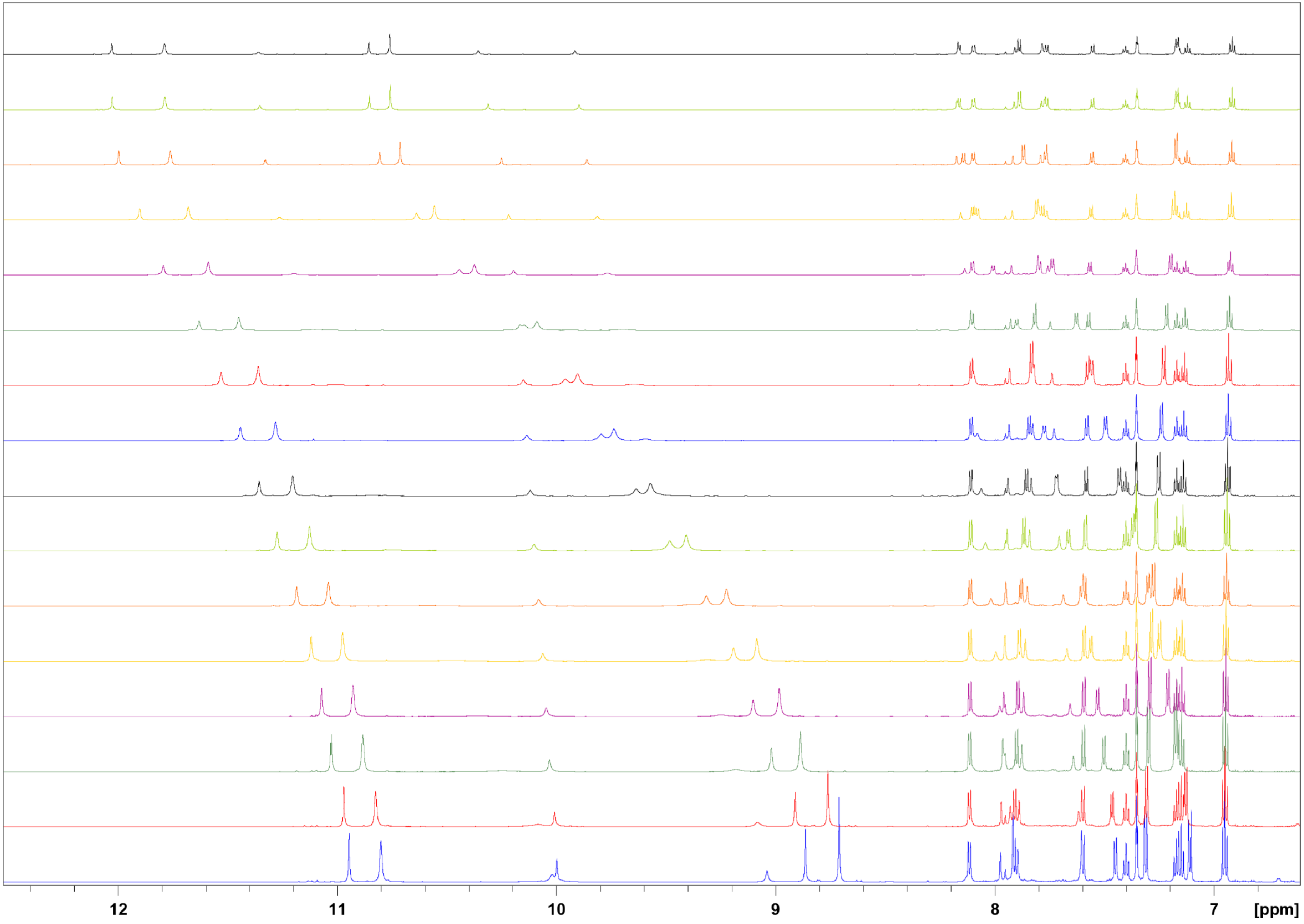

Stacked ^{1}H NMR spectra (700.1 MHz) of a mixture of receptors **MC006**; 1,3-diindolylurea; 1,3-dicarbazolylurea + TBA-acetate

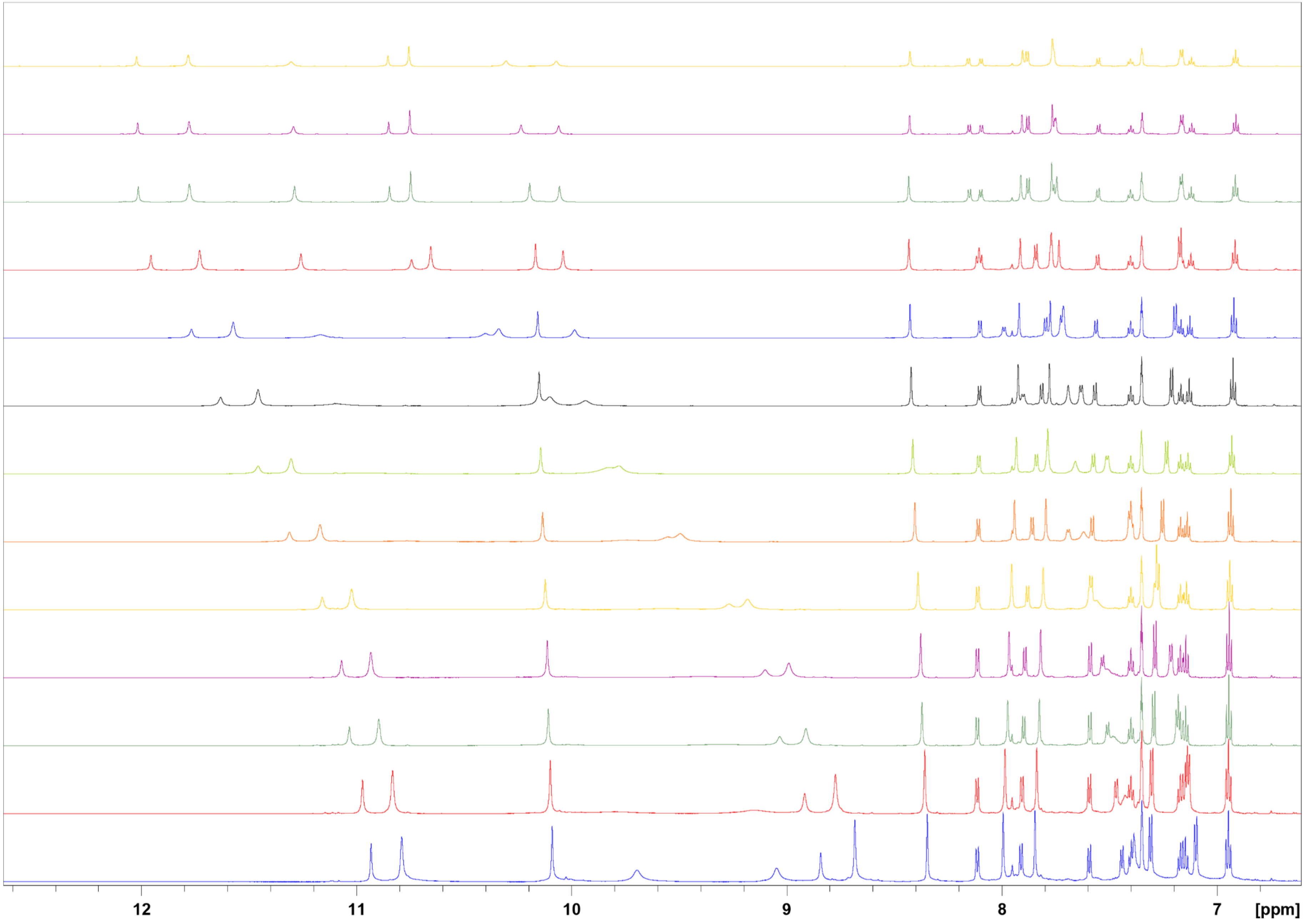

Stacked ^{1}H NMR spectra (700.1 MHz) of a mixture of receptors **MC007**; 1,3-diindolylurea; 1,3-dicarbazolylurea + TBA-acetate

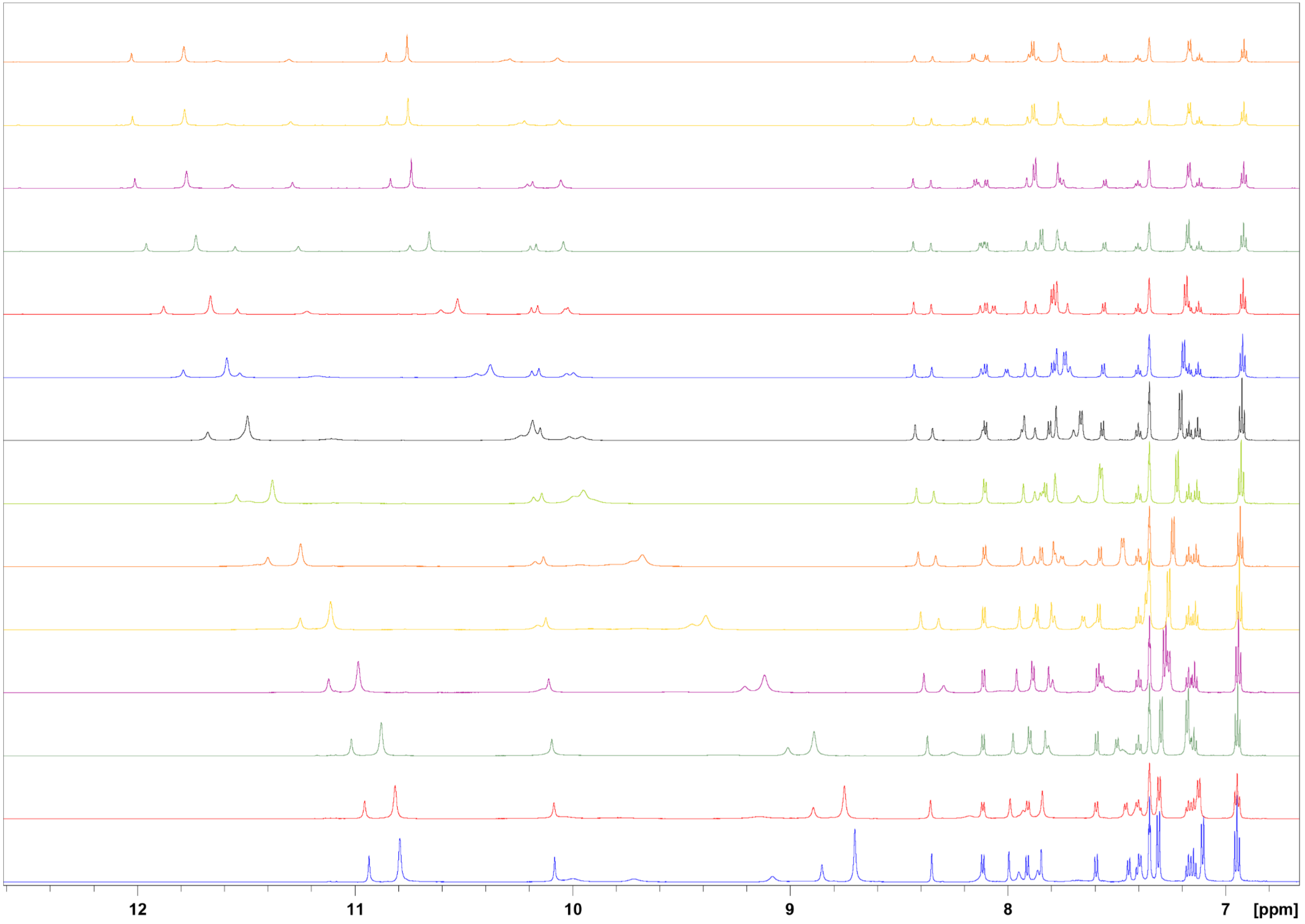

Stacked ^{1}H NMR spectra (700.1 MHz) of a mixture of receptors **MC008**; 1,3-diindolylurea; 1,3-dicarbazolylurea + TBA-acetate

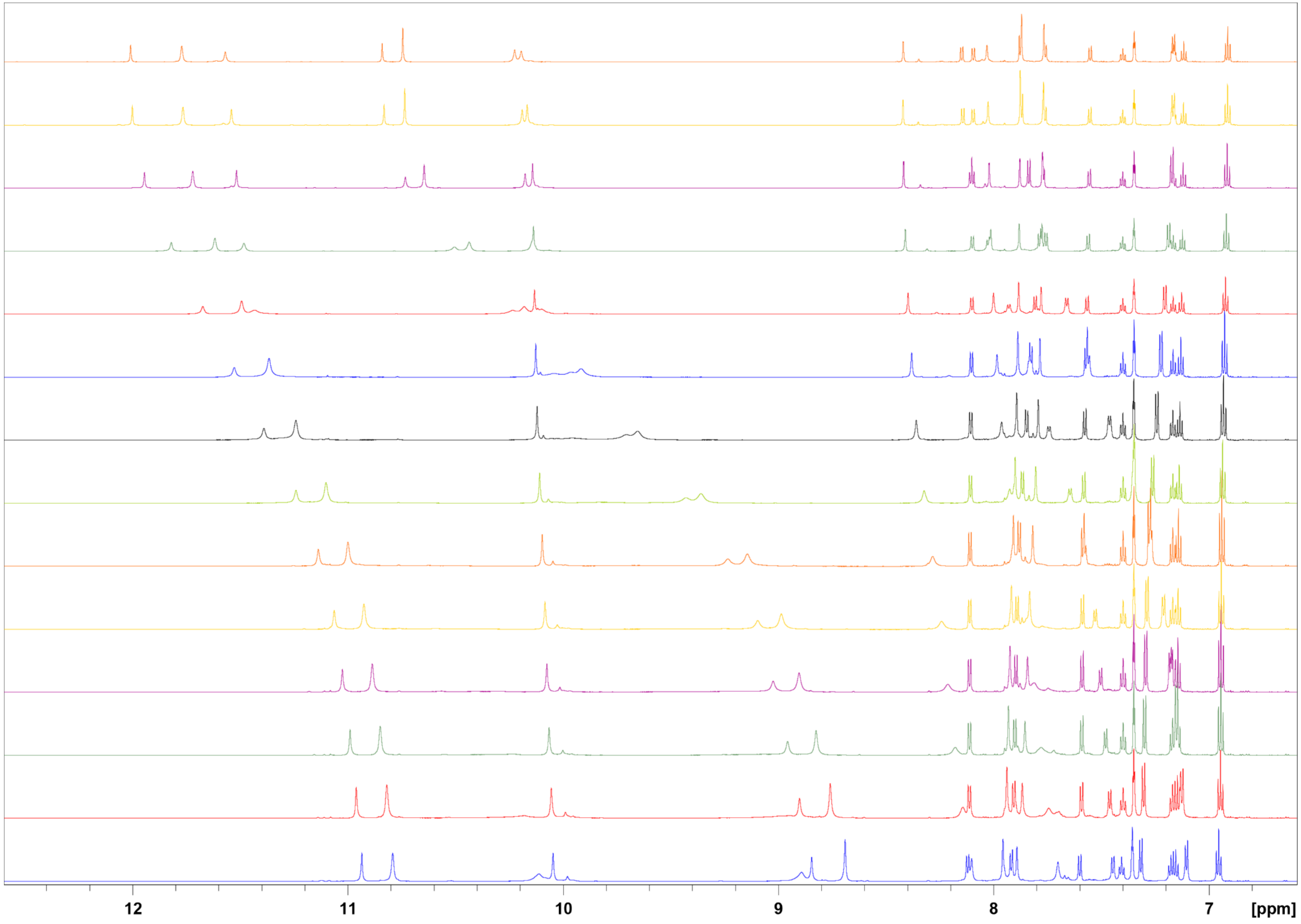

Stacked ^{1}H NMR spectra (700.1 MHz) of a mixture of receptors **MC009**; **MC008**; 1,3-diindolylurea + TBA-acetate

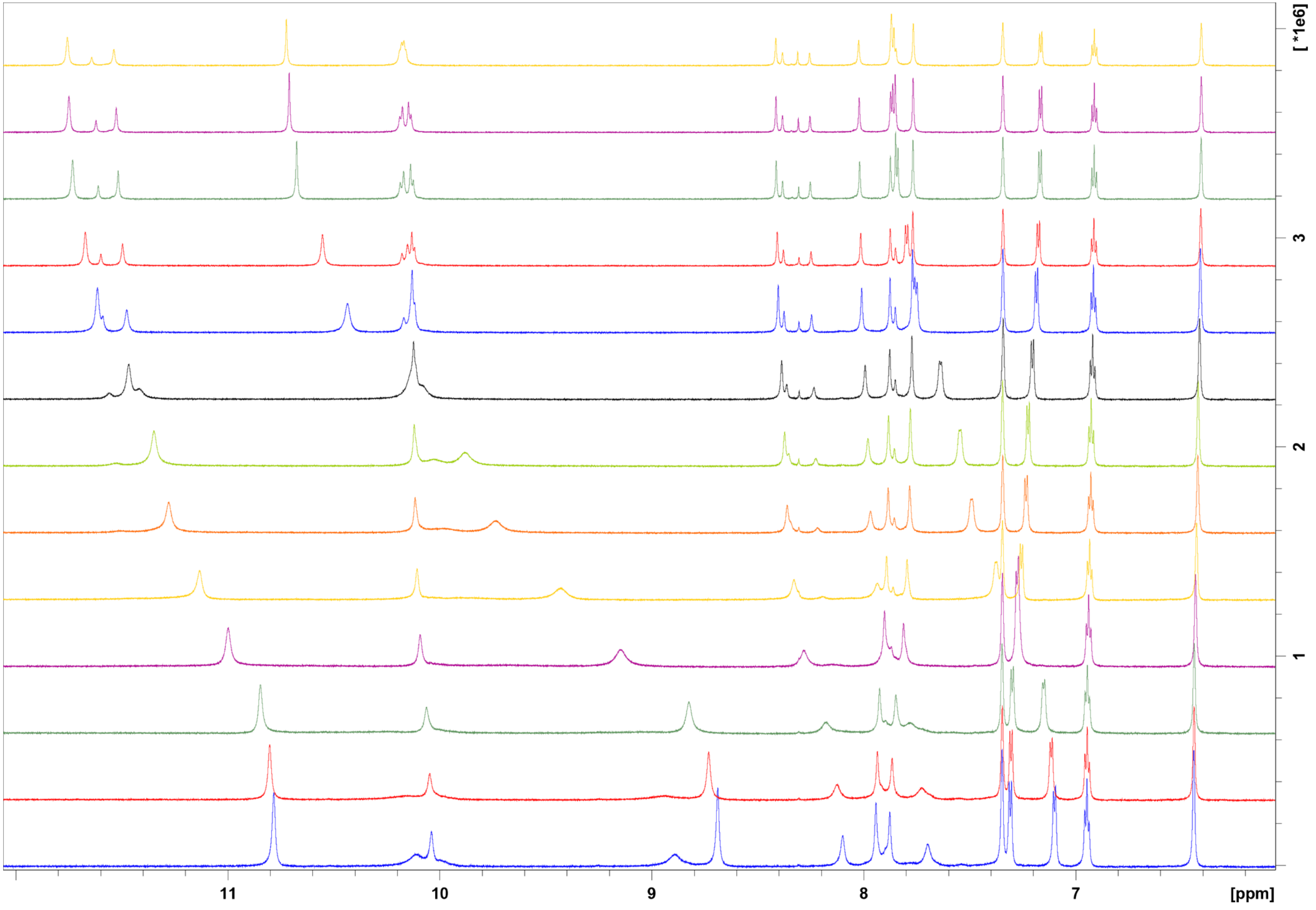

Stacked ^{1}H NMR spectra (700.1 MHz) of a mixture of receptors **MC010**; 1,3-diindolylurea; 1,3-dicarbazolylurea + TBA-acetate

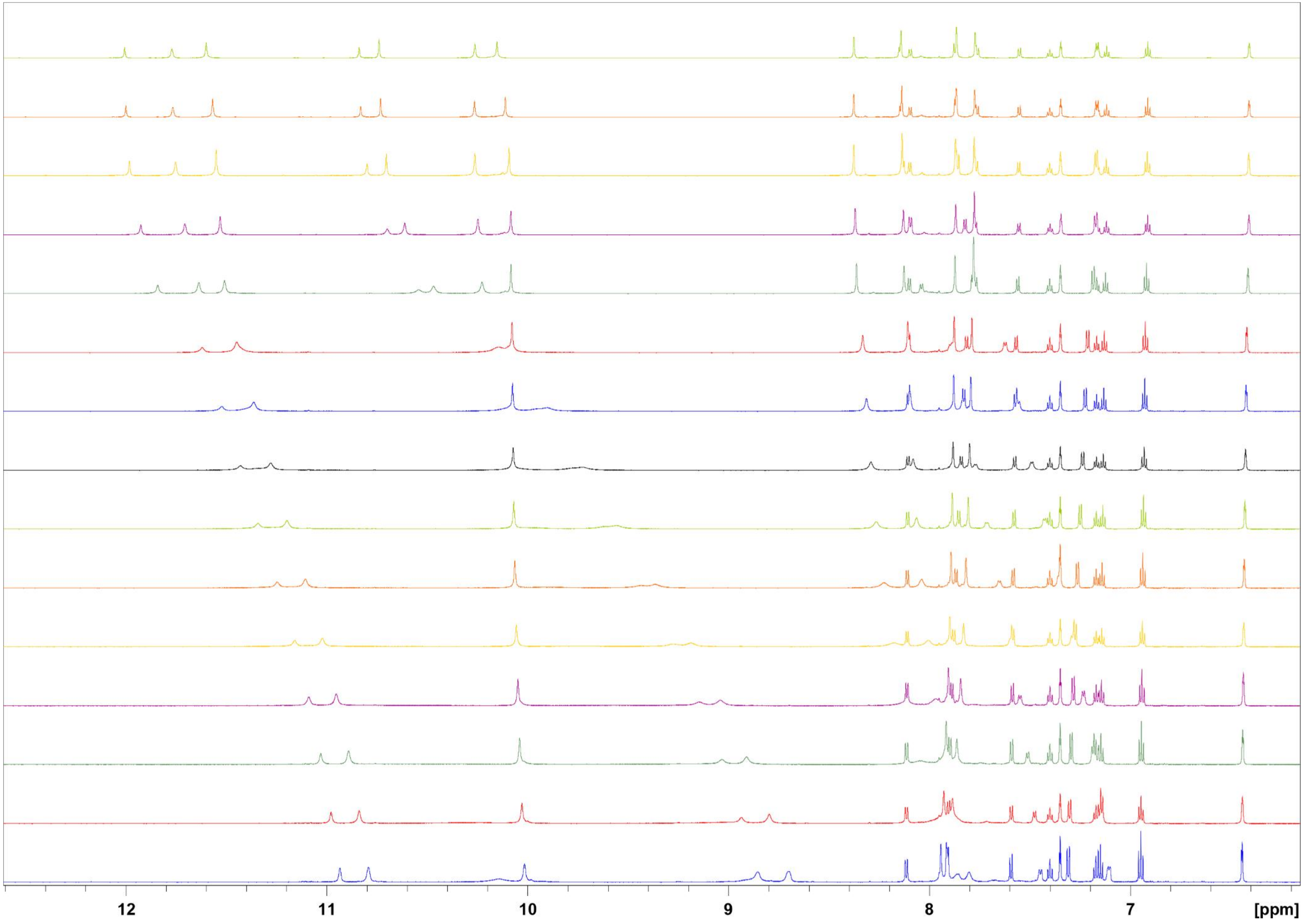

Stacked ^{1}H NMR spectra (700.1 MHz) of a mixture of receptors **MC011**; 1,3-diindolylurea; 1,3-dicarbazolylurea + TBA-acetate

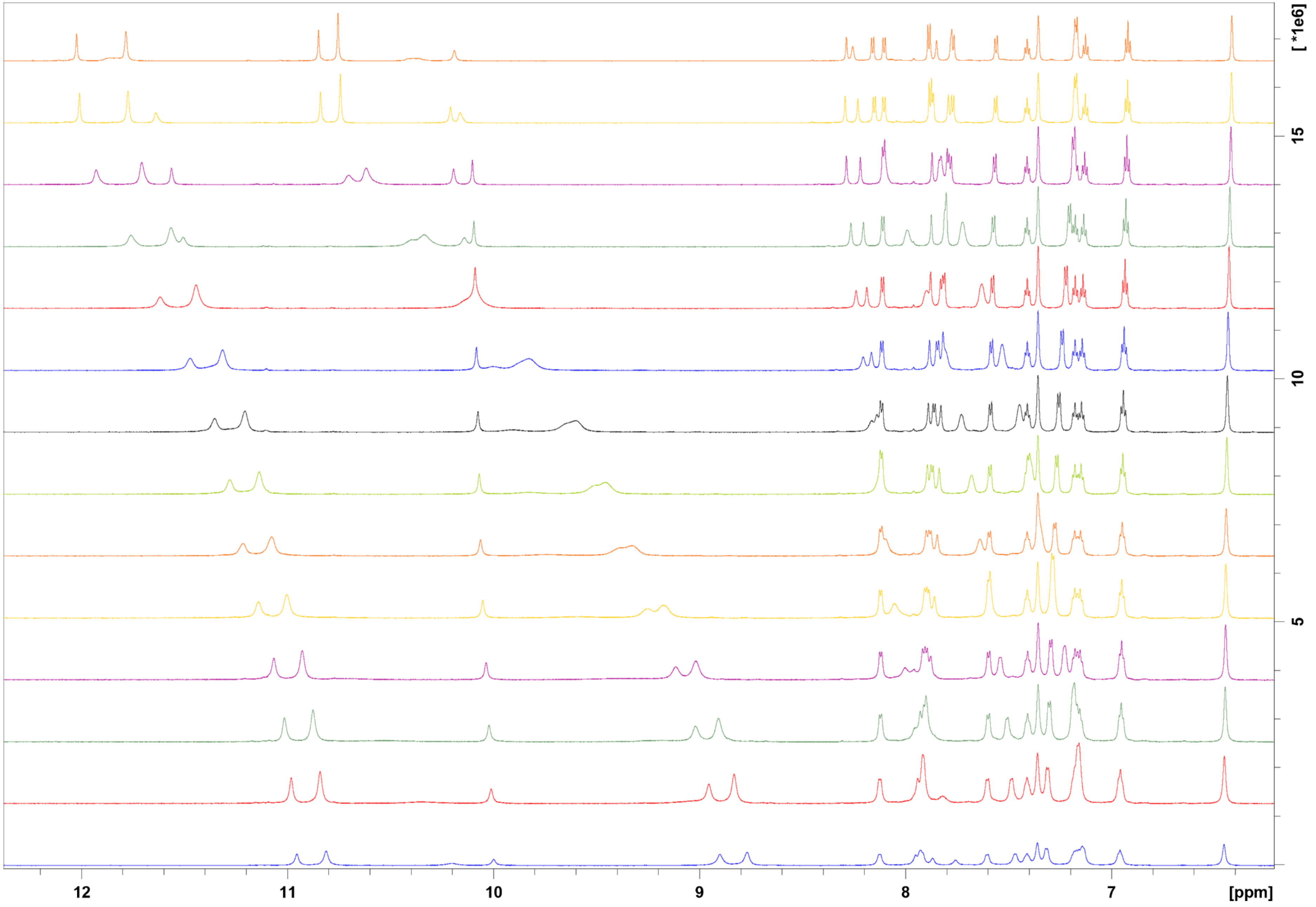

Stacked ^{1}H NMR spectra (700.1 MHz) of a mixture of receptors **MC012**; 1,3-diindolylurea; 1,3-dicarbazolylurea + TBA-acetate

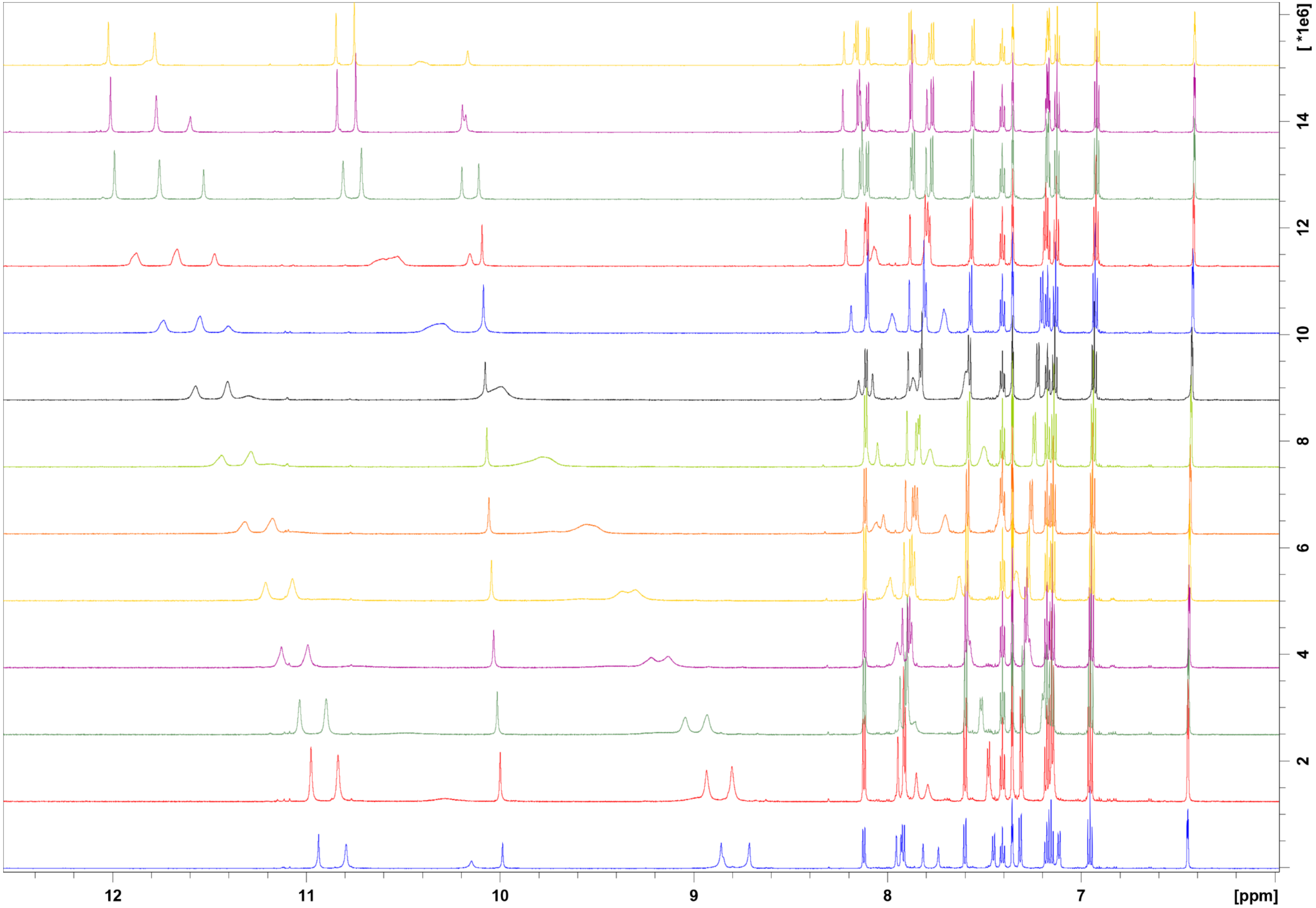

Stacked ^{1}H NMR spectra (700.1 MHz) of a mixture of receptors **MC013**; 1,3-diindolylurea; 1,3-dicarbazolylurea + TBA-acetate

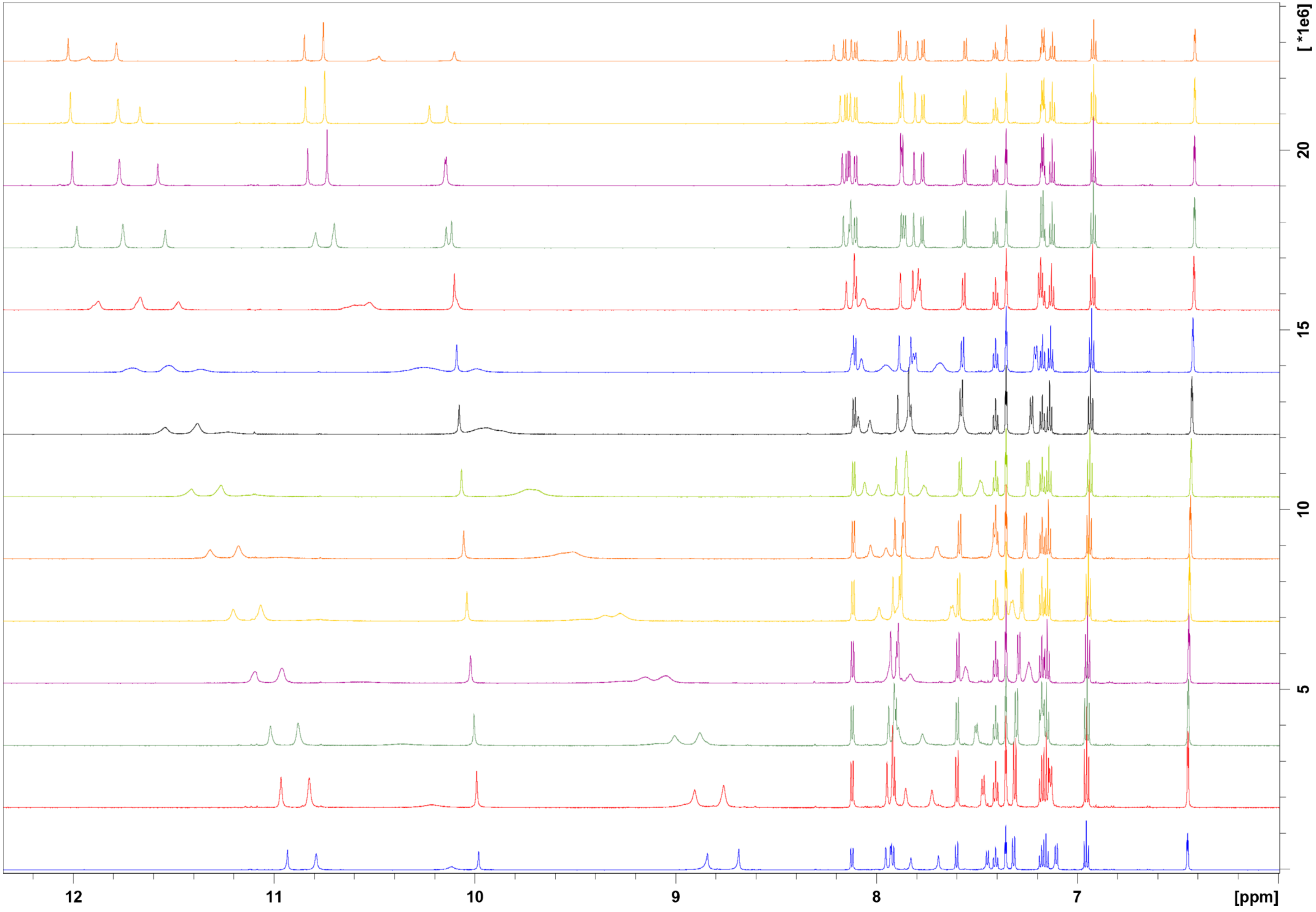

Stacked ^{1}H NMR spectra (700.1 MHz) of a mixture of receptors **MC014**; 1,3-diindolylurea; 1,3-dicarbazolylurea + TBA-acetate

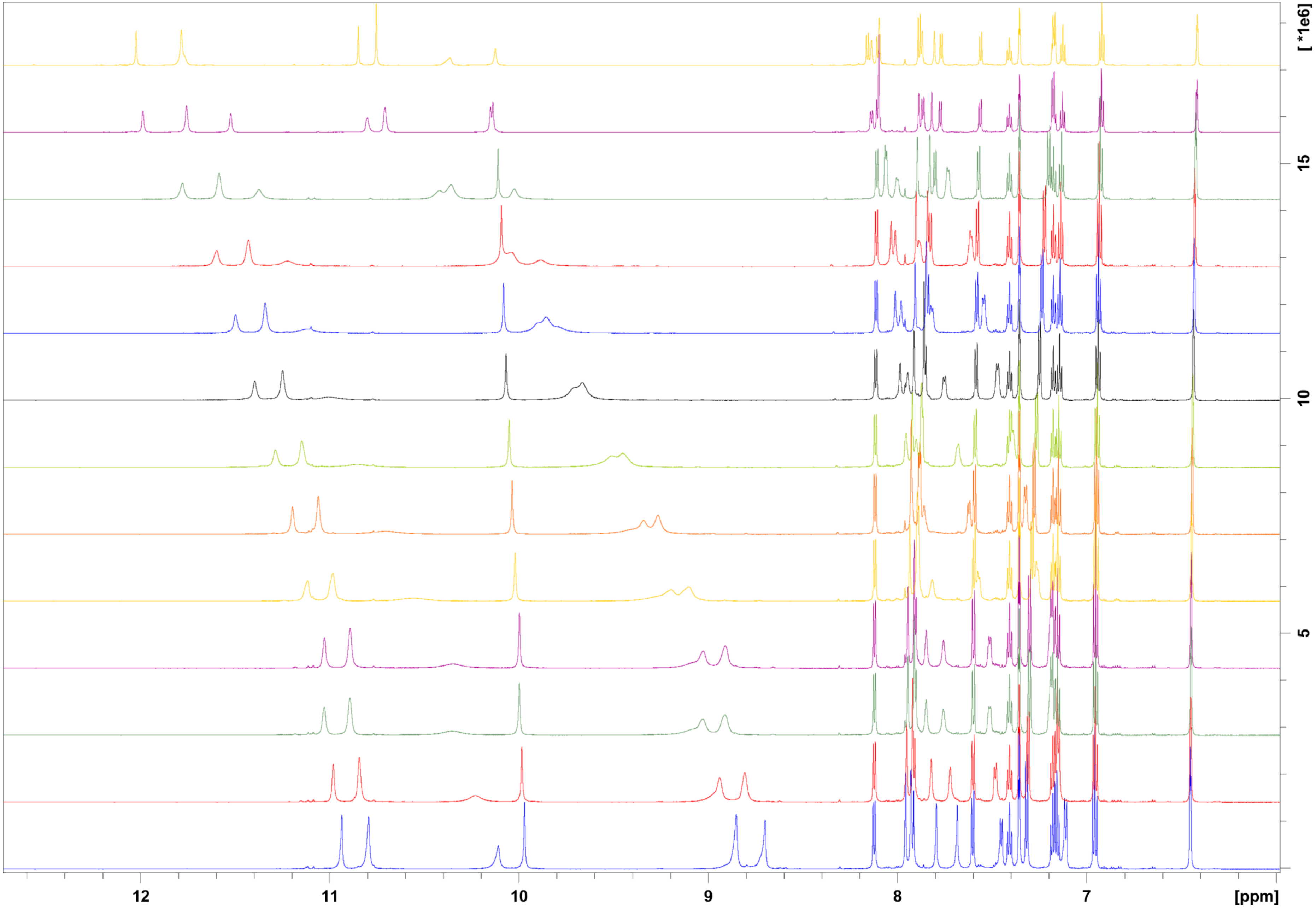

Stacked ^{1}H NMR spectra (700.1 MHz) of a mixture of receptors **CZ016**; **MC002**; 2,7-$(COOBu)_2$-indolocarbazole; 2,9-$(COOBu)_2$-indolocarbazole + TBA-benzoate

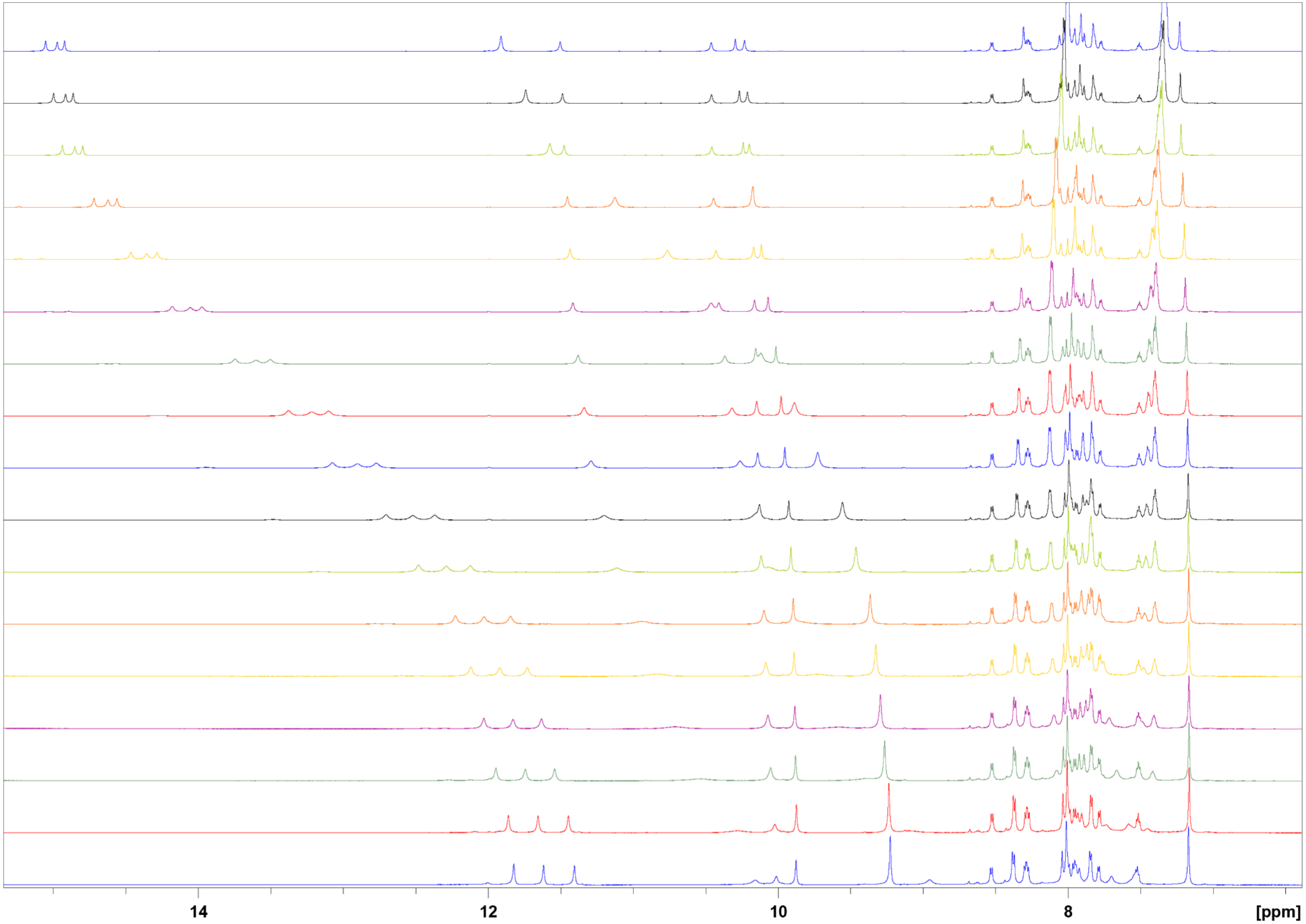

Stacked ^{1}H NMR spectra (700.1 MHz) of a mixture of receptors **MC001**; 2,7-$(COOBu)_2$-indolocarbazole; 2,9-$(COOBu)_2$-indolocarbazole + TBA-benzoate

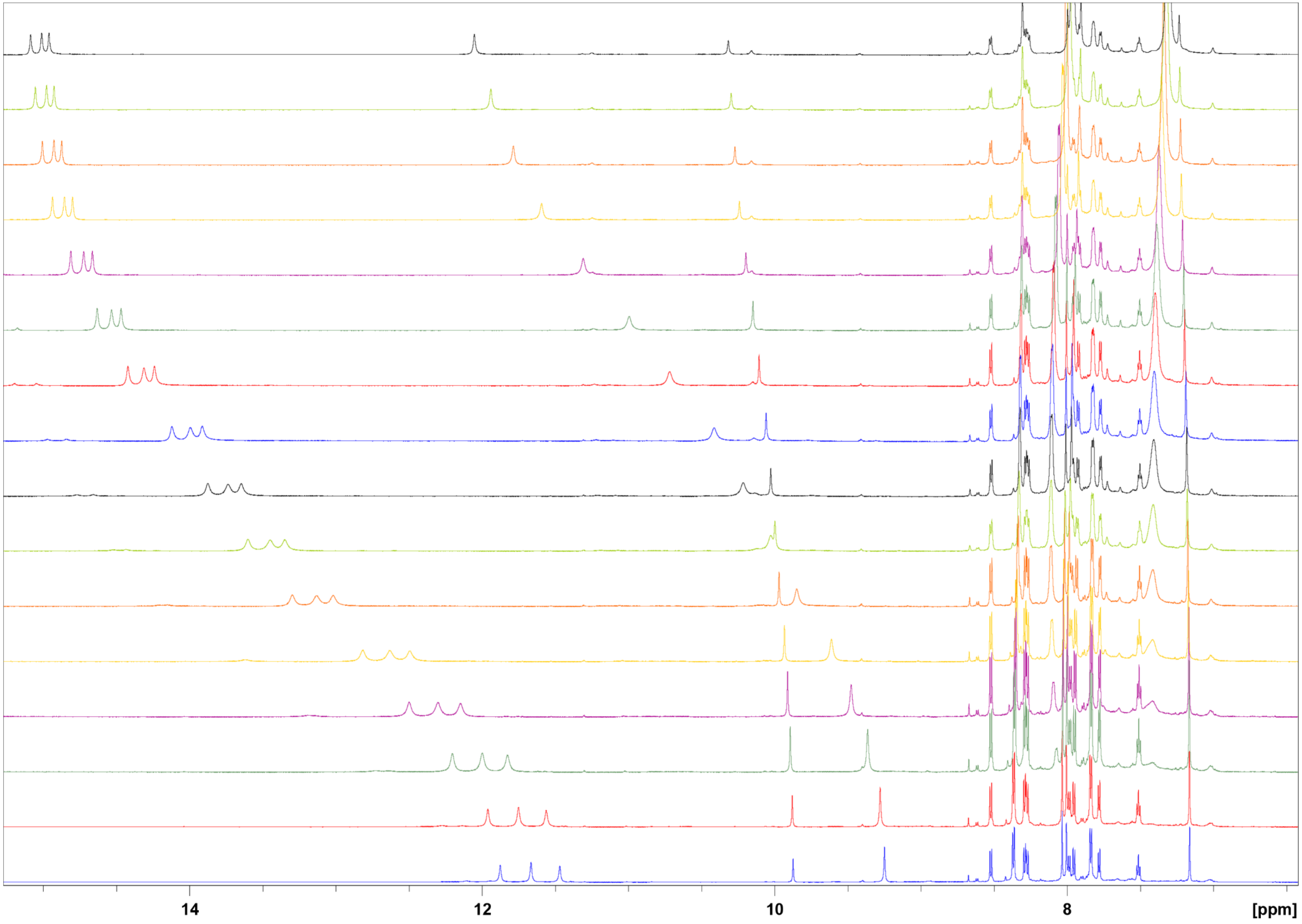

Stacked ^{1}H NMR spectra (700.1 MHz) of a mixture of receptors **MC003**; 1,3-diindolylurea; 1,3-dicarbazolylurea + TBA-benzoate

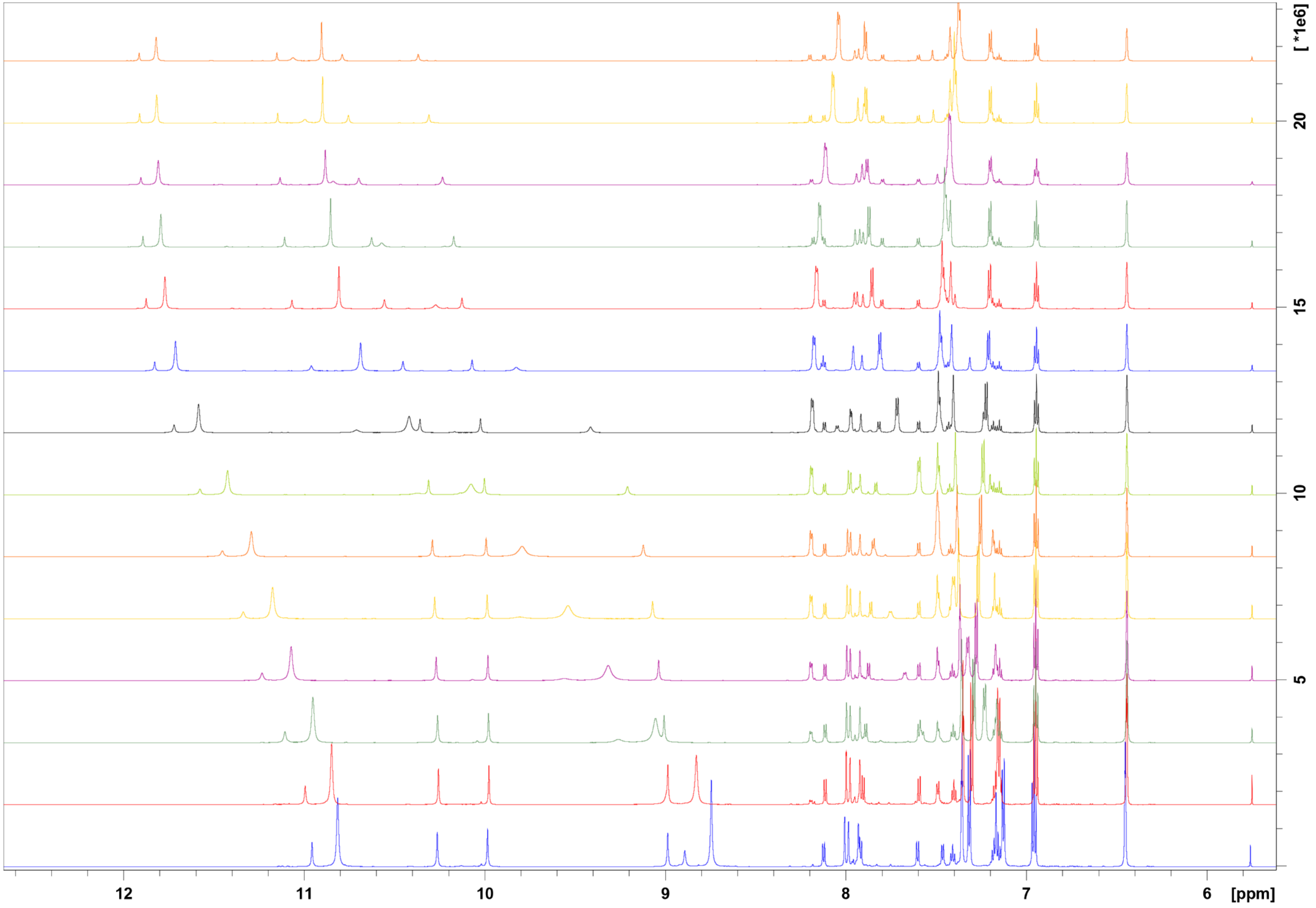

Stacked ^{1}H NMR spectra (700.1 MHz) of a mixture of receptors **MC004**; 1,3-diindolylurea; 1,3-dicarbazolylurea + TBA-benzoate

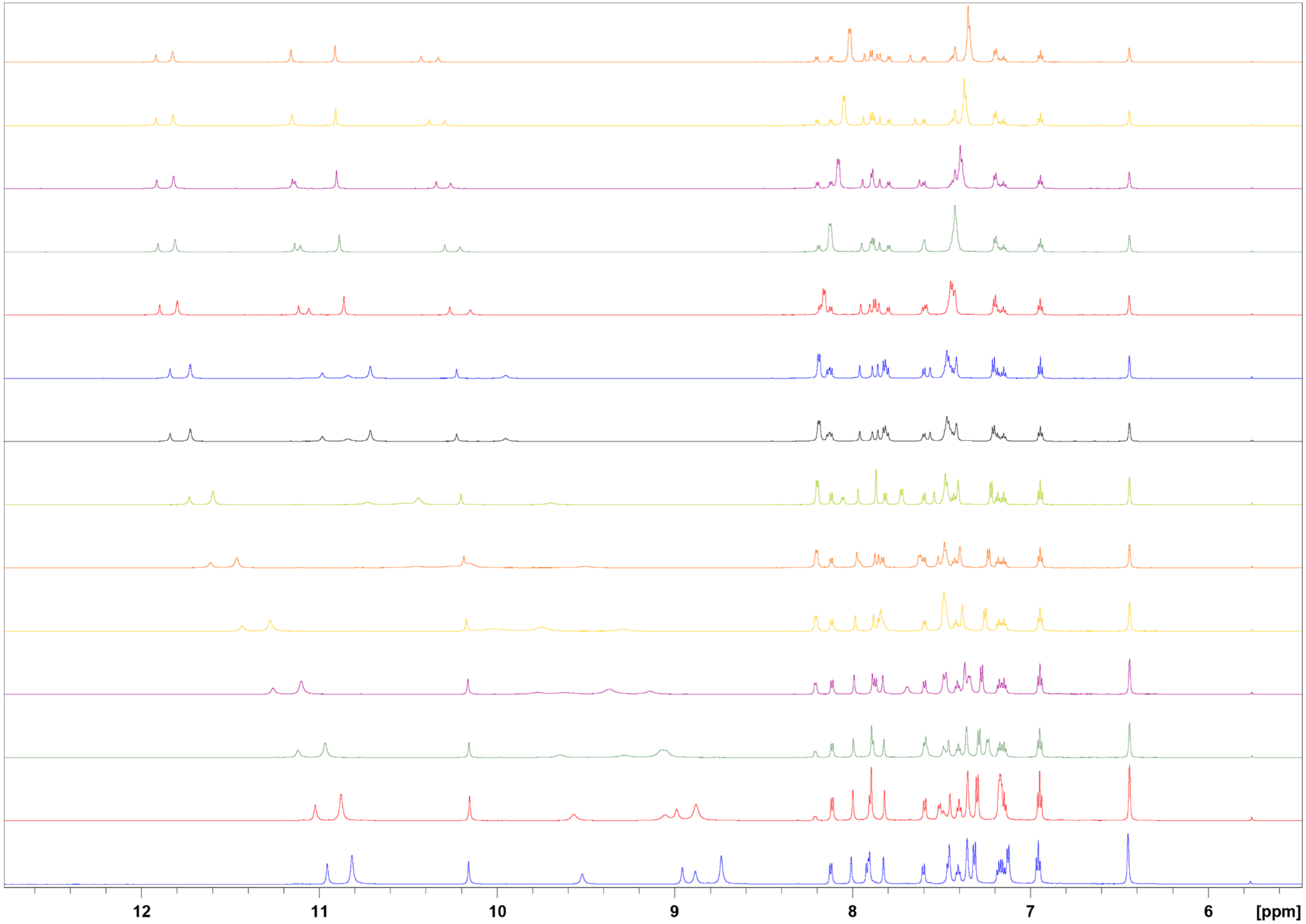

Stacked ^{1}H NMR spectra (700.1 MHz) of a mixture of receptors **MC005**; 1,3-diindolylurea; 1,3-dicarbazolylurea + TBA-benzoate

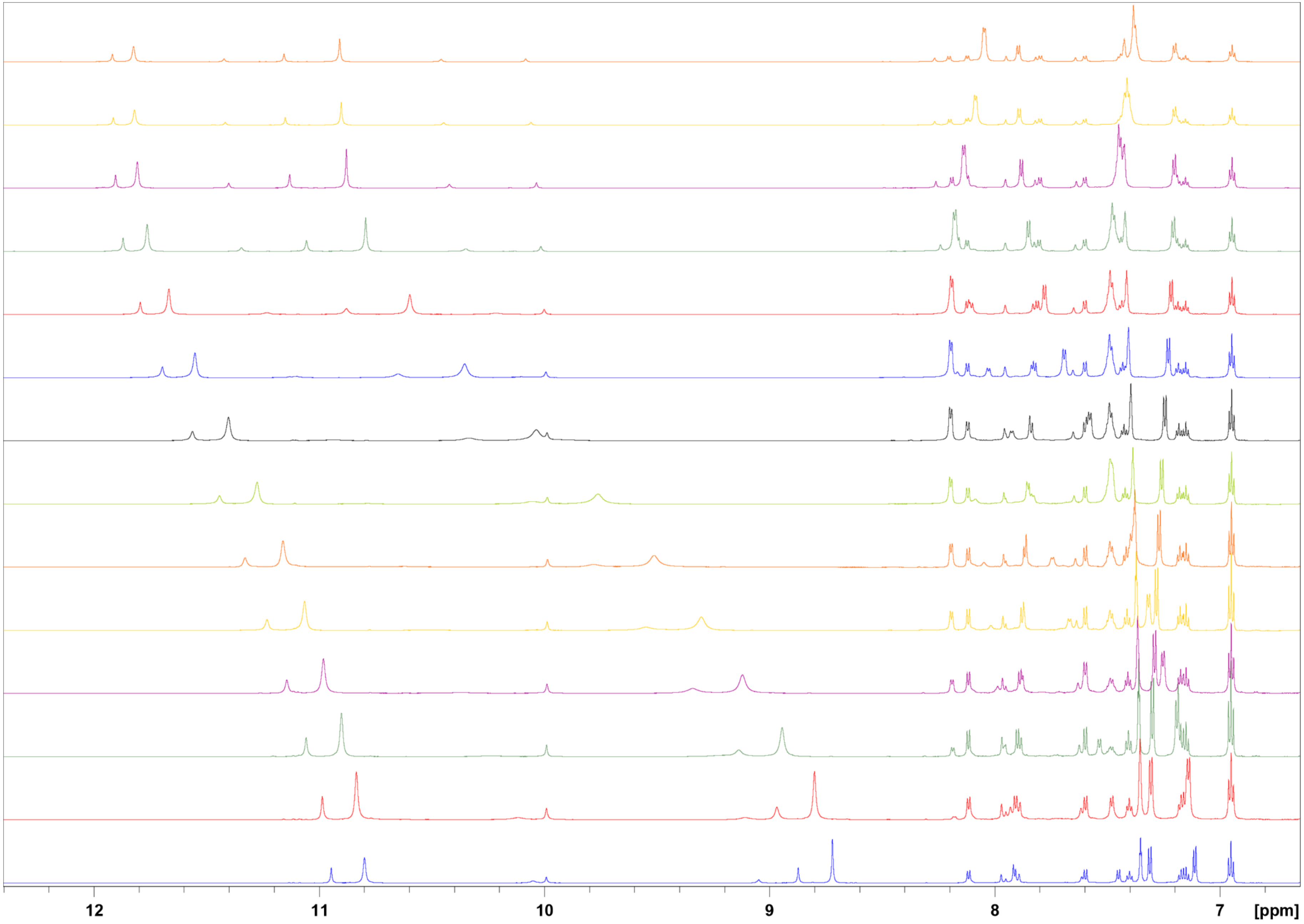

Stacked ^{1}H NMR spectra (700.1 MHz) of a mixture of receptors **MC006**; 1,3-diindolylurea; 1,3-dicarbazolylurea + TBA-benzoate

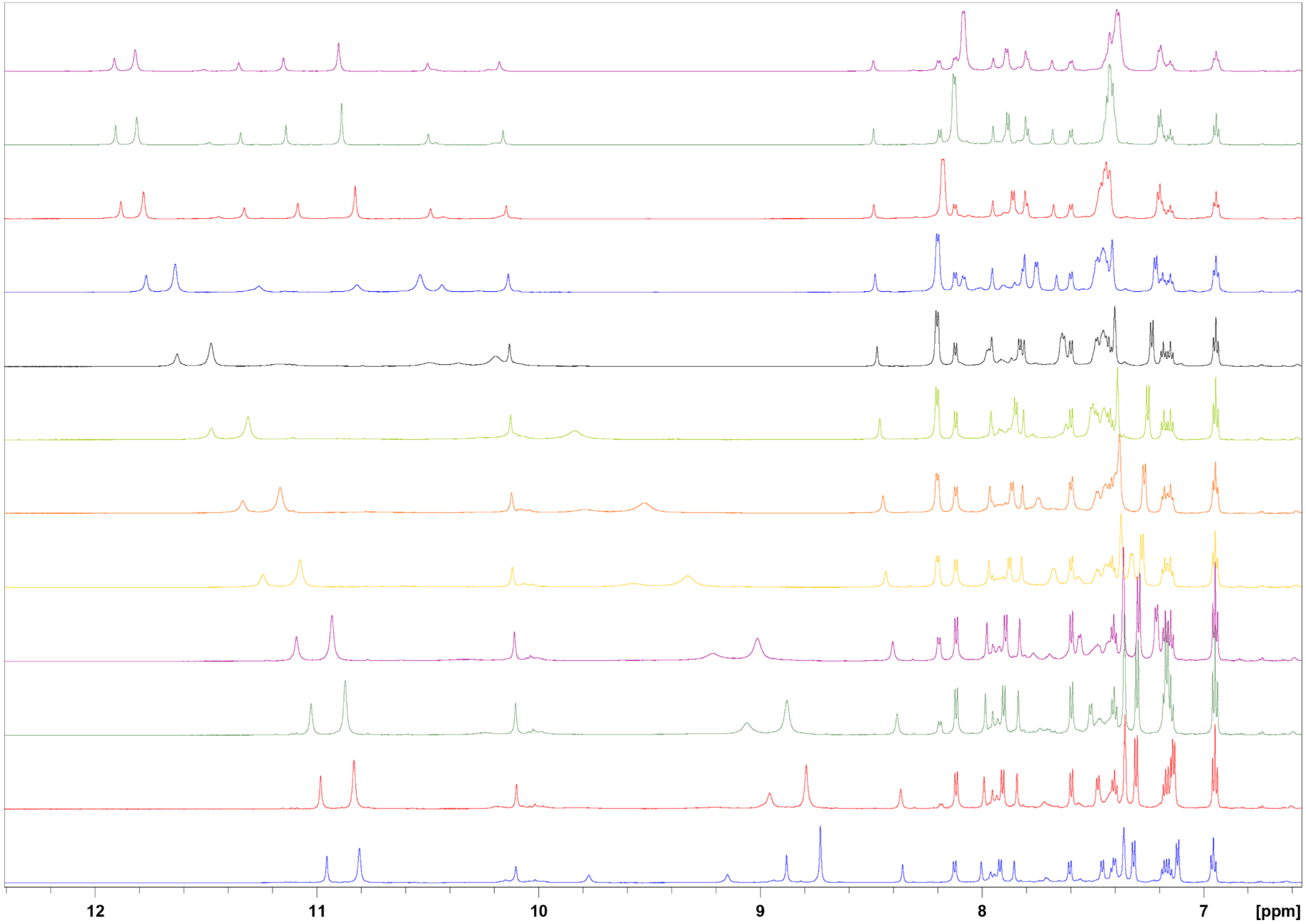

Stacked ^{1}H NMR spectra (700.1 MHz) of a mixture of receptors **MC007**; 1,3-diindolylurea; 1,3-dicarbazolylurea + TBA-benzoate

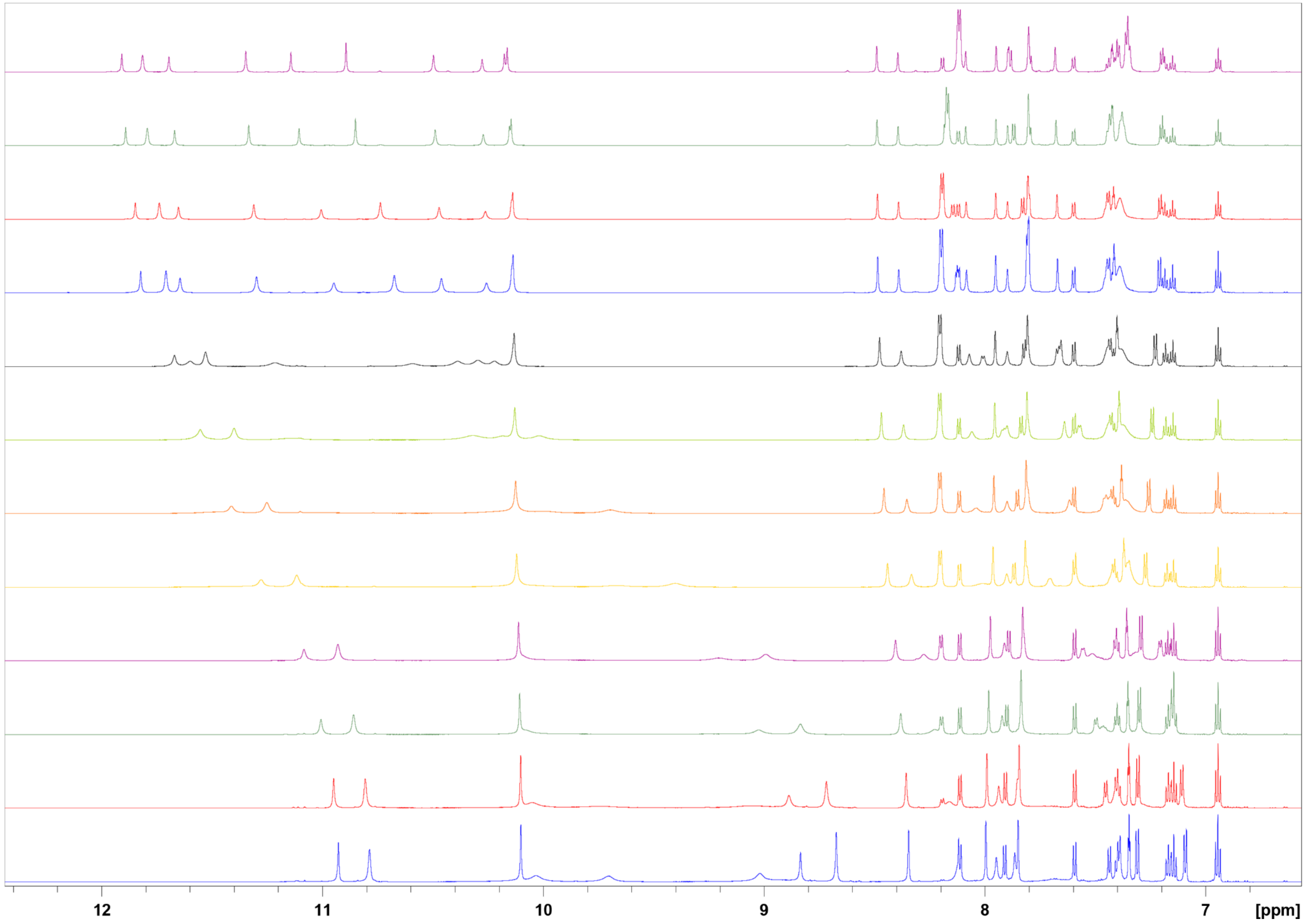

Stacked ^{1}H NMR spectra (700.1 MHz) of a mixture of receptors **MC008**; 1,3-diindolylurea; 1,3-dicarbazolylurea + TBA-benzoate

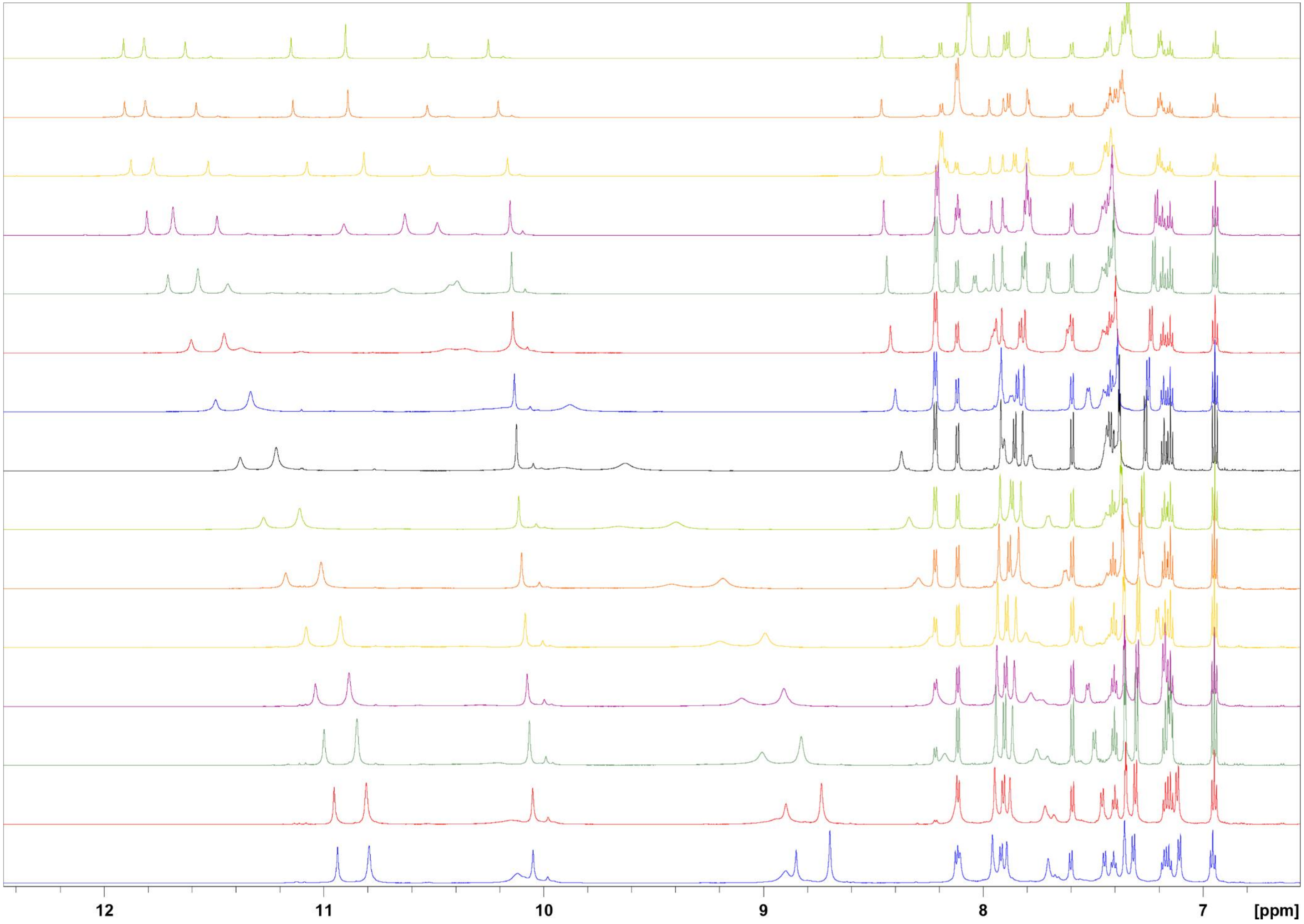

Stacked ^{1}H NMR spectra (700.1 MHz) of a mixture of receptors **MC009**; 1,3-diindolylurea; 1,3-dicarbazolylurea + TBA-benzoate

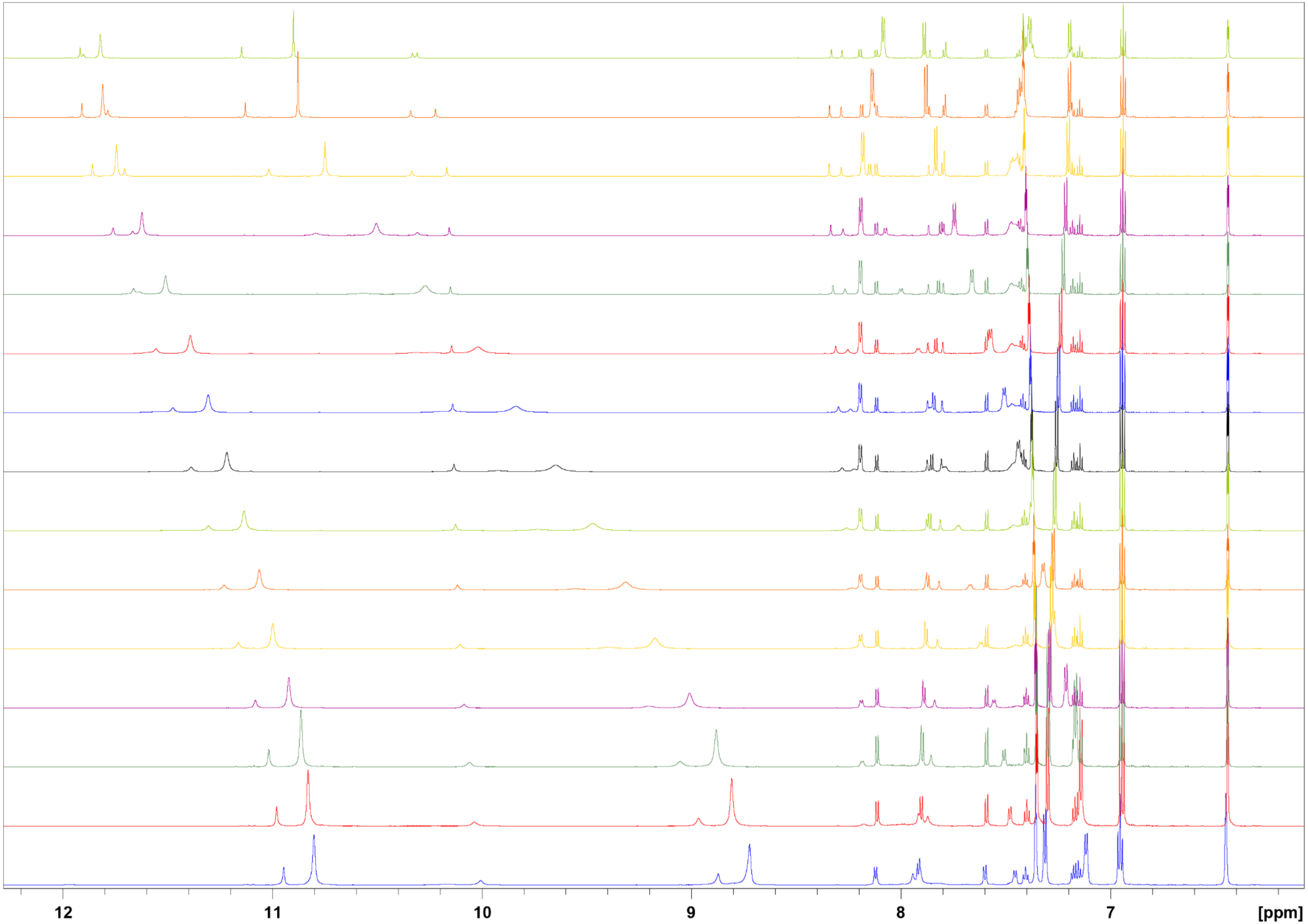

Stacked ^{1}H NMR spectra (700.1 MHz) of a mixture of receptors **MC010**; 1,3-diindolylurea; 1,3-dicarbazolylurea + TBA-benzoate

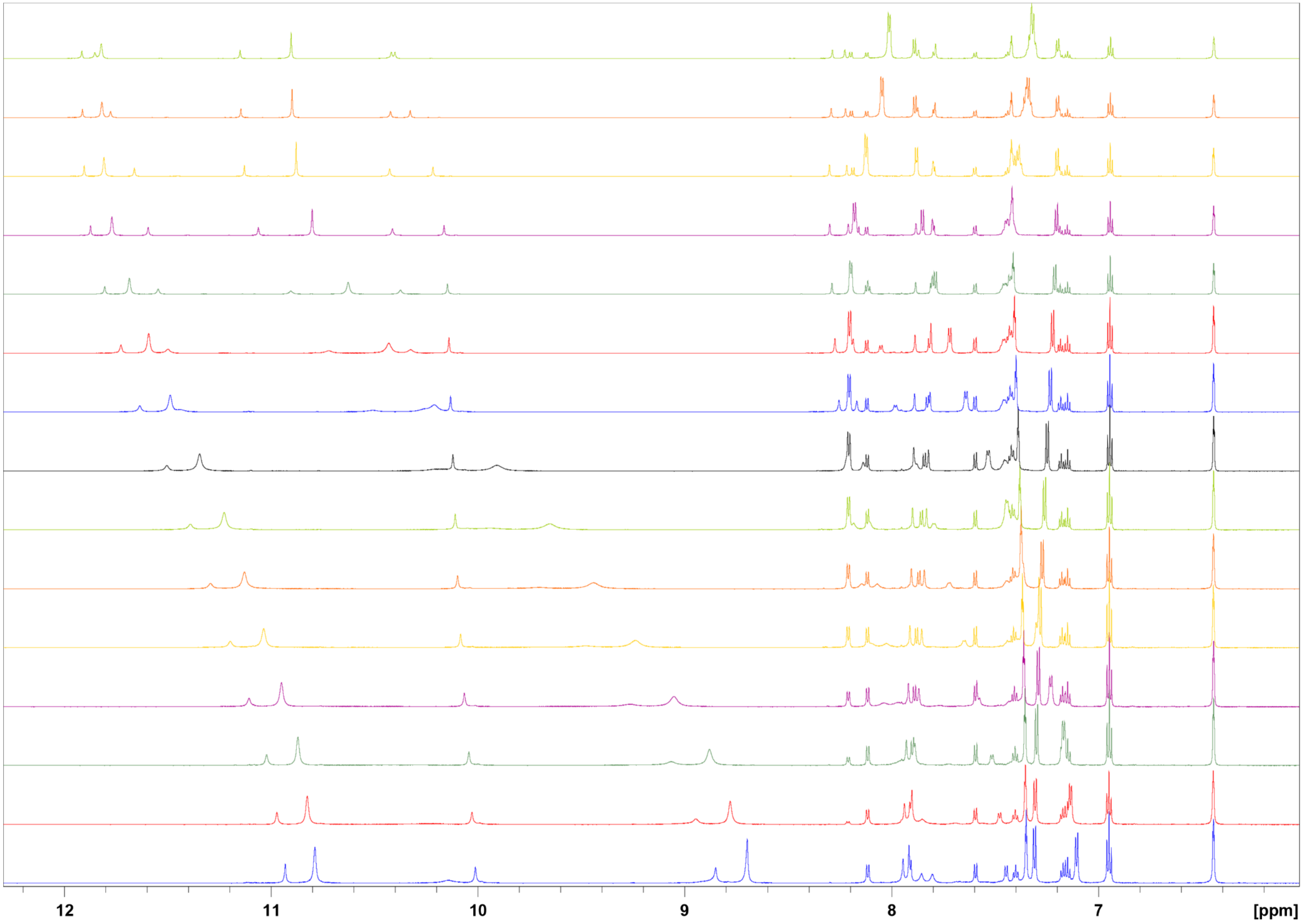

Stacked ^{1}H NMR spectra (700.1 MHz) of a mixture of receptors **MC011**; 1,3-diindolylurea; 1,3-dicarbazolylurea + TBA-benzoate

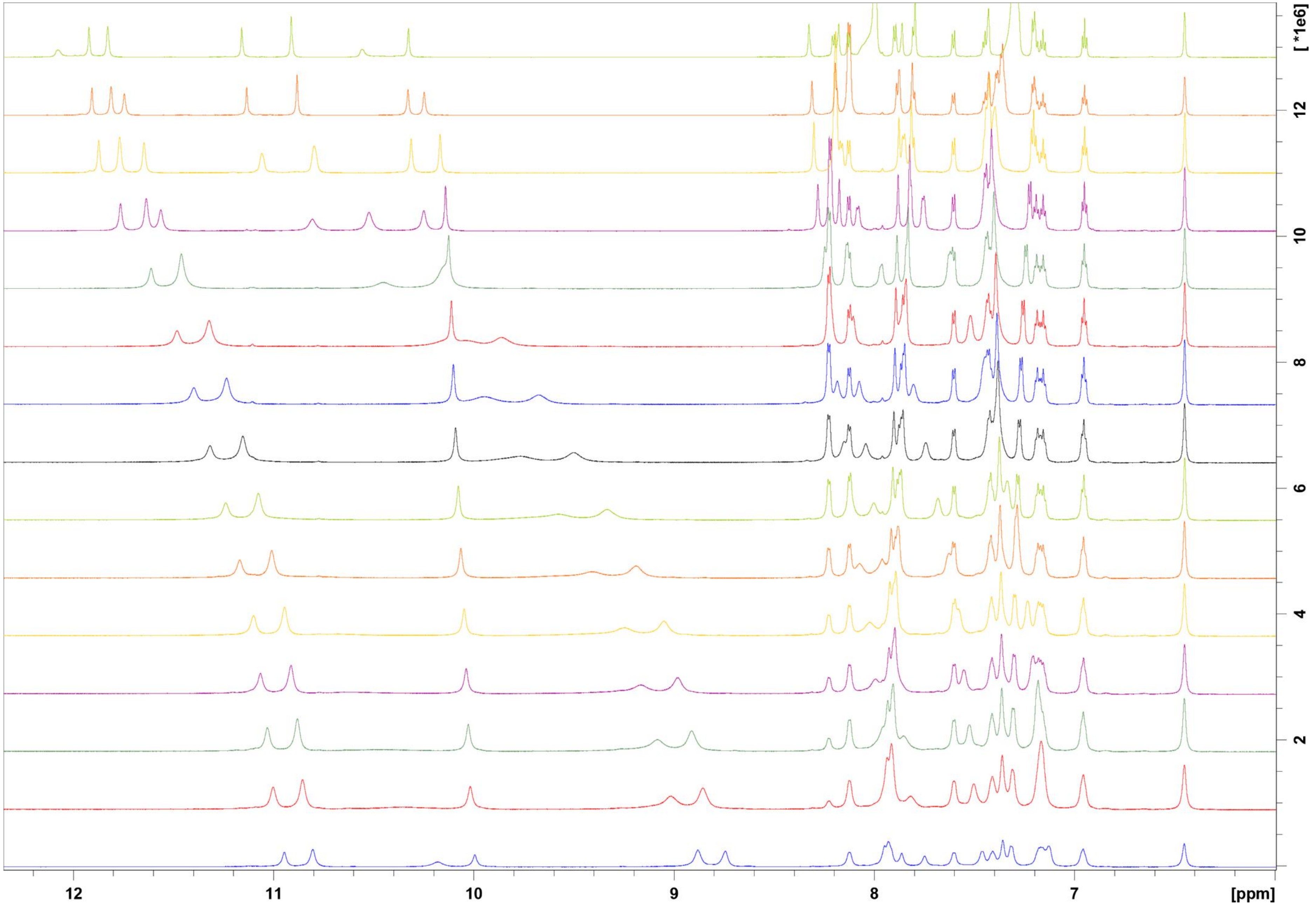

Stacked ^{1}H NMR spectra (700.1 MHz) of a mixture of receptors **MC012**; 1,3-diindolylurea; 1,3-dicarbazolylurea + TBA-benzoate

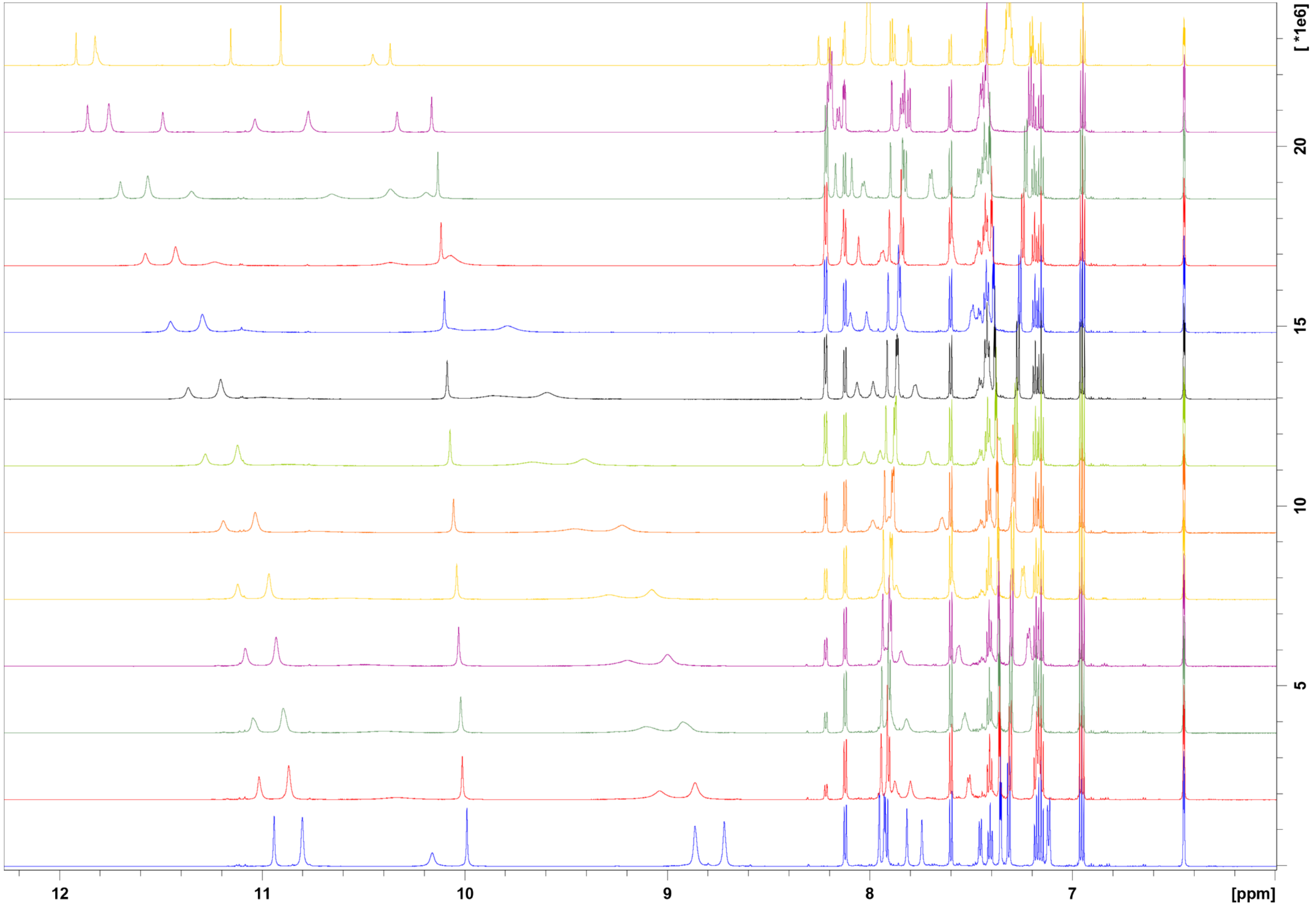

Stacked ^1H NMR spectra (700.1 MHz) of a mixture of receptors **MC013**; 1,3-diindolylurea; 1,3-dicarbazolylurea + TBA-benzoate

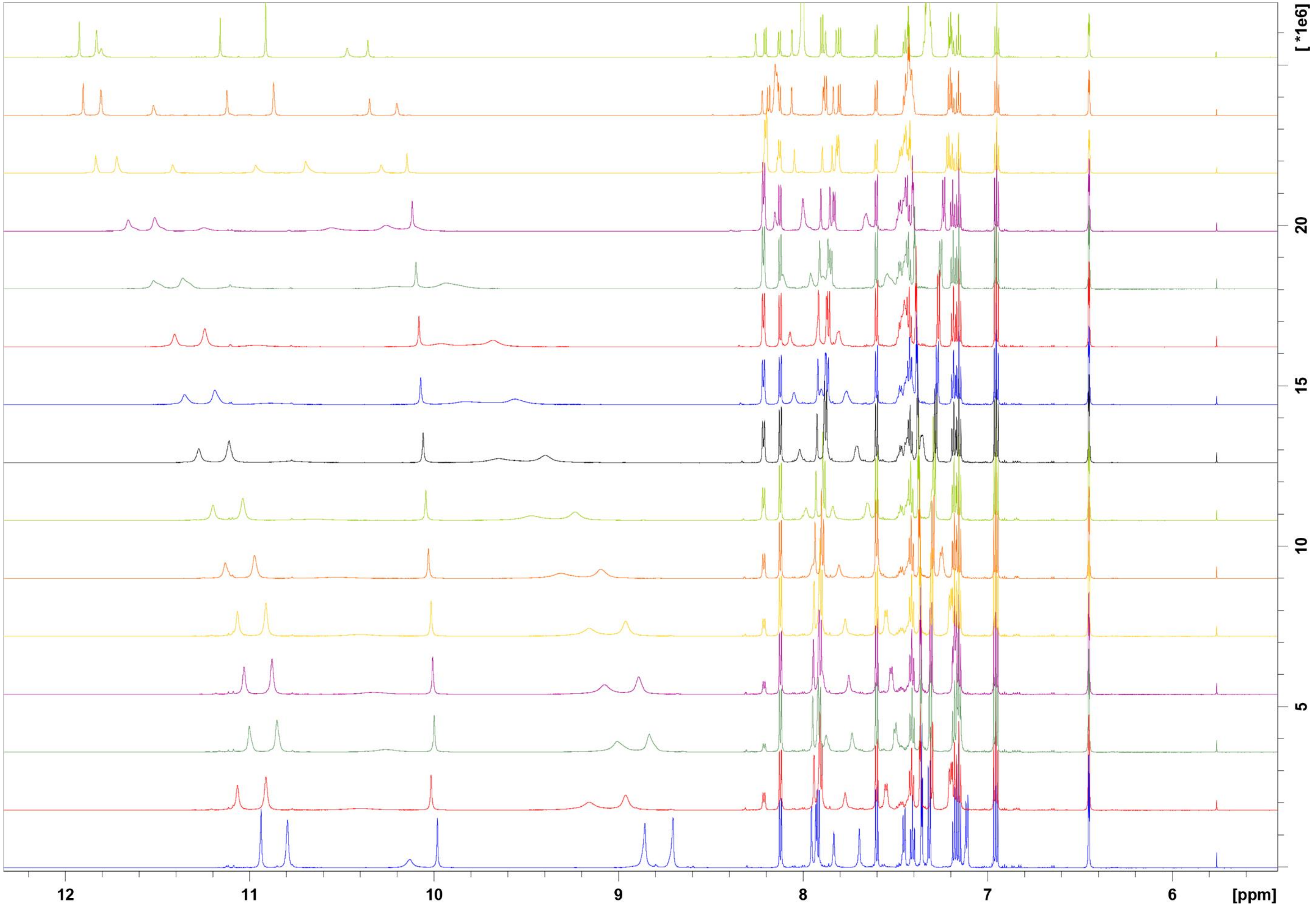

Stacked ^{1}H NMR spectra (700.1 MHz) of a mixture of receptors **MC014**; 1,3-diindolylurea; 1,3-dicarbazolylurea + TBA-benzoate

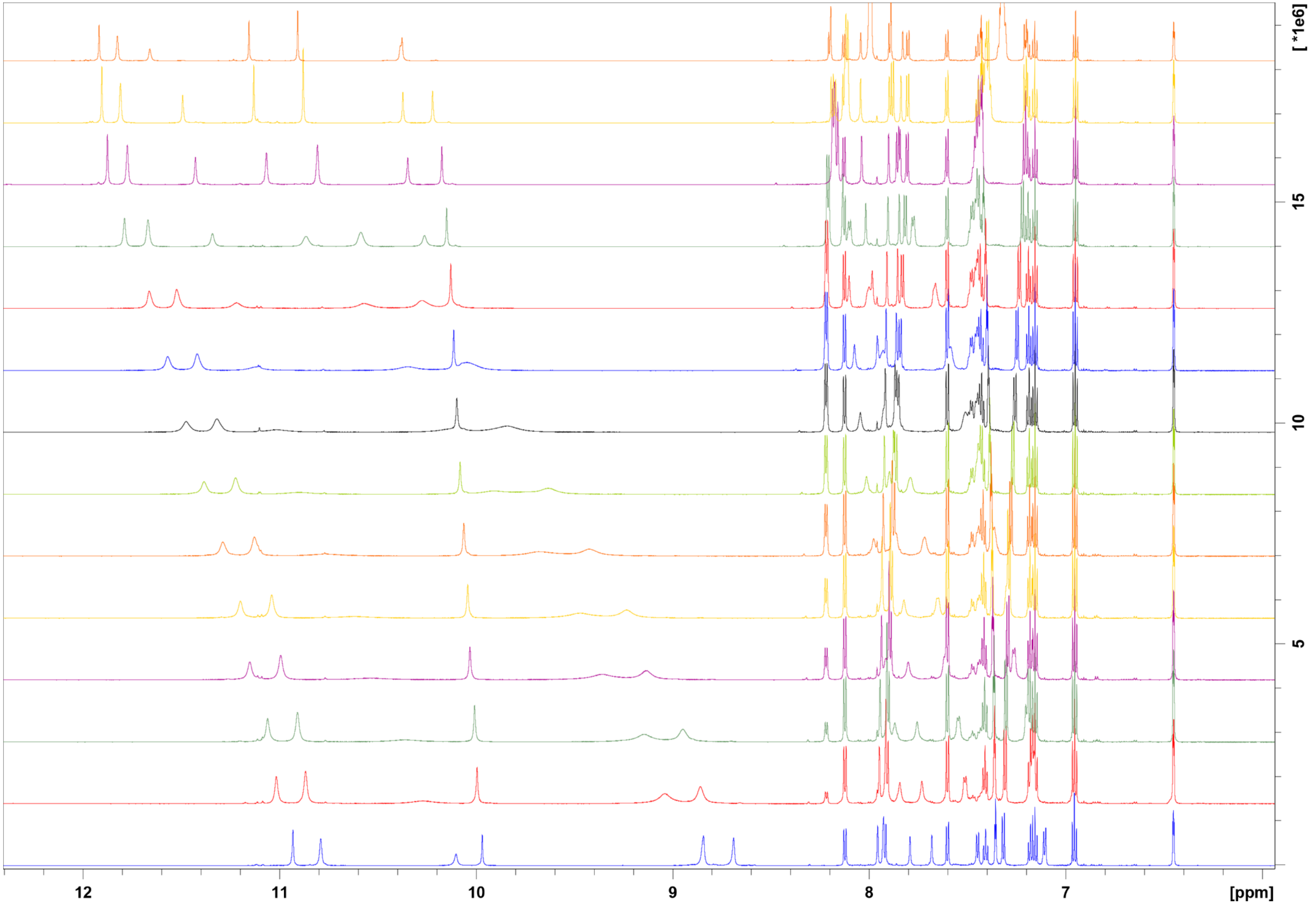

Stacked ^{1}H NMR spectra (700.1 MHz) of a mixture of receptors **CZ016**; 2,7-(COOBu)$_2$-indolocarbazole; 2,9-(COOBu)$_2$-indolocarbazole + TBA-formate

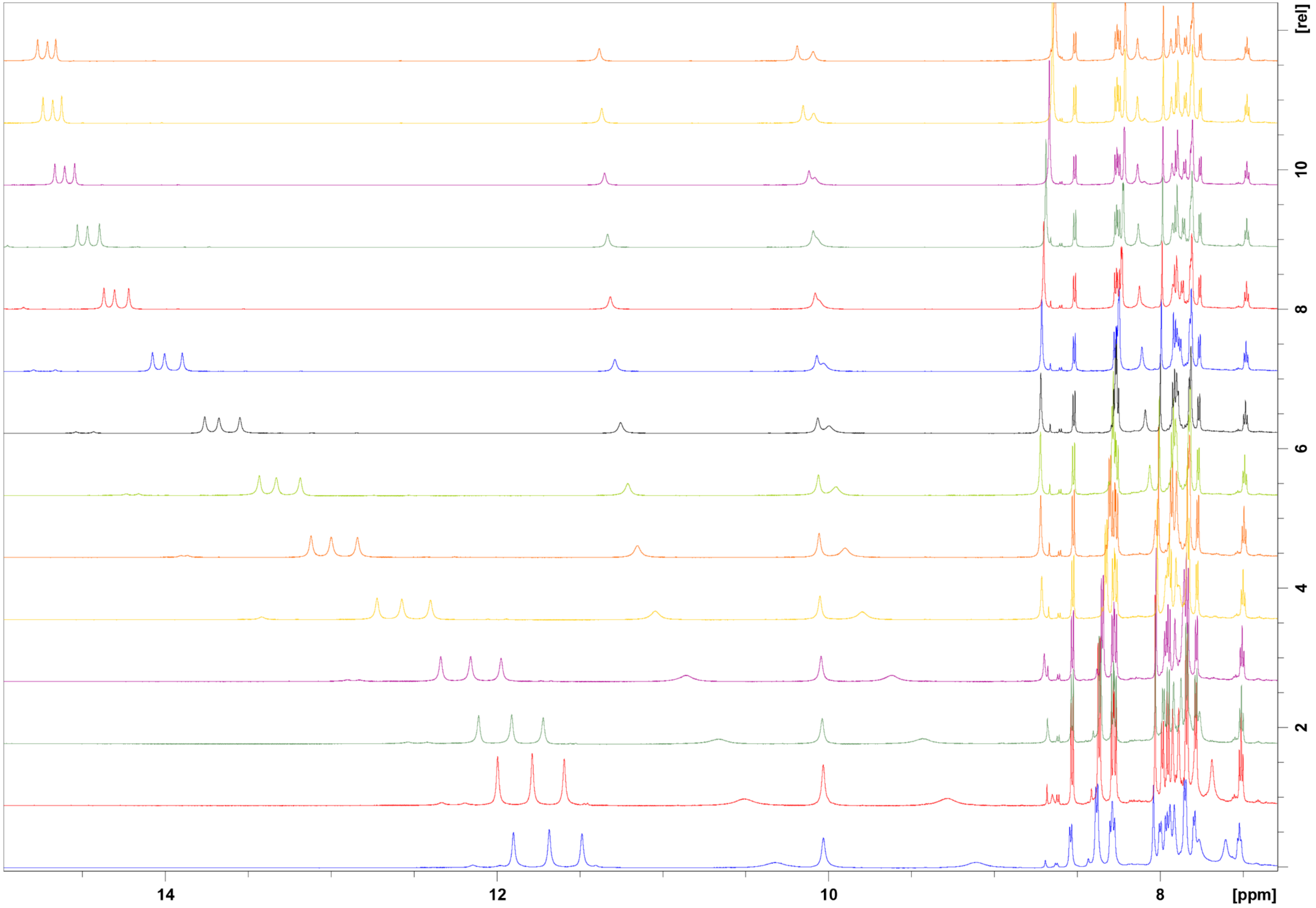

Stacked ^{1}H NMR spectra (700.1 MHz) of a mixture of receptors **MC001**; **MC004**; 1,3-diindolylurea + TBA-formate

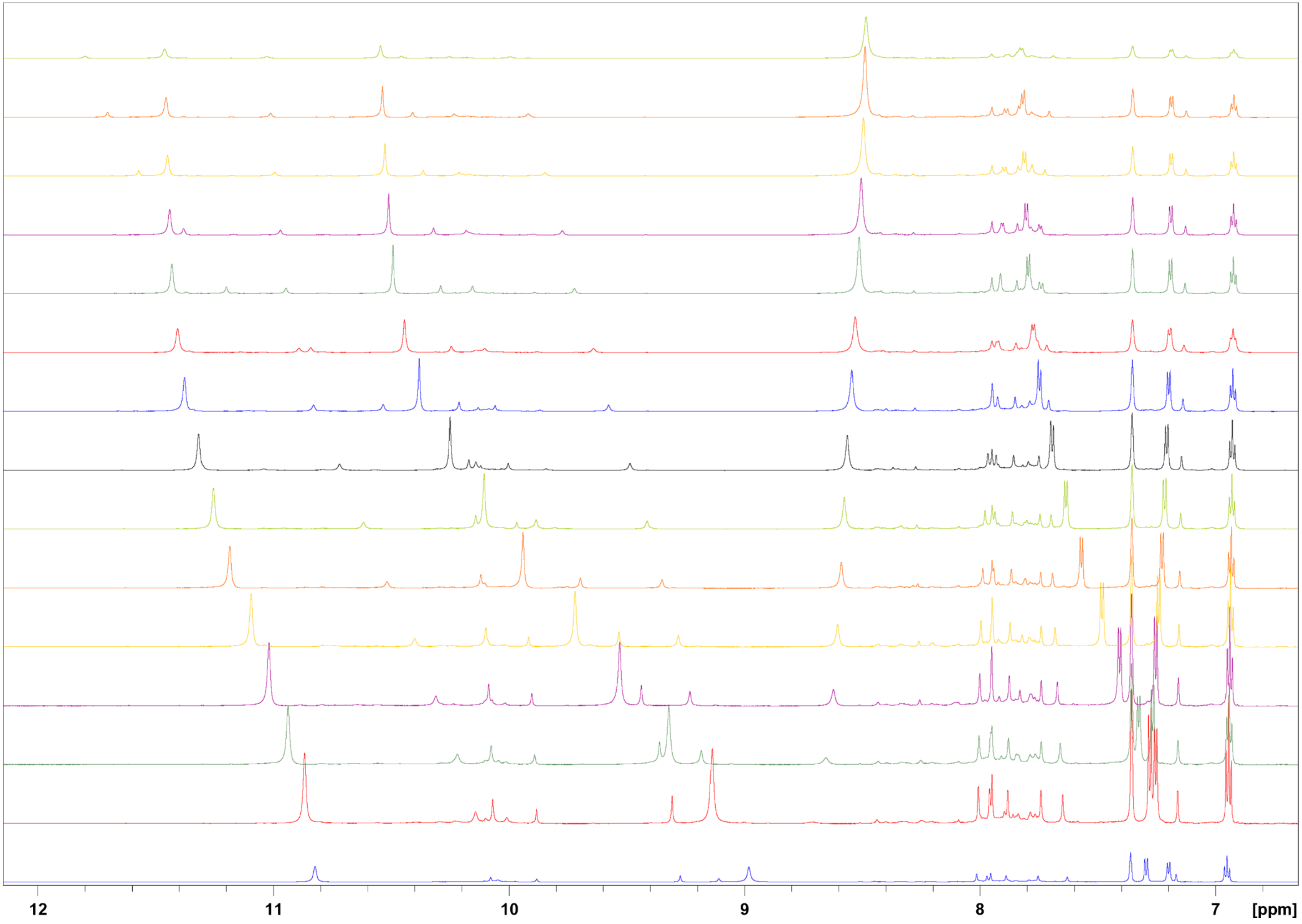

Stacked ^{1}H NMR spectra (700.1 MHz) of a mixture of receptors **MC002**; 1,3-diindolylurea; 1,3-dicarbazolylurea + TBA-formate

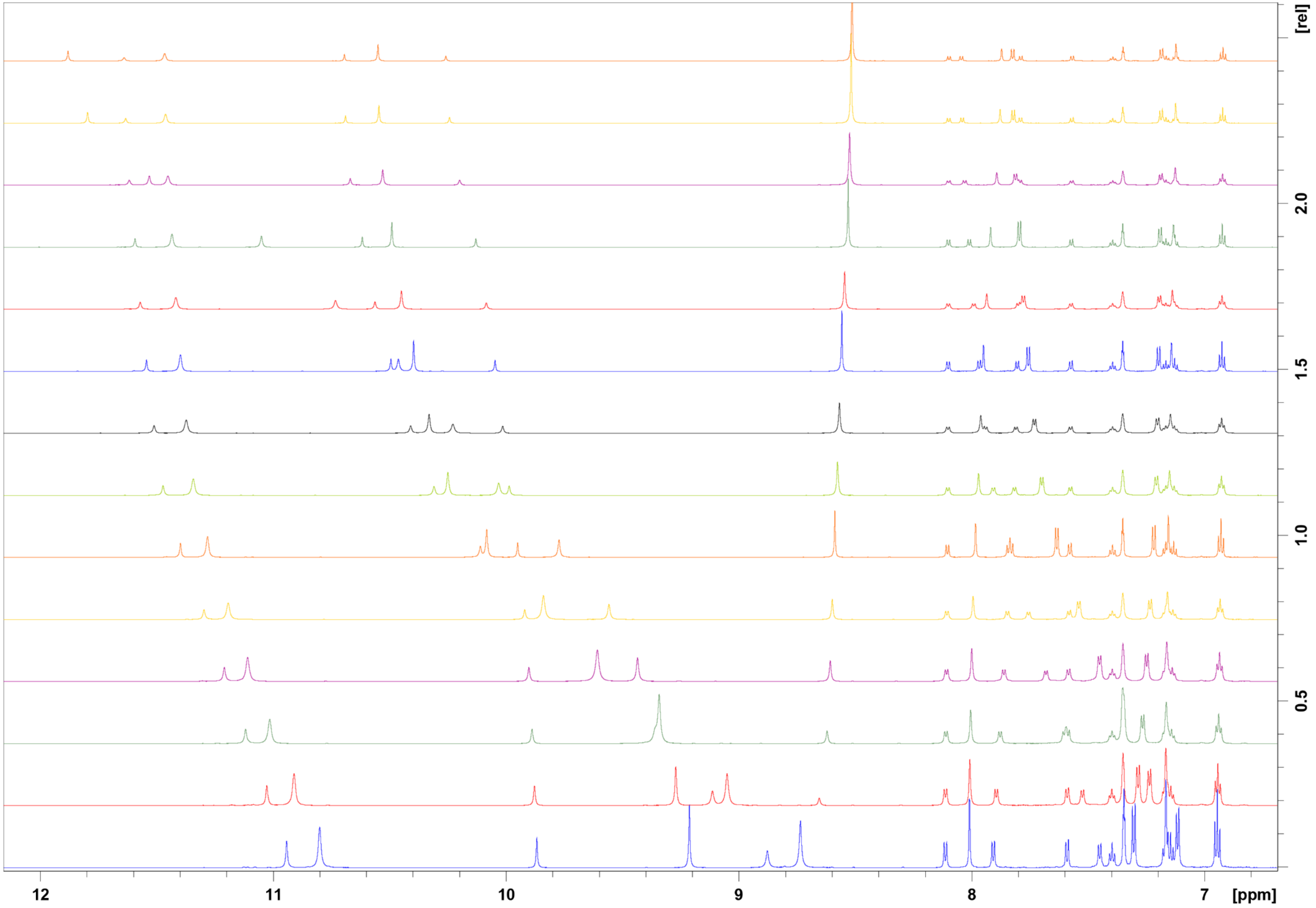

Stacked ^{1}H NMR spectra (700.1 MHz) of a mixture of receptors **MC003**; 1,3-diindolylurea; 1,3-dicarbazolylurea + TBA-formate

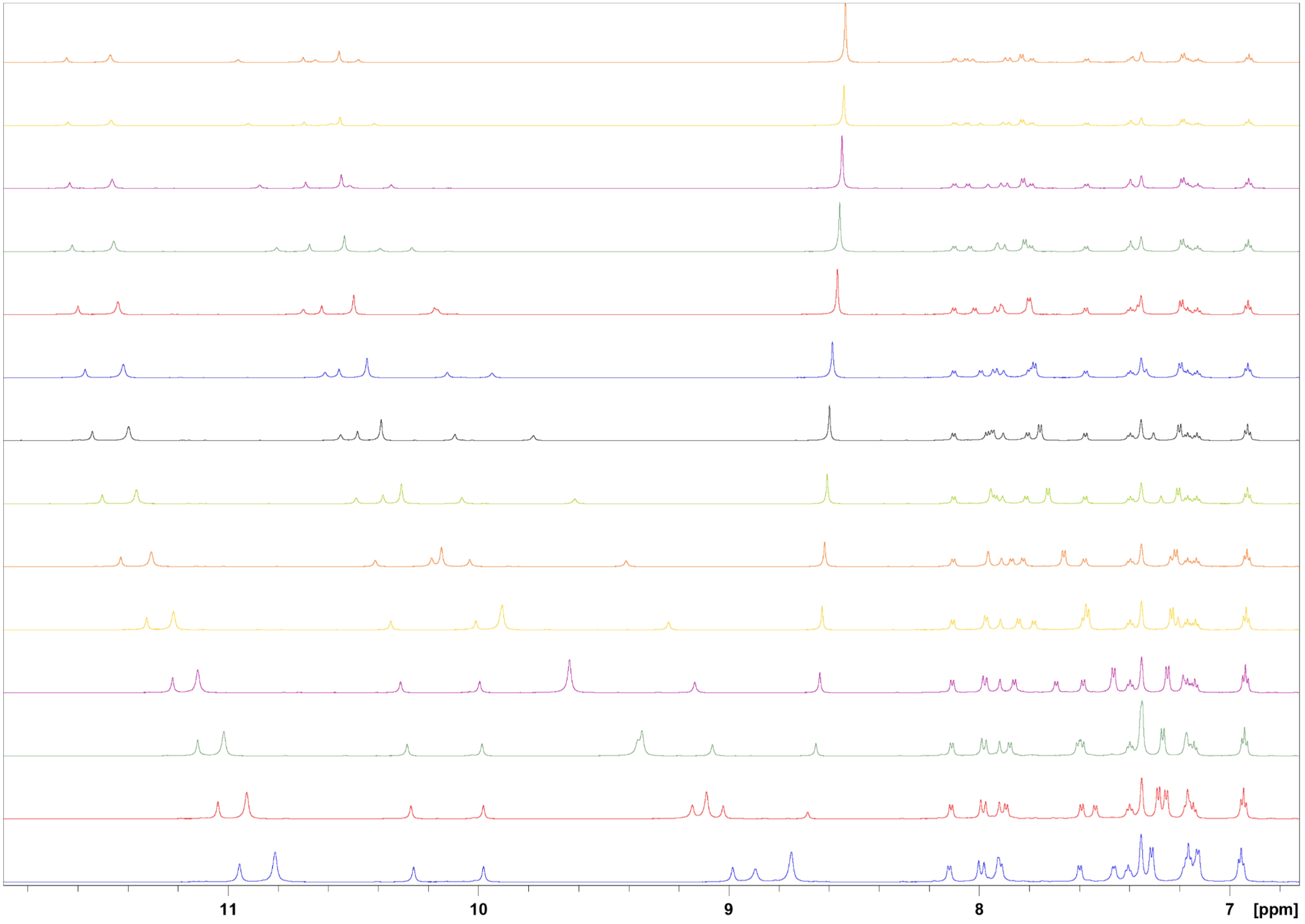

Stacked ^{1}H NMR spectra (700.1 MHz) of a mixture of receptors **MC005**; 1,3-diindolylurea; 1,3-dicarbazolylurea + TBA-formate

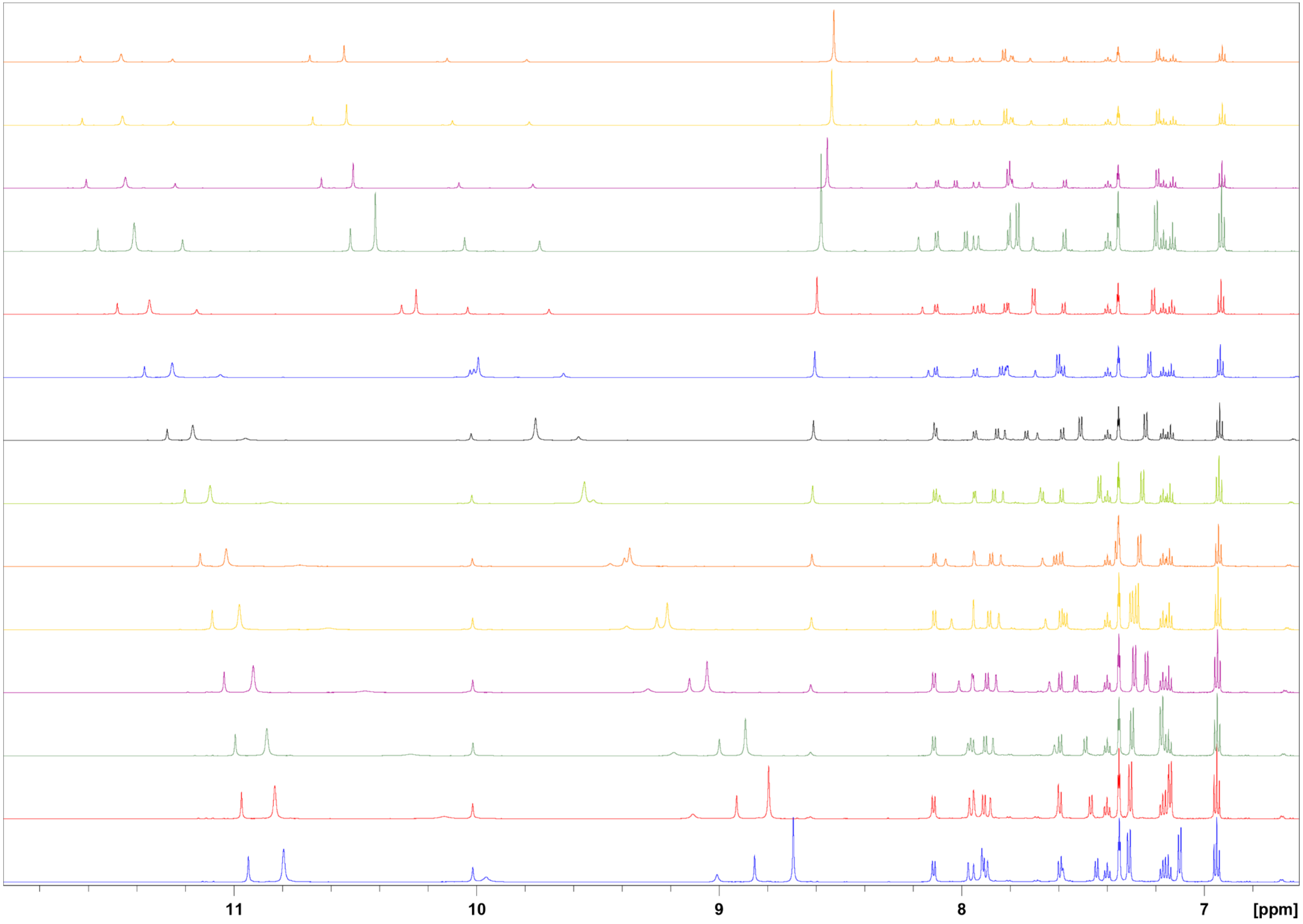

Stacked ^{1}H NMR spectra (700.1 MHz) of a mixture of receptors **MC006**; **MC008**; 1,3-diindolylurea; 1,3-dicarbazolylurea + TBA-formate

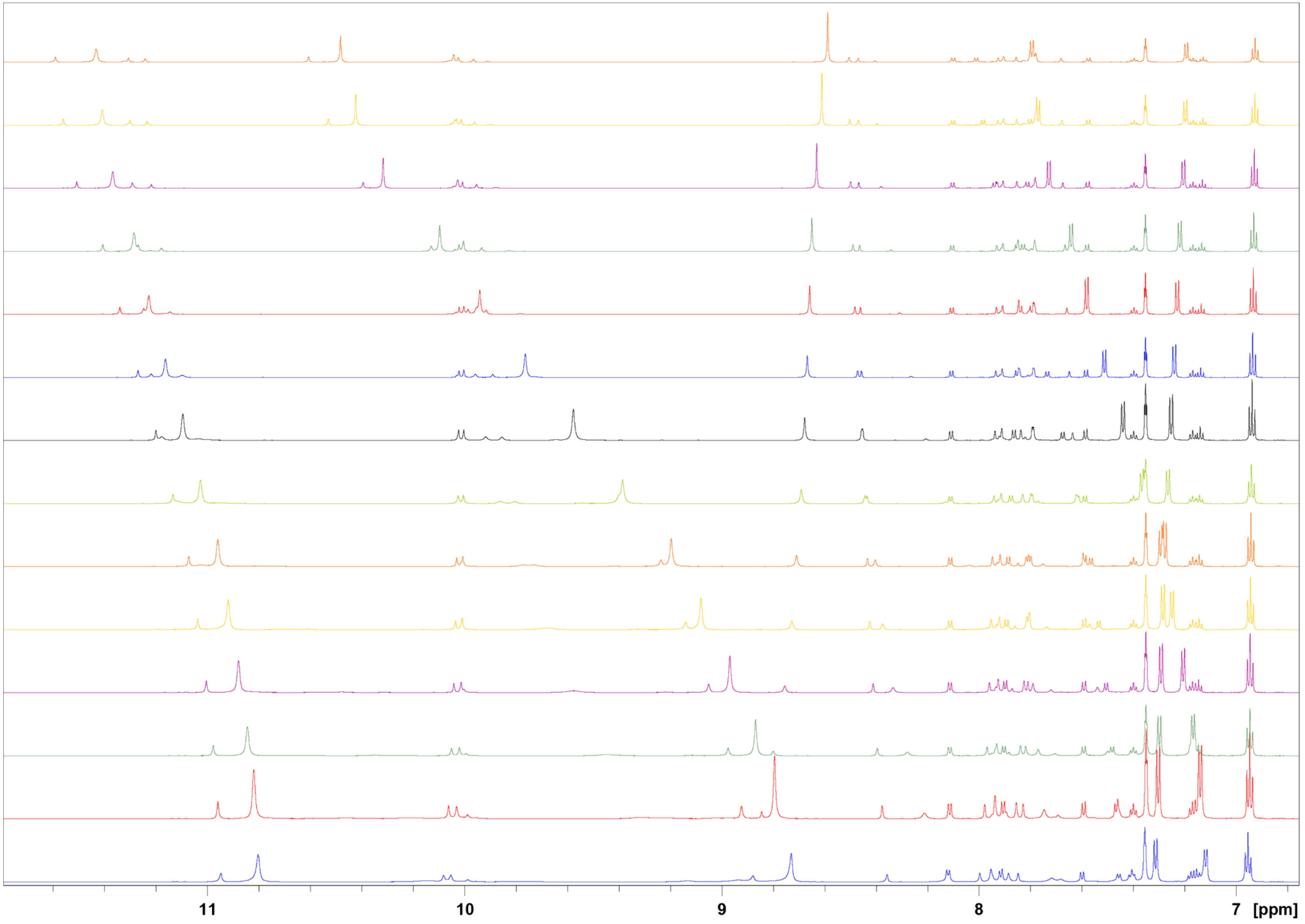

Stacked ^{1}H NMR spectra (700.1 MHz) of a mixture of receptors **MC007**; 1,3-diindolylurea; 1,3-dicarbazolylurea + TBA-formate

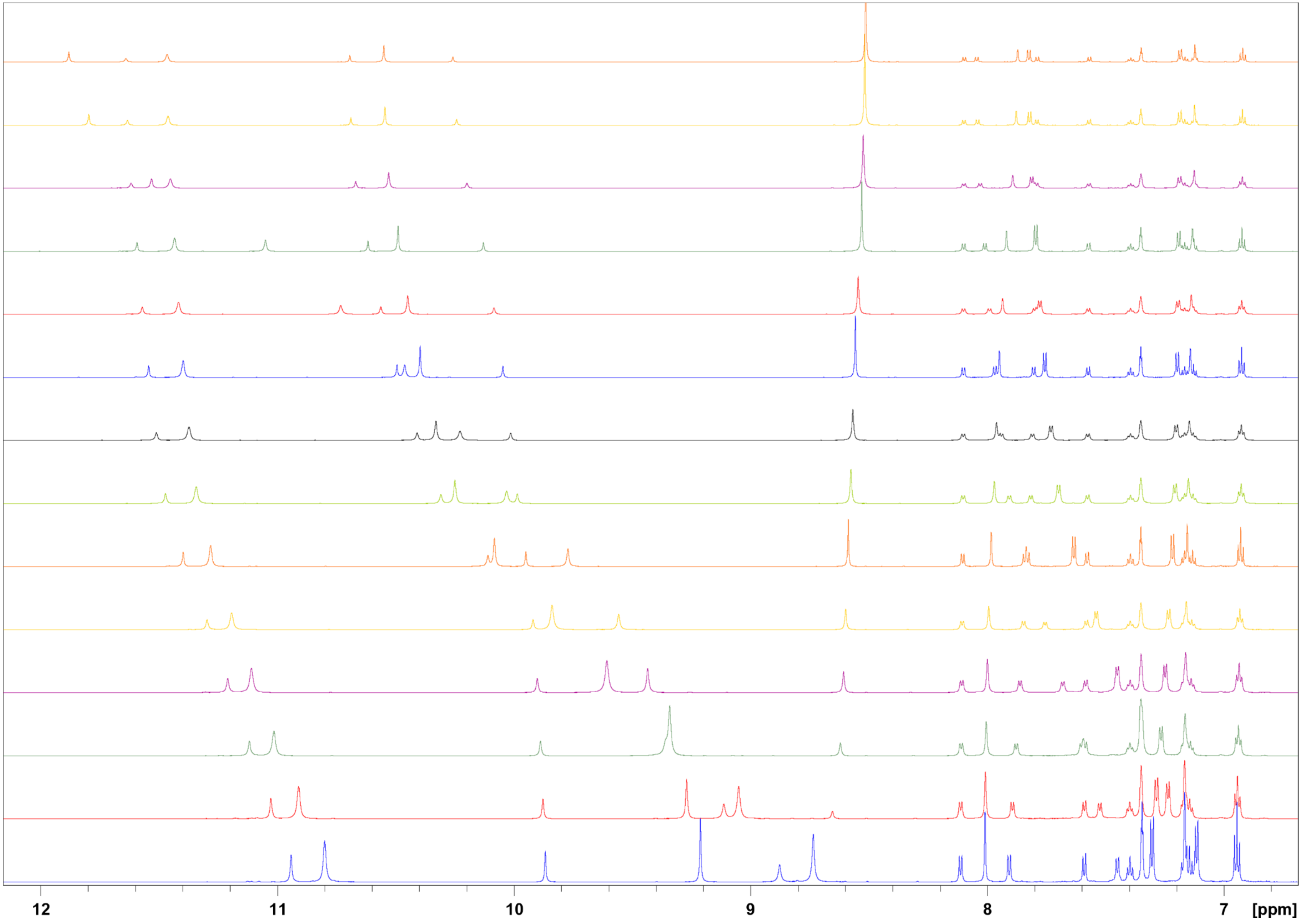

Stacked ^{1}H NMR spectra (700.1 MHz) of a mixture of receptors **MC008**; **MC009**; 1,3-diindolylurea; 1,3-dicarbazolylurea + TBA-formate

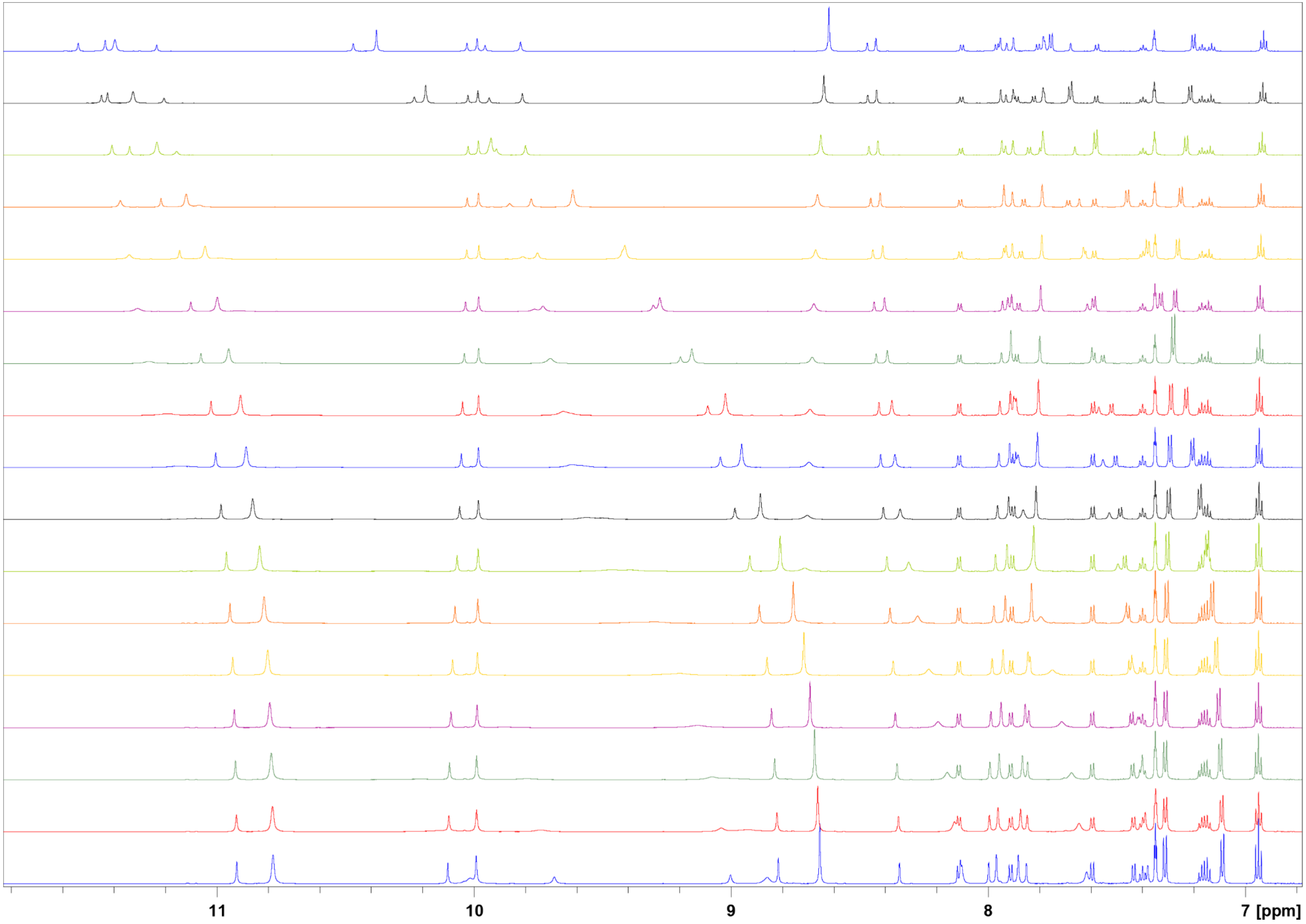

Stacked ^{1}H NMR spectra (700.1 MHz) of a mixture of receptors **MC010**; 1,3-diindolylurea; 1,3-dicarbazolylurea + TBA-formate

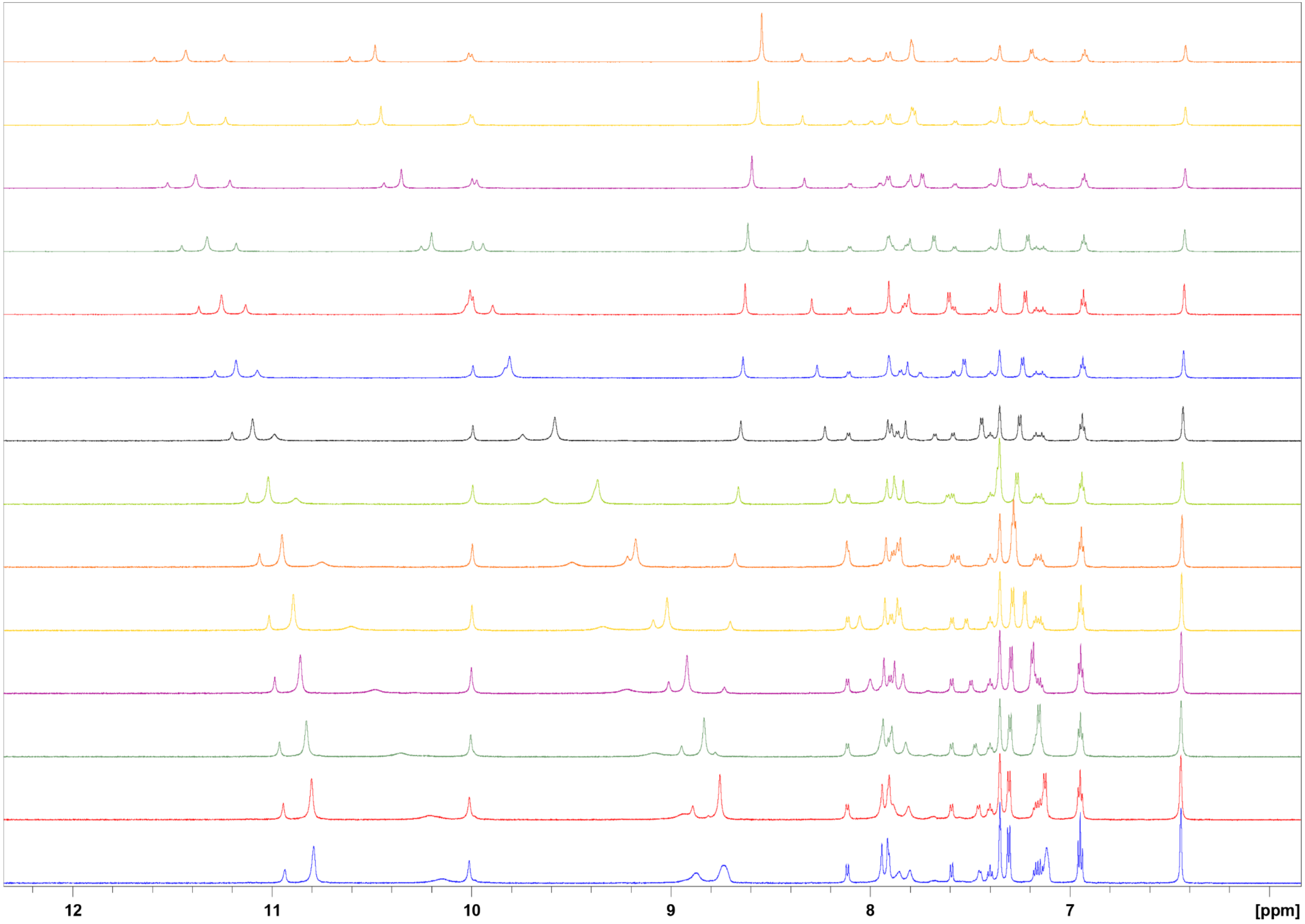

Stacked ^{1}H NMR spectra (700.1 MHz) of a mixture of receptors **MC011**; 1,3-diindolylurea; 1,3-dicarbazolylurea + TBA-formate

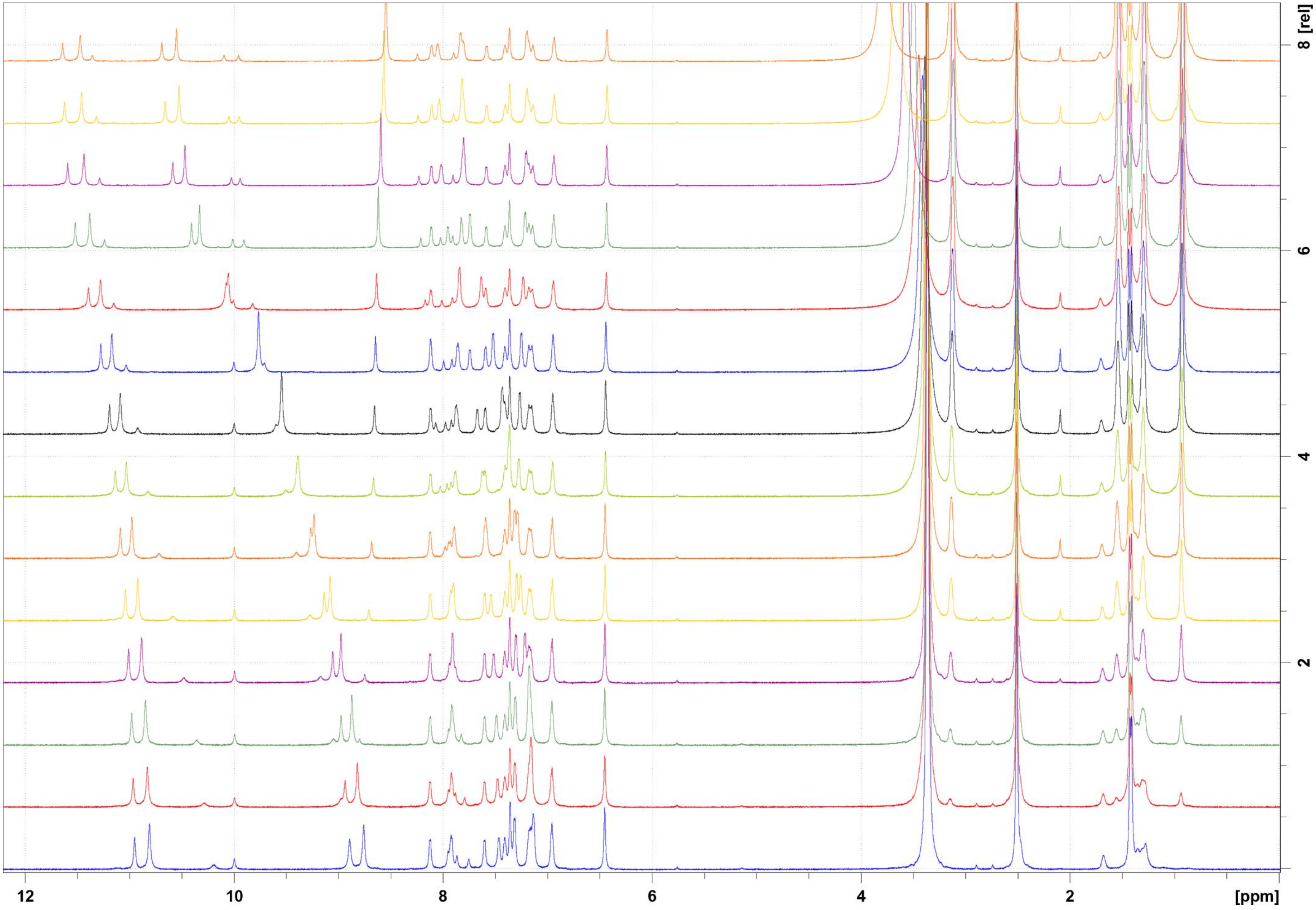

Stacked ^{1}H NMR spectra (700.1 MHz) of a mixture of receptors **MC012**; 1,3-diindolylurea; 1,3-dicarbazolylurea + TBA-formate

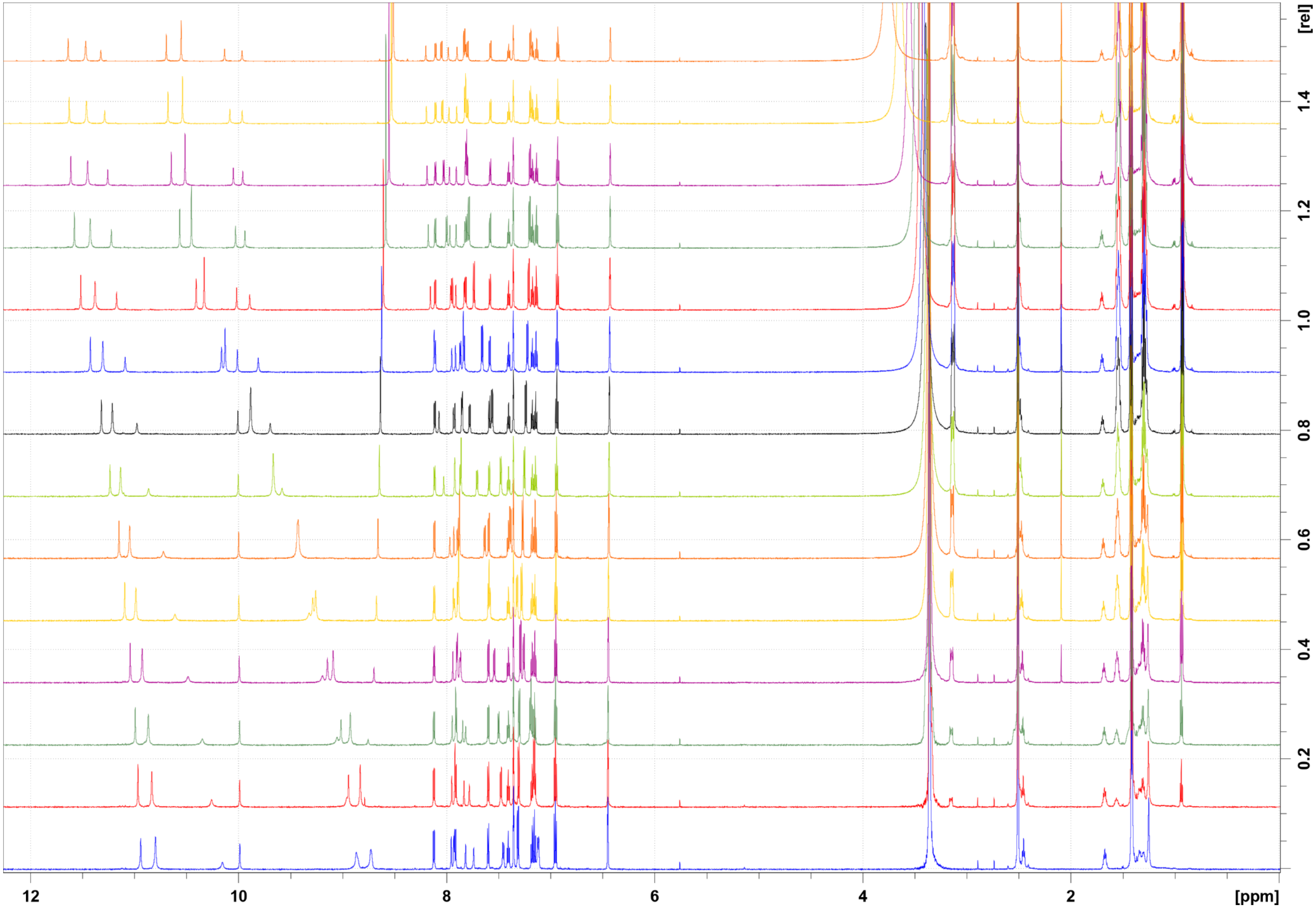

Stacked ^{1}H NMR spectra (700.1 MHz) of a mixture of receptors **MC013**; 1,3-diindolylurea; 1,3-dicarbazolylurea + TBA-formate

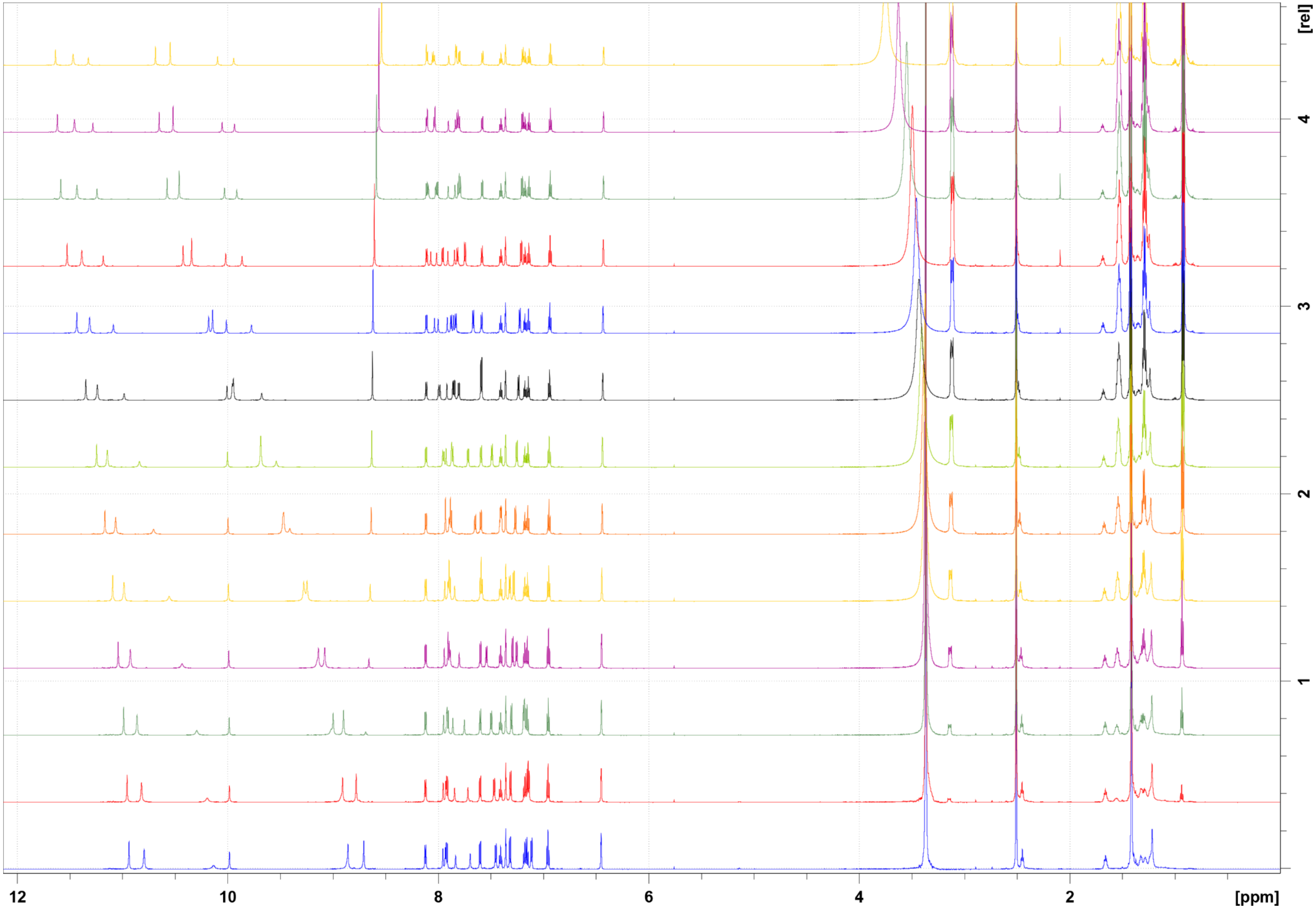

Stacked ^{1}H NMR spectra (700.1 MHz) of a mixture of receptors **MC014**; 1,3-diindolylurea; 1,3-dicarbazolylurea + TBA-formate

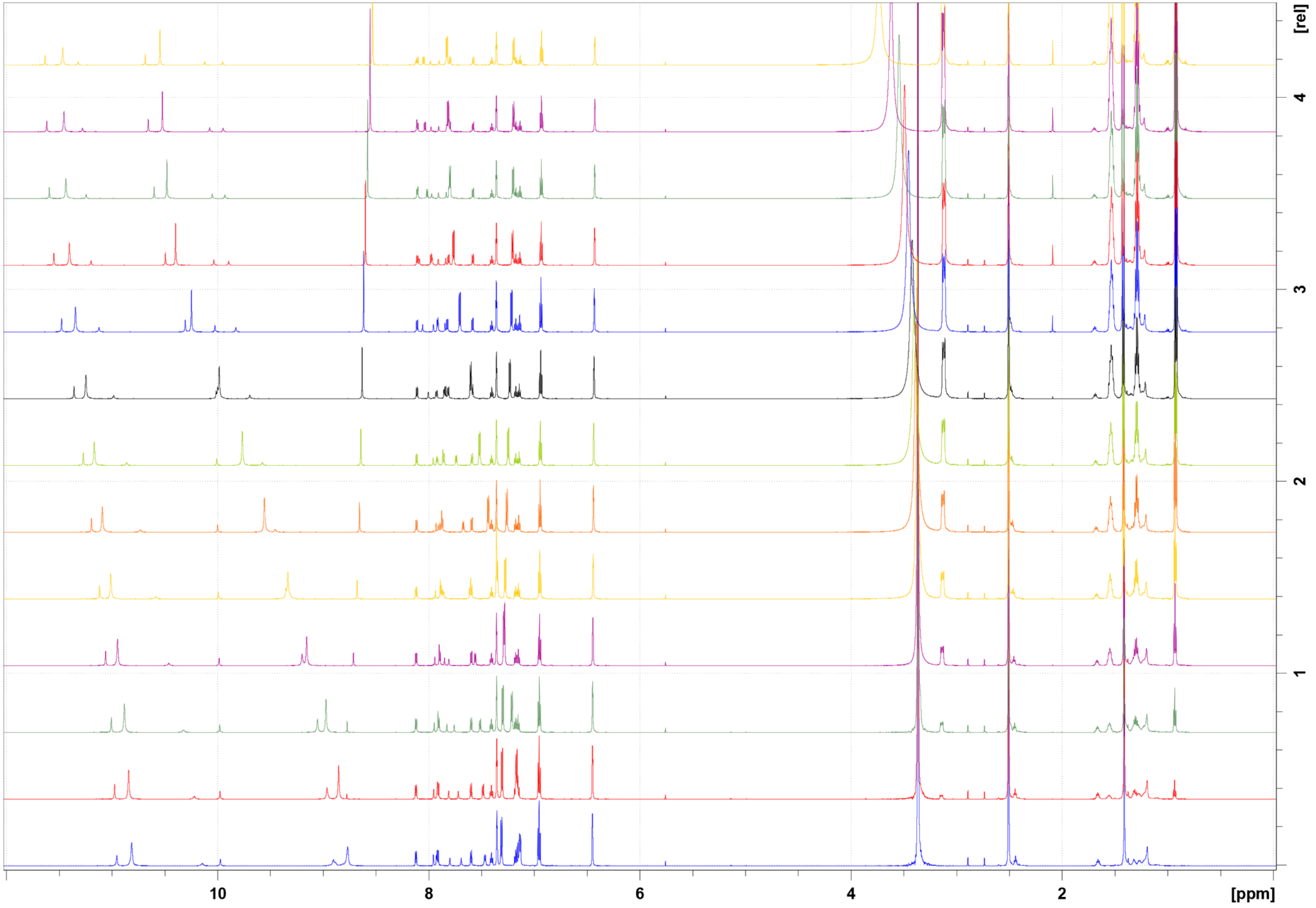

Stacked ^{1}H NMR spectra (700.1 MHz) of a mixture of receptors **CZ016**; 1,3-diindolylurea; 1,3-dicarbazolylurea + TBA-lactate

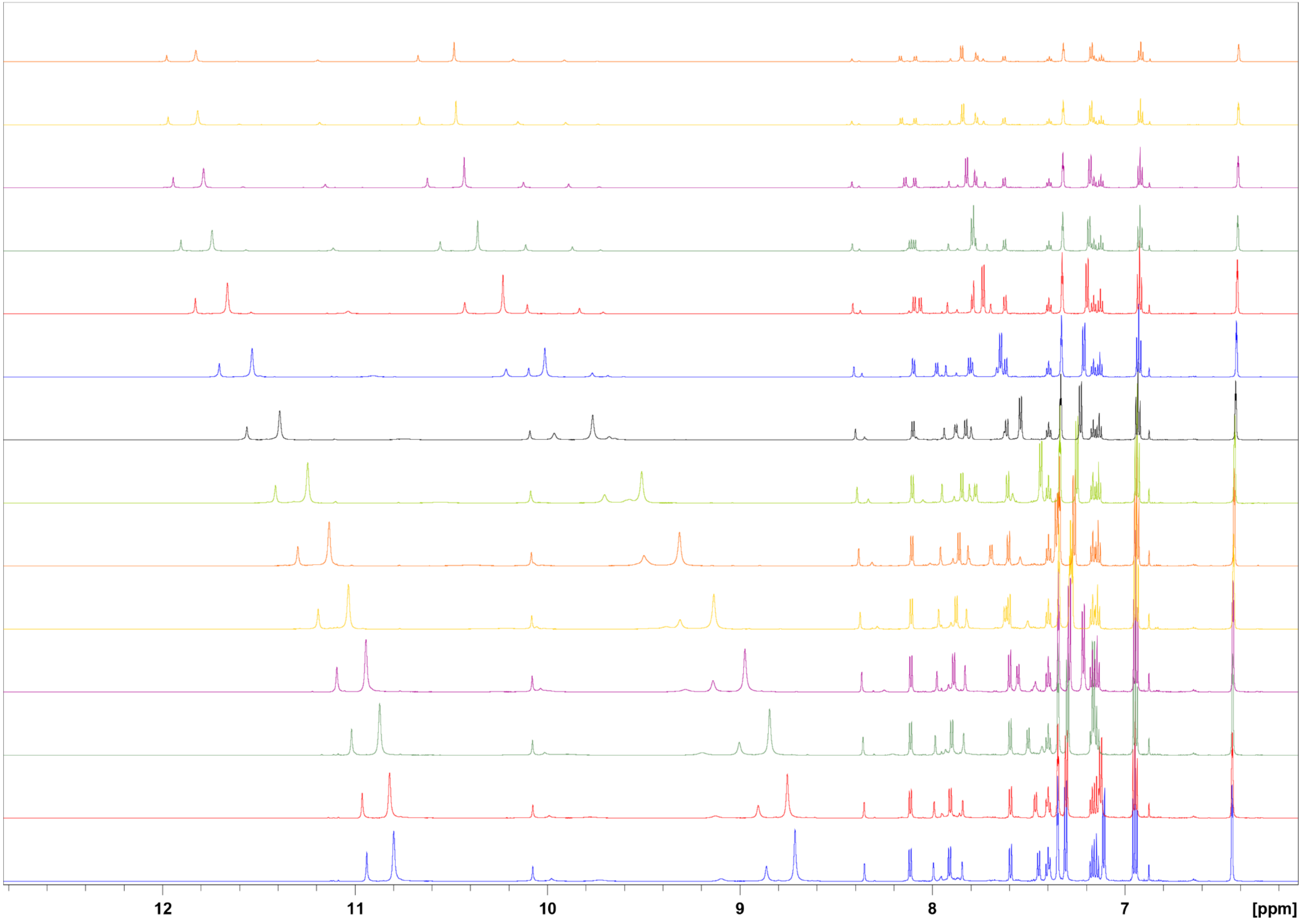

Stacked ^{1}H NMR spectra (700.1 MHz) of a mixture of receptors **MC001**; 2,7-(COOBu)$_2$-indolocarbazole; 2,9-(COOBu)$_2$-indolocarbazole + TBA-lactate

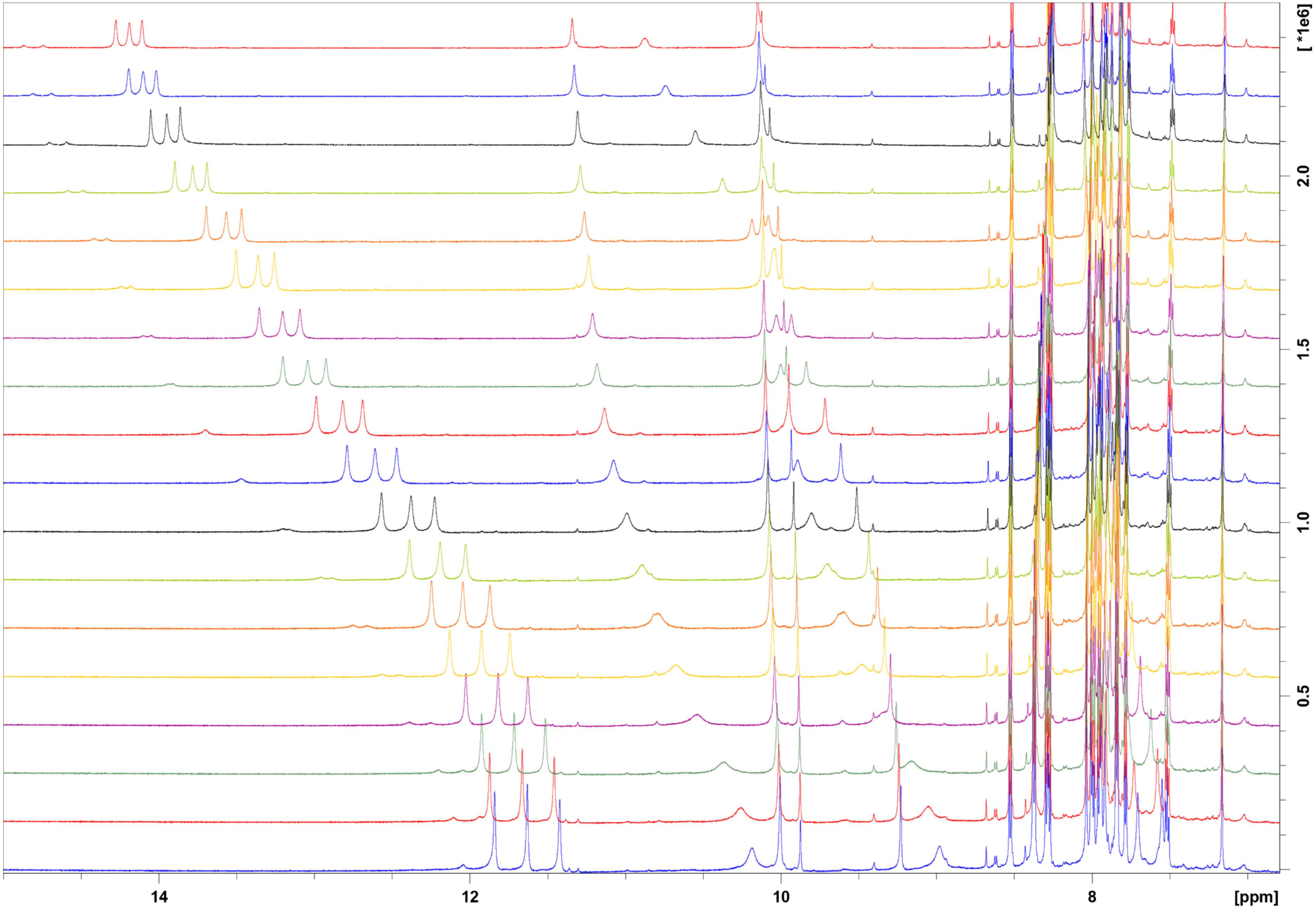

Stacked ^{1}H NMR spectra (700.1 MHz) of a mixture of receptors **MC003**; 1,3-diindolylurea; 1,3-dicarbazolylurea + TBA-lactate

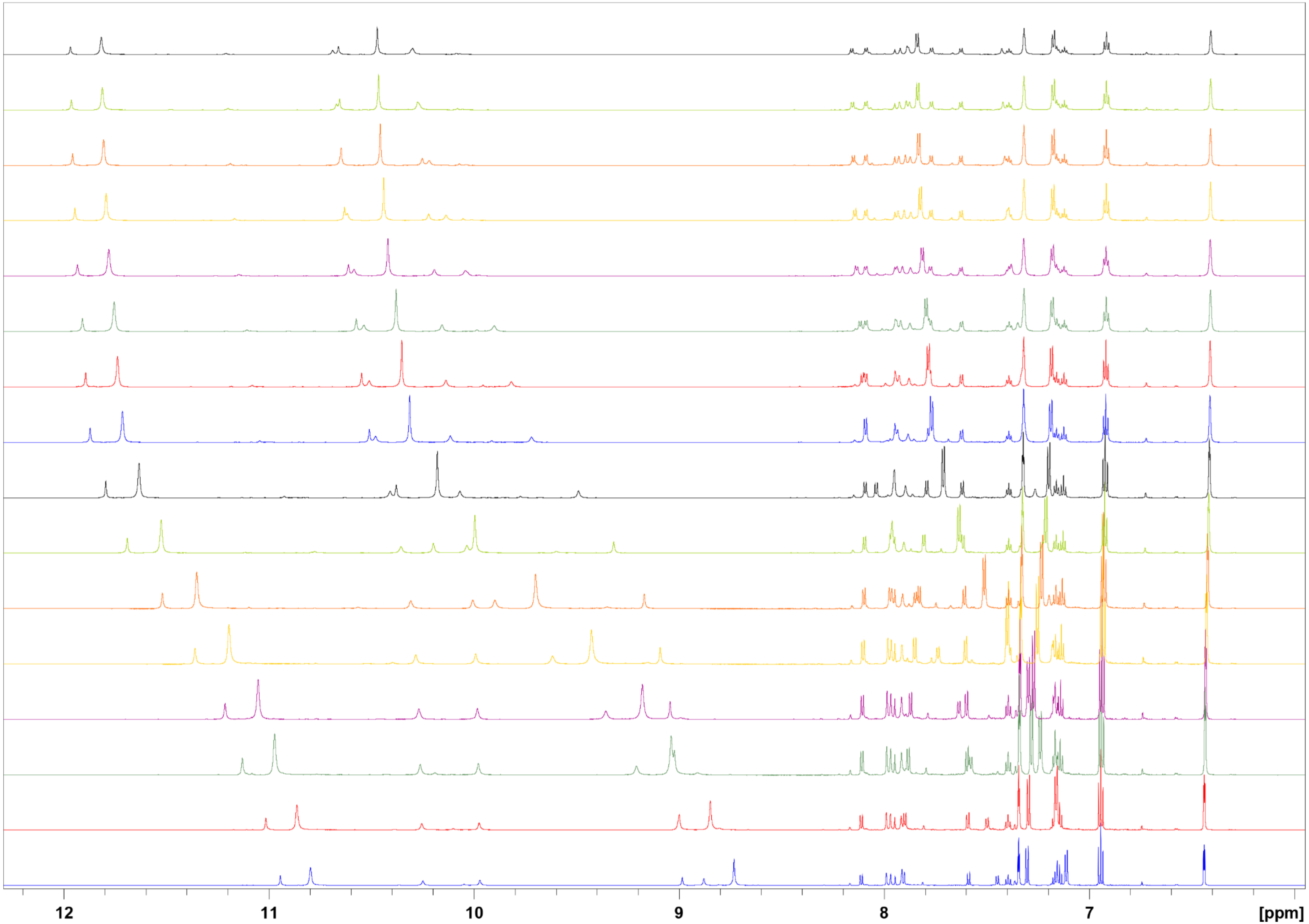

Stacked ^{1}H NMR spectra (700.1 MHz) of a mixture of receptors **MC004**; 1,3-diindolylurea; 1,3-dicarbazolylurea + TBA-lactate

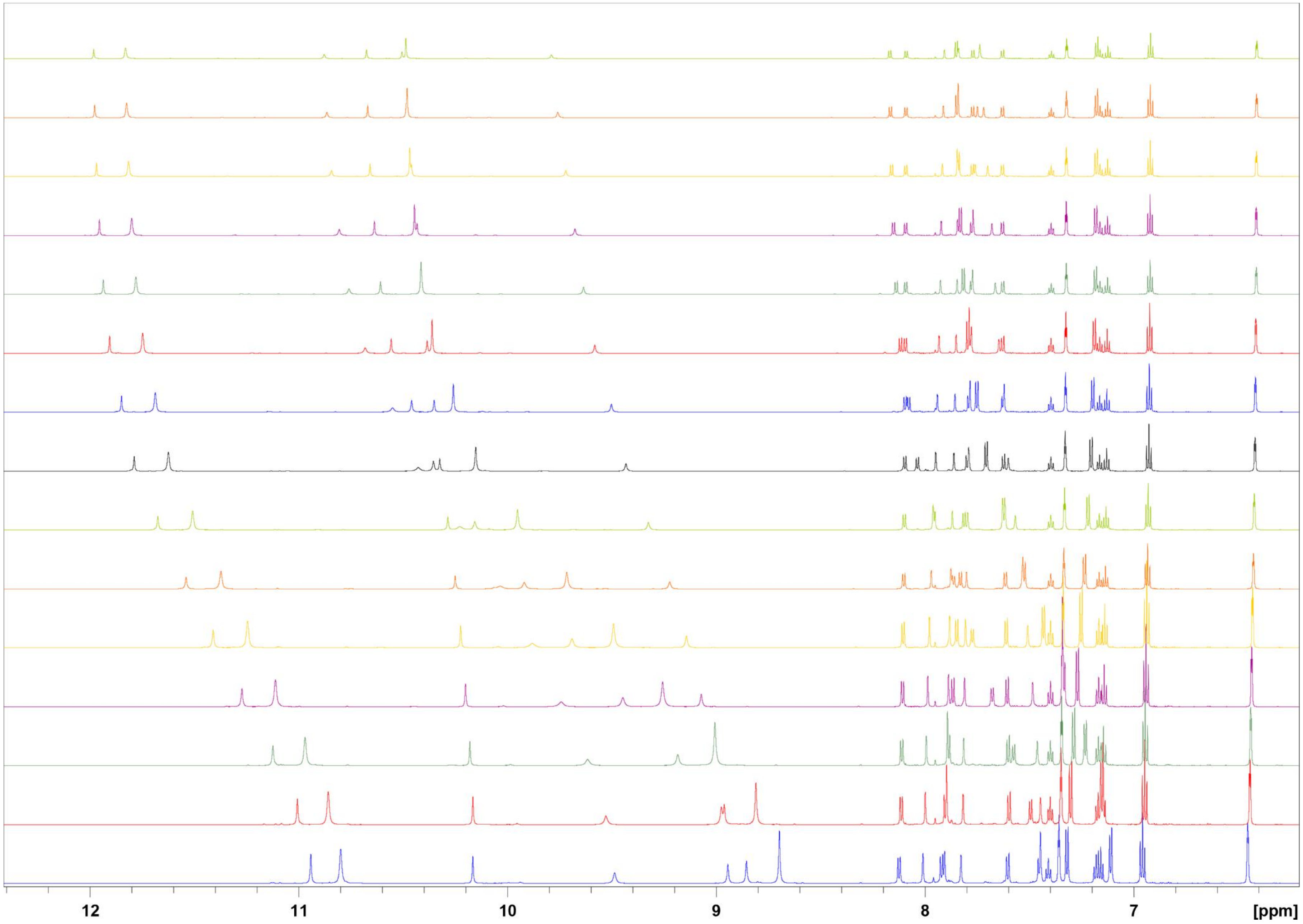

Stacked ^{1}H NMR spectra (700.1 MHz) of a mixture of receptors **MC005**; 1,3-diindolylurea; 1,3-dicarbazolylurea + TBA-lactate

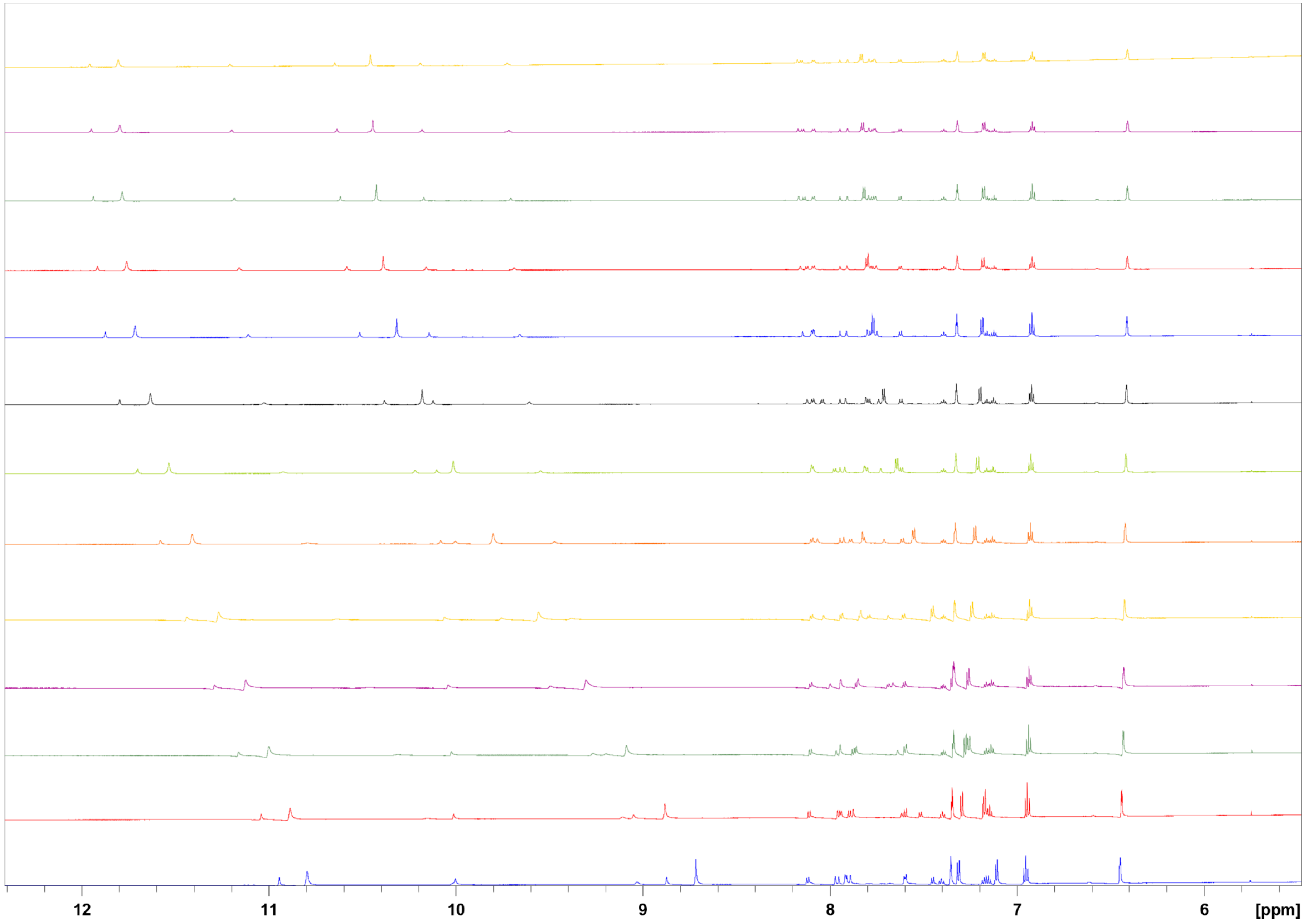

Stacked ^{1}H NMR spectra (700.1 MHz) of a mixture of receptors **MC006**; 1,3-diindolylurea; 1,3-dicarbazolylurea + TBA-lactate

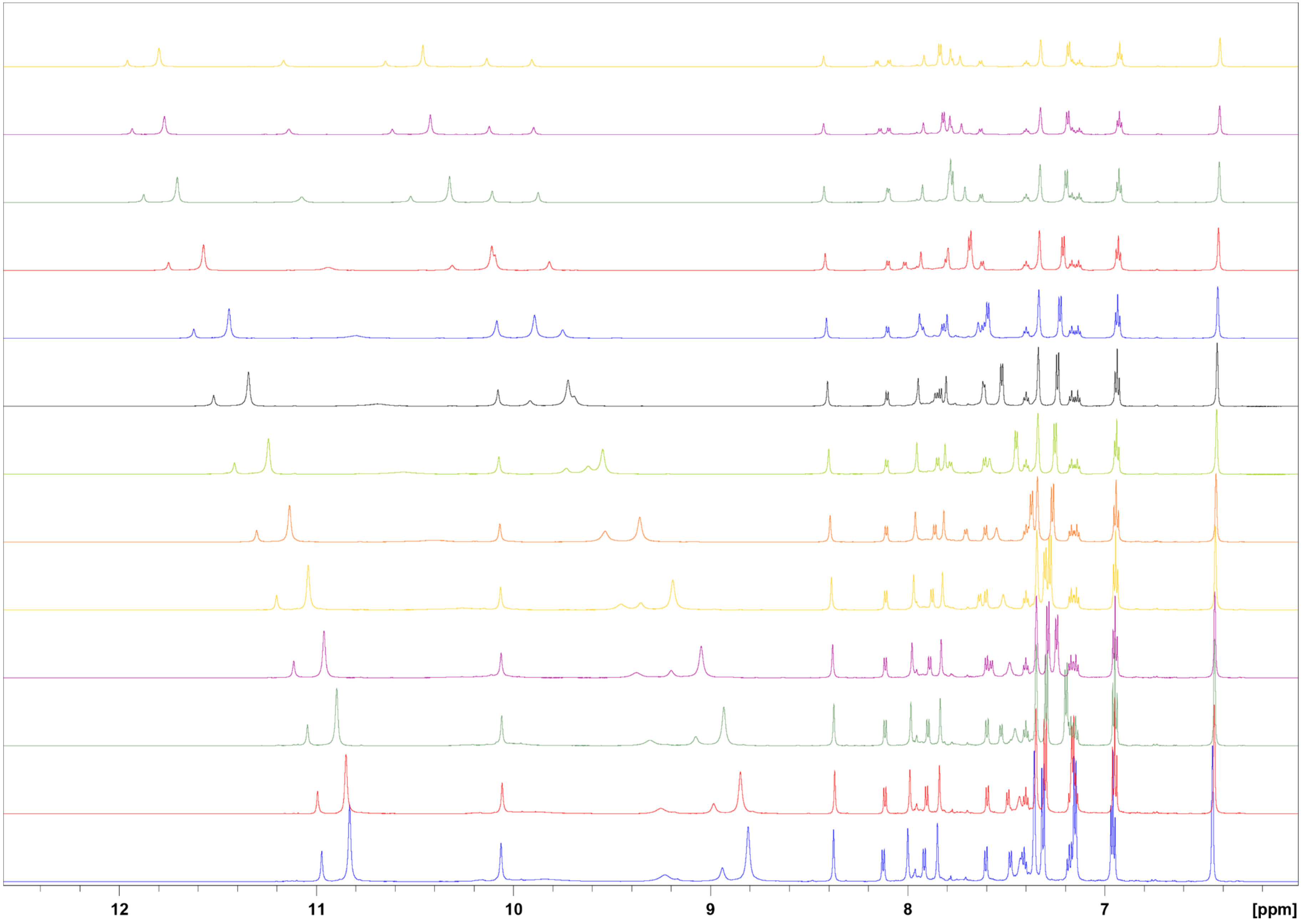

Stacked ^{1}H NMR spectra (700.1 MHz) of a mixture of receptors **MC007**; 1,3-diindolylurea; 1,3-dicarbazolylurea + TBA-lactate

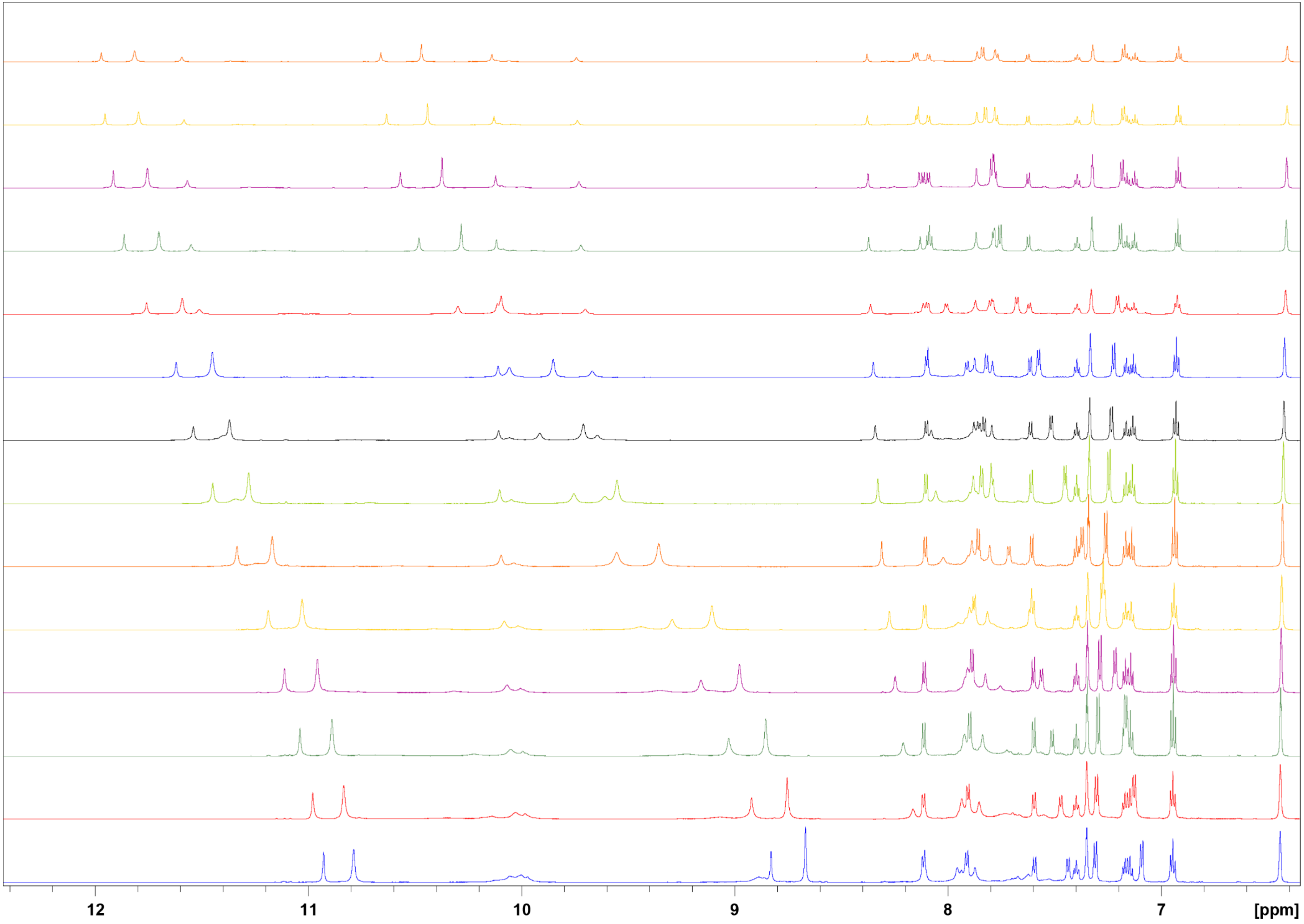

Stacked ^{1}H NMR spectra (700.1 MHz) of a mixture of receptors **MC008**; 1,3-diindolylurea; 1,3-dicarbazolylurea + TBA-lactate

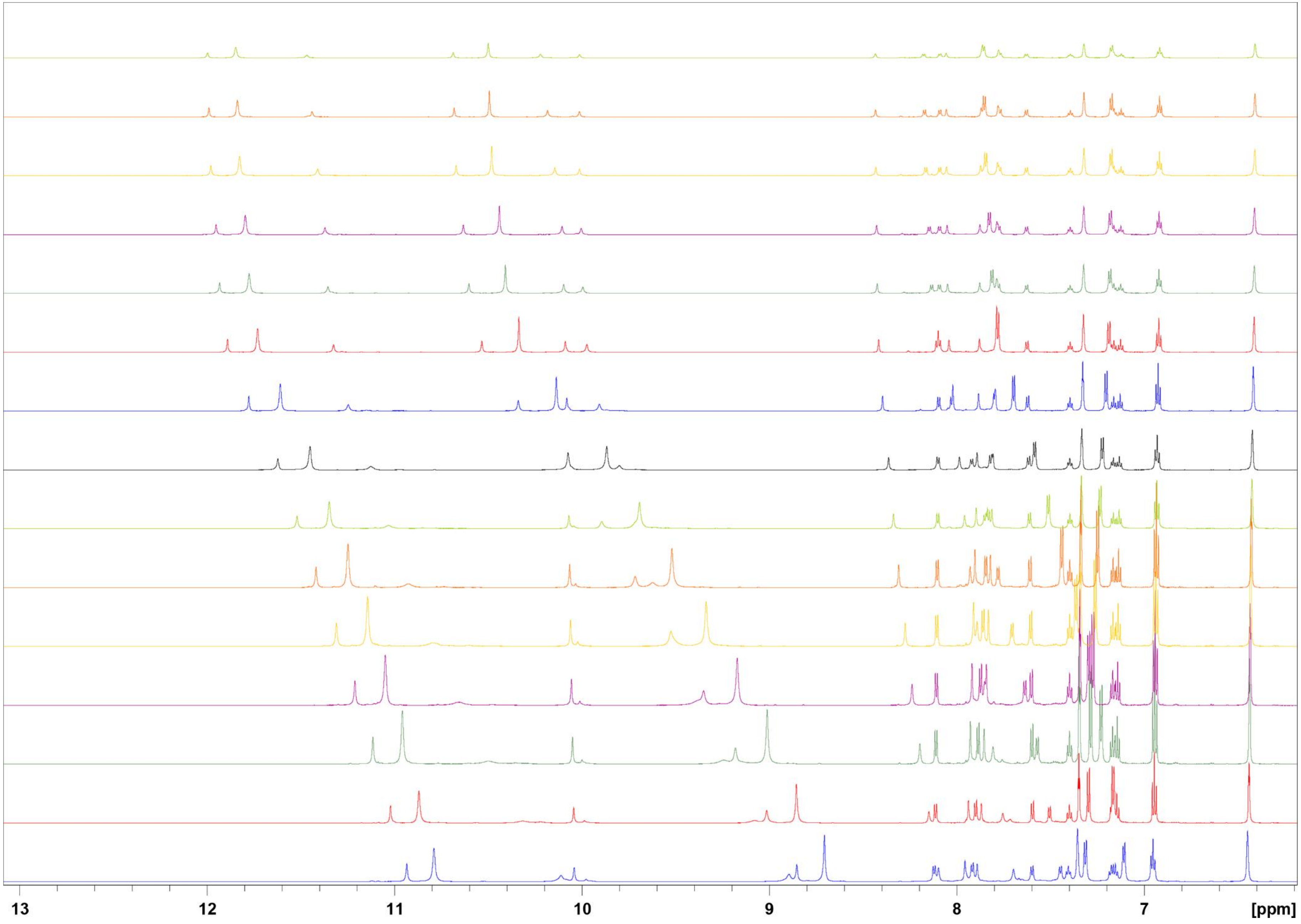

Stacked ^{1}H NMR spectra (700.1 MHz) of a mixture of receptors **MC009**; 1,3-diindolylurea; 1,3-dicarbazolylurea + TBA-lactate

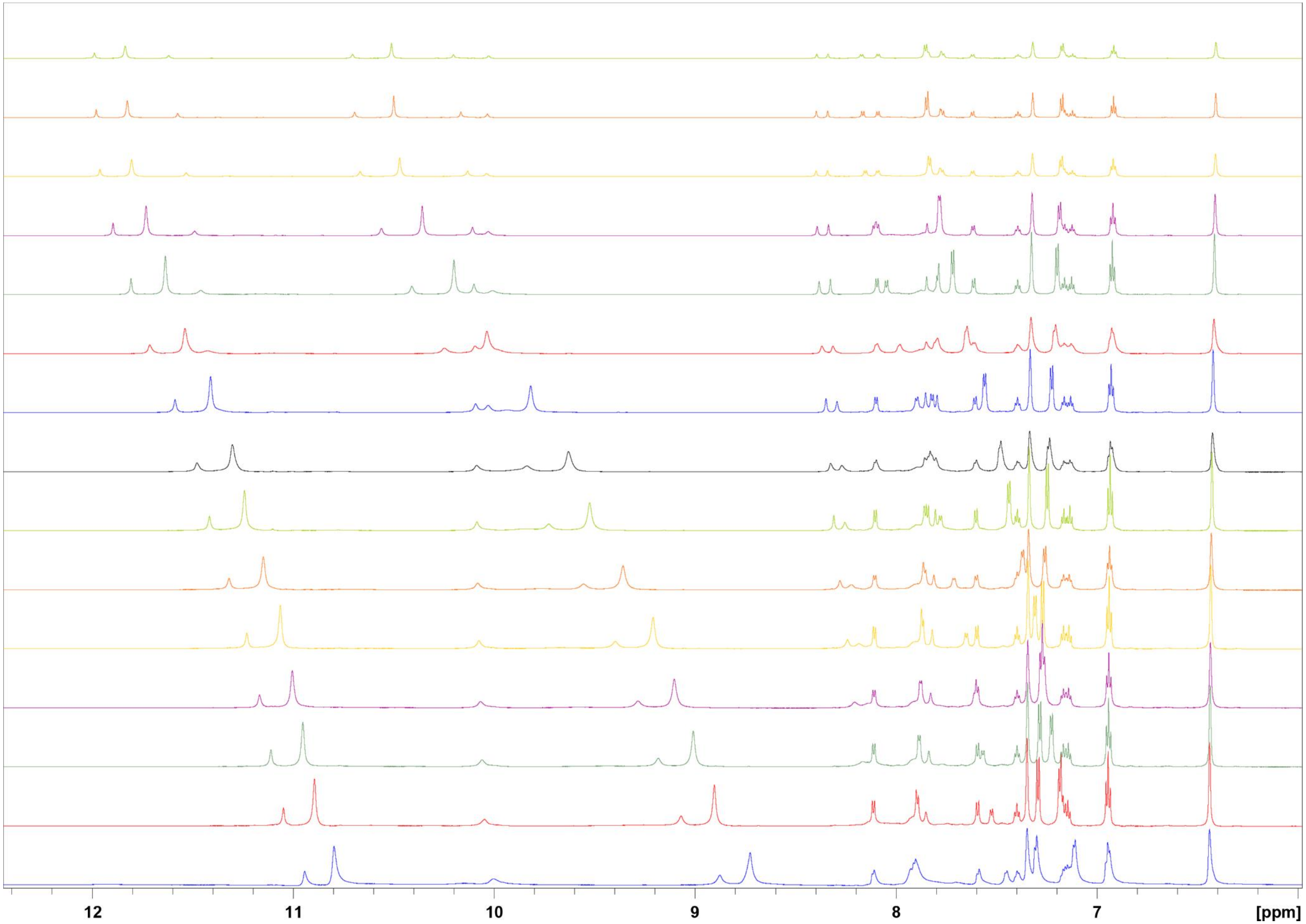

Stacked ^{1}H NMR spectra (700.1 MHz) of a mixture of receptors **MC010**; 1,3-diindolylurea; 1,3-dicarbazolylurea + TBA-lactate

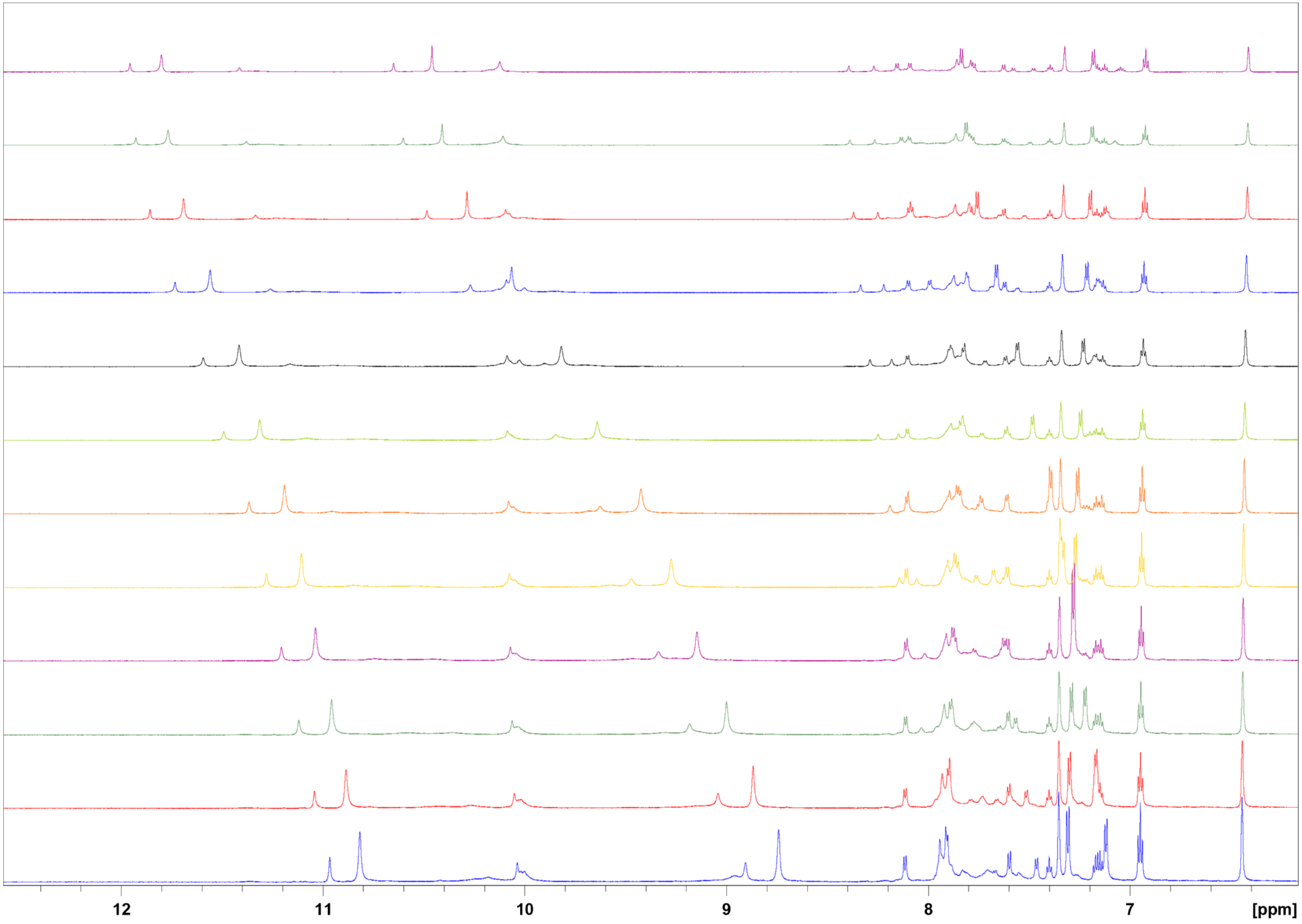

Stacked ^{1}H NMR spectra (700.1 MHz) of a mixture of receptors **MC011**; 1,3-diindolylurea; 1,3-dicarbazolylurea + TBA-lactate

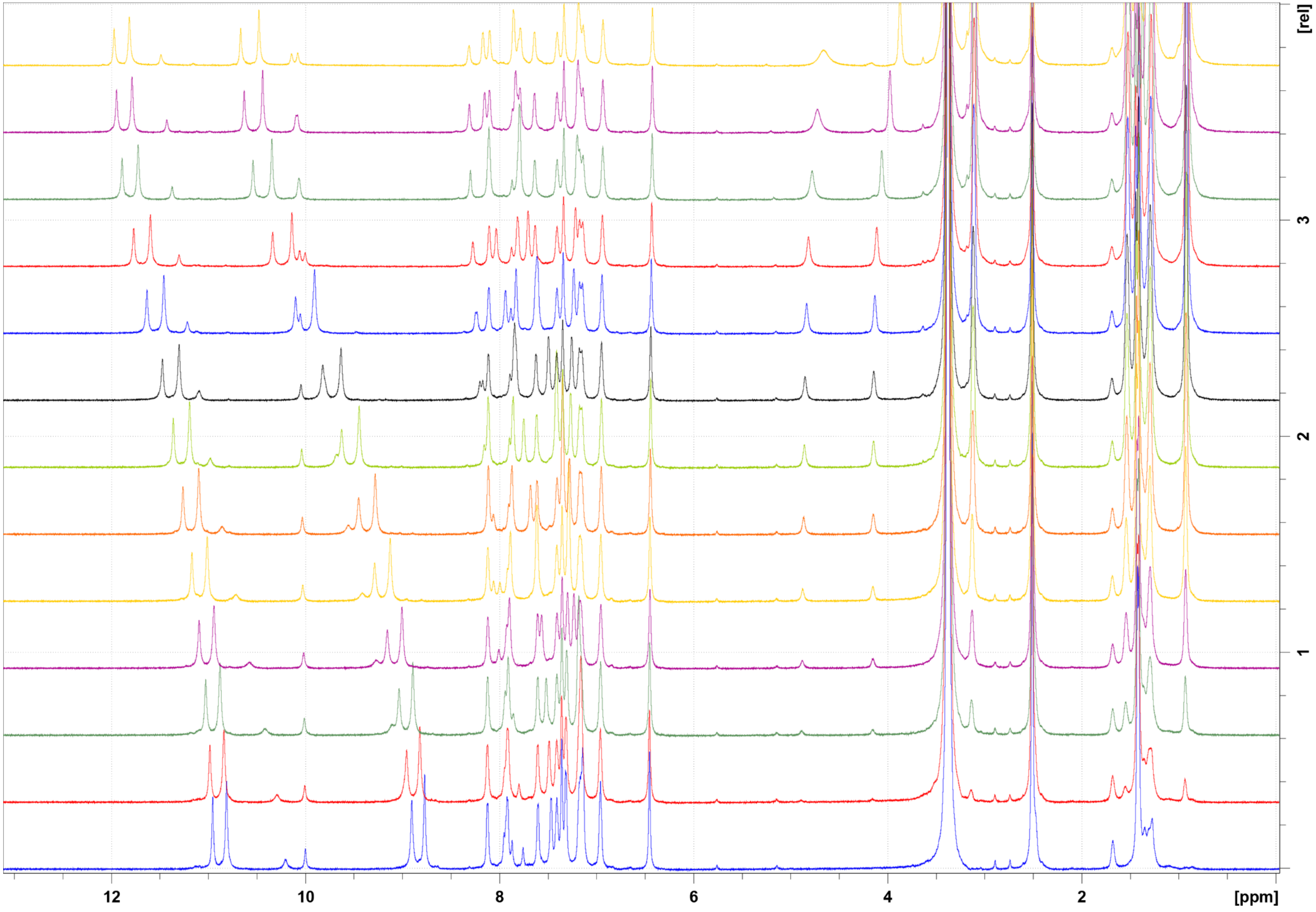

Stacked ^{1}H NMR spectra (700.1 MHz) of a mixture of receptors **MC012**; 1,3-diindolylurea; 1,3-dicarbazolylurea + TBA-lactate

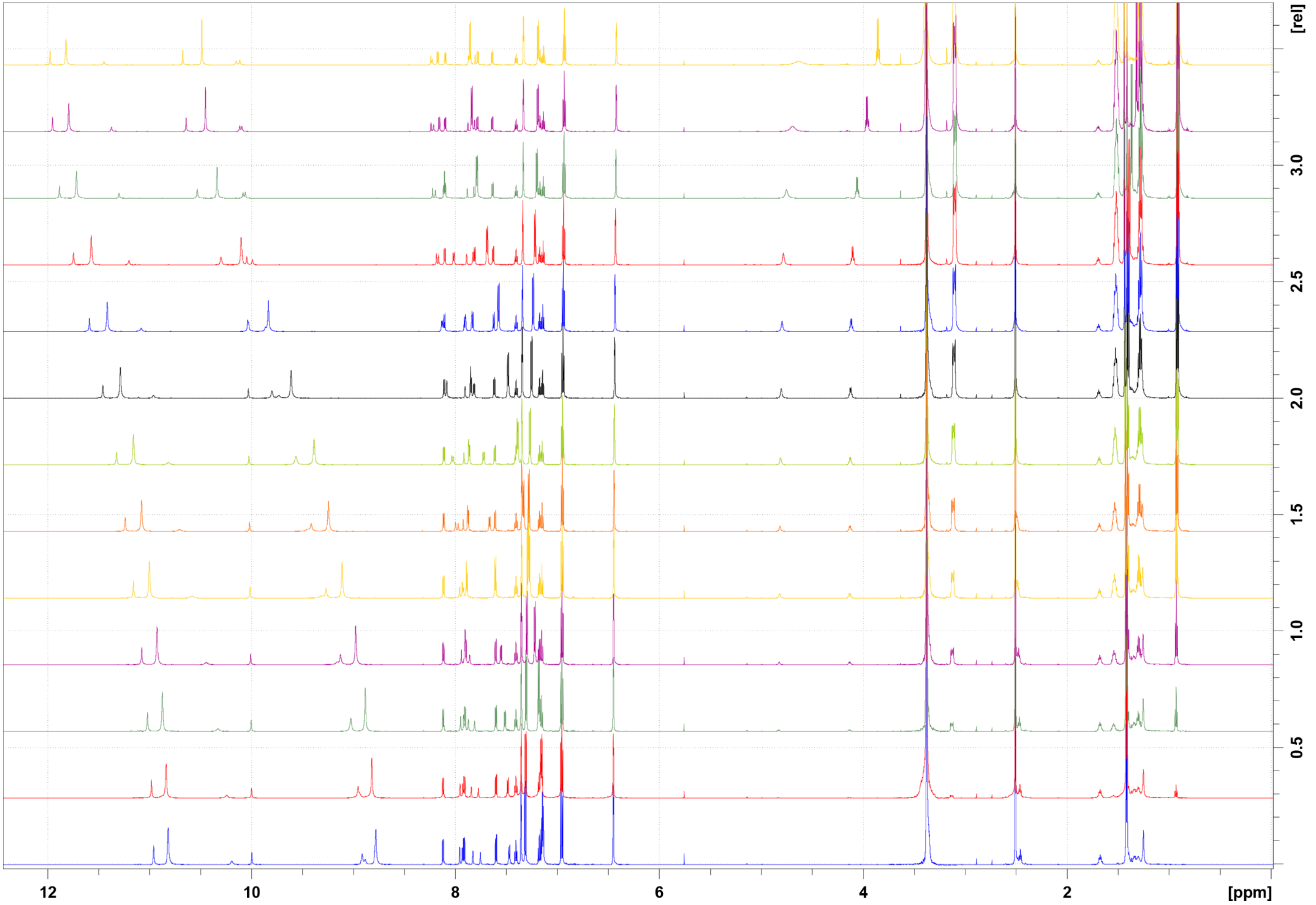

Stacked ^{1}H NMR spectra (700.1 MHz) of a mixture of receptors **MC013**; 1,3-diindolylurea; 1,3-dicarbazolylurea + TBA-lactate

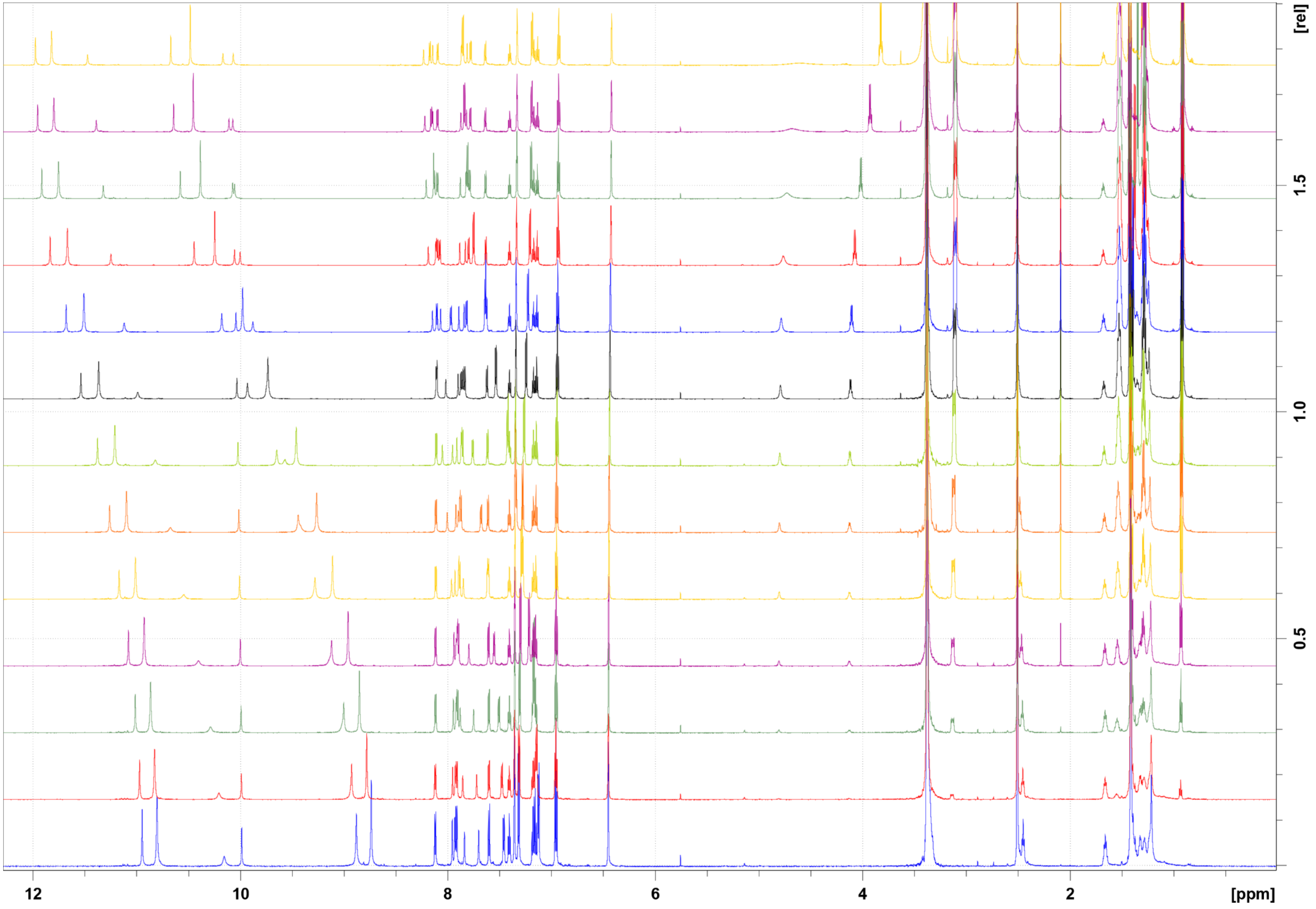

Stacked ^{1}H NMR spectra (700.1 MHz) of a mixture of receptors **MC014**; 1,3-diindolylurea; 1,3-dicarbazolylurea + TBA-lactate

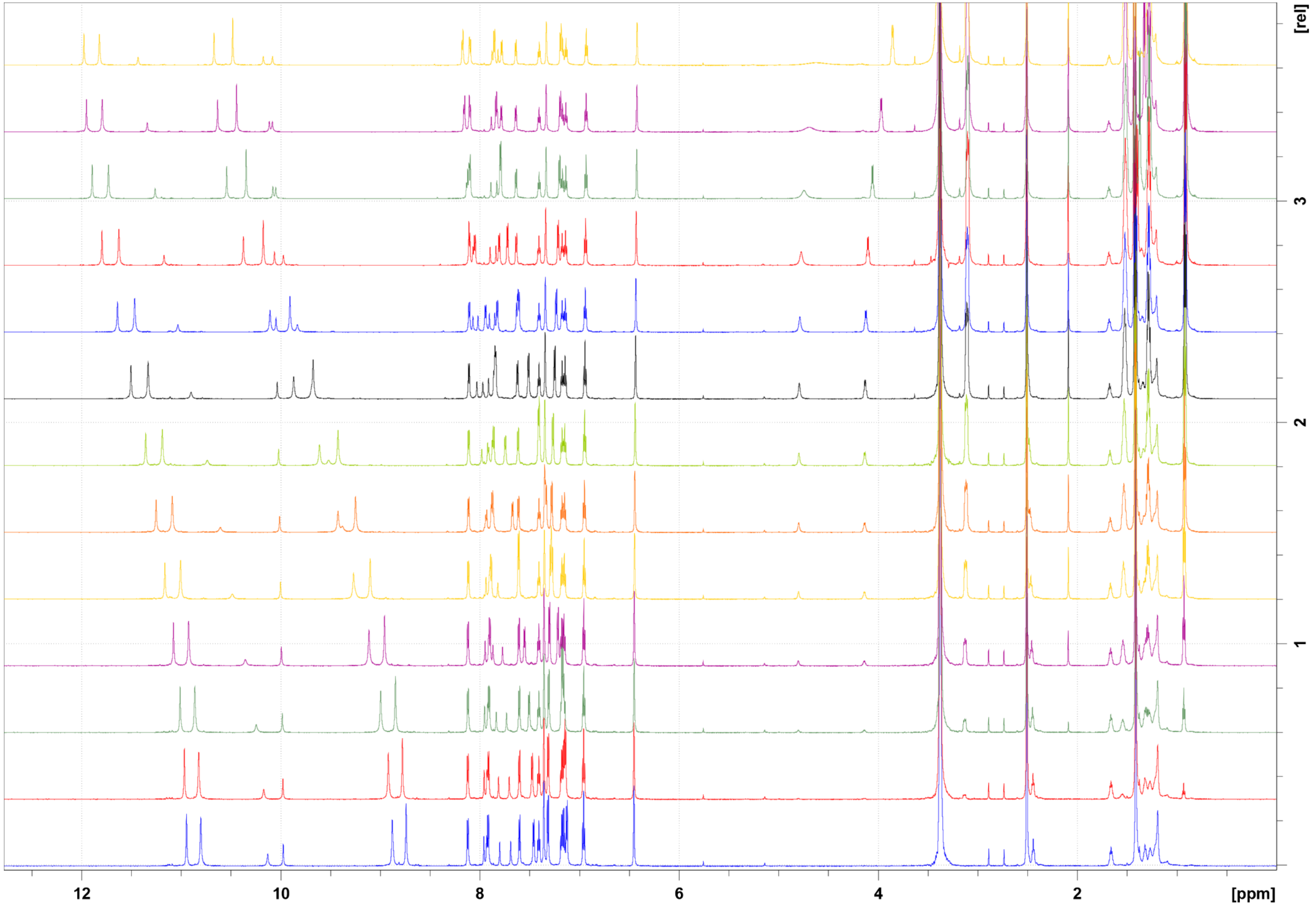

Stacked ^1H NMR spectra (700.1 MHz) of a mixture of receptors **CZ016**; 1,3-diindolylurea; 1,3-dicarbazolylurea + TBA-pivalate

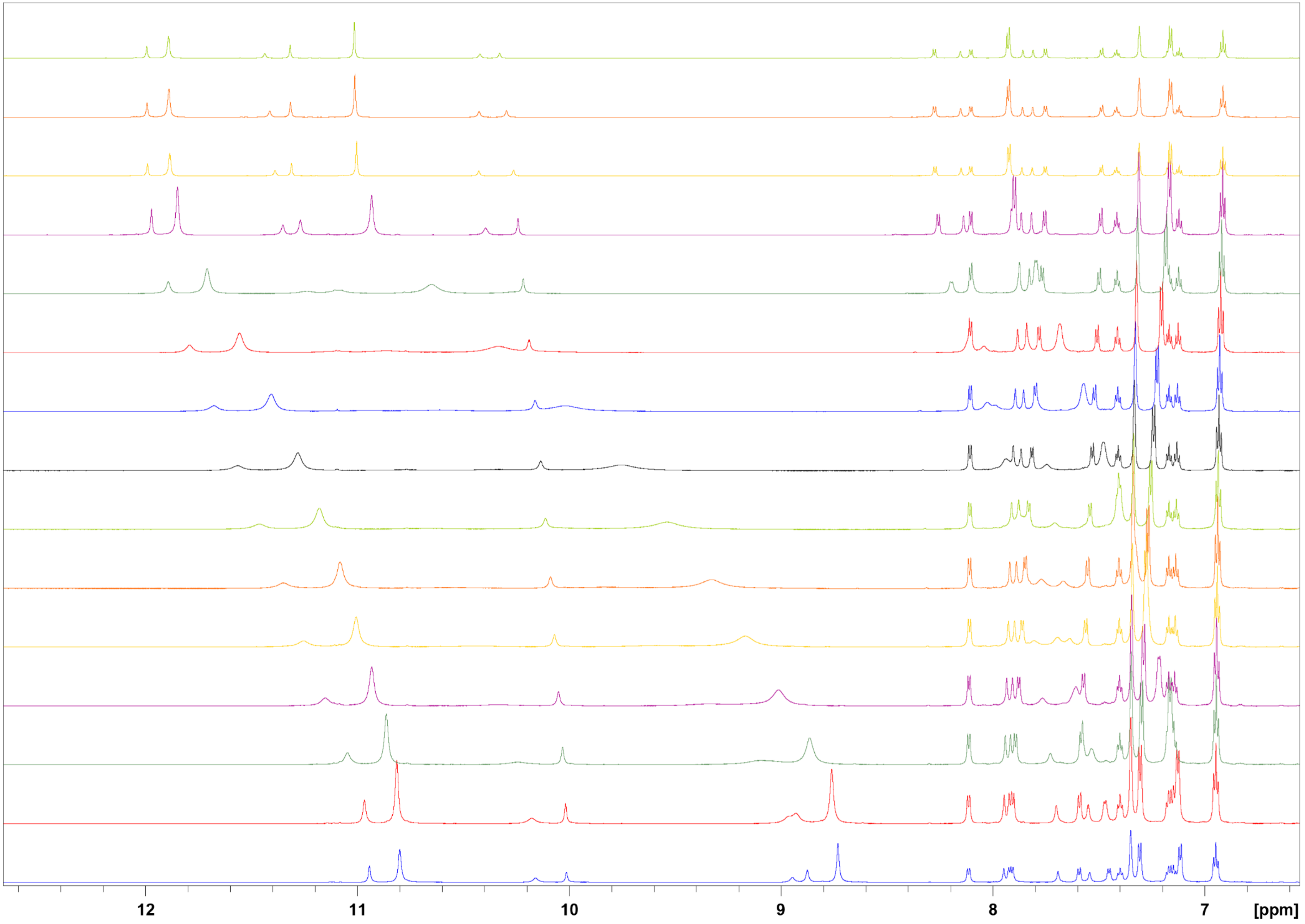

Stacked ^{1}H NMR spectra (700.1 MHz) of a mixture of receptors **MC001**; 2,7-$(COOBu)_2$-indolocarbazole; 2,9-$(COOBu)_2$-indolocarbazole + TBA-pivalate

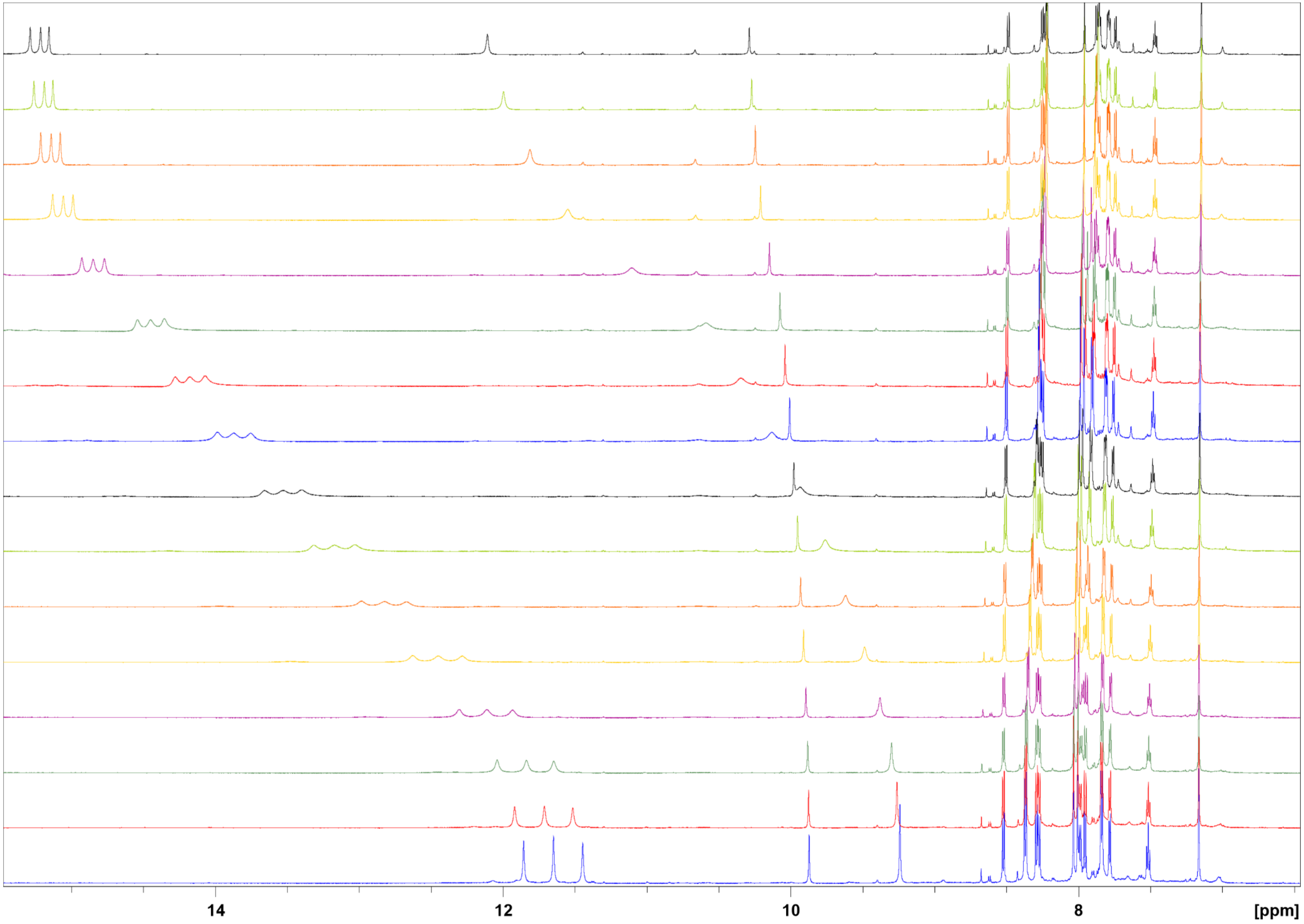

Stacked ^{1}H NMR spectra (700.1 MHz) of a mixture of receptors **MC003**; 1,3-diindolylurea; 1,3-dicarbazolylurea + TBA-pivalate

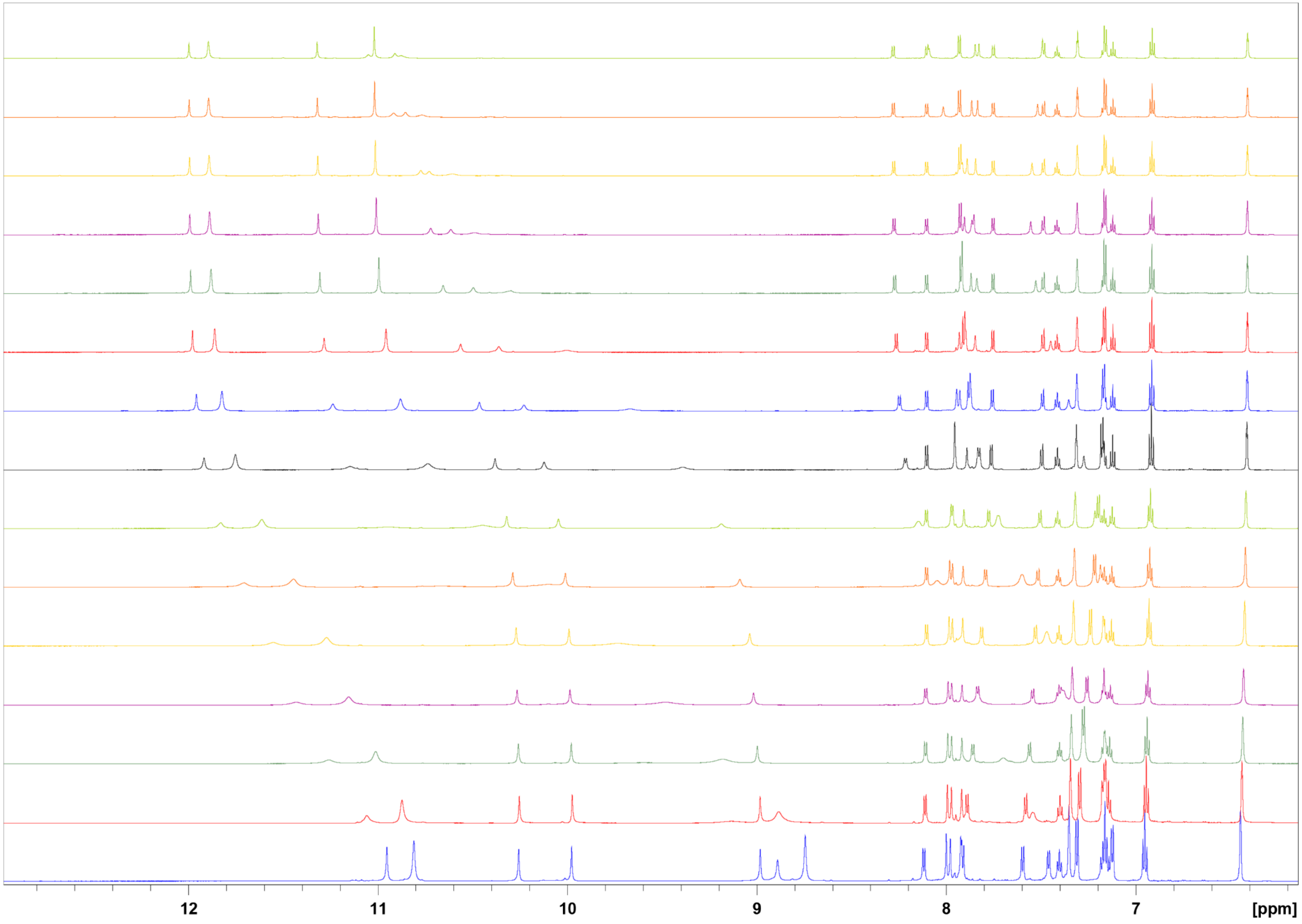

Stacked ^{1}H NMR spectra (700.1 MHz) of a mixture of receptors **MC004**; 3,4,4-Cl_3-diphenylurea; 2,7-$(COOBu)_2$-indolocarbazole; 2,9-$(COOBu)_2$-indolocarbazole + TBA-pivalate

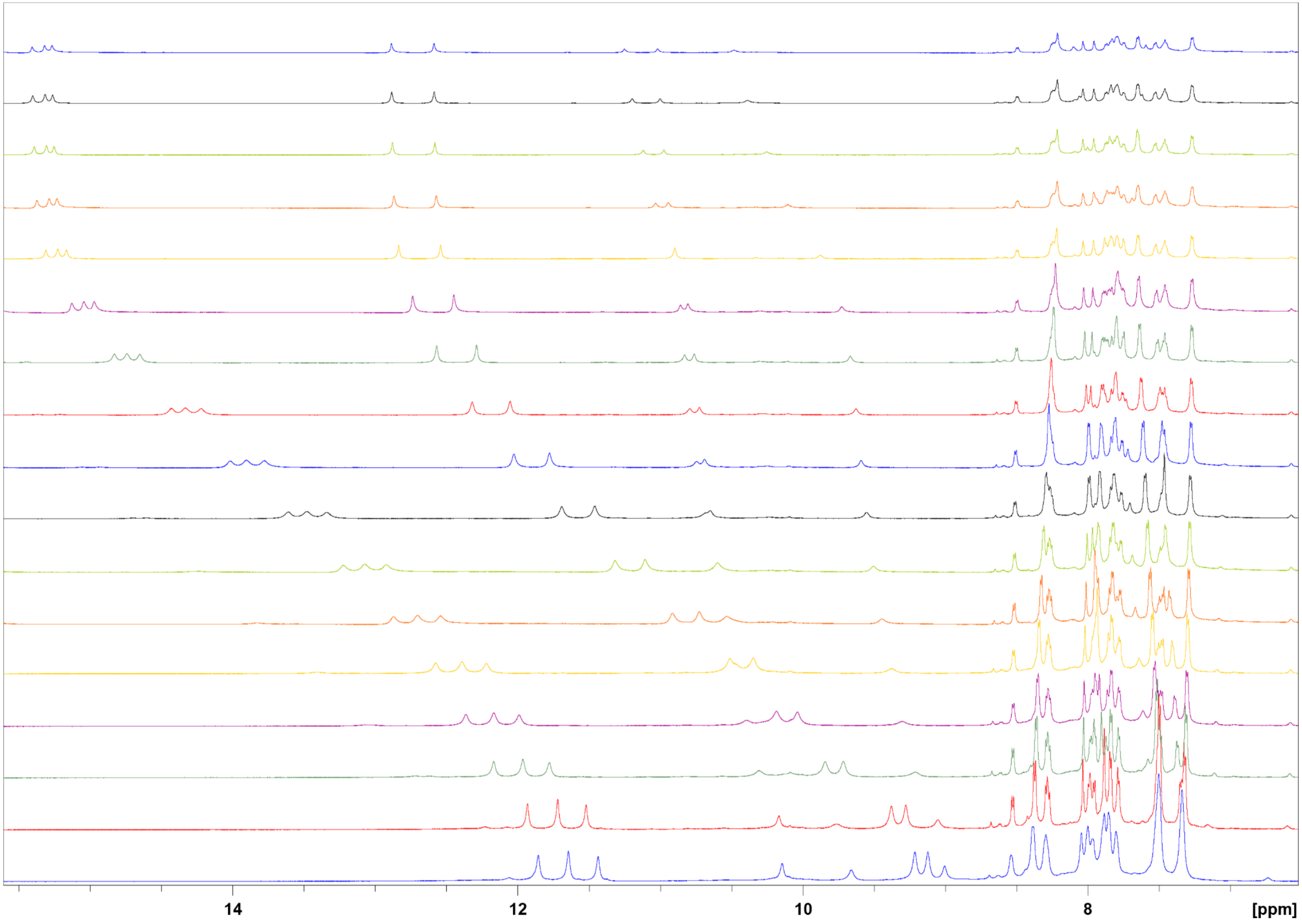

Stacked ^{1}H NMR spectra (700.1 MHz) of a mixture of receptors **MC005**; 1,3-diindolylurea; 1,3-dicarbazolylurea + TBA-pivalate

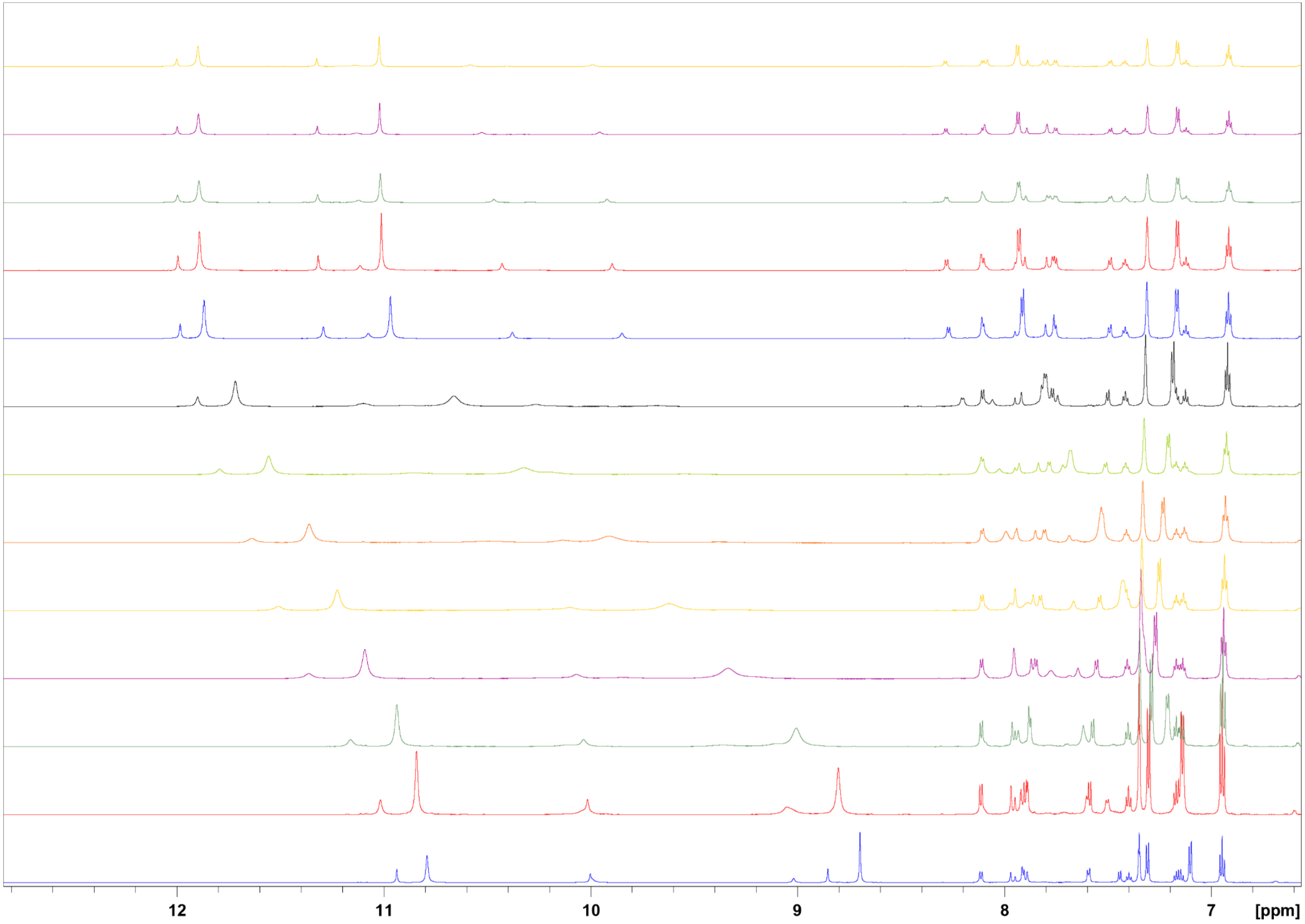

Stacked ^{1}H NMR spectra (700.1 MHz) of a mixture of receptors **MC006**; 2,7-$(COOBu)_2$-indolocarbazole; 2,9-$(COOBu)_2$-indolocarbazole + TBA-pivalate

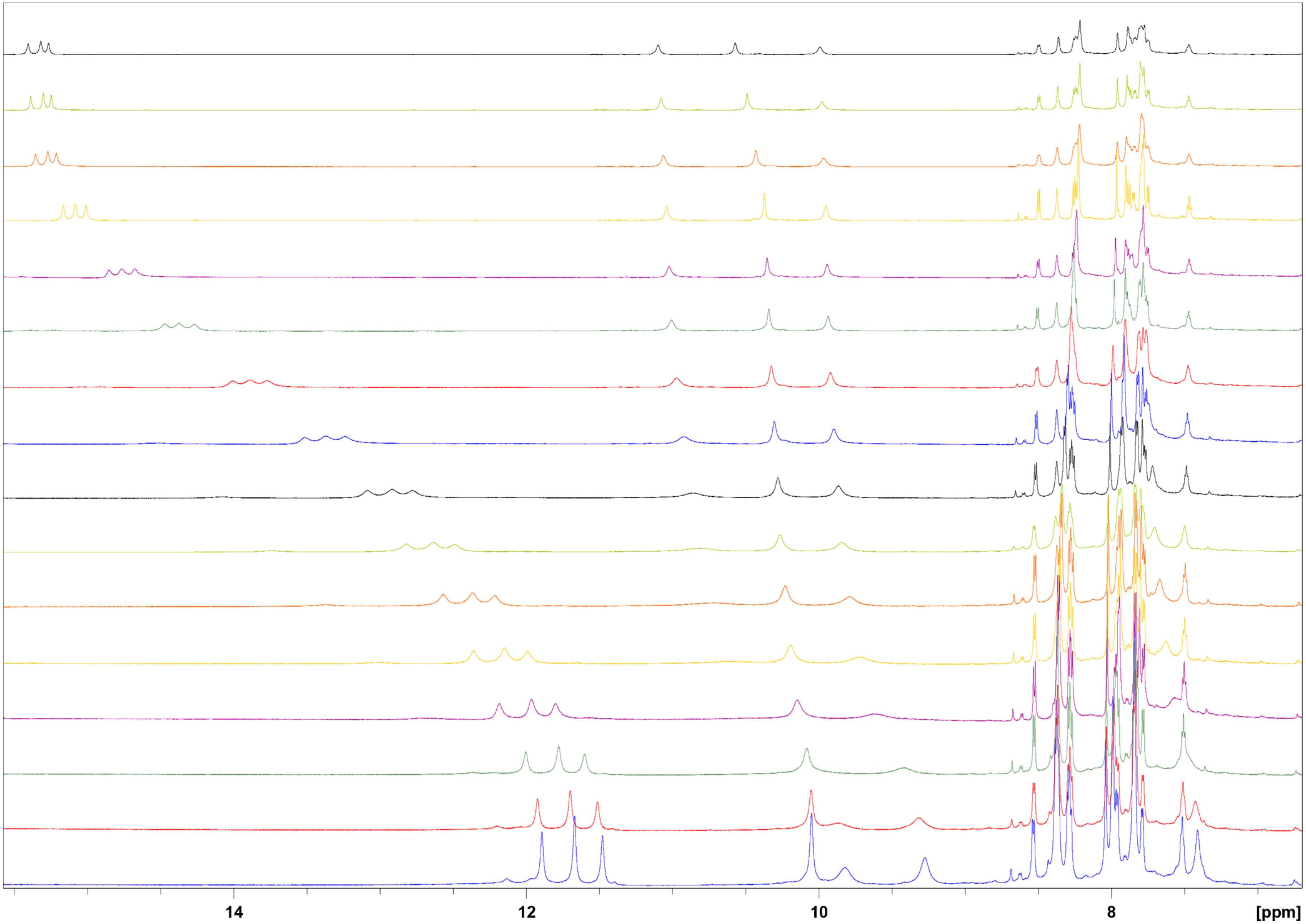

Stacked ^{1}H NMR spectra (700.1 MHz) of a mixture of receptors **MC007**; 1,3-diindolylurea; 1,3-dicarbazolylurea + TBA-pivalate

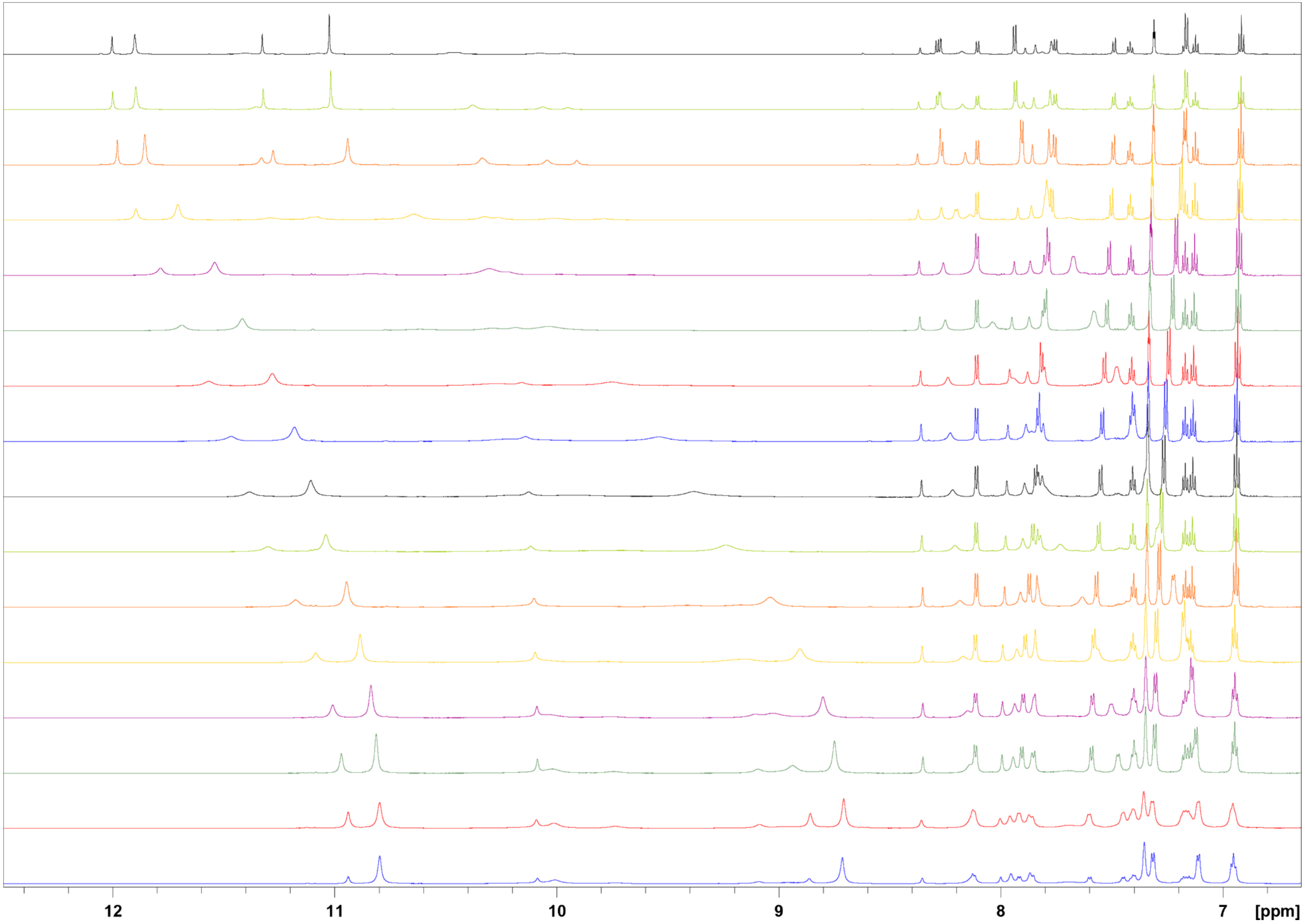

Stacked ^{1}H NMR spectra (700.1 MHz) of a mixture of receptors **MC008**; 1,3-diindolylurea; 1,3-dicarbazolylurea + TBA-pivalate

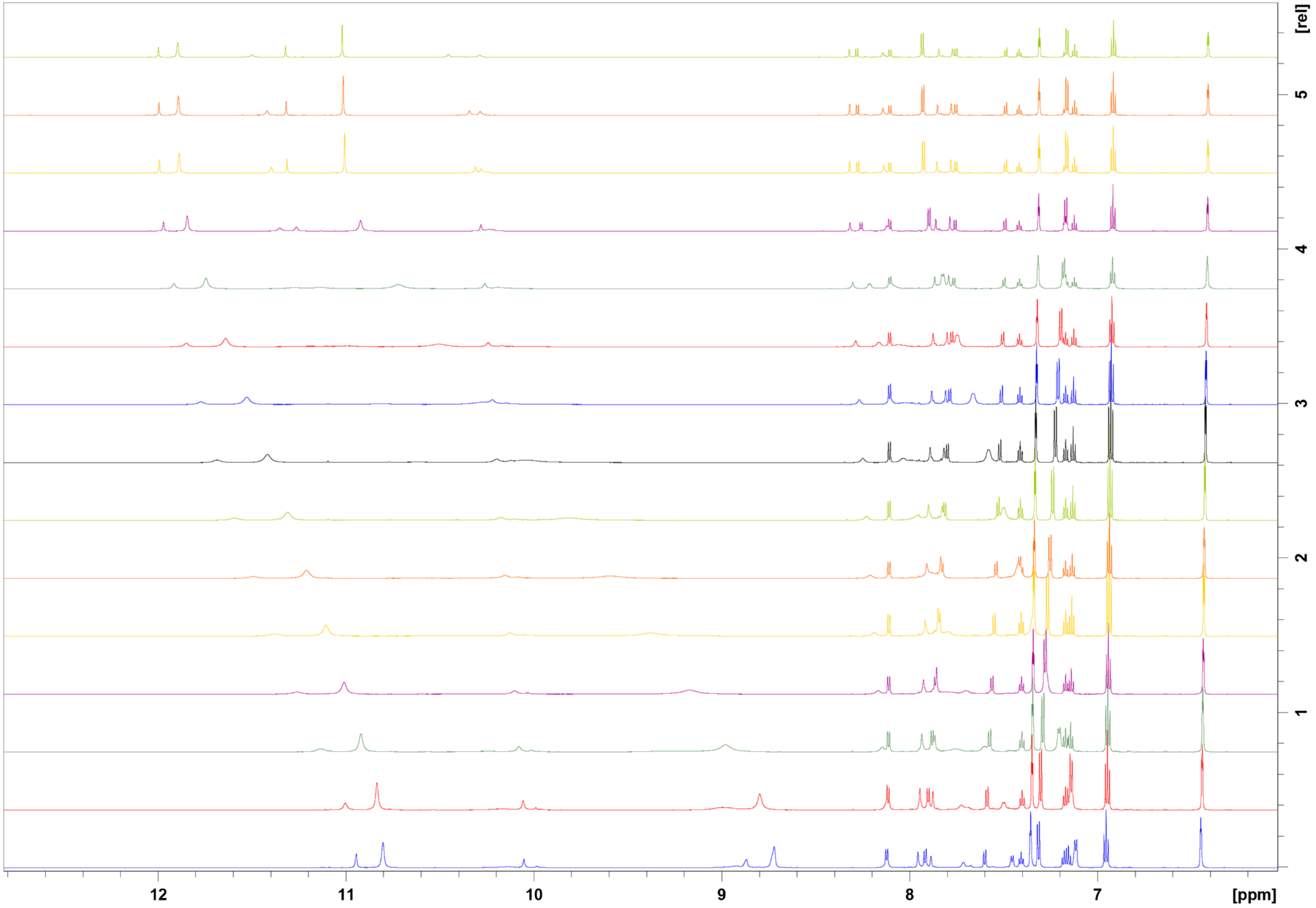

Stacked ^{1}H NMR spectra (700.1 MHz) of a mixture of receptors **MC009**; 1,3-diindolylurea; 1,3-dicarbazolylurea + TBA-pivalate

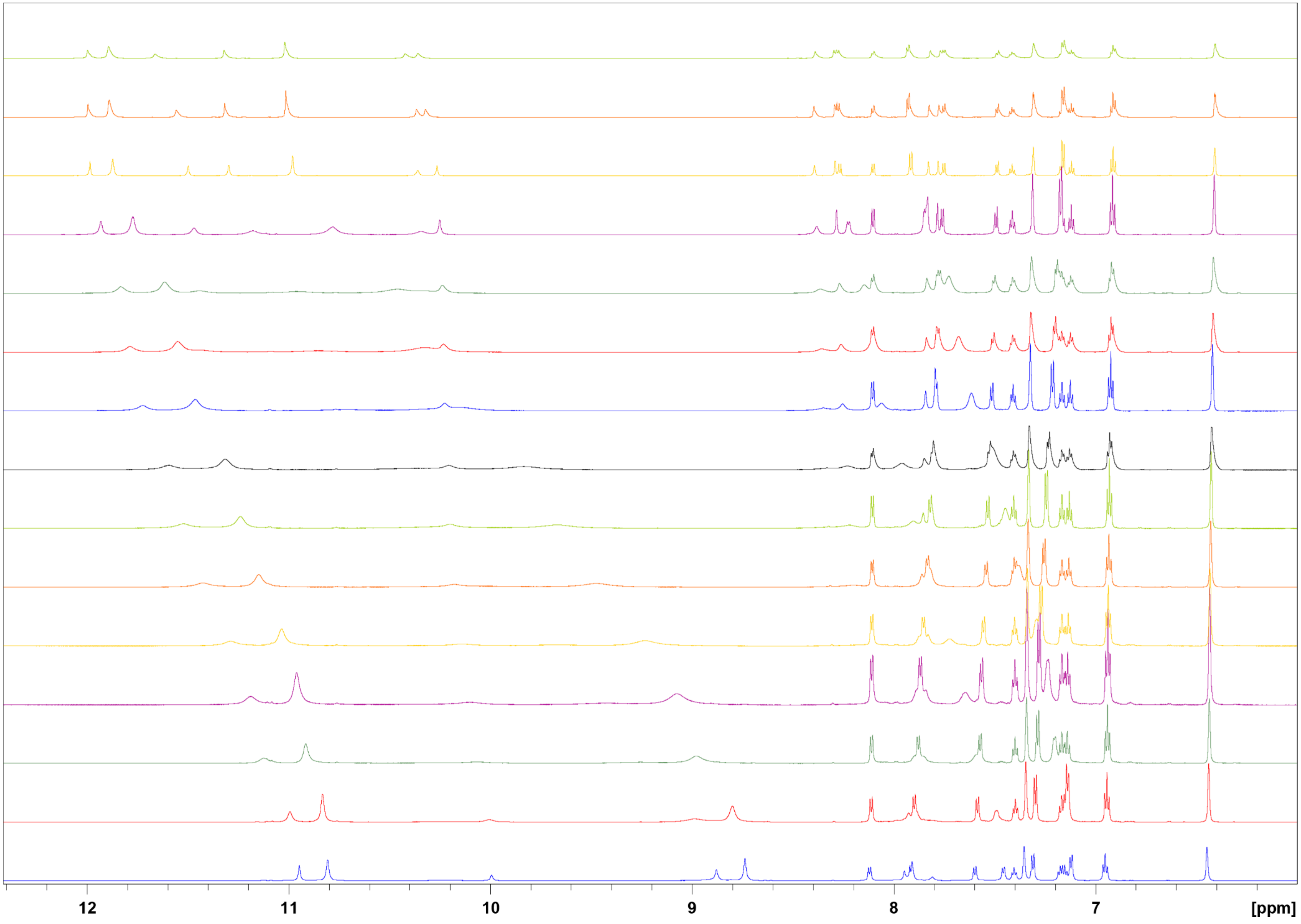

Stacked ^{1}H NMR spectra (700.1 MHz) of a mixture of receptors **MC010**; 1,3-diindolylurea; 1,3-dicarbazolylurea + TBA-pivalate

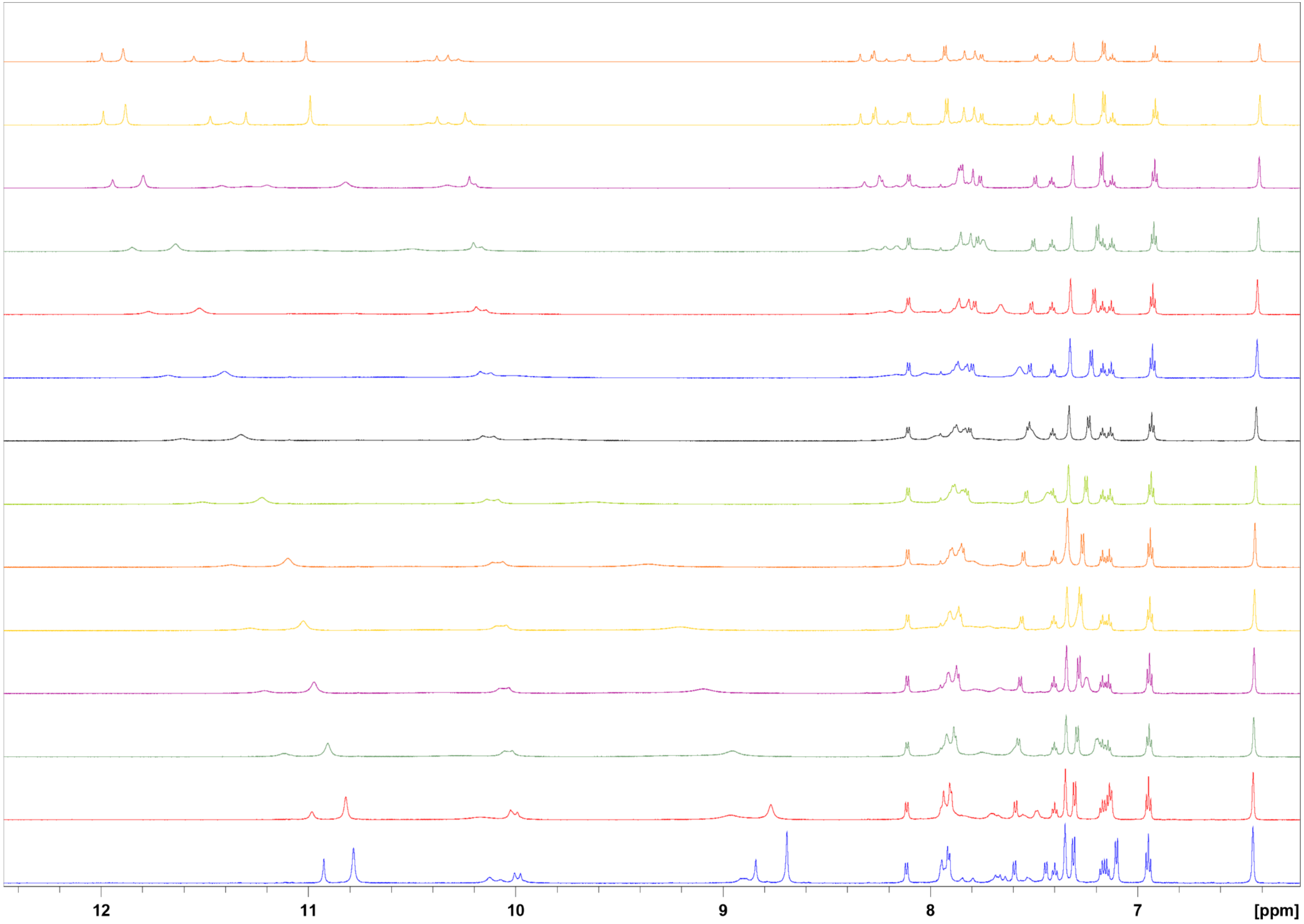

Stacked ^{1}H NMR spectra (700.1 MHz) of a mixture of receptors **MC011**; 1,3-diindolylurea; 1,3-dicarbazolylurea + TBA-pivalate

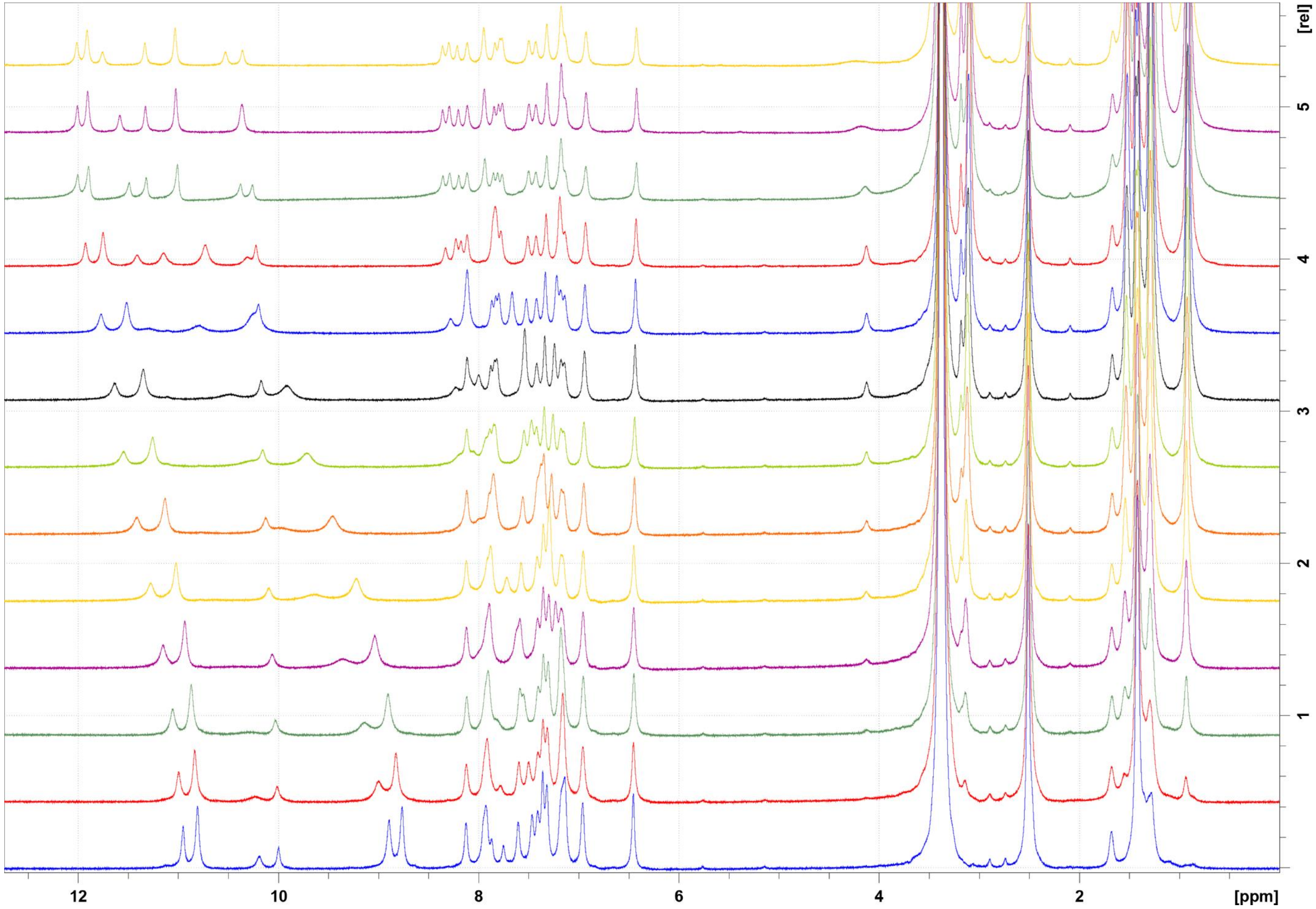

Stacked ^{1}H NMR spectra (700.1 MHz) of a mixture of receptors **MC012**; 1,3-diindolylurea; 1,3-dicarbazolylurea + TBA-pivalate

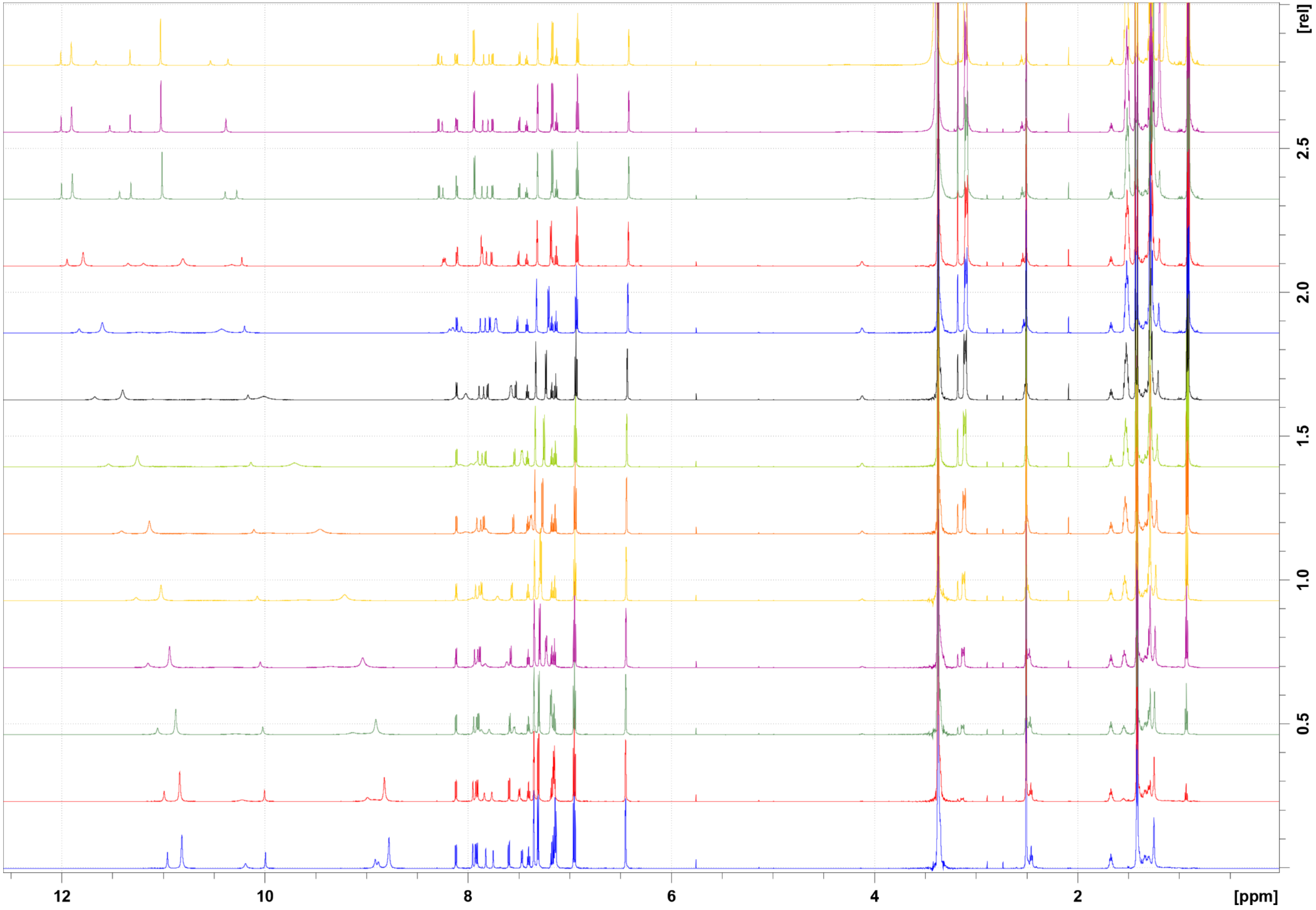

Stacked ^{1}H NMR spectra (700.1 MHz) of a mixture of receptors **MC013**; 1,3-diindolylurea; 1,3-dicarbazolylurea + TBA-pivalate

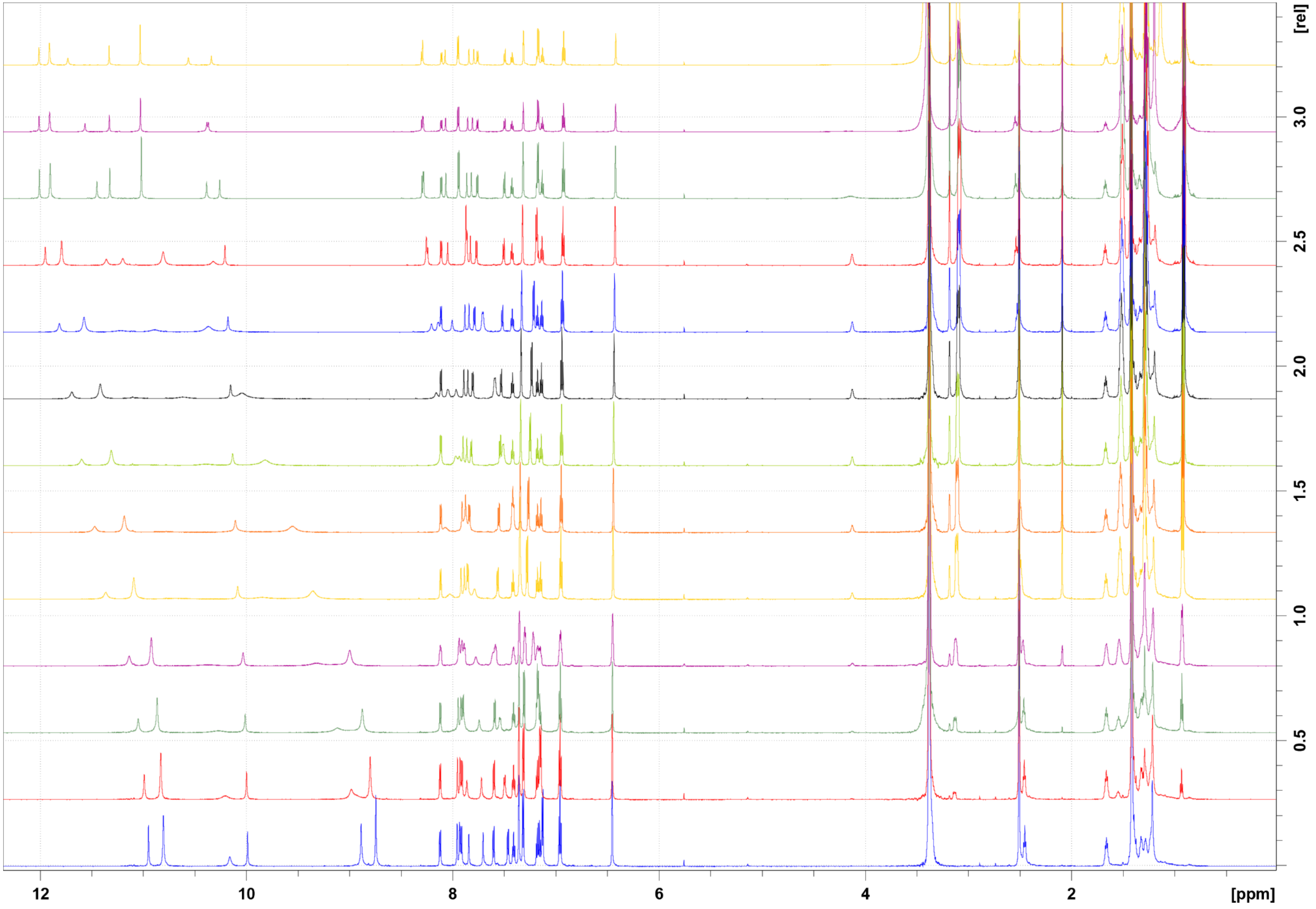

Stacked ^{1}H NMR spectra (700.1 MHz) of a mixture of receptors **MC014**; 1,3-diindolylurea; 1,3-dicarbazolylurea + TBA-pivalate

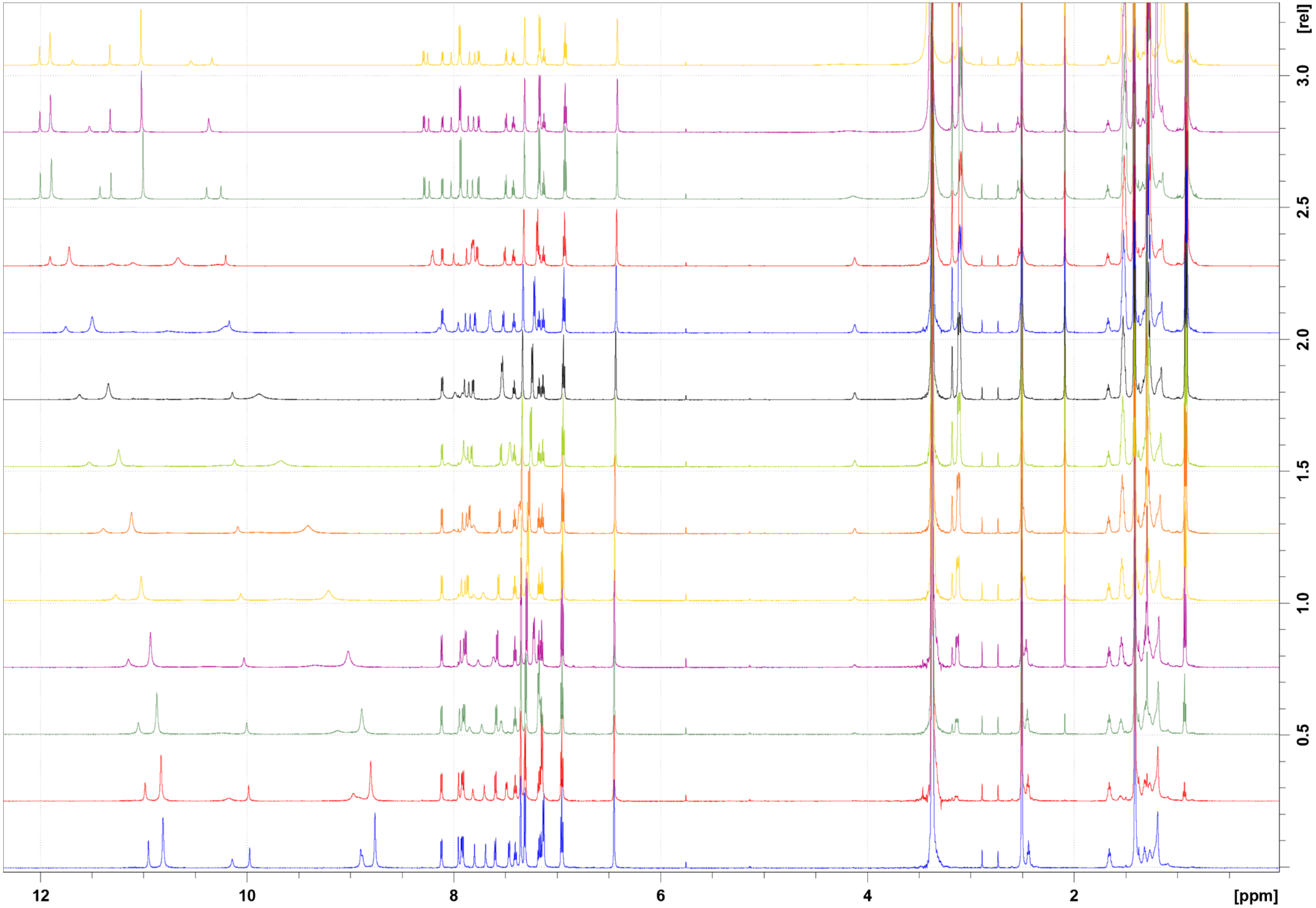

UV–vis spectra of absolute binding affinity measurement for **MC002** with TBA-lactate in 99.5%:0.5% m/m DMSO-H_2O

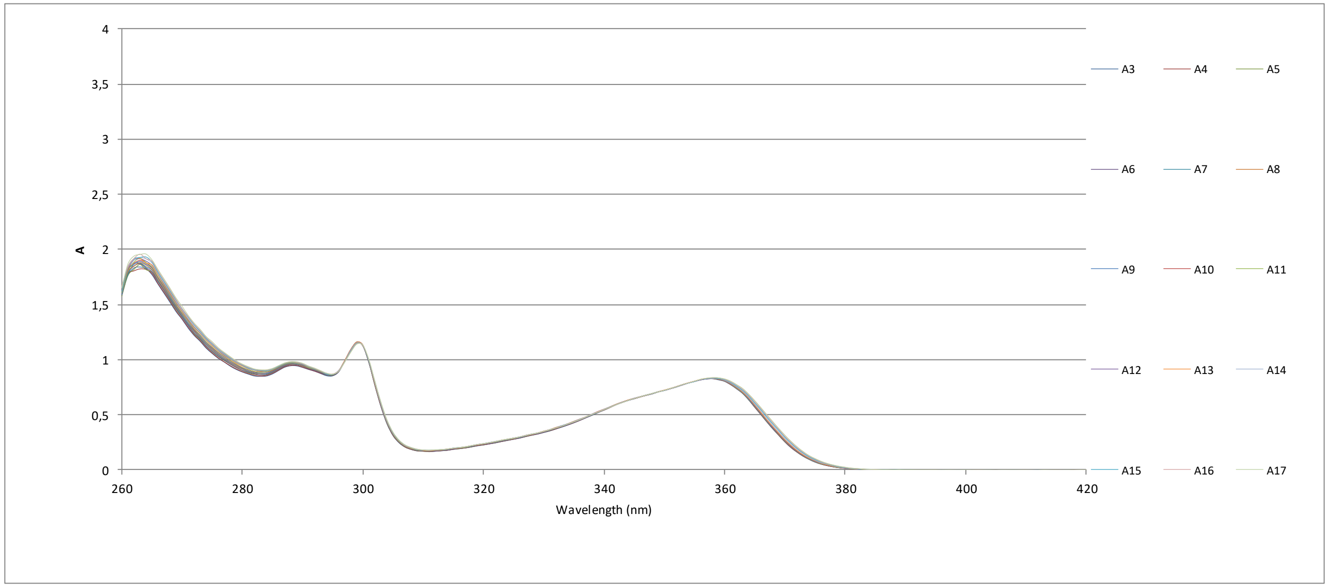

UV–vis spectra of absolute binding affinity measurement for **MC002** with TBA-pivalate 99.5%:0.5% m/m DMSO-H_2O

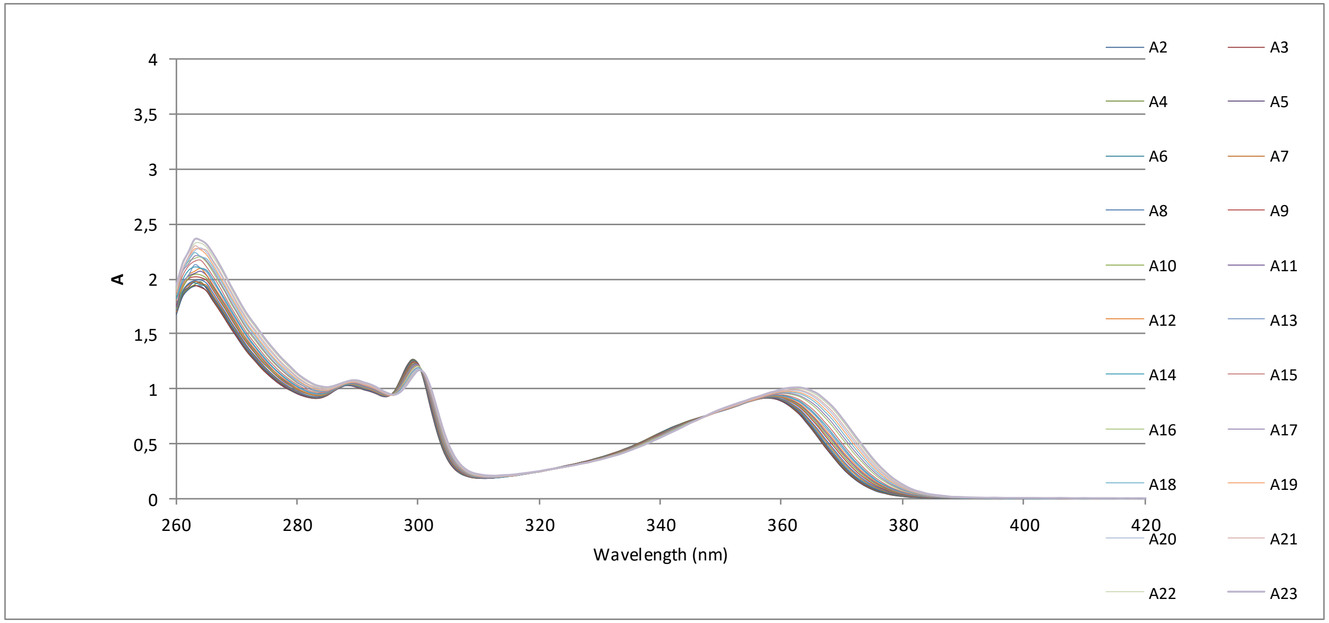

UV–vis spectra of absolute binding affinity measurement for **MC004** with TBA-acetate 90.0%:10.0% m/m DMSO-H_2O

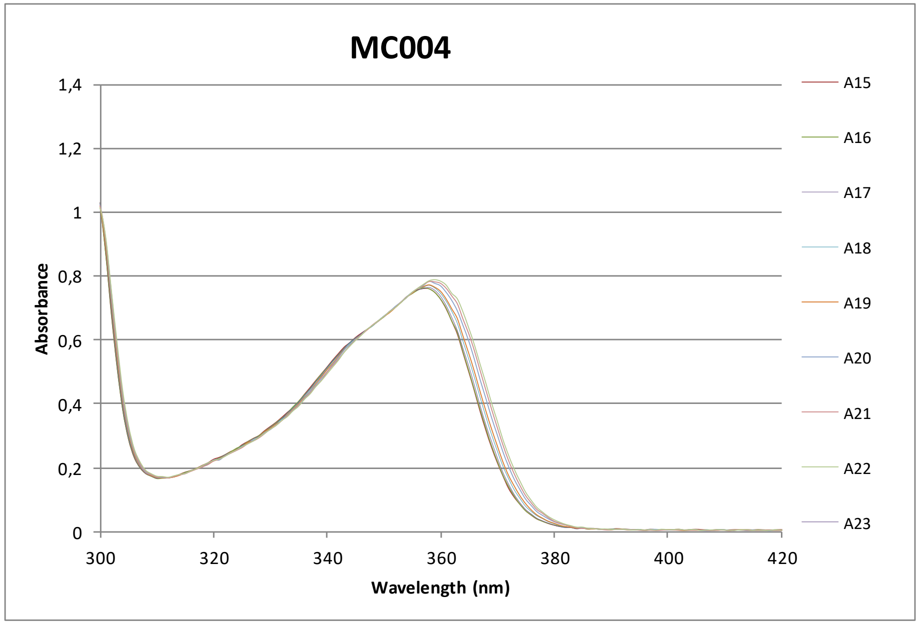

UV–vis spectra of absolute binding affinity measurement for **MC005** with TBA-acetate 90.0%:10.0% m/m DMSO-H_2O

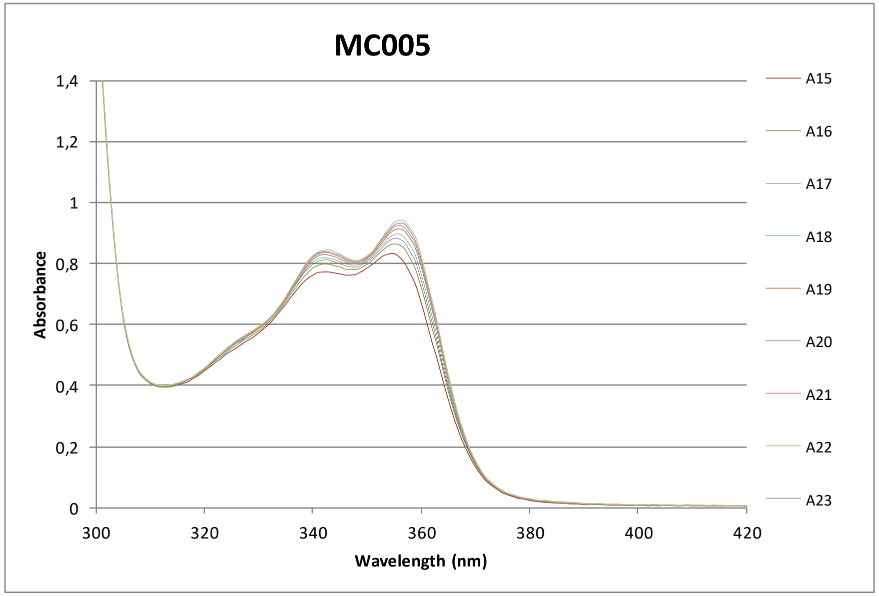

UV–vis spectra of absolute binding affinity measurement for **MC007** with TBA-acetate 90.0%:10.0% m/m DMSO-H_2O

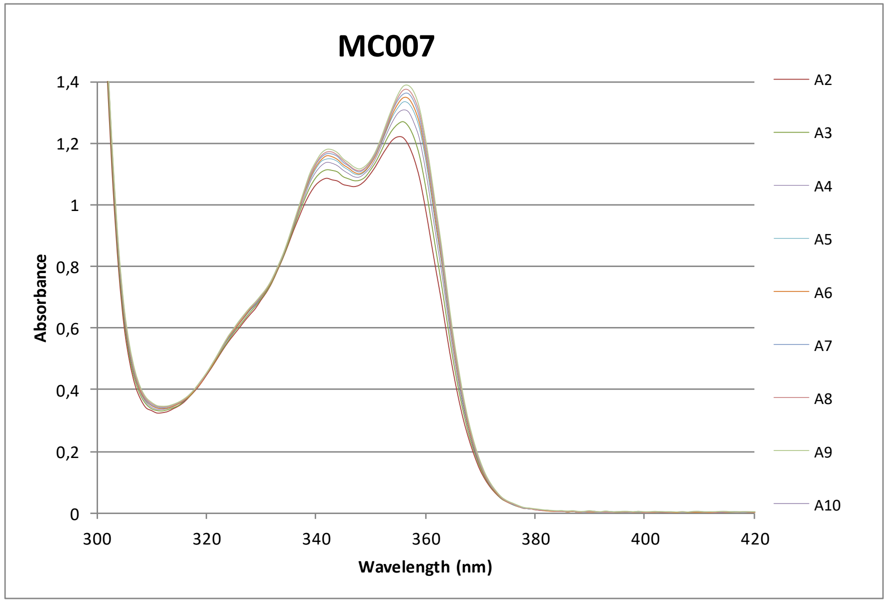

UV–vis spectra of absolute binding affinity measurement for **MC008** with TBA-acetate 90.0%:10.0% m/m DMSO-H_2O

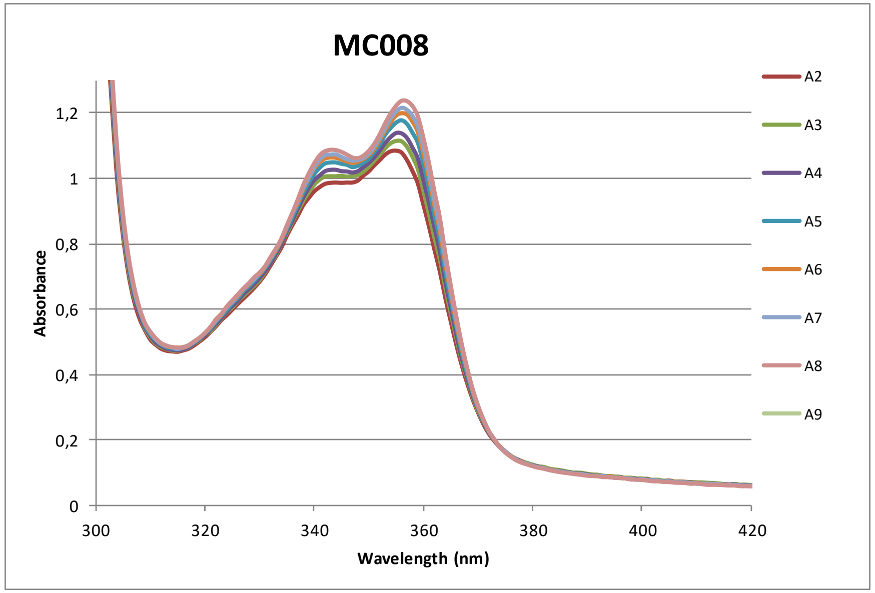

UV–vis spectra of absolute binding affinity measurement for **MC009** with TBA-acetate 90.0%:10.0% m/m DMSO-H_2O

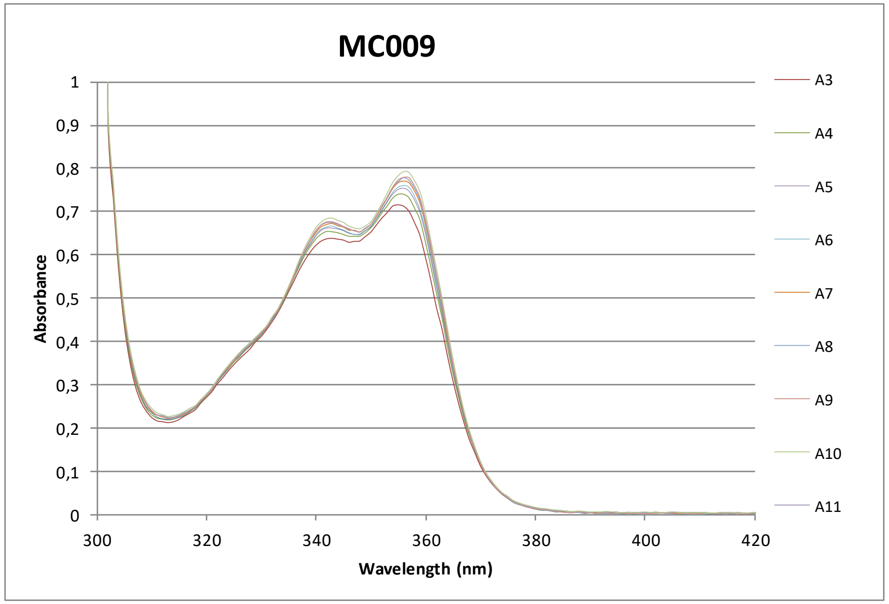

UV–vis spectra of absolute binding affinity measurement for **MC004** with TBA-benzoate 90.0%:10.0% m/m DMSO-H_2O

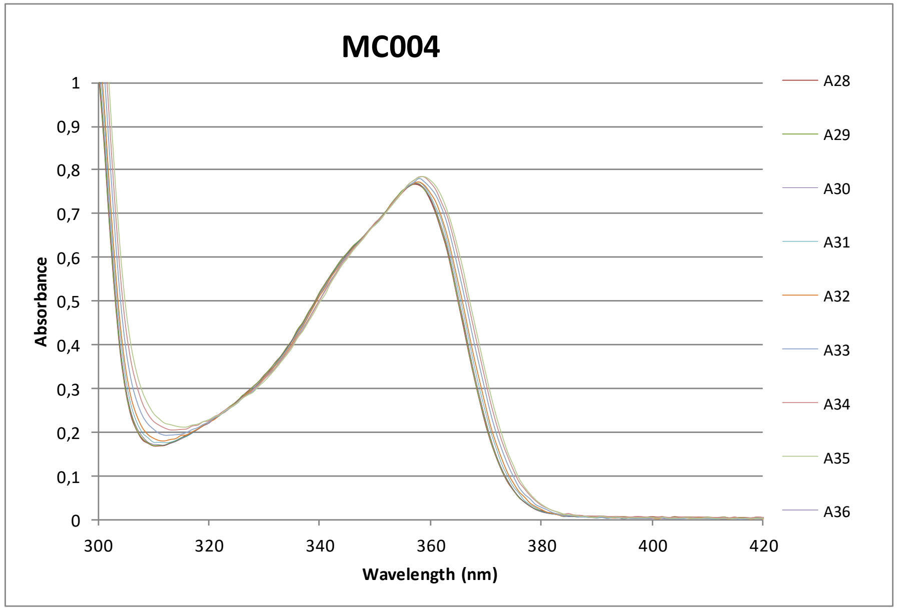

UV–vis spectra of absolute binding affinity measurement for **MC005** with TBA-benzoate 90.0%:10.0% m/m DMSO-H_2O

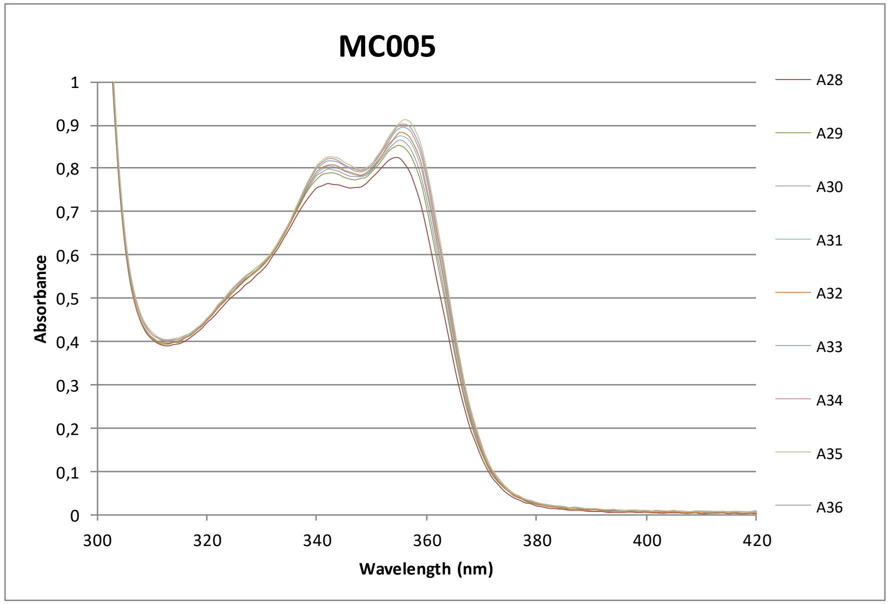

UV–vis spectra of absolute binding affinity measurement for **MC007** with TBA-benzoate 90.0%:10.0% m/m DMSO-H_2O

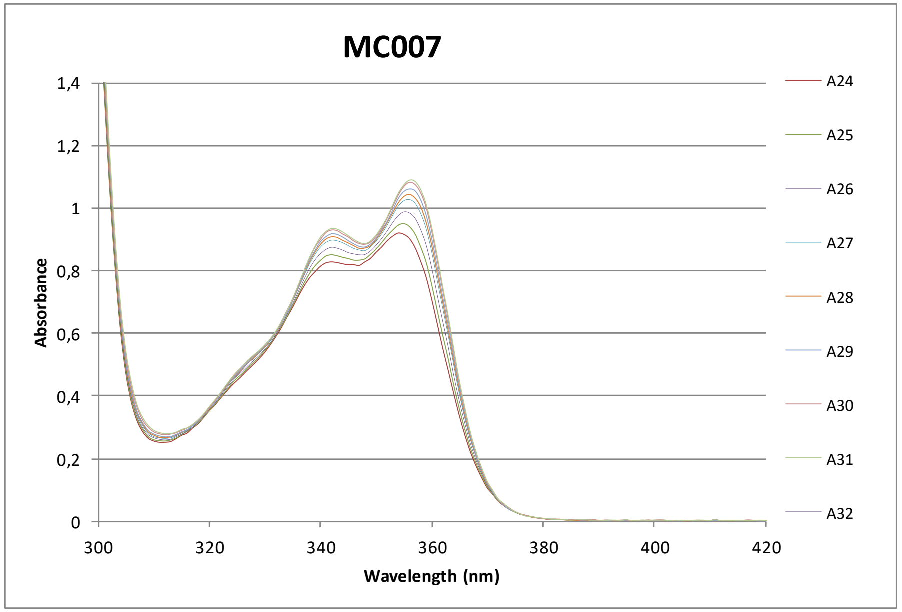

UV–vis spectra of absolute binding affinity measurement for **MC008** with TBA-benzoate 90.0%:10.0% m/m DMSO-H_2O

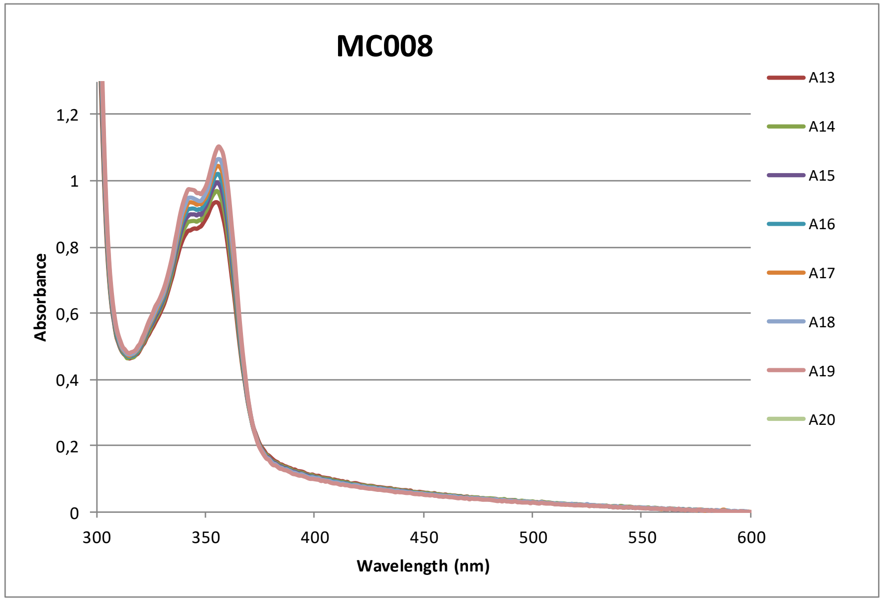

UV–vis spectra of absolute binding affinity measurement for **MC009** with TBA-benzoate 90.0%:10.0% m/m DMSO-H_2O

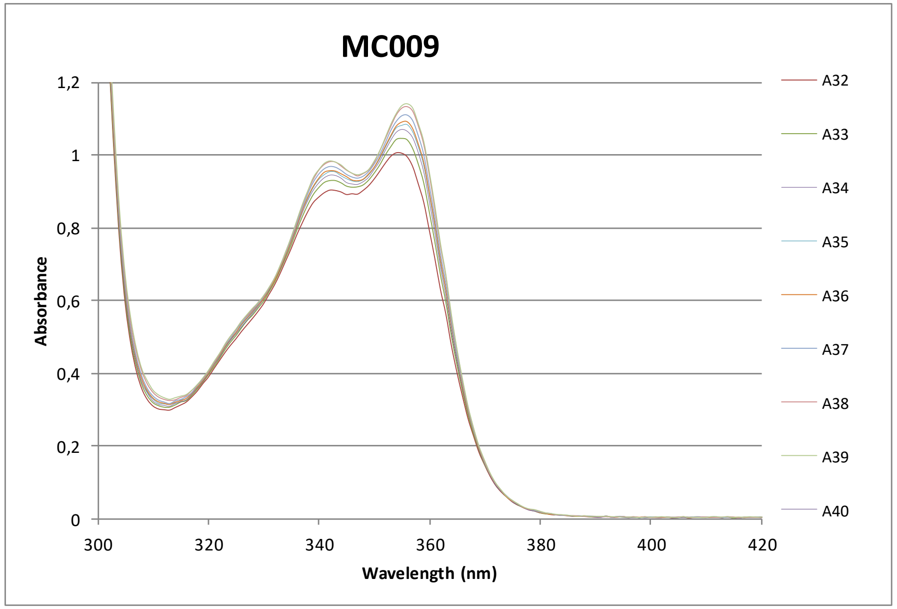

UV–vis spectra of absolute binding affinity measurement for **MC004** with TBA-formate 90.0%:10.0% m/m DMSO-H_2O

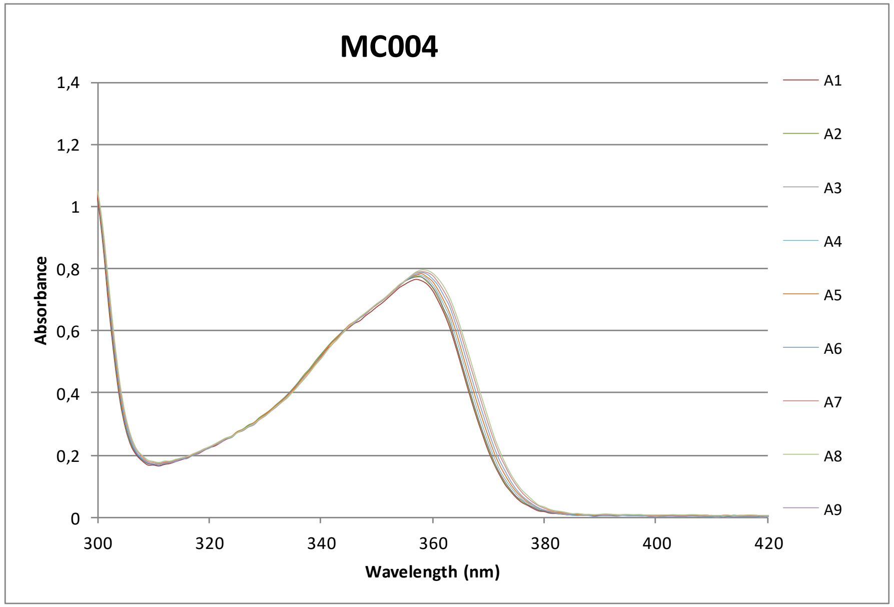

UV–vis spectra of absolute binding affinity measurement for **MC005** with TBA-formate 90.0%:10.0% m/m DMSO-H_2O

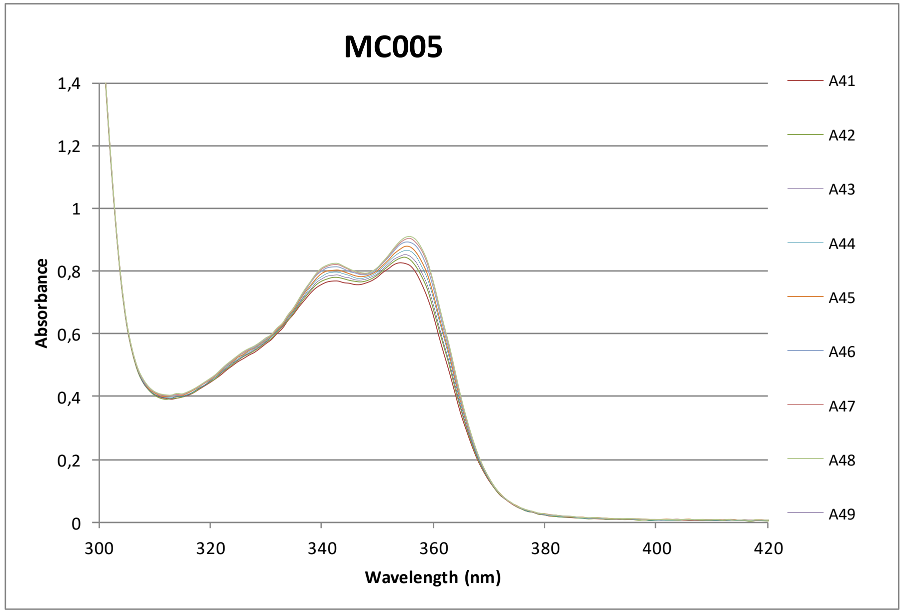

UV–vis spectra of absolute binding affinity measurement for **MC007** with TBA-formate 90.0%:10.0% m/m DMSO-H_2O

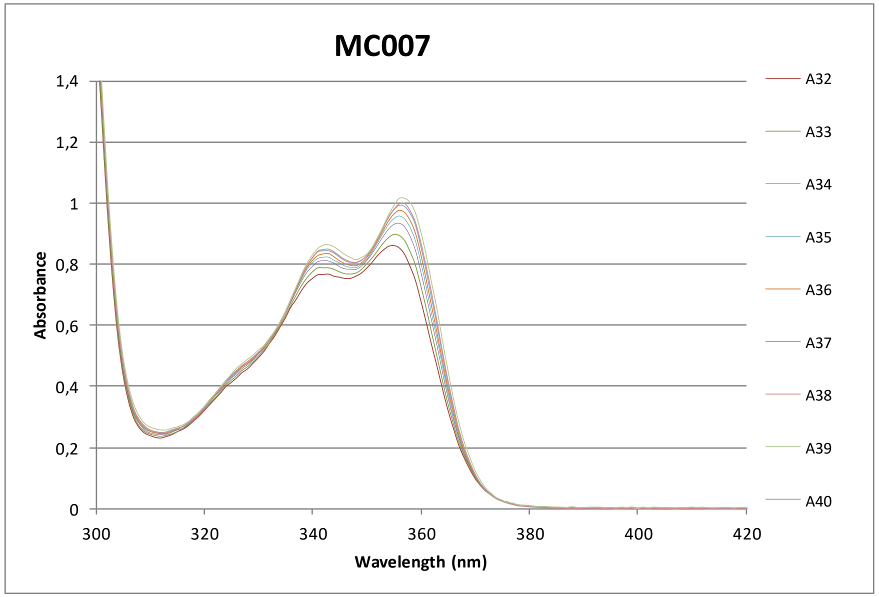

UV–vis spectra of absolute binding affinity measurement for **MC008** with TBA-formate 90.0%:10.0% m/m DMSO-H_2O

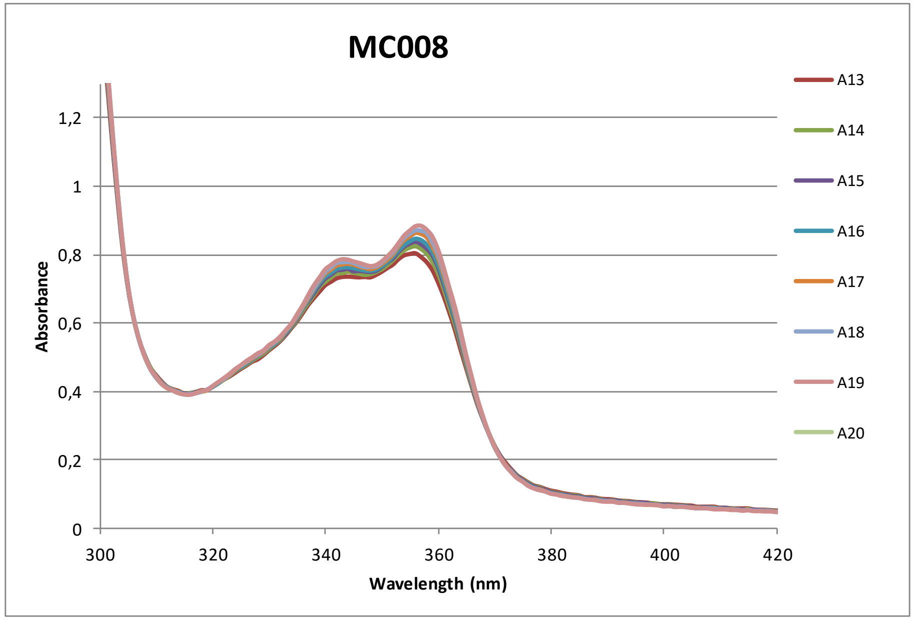

UV–vis spectra of absolute binding affinity measurement for **MC009** with TBA-formate 90.0%:10.0% m/m DMSO-H_2O

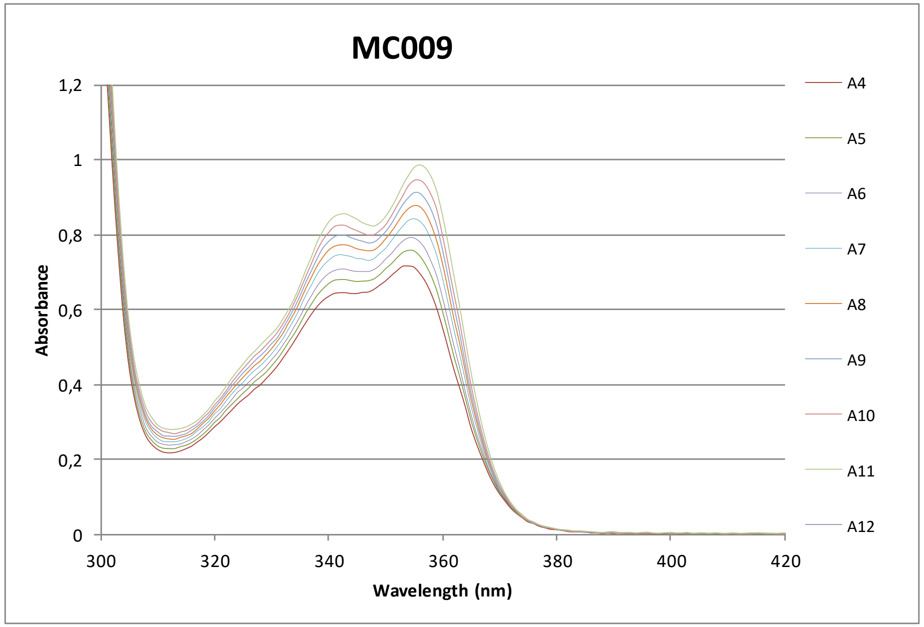

UV–vis spectra of absolute binding affinity measurement for **MC004** with TBA-lactate 90.0%:10.0% m/m DMSO-H_2O

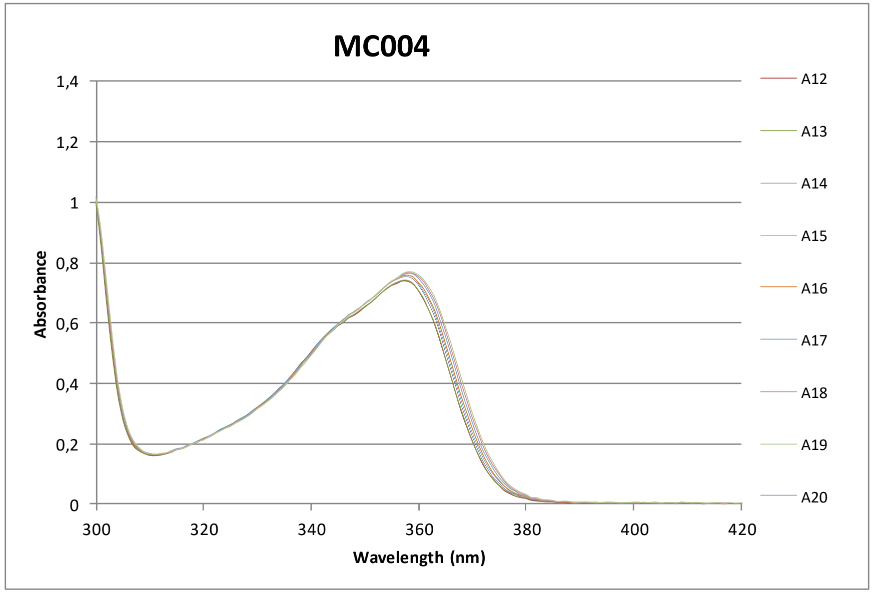

UV–vis spectra of absolute binding affinity measurement for **MC005** with TBA-lactate 90.0%:10.0% m/m DMSO-H_2O

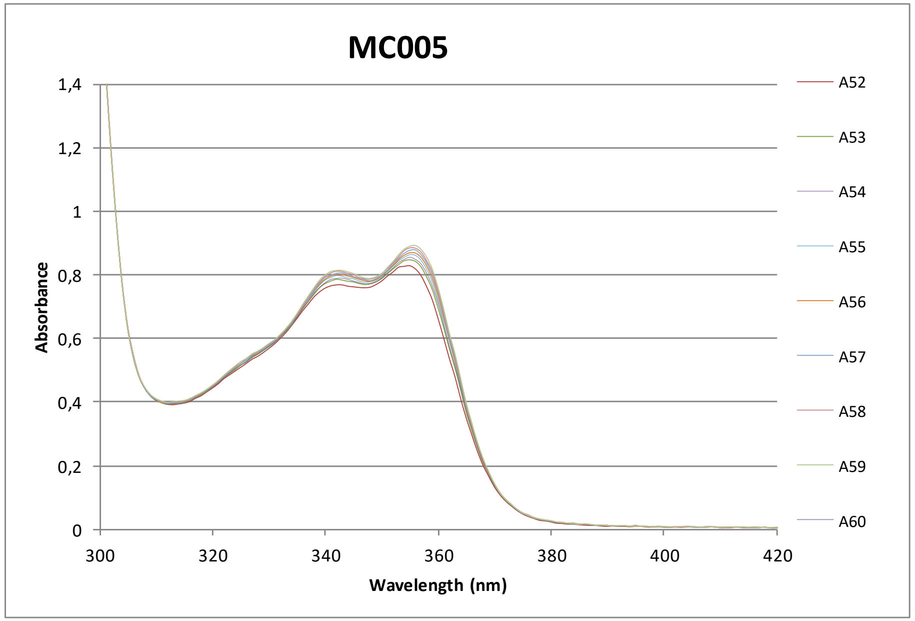

UV–vis spectra of absolute binding affinity measurement for **MC007** with TBA-lactate 90.0%:10.0% m/m DMSO-H_2O

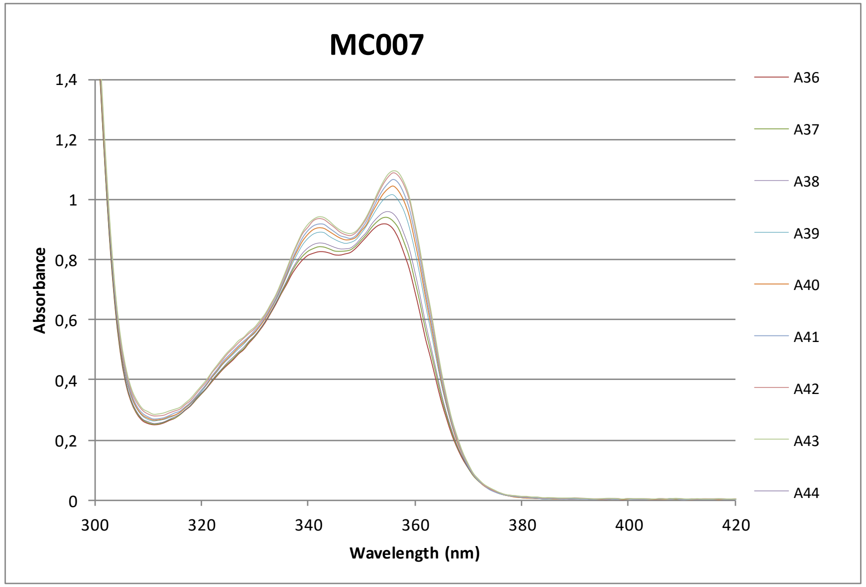

UV–vis spectra of absolute binding affinity measurement for **MC008** with TBA-lactate 90.0%:10.0% m/m DMSO-H_2O

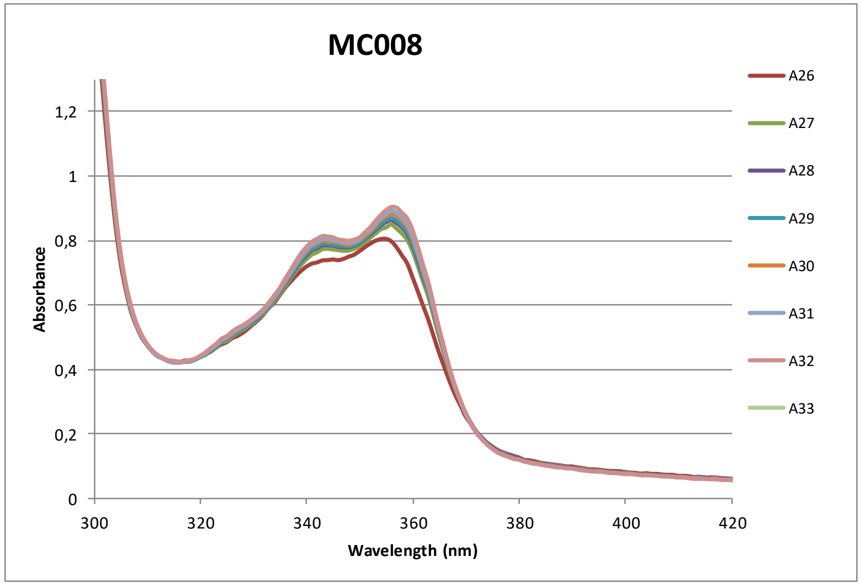

UV–vis spectra of absolute binding affinity measurement for **MC009** with TBA-lactate 90.0%:10.0% m/m DMSO-H_2O

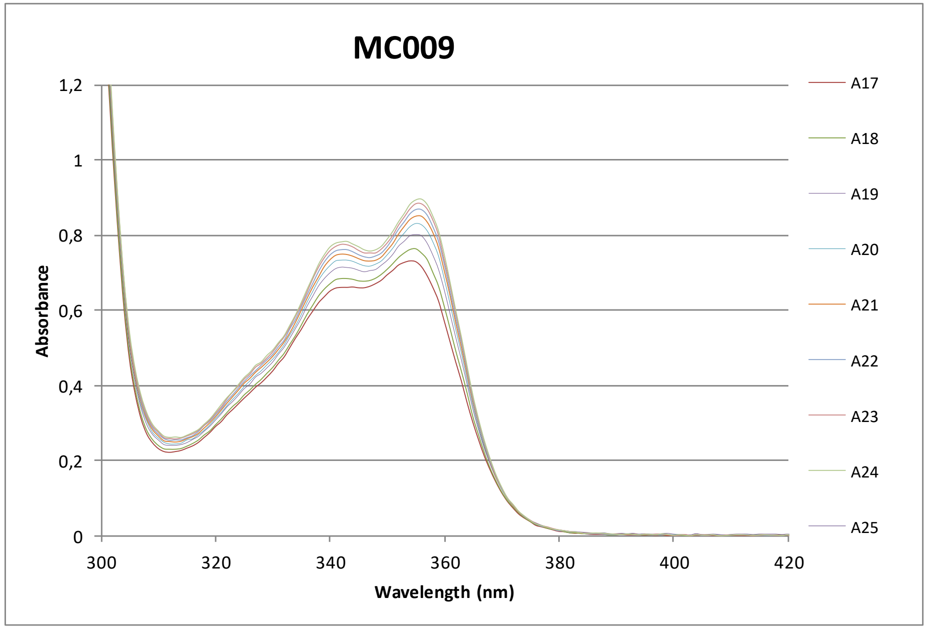

UV–vis spectra of absolute binding affinity measurement for **MC004** with TBA-pivalate 90.0%:10.0% m/m DMSO-H_2O

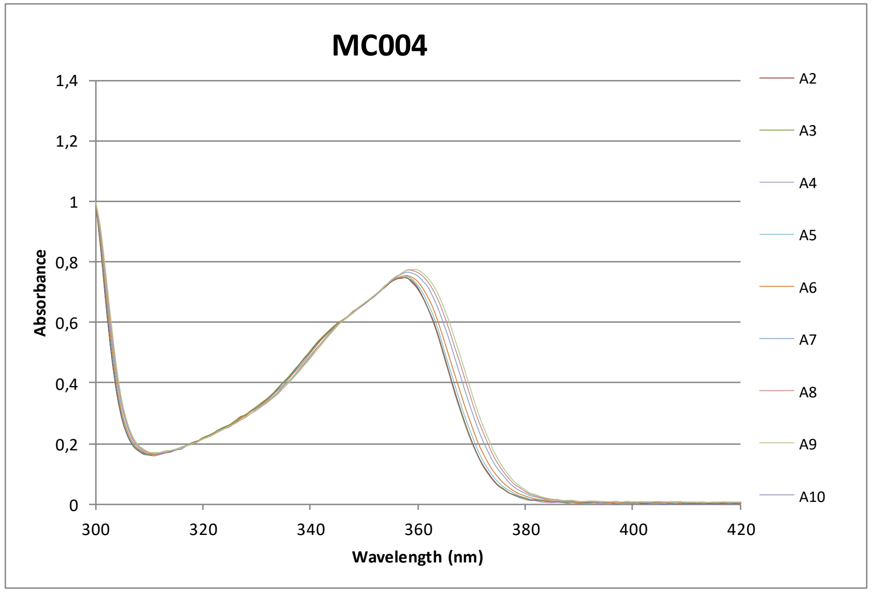

UV–vis spectra of absolute binding affinity measurement for **MC005** with TBA-pivalate 90.0%:10.0% m/m DMSO-H_2O

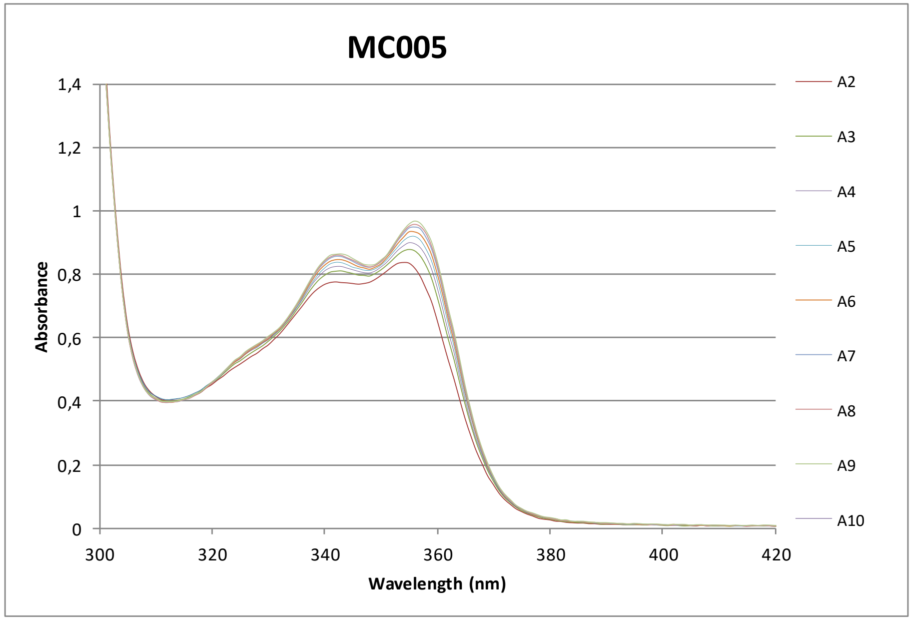

UV–vis spectra of absolute binding affinity measurement for **MC007** with TBA-pivalate 90.0%:10.0% m/m DMSO-H_2O

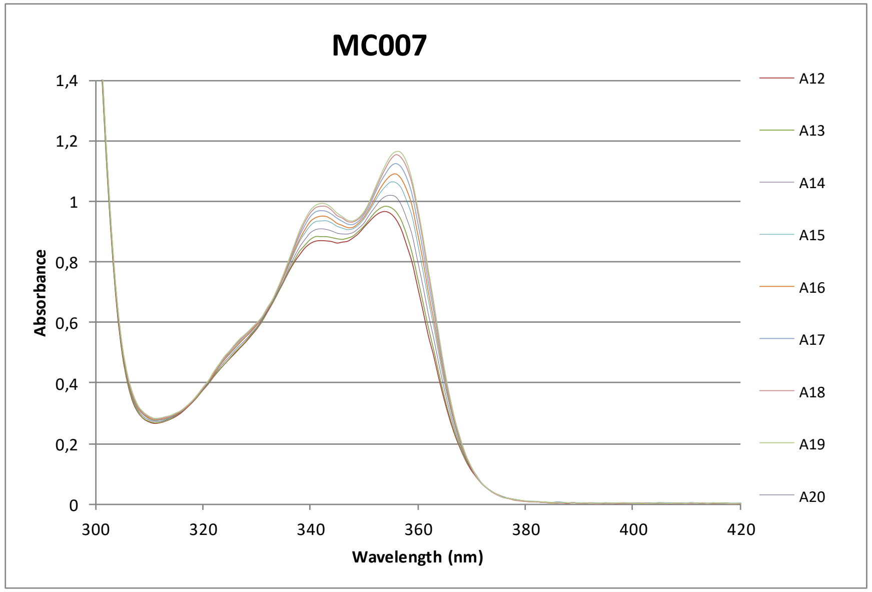

UV–vis spectra of absolute binding affinity measurement for **MC008** with TBA-pivalate 90.0%:10.0% m/m DMSO-H_2O

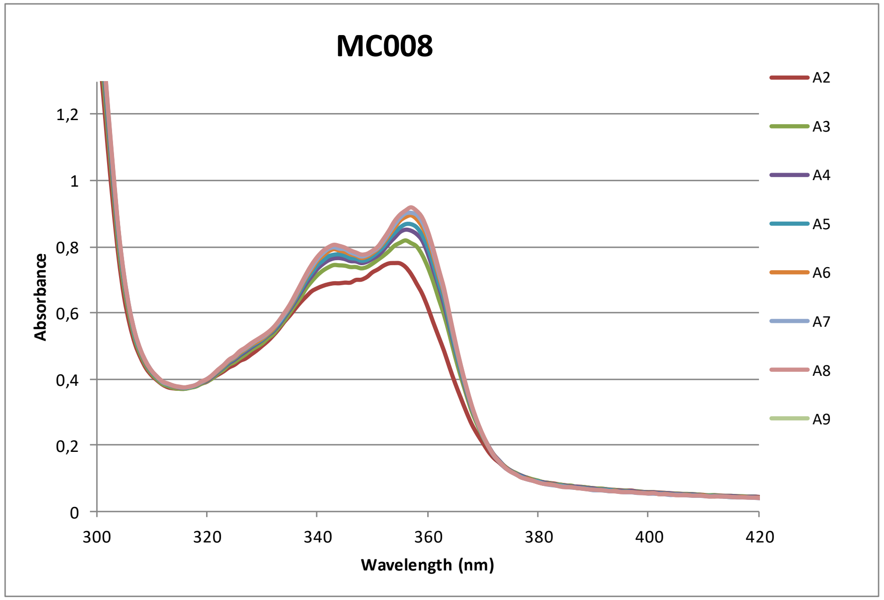

UV–vis spectra of absolute binding affinity measurement for **MC009** with TBA-pivalate 90.0%:10.0% m/m DMSO-H2O

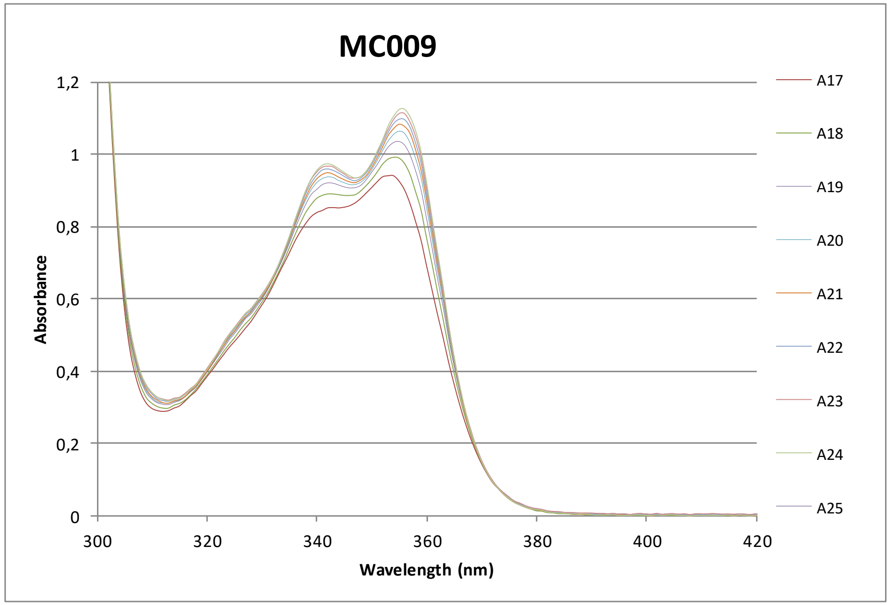

Lowest energy conformation of receptor **CZ016**

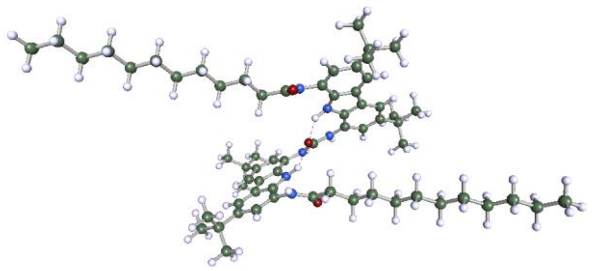

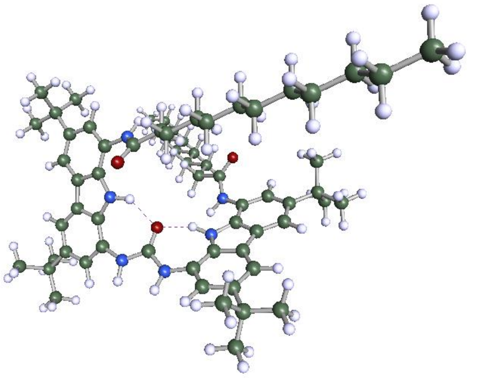

G = -1939212.08127 kcal/mol

Lowest energy conformation of receptor **CZ016** with pivalate anion

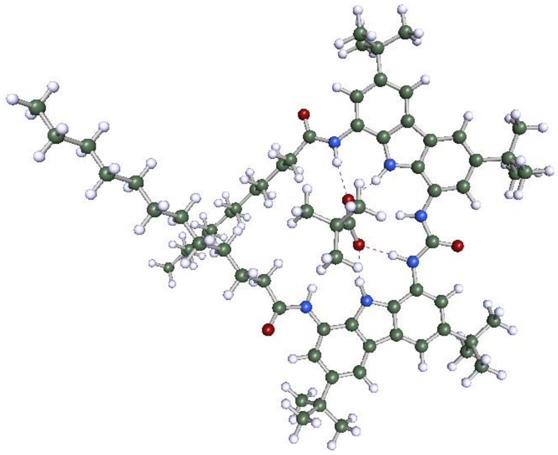

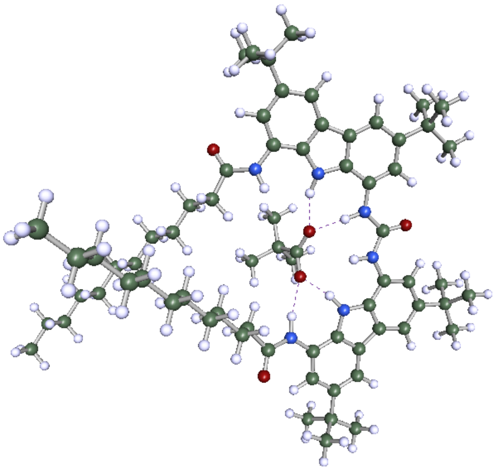

G = -2156792.28741 kcal/mol

Lowest energy conformation of receptor **CZ016** with acetate anion

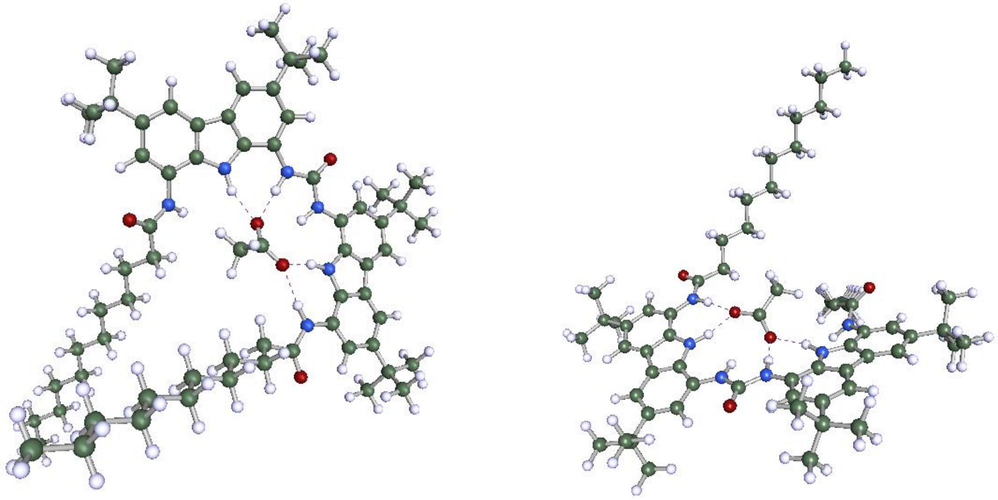

G = -2082766.14645 kcal/mol

Lowest energy conformation of receptor **CZ016** with benzoate anion

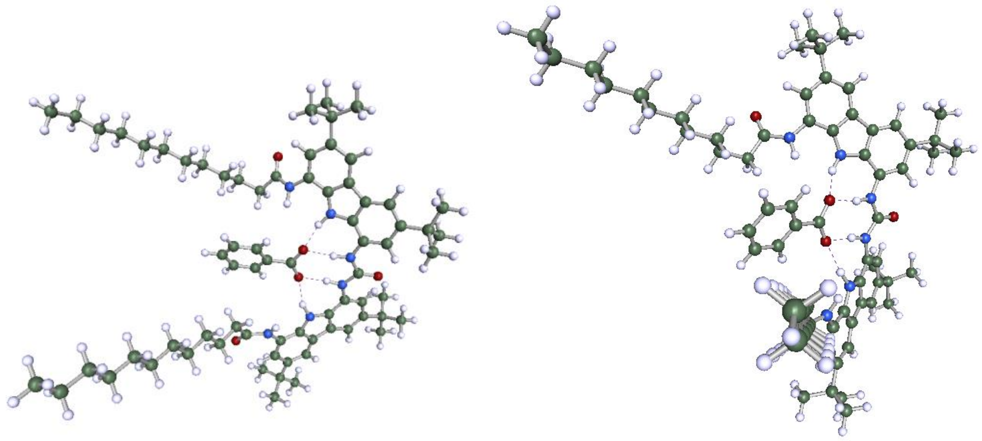

G = 2203124.50120 kcal/mol

Lowest energy conformation of receptor **CZ016** with lactate anion

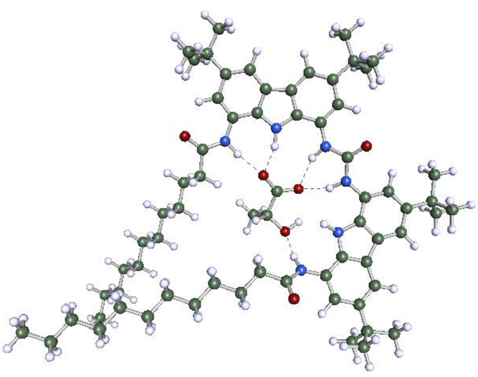

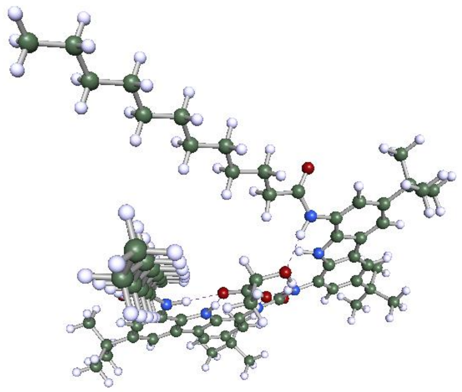

G = -2154669.70262 kcal/mol

Lowest energy conformation of receptor **CZ016** with formate anion

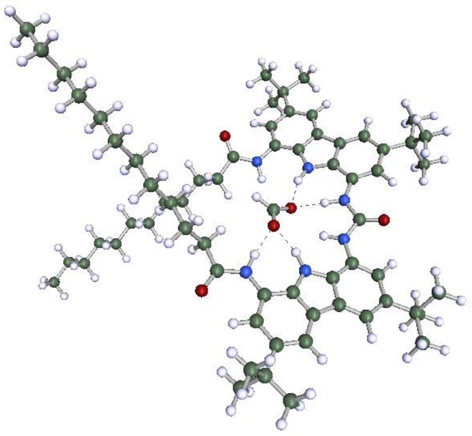

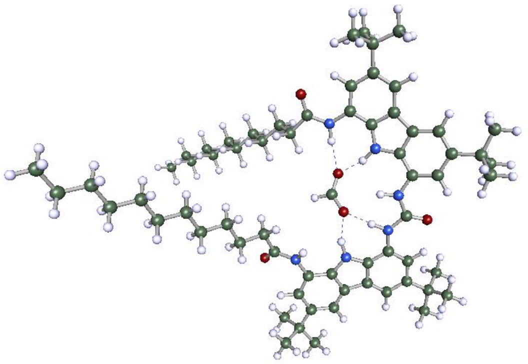

G = -2058084.08381 kcal/mol

Lowest energy conformation of receptor **MC001**

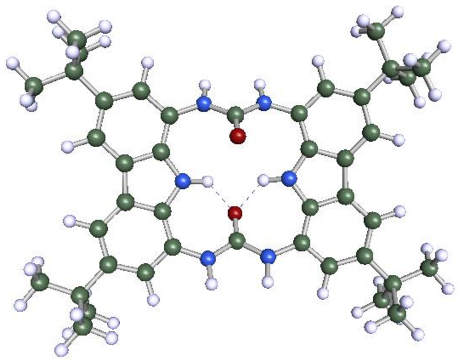

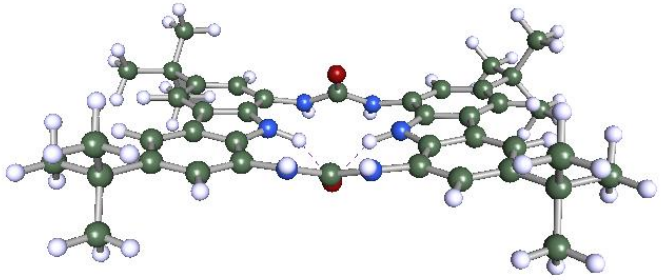

G = -1324377.24934 kcal/mol

Lowest energy conformation of receptor **MC001** with pivalate anion

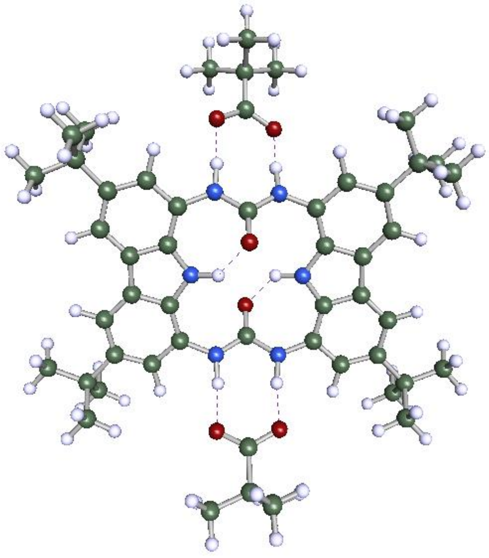

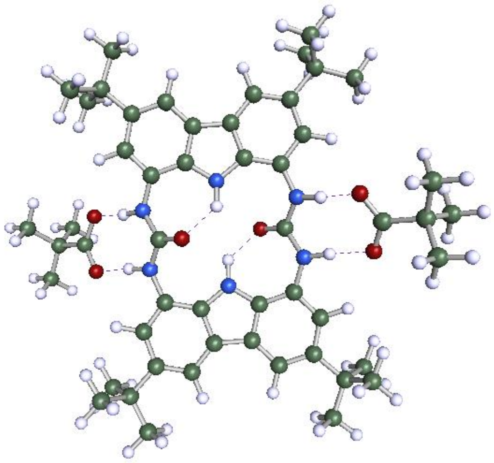

G = -1759540.52493 kcal/mol

Lowest energy conformation of receptor **MC001** with acetate anion

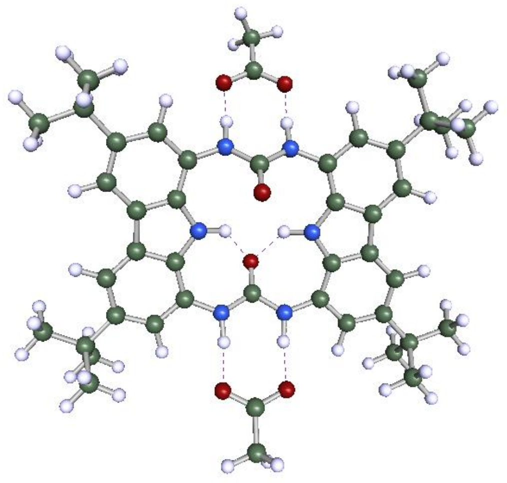

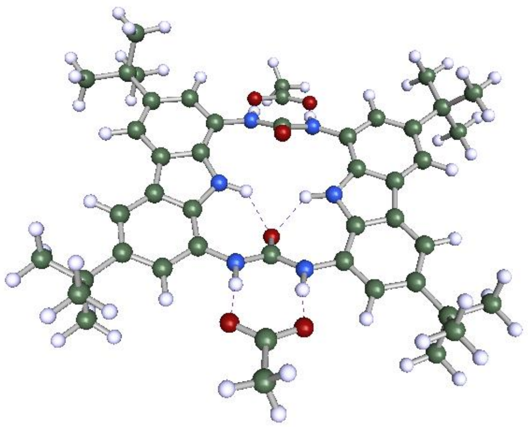

G = -1611476.38251 kcal/mol

Lowest energy conformation of receptor **MC001** with benzoate anion

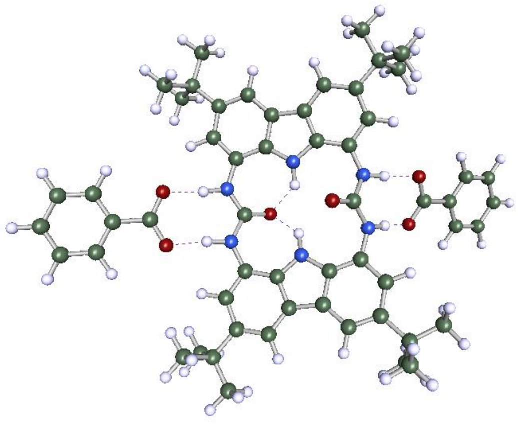

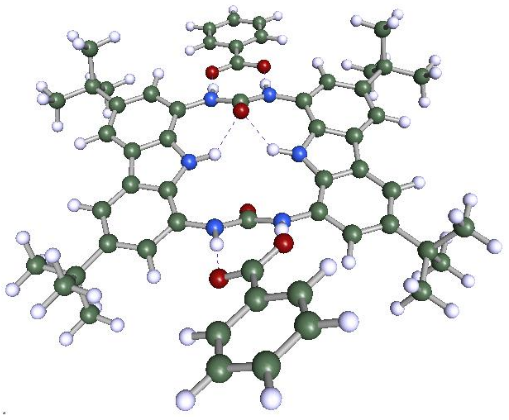

G = -1852203.69392 kcal/mol

Lowest energy conformation of receptor **MC001** with lactate anion

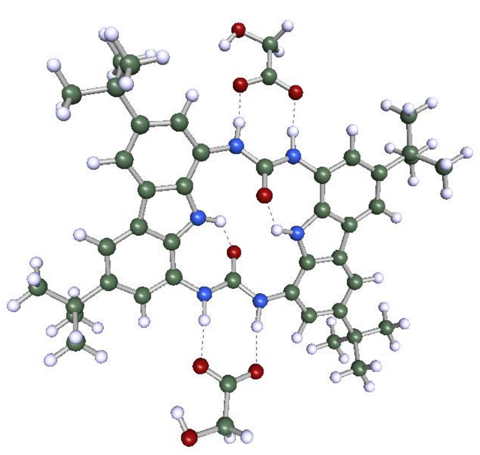

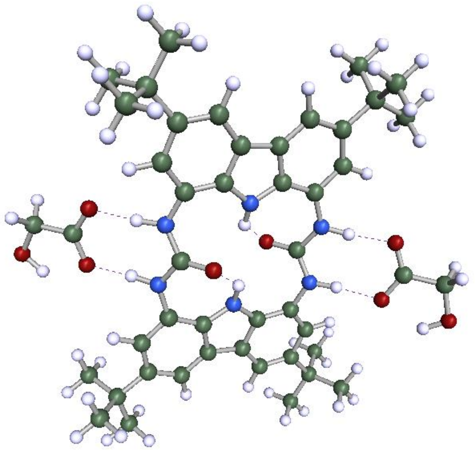

G = -1705929.89393 kcal/mol

Lowest energy conformation of receptor **MC001** with formate anion

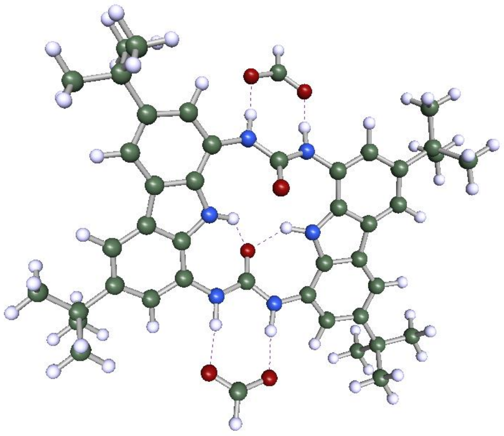

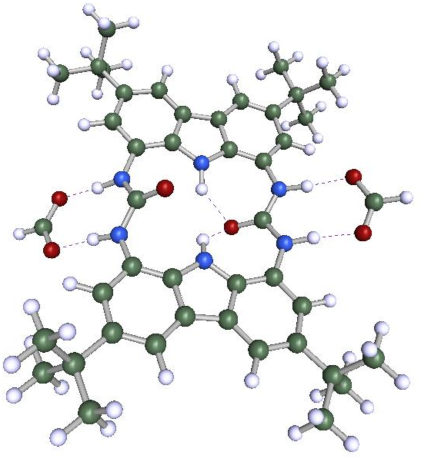

G = -1562111.52768 kcal/mol

Lowest energy conformation of receptor **MC002**

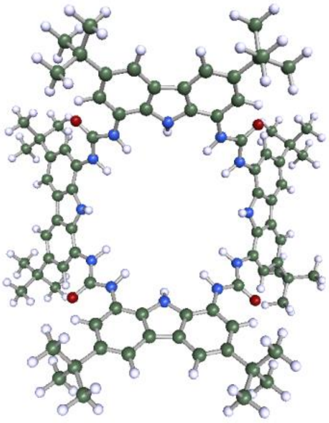

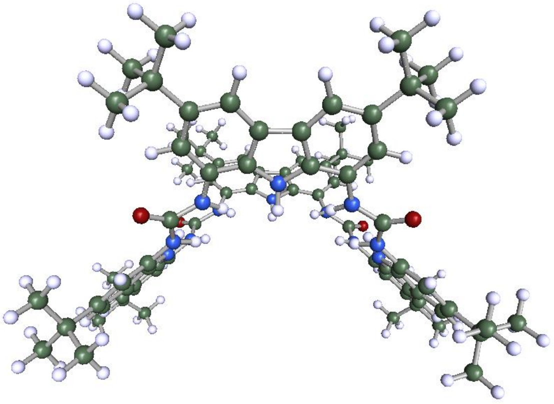

G = -2648766.27994 kcal/mol

Lowest energy conformation of receptor **MC002** with pivalate anion

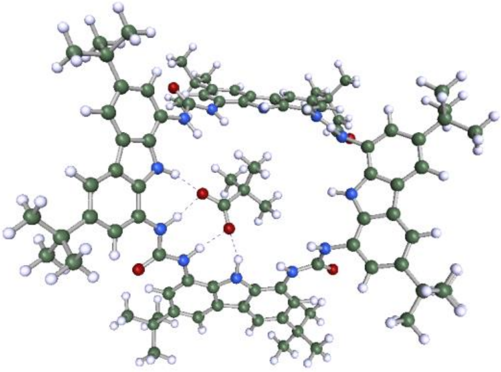

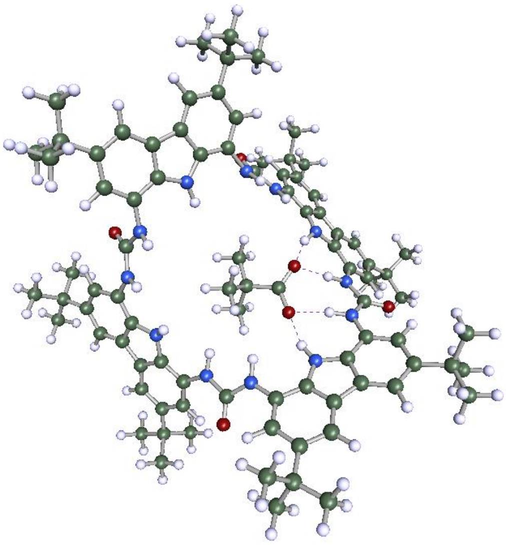

G = -2866337.50211 kcal/mol

Lowest energy conformation of receptor **MC002** with acetate anion

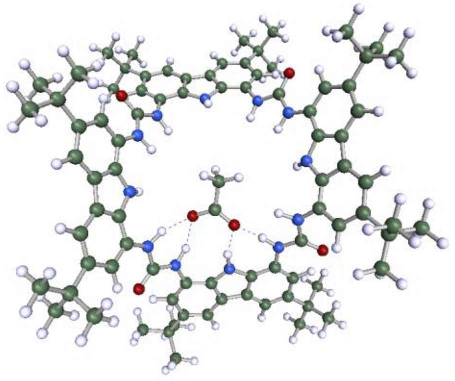

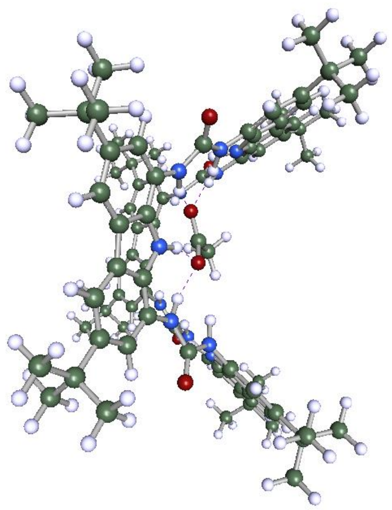

G = -2792310.16571 kcal/mol

Lowest energy conformation of receptor **MC002** with benzoate anion

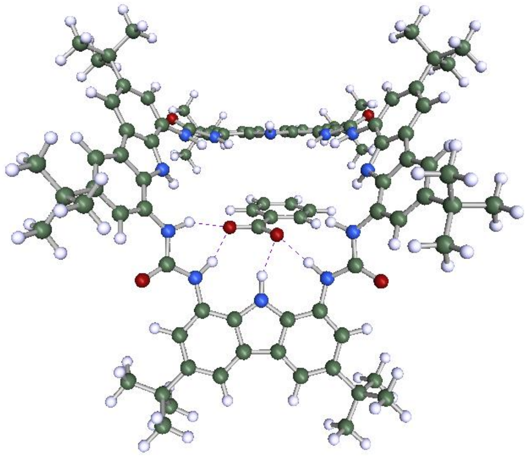

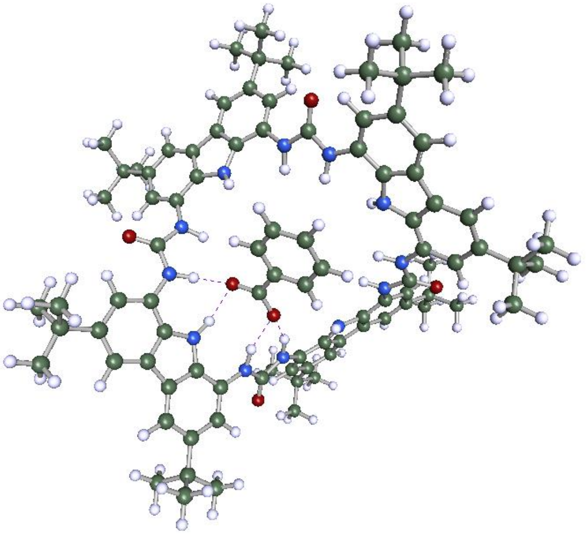

G = -2912671.14577 kcal/mol

Lowest energy conformation of receptor **MC002** with lactate anion

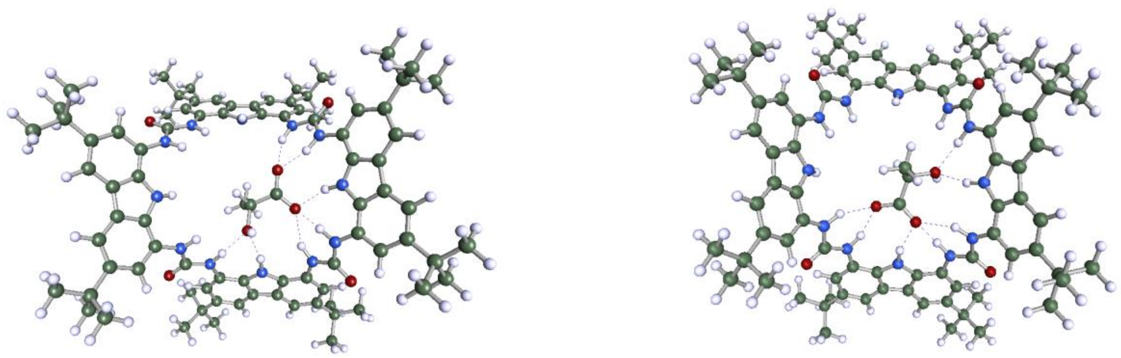

G = -2864220.05839 kcal/mol

Lowest energy conformation of receptor **MC002** with formate anion

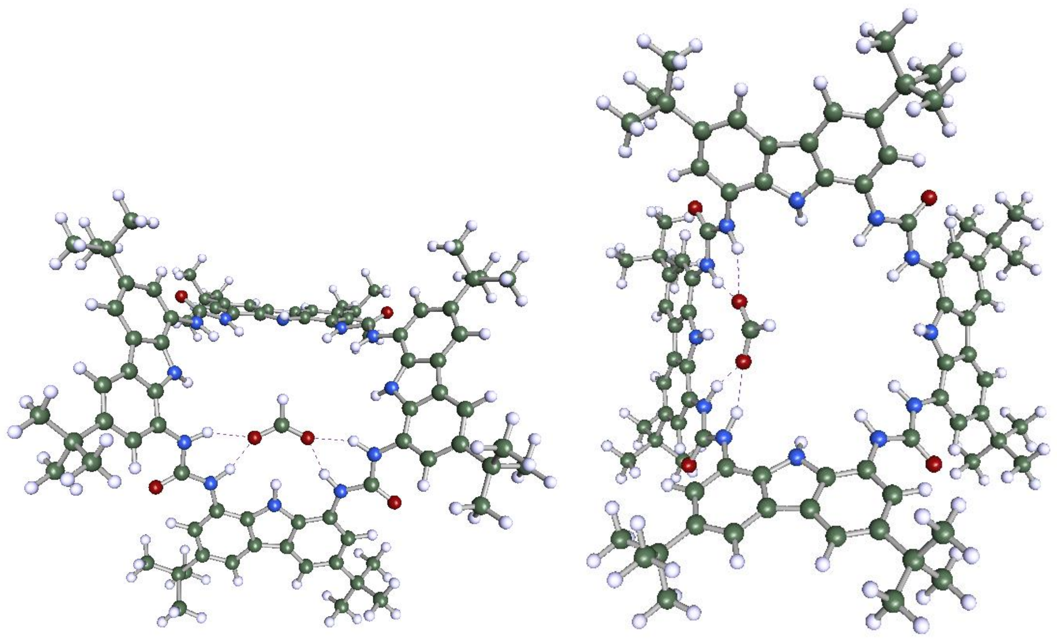

G = -2767634.54569 kcal/mol

Lowest energy conformation of receptor **MC003**

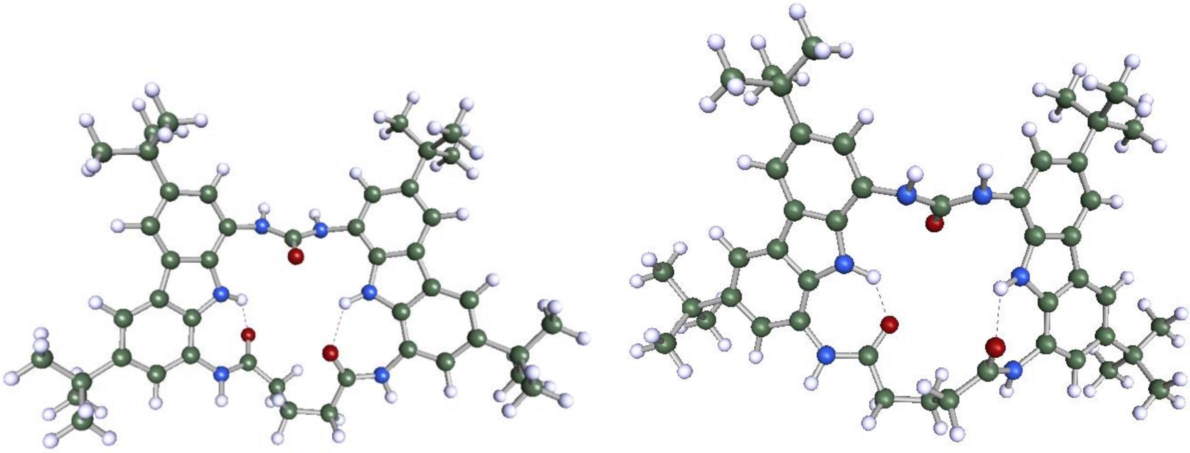

G = -1469566.91734 kcal/mol

Lowest energy conformation of receptor **MC003** with pivalate anion

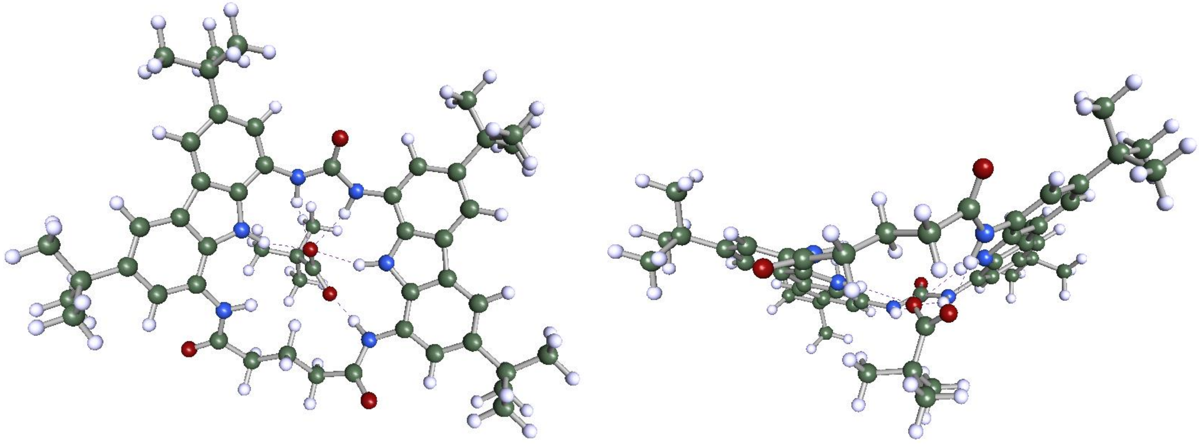

G = -1687151.33703 kcal/mol

Lowest energy conformation of receptor **MC003** with acetate anion

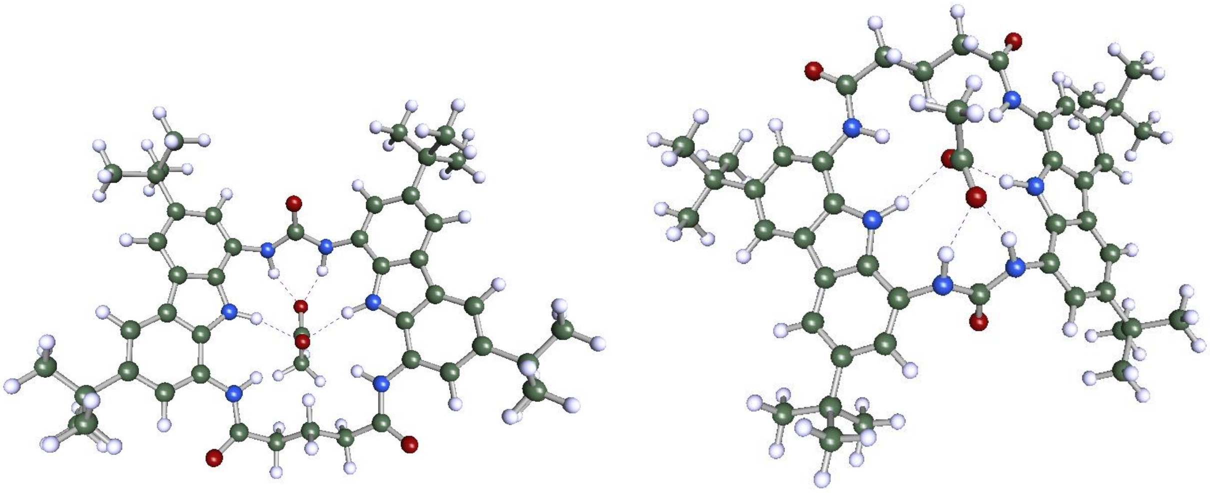

G = -1613118.82814 kcal/mol

Lowest energy conformation of receptor **MC003** with benzoate anion

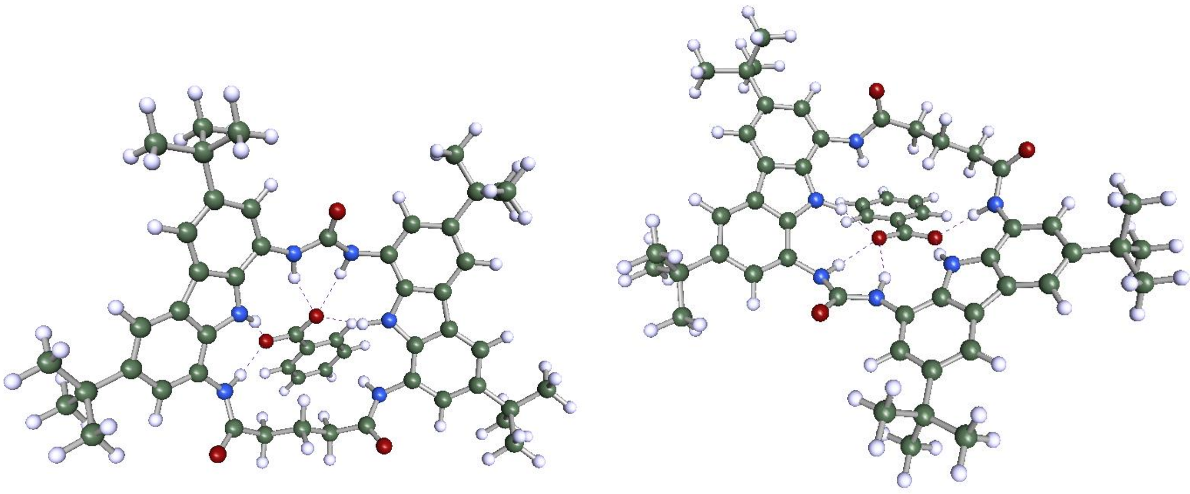

G = -1733483.80492 kcal/mol

Lowest energy conformation of receptor **MC003** with lactate anion

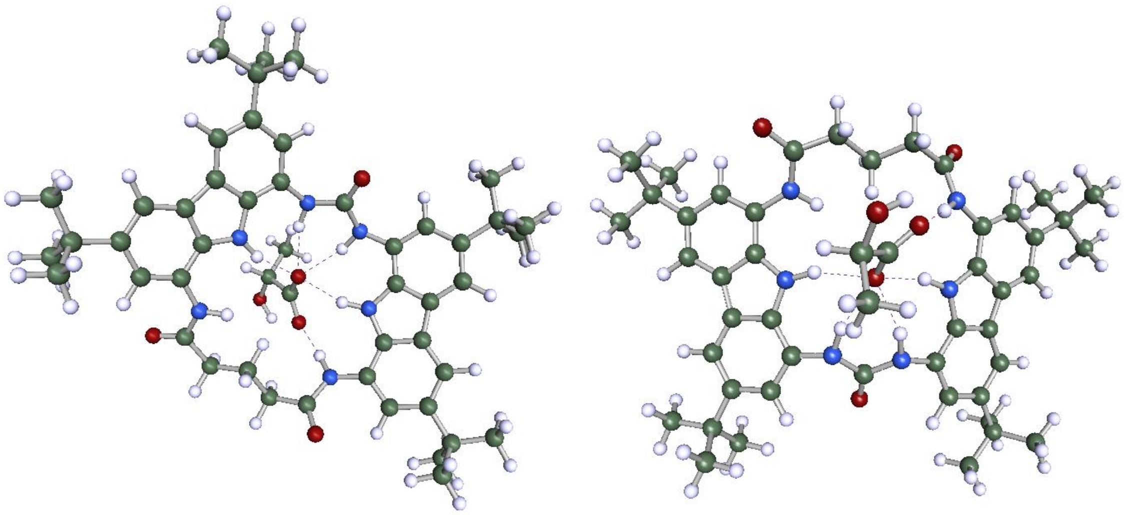

G = -1685024.93247 kcal/mol

Lowest energy conformation of receptor **MC003** with formate anion

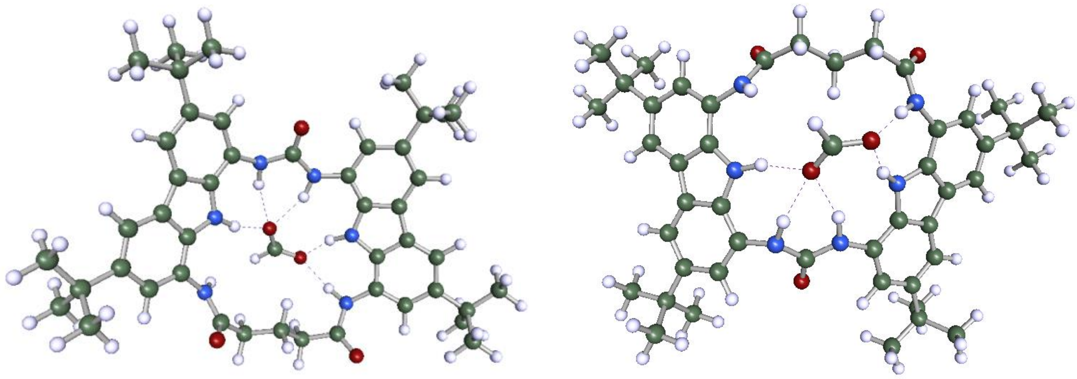

G = -1588438.70245 kcal/mol

Lowest energy conformation of receptor **MC004**

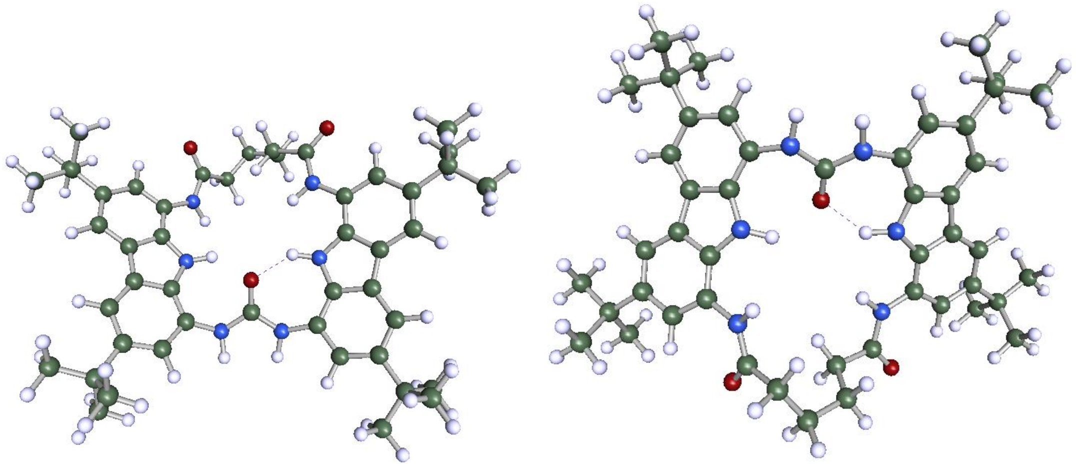

G = -1494248.55193 kcal/mol

Lowest energy conformation of receptor **MC004** with pivalate anion

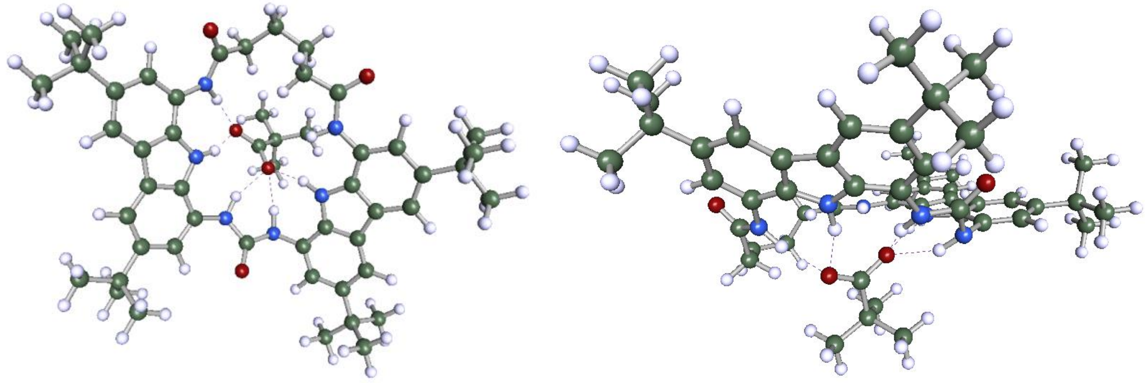

G = -1711829.70872 kcal/mol

Lowest energy conformation of receptor **MC004** with acetate anion

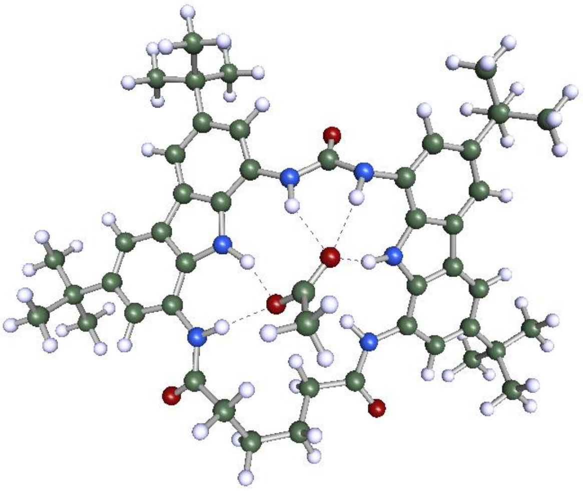 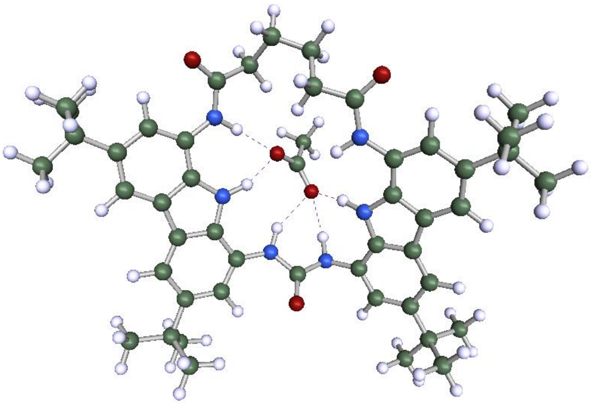

G = -1637796.77666 kcal/mol

Lowest energy conformation of receptor **MC004** with benzoate anion

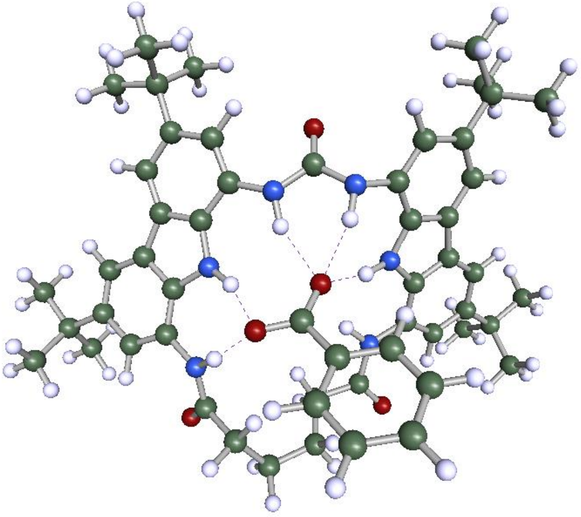 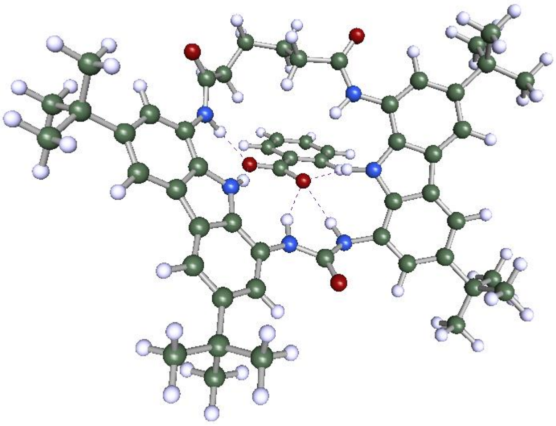

G = -1758160.92459 kcal/mol

Lowest energy conformation of receptor **MC004** with lactate anion

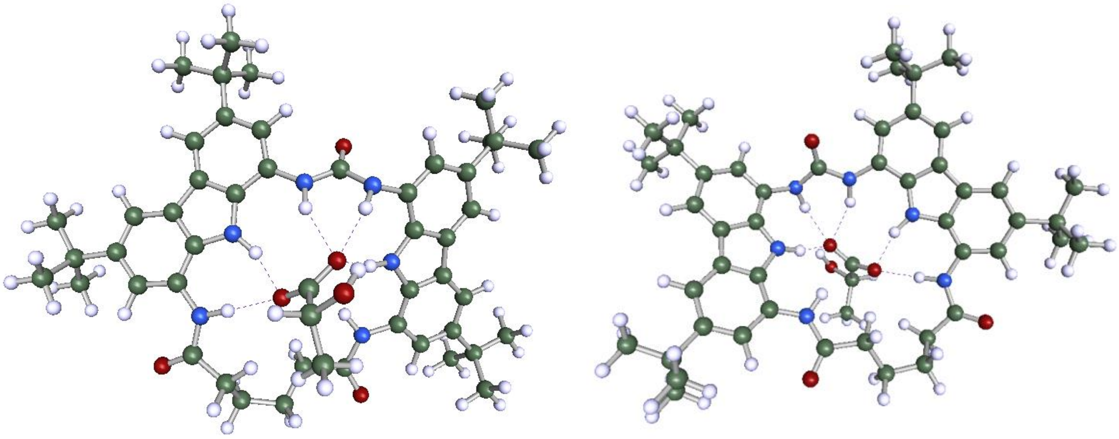

G = -1709700.31149 kcal/mol

Lowest energy conformation of receptor **MC004** with formate anion

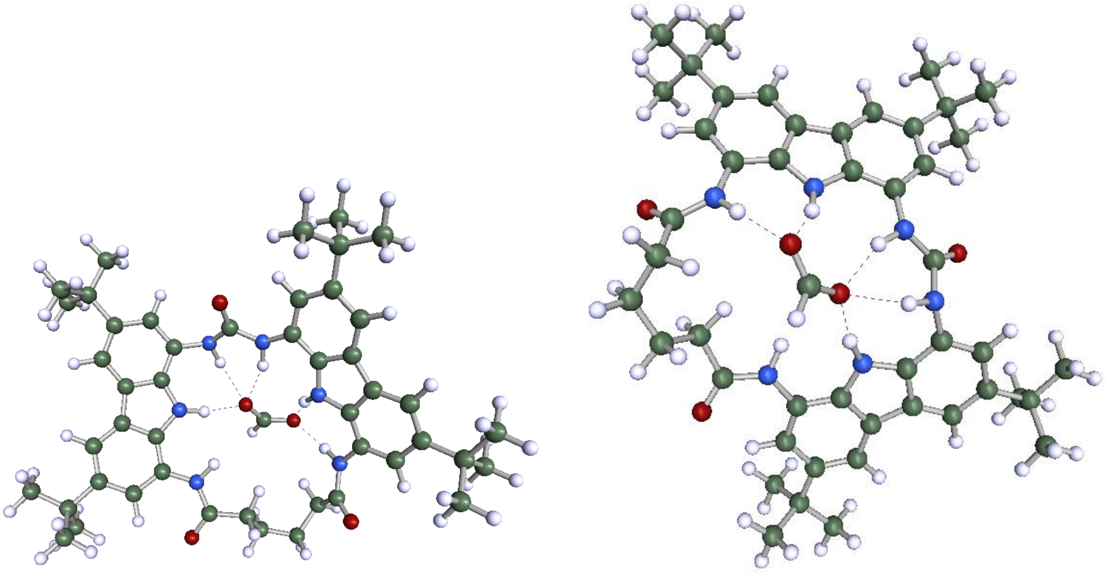

G = -1613115.44806 kcal/mol

Lowest energy conformation of receptor **MC005**

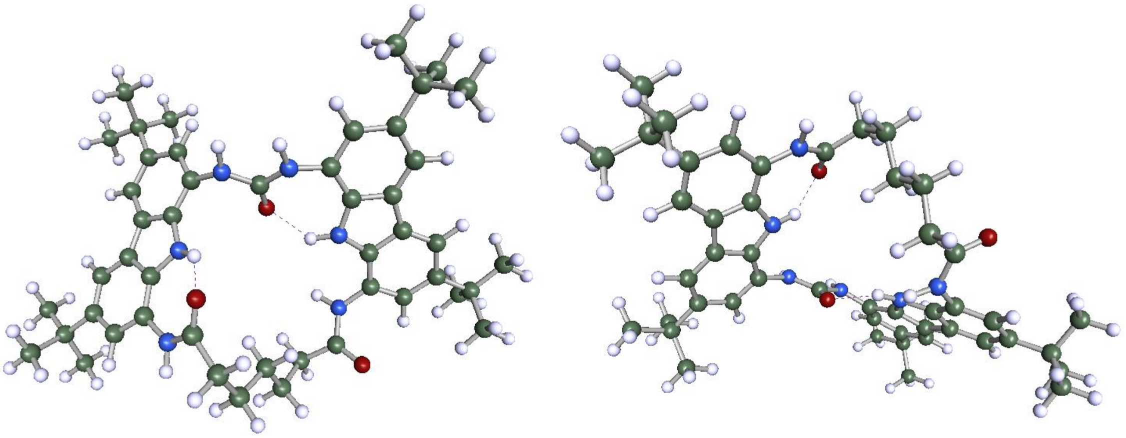

G = -1518926.98220 kcal/mol

Lowest energy conformation of receptor **MC005** with pivalate anion

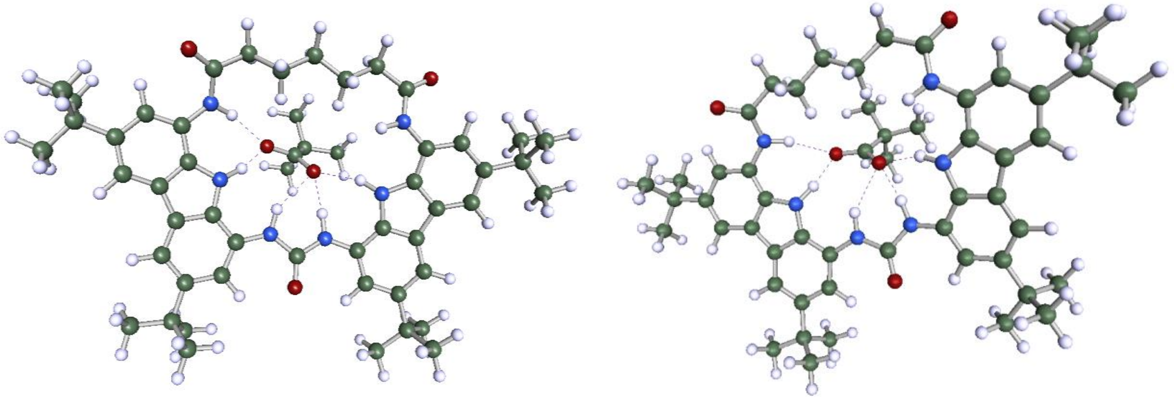

G = -1736508.43110 kcal/mol

Lowest energy conformation of receptor **MC005** with acetate anion

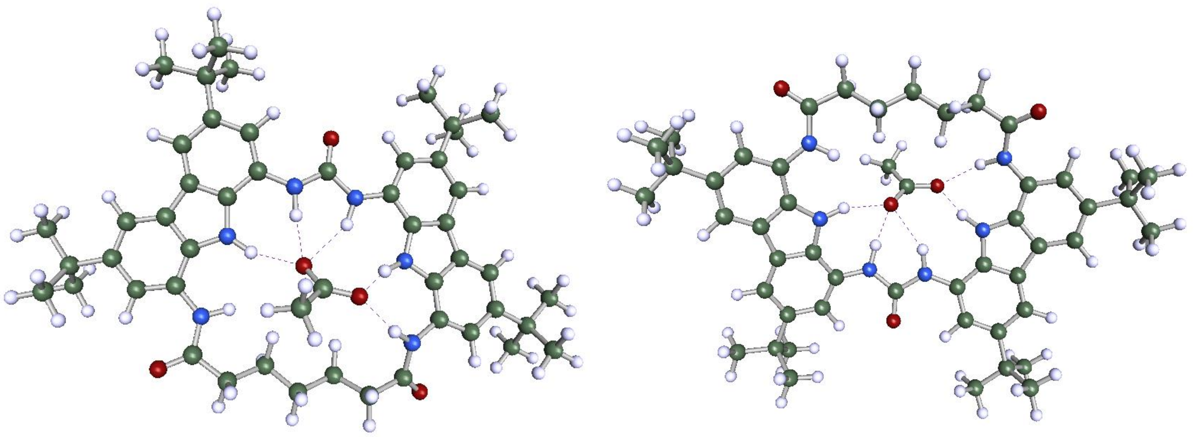

G = -1662477.72110 kcal/mol

Lowest energy conformation of receptor **MC005** with benzoate anion

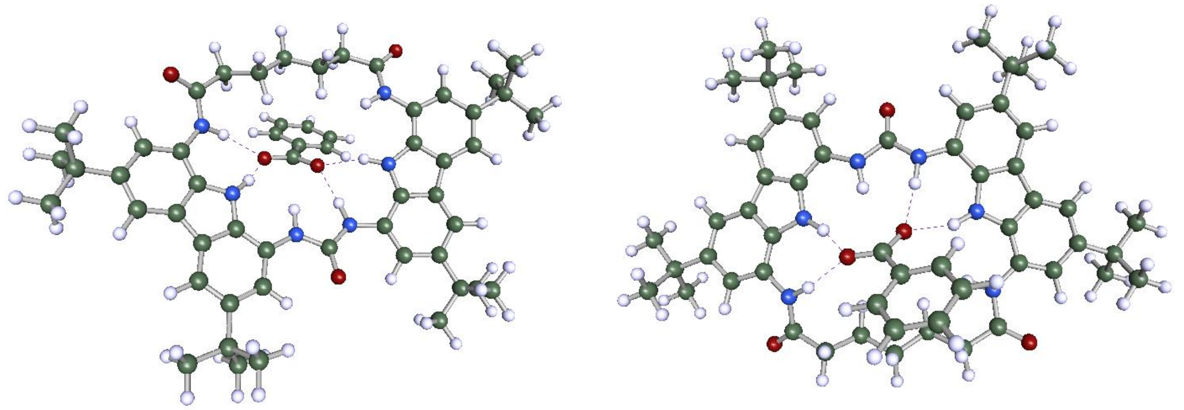

G = -1782840.25461 kcal/mol

Lowest energy conformation of receptor **MC005** with lactate anion

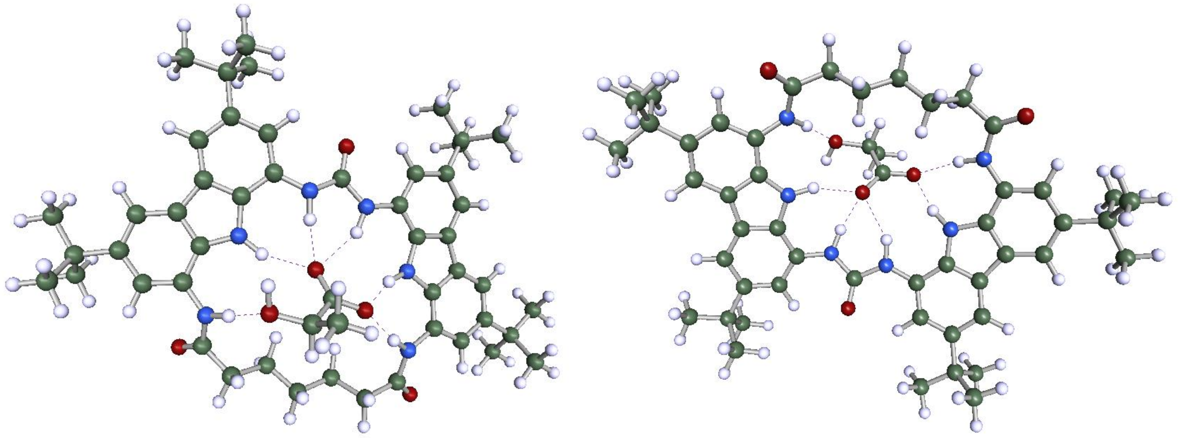

G = -1734386.24267 kcal/mol

Lowest energy conformation of receptor **MC005** with formate anion

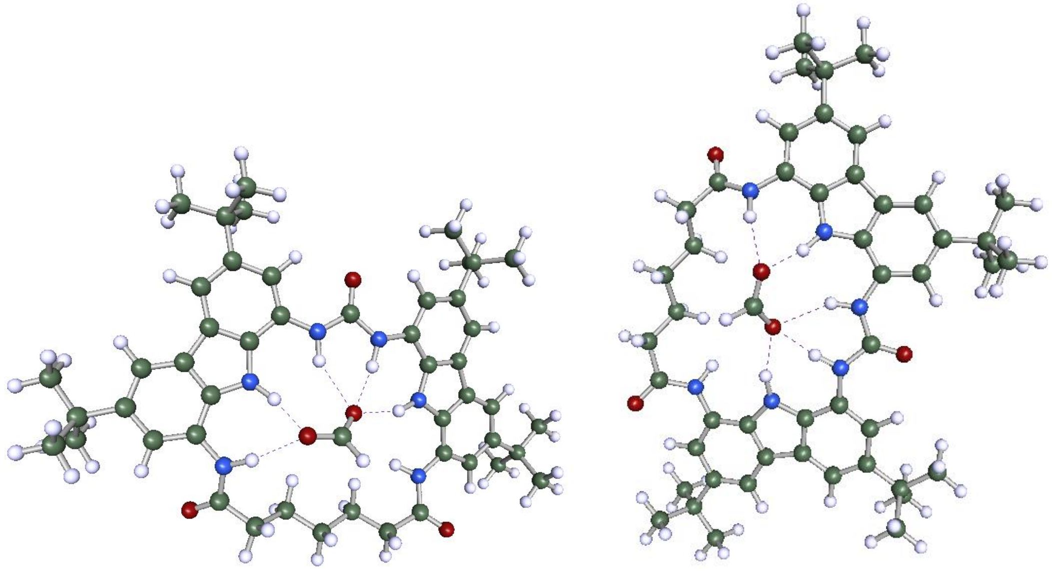

G = -1637795.20174 kcal/mol

Lowest energy conformation of receptor **MC006**

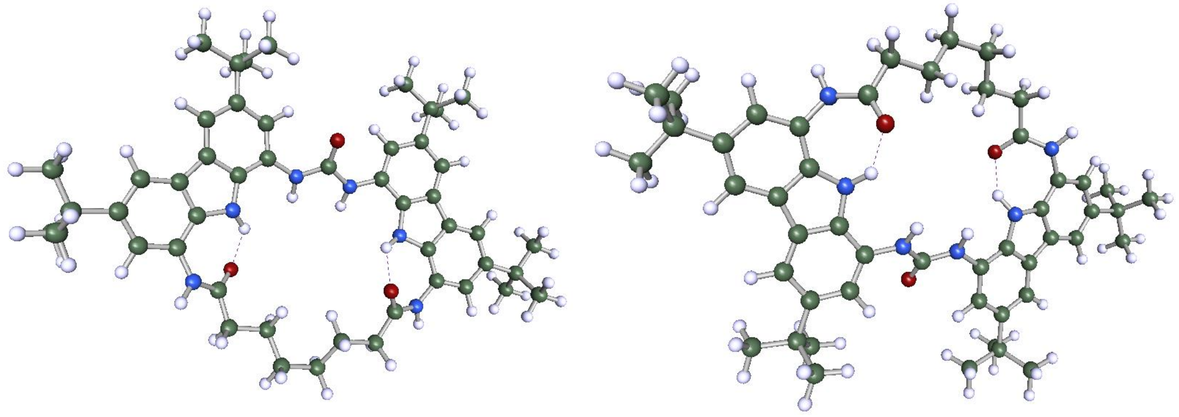

G = -1543604.89540 kcal/mol

Lowest energy conformation of receptor **MC006** with pivalate anion

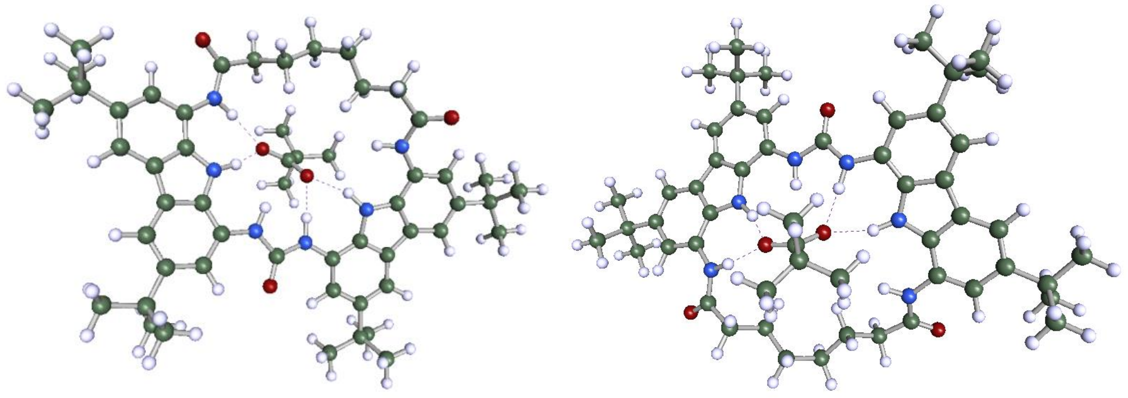

G = -1761185.90669 kcal/mol

Lowest energy conformation of receptor **MC006** with acetate anion

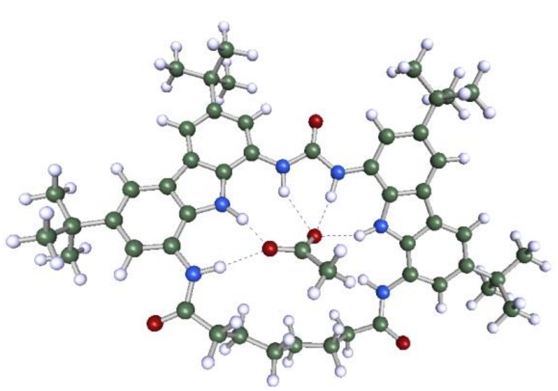
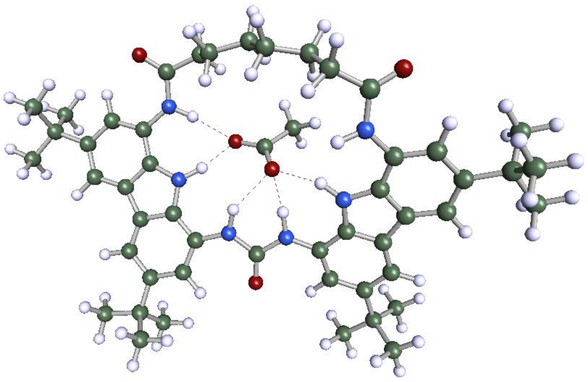

G = -1687156.27049 kcal/mol

Lowest energy conformation of receptor **MC006** with benzoate anion

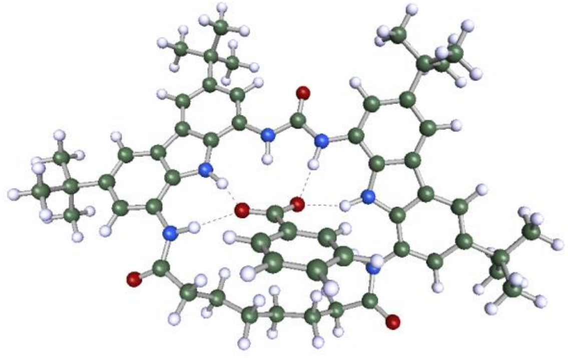
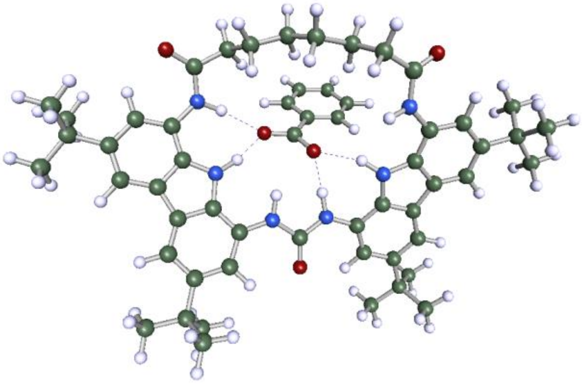

G = -1807517.68972 kcal/mol

Lowest energy conformation of receptor **MC006** with lactate anion

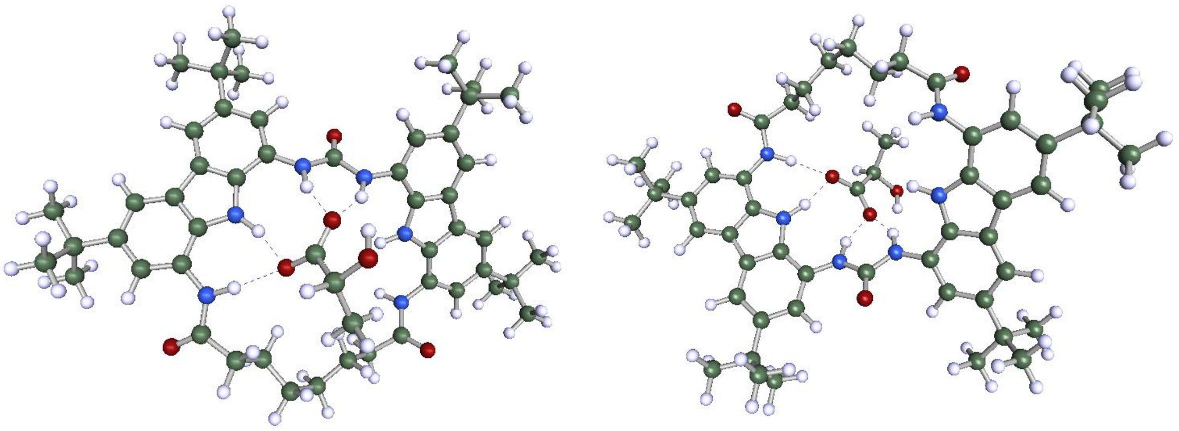

G = -1759060.05069 kcal/mol

Lowest energy conformation of receptor **MC006** with formate anion

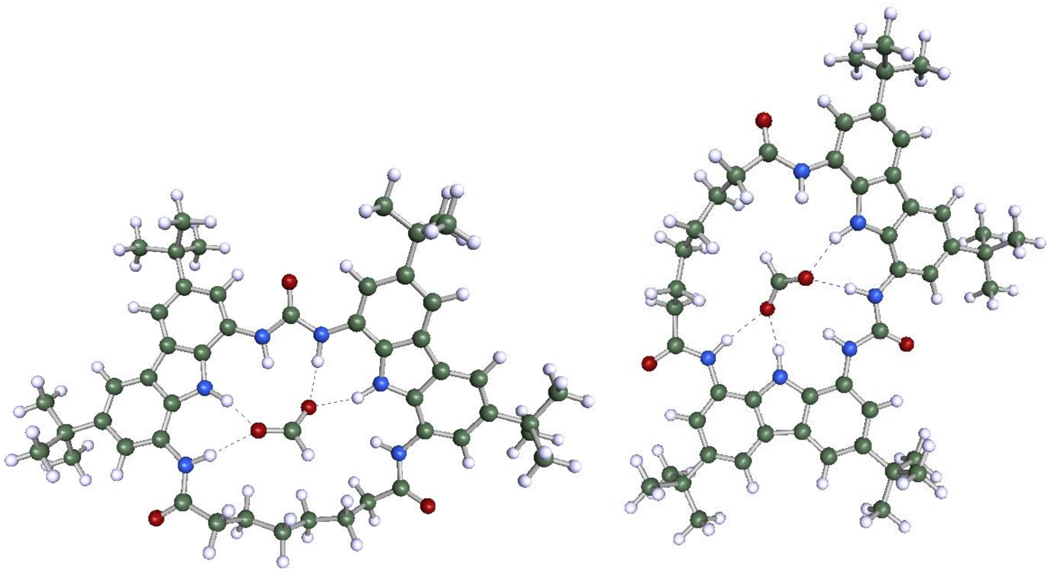

G = -1662474.70525 kcal/mol

Lowest energy conformation of receptor **MC007**

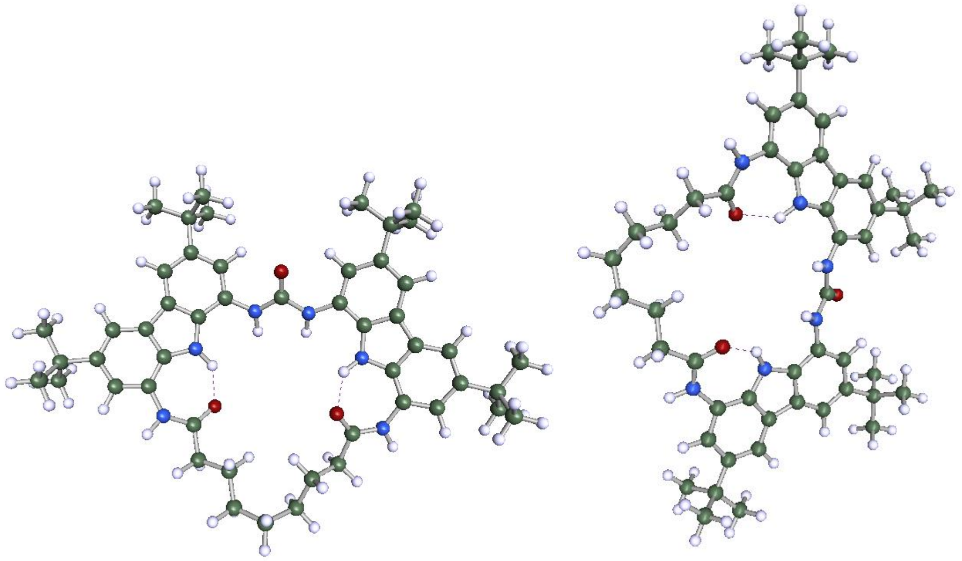

G = -1568283.77596 kcal/mol

Lowest energy conformation of receptor **MC007** with pivalate anion

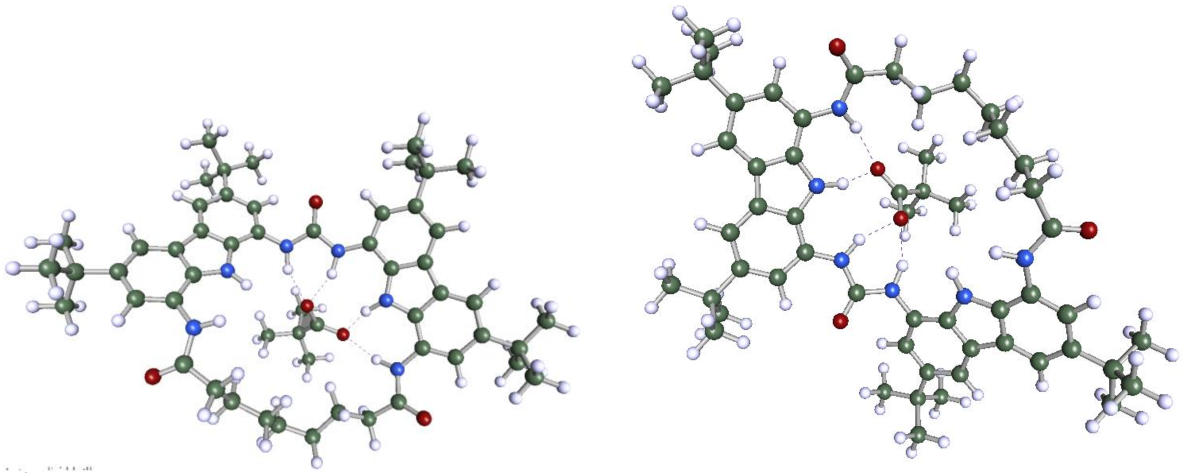

G = -1785862.89606 kcal/mol

Lowest energy conformation of receptor **MC007** with acetate anion

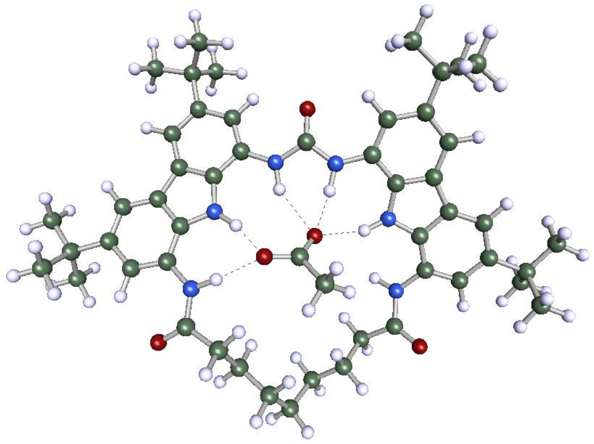

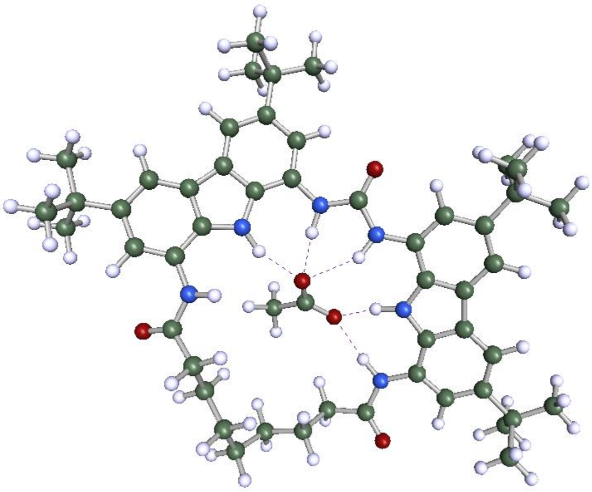

G = -1711837.51767 kcal/mol

Lowest energy conformation of receptor **MC007** with benzoate anion

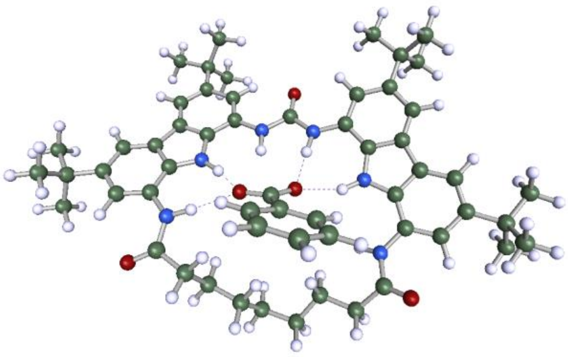

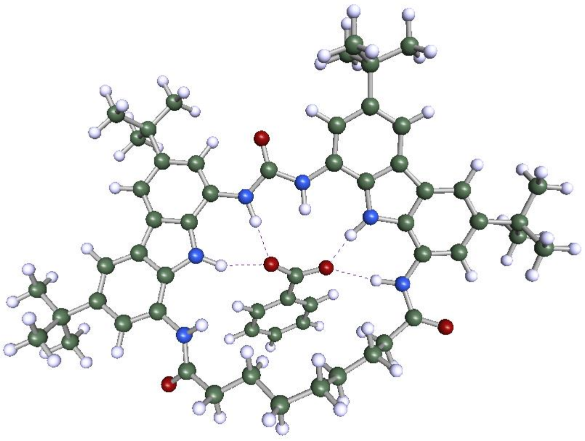

G = -1832195.75582 kcal/mol

Lowest energy conformation of receptor **MC007** with lactate anion

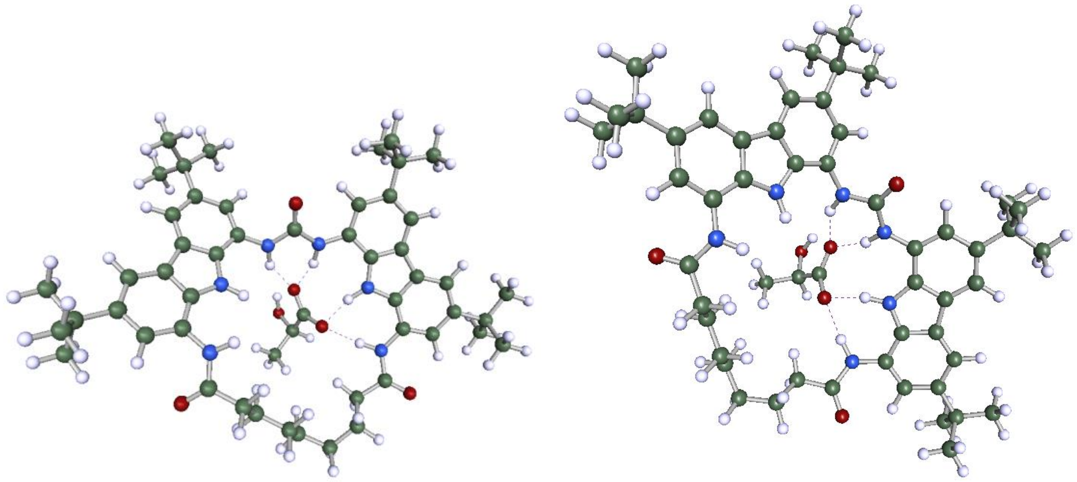

G = -1783739.48423 kcal/mol

Lowest energy conformation of receptor **MC007** with formate anion

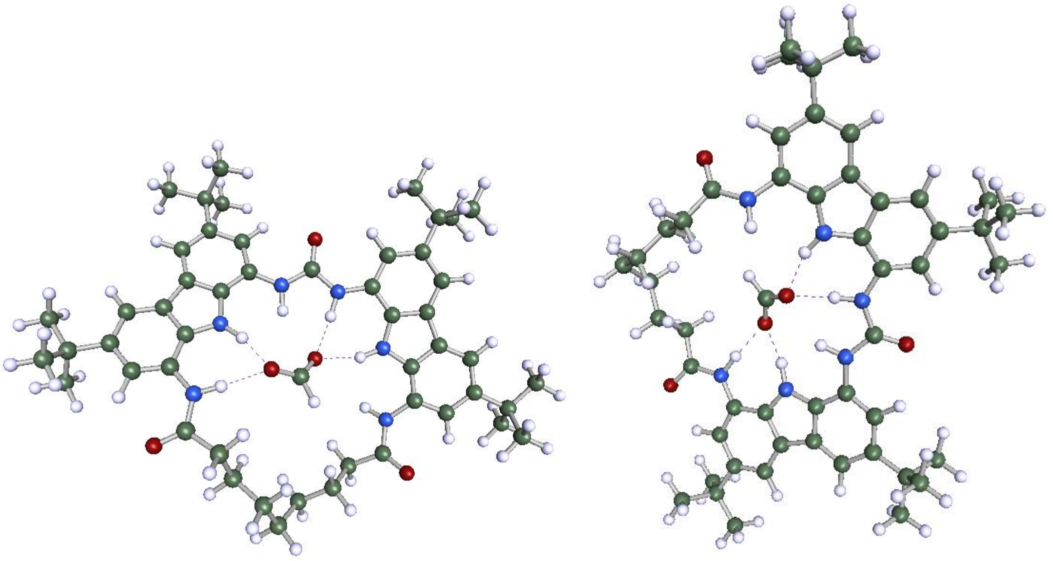

G = -1687155.70120 kcal/mol

Lowest energy conformation of receptor **MC008**

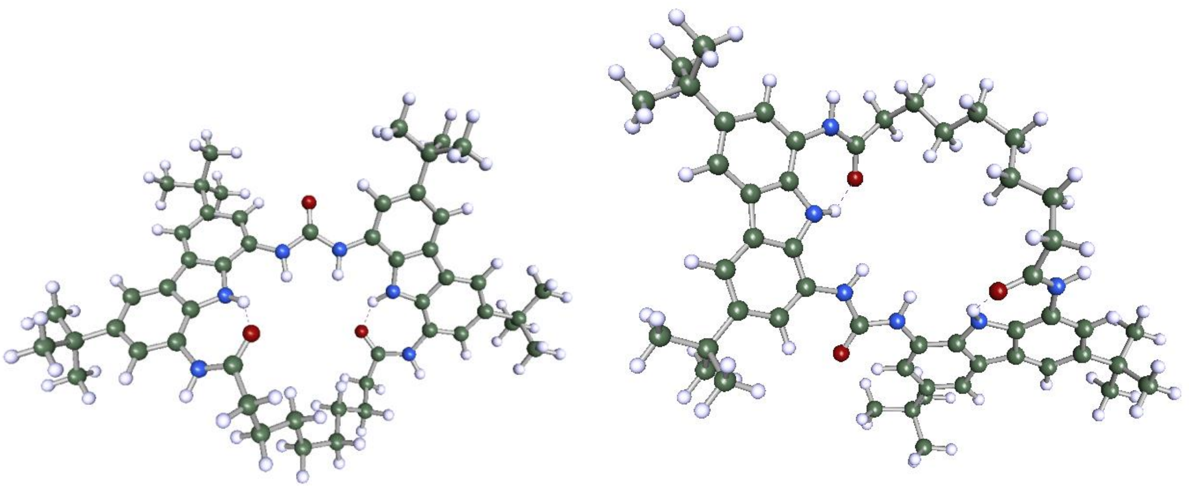

G = -1592960.38942 kcal/mol

Lowest energy conformation of receptor **MC008** with pivalate anion

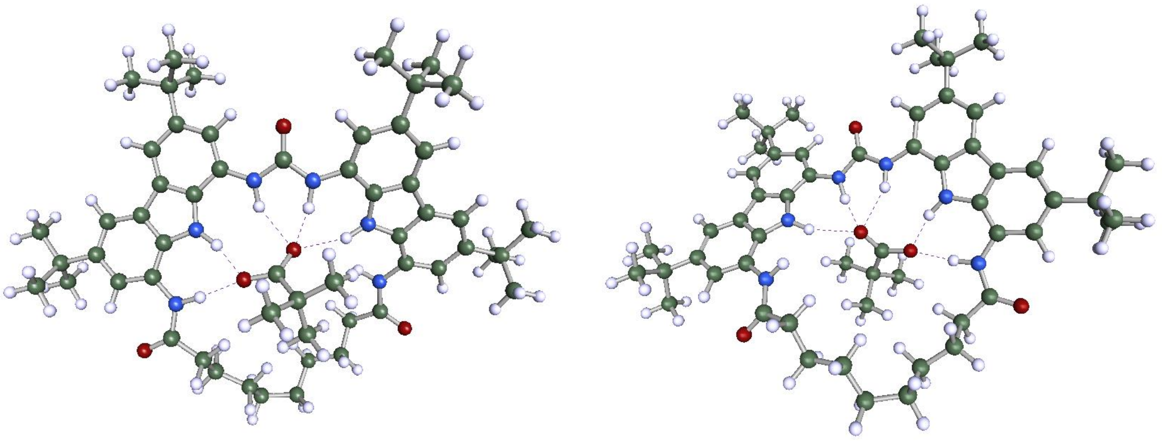

G = -1810542.30752 kcal/mol

Lowest energy conformation of receptor **MC008** with acetate anion

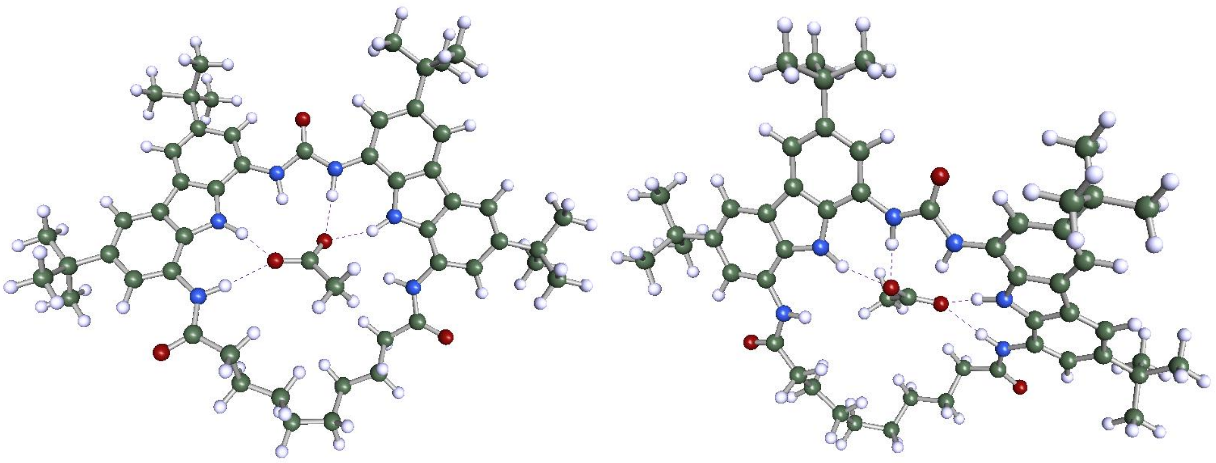

G = -1736513.26323 kcal/mol

Lowest energy conformation of receptor **MC008** with benzoate anion

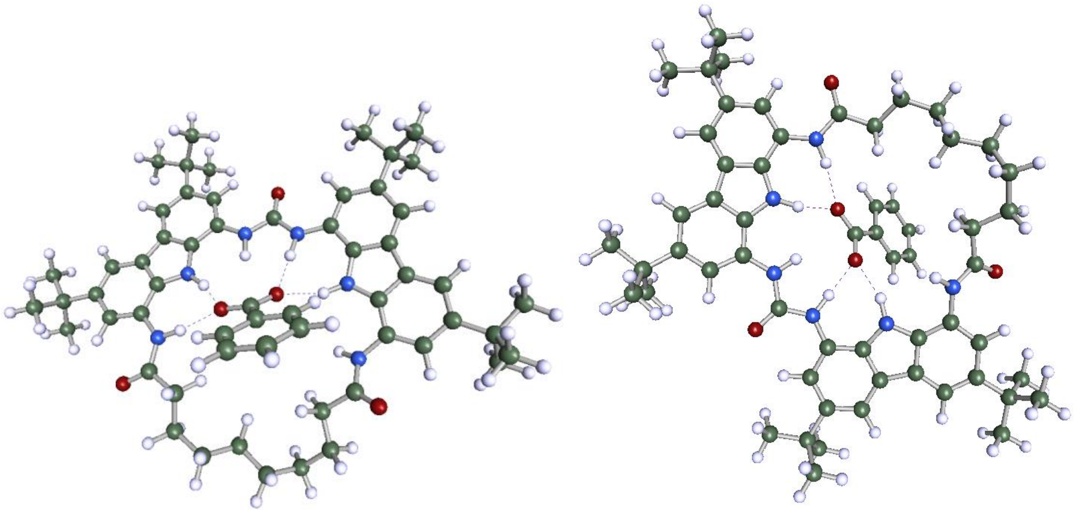

G = -1856873.88068 kcal/mol

Lowest energy conformation of receptor **MC008** with lactate anion

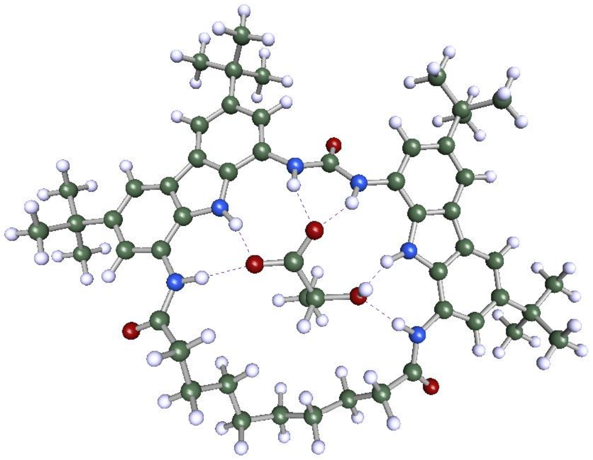

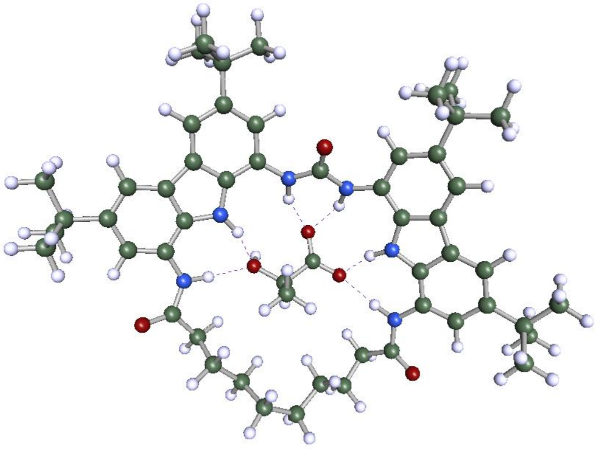

G = -1808417.42913 kcal/mol

Lowest energy conformation of receptor **MC008** with formate anion

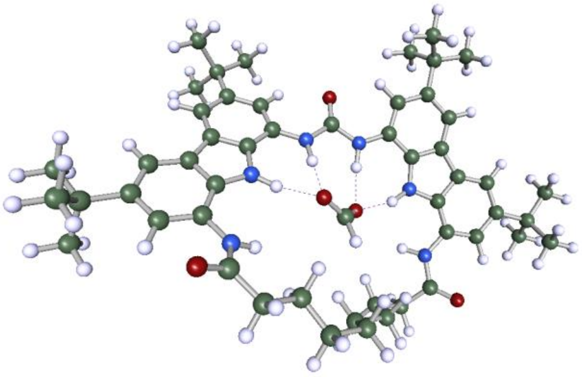

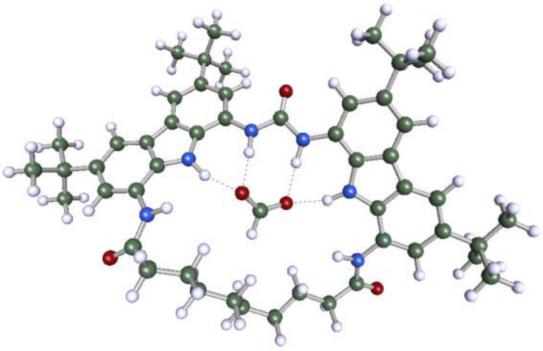

G = -1711828.05606 kcal/mol

Lowest energy conformation of receptor **MC009**

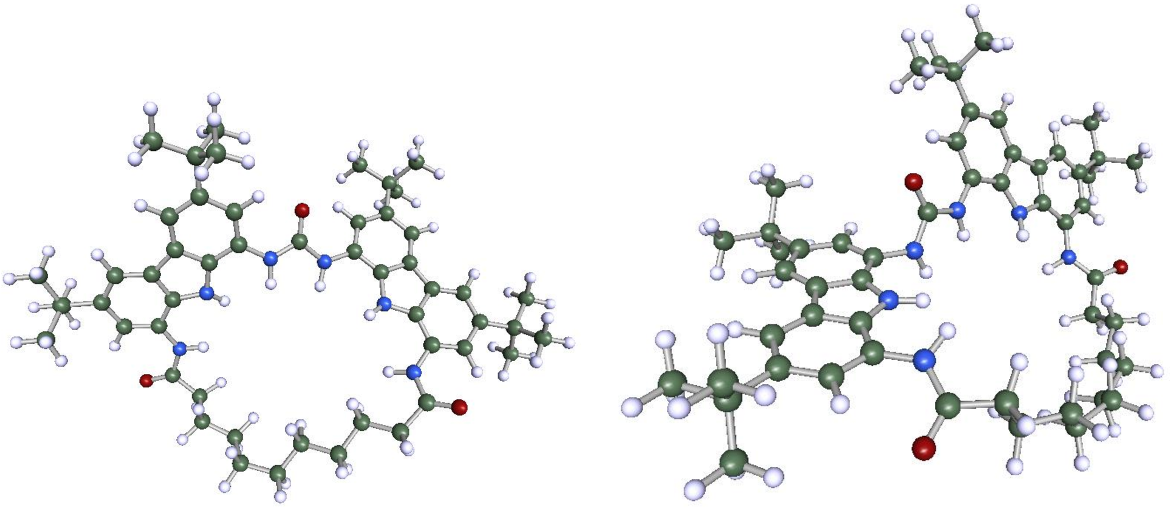

G = -1617636.36547 kcal/mol

Lowest energy conformation of receptor **MC009** with pivalate anion

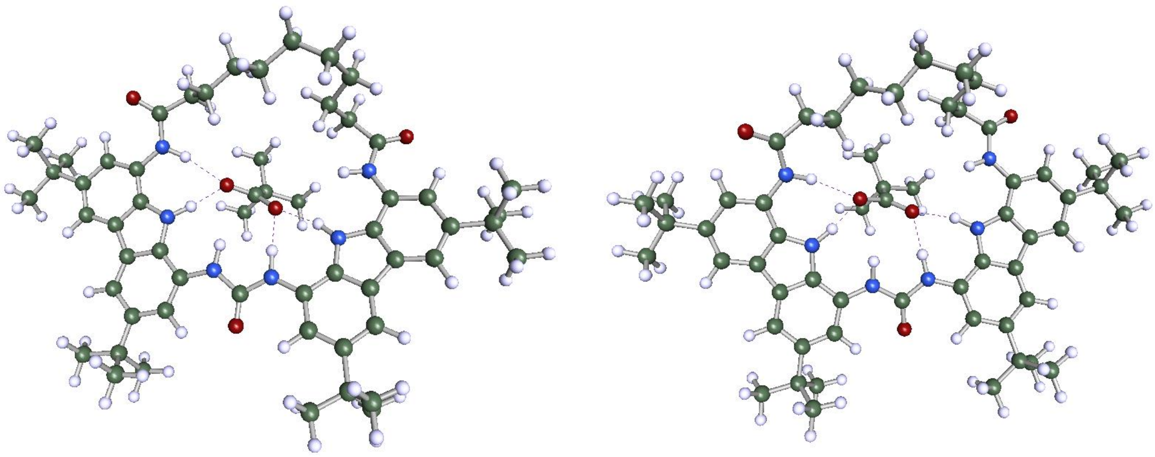

G = -1835214.67834 kcal/mol

Lowest energy conformation of receptor **MC009** with acetate anion

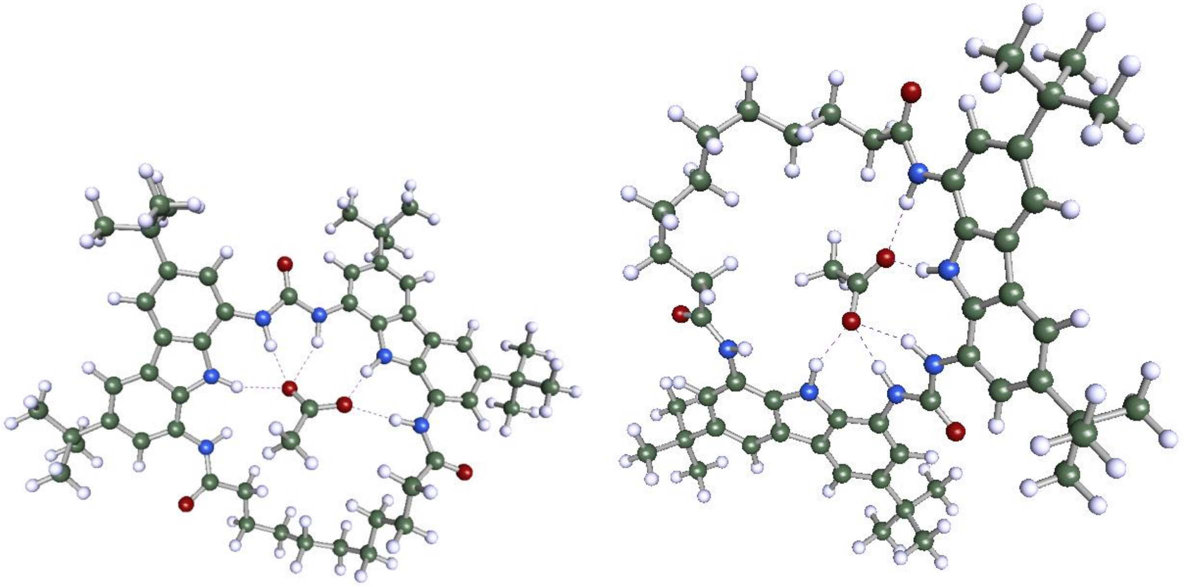

G = -1761192.93428 kcal/mol

Lowest energy conformation of receptor **MC009** with benzoate anion

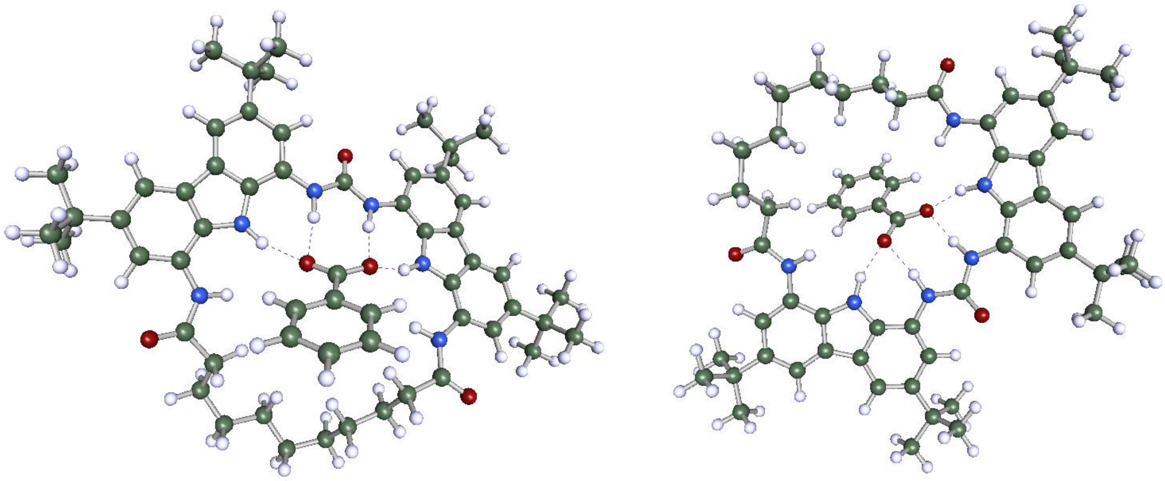

G = -1881553.39445 kcal/mol

Lowest energy conformation of receptor **MC009** with lactate anion

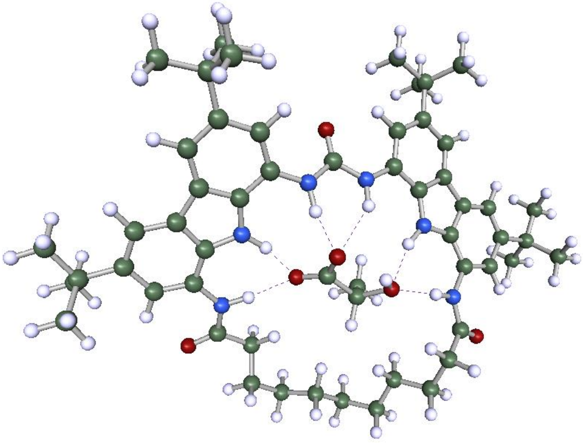

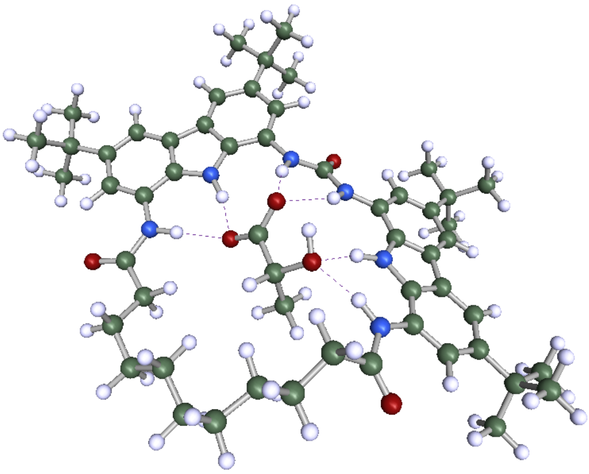

G = -1833097.81583 kcal/mol

Lowest energy conformation of receptor **MC009** with formate anion

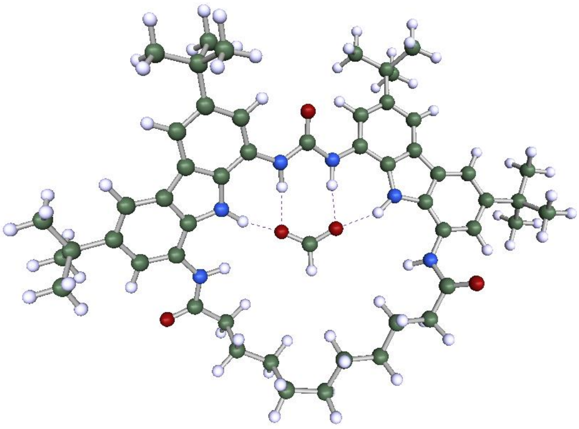

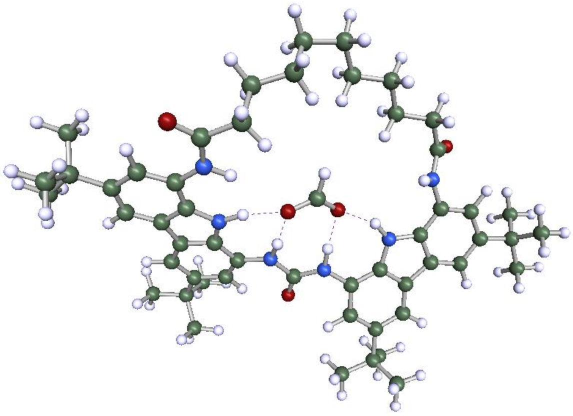

G = -1736506.44416 kcal/mol

Lowest energy conformation of receptor **MC010**

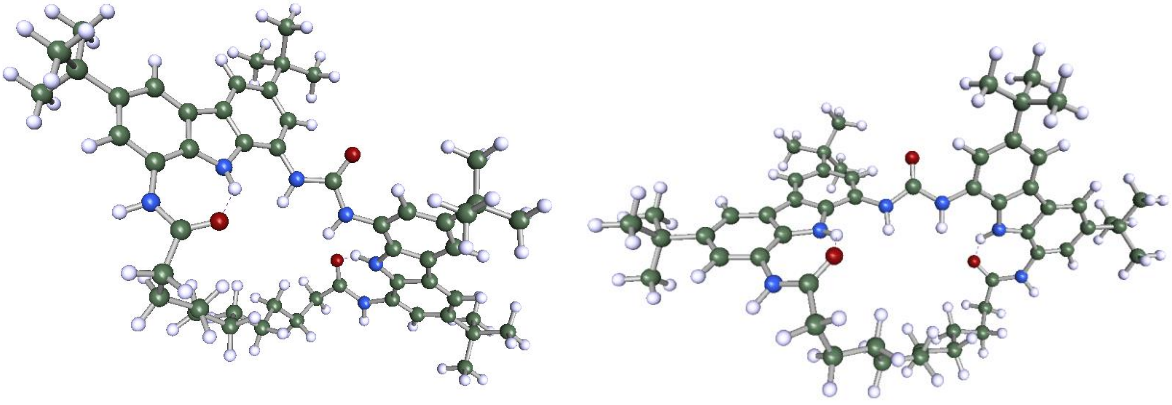

G = -1642313.24091 kcal/mol

Lowest energy conformation of receptor **MC010** with pivalate anion

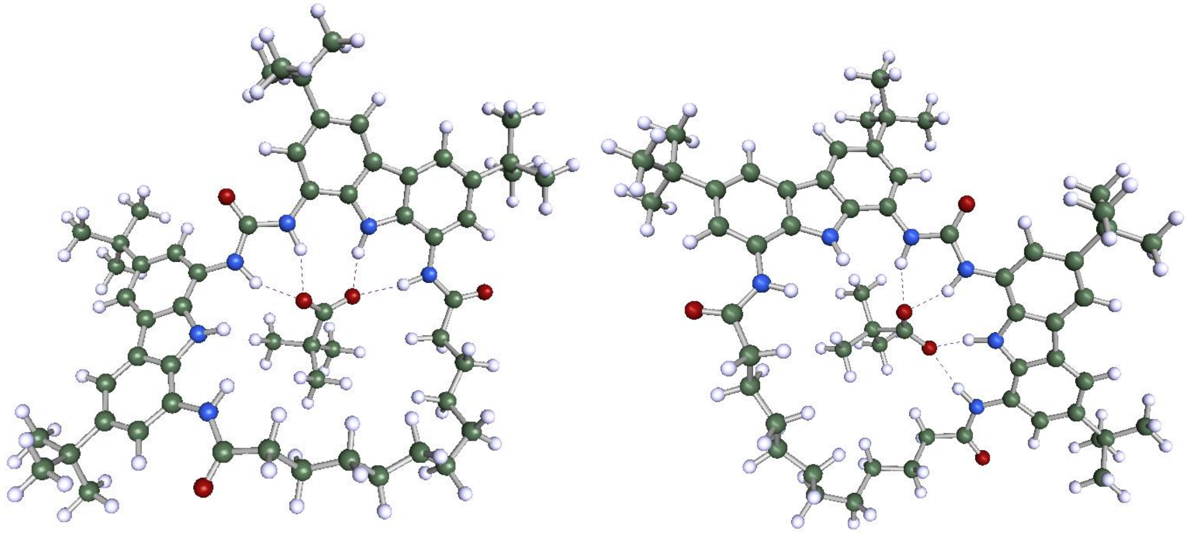

G = -1859899.36917 kcal/mol

Lowest energy conformation of receptor **MC010** with acetate anion

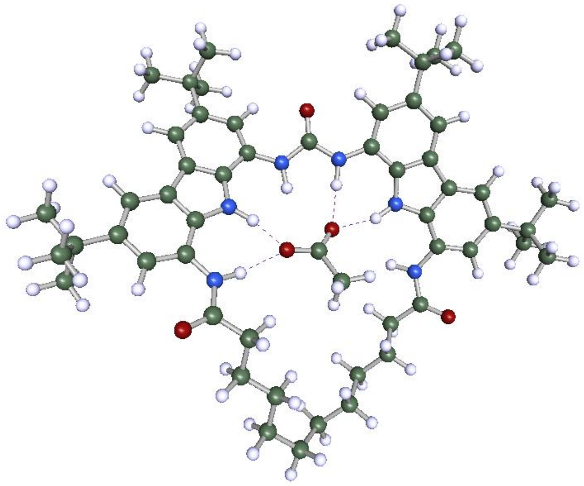

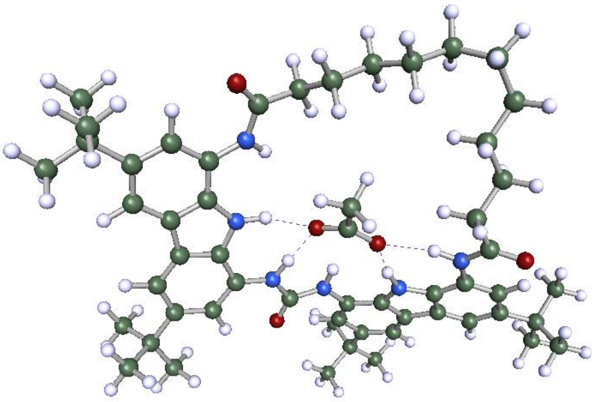

G = -1785869.40606 kcal/mol

Lowest energy conformation of receptor **MC010** with benzoate anion

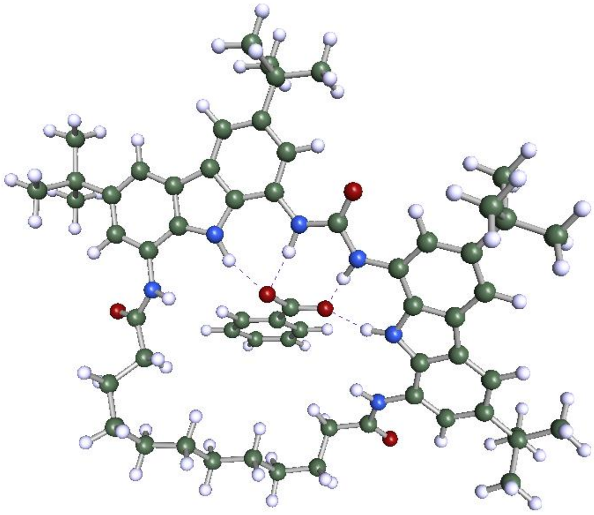

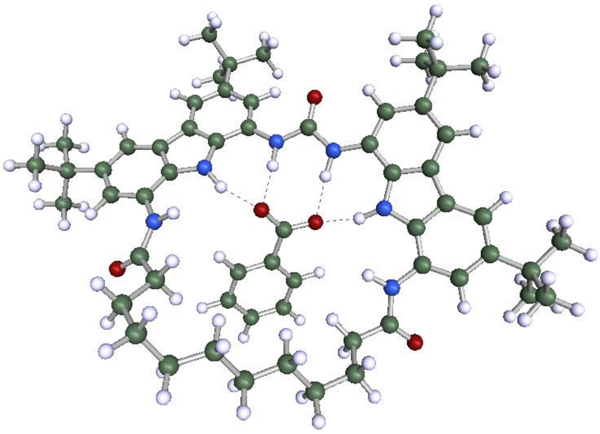

G = -1906229.24005 kcal/mol

Lowest energy conformation of receptor **MC010** with lactate anion

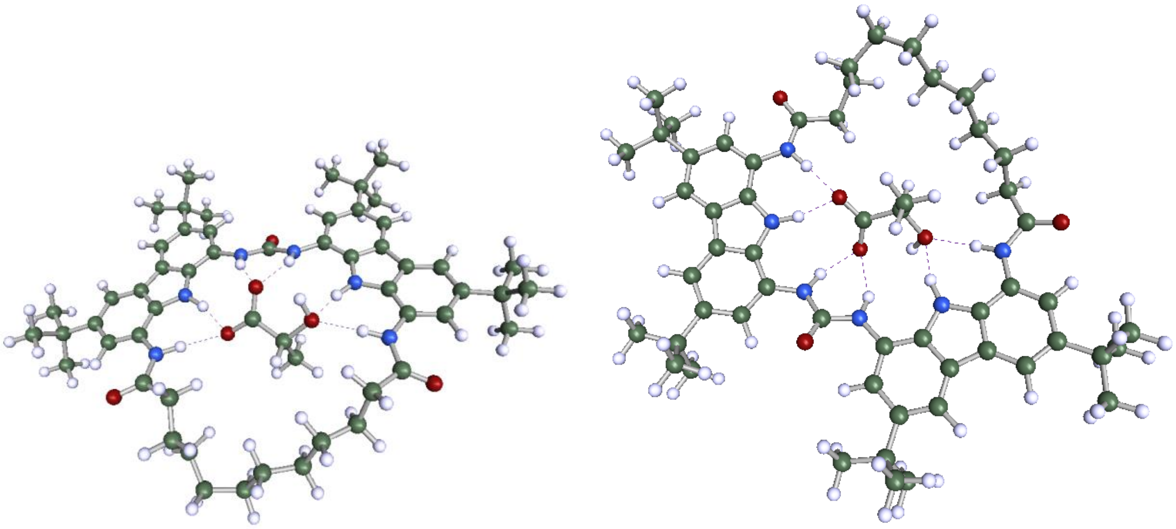

G = -1857774.74552 kcal/mol

Lowest energy conformation of receptor **MC010** with formate anion

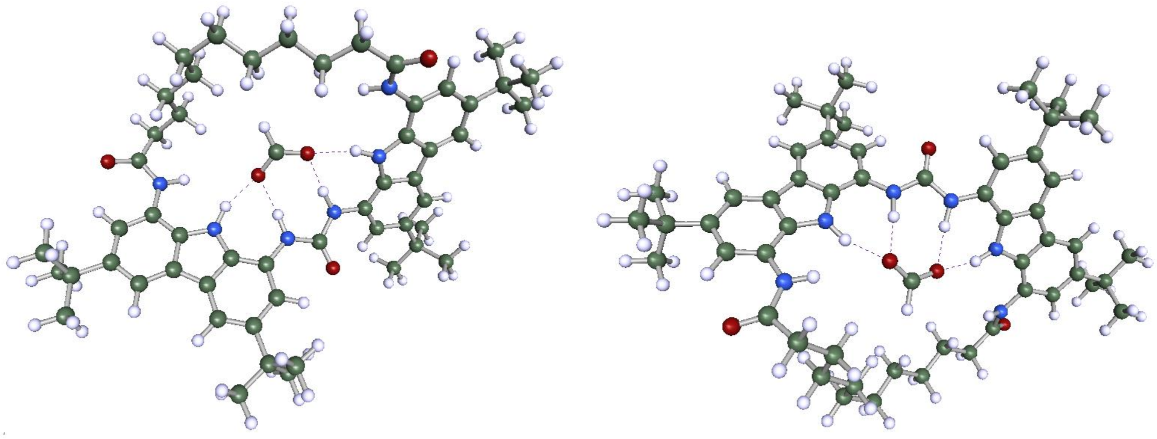

G = -1761183.57068 kcal/mol

Lowest energy conformation of receptor **MC011**

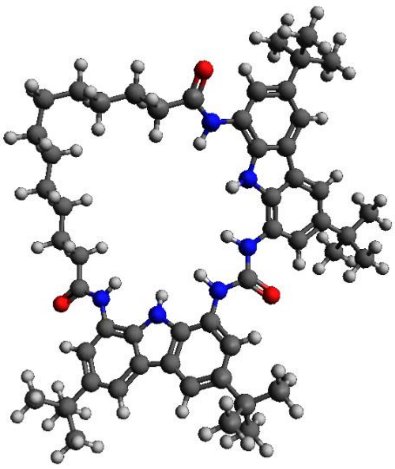

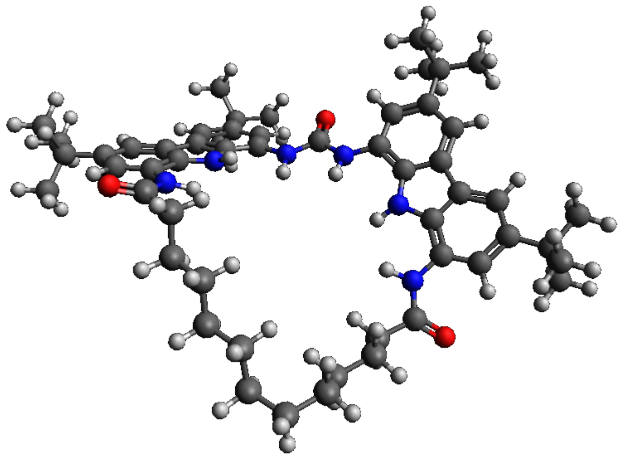

G = -1666995.75494 kcal/mol

Lowest energy conformation of receptor **MC011** with pivalate anion

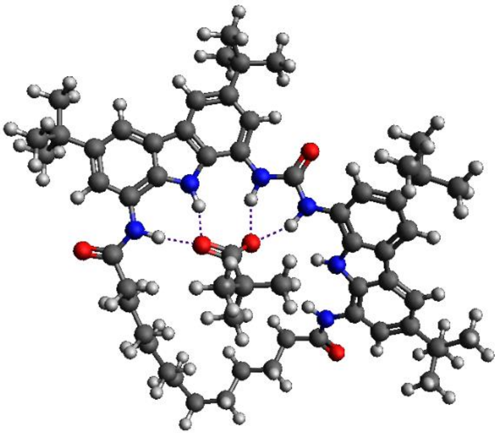

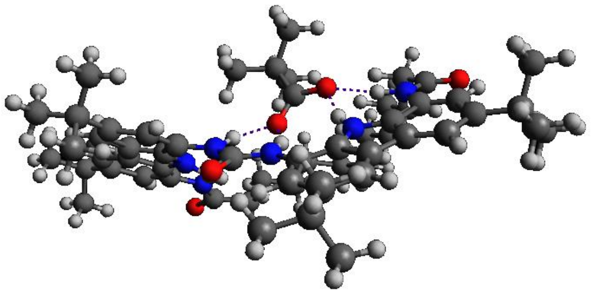

G = -11884578.42968 kcal/mol

Lowest energy conformation of receptor **MC011** with acetate anion

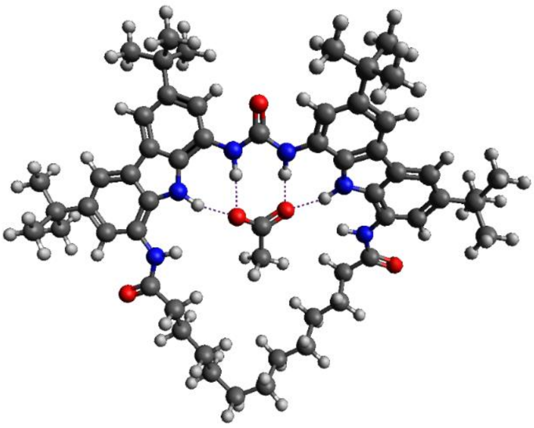

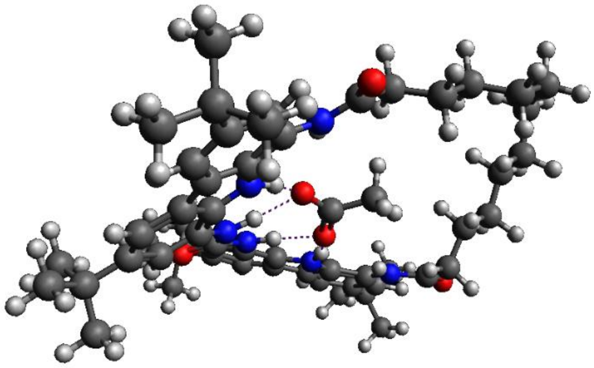

G = -1810547.72110 kcal/mol

Lowest energy conformation of receptor **MC011** with benzoate anion

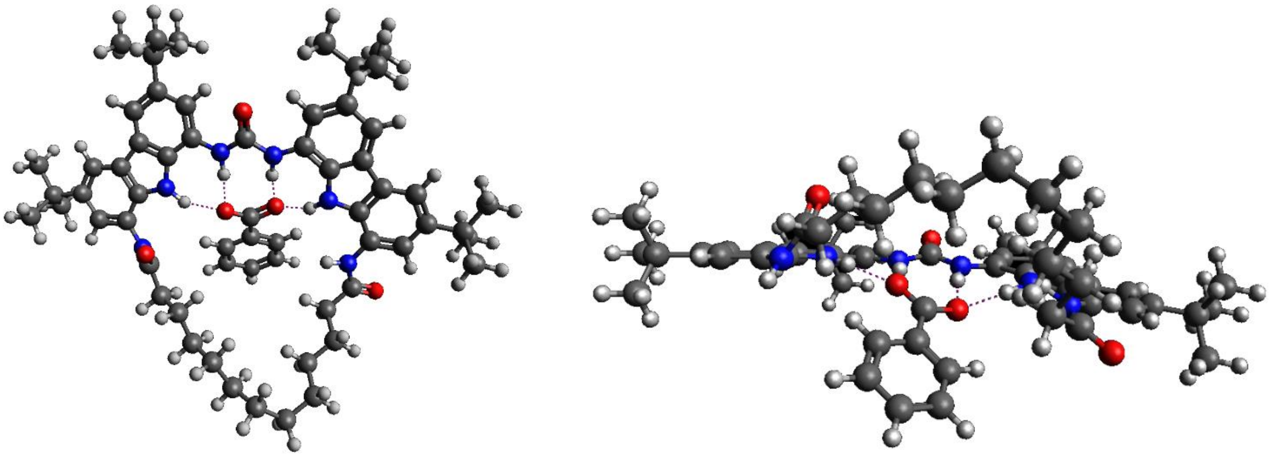

G = -1930905.78073 kcal/mol

Lowest energy conformation of receptor **MC011** with lactate anion

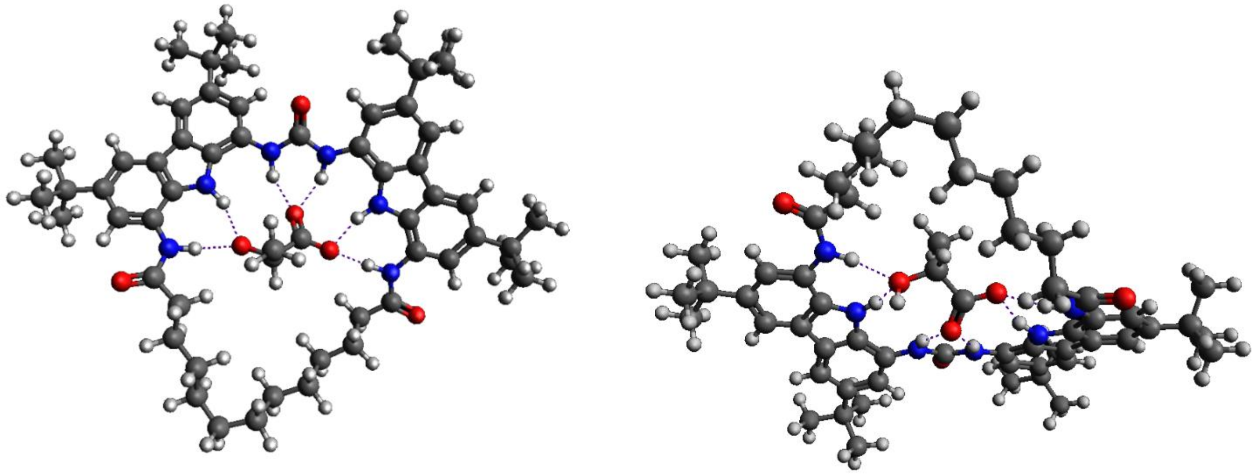

G = -1882455.59817 kcal/mol

Lowest energy conformation of receptor **MC011** with formate anion

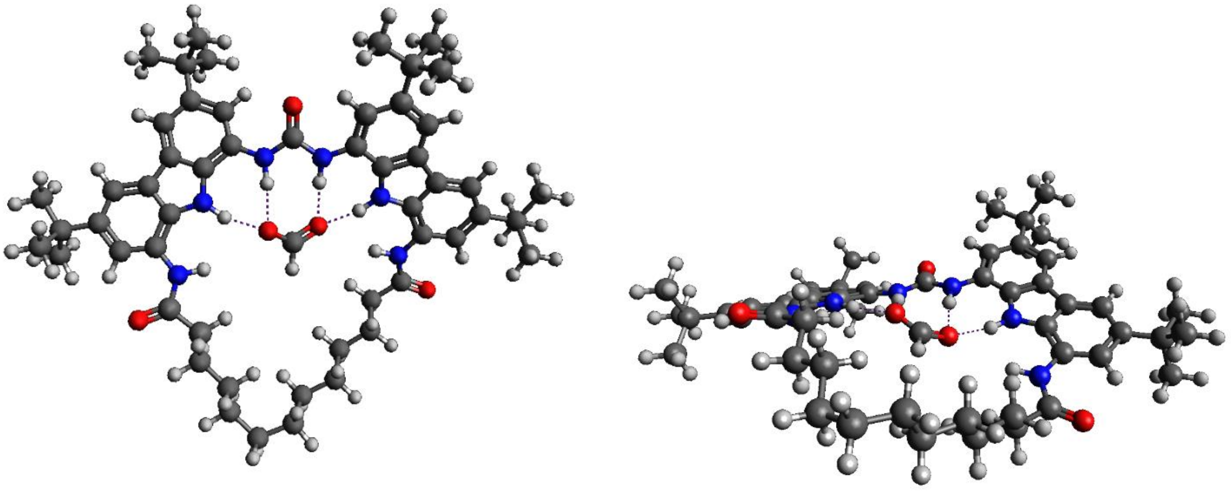

G = -1785865.82166 kcal/mol

Lowest energy conformation of receptor **MC012**

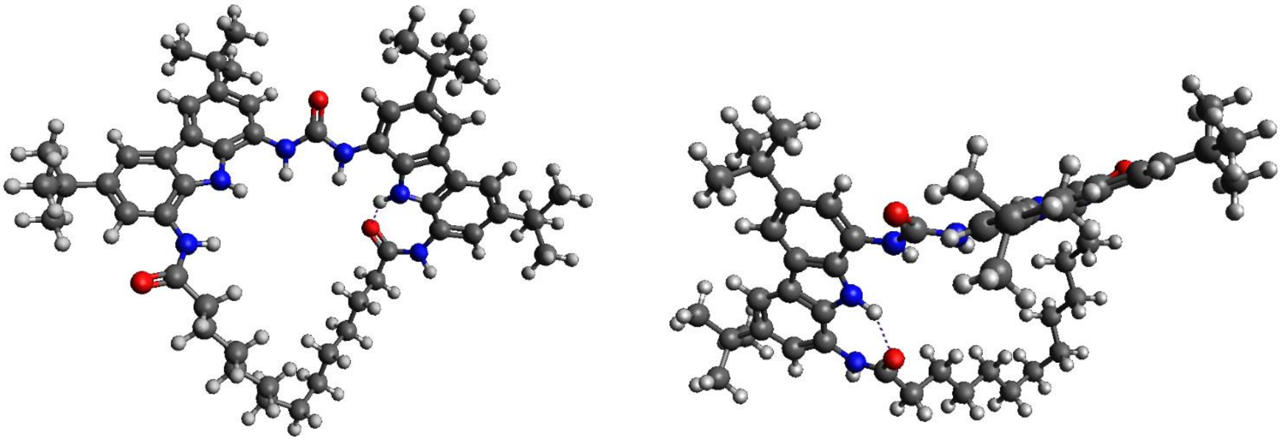

G = -1691674.08605 kcal/mol

Lowest energy conformation of receptor **MC012** with pivalate anion

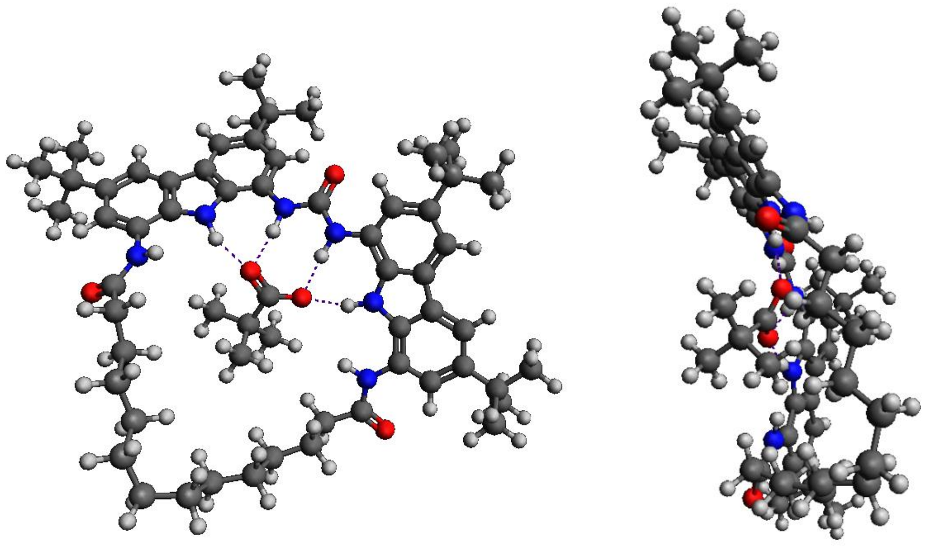

G = -1909252.26264 kcal/mol

Lowest energy conformation of receptor **MC012** with acetate anion

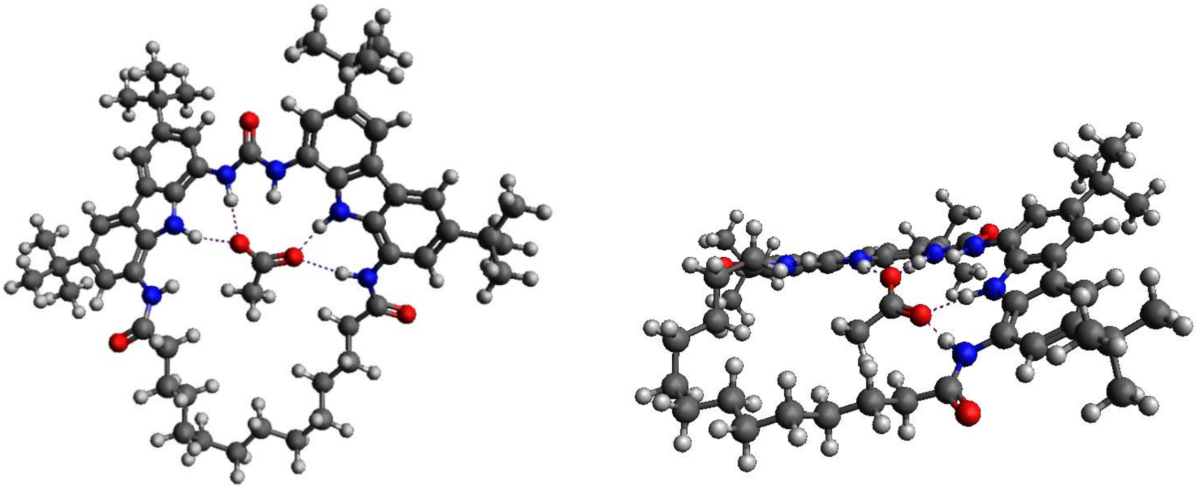

G = -1835225.73860 kcal/mol

Lowest energy conformation of receptor **MC012** with benzoate anion

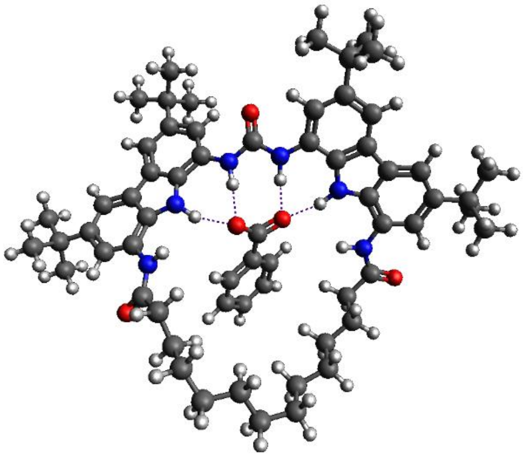

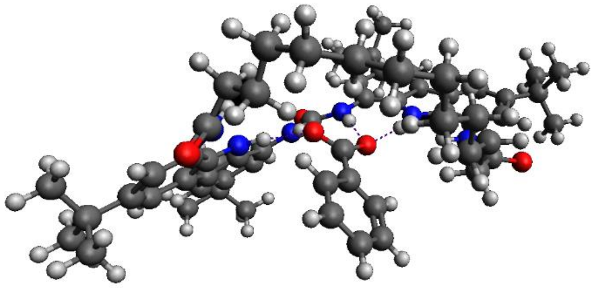

G = -1955585.03038 kcal/mol

Lowest energy conformation of receptor **MC012** with lactate anion

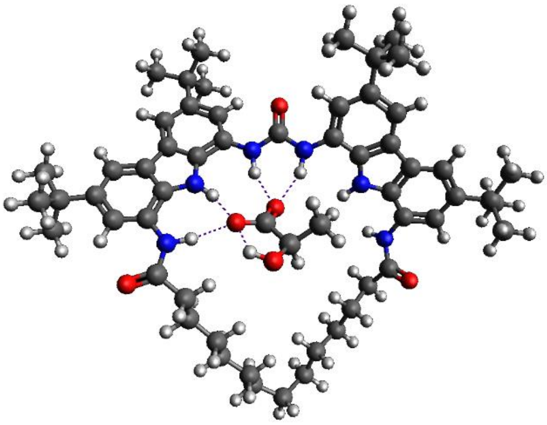

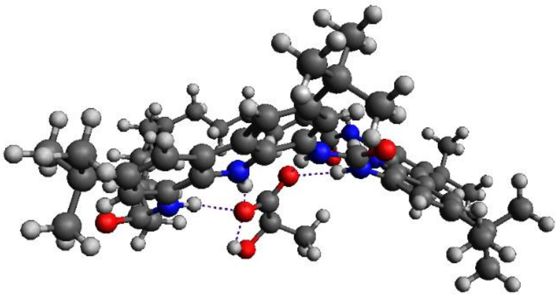

G = -1907129.15110 kcal/mol

Lowest energy conformation of receptor **MC012** with formate anion

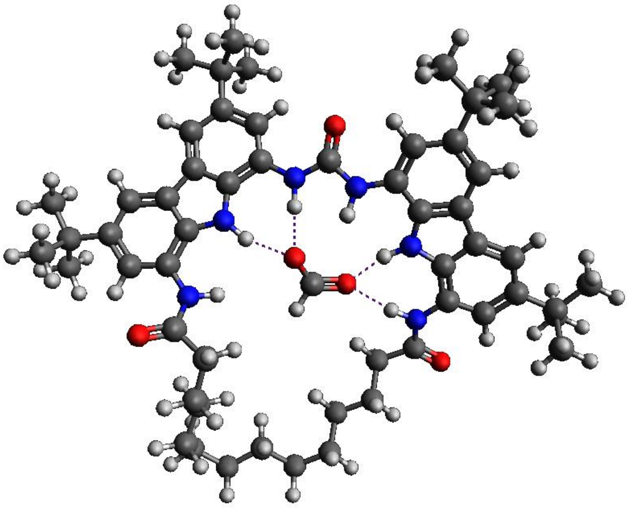

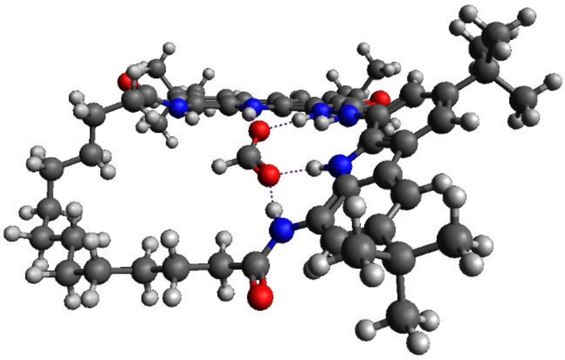

G = -1810543.68556 kcal/mol

Lowest energy conformation of receptor **MC013**

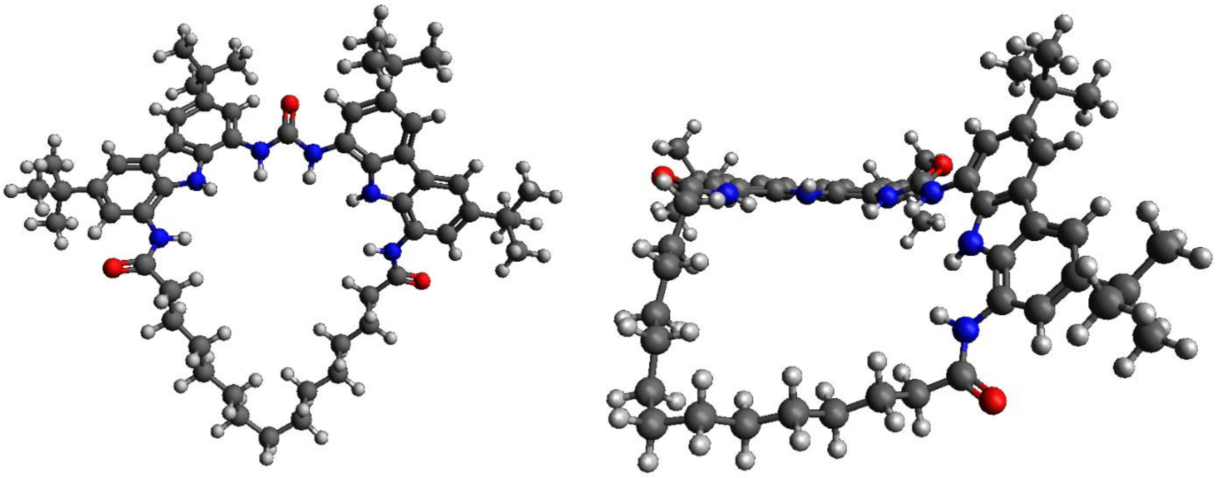

G = -1716352.25505 kcal/mol

Lowest energy conformation of receptor **MC013** with pivalate anion

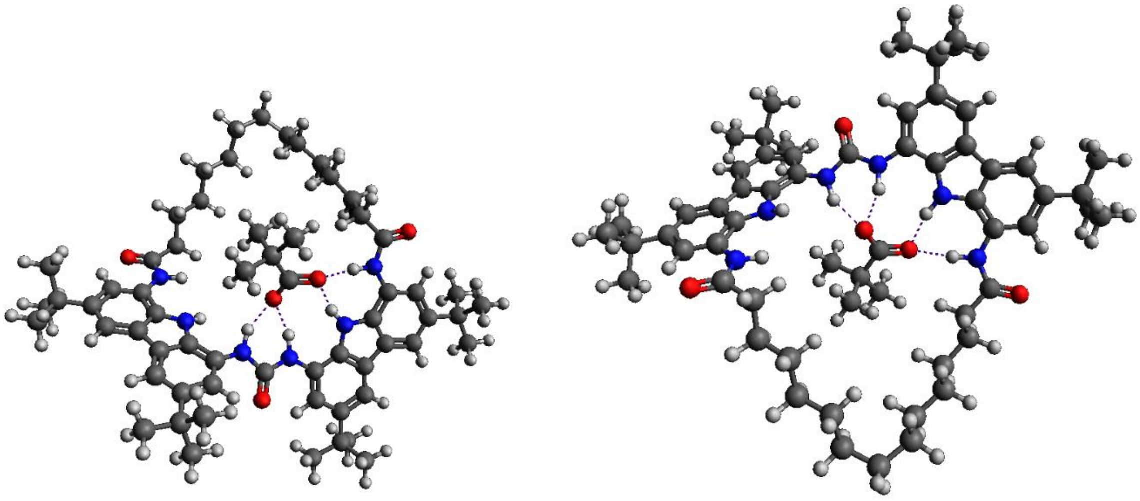

G = -1933934.70969 kcal/mol

Lowest energy conformation of receptor **MC013** with acetate anion

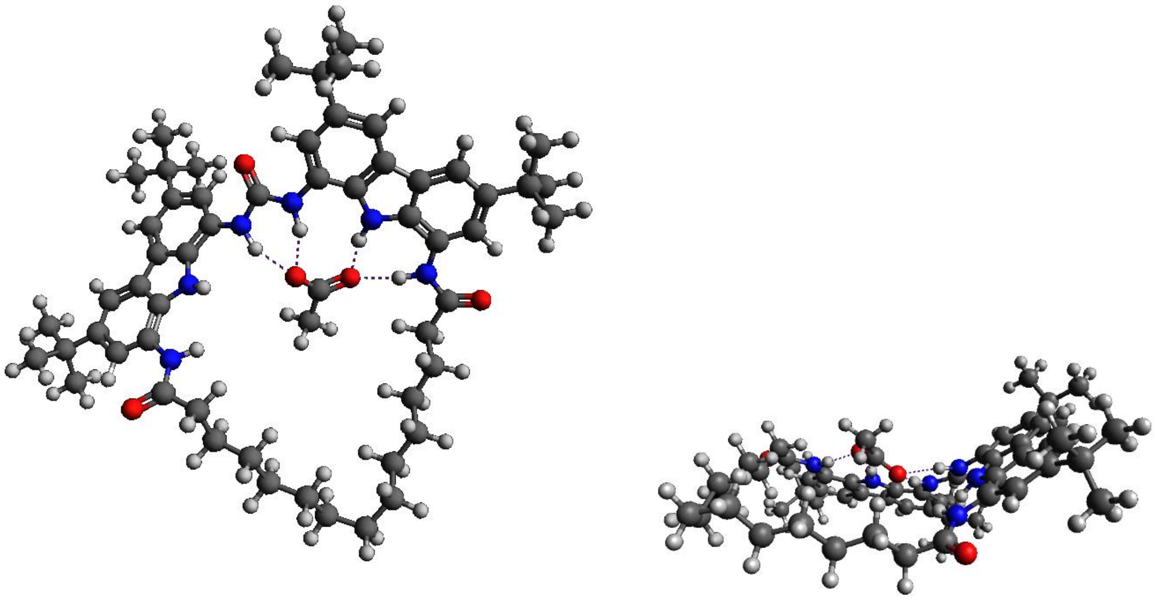

G = -1859903.77757 kcal/mol

Lowest energy conformation of receptor **MC013** with benzoate anion

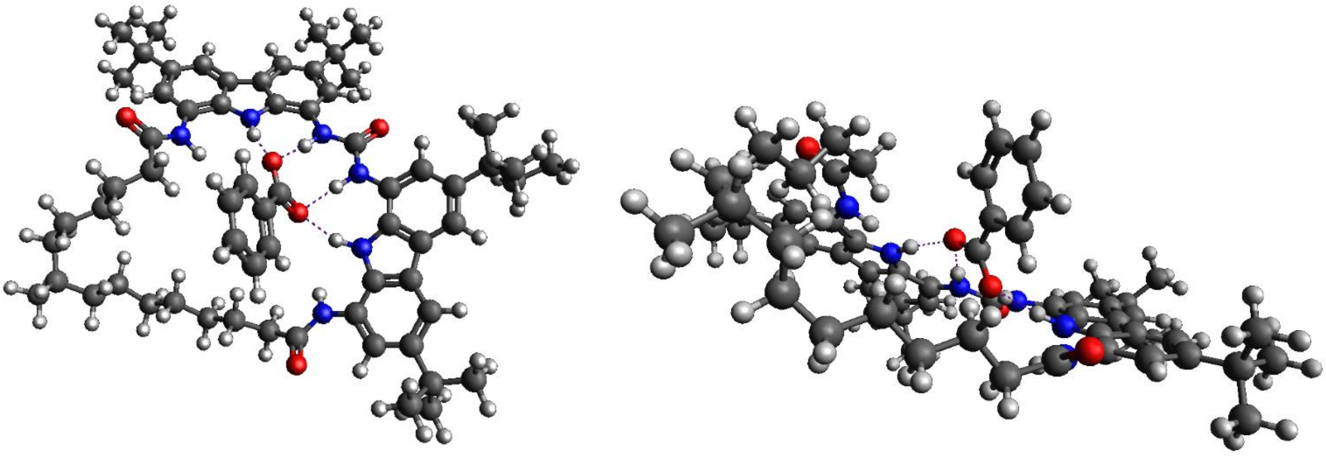

G = -1980264.91917 kcal/mol

Lowest energy conformation of receptor **MC013** with lactate anion

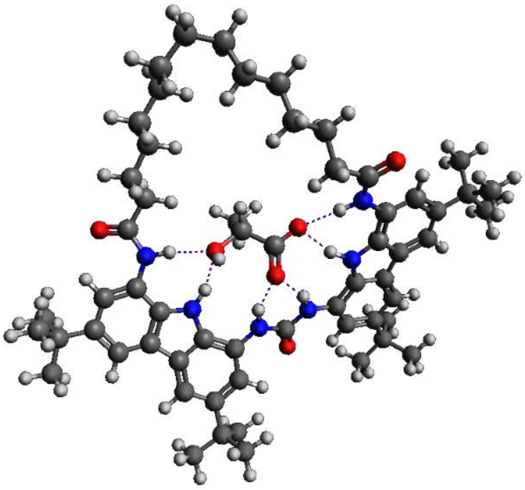

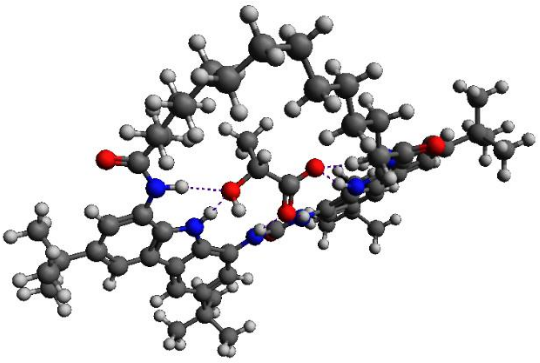

G = -1931811.69804 kcal/mol

Lowest energy conformation of receptor **MC013** with formate anion

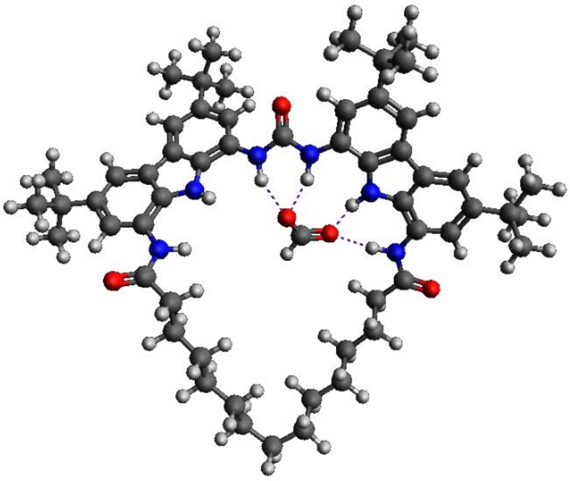

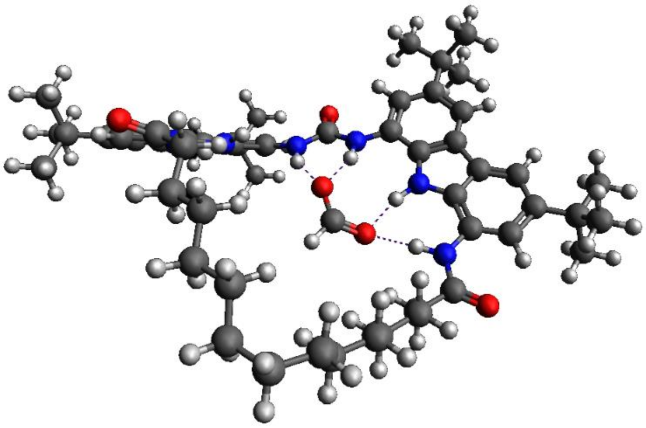

G = -1835222.20163 kcal/mol

Lowest energy conformation of receptor **MC014**

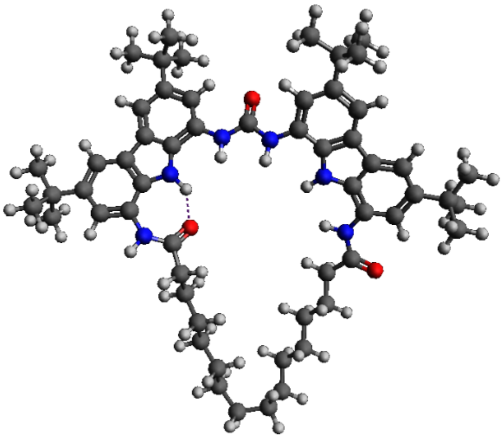

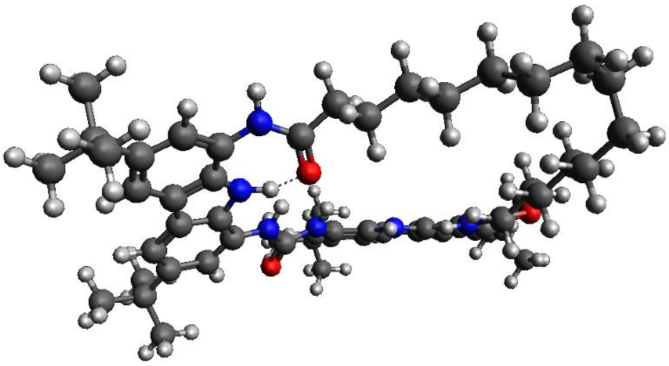

G = -1741028.91954 kcal/mol

Lowest energy conformation of receptor **MC014** with pivalate anion

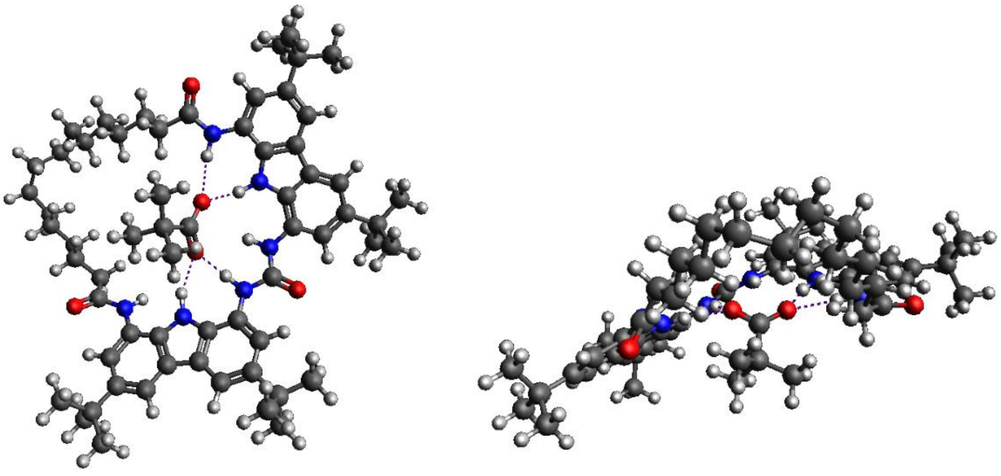

G = -1958610.17256 kcal/mol

Lowest energy conformation of receptor **MC014** with acetate anion

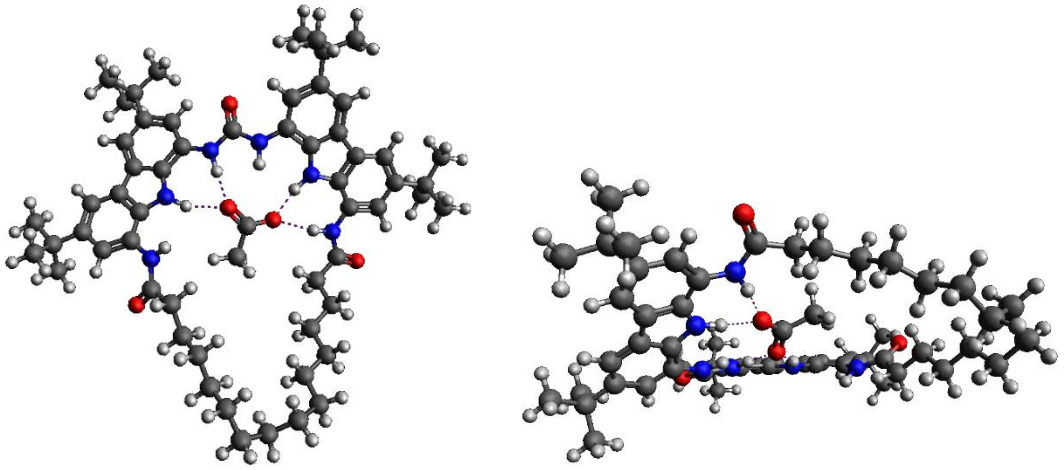

G = -1884581.51422 kcal/mol

Lowest energy conformation of receptor **MC014** with benzoate anion

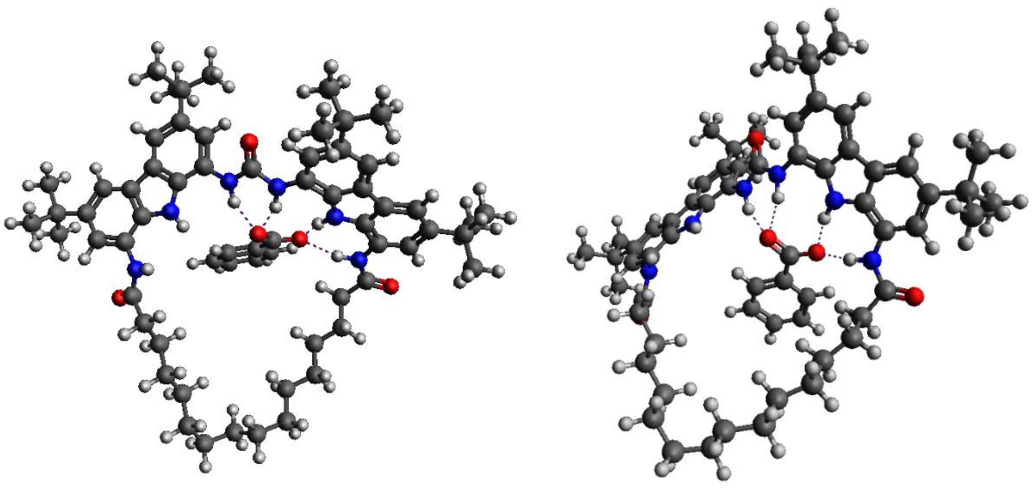

G = -2004943.68243 kcal/mol

Lowest energy conformation of receptor **MC014** with lactate anion

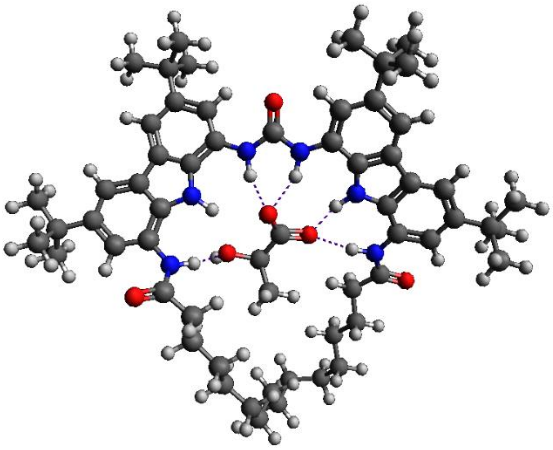

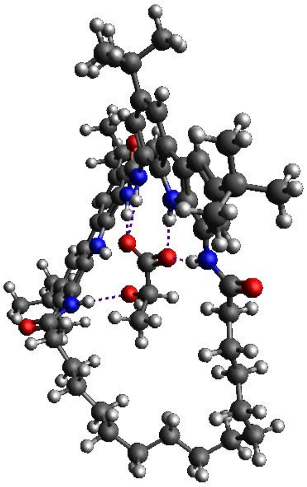

G = -1956485.72757 kcal/mol

Lowest energy conformation of receptor **MC014** with formate anion

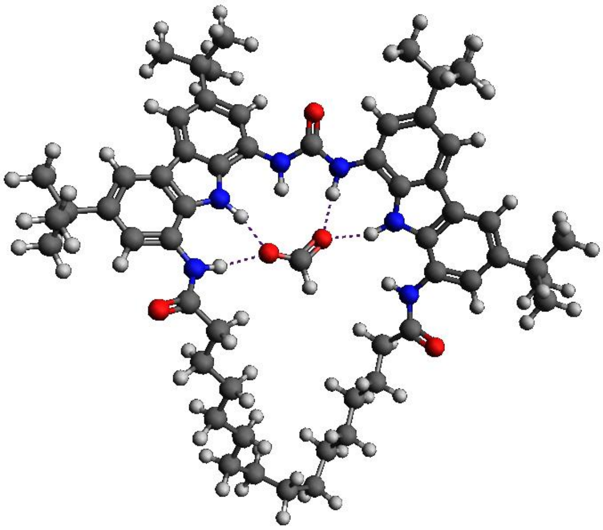

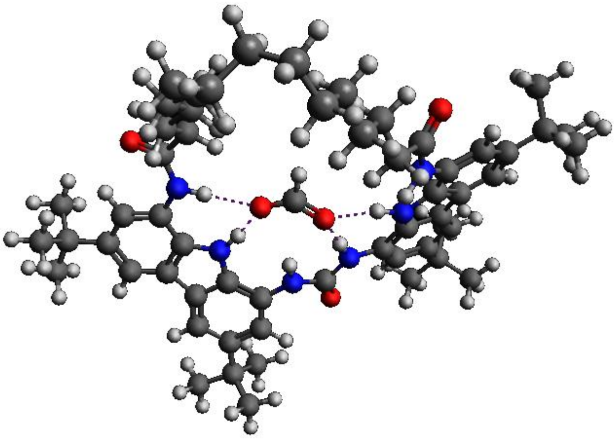

G = -1859899.50598 kcal/mol
